# Supplementary figures and images for: ORMDL3 restrains type I interferon signaling and anti-tumor immunity by promoting RIG-I degradation (part 1 of 3)
Source: eLife. 2025 Mar 24;13:RP101973. doi: 10.7554/eLife.101973 (PMC11932694; doi:10.7554/eLife.101973)

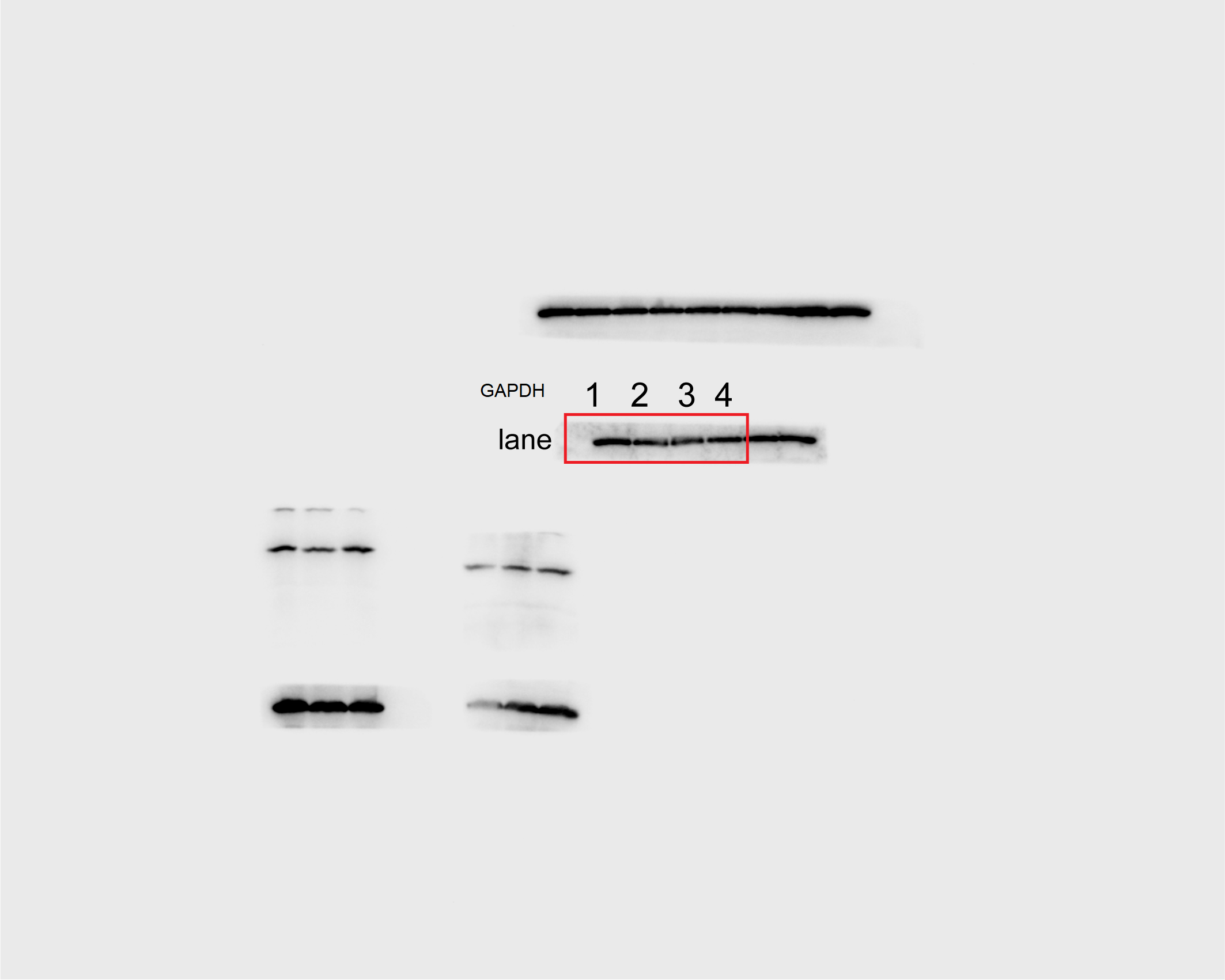

Supplement: Figure 1—source data 1. [file elife-101973-fig1-data1.zip › Figure 1-source data 1/Fig1B-labeled/GAPDH.tif]

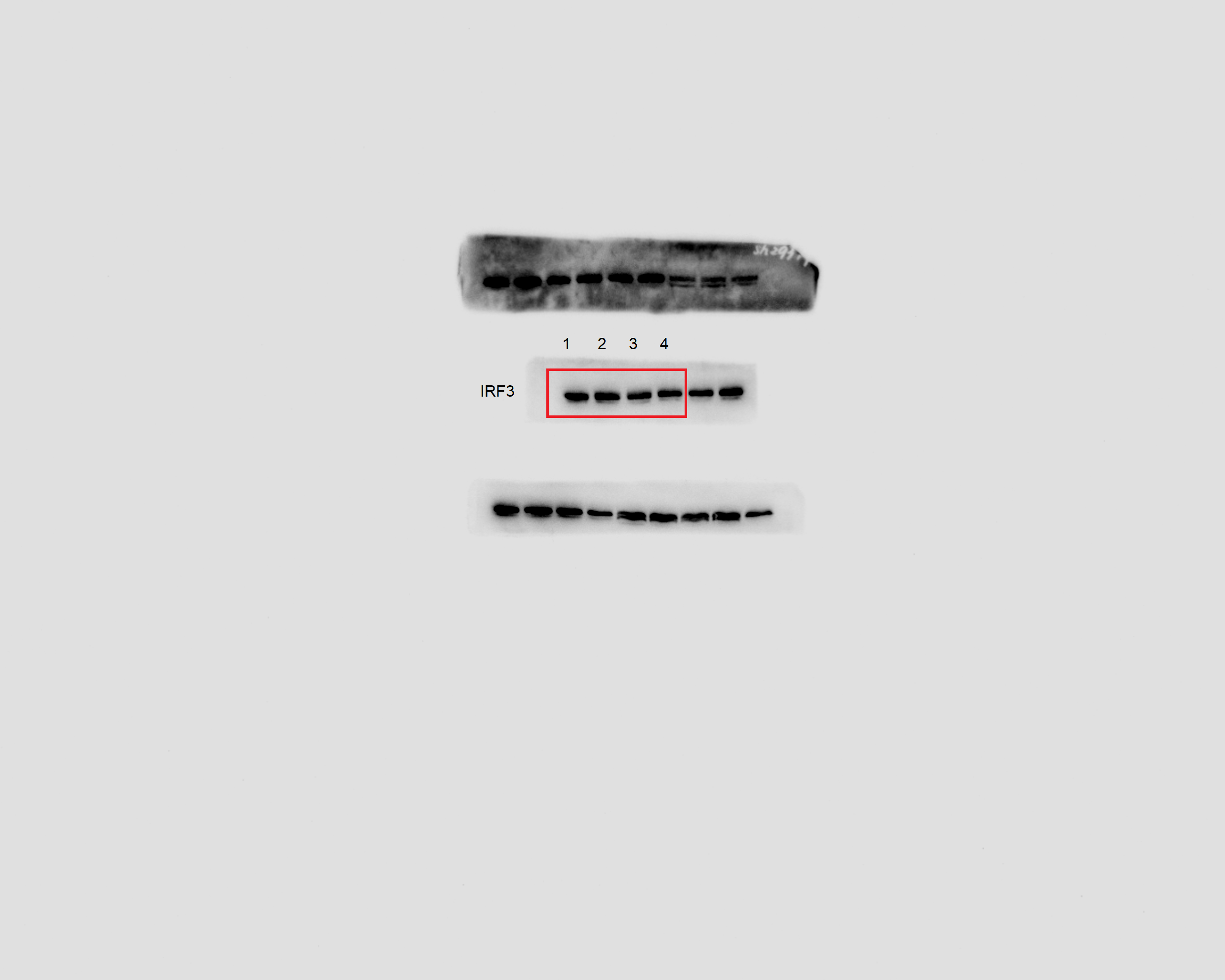

Supplement: Figure 1—source data 1. [file elife-101973-fig1-data1.zip › Figure 1-source data 1/Fig1B-labeled/IRF3.tif]

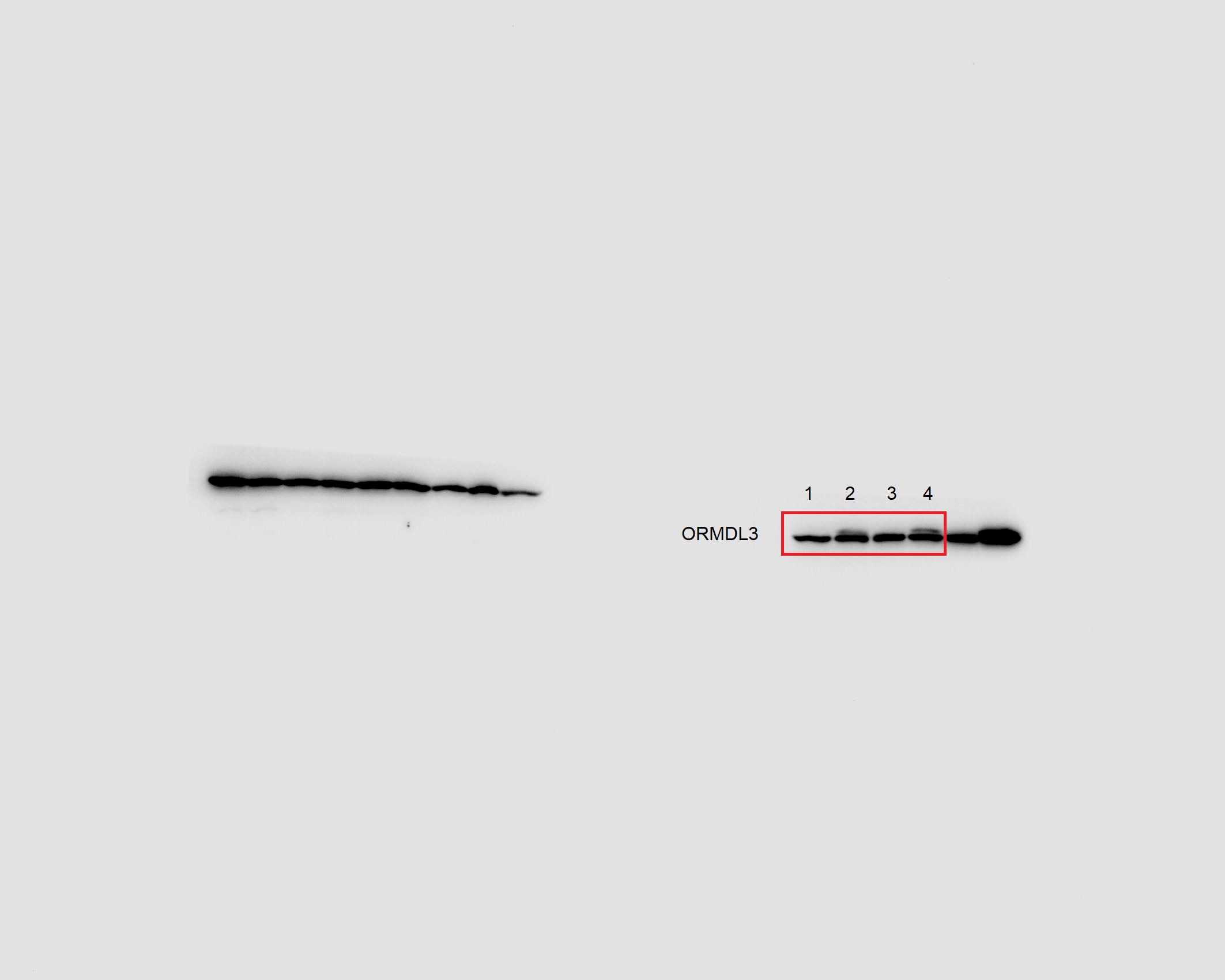

Supplement: Figure 1—source data 1. [file elife-101973-fig1-data1.zip › Figure 1-source data 1/Fig1B-labeled/ORMDL3.tif]

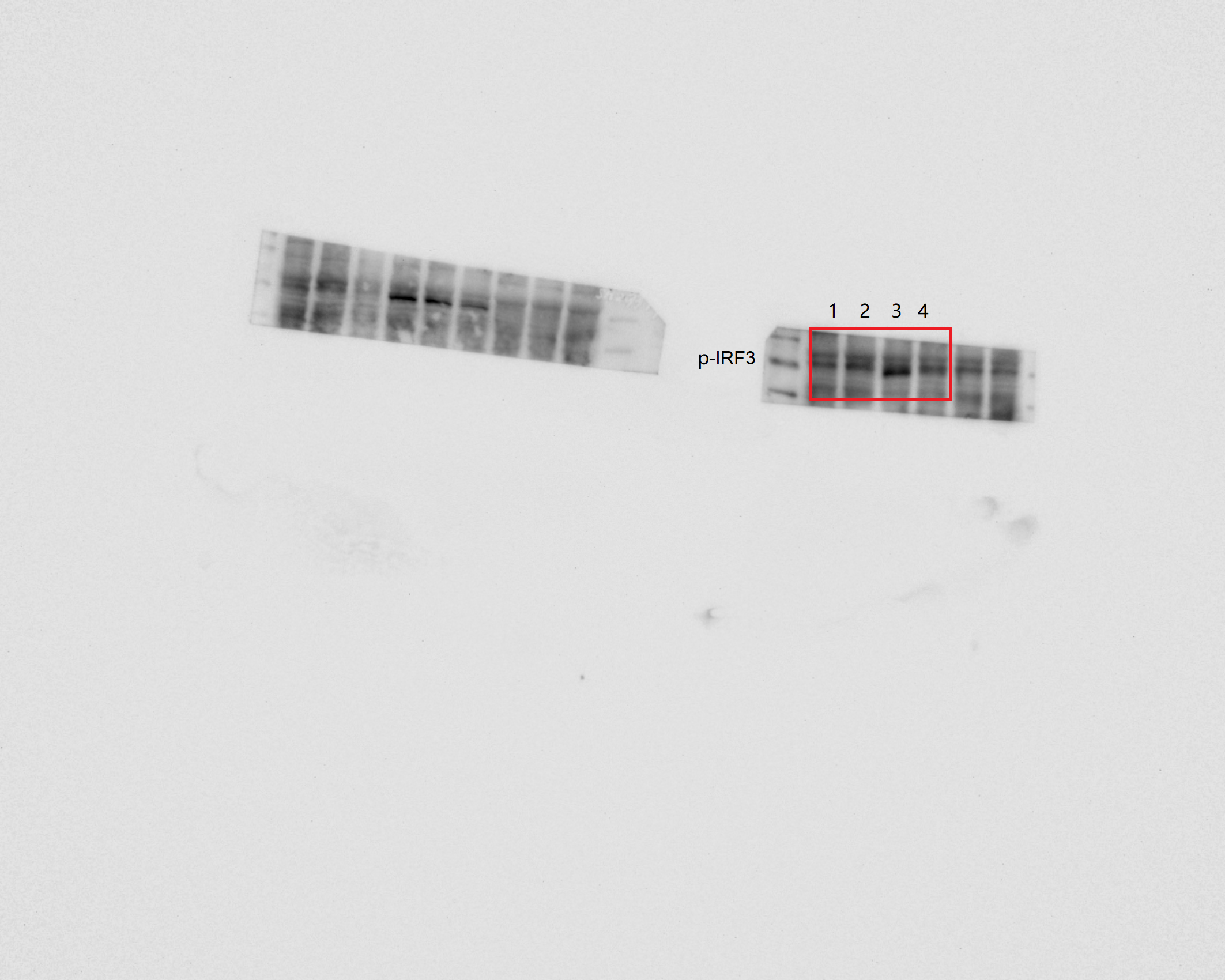

Supplement: Figure 1—source data 1. [file elife-101973-fig1-data1.zip › Figure 1-source data 1/Fig1B-labeled/p-IRF3.tif]

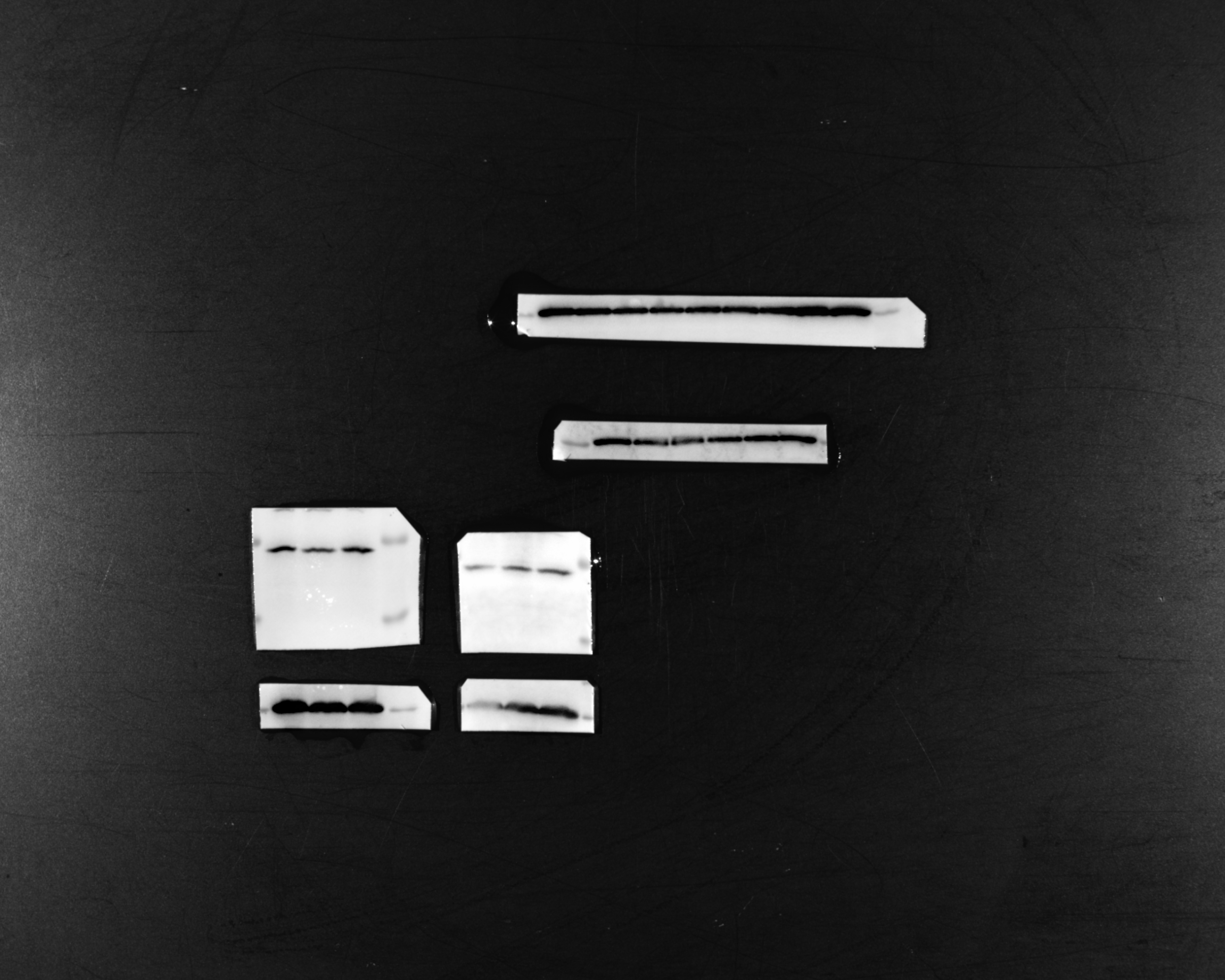

Supplement: Figure 1—source data 2. [file elife-101973-fig1-data2.zip › Figure 1-source data 2/figure 1B/GAPDH.jpg]

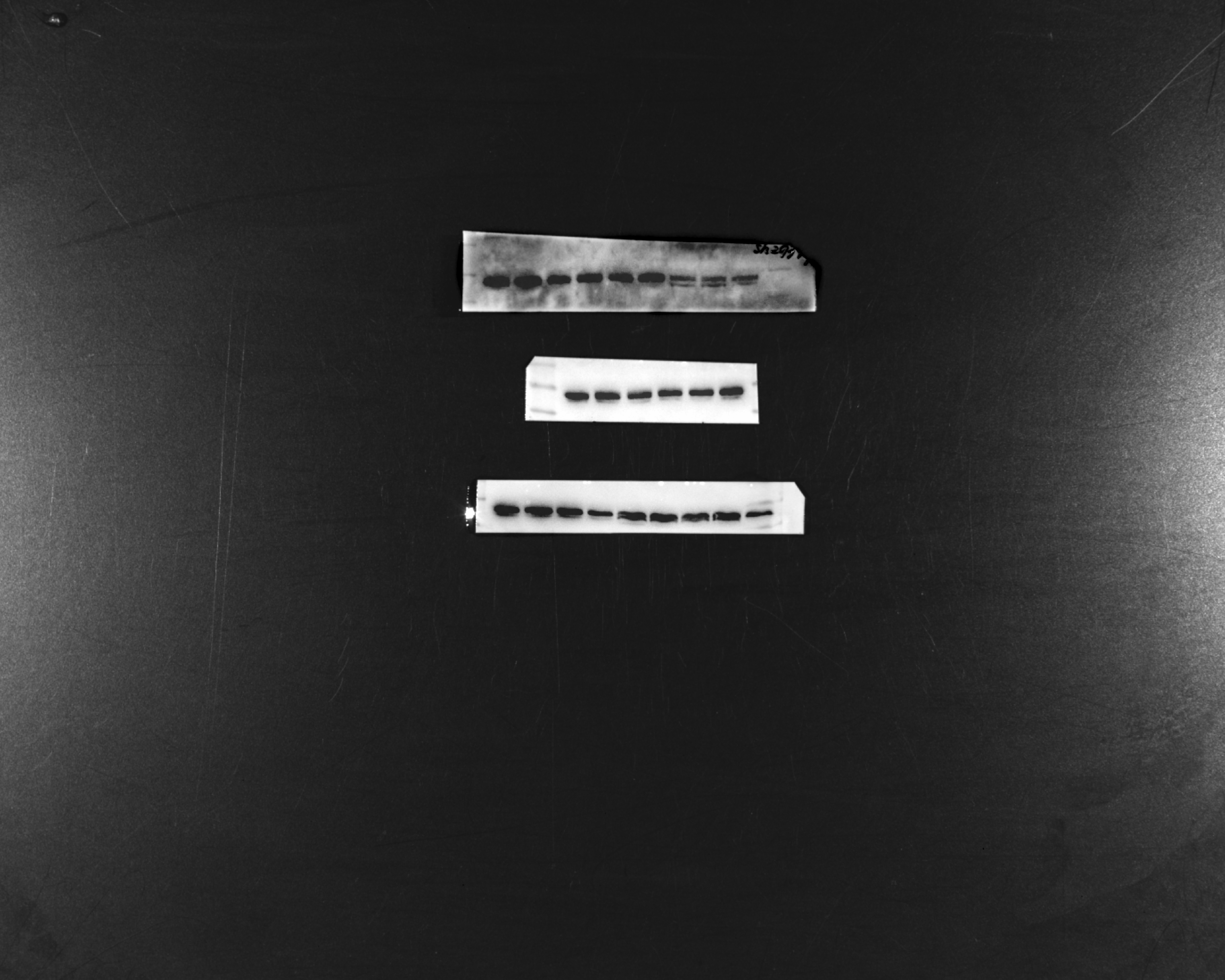

Supplement: Figure 1—source data 2. [file elife-101973-fig1-data2.zip › Figure 1-source data 2/figure 1B/IRF3.jpg]

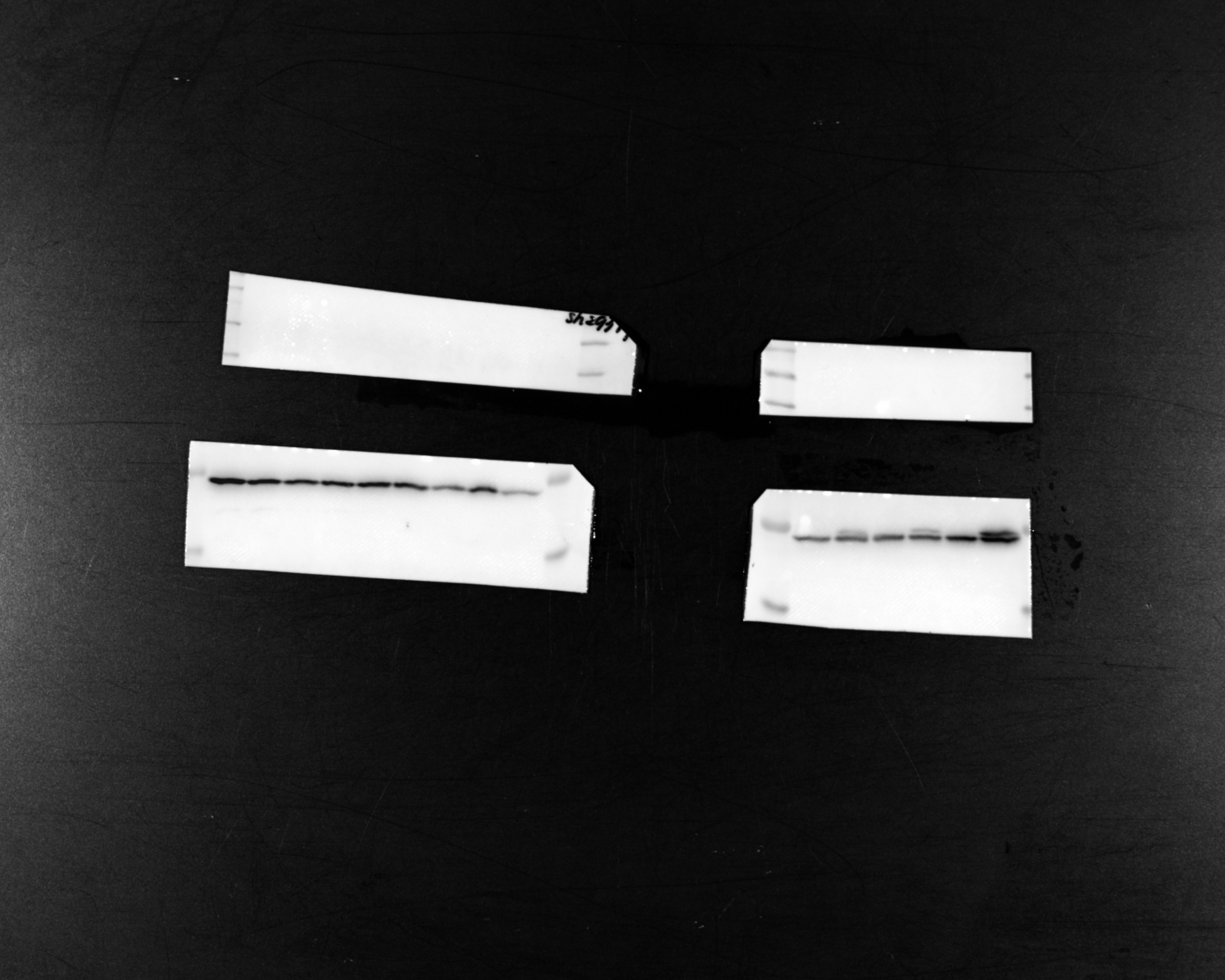

Supplement: Figure 1—source data 2. [file elife-101973-fig1-data2.zip › Figure 1-source data 2/figure 1B/ORMDL3.jpg]

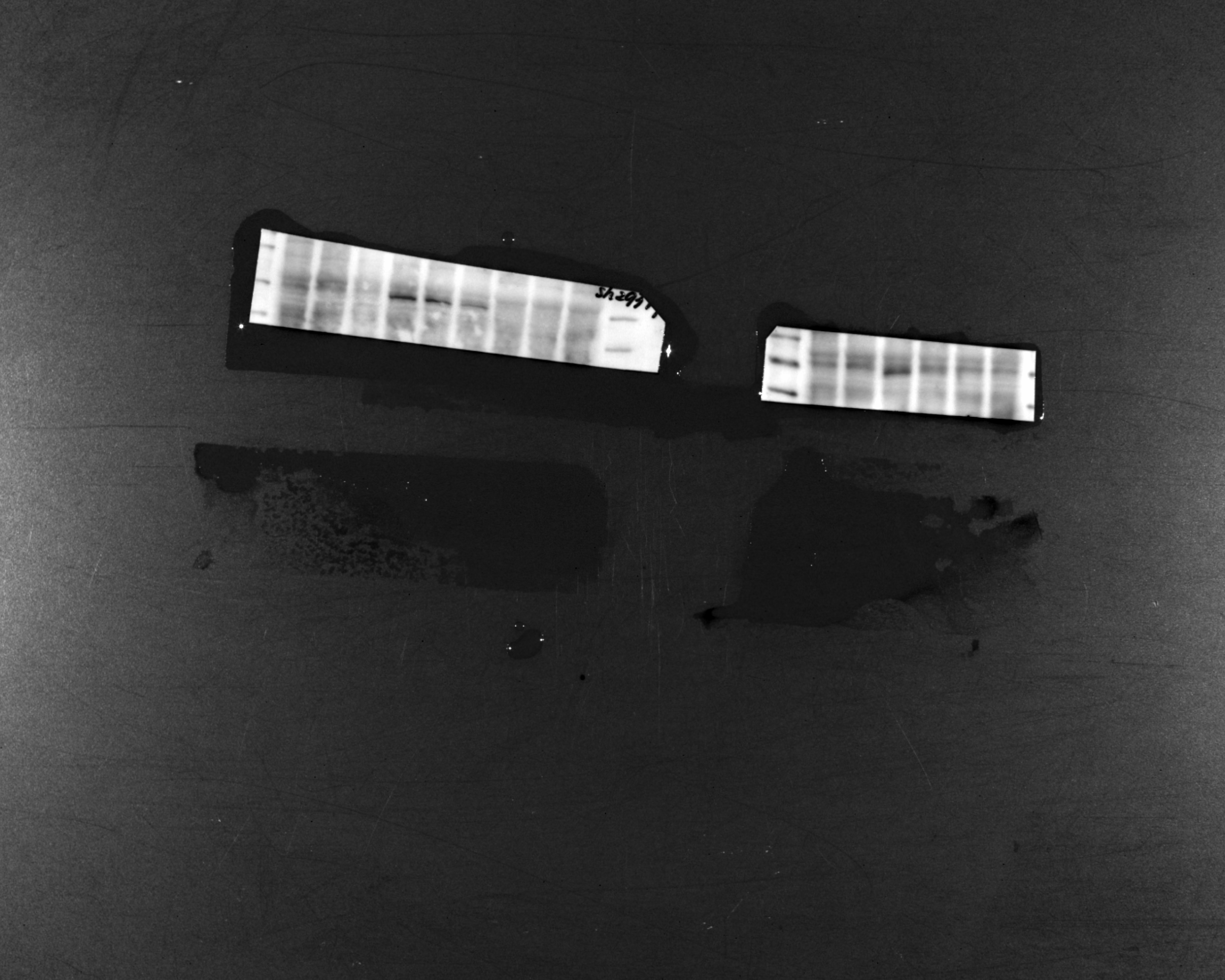

Supplement: Figure 1—source data 2. [file elife-101973-fig1-data2.zip › Figure 1-source data 2/figure 1B/p-IRF3.jpg]

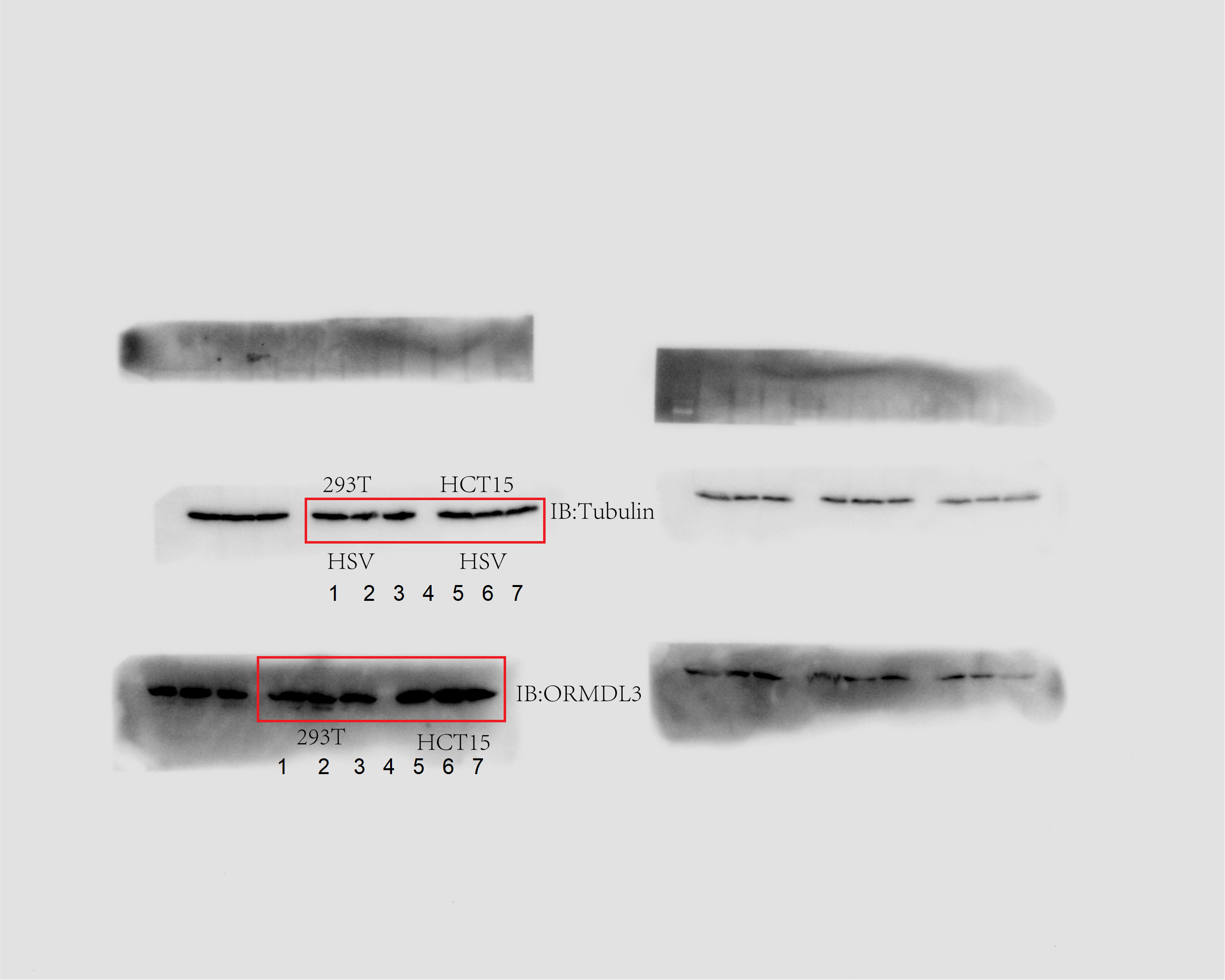

Supplement: Figure 1—figure supplement 1—source data 1. [file elife-101973-fig1-figsupp1-data1.zip › Figure 1-figure supplement 1-source data 1/Figure 1-figure supplement 1-labeled/HSV Figure 1-figure supplement 1A-labeled/293T HCT15 HSV Tubulin.jpeg]

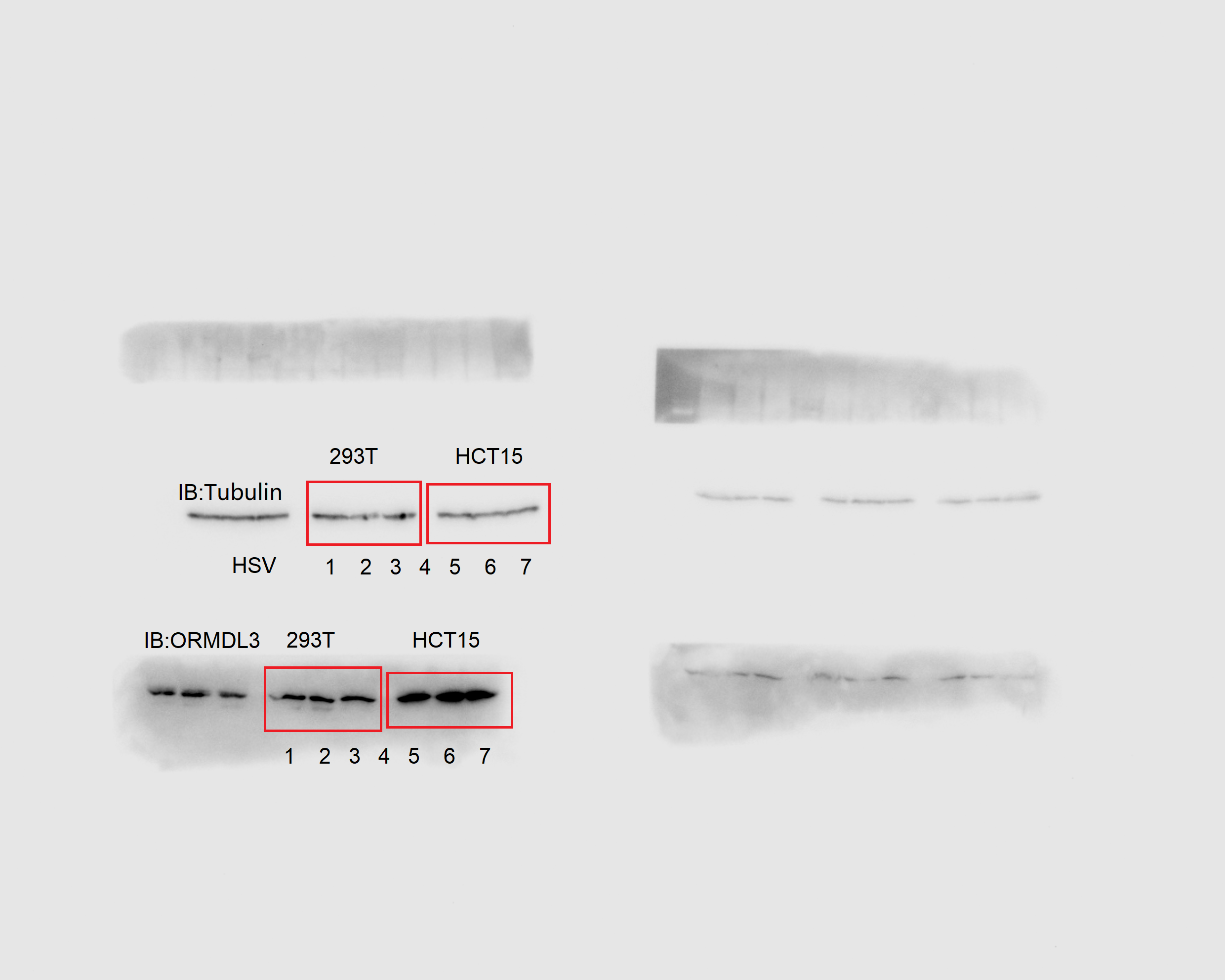

Supplement: Figure 1—figure supplement 1—source data 1. [file elife-101973-fig1-figsupp1-data1.zip › Figure 1-figure supplement 1-source data 1/Figure 1-figure supplement 1-labeled/HSV Figure 1-figure supplement 1A-labeled/293T HCT15 short exposure.jpeg]

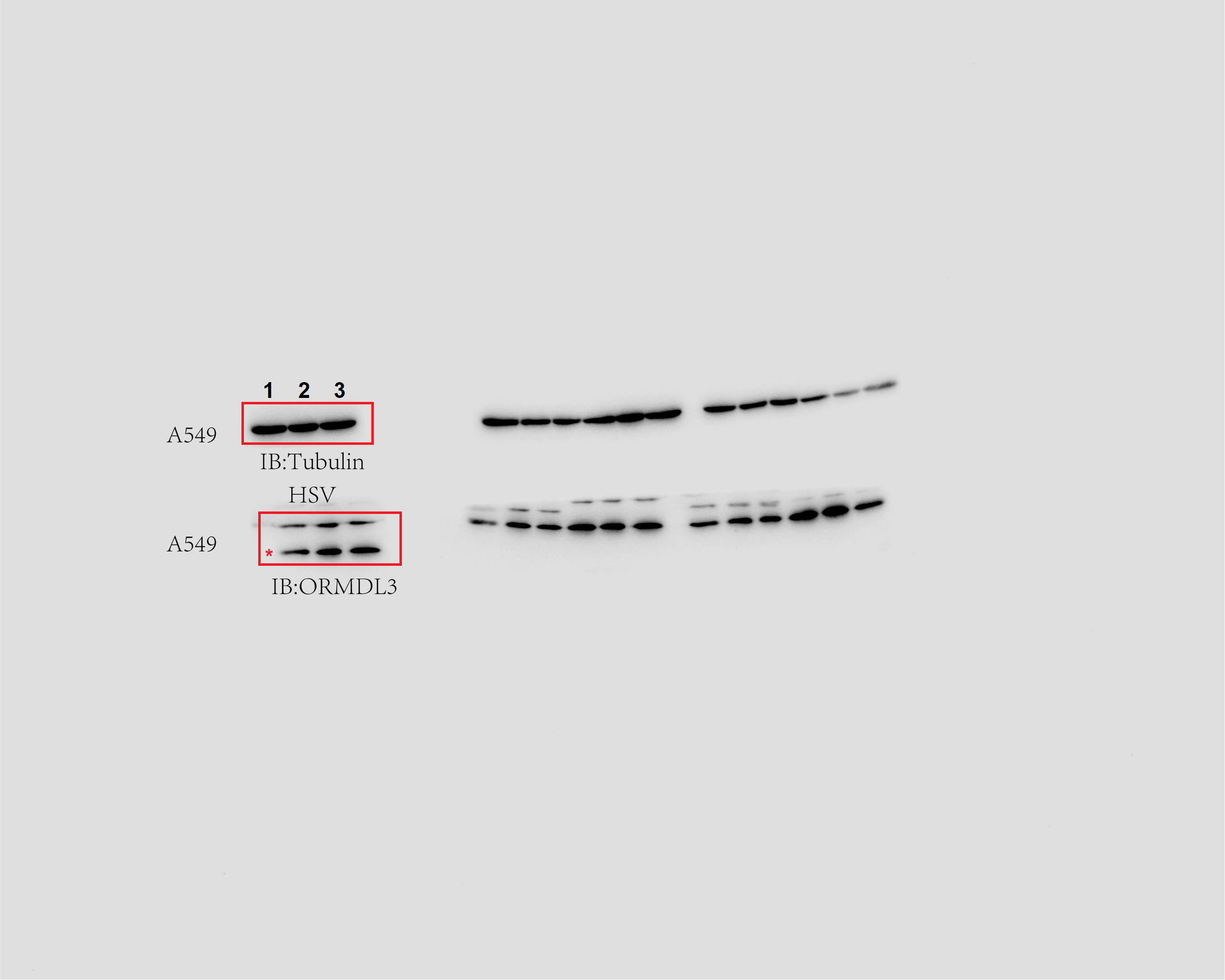

Supplement: Figure 1—figure supplement 1—source data 1. [file elife-101973-fig1-figsupp1-data1.zip › Figure 1-figure supplement 1-source data 1/Figure 1-figure supplement 1-labeled/HSV Figure 1-figure supplement 1A-labeled/A549 HSV.jpeg]

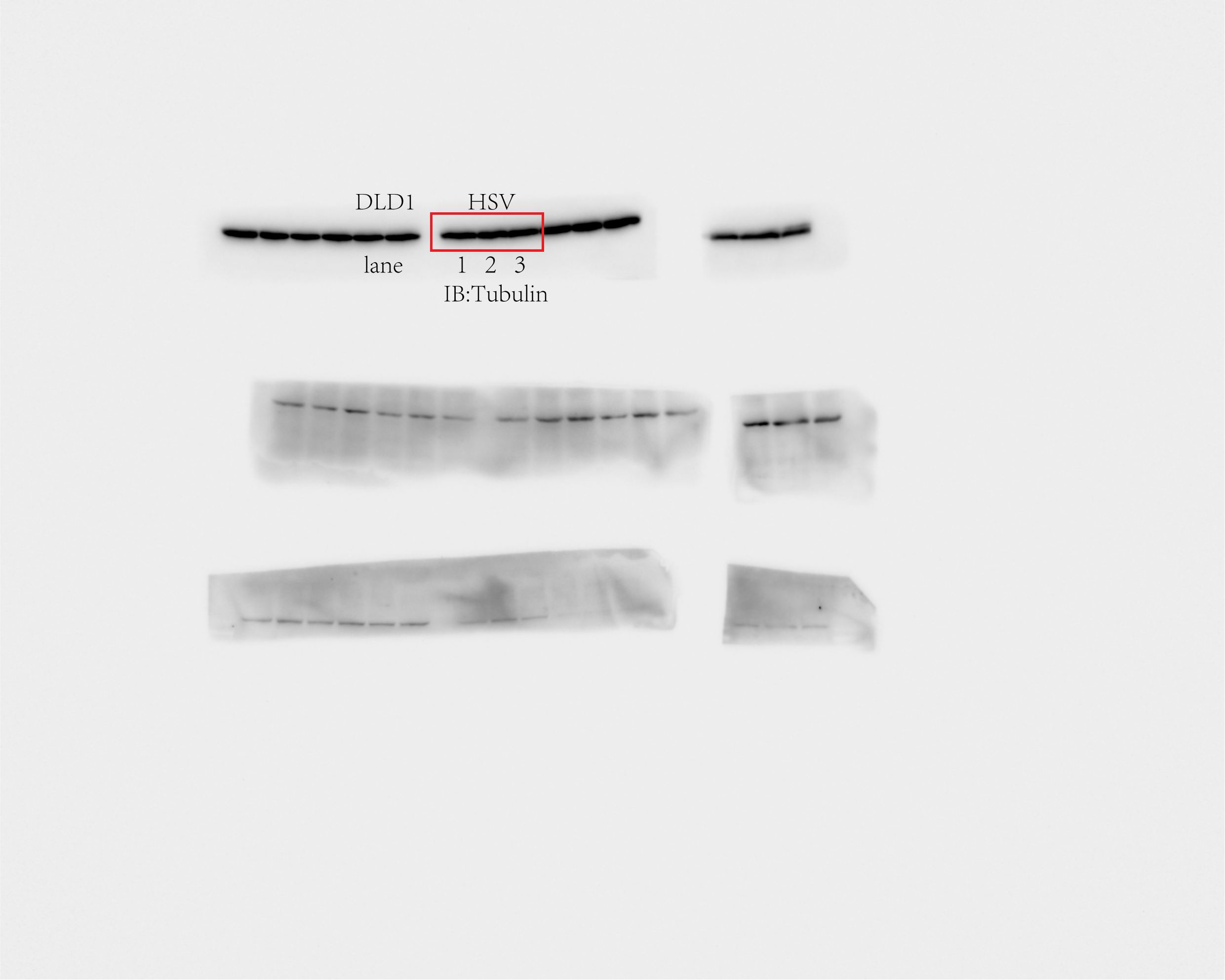

Supplement: Figure 1—figure supplement 1—source data 1. [file elife-101973-fig1-figsupp1-data1.zip › Figure 1-figure supplement 1-source data 1/Figure 1-figure supplement 1-labeled/HSV Figure 1-figure supplement 1A-labeled/DLD1 HSV Tubulin.jpeg]

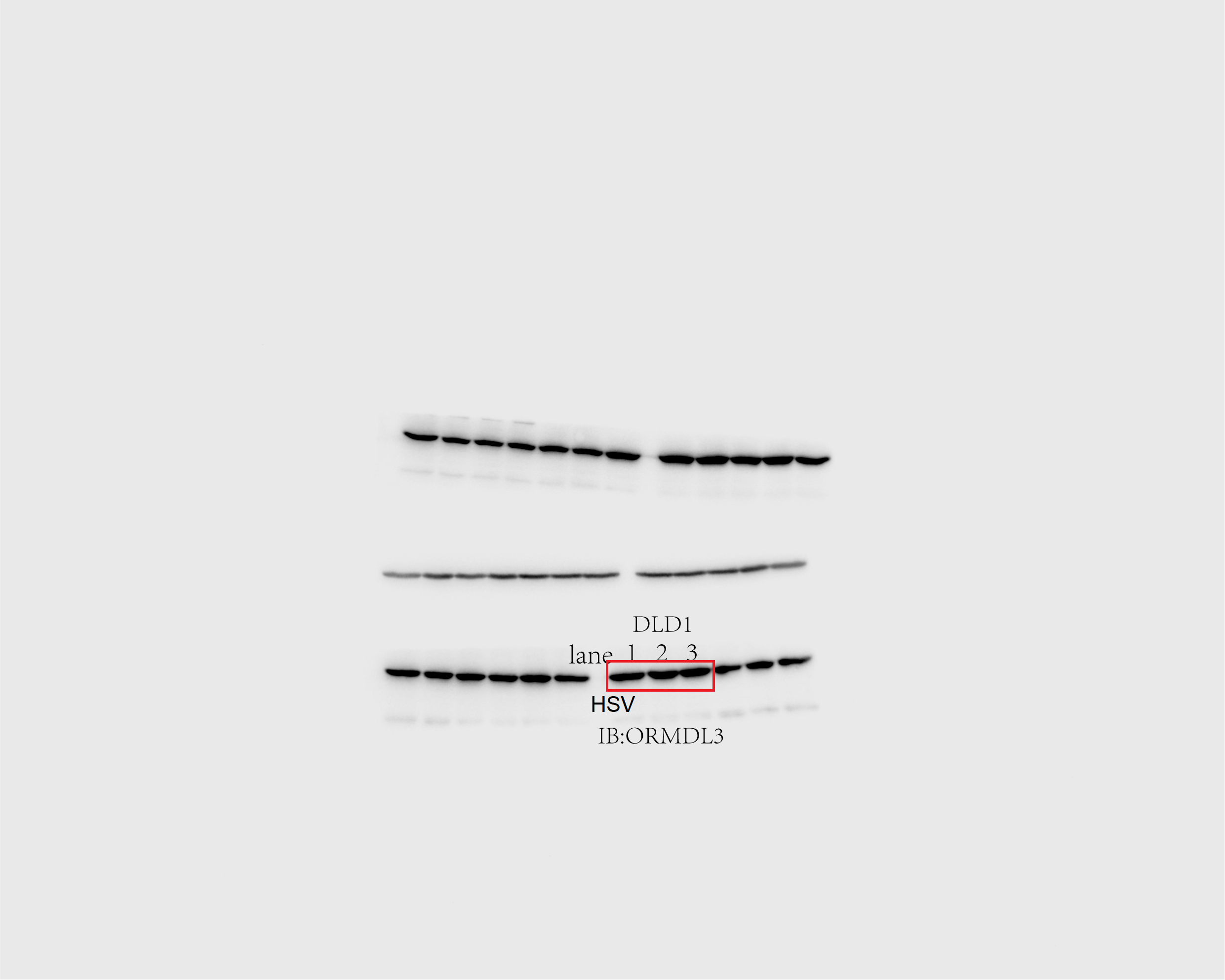

Supplement: Figure 1—figure supplement 1—source data 1. [file elife-101973-fig1-figsupp1-data1.zip › Figure 1-figure supplement 1-source data 1/Figure 1-figure supplement 1-labeled/HSV Figure 1-figure supplement 1A-labeled/DLD1 HSV.jpeg]

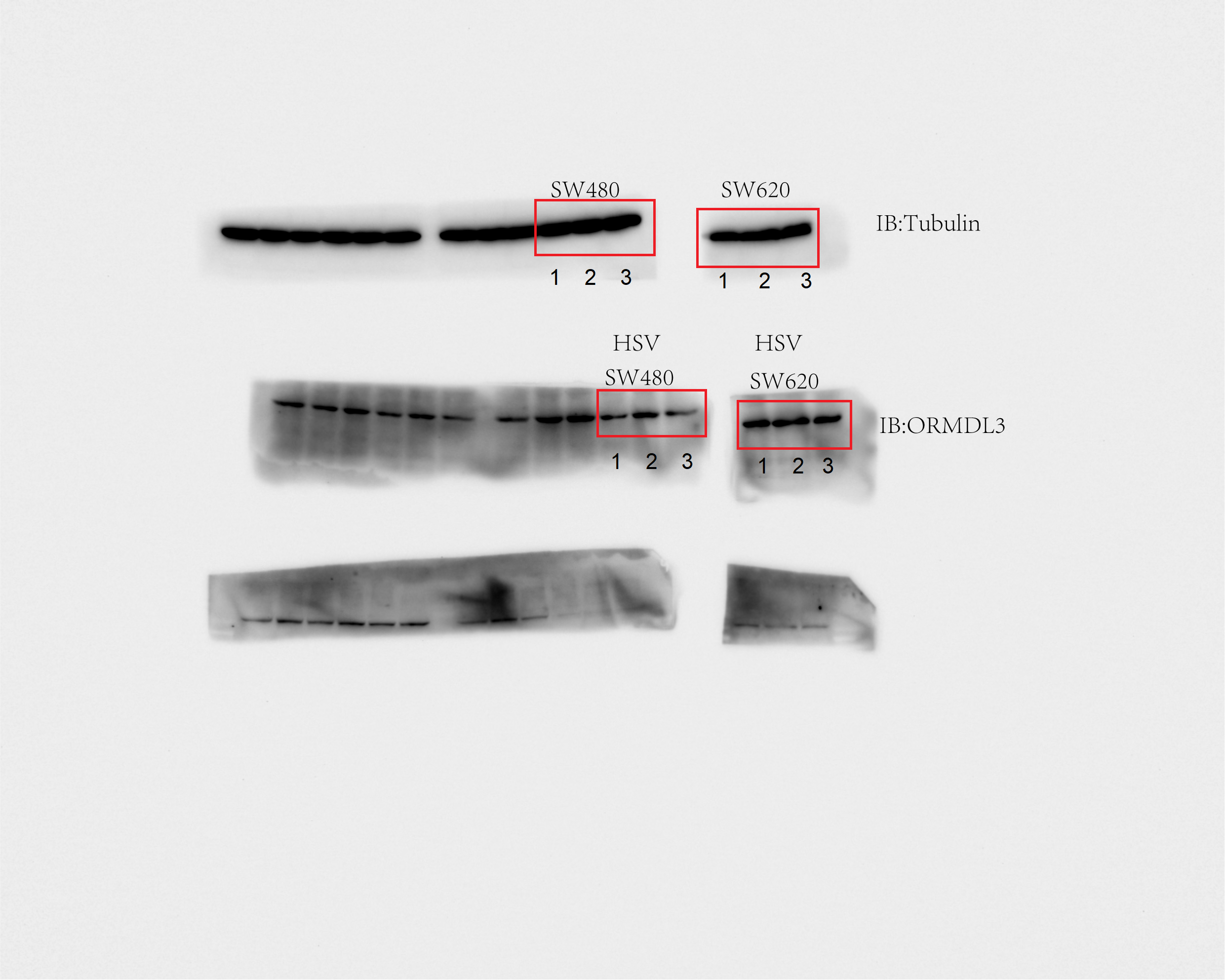

Supplement: Figure 1—figure supplement 1—source data 1. [file elife-101973-fig1-figsupp1-data1.zip › Figure 1-figure supplement 1-source data 1/Figure 1-figure supplement 1-labeled/HSV Figure 1-figure supplement 1A-labeled/SW480 SW620 HSV.jpeg]

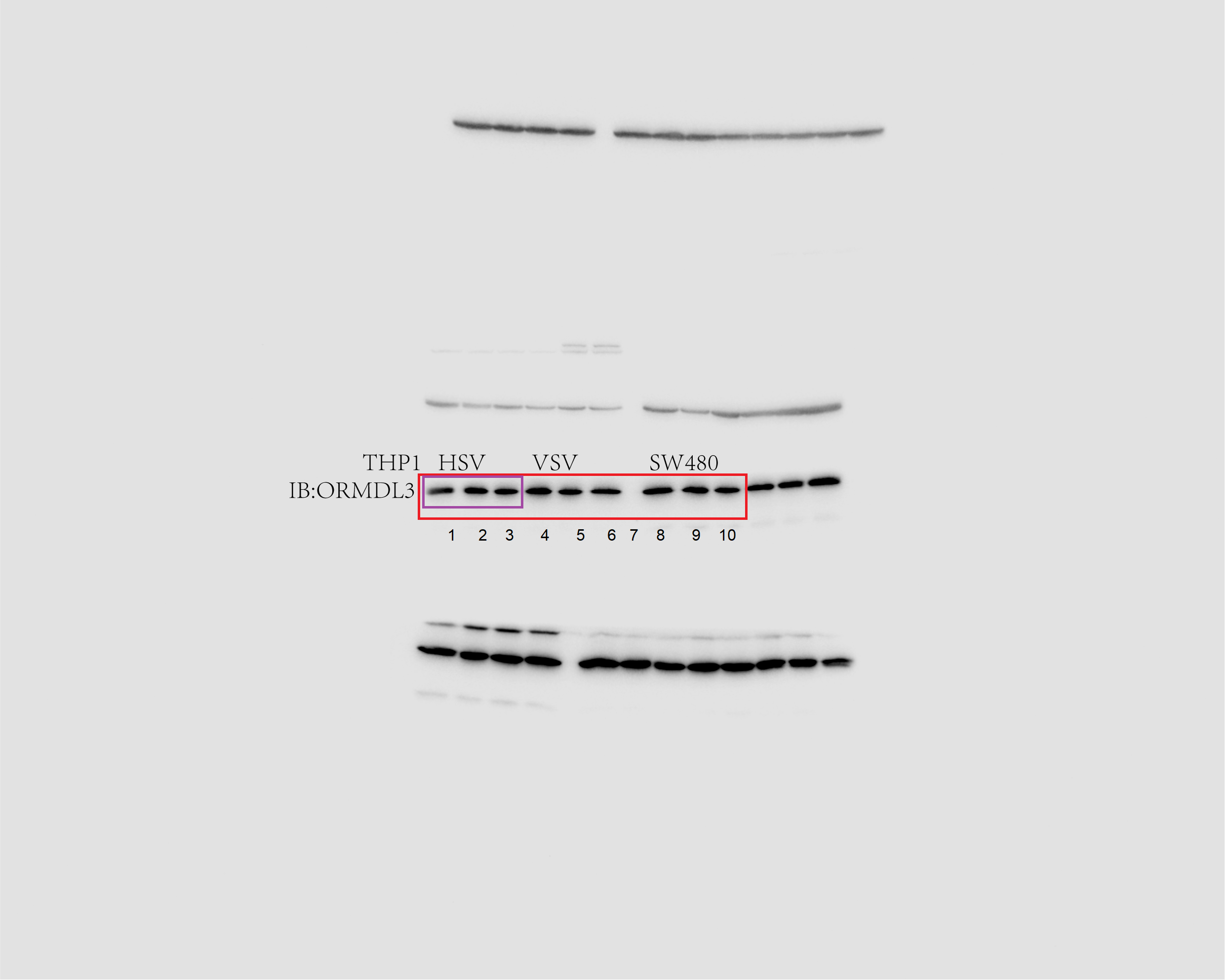

Supplement: Figure 1—figure supplement 1—source data 1. [file elife-101973-fig1-figsupp1-data1.zip › Figure 1-figure supplement 1-source data 1/Figure 1-figure supplement 1-labeled/HSV Figure 1-figure supplement 1A-labeled/THP1 HSV .jpeg]

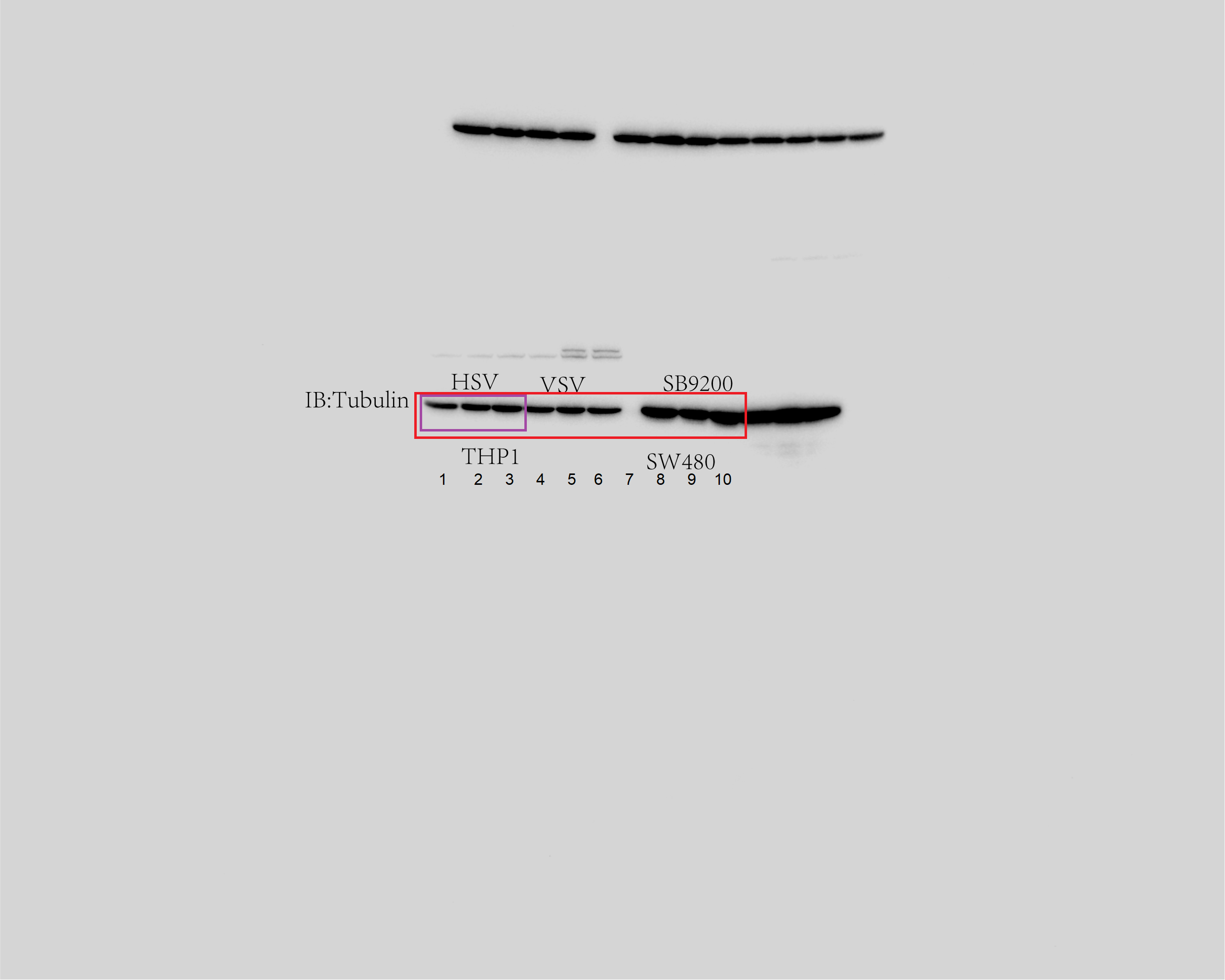

Supplement: Figure 1—figure supplement 1—source data 1. [file elife-101973-fig1-figsupp1-data1.zip › Figure 1-figure supplement 1-source data 1/Figure 1-figure supplement 1-labeled/HSV Figure 1-figure supplement 1A-labeled/THP1 HSV Tubulin .jpeg]

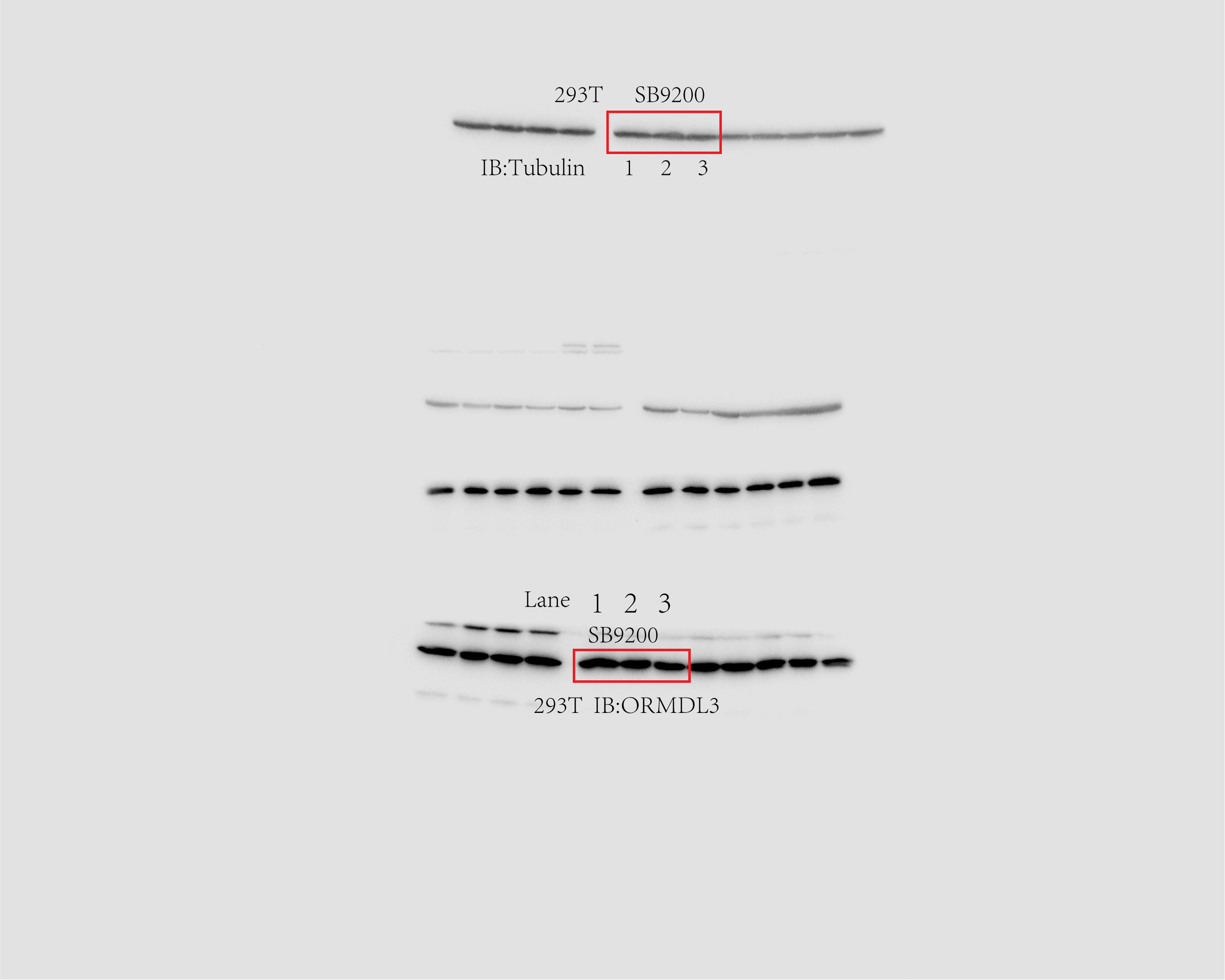

Supplement: Figure 1—figure supplement 1—source data 1. [file elife-101973-fig1-figsupp1-data1.zip › Figure 1-figure supplement 1-source data 1/Figure 1-figure supplement 1-labeled/SB9200 Figure 1-figure supplement 1C-labeled/293T SB9200.jpeg]

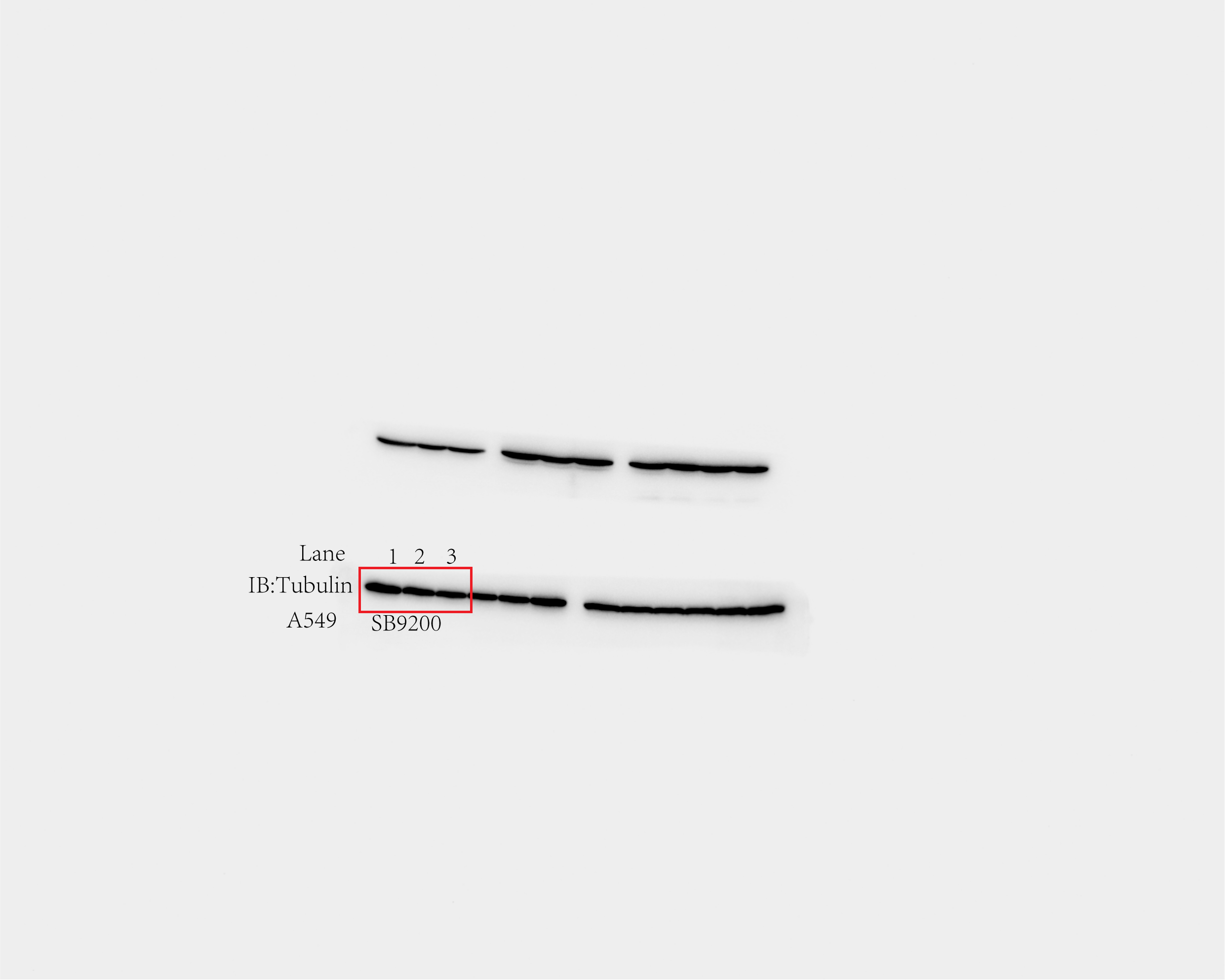

Supplement: Figure 1—figure supplement 1—source data 1. [file elife-101973-fig1-figsupp1-data1.zip › Figure 1-figure supplement 1-source data 1/Figure 1-figure supplement 1-labeled/SB9200 Figure 1-figure supplement 1C-labeled/A549 SB9200 Tubulin.tif]

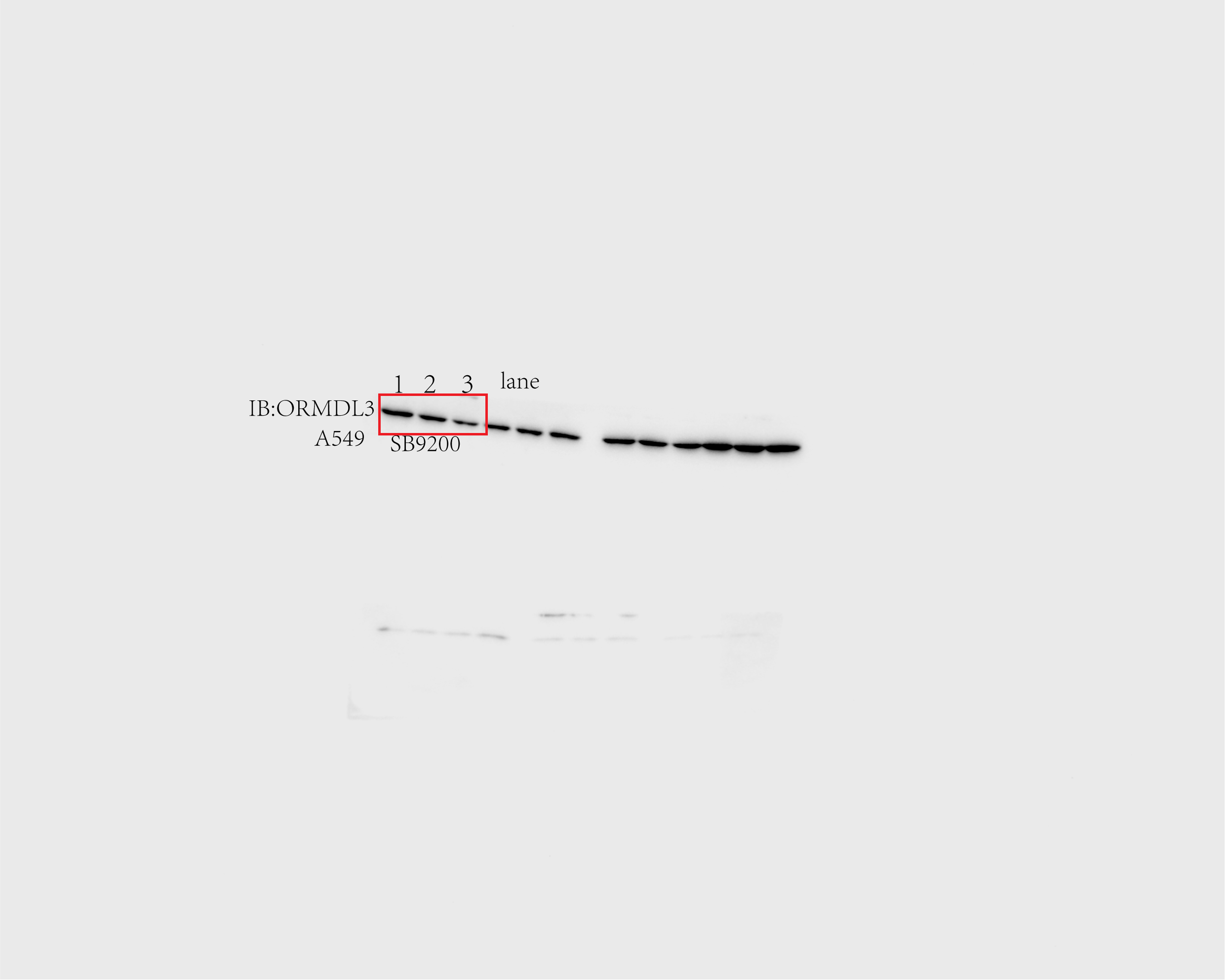

Supplement: Figure 1—figure supplement 1—source data 1. [file elife-101973-fig1-figsupp1-data1.zip › Figure 1-figure supplement 1-source data 1/Figure 1-figure supplement 1-labeled/SB9200 Figure 1-figure supplement 1C-labeled/A549 SB9200.tif]

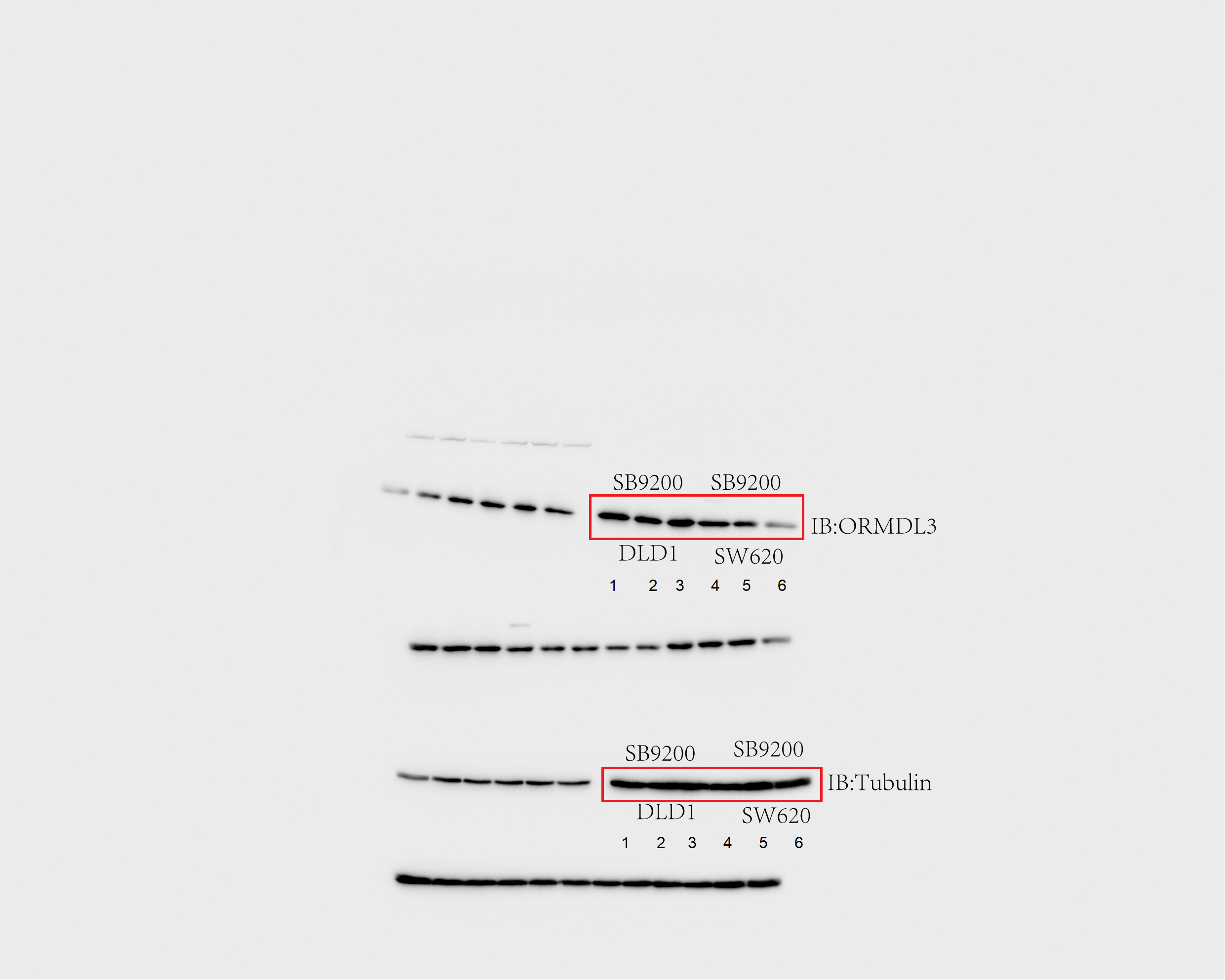

Supplement: Figure 1—figure supplement 1—source data 1. [file elife-101973-fig1-figsupp1-data1.zip › Figure 1-figure supplement 1-source data 1/Figure 1-figure supplement 1-labeled/SB9200 Figure 1-figure supplement 1C-labeled/DLD1 SW620 SB9200.tif]

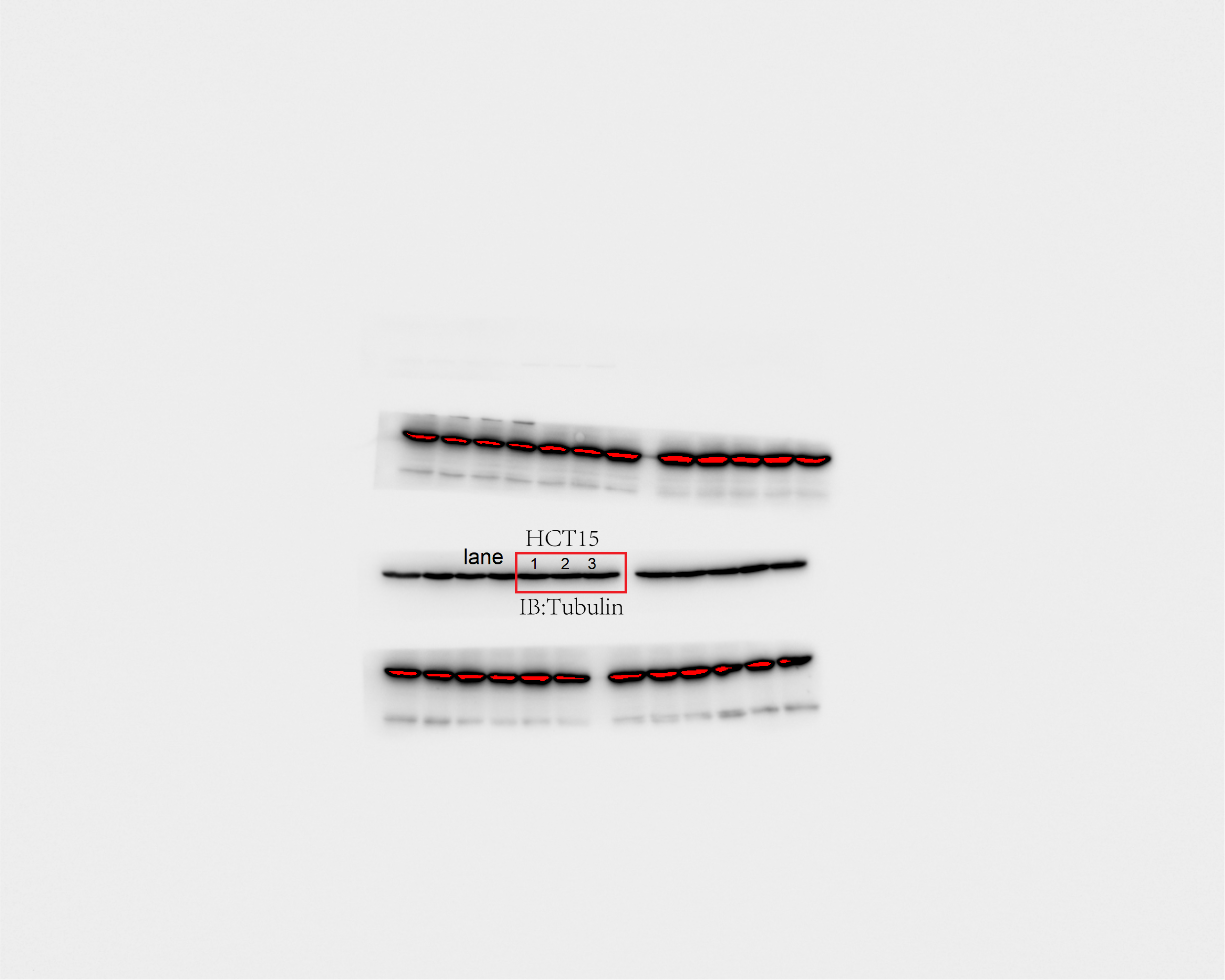

Supplement: Figure 1—figure supplement 1—source data 1. [file elife-101973-fig1-figsupp1-data1.zip › Figure 1-figure supplement 1-source data 1/Figure 1-figure supplement 1-labeled/SB9200 Figure 1-figure supplement 1C-labeled/HCT15 SB9200 Tubulin.tif]

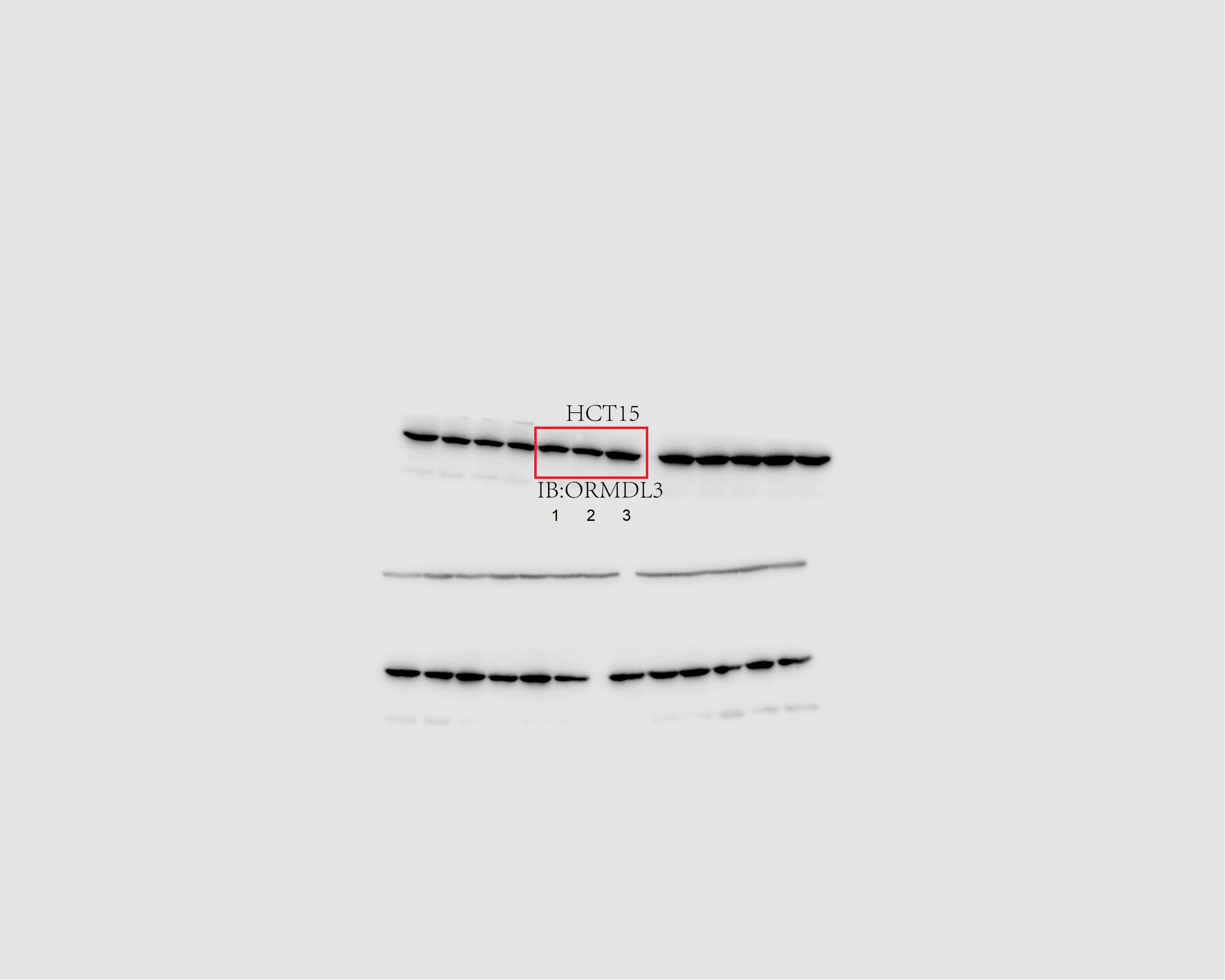

Supplement: Figure 1—figure supplement 1—source data 1. [file elife-101973-fig1-figsupp1-data1.zip › Figure 1-figure supplement 1-source data 1/Figure 1-figure supplement 1-labeled/SB9200 Figure 1-figure supplement 1C-labeled/HCT15 SB9200.tif]

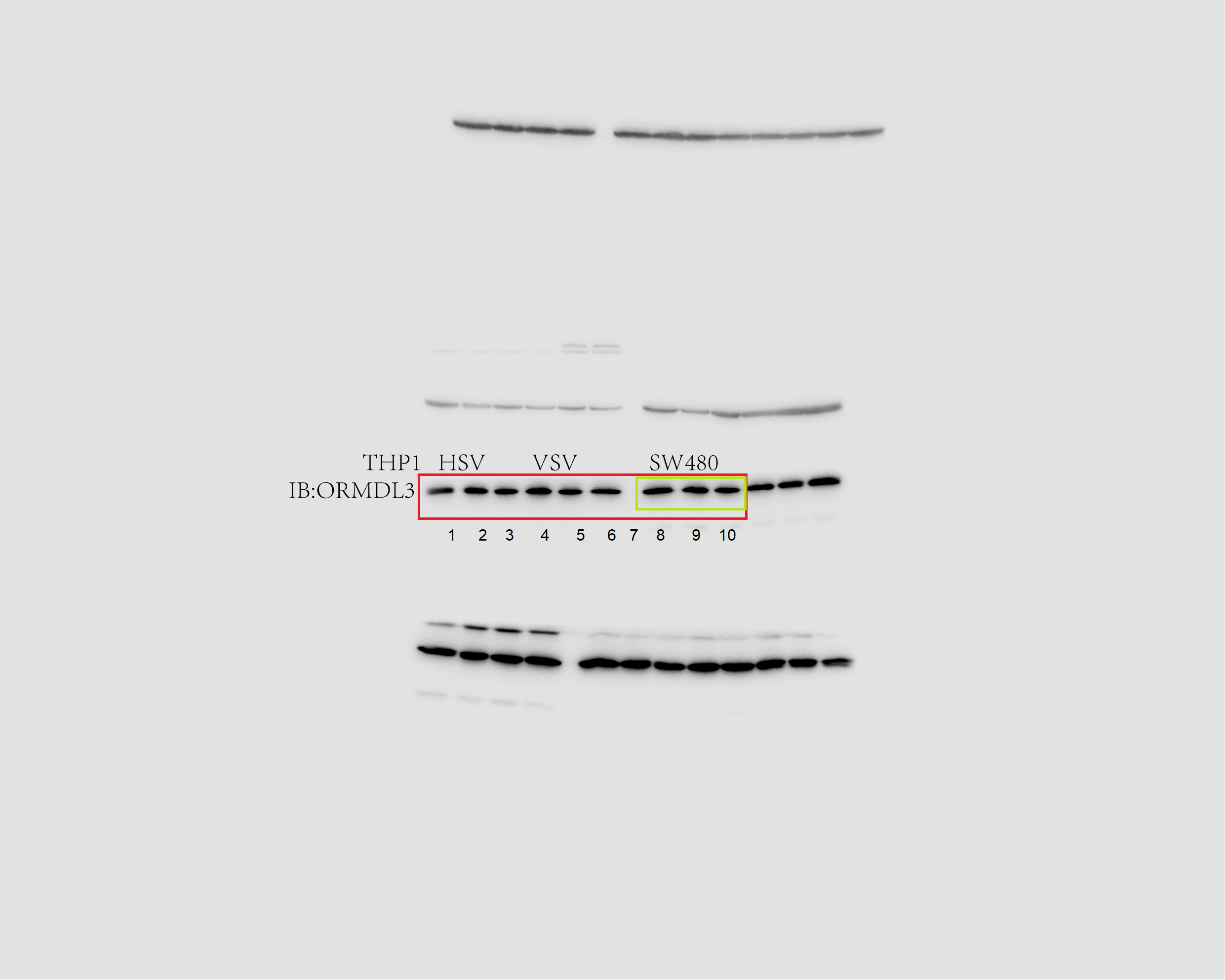

Supplement: Figure 1—figure supplement 1—source data 1. [file elife-101973-fig1-figsupp1-data1.zip › Figure 1-figure supplement 1-source data 1/Figure 1-figure supplement 1-labeled/SB9200 Figure 1-figure supplement 1C-labeled/SW480 SB9200 .tif]

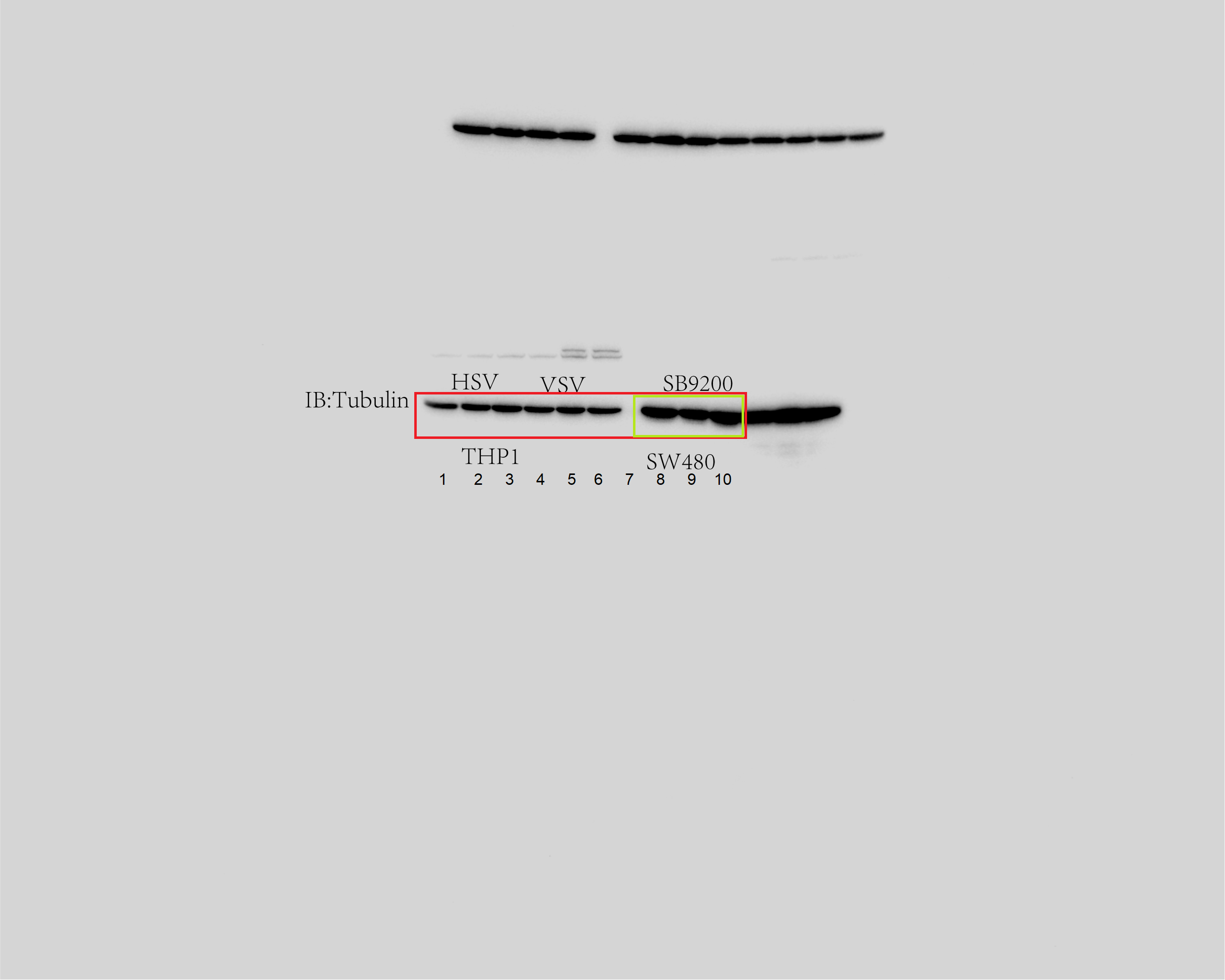

Supplement: Figure 1—figure supplement 1—source data 1. [file elife-101973-fig1-figsupp1-data1.zip › Figure 1-figure supplement 1-source data 1/Figure 1-figure supplement 1-labeled/SB9200 Figure 1-figure supplement 1C-labeled/SW480 SB9200 Tubulin.tif]

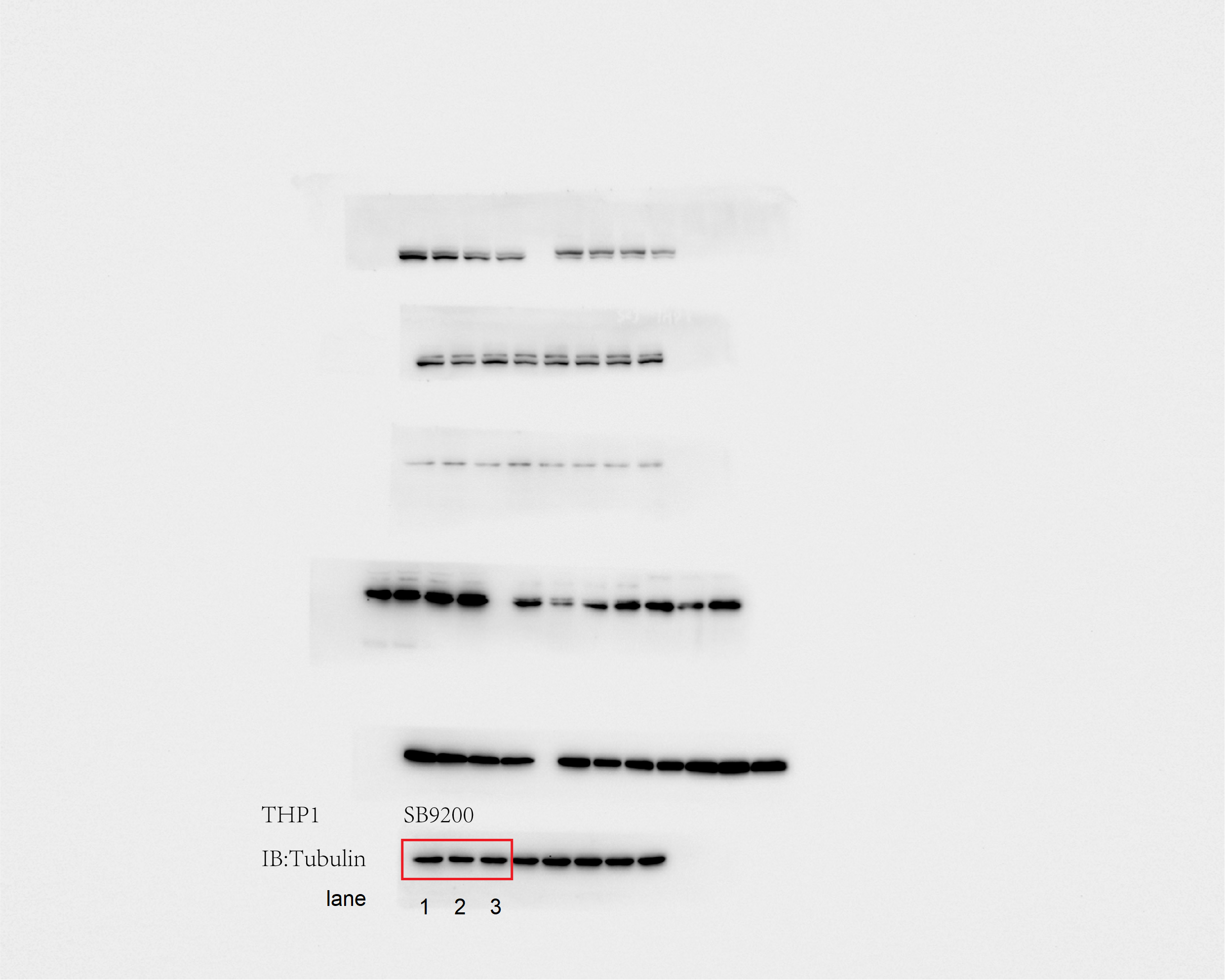

Supplement: Figure 1—figure supplement 1—source data 1. [file elife-101973-fig1-figsupp1-data1.zip › Figure 1-figure supplement 1-source data 1/Figure 1-figure supplement 1-labeled/SB9200 Figure 1-figure supplement 1C-labeled/THP1 SB9200 Tubulin.tif]

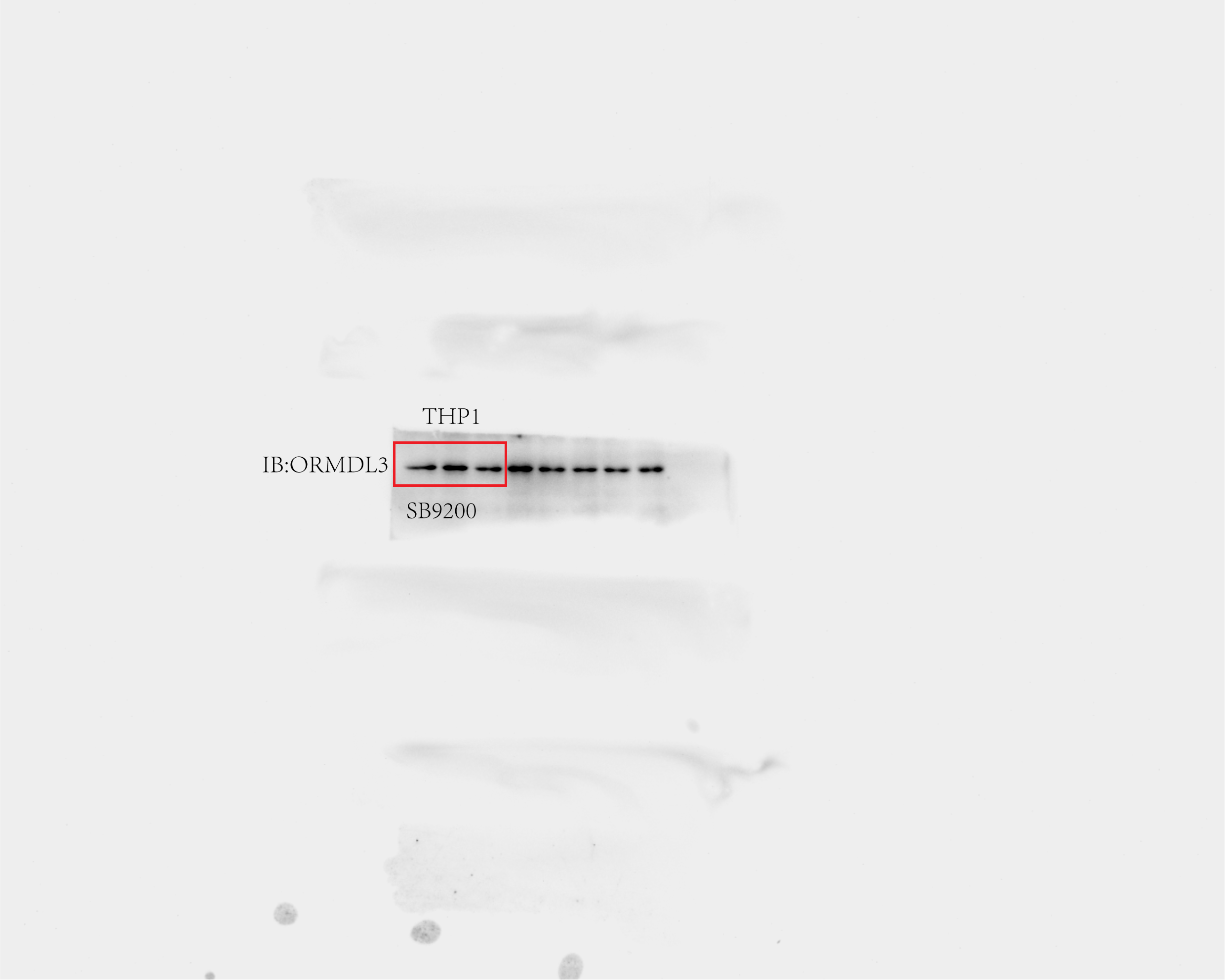

Supplement: Figure 1—figure supplement 1—source data 1. [file elife-101973-fig1-figsupp1-data1.zip › Figure 1-figure supplement 1-source data 1/Figure 1-figure supplement 1-labeled/SB9200 Figure 1-figure supplement 1C-labeled/THP1 SB9200.tif]

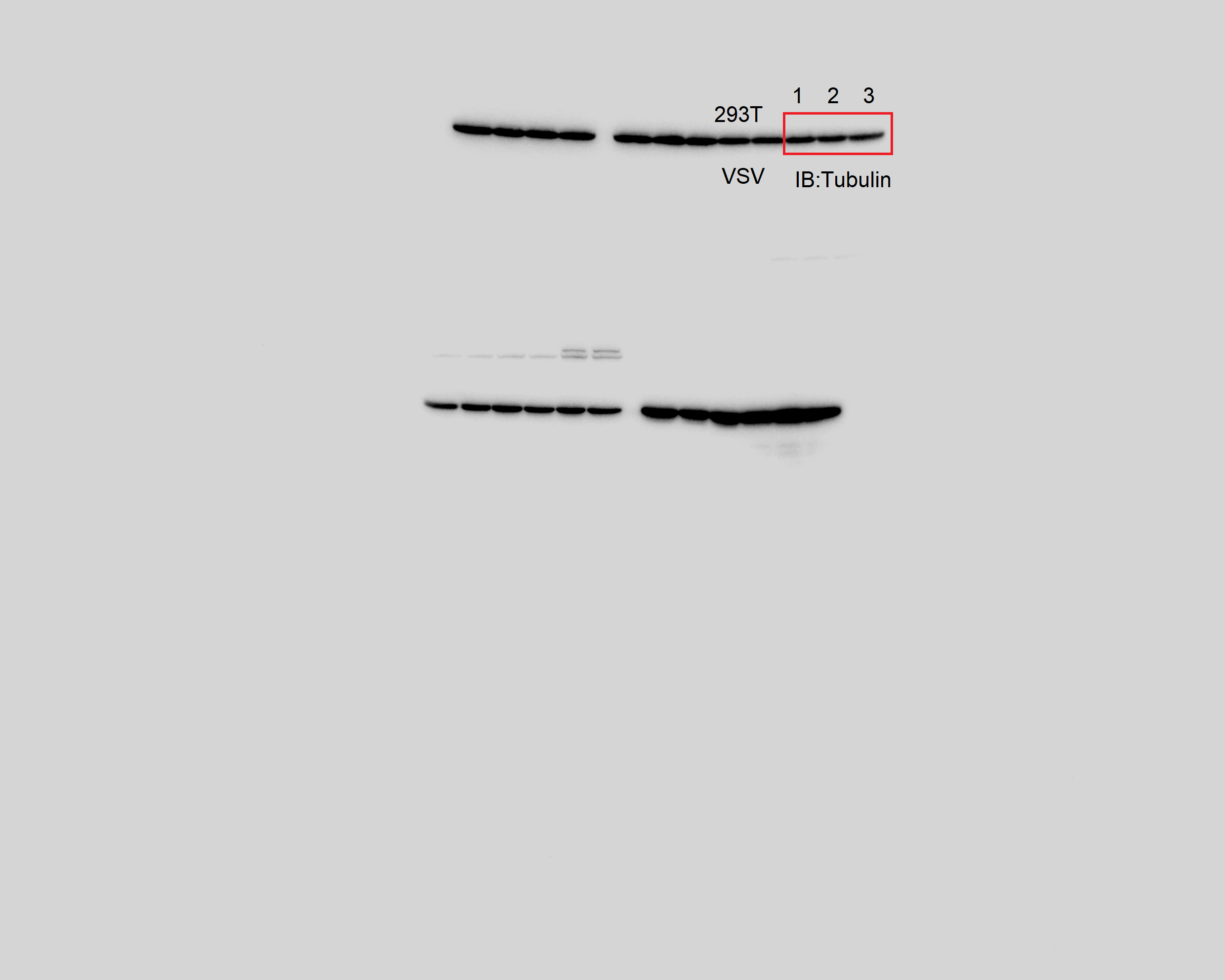

Supplement: Figure 1—figure supplement 1—source data 1. [file elife-101973-fig1-figsupp1-data1.zip › Figure 1-figure supplement 1-source data 1/Figure 1-figure supplement 1-labeled/VSV Figure 1-figure supplement 1B-labeled/293T VSV Tubulin long exposure.tif]

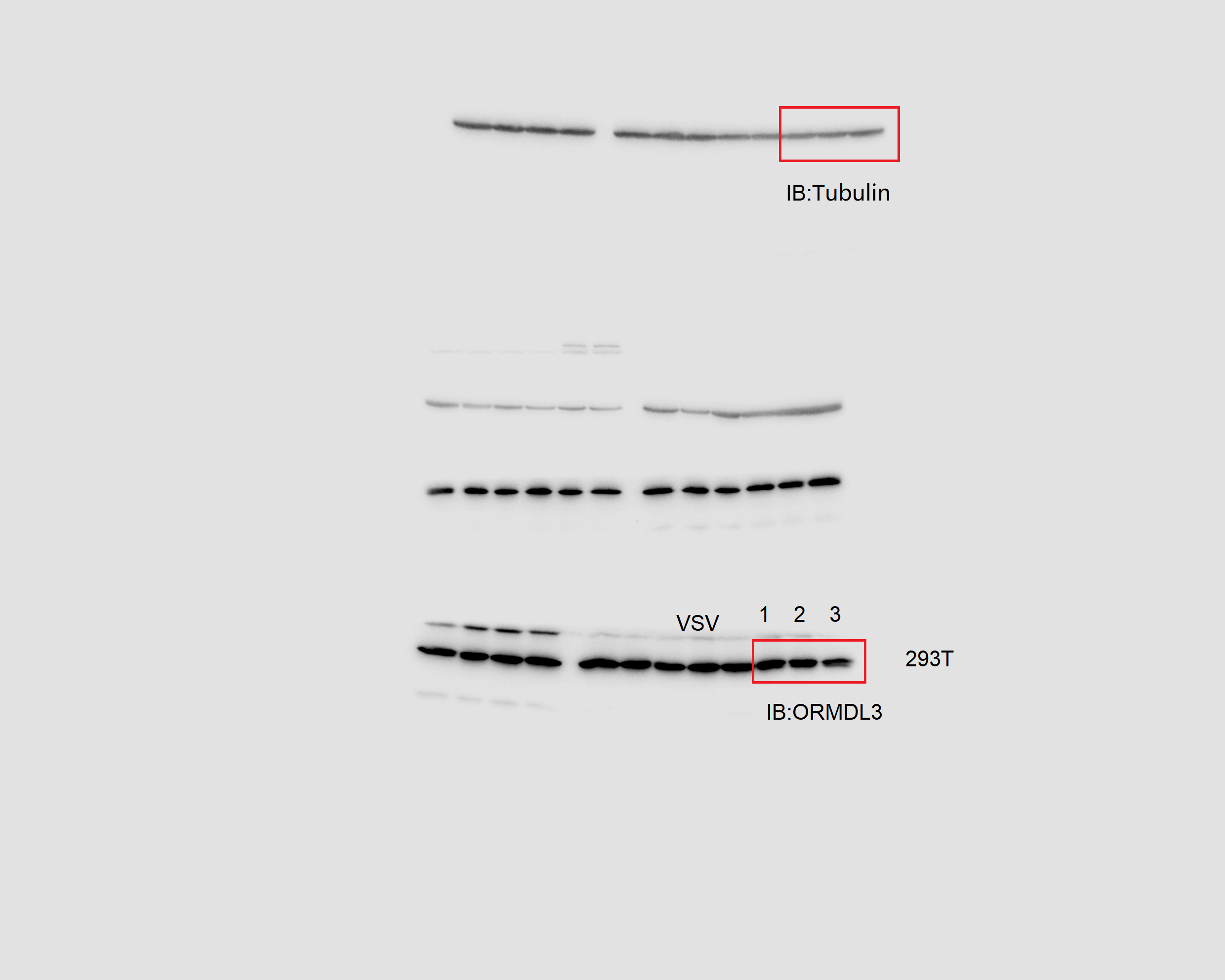

Supplement: Figure 1—figure supplement 1—source data 1. [file elife-101973-fig1-figsupp1-data1.zip › Figure 1-figure supplement 1-source data 1/Figure 1-figure supplement 1-labeled/VSV Figure 1-figure supplement 1B-labeled/293T VSV.tif]

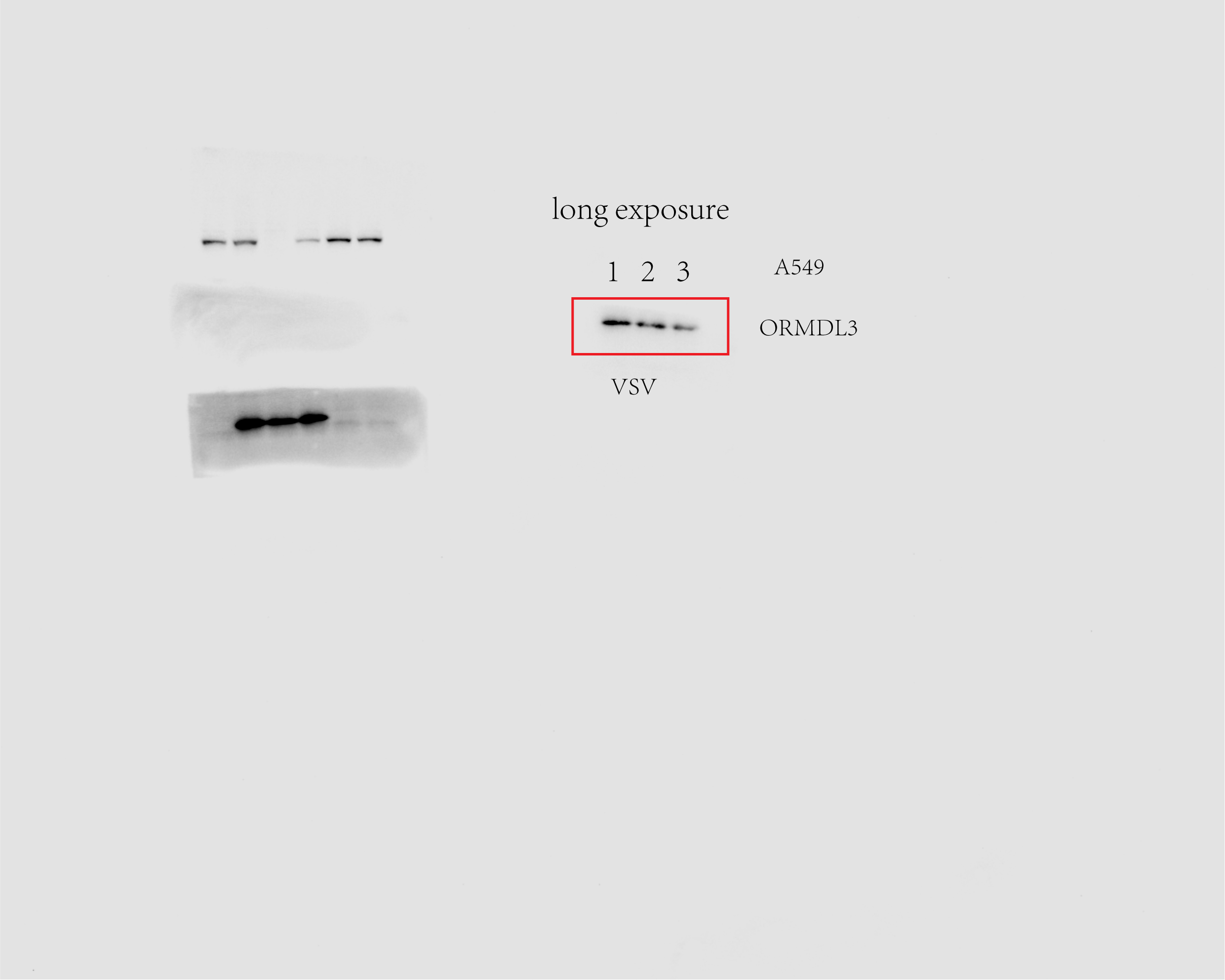

Supplement: Figure 1—figure supplement 1—source data 1. [file elife-101973-fig1-figsupp1-data1.zip › Figure 1-figure supplement 1-source data 1/Figure 1-figure supplement 1-labeled/VSV Figure 1-figure supplement 1B-labeled/A549 VSV long exposure.tif]

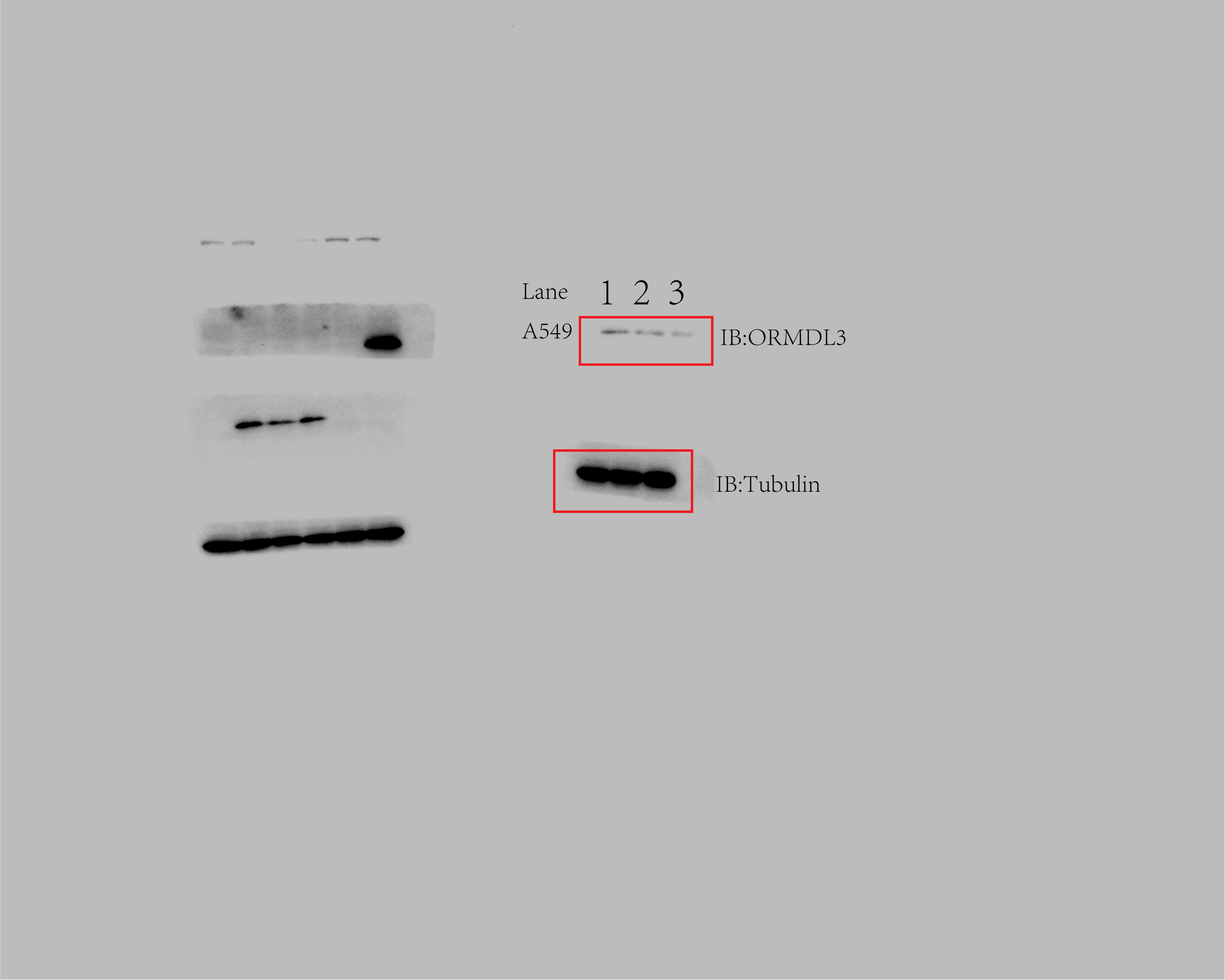

Supplement: Figure 1—figure supplement 1—source data 1. [file elife-101973-fig1-figsupp1-data1.zip › Figure 1-figure supplement 1-source data 1/Figure 1-figure supplement 1-labeled/VSV Figure 1-figure supplement 1B-labeled/A549 VSV.tif]

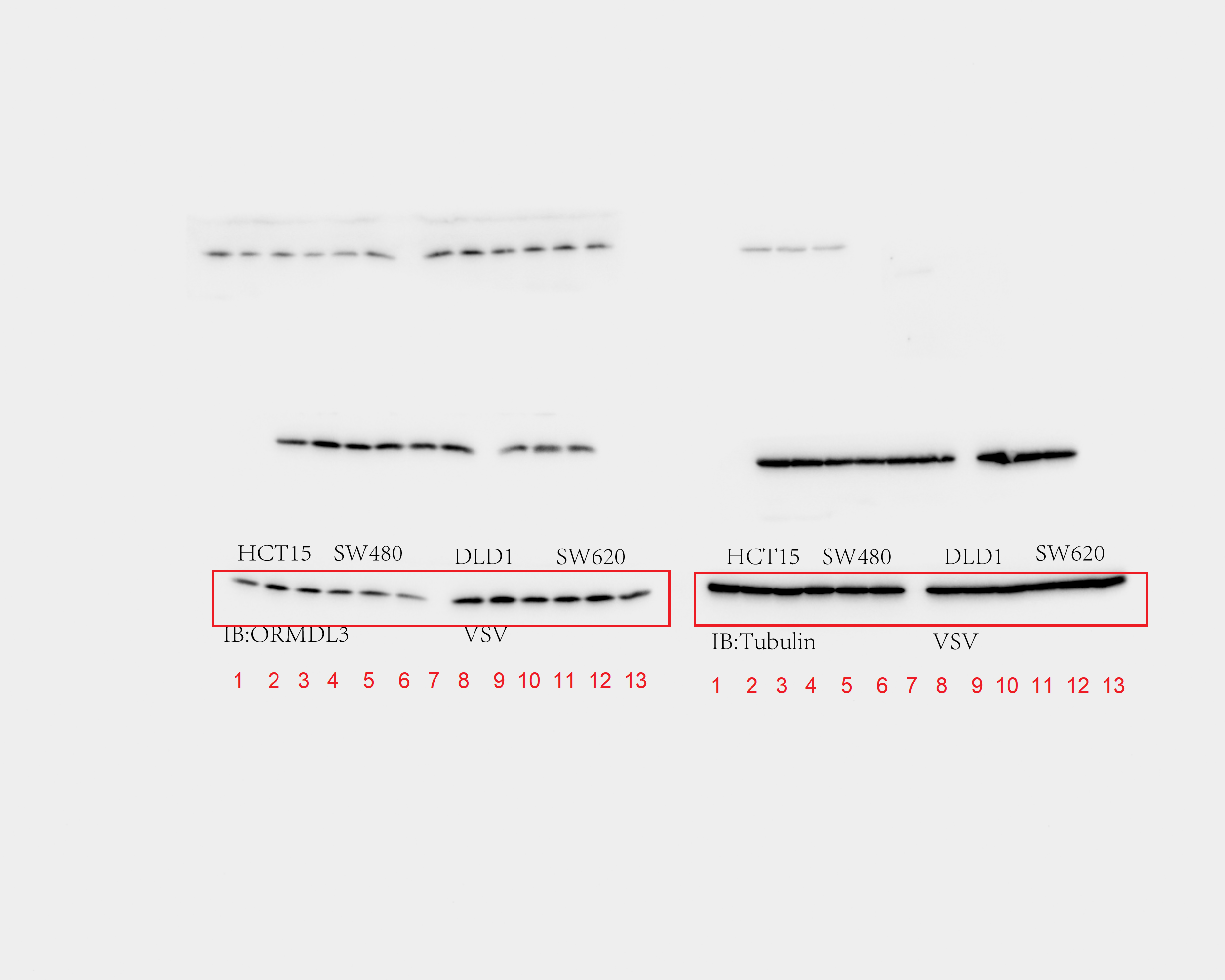

Supplement: Figure 1—figure supplement 1—source data 1. [file elife-101973-fig1-figsupp1-data1.zip › Figure 1-figure supplement 1-source data 1/Figure 1-figure supplement 1-labeled/VSV Figure 1-figure supplement 1B-labeled/HCT15 SW480 DLD1 SW620 VSV.tif]

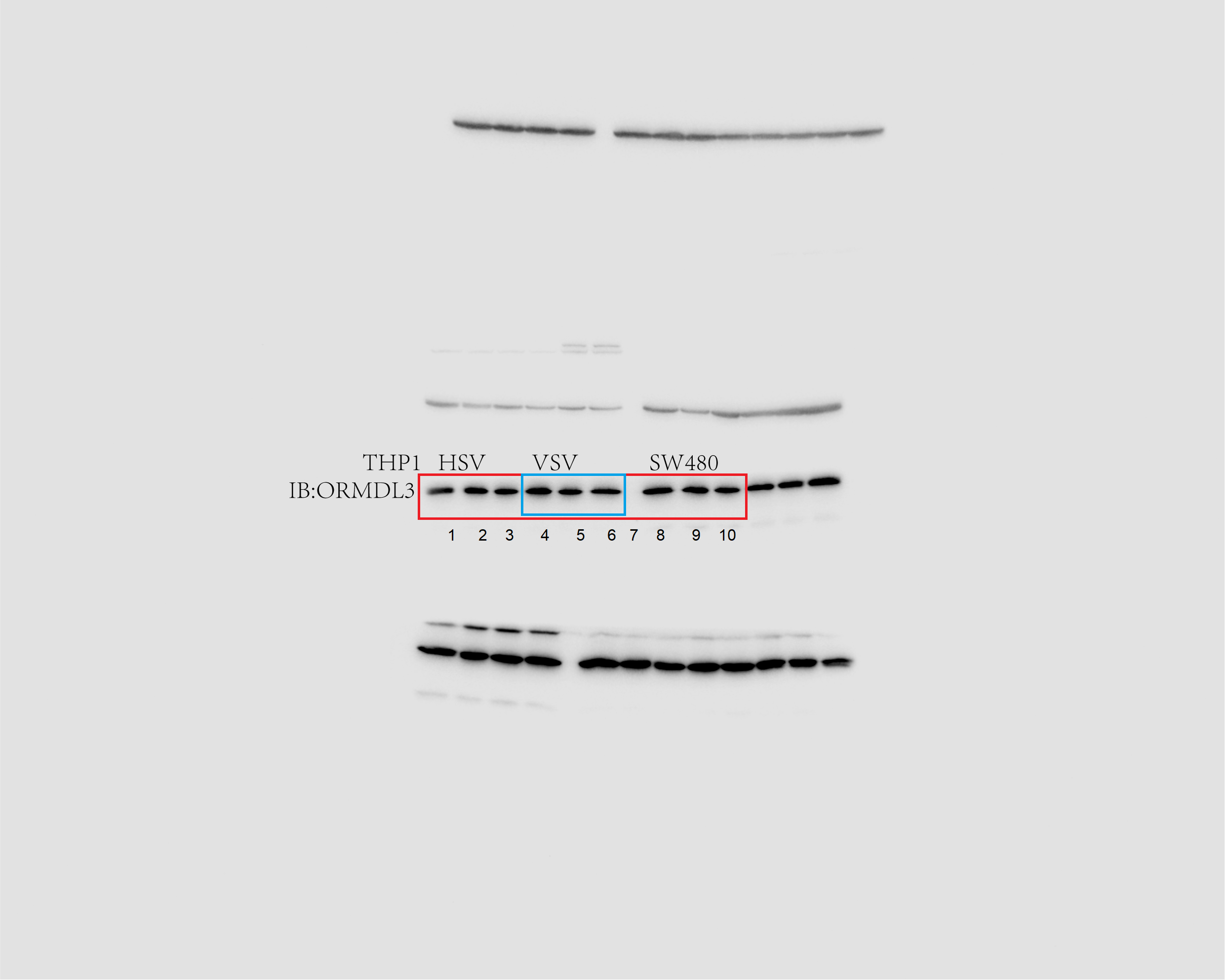

Supplement: Figure 1—figure supplement 1—source data 1. [file elife-101973-fig1-figsupp1-data1.zip › Figure 1-figure supplement 1-source data 1/Figure 1-figure supplement 1-labeled/VSV Figure 1-figure supplement 1B-labeled/THP1 VSV .tif]

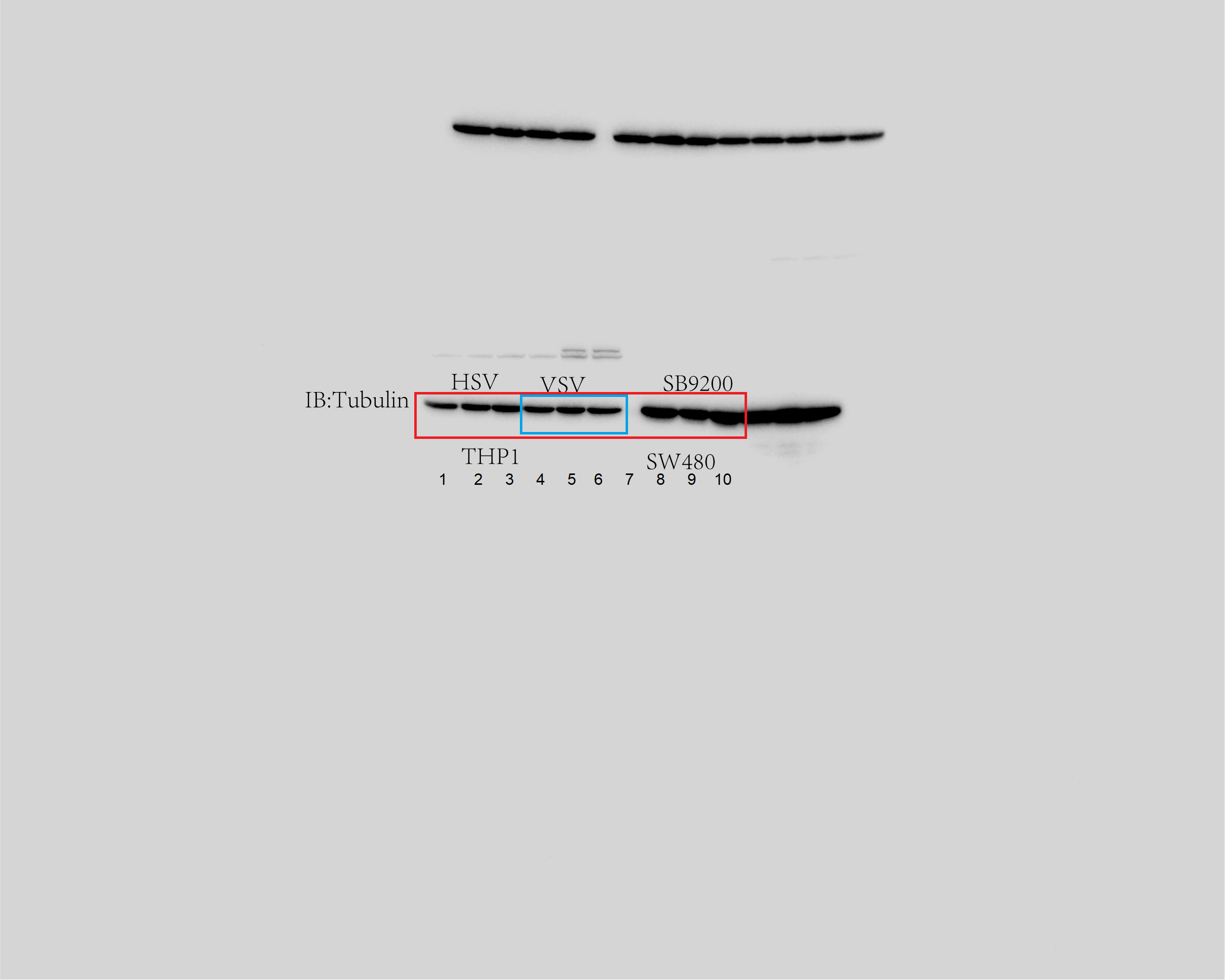

Supplement: Figure 1—figure supplement 1—source data 1. [file elife-101973-fig1-figsupp1-data1.zip › Figure 1-figure supplement 1-source data 1/Figure 1-figure supplement 1-labeled/VSV Figure 1-figure supplement 1B-labeled/THP1 VSV Tubulin .tif]

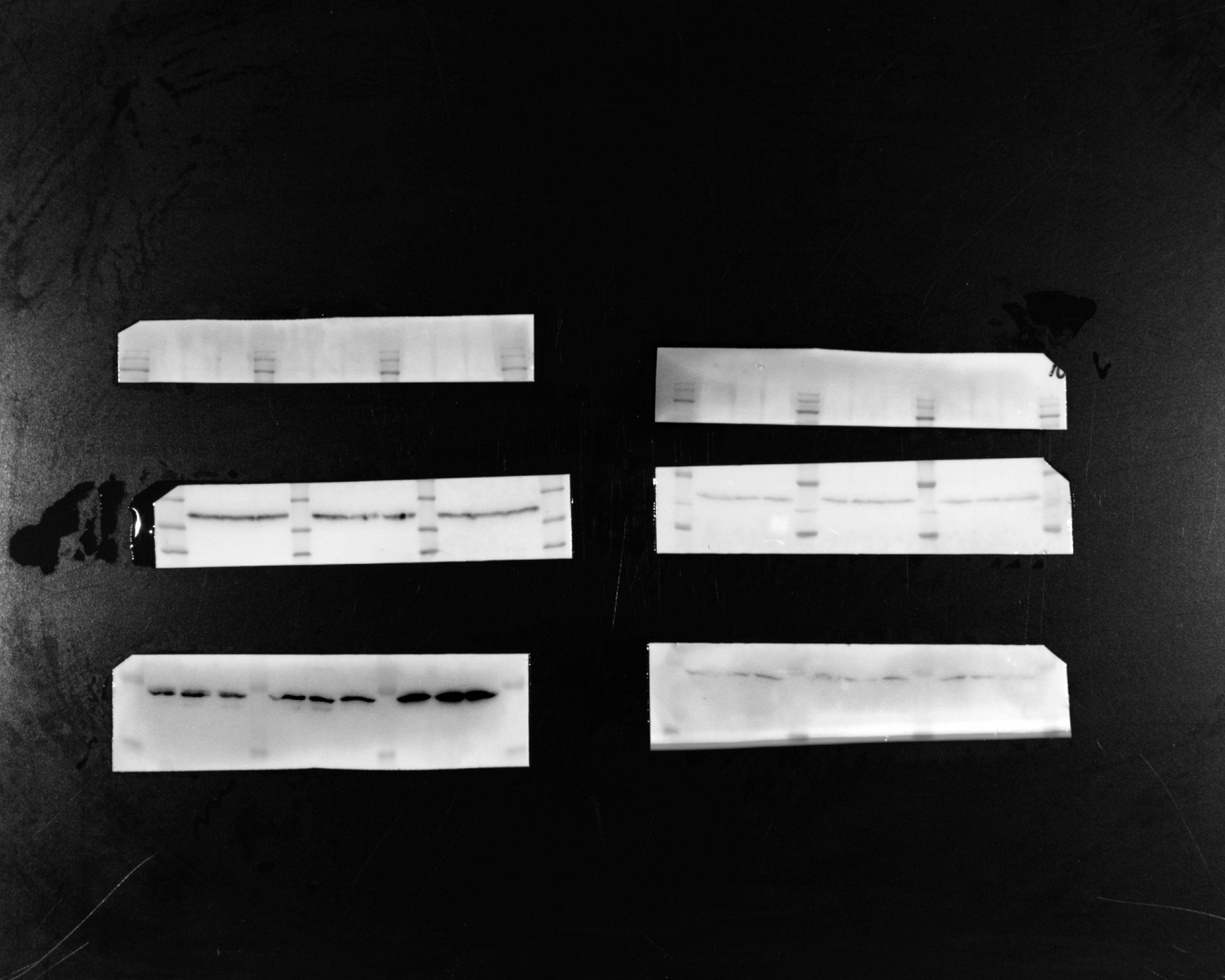

Supplement: Figure 1—figure supplement 1—source data 2. [file elife-101973-fig1-figsupp1-data2.zip › Figure 1-figure supplement 1-source data 2/Figure 1-figure supplement 1/HSV Figure 1–figure supplement 1A/293T HCT15 ORMDL3 and tubulin.jpg]

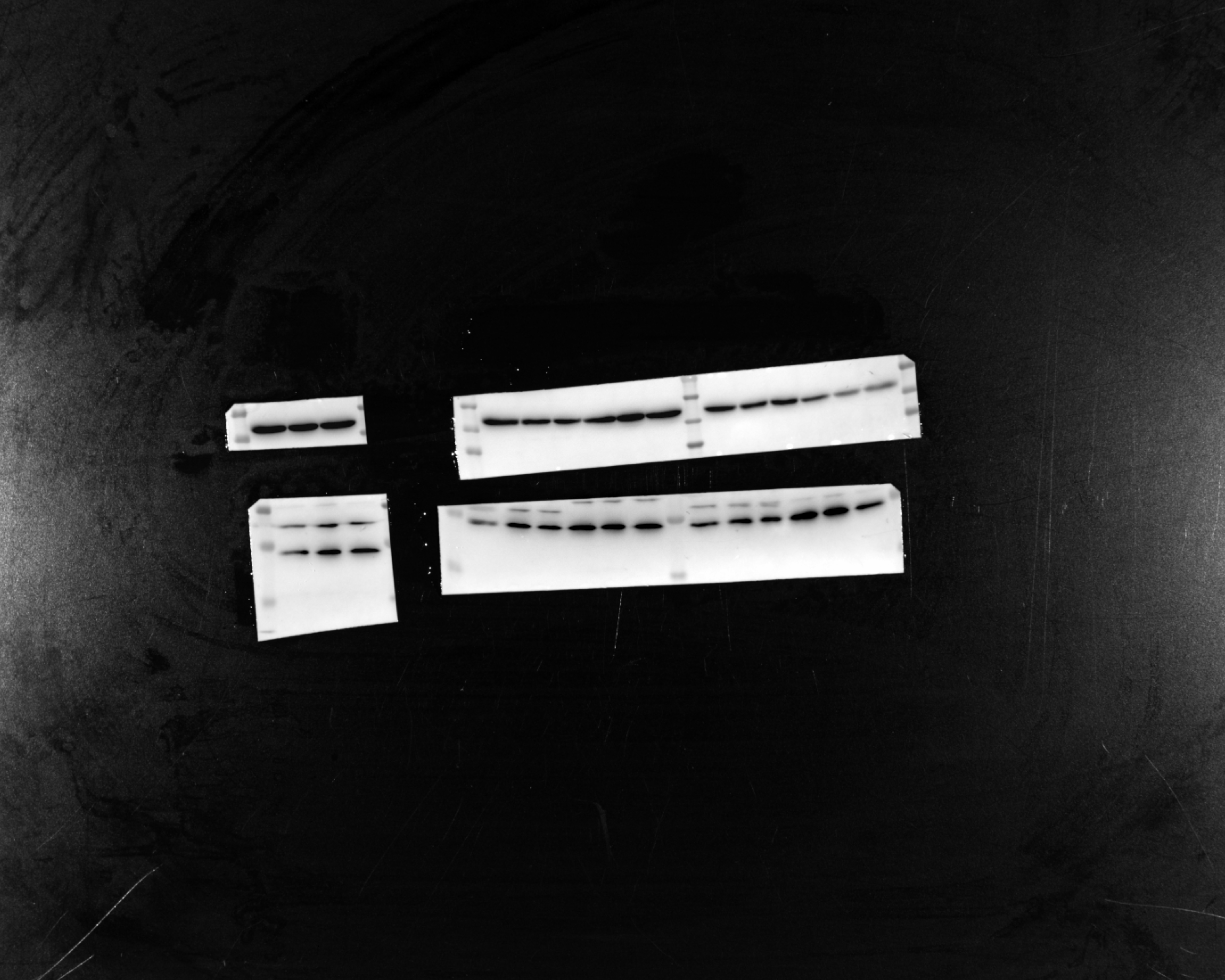

Supplement: Figure 1—figure supplement 1—source data 2. [file elife-101973-fig1-figsupp1-data2.zip › Figure 1-figure supplement 1-source data 2/Figure 1-figure supplement 1/HSV Figure 1–figure supplement 1A/A549 ORMDL3 Tubulin.jpg]

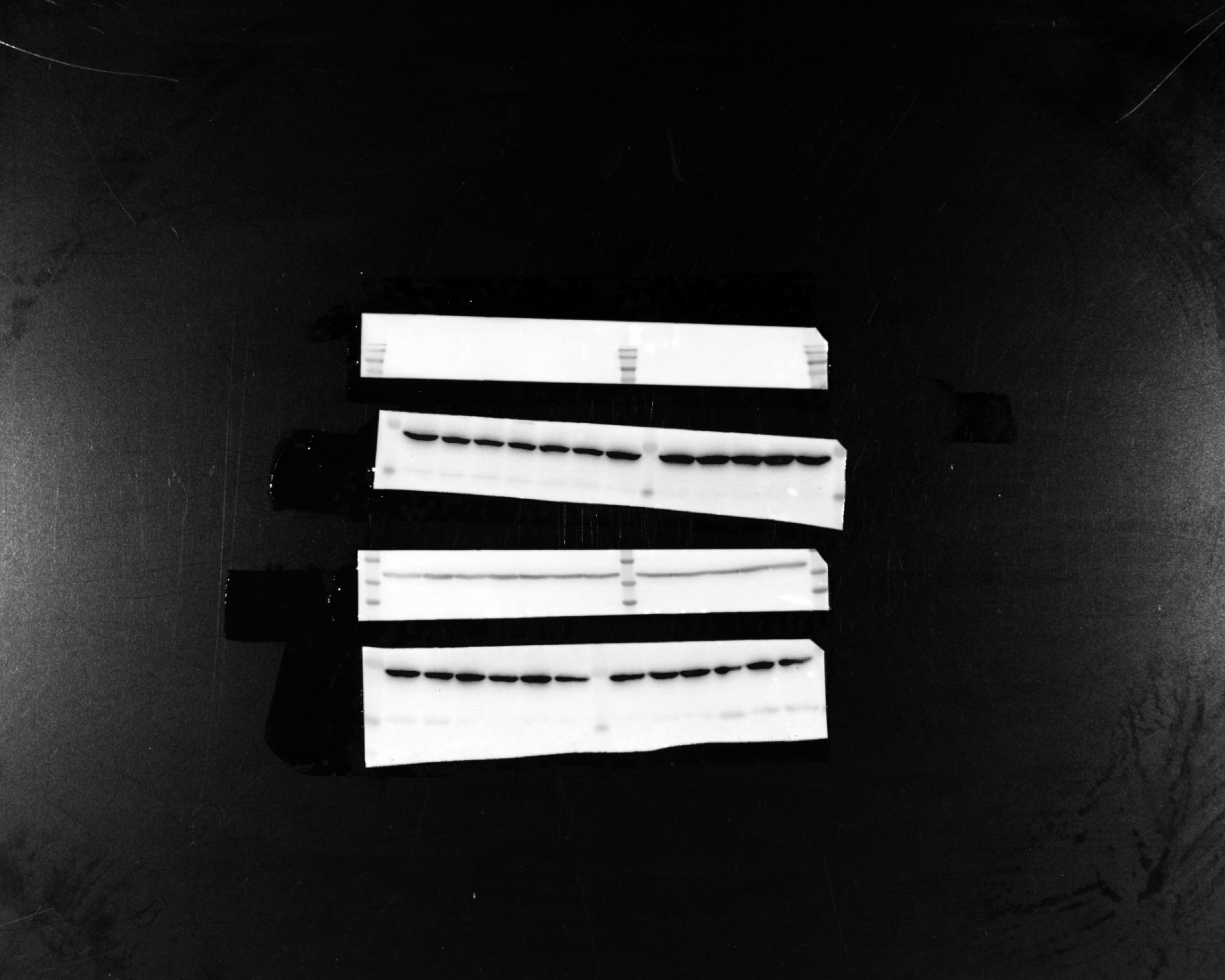

Supplement: Figure 1—figure supplement 1—source data 2. [file elife-101973-fig1-figsupp1-data2.zip › Figure 1-figure supplement 1-source data 2/Figure 1-figure supplement 1/HSV Figure 1–figure supplement 1A/DLD1 HSV ORMDL3.jpg]

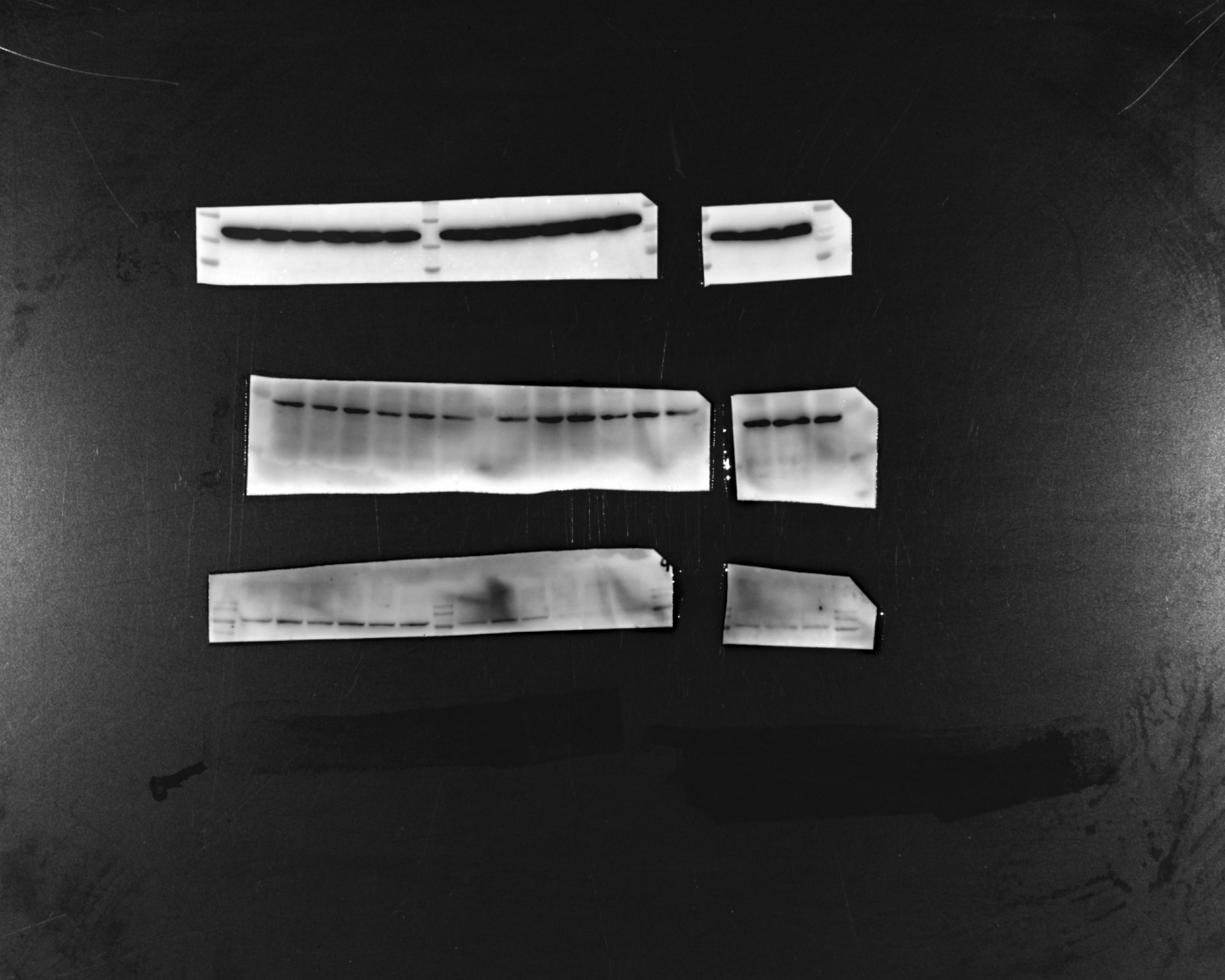

Supplement: Figure 1—figure supplement 1—source data 2. [file elife-101973-fig1-figsupp1-data2.zip › Figure 1-figure supplement 1-source data 2/Figure 1-figure supplement 1/HSV Figure 1–figure supplement 1A/DLD1 HSV Tubulin.jpg]

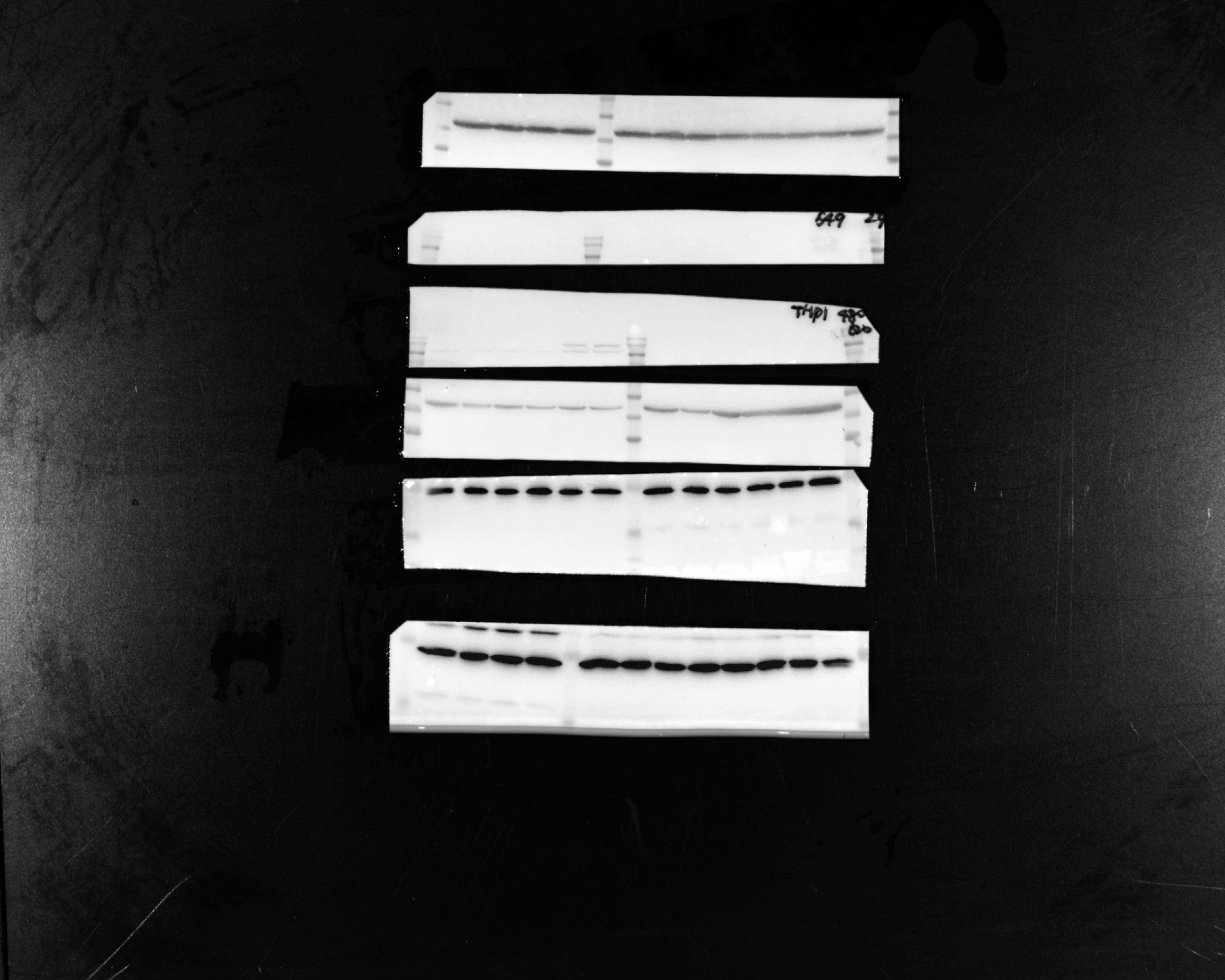

Supplement: Figure 1—figure supplement 1—source data 2. [file elife-101973-fig1-figsupp1-data2.zip › Figure 1-figure supplement 1-source data 2/Figure 1-figure supplement 1/HSV Figure 1–figure supplement 1A/THP1 HSV ORMDL3.jpg]

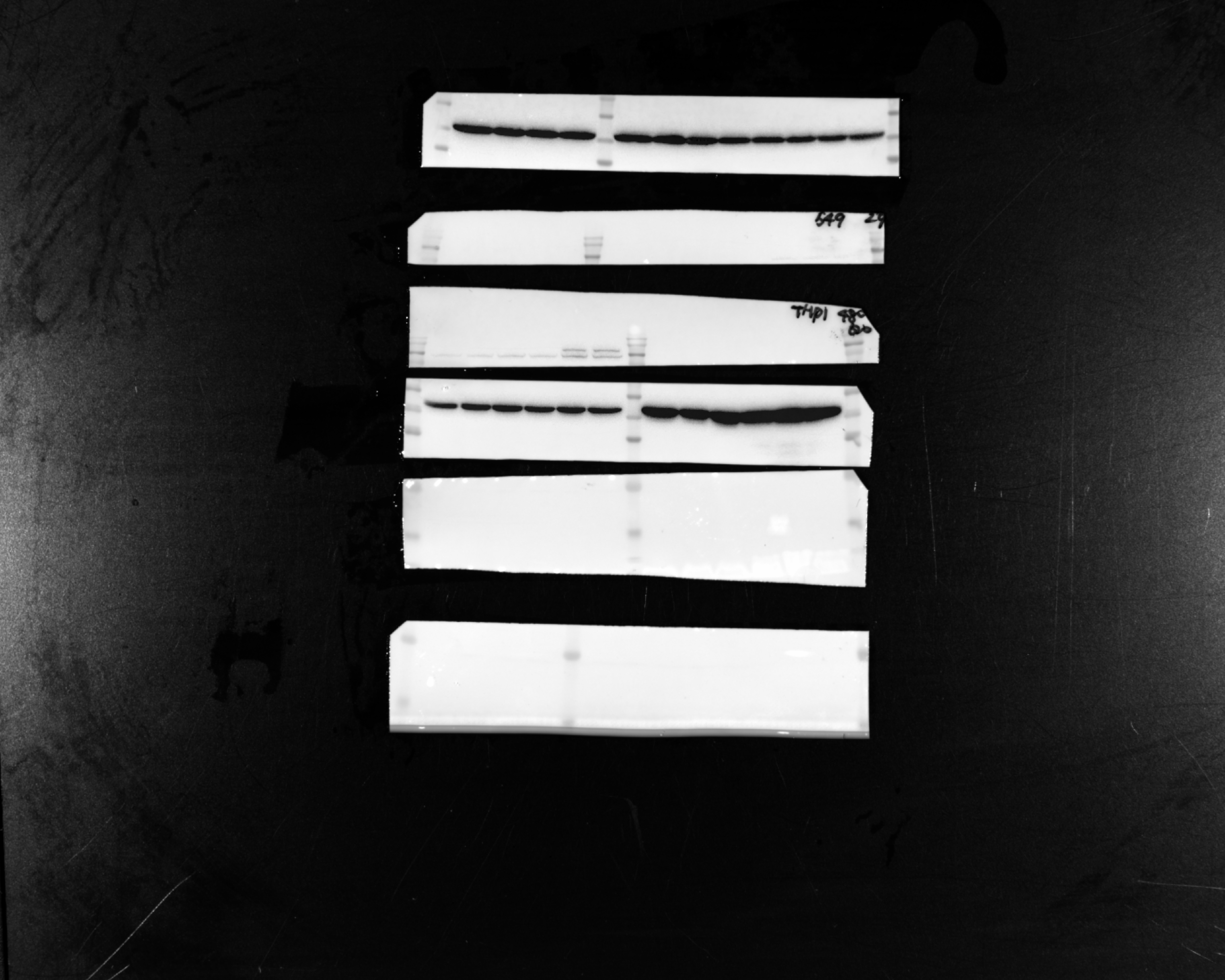

Supplement: Figure 1—figure supplement 1—source data 2. [file elife-101973-fig1-figsupp1-data2.zip › Figure 1-figure supplement 1-source data 2/Figure 1-figure supplement 1/HSV Figure 1–figure supplement 1A/THP1 VSV Tubulin.jpg]

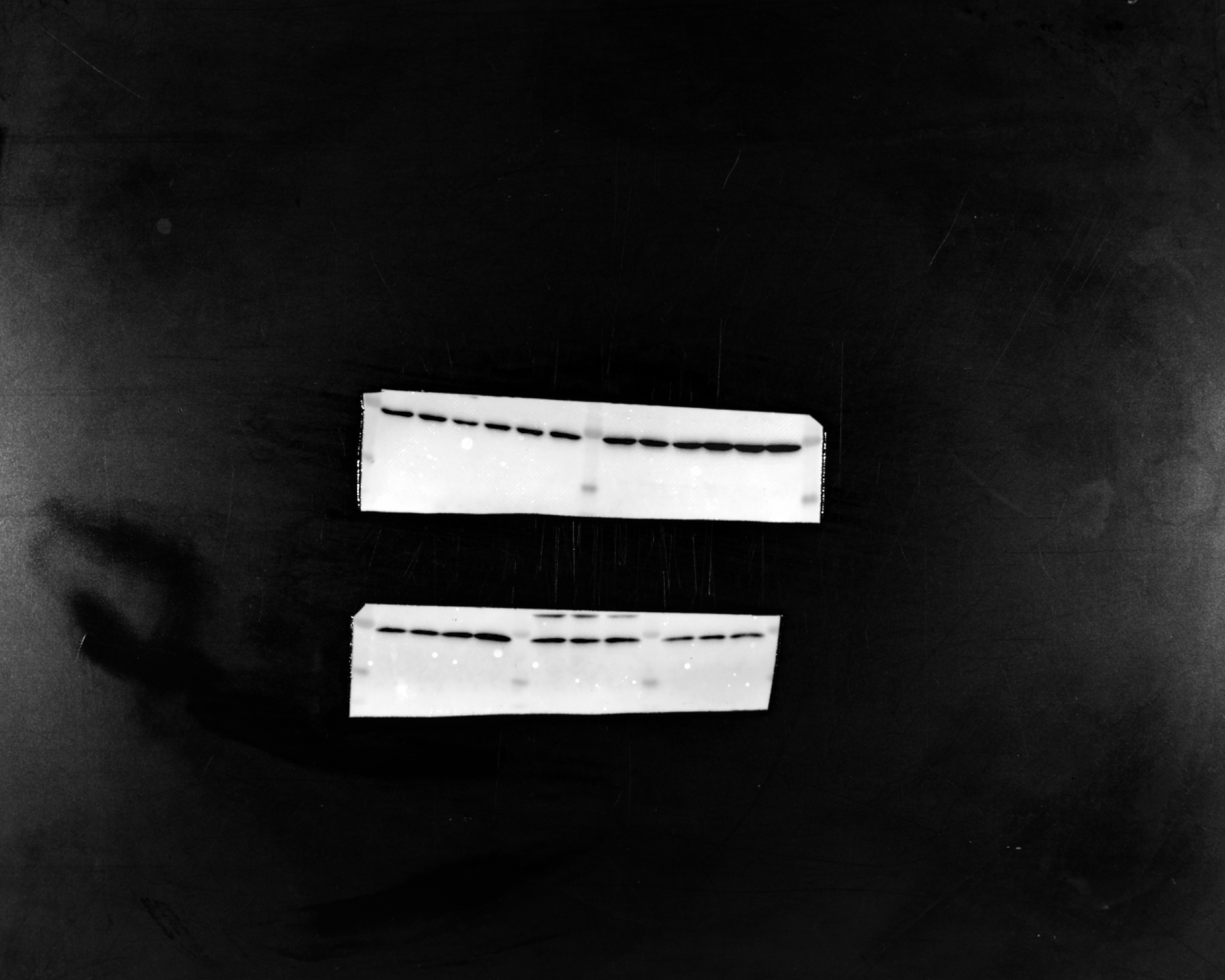

Supplement: Figure 1—figure supplement 1—source data 2. [file elife-101973-fig1-figsupp1-data2.zip › Figure 1-figure supplement 1-source data 2/Figure 1-figure supplement 1/SB9200 Figure 1–figure supplement 1C/A549 SB9200 ORMDL3.jpg]

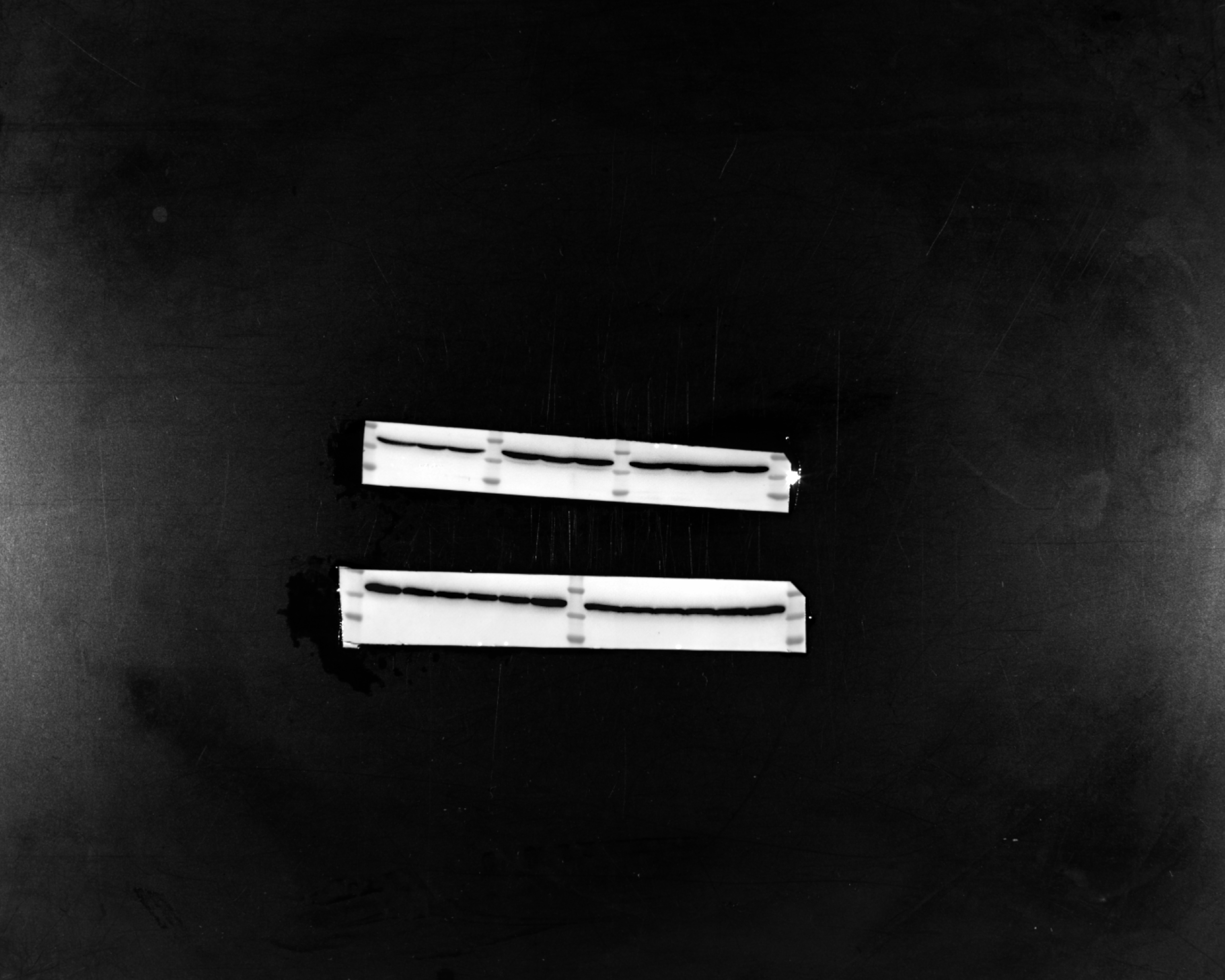

Supplement: Figure 1—figure supplement 1—source data 2. [file elife-101973-fig1-figsupp1-data2.zip › Figure 1-figure supplement 1-source data 2/Figure 1-figure supplement 1/SB9200 Figure 1–figure supplement 1C/A549 SB9200 tubulin.jpg]

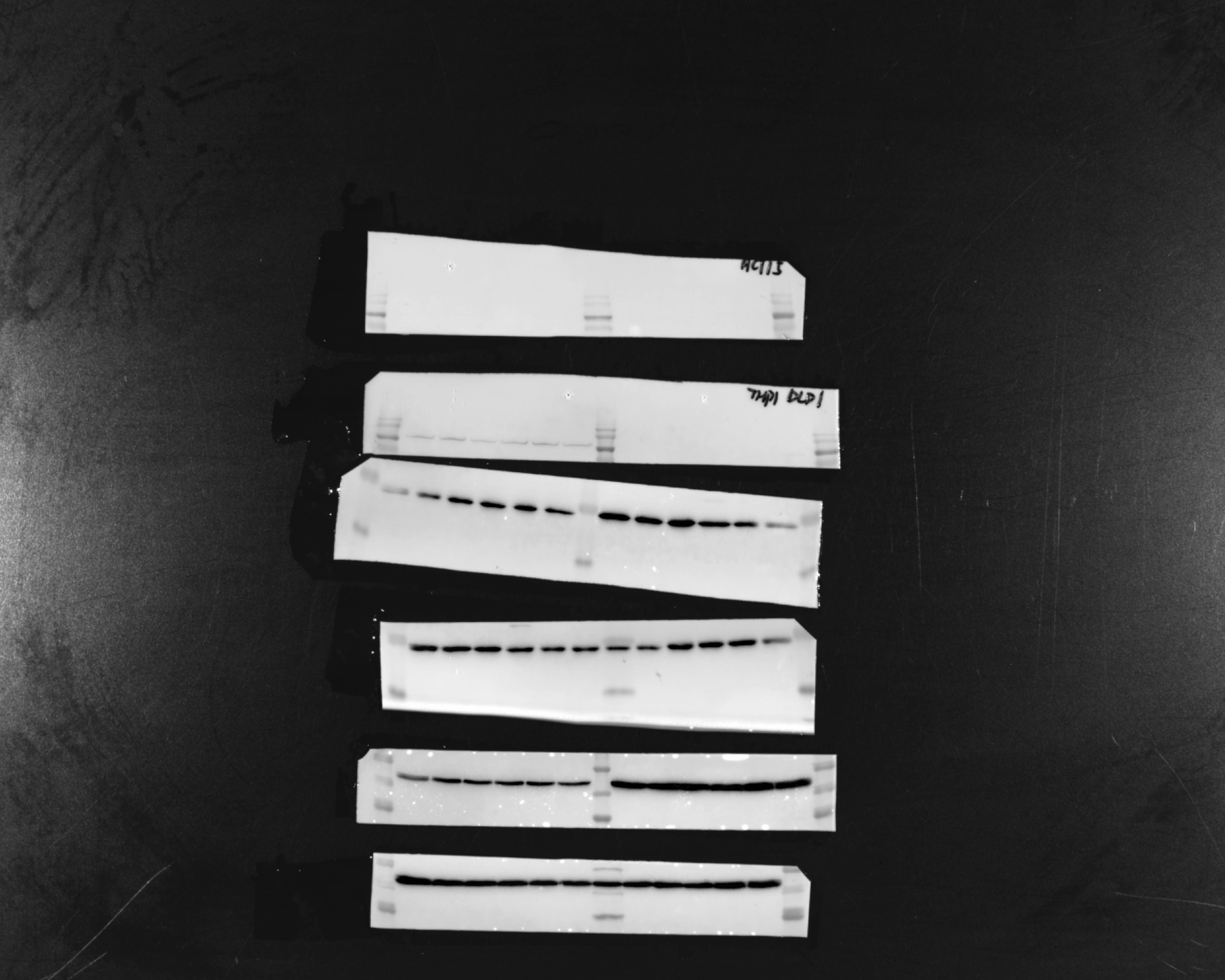

Supplement: Figure 1—figure supplement 1—source data 2. [file elife-101973-fig1-figsupp1-data2.zip › Figure 1-figure supplement 1-source data 2/Figure 1-figure supplement 1/SB9200 Figure 1–figure supplement 1C/DLD1 SW620 ORMDL3 and tubulin.jpg]

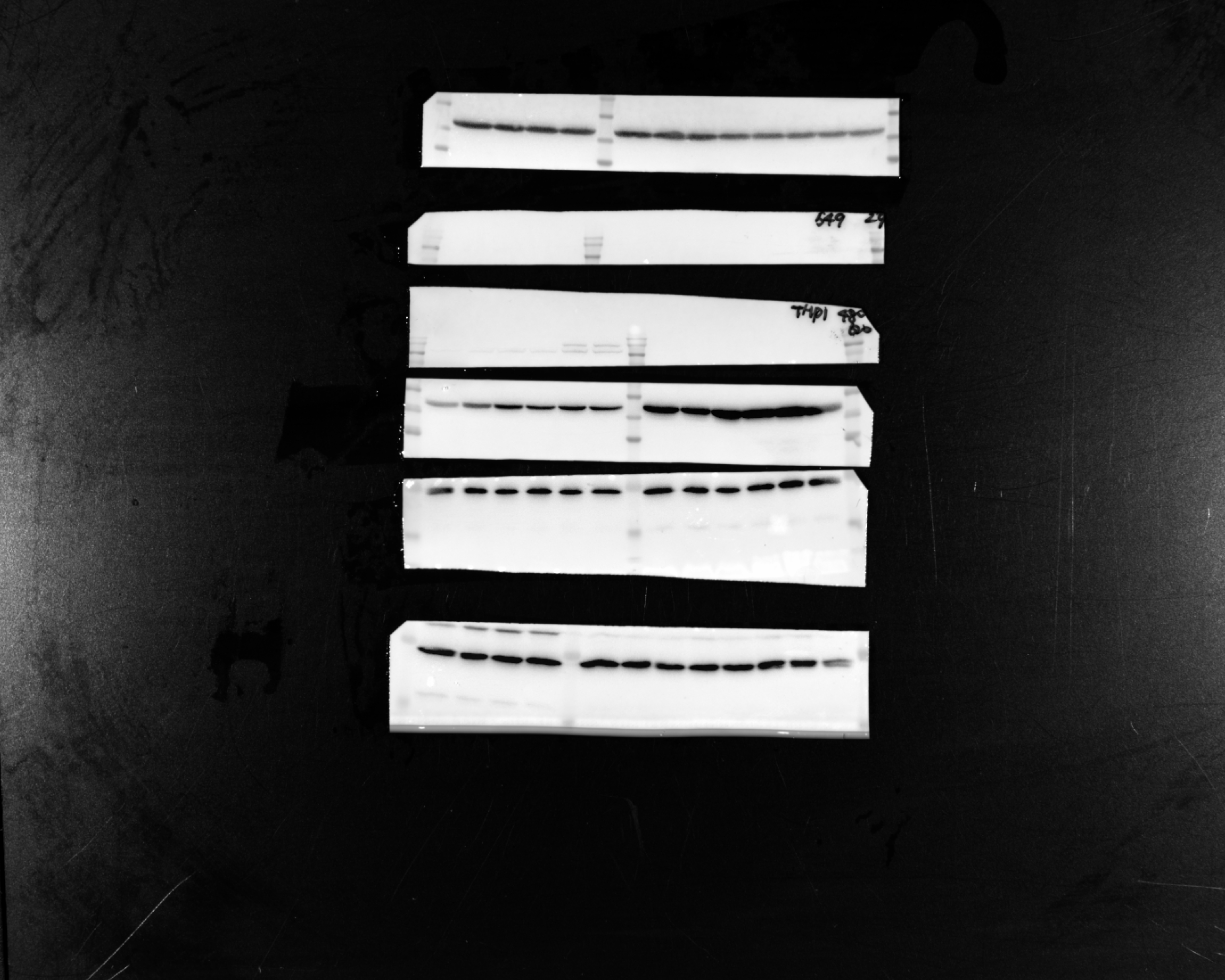

Supplement: Figure 1—figure supplement 1—source data 2. [file elife-101973-fig1-figsupp1-data2.zip › Figure 1-figure supplement 1-source data 2/Figure 1-figure supplement 1/SB9200 Figure 1–figure supplement 1C/SW480 SB9200 ORMDL3.jpg]

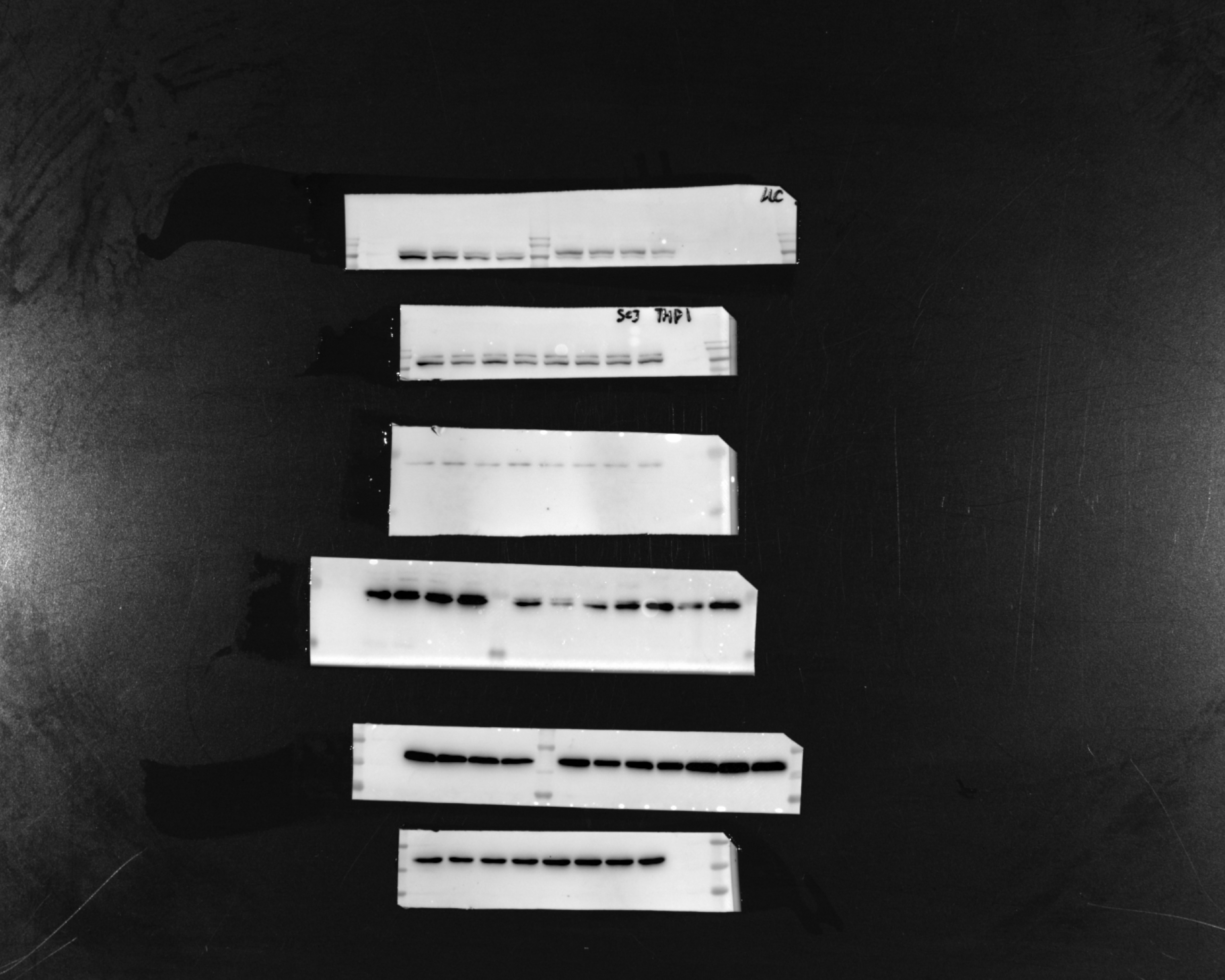

Supplement: Figure 1—figure supplement 1—source data 2. [file elife-101973-fig1-figsupp1-data2.zip › Figure 1-figure supplement 1-source data 2/Figure 1-figure supplement 1/SB9200 Figure 1–figure supplement 1C/THP1 SB9200 ORMDL3 and tubulin.jpg]

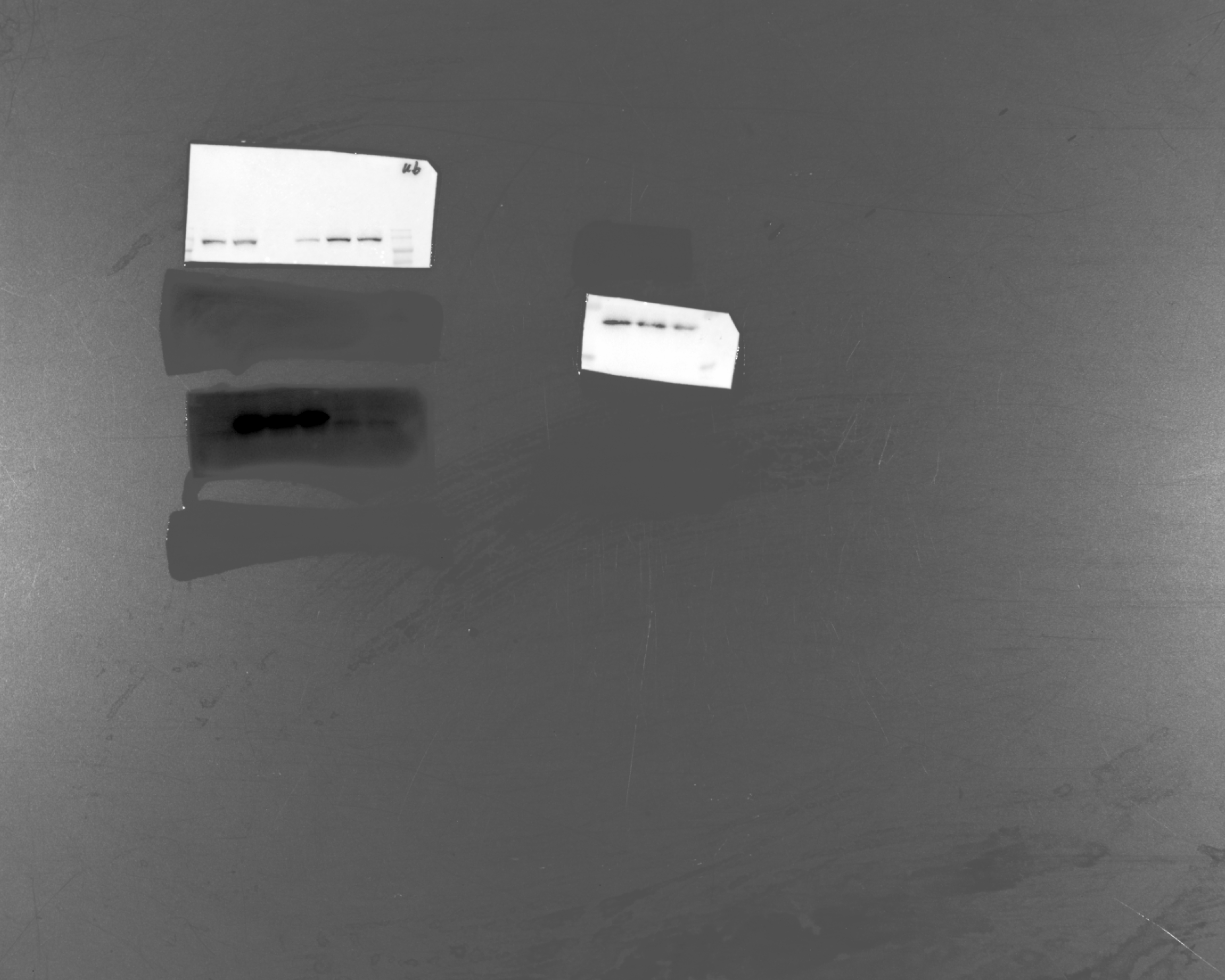

Supplement: Figure 1—figure supplement 1—source data 2. [file elife-101973-fig1-figsupp1-data2.zip › Figure 1-figure supplement 1-source data 2/Figure 1-figure supplement 1/VSV Figure 1–figure supplement 1B/A549 VSV ORMDL3.jpg]

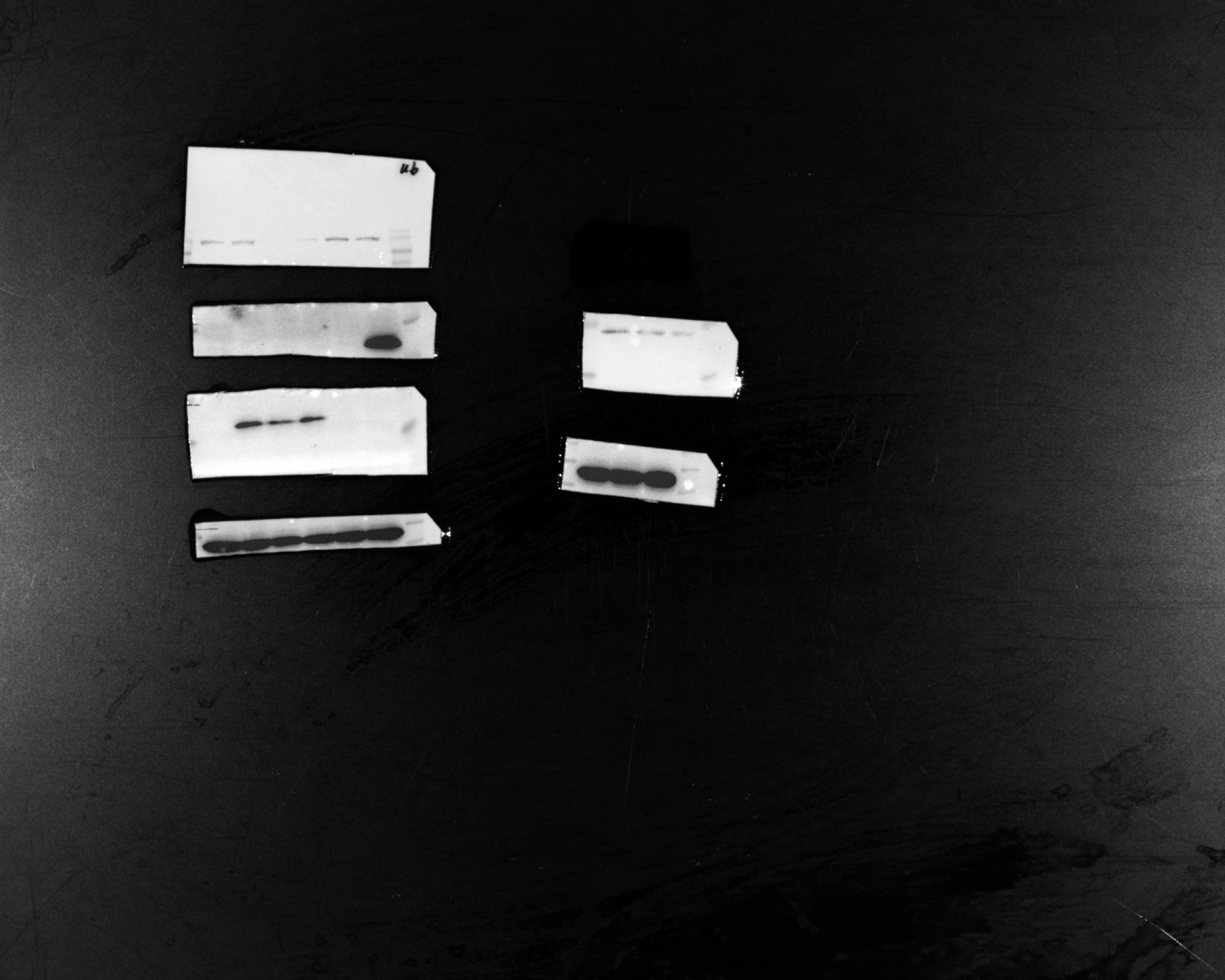

Supplement: Figure 1—figure supplement 1—source data 2. [file elife-101973-fig1-figsupp1-data2.zip › Figure 1-figure supplement 1-source data 2/Figure 1-figure supplement 1/VSV Figure 1–figure supplement 1B/A549 VSV tubulin.jpg]

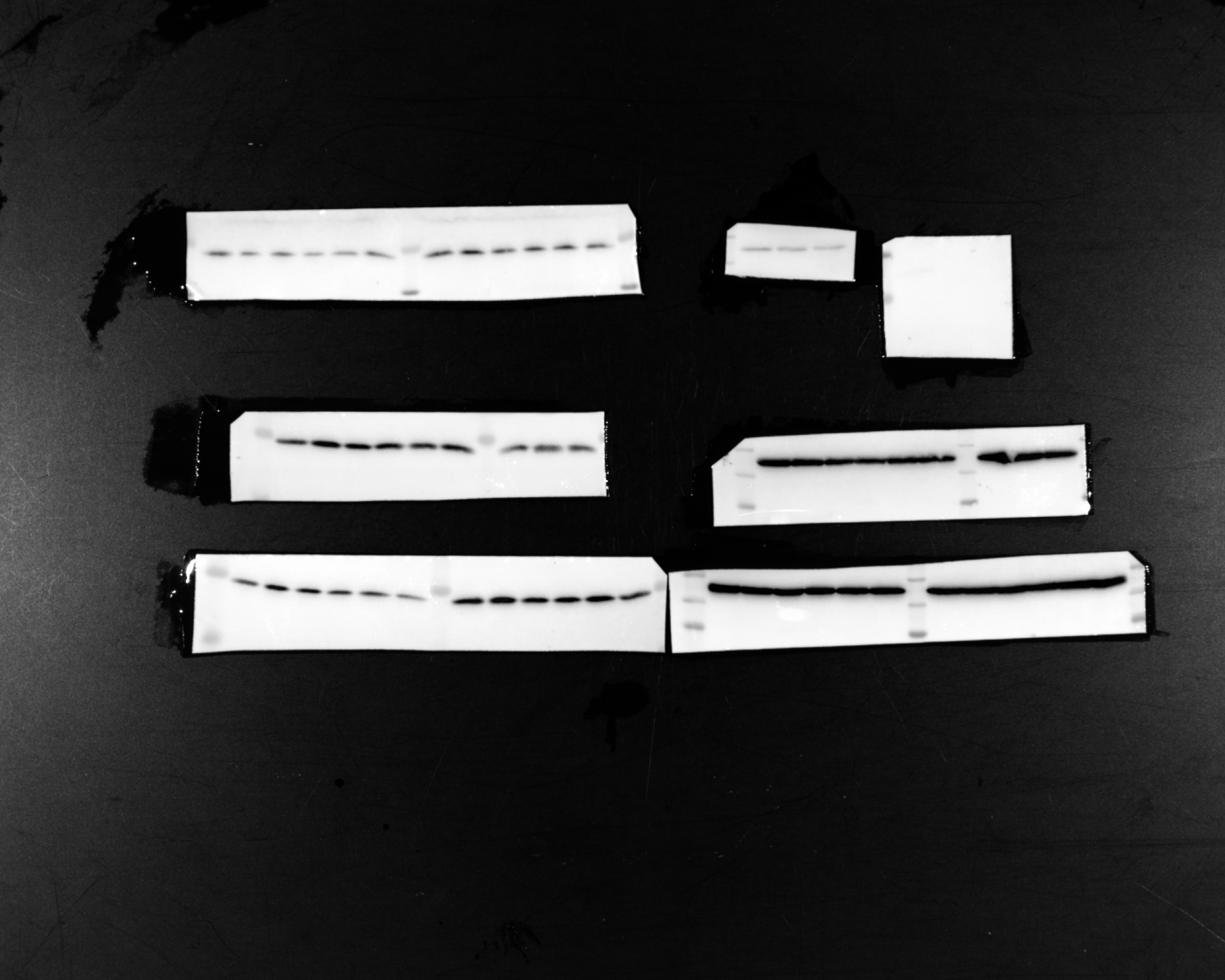

Supplement: Figure 1—figure supplement 1—source data 2. [file elife-101973-fig1-figsupp1-data2.zip › Figure 1-figure supplement 1-source data 2/Figure 1-figure supplement 1/VSV Figure 1–figure supplement 1B/HCT15 SW480 DLD1 SW620 ORMDL3 and tubulin.jpg]

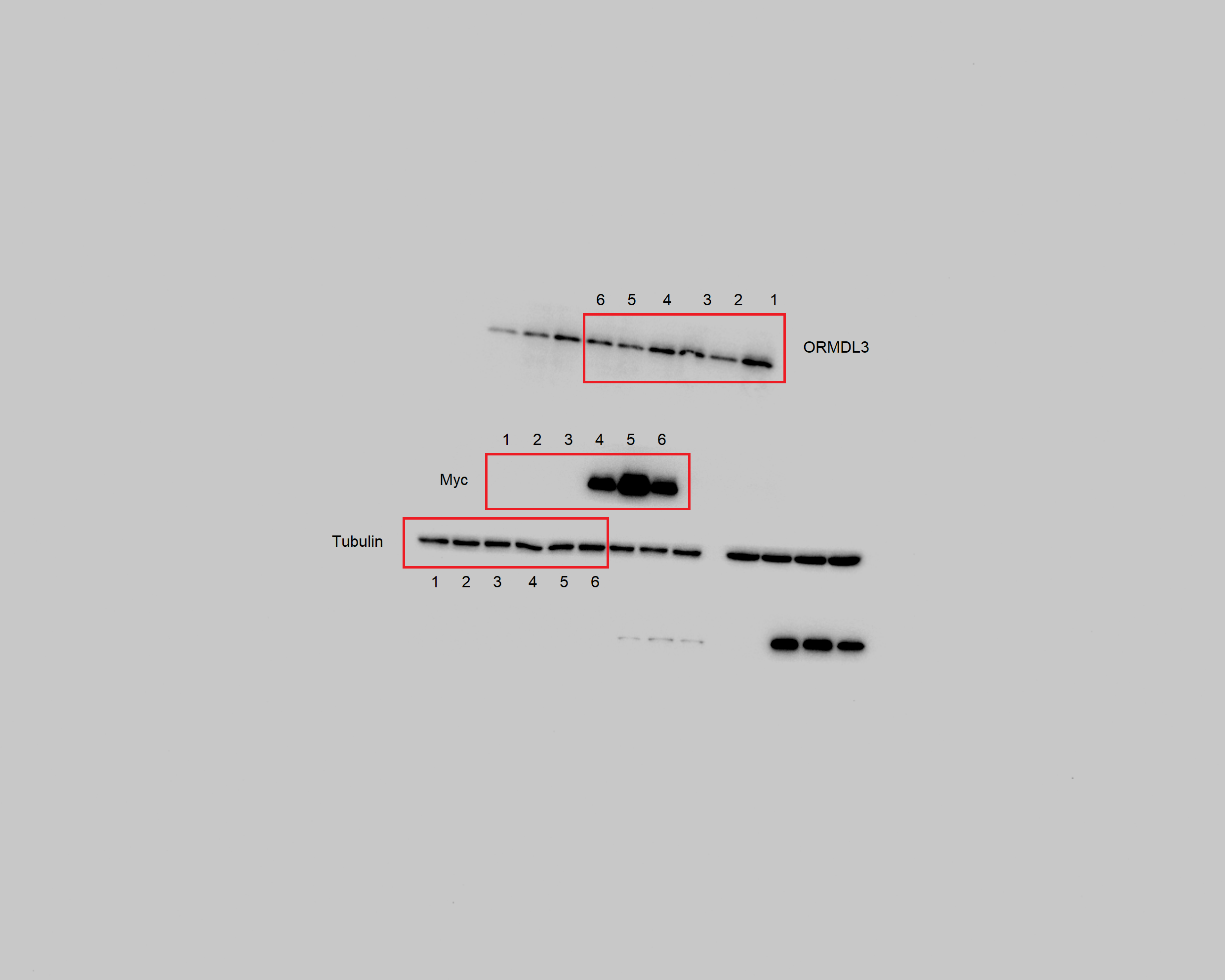

Supplement: Figure 2—source data 1. [file elife-101973-fig2-data1.zip › Figure 2-source data 1/Fig2D-labeled/Myc ORMDL3 and tubulin.tif]

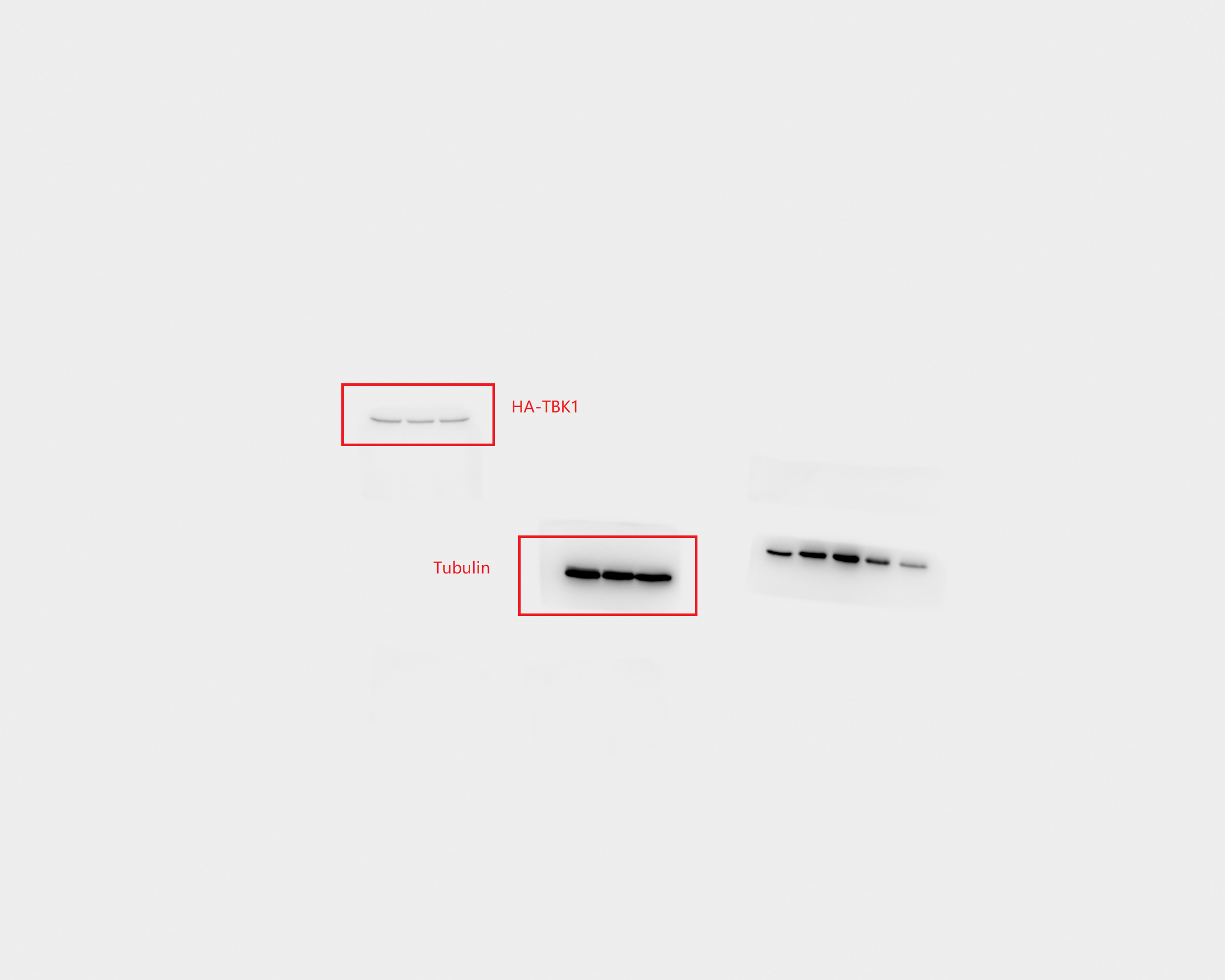

Supplement: Figure 2—source data 1. [file elife-101973-fig2-data1.zip › Figure 2-source data 1/Fig2E-labeled/HA-TBK1 and tubulin.tif]

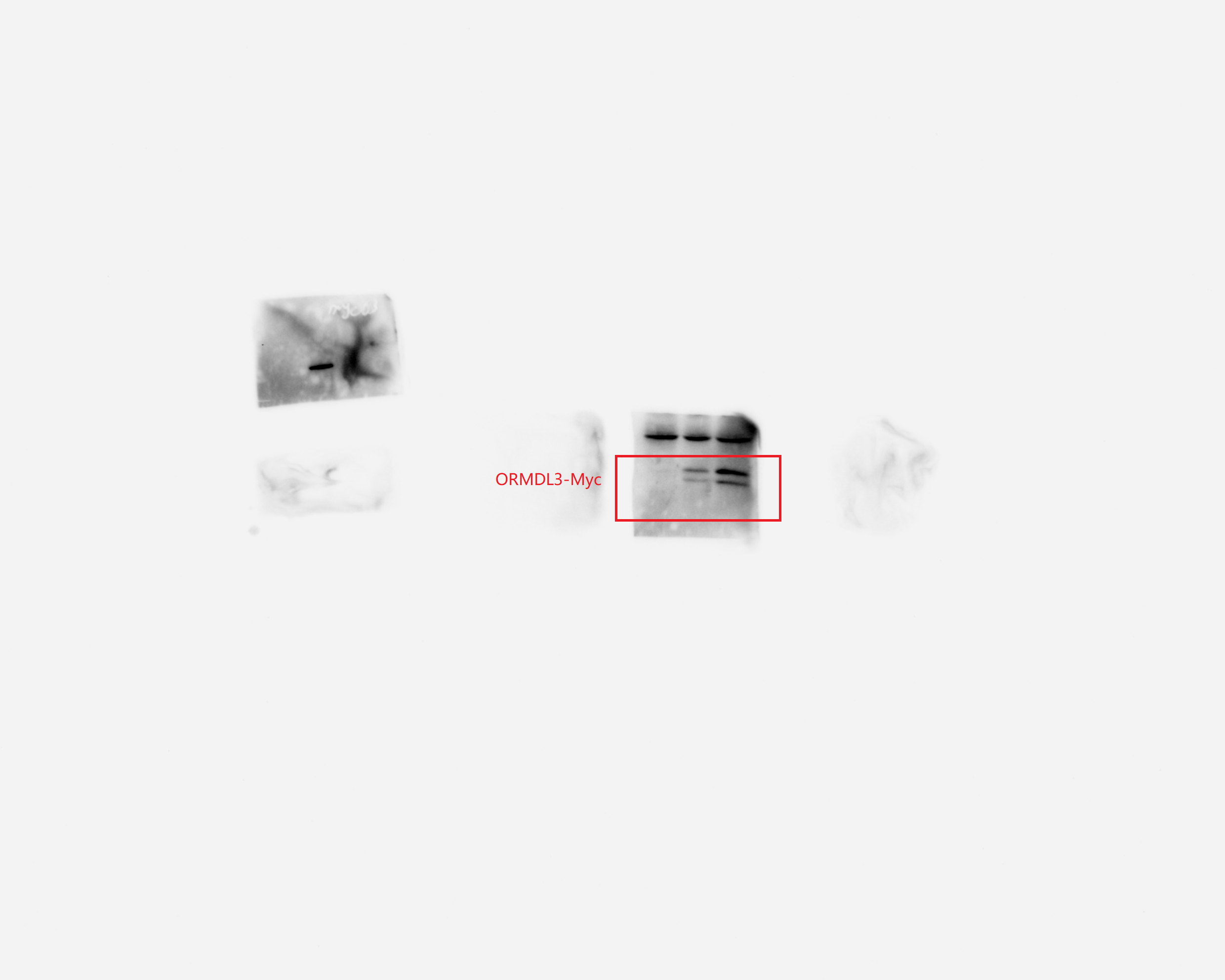

Supplement: Figure 2—source data 1. [file elife-101973-fig2-data1.zip › Figure 2-source data 1/Fig2E-labeled/ORMDL3-myc linked to HA-TBK1 .tif]

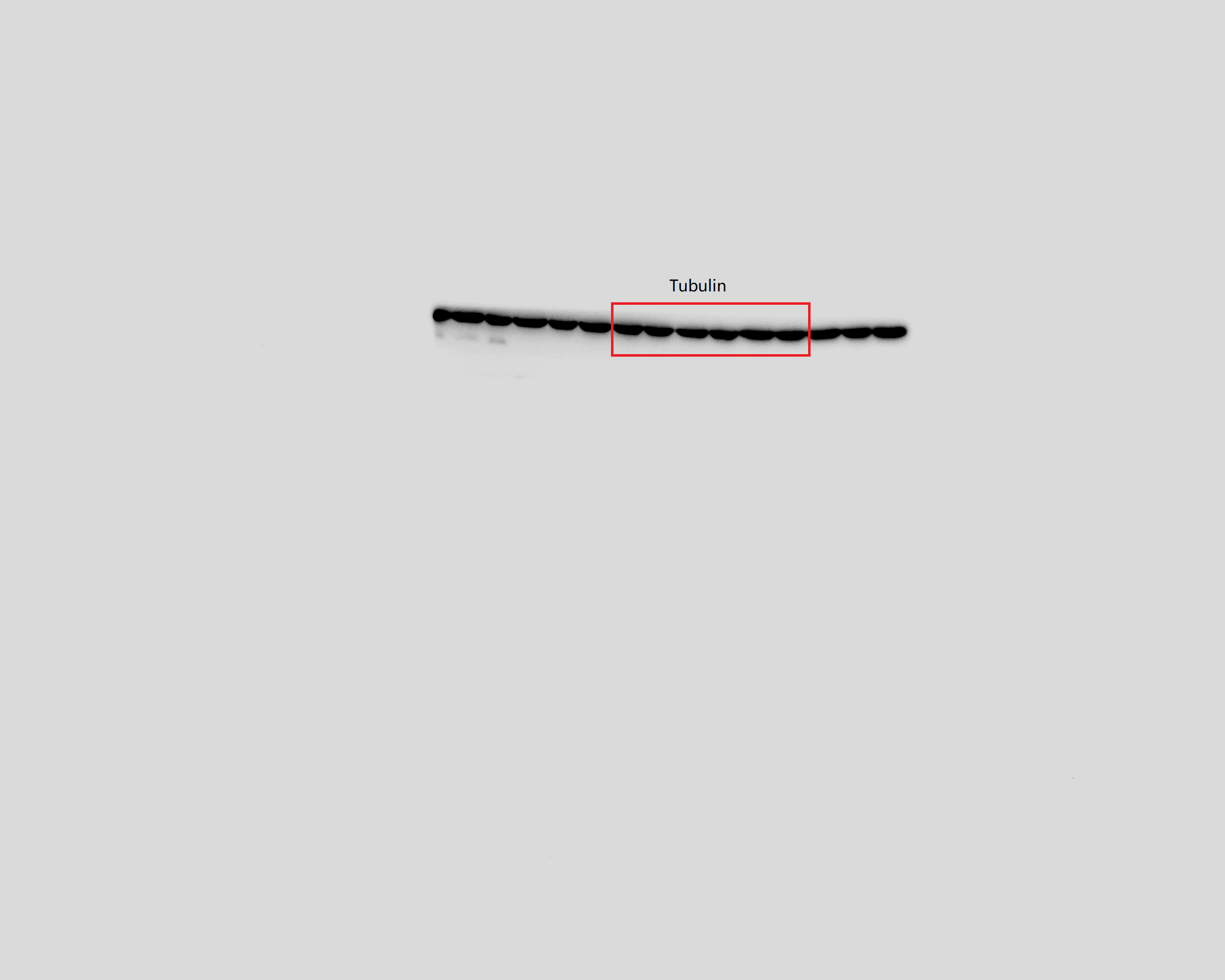

Supplement: Figure 2—source data 1. [file elife-101973-fig2-data1.zip › Figure 2-source data 1/Fig2E-labeled/Tubulin linked to flag-RIG-I MAVS.tif]

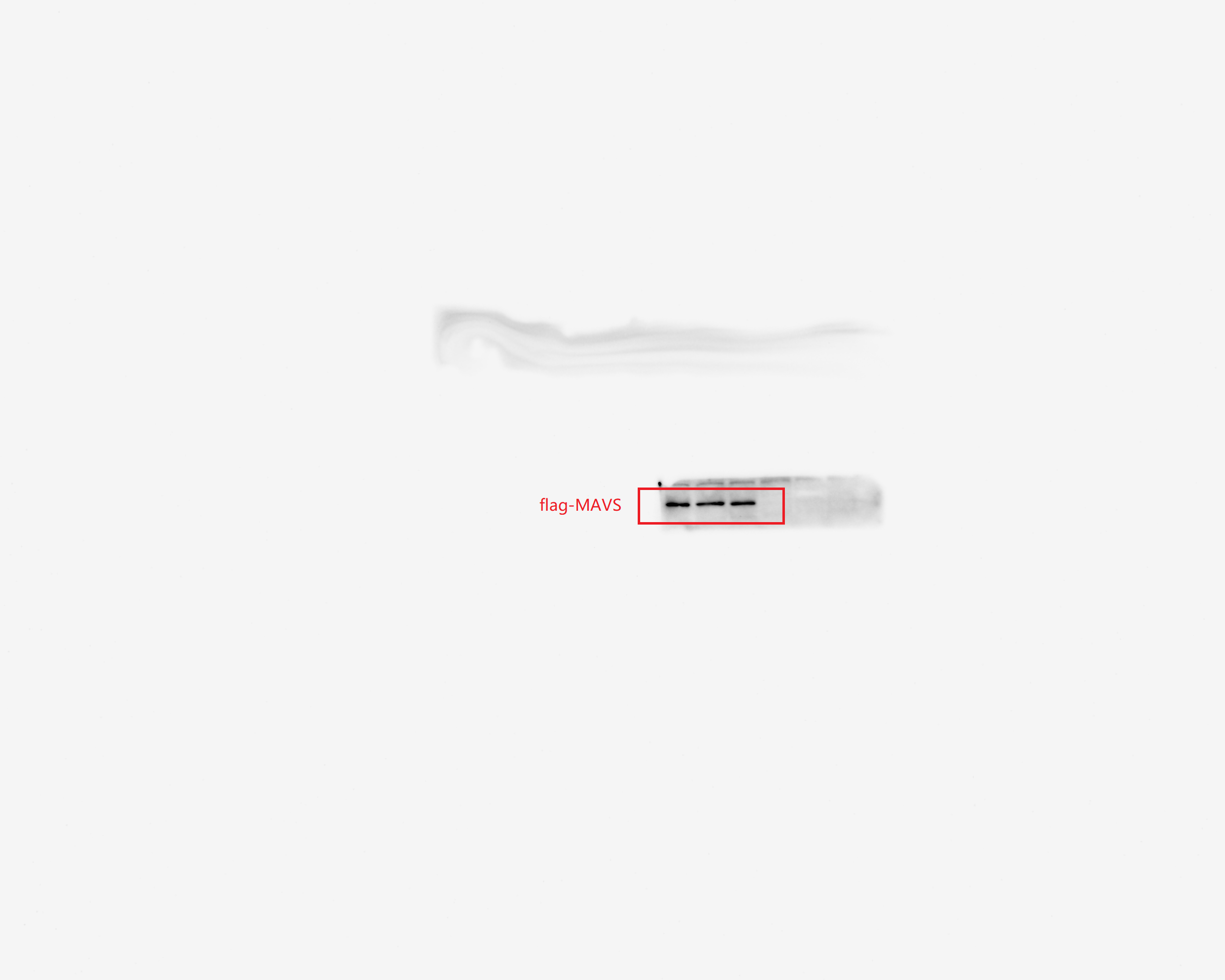

Supplement: Figure 2—source data 1. [file elife-101973-fig2-data1.zip › Figure 2-source data 1/Fig2E-labeled/flag-MAVS.tif]

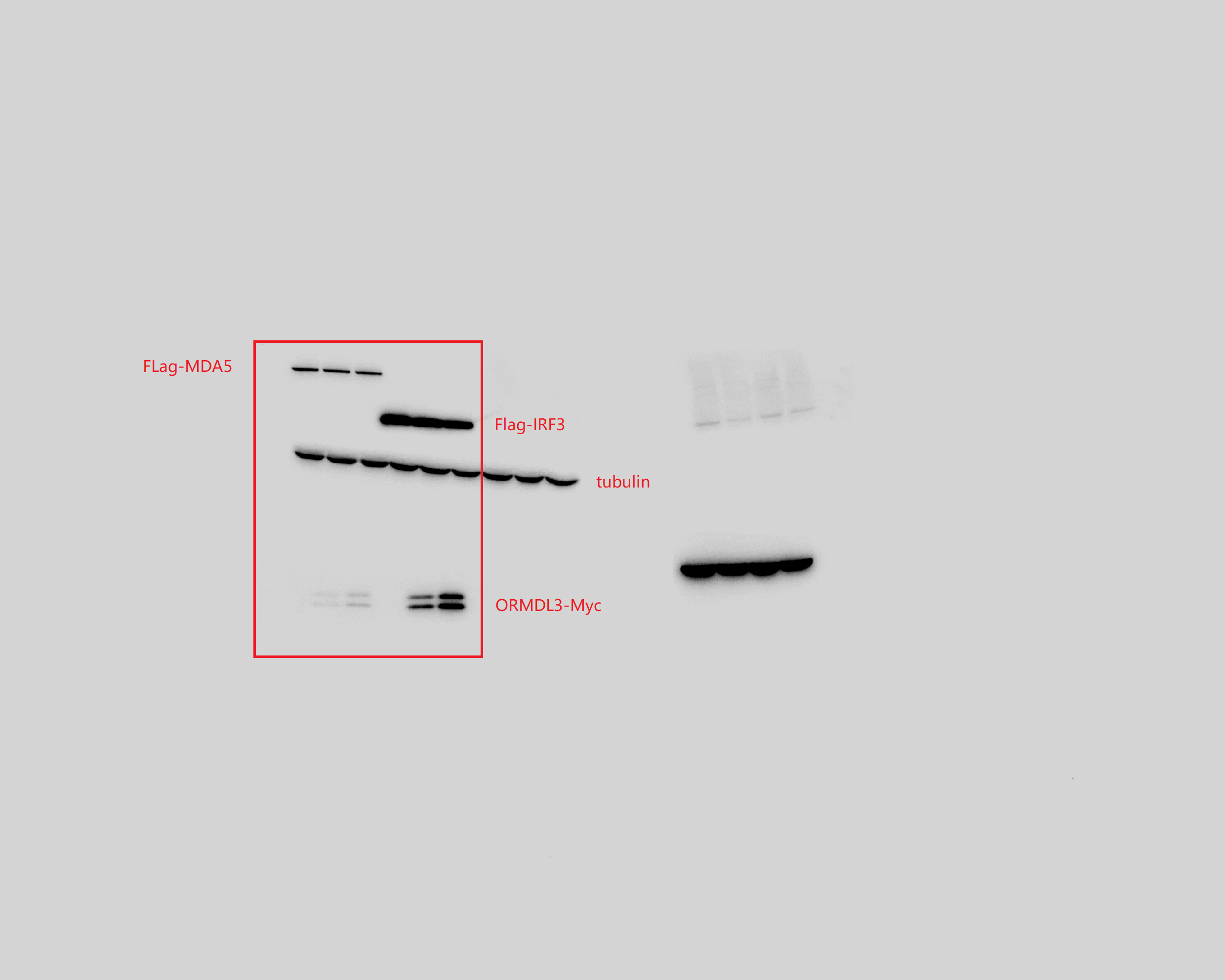

Supplement: Figure 2—source data 1. [file elife-101973-fig2-data1.zip › Figure 2-source data 1/Fig2E-labeled/flag-MDA5 Flag-IRF3 ORMDL3andtubulin.tif]

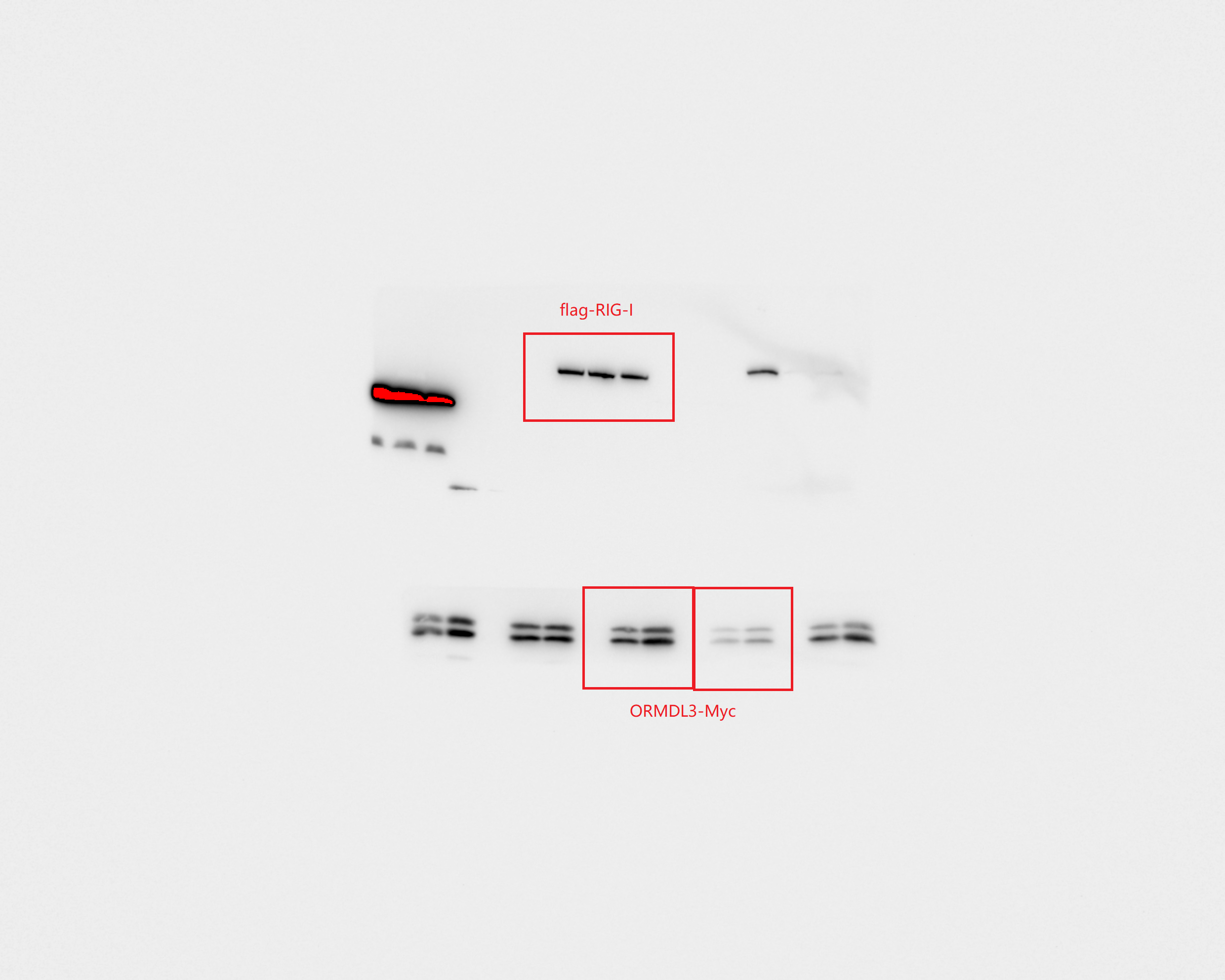

Supplement: Figure 2—source data 1. [file elife-101973-fig2-data1.zip › Figure 2-source data 1/Fig2E-labeled/flag-RIG-I and ORMDL3-myc linked to flag-RIG-I and flag-MAVS.tif]

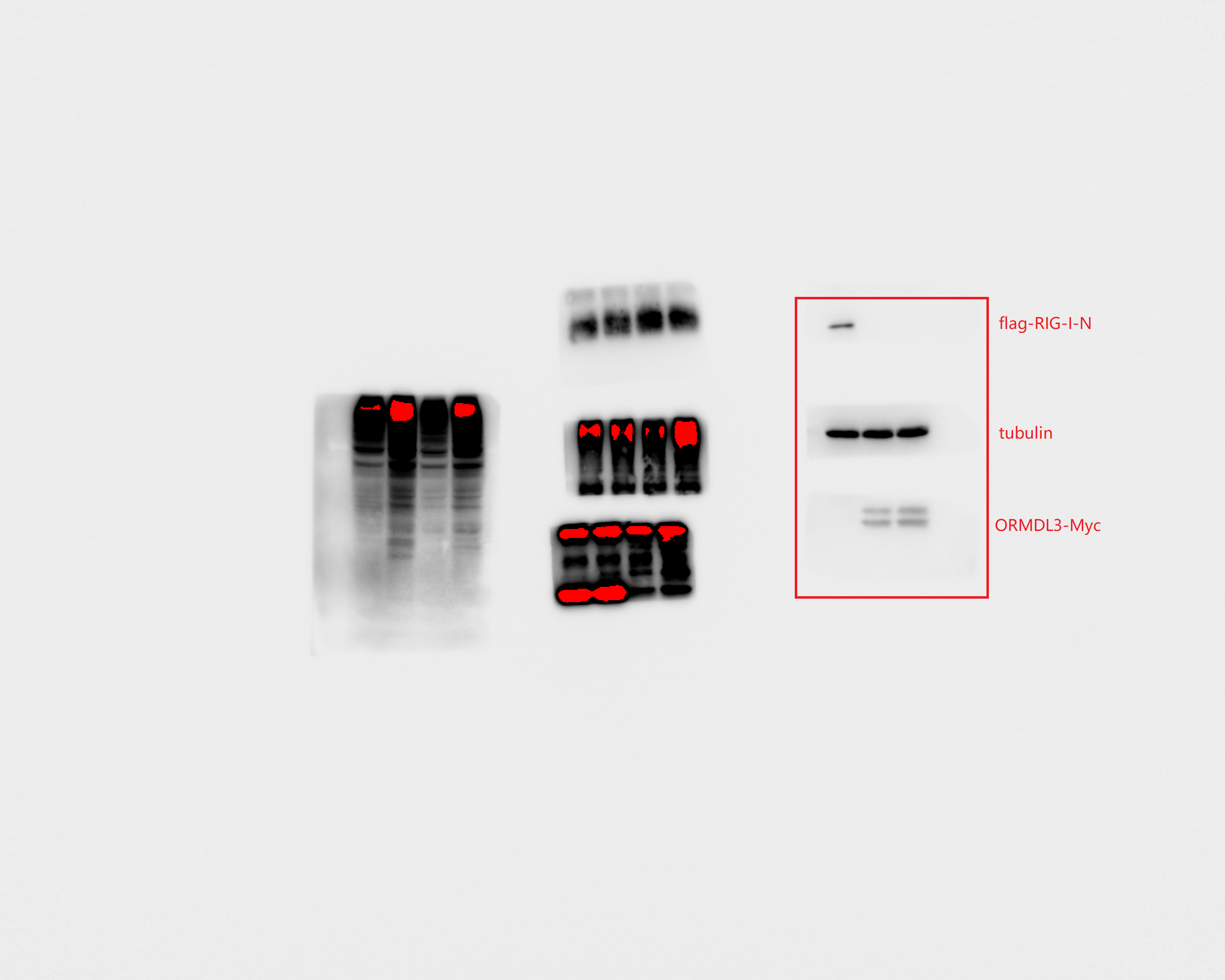

Supplement: Figure 2—source data 1. [file elife-101973-fig2-data1.zip › Figure 2-source data 1/Fig2E-labeled/flag-RIG-I-N ORMDL3-MycTubulin.tif]

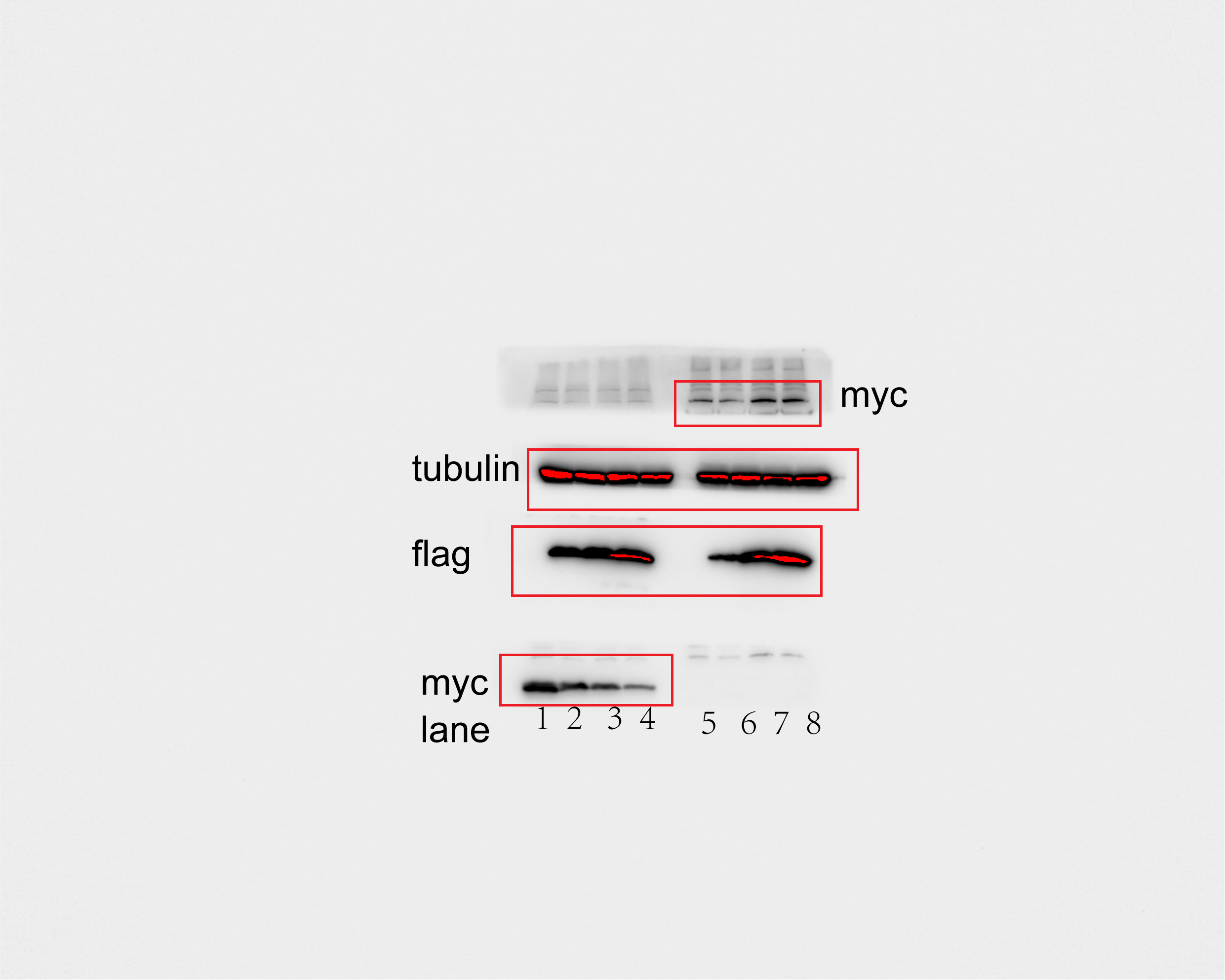

Supplement: Figure 2—source data 1. [file elife-101973-fig2-data1.zip › Figure 2-source data 1/Fig2F-labeled/Myc flag and Tubulin.tif]

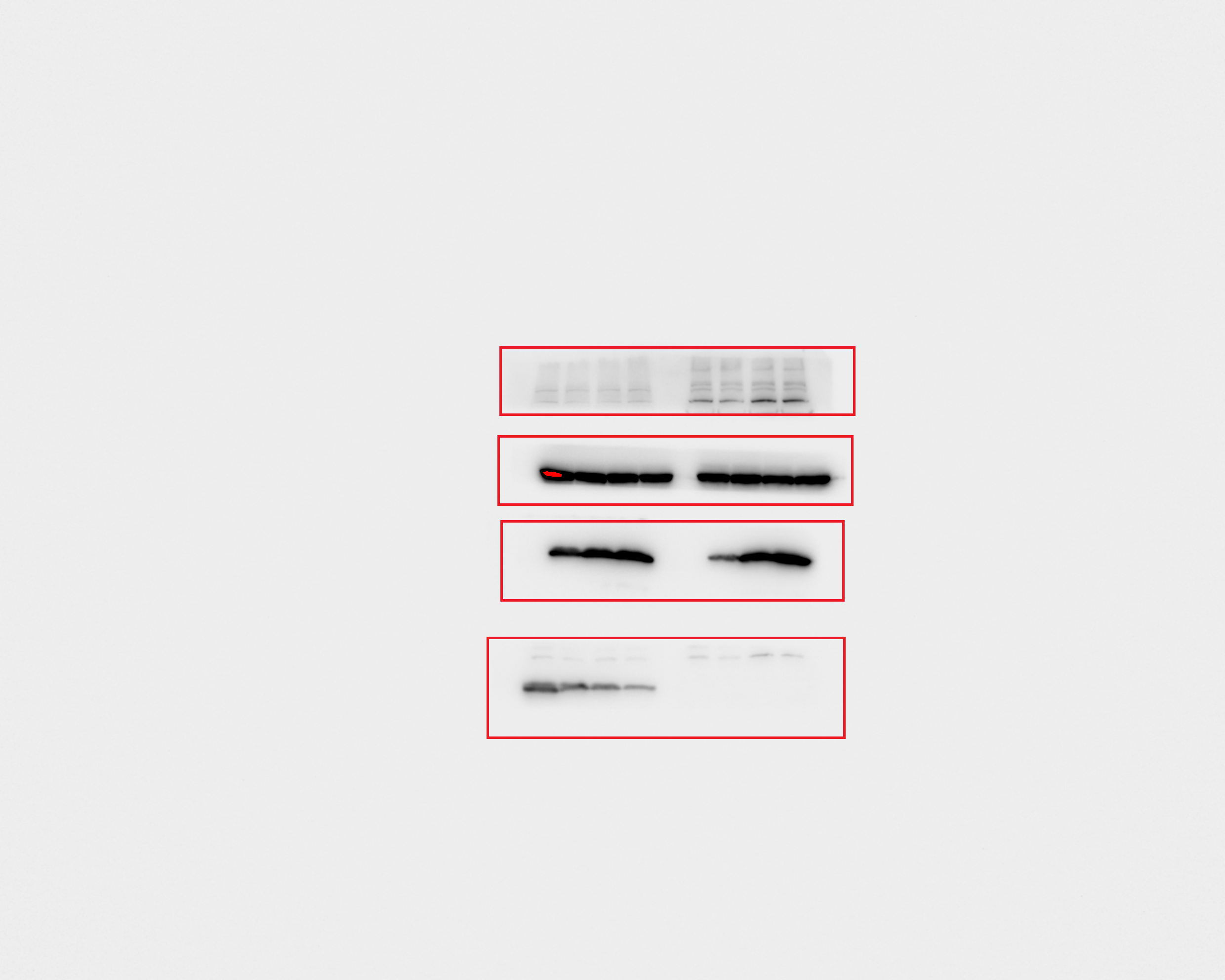

Supplement: Figure 2—source data 1. [file elife-101973-fig2-data1.zip › Figure 2-source data 1/Fig2F-labeled/short exposure.tif]

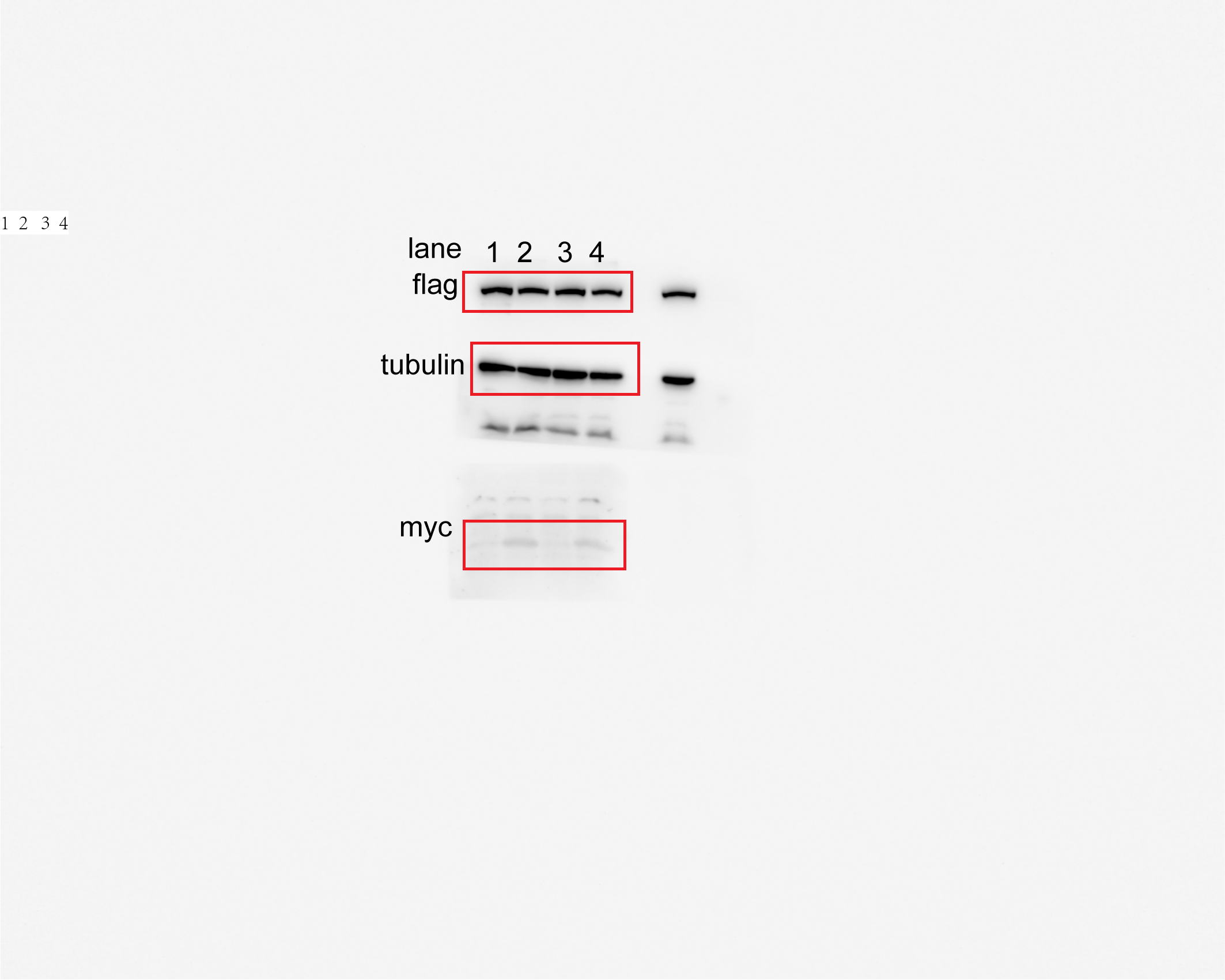

Supplement: Figure 2—source data 1. [file elife-101973-fig2-data1.zip › Figure 2-source data 1/Fig2G-labeled/Flag Myc and Tubulin.tif]

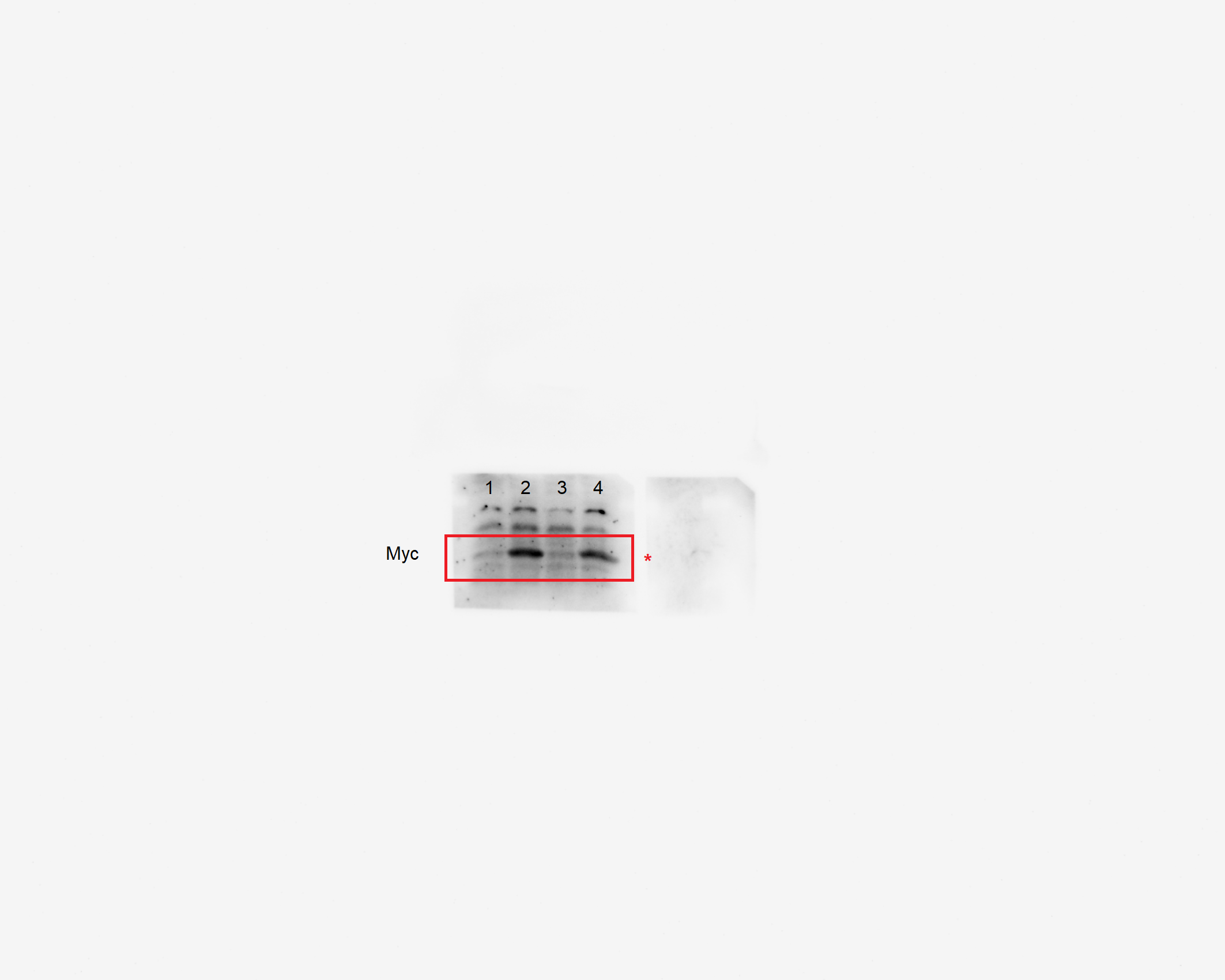

Supplement: Figure 2—source data 1. [file elife-101973-fig2-data1.zip › Figure 2-source data 1/Fig2G-labeled/long exposure of Myc .tif]

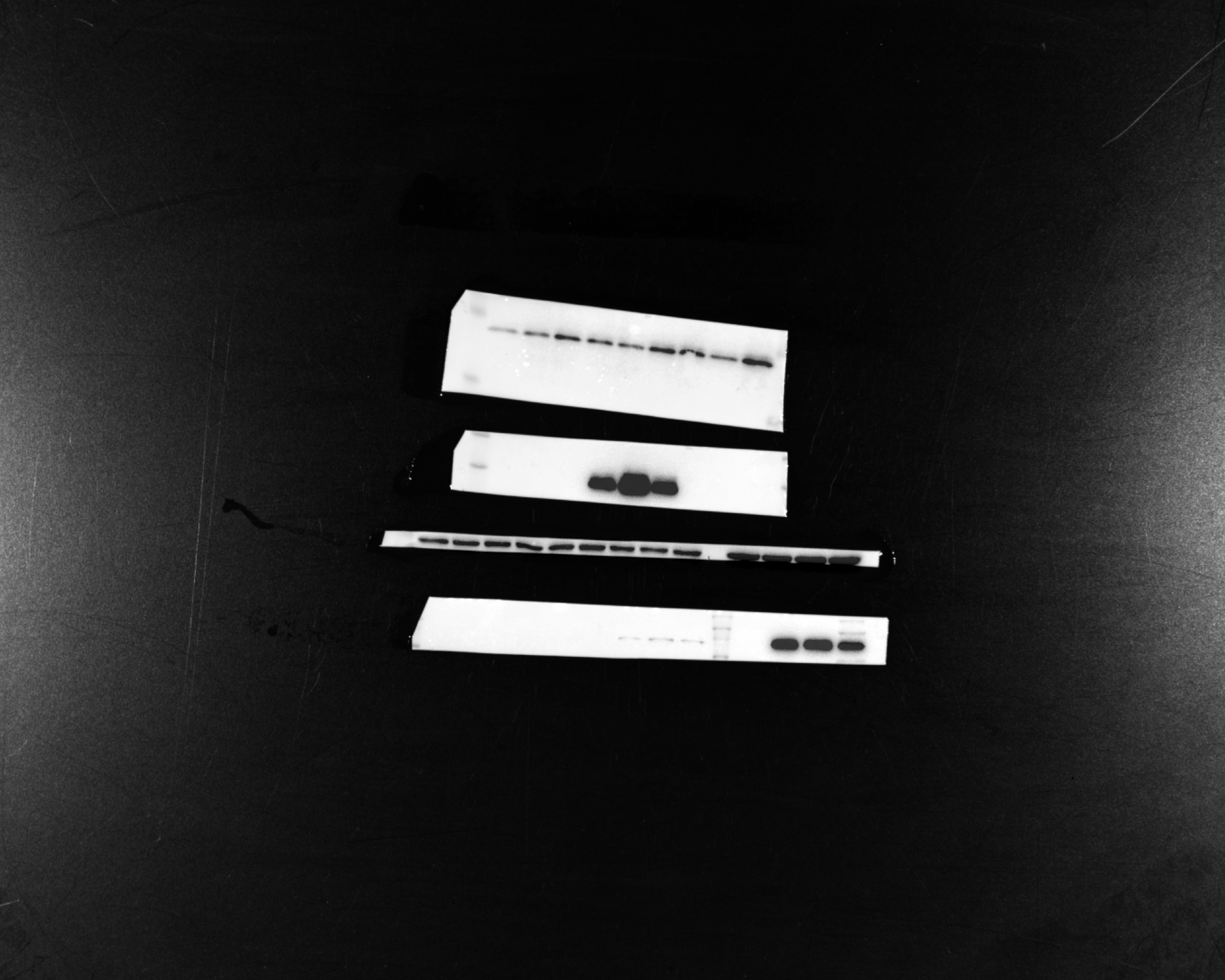

Supplement: Figure 2—source data 2. [file elife-101973-fig2-data2.zip › Figure 2-source data 2/figure 2D/ORMDL3 RIG-I-N-myc and tubulin.jpg]

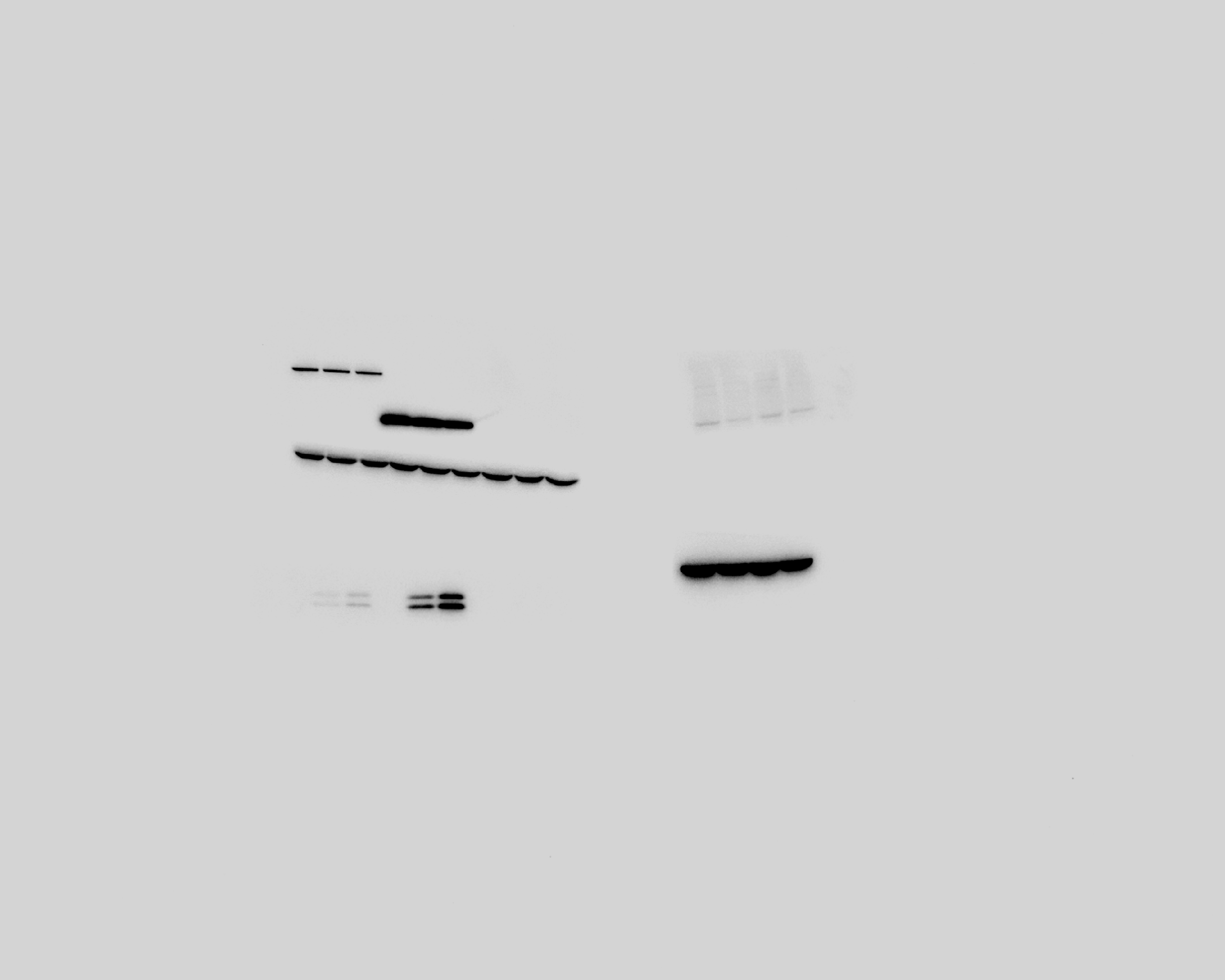

Supplement: Figure 2—source data 2. [file elife-101973-fig2-data2.zip › Figure 2-source data 2/figure 2E/Flag MDA5 IRF3 ORMDL3-Myc and tubulin.tif]

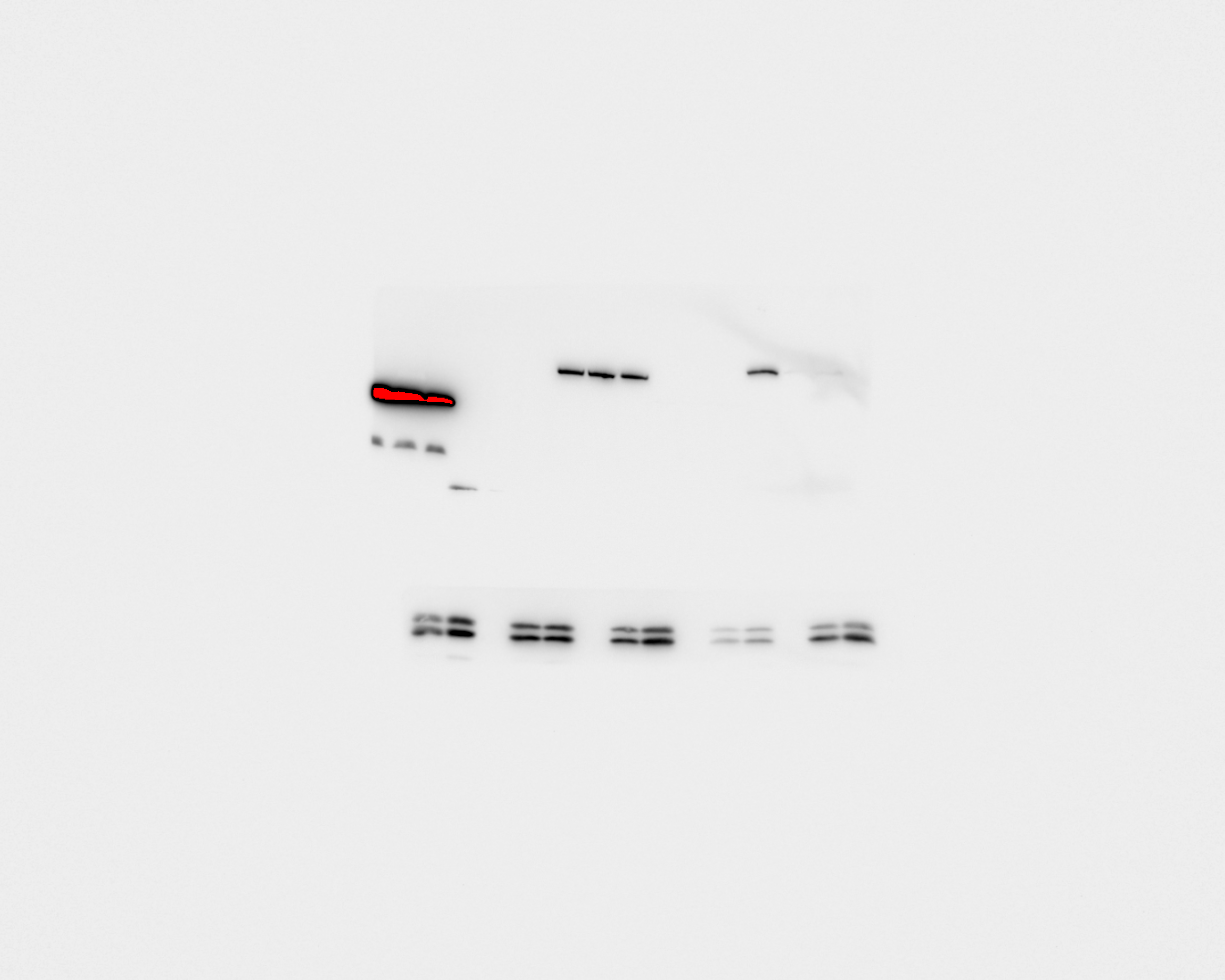

Supplement: Figure 2—source data 2. [file elife-101973-fig2-data2.zip › Figure 2-source data 2/figure 2E/Flag-RIG-I MAVS ORMDL3-Myc.tif]

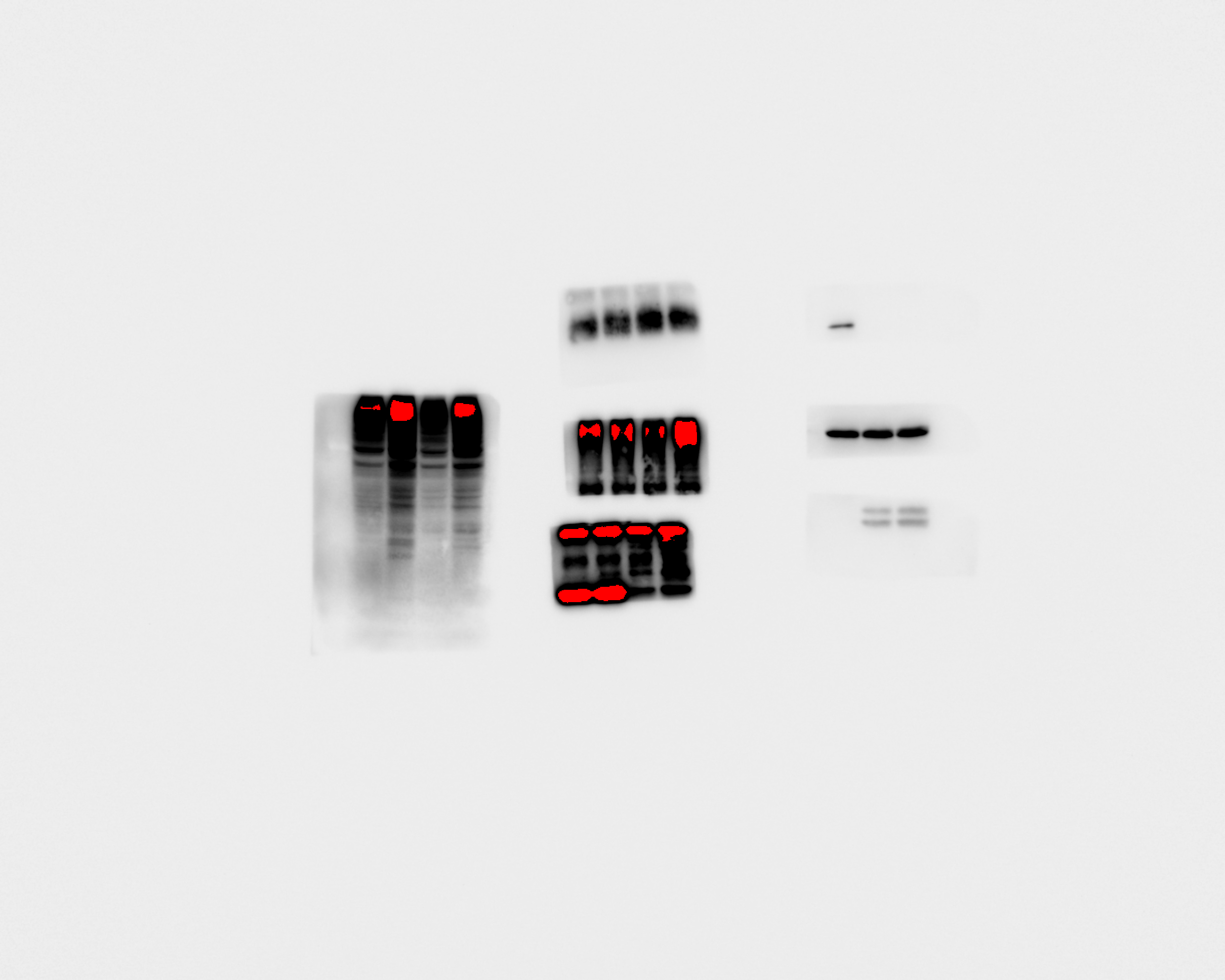

Supplement: Figure 2—source data 2. [file elife-101973-fig2-data2.zip › Figure 2-source data 2/figure 2E/Flag-RIG-I-N, ORMDL3 and tubulin.tif]

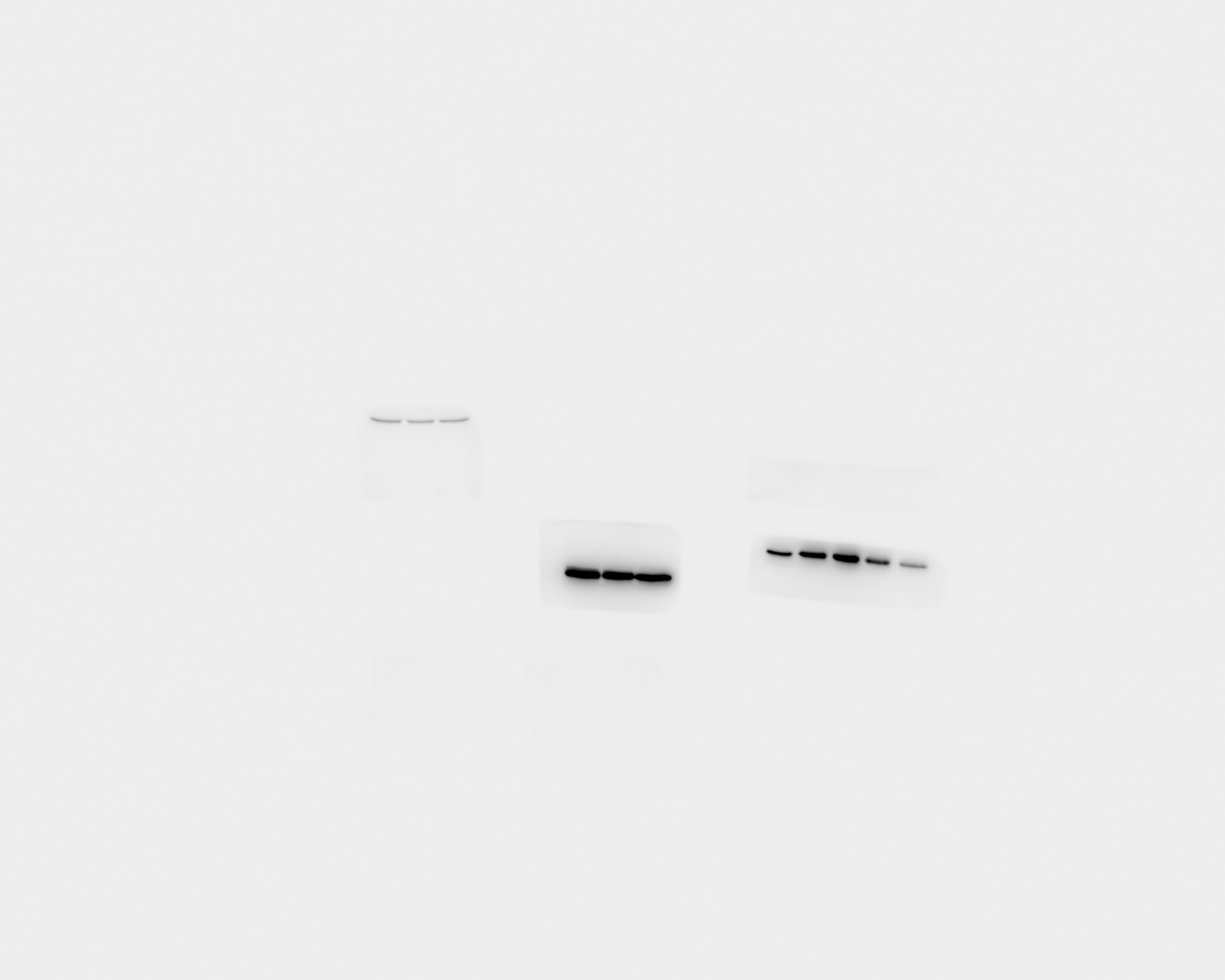

Supplement: Figure 2—source data 2. [file elife-101973-fig2-data2.zip › Figure 2-source data 2/figure 2E/HA-TBK1 and tubulin.tif]

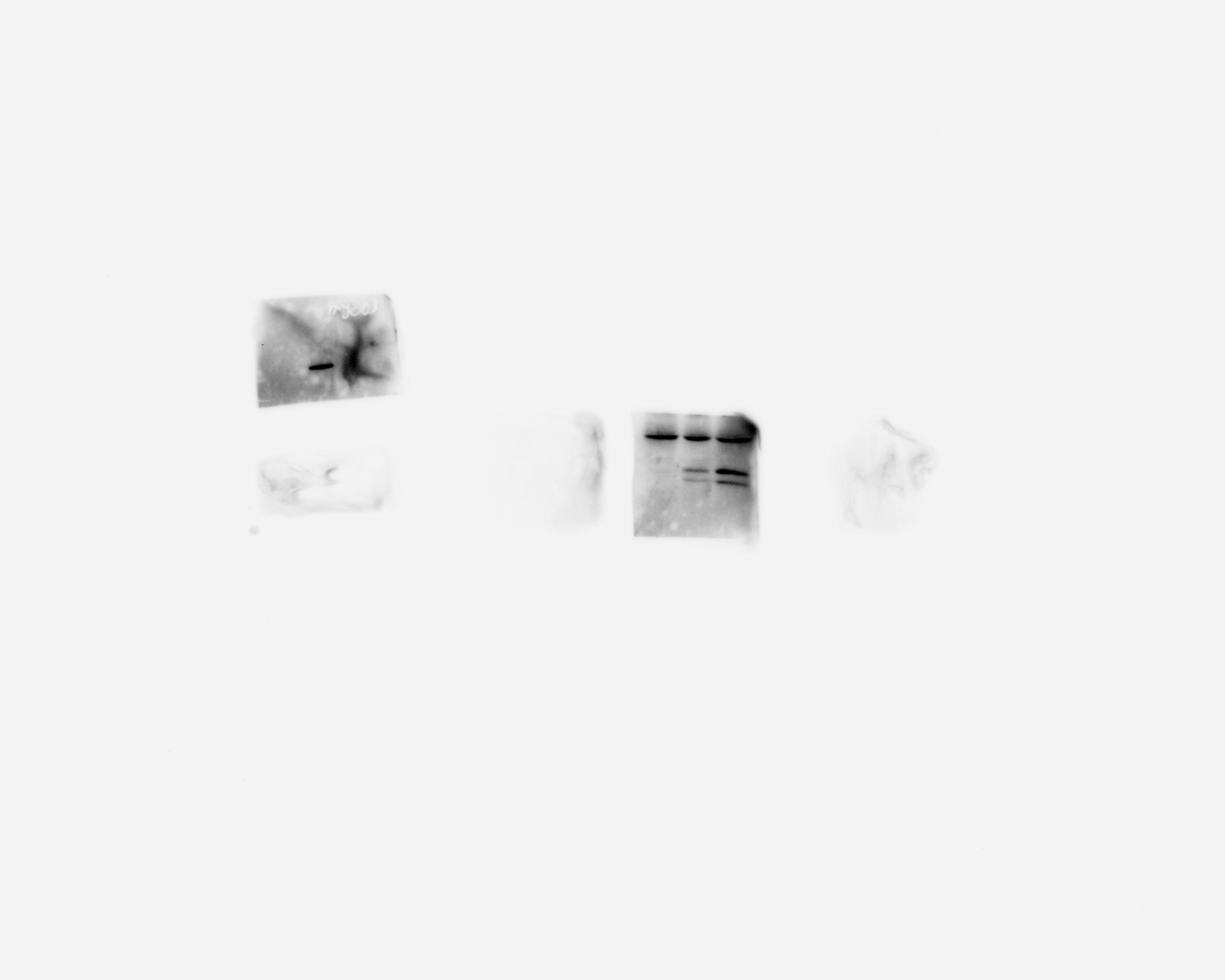

Supplement: Figure 2—source data 2. [file elife-101973-fig2-data2.zip › Figure 2-source data 2/figure 2E/ORMDL3-Myc linked to HA-TBK1.tif]

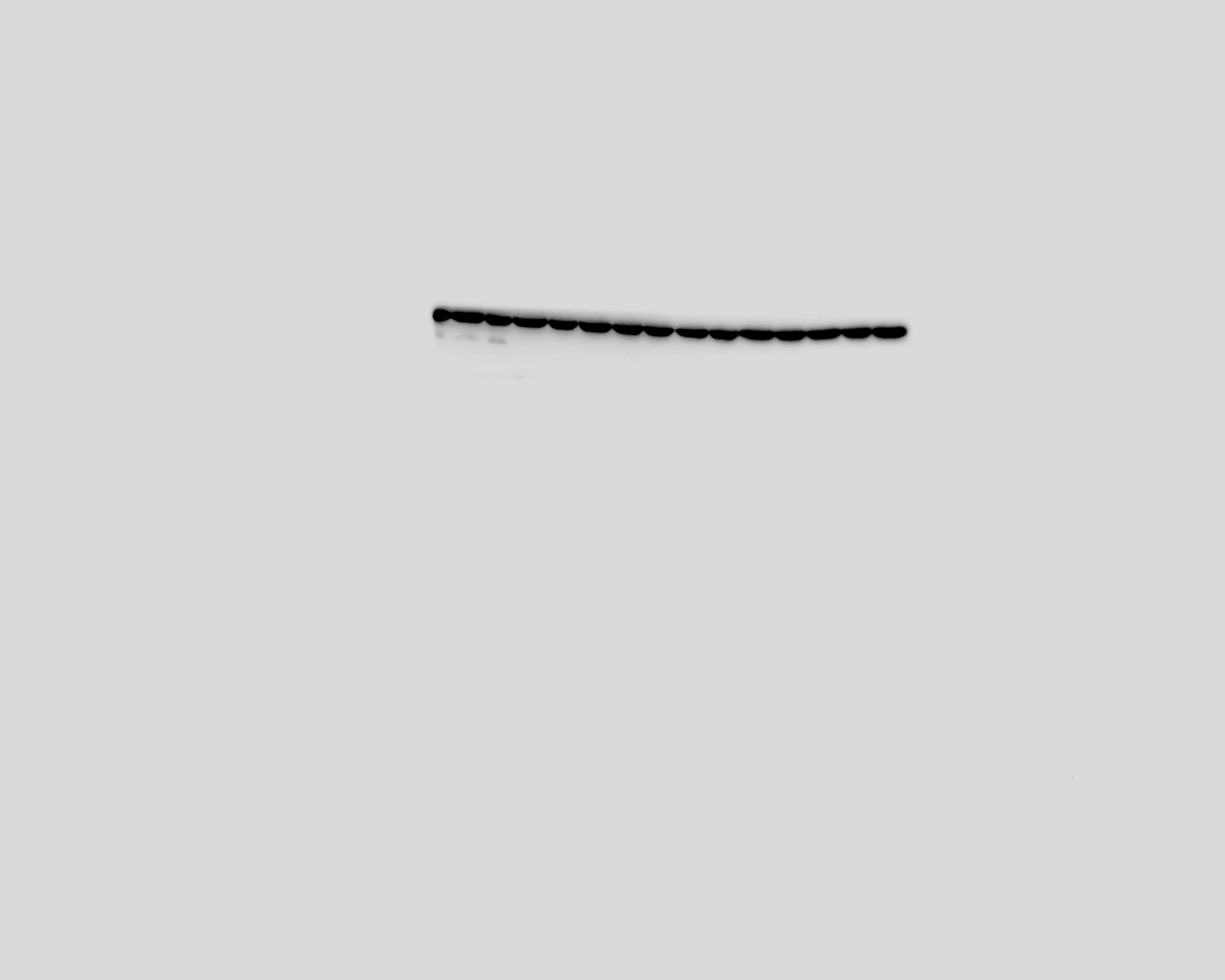

Supplement: Figure 2—source data 2. [file elife-101973-fig2-data2.zip › Figure 2-source data 2/figure 2E/Tubulin link to Flag-RIG-I MAVS .tif]

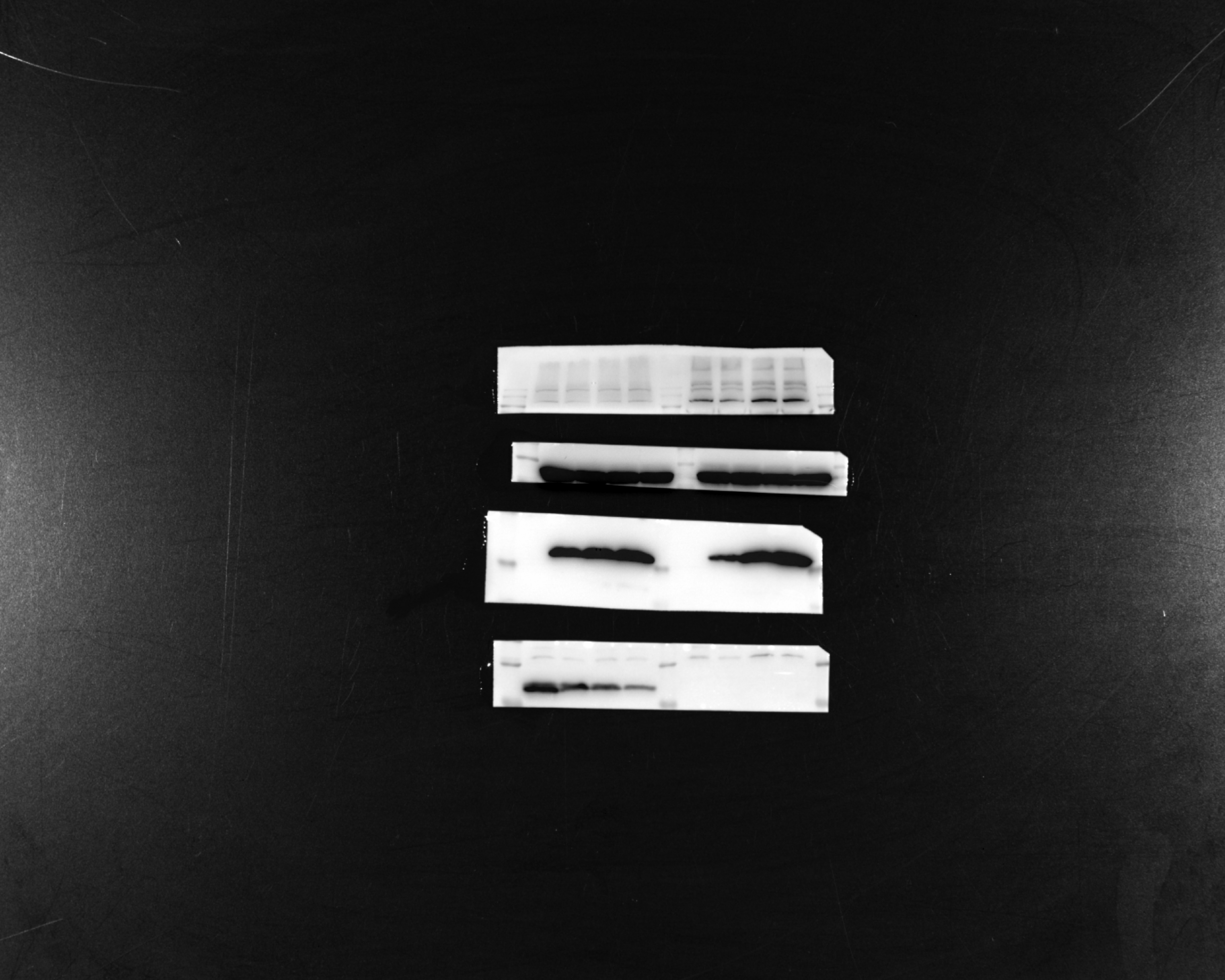

Supplement: Figure 2—source data 2. [file elife-101973-fig2-data2.zip › Figure 2-source data 2/figure 2F/myc flag and tubulin.jpg]

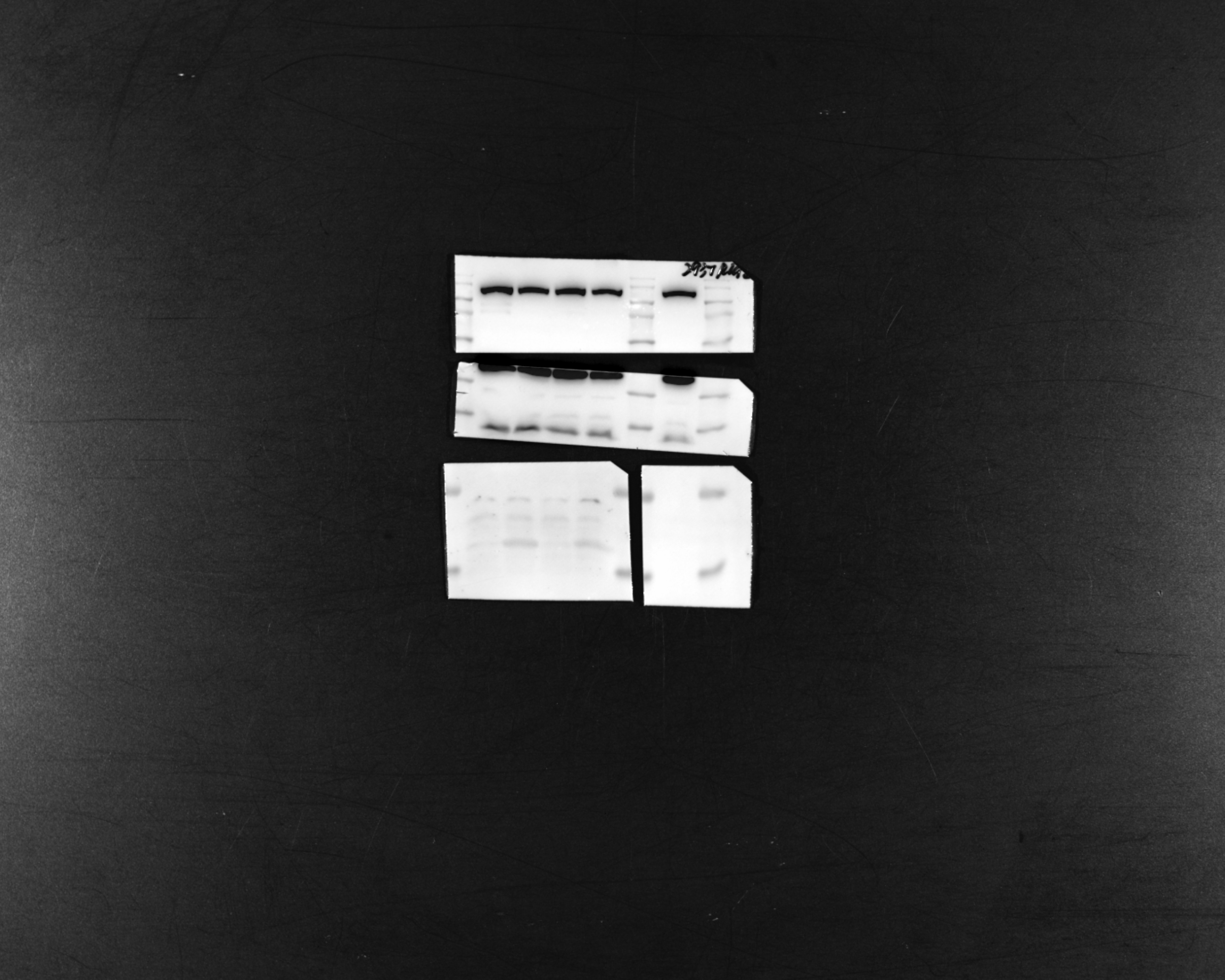

Supplement: Figure 2—source data 2. [file elife-101973-fig2-data2.zip › Figure 2-source data 2/figure 2G/flag myc and tubulin.jpg]

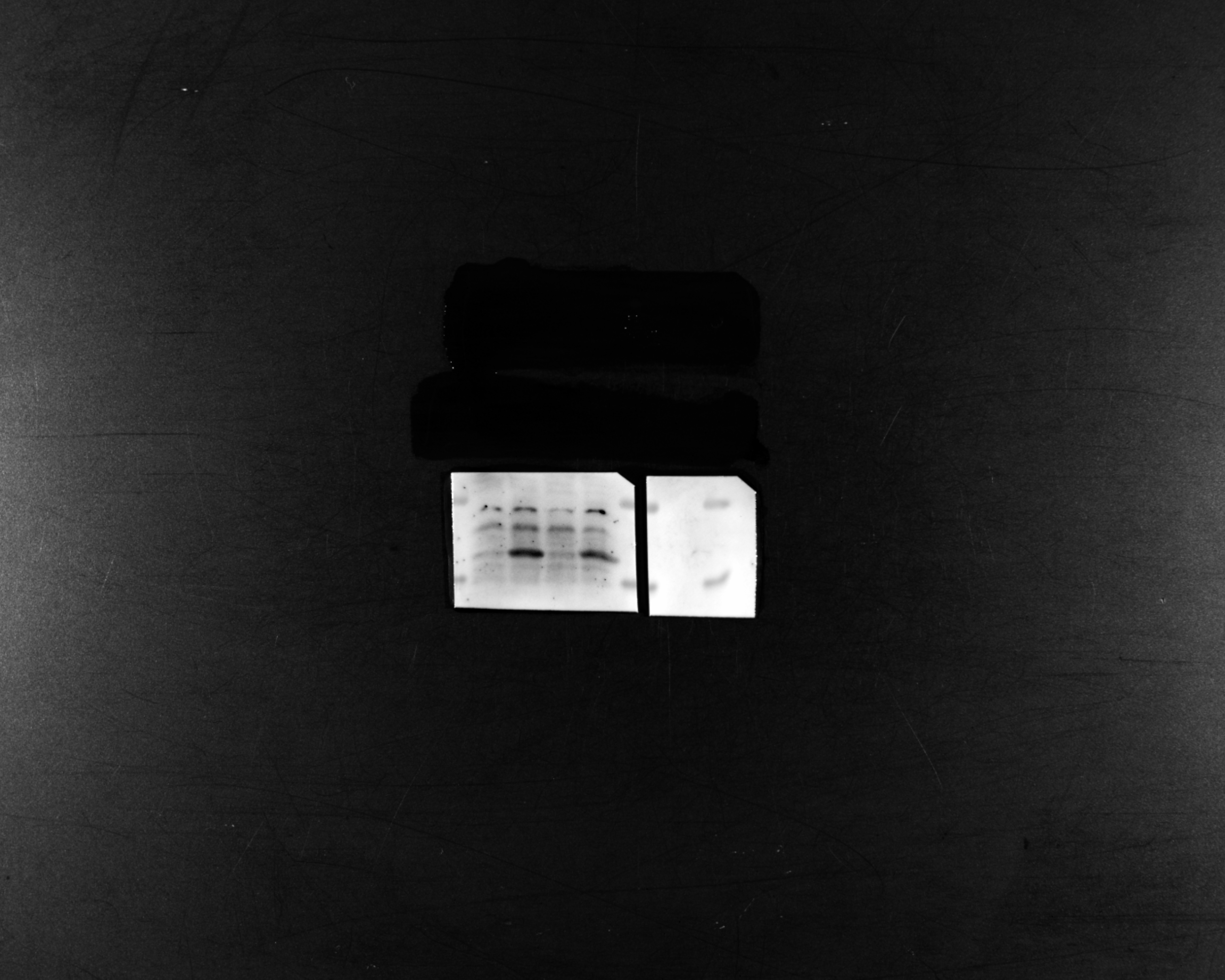

Supplement: Figure 2—source data 2. [file elife-101973-fig2-data2.zip › Figure 2-source data 2/figure 2G/long exposure of myc.jpg]

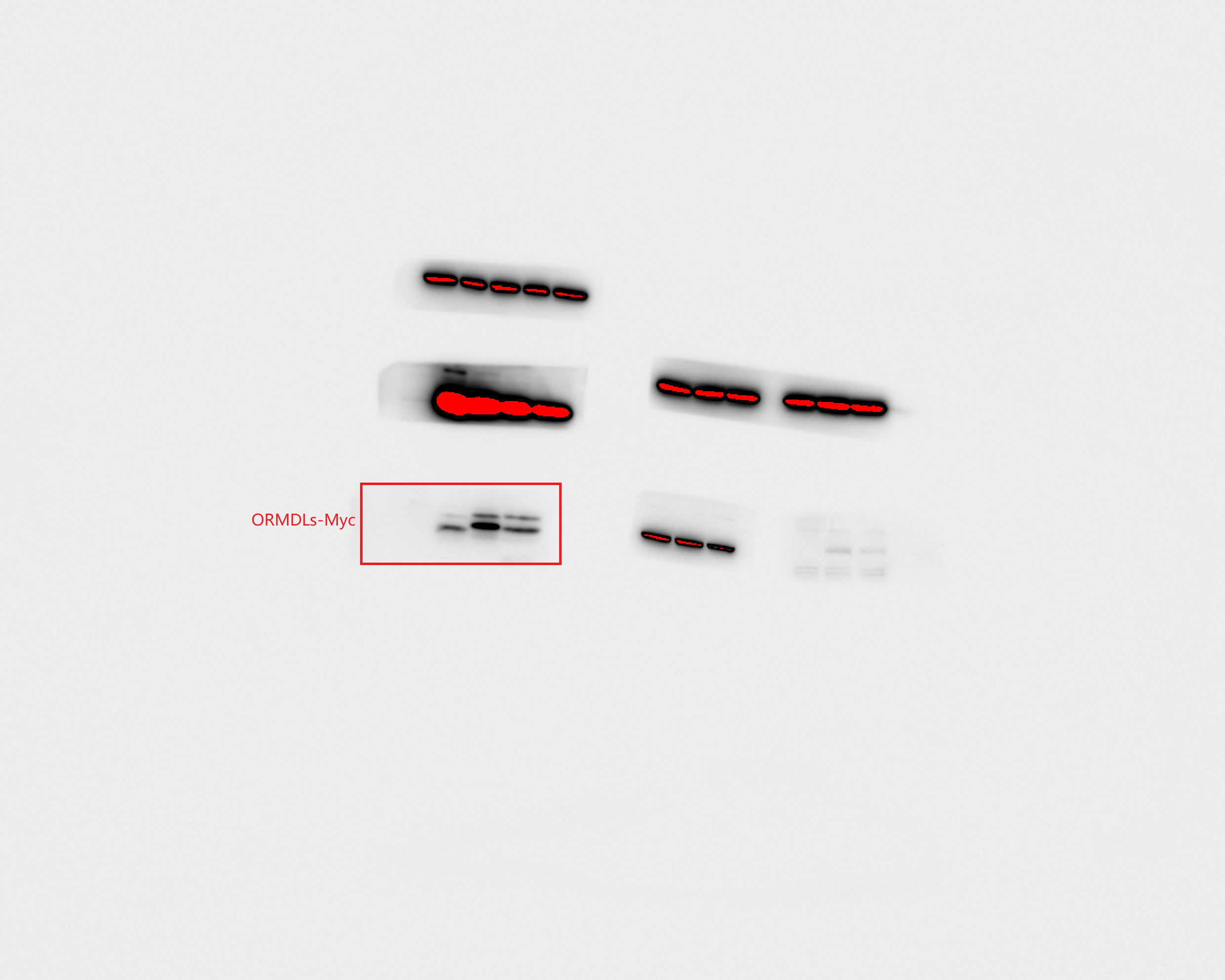

Supplement: Figure 2—figure supplement 1—source data 1. [file elife-101973-fig2-figsupp1-data1.zip › Figure 2–figure supplement 1-source data 1/Figure 2–figure supplement 1D-labeled/ORMDLs-Myc.tif]

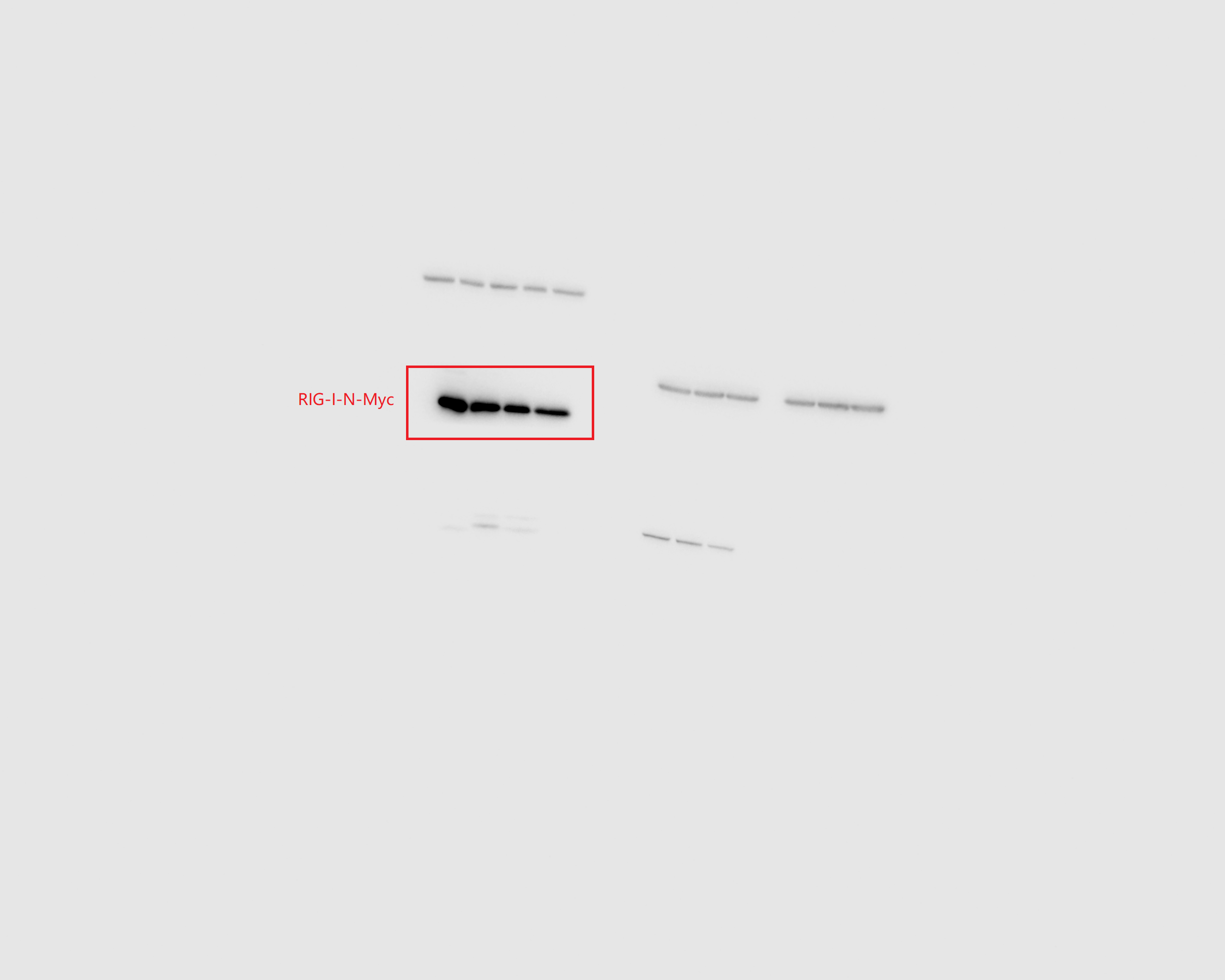

Supplement: Figure 2—figure supplement 1—source data 1. [file elife-101973-fig2-figsupp1-data1.zip › Figure 2–figure supplement 1-source data 1/Figure 2–figure supplement 1D-labeled/RIG-I-N-Myc.tif]

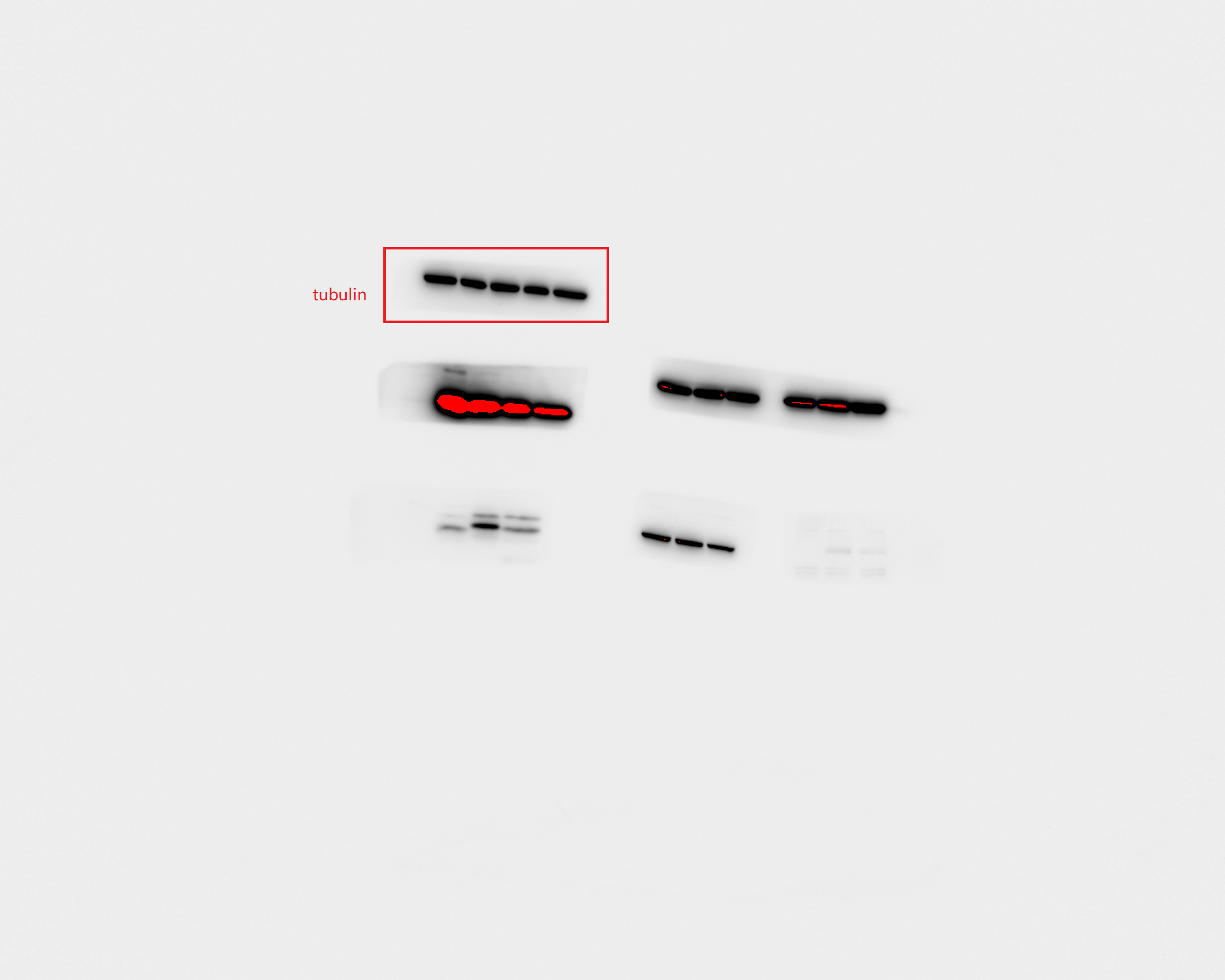

Supplement: Figure 2—figure supplement 1—source data 1. [file elife-101973-fig2-figsupp1-data1.zip › Figure 2–figure supplement 1-source data 1/Figure 2–figure supplement 1D-labeled/tubulin .tif]

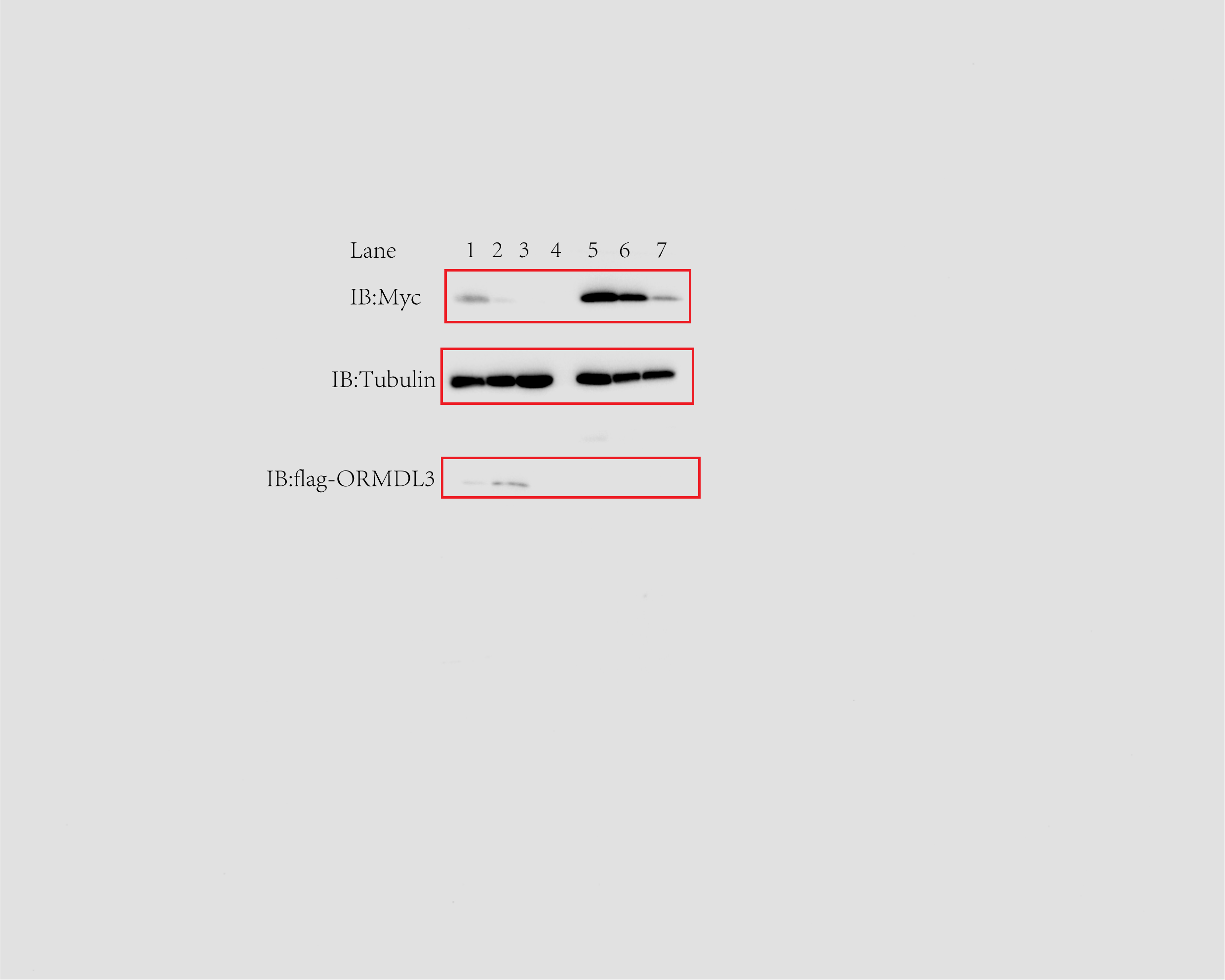

Supplement: Figure 2—figure supplement 1—source data 1. [file elife-101973-fig2-figsupp1-data1.zip › Figure 2–figure supplement 1-source data 1/Figure 2–figure supplement 1E 1F-labeled/Myc flag and tubulin.tif]

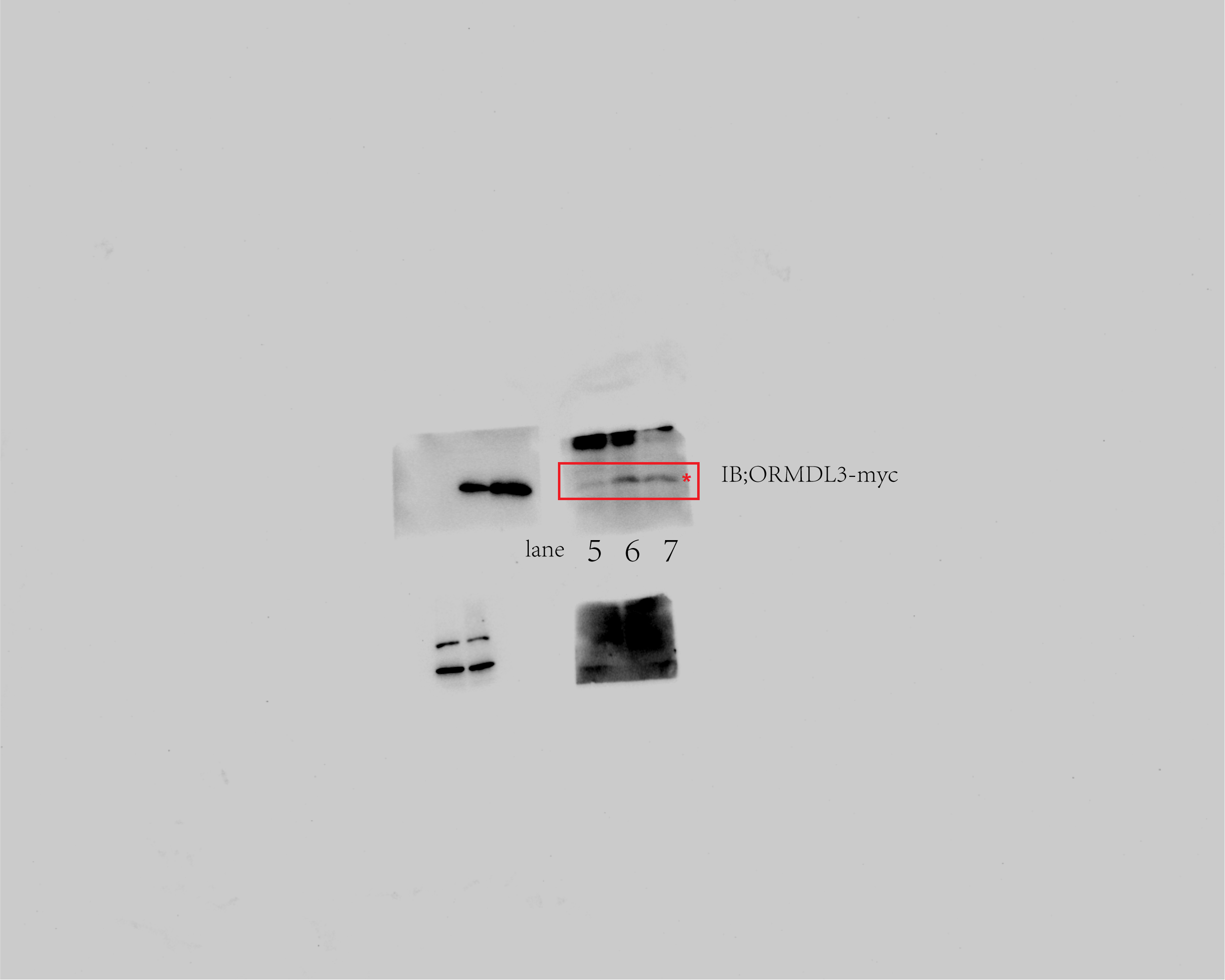

Supplement: Figure 2—figure supplement 1—source data 1. [file elife-101973-fig2-figsupp1-data1.zip › Figure 2–figure supplement 1-source data 1/Figure 2–figure supplement 1E 1F-labeled/ORMDL3-Myc.tif]

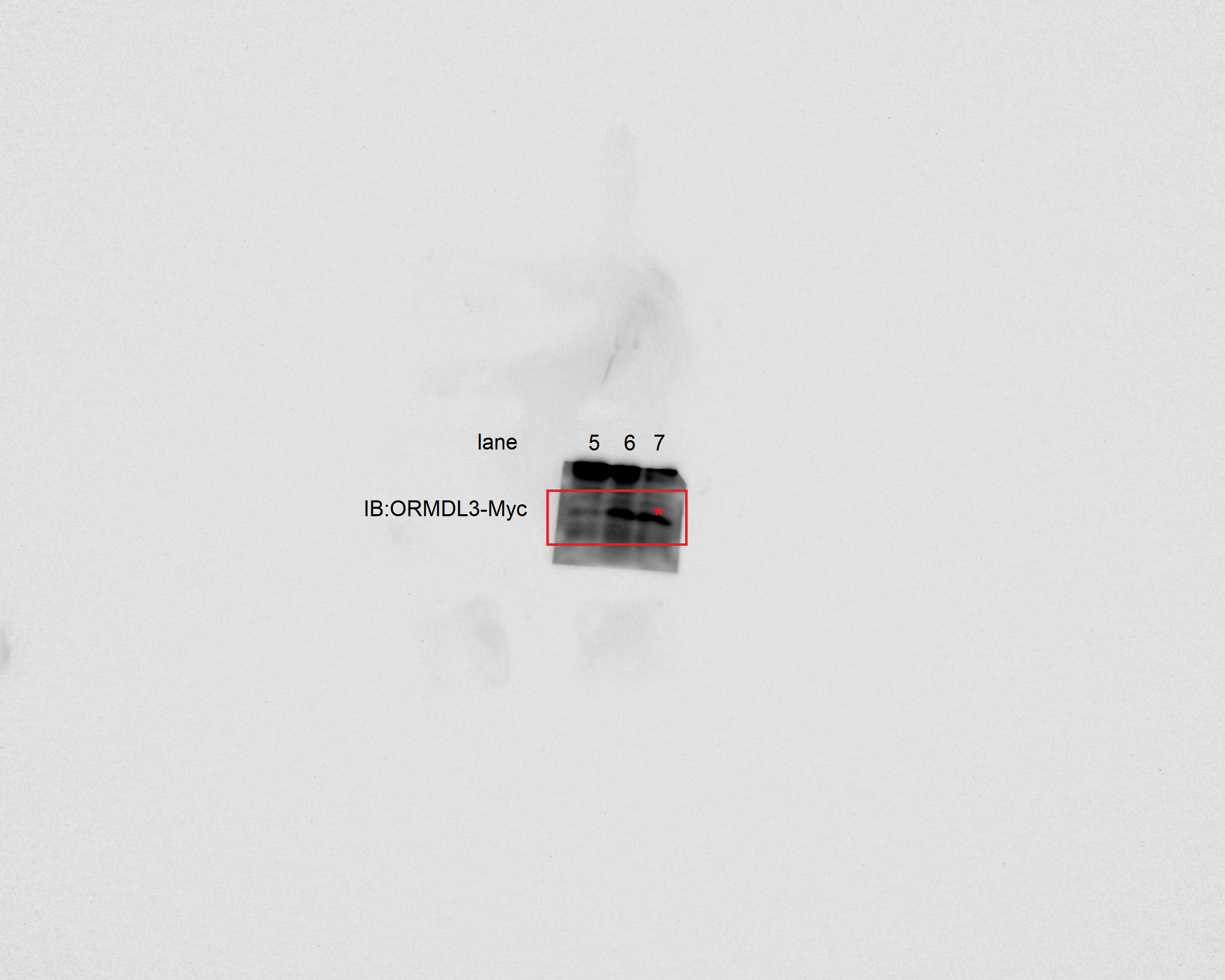

Supplement: Figure 2—figure supplement 1—source data 1. [file elife-101973-fig2-figsupp1-data1.zip › Figure 2–figure supplement 1-source data 1/Figure 2–figure supplement 1E 1F-labeled/long exposure of ORMDL3-myc.tif]

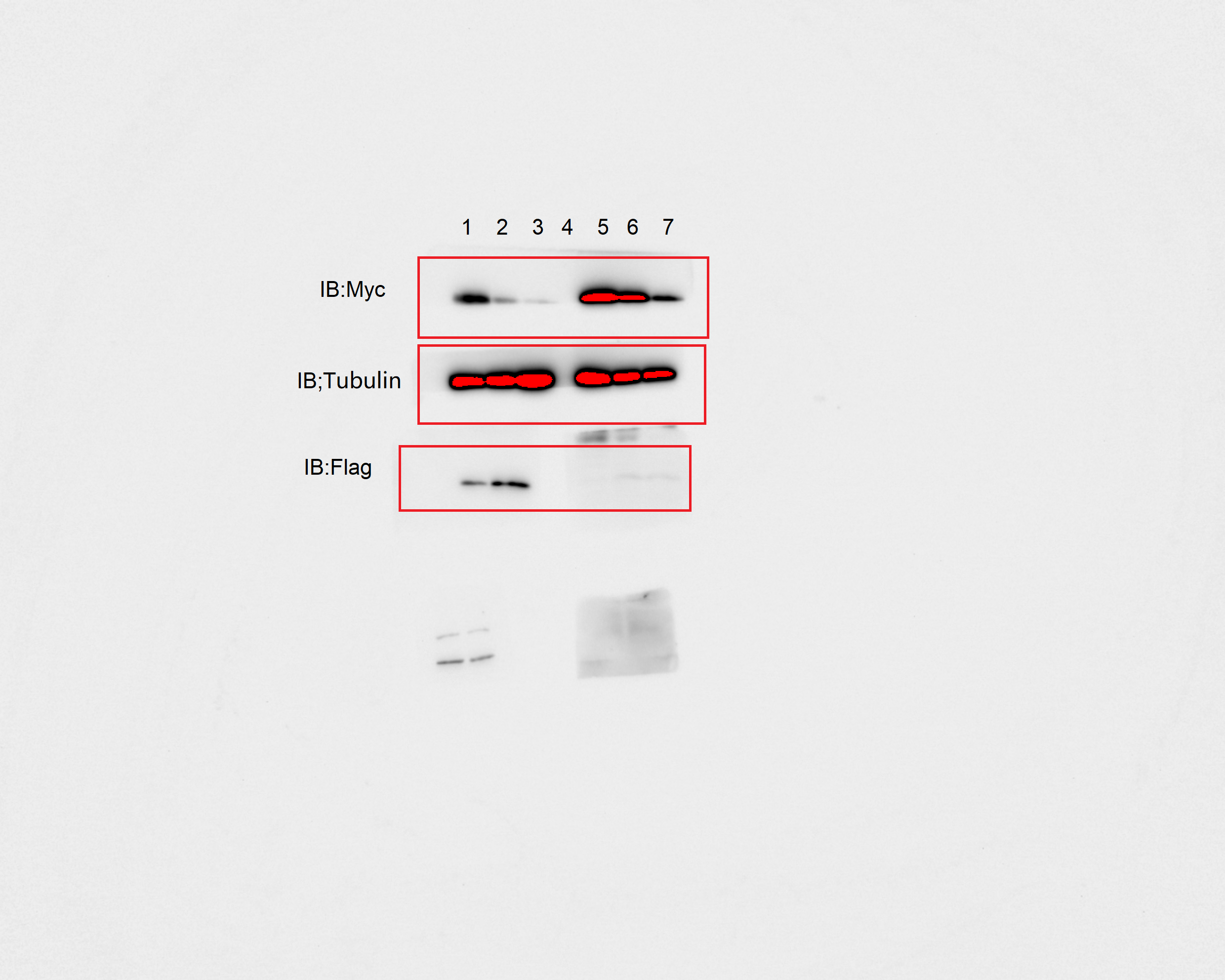

Supplement: Figure 2—figure supplement 1—source data 1. [file elife-101973-fig2-figsupp1-data1.zip › Figure 2–figure supplement 1-source data 1/Figure 2–figure supplement 1E 1F-labeled/long exposure of myc flag and tubulin.tif]

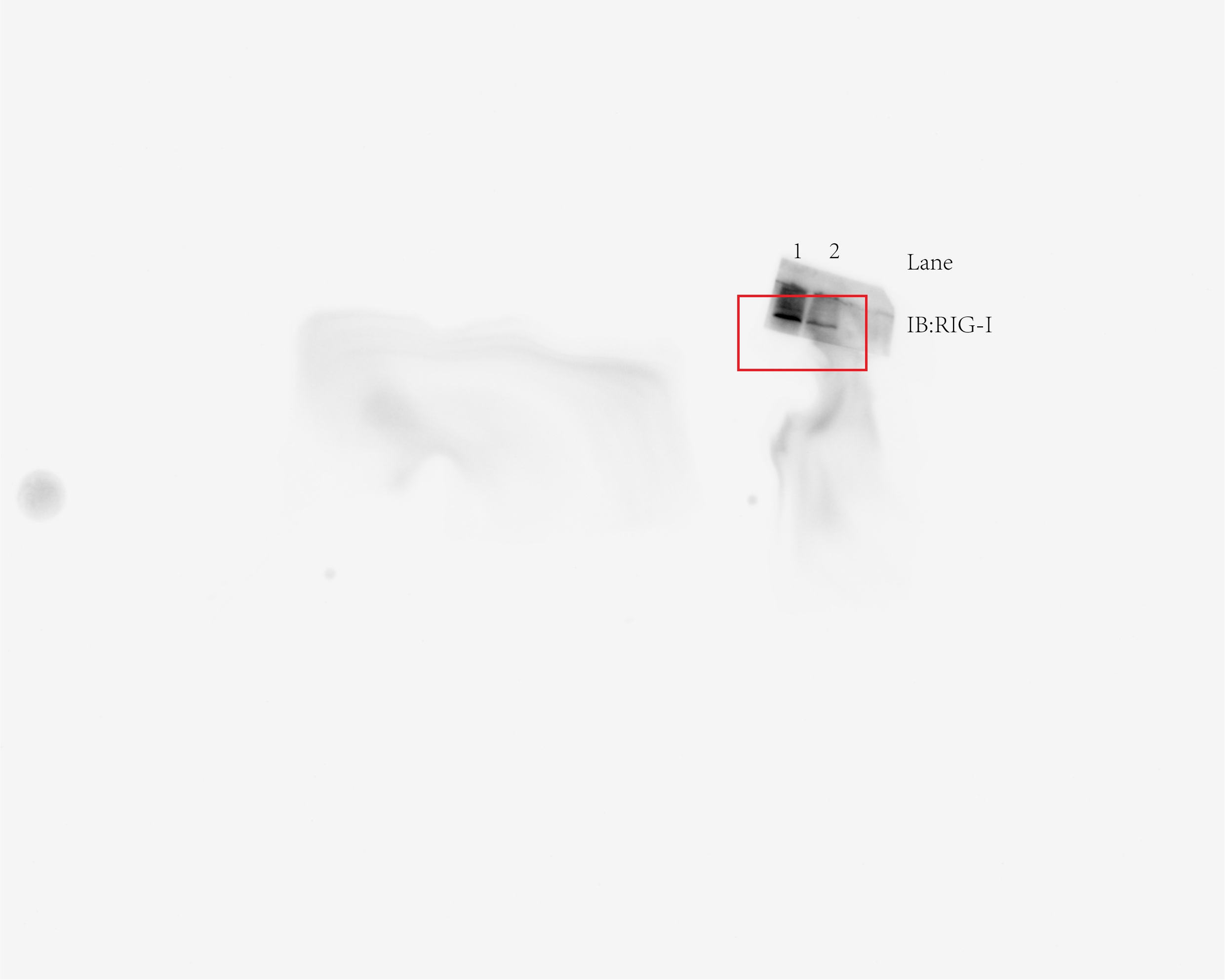

Supplement: Figure 2—figure supplement 1—source data 1. [file elife-101973-fig2-figsupp1-data1.zip › Figure 2–figure supplement 1-source data 1/Figure 2–figure supplement 1G-labeled/RIG-I.tif]

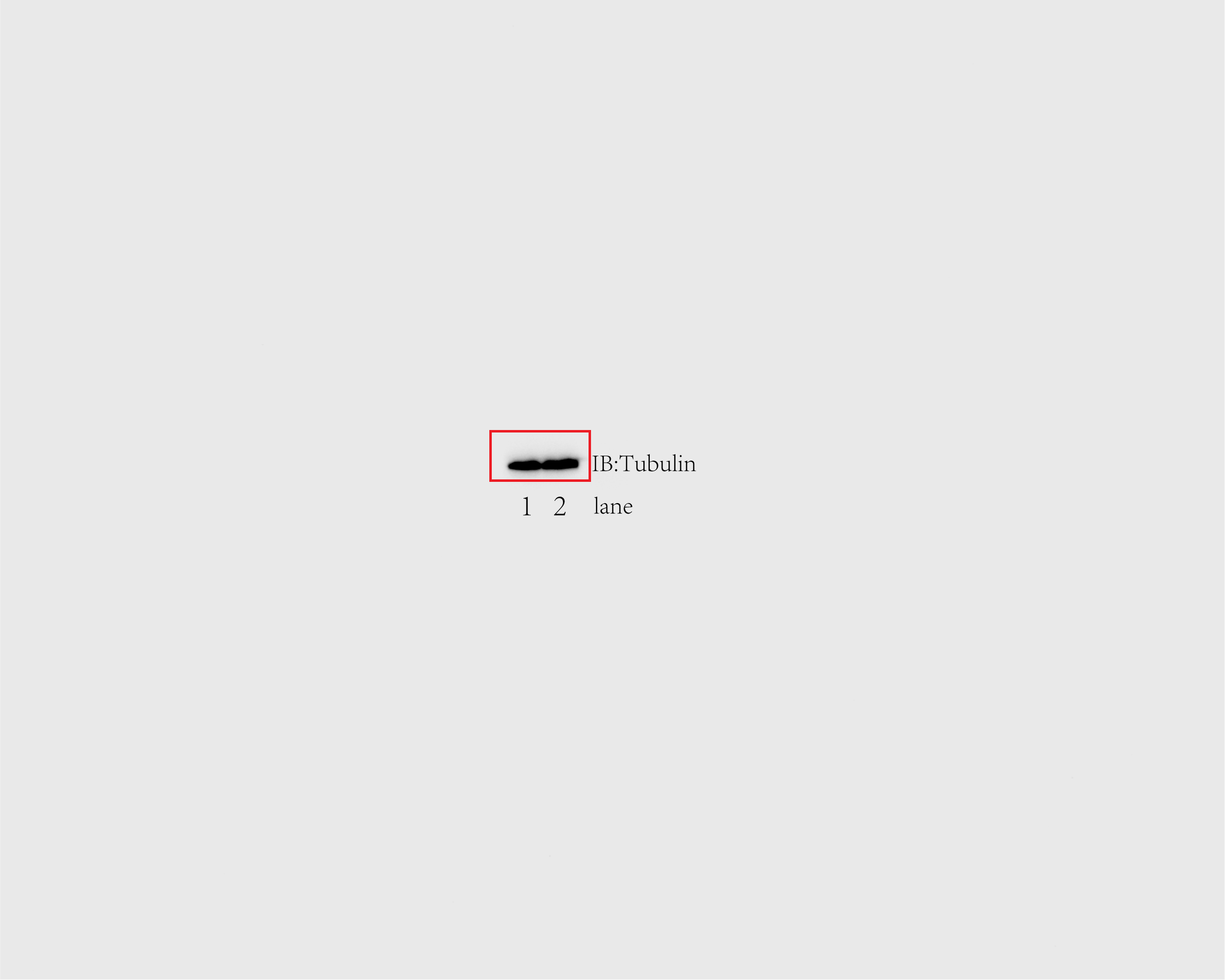

Supplement: Figure 2—figure supplement 1—source data 1. [file elife-101973-fig2-figsupp1-data1.zip › Figure 2–figure supplement 1-source data 1/Figure 2–figure supplement 1G-labeled/Tubulin.tif]

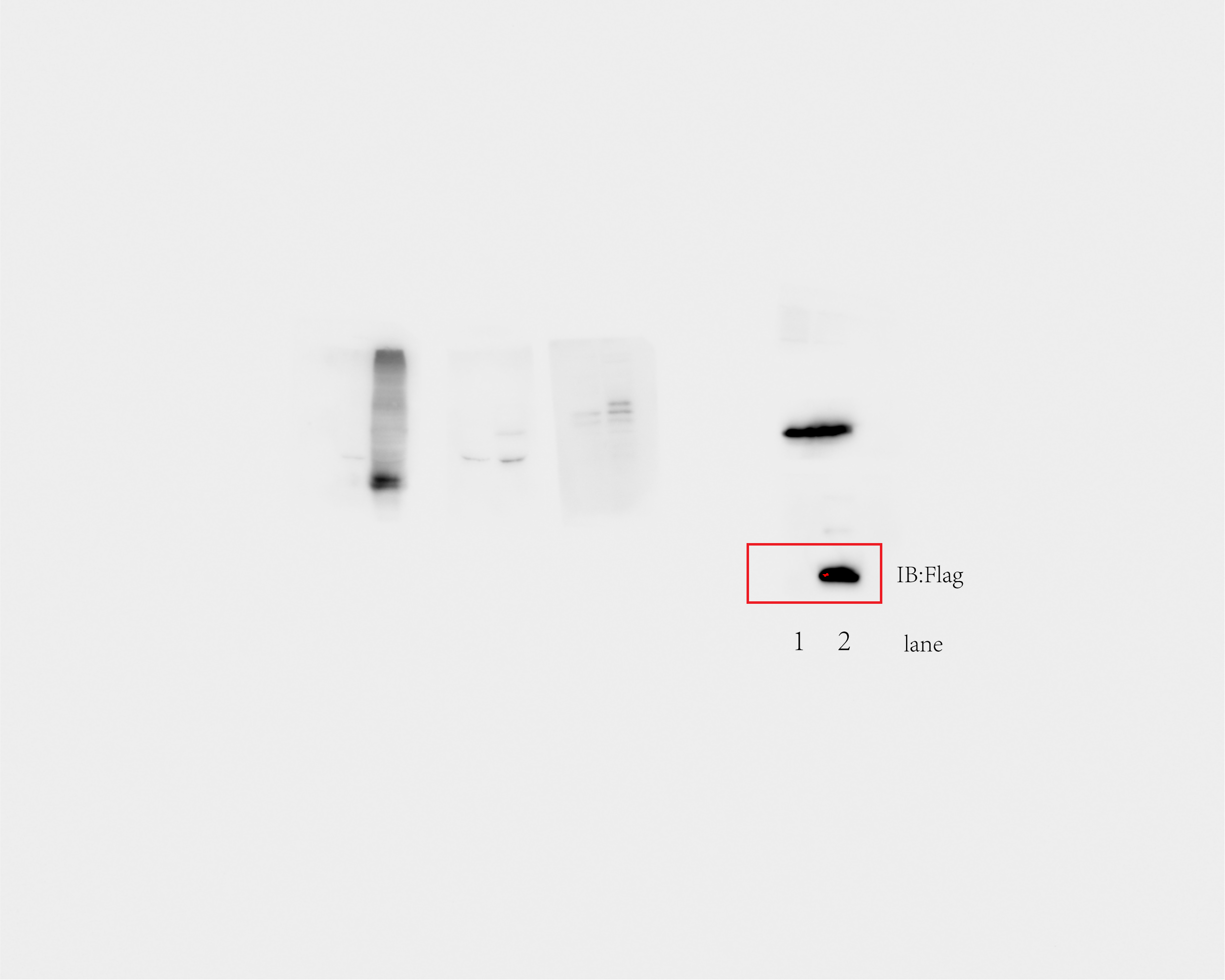

Supplement: Figure 2—figure supplement 1—source data 1. [file elife-101973-fig2-figsupp1-data1.zip › Figure 2–figure supplement 1-source data 1/Figure 2–figure supplement 1G-labeled/flag-ORMDL3.tif]

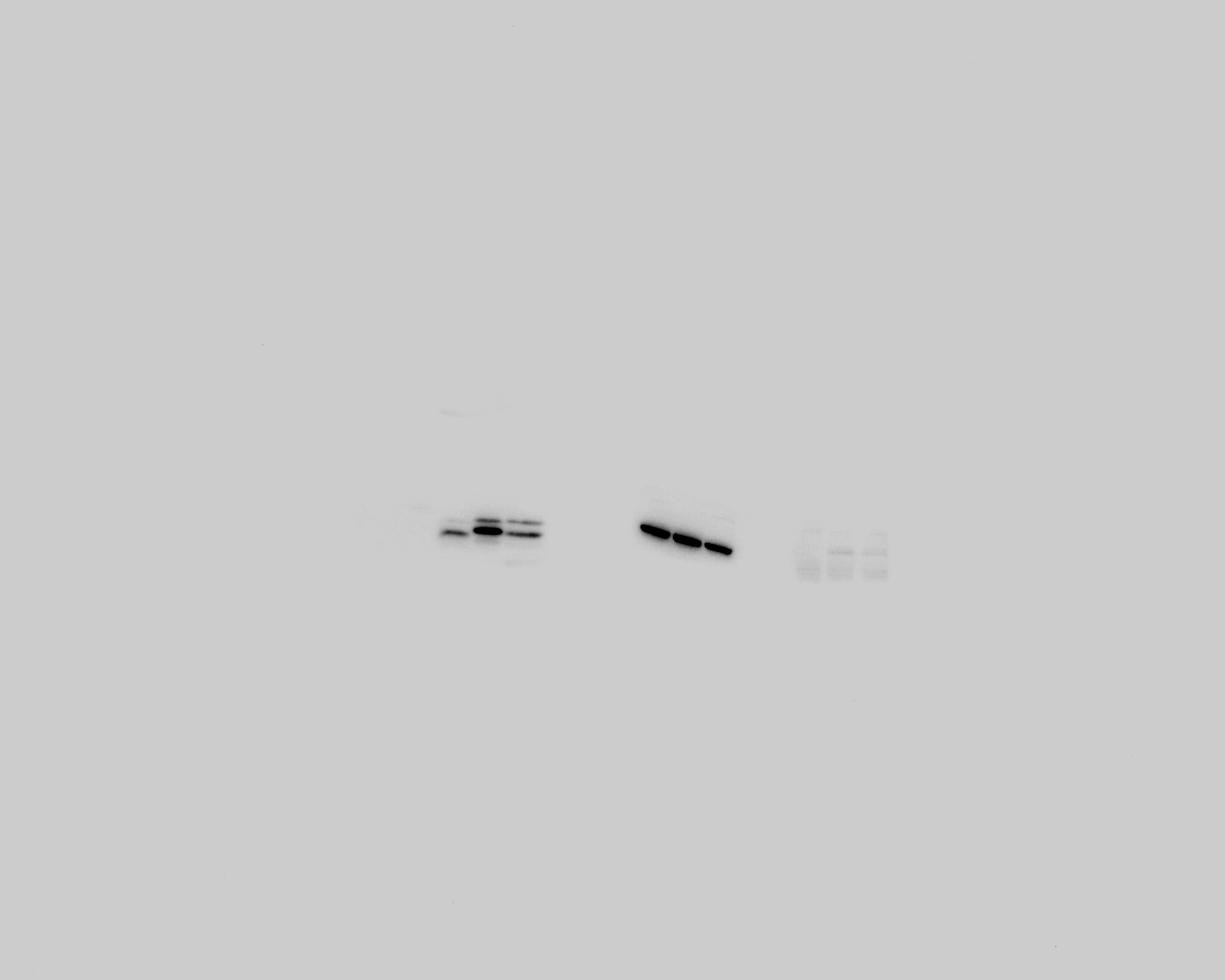

Supplement: Figure 2—figure supplement 1—source data 2. [file elife-101973-fig2-figsupp1-data2.zip › Figure 2-figure supplement 1-source data 2/Figure 2-figure supplement 1D/ORMDLs-Myc.tif]

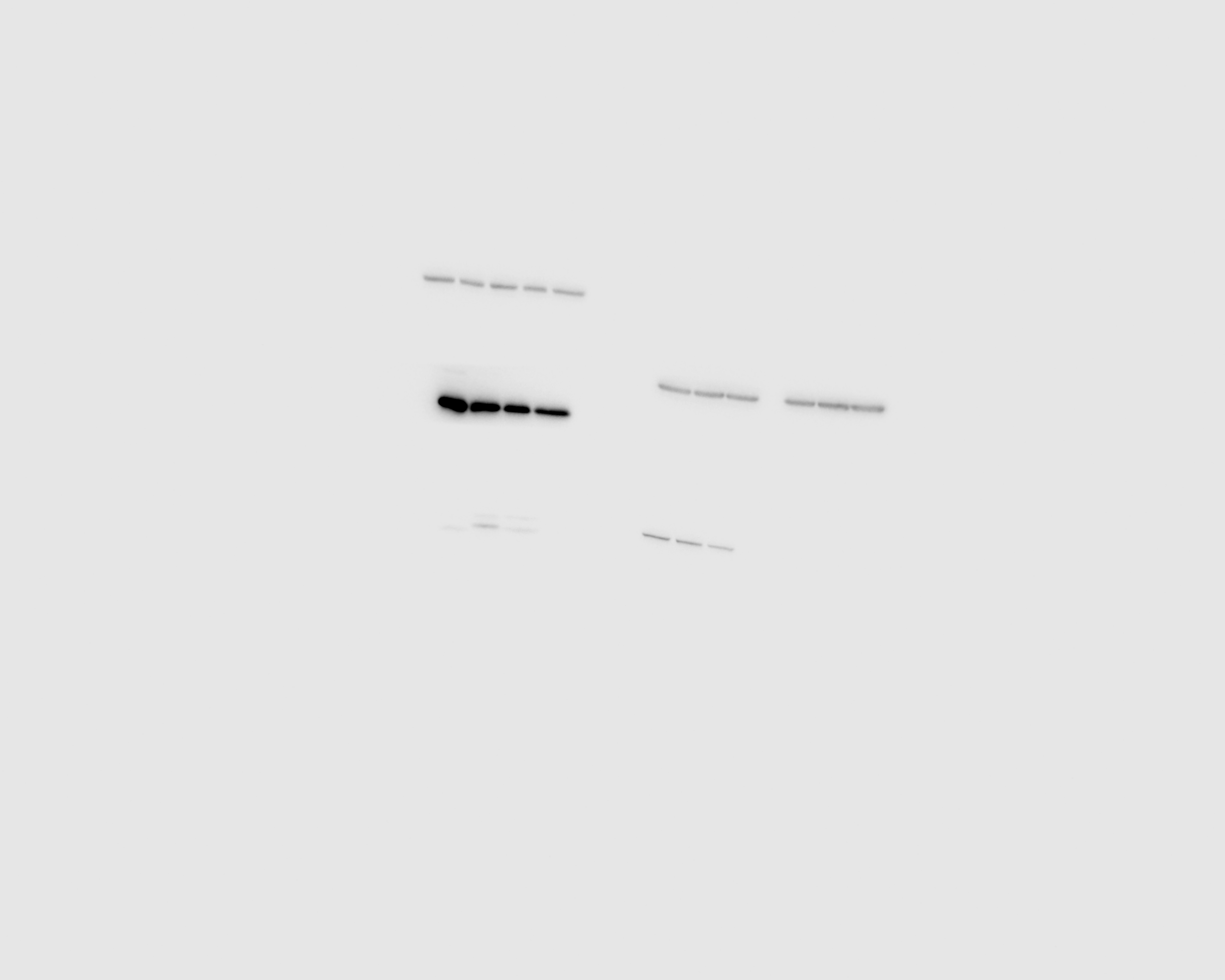

Supplement: Figure 2—figure supplement 1—source data 2. [file elife-101973-fig2-figsupp1-data2.zip › Figure 2-figure supplement 1-source data 2/Figure 2-figure supplement 1D/RIG-I-N-Myc.tif]

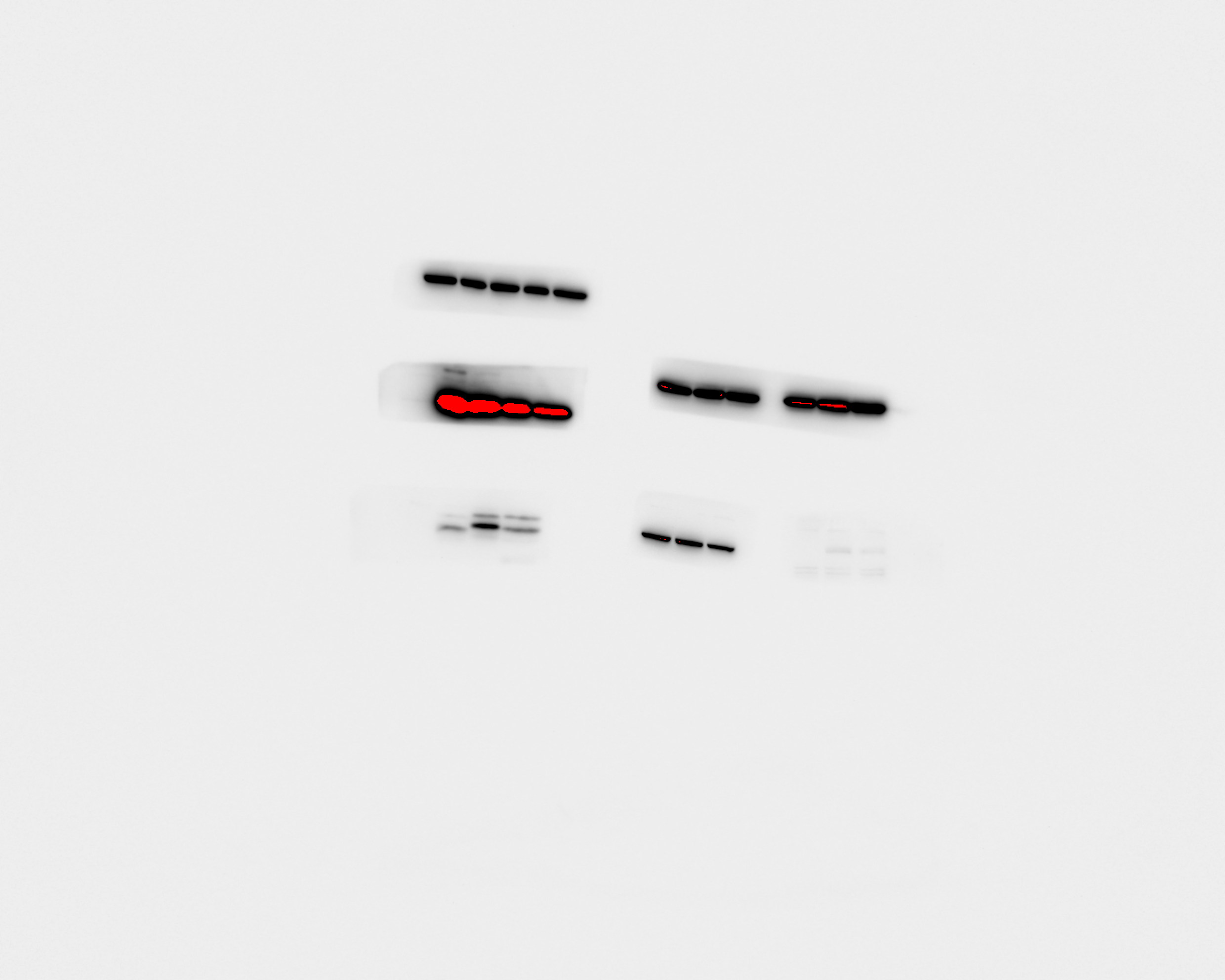

Supplement: Figure 2—figure supplement 1—source data 2. [file elife-101973-fig2-figsupp1-data2.zip › Figure 2-figure supplement 1-source data 2/Figure 2-figure supplement 1D/tubulin.tif]

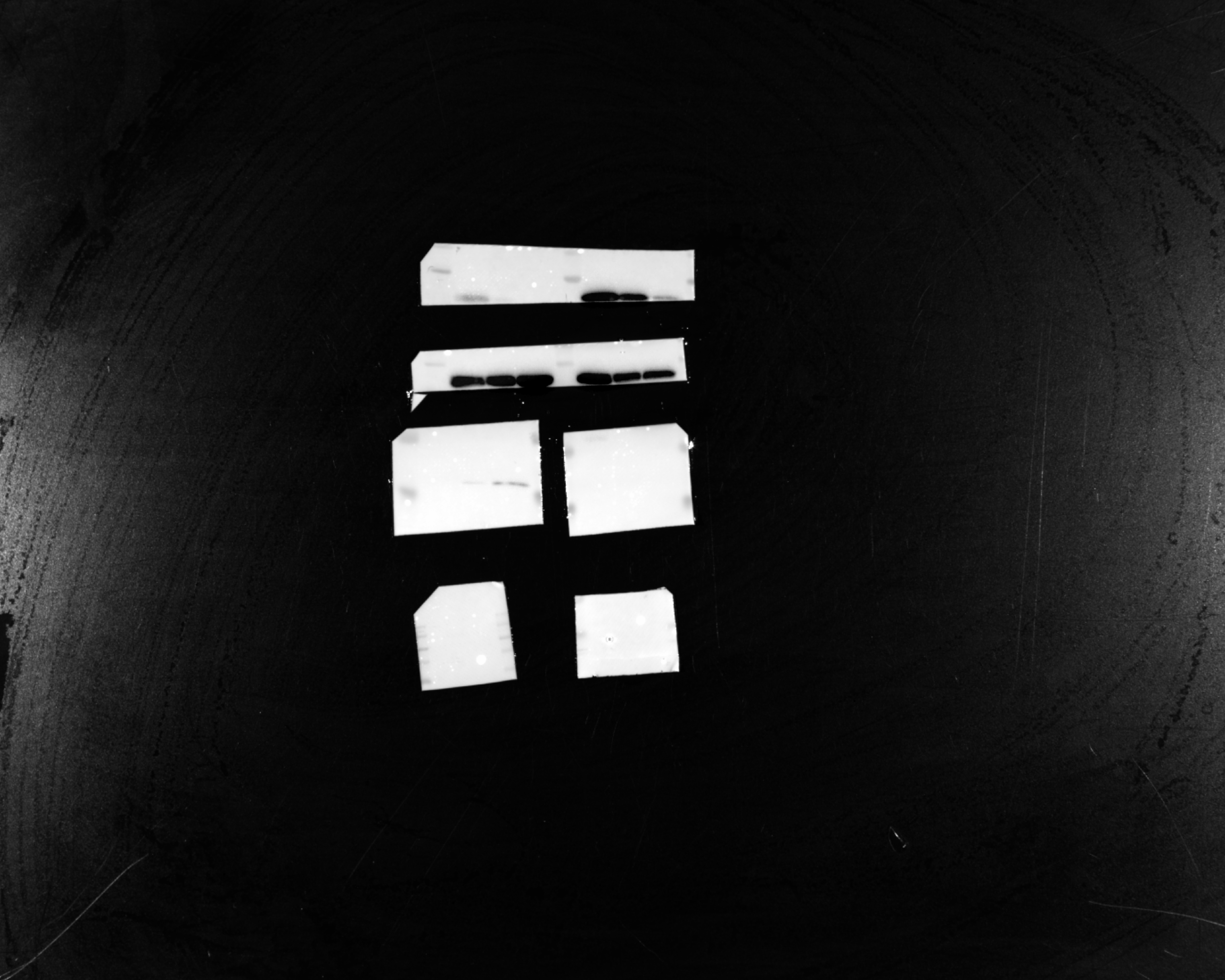

Supplement: Figure 2—figure supplement 1—source data 2. [file elife-101973-fig2-figsupp1-data2.zip › Figure 2-figure supplement 1-source data 2/Figure 2-figure supplement 1E 1F/Myc flag and tubulin.jpg]

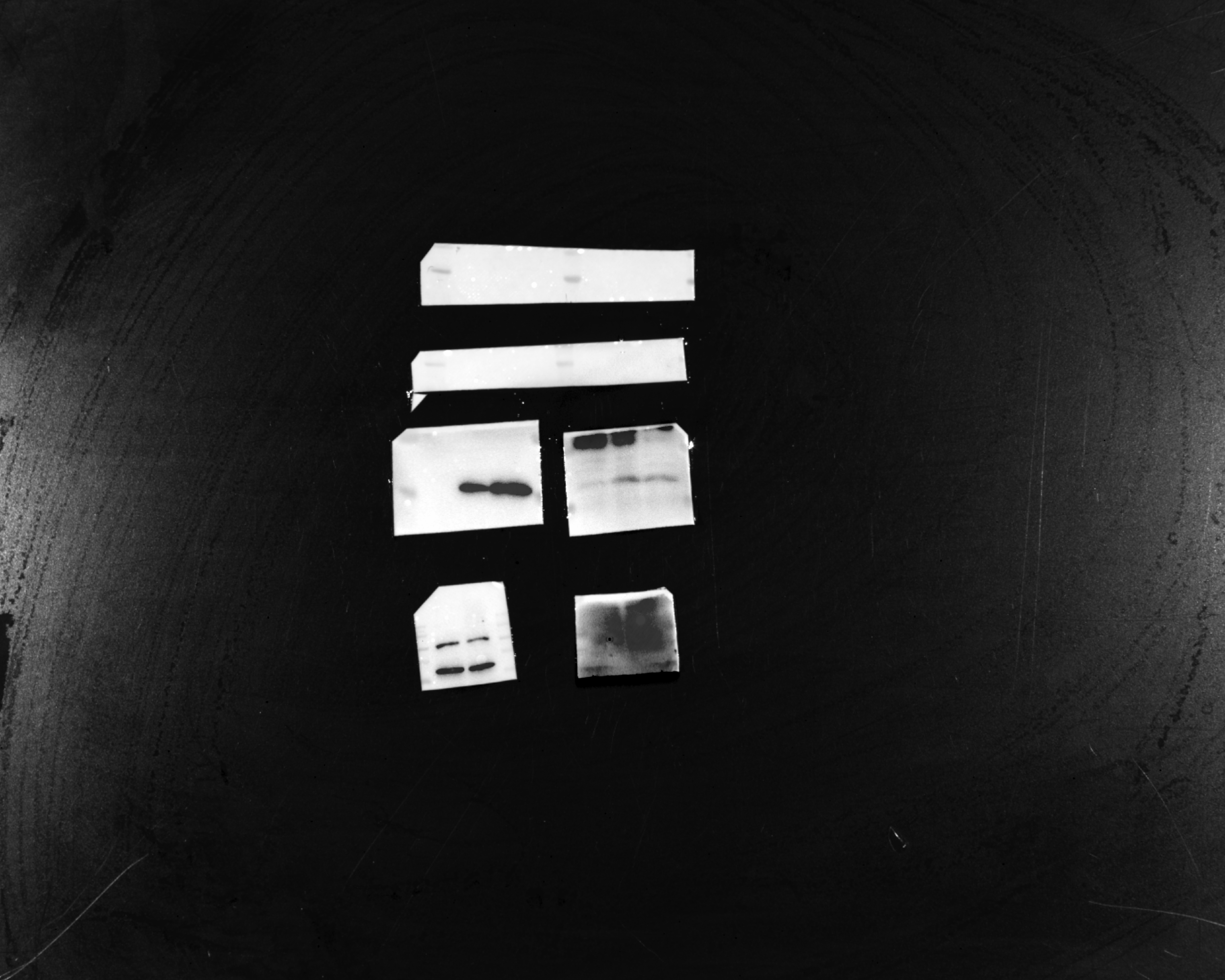

Supplement: Figure 2—figure supplement 1—source data 2. [file elife-101973-fig2-figsupp1-data2.zip › Figure 2-figure supplement 1-source data 2/Figure 2-figure supplement 1E 1F/longer exposure of ORMDL3-Myc.jpg]

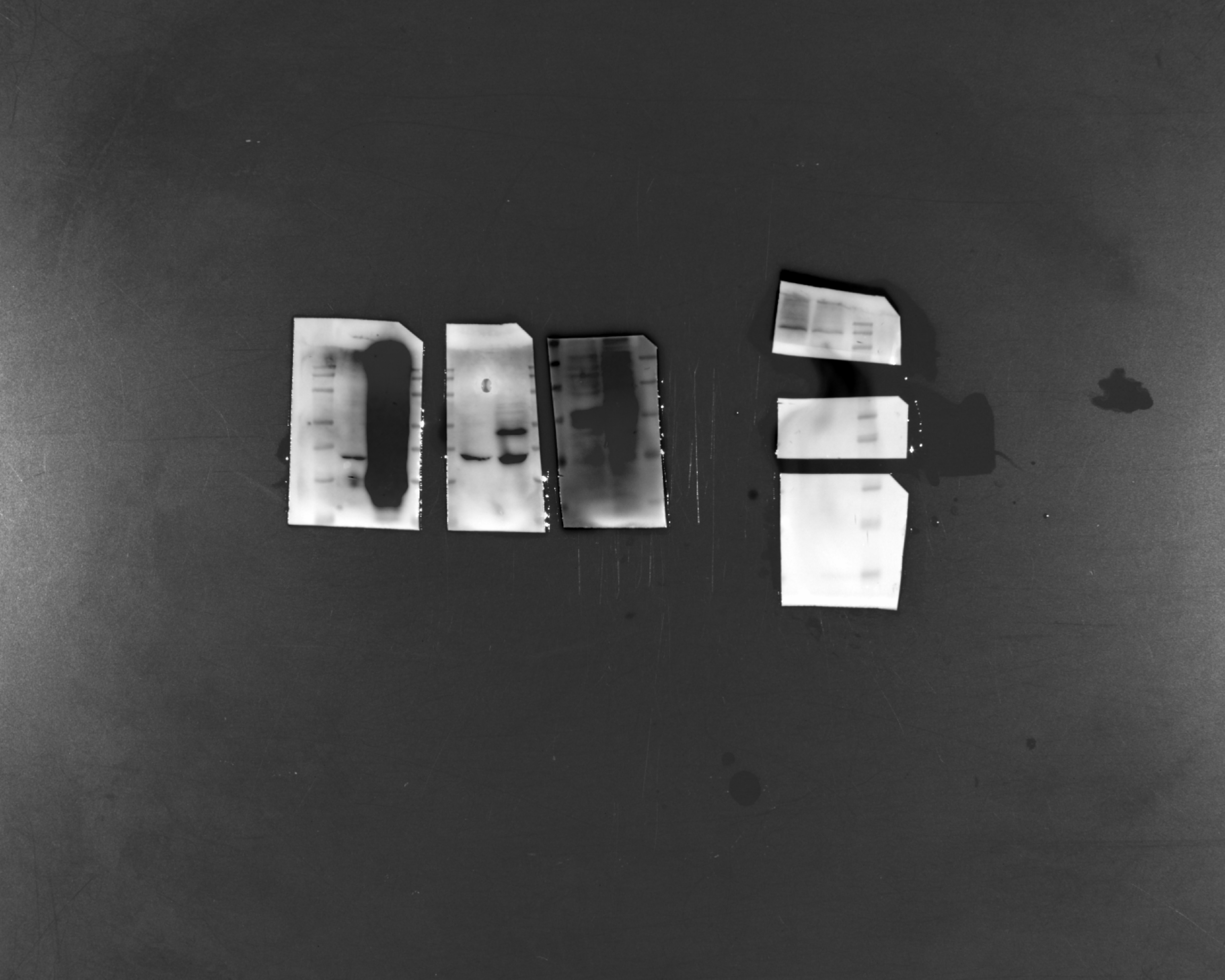

Supplement: Figure 2—figure supplement 1—source data 2. [file elife-101973-fig2-figsupp1-data2.zip › Figure 2-figure supplement 1-source data 2/Figure 2-figure supplement 1G/RIG-I.jpg]

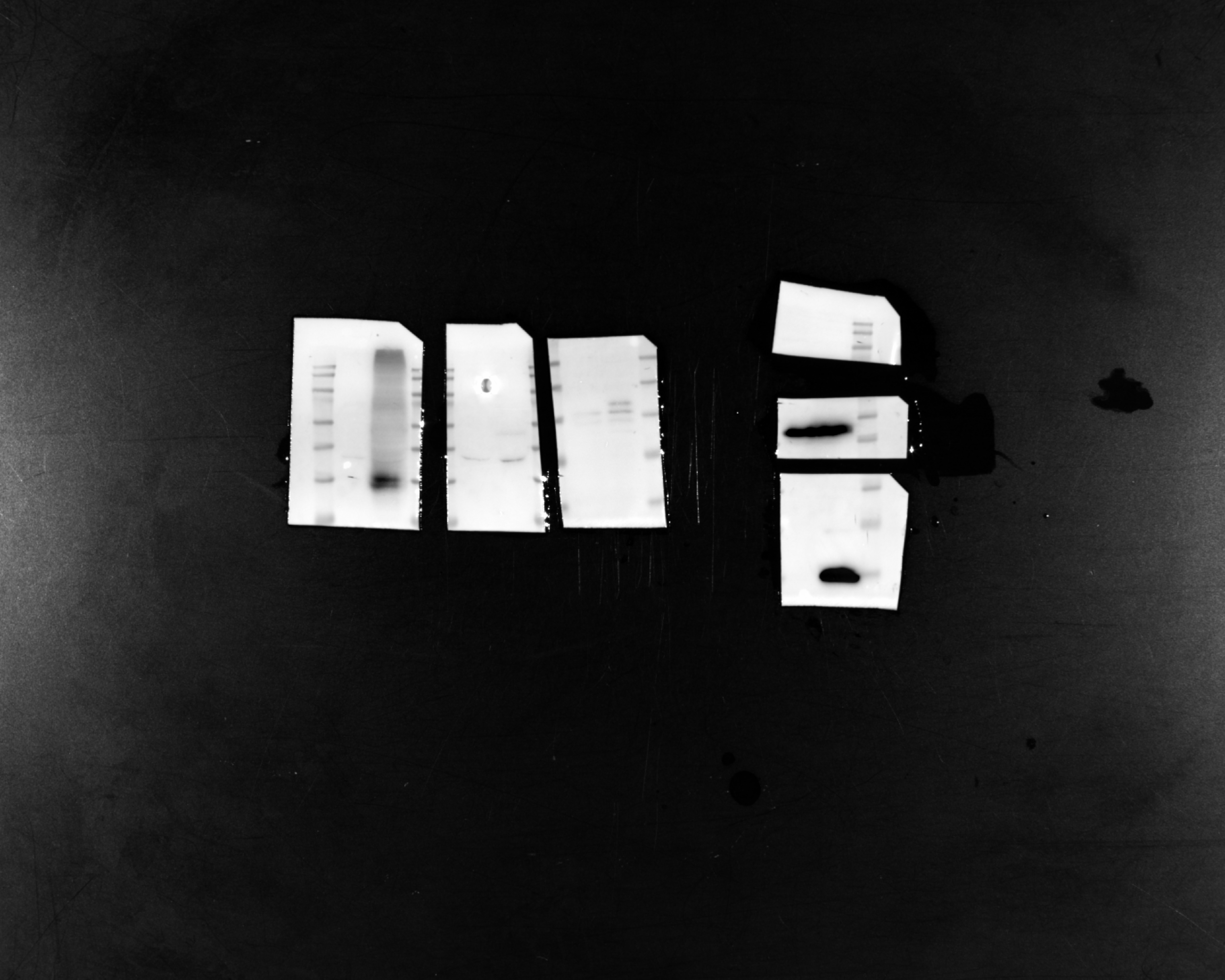

Supplement: Figure 2—figure supplement 1—source data 2. [file elife-101973-fig2-figsupp1-data2.zip › Figure 2-figure supplement 1-source data 2/Figure 2-figure supplement 1G/flag-ORMDL3.jpg]

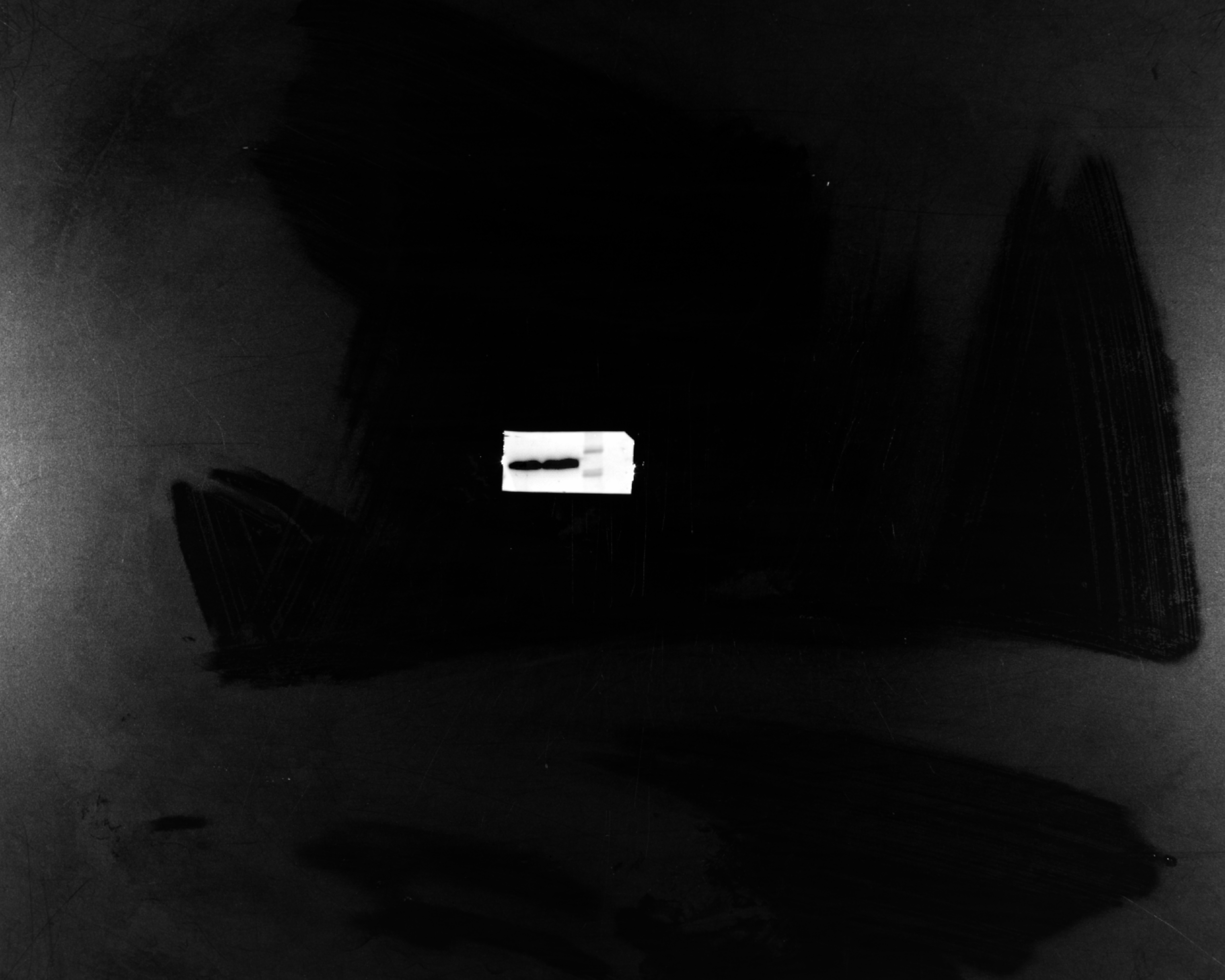

Supplement: Figure 2—figure supplement 1—source data 2. [file elife-101973-fig2-figsupp1-data2.zip › Figure 2-figure supplement 1-source data 2/Figure 2-figure supplement 1G/tubulin.jpg]

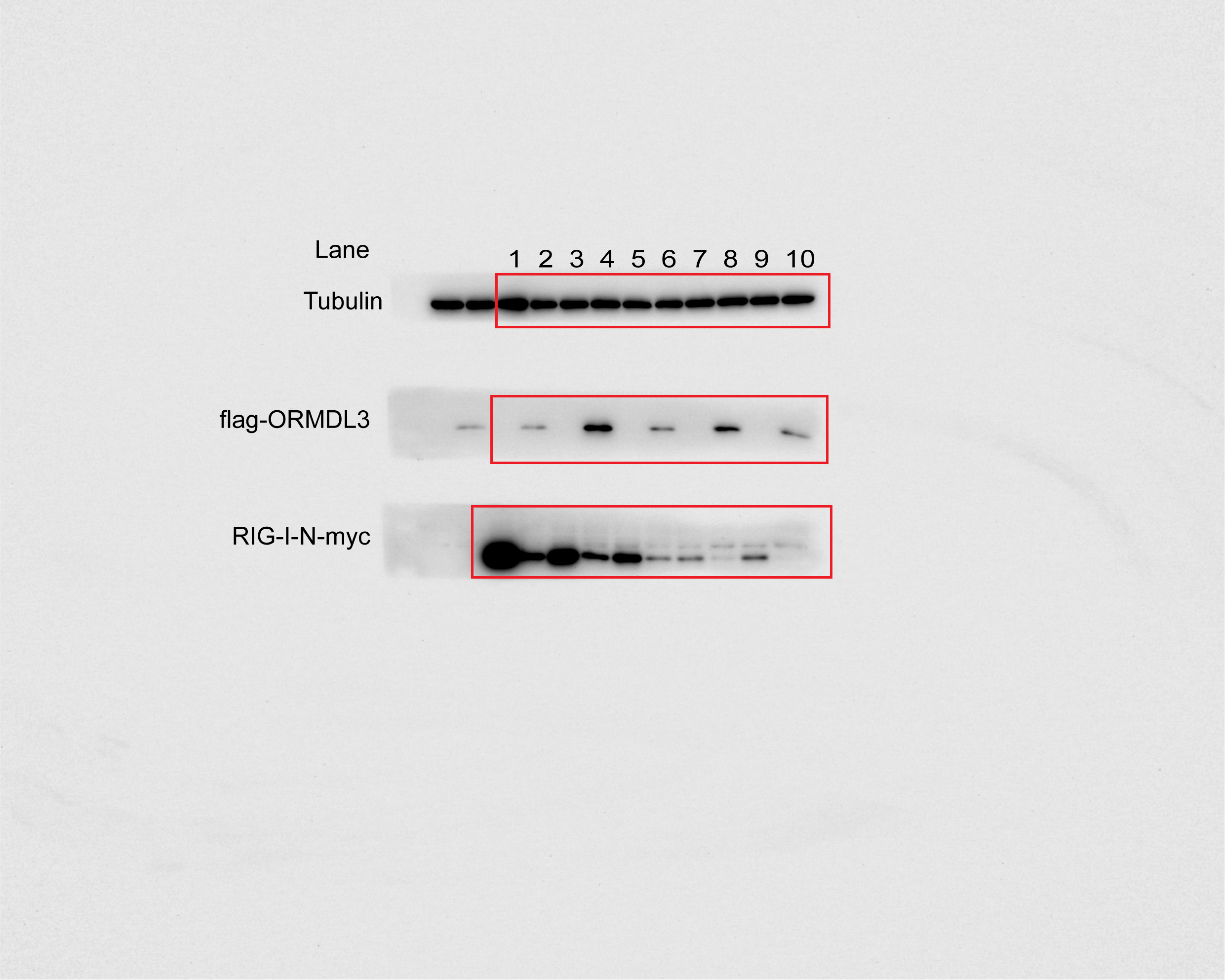

Supplement: Figure 3—source data 1. [file elife-101973-fig3-data1.zip › Figure 3-source data1/Fig3E-labeled/MYC FLAG and tubulin.tif]

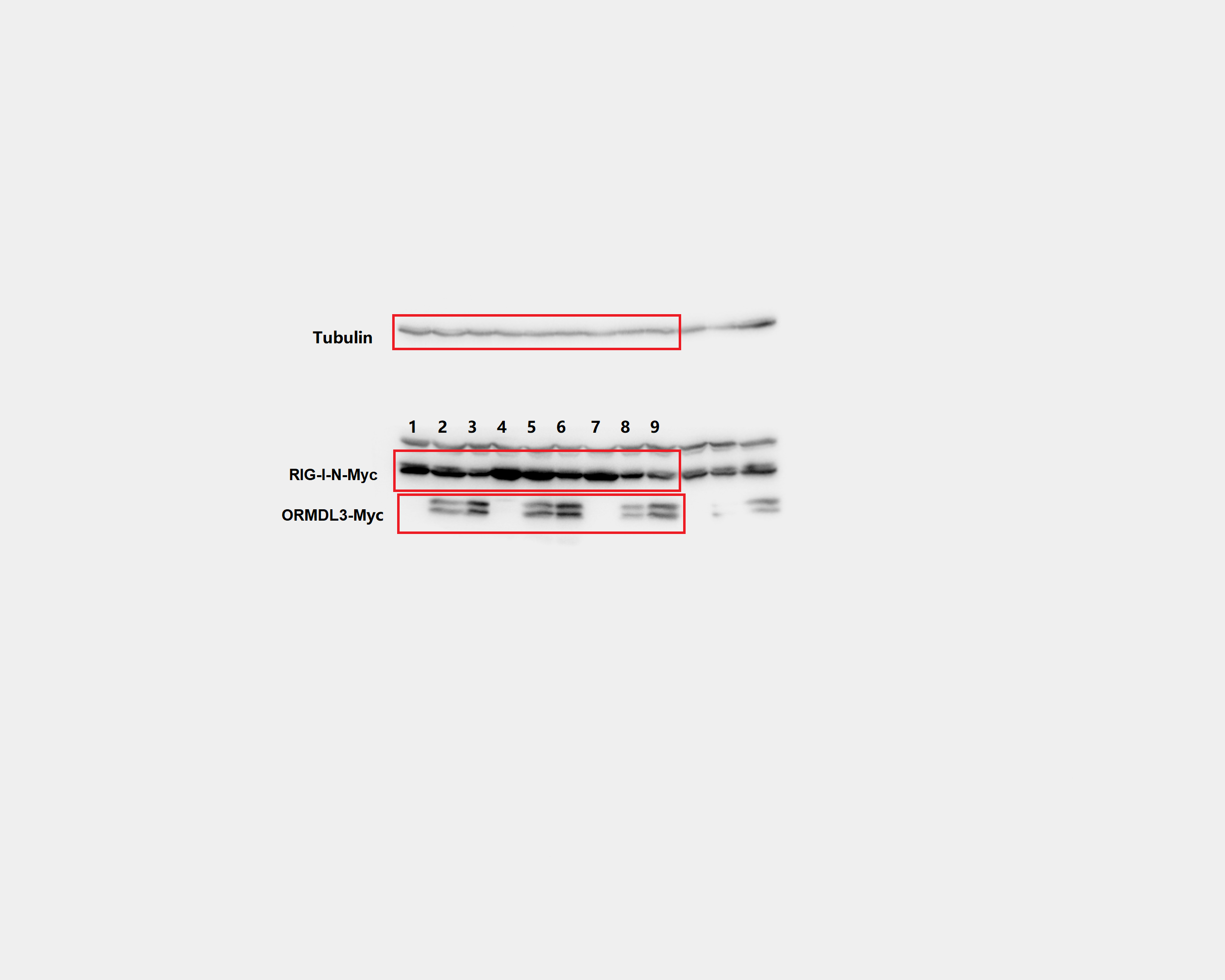

Supplement: Figure 3—source data 1. [file elife-101973-fig3-data1.zip › Figure 3-source data1/Fig3A-labeled/RIG-I-N-Myc,ORMDL3-Myc and tubulin .tif]

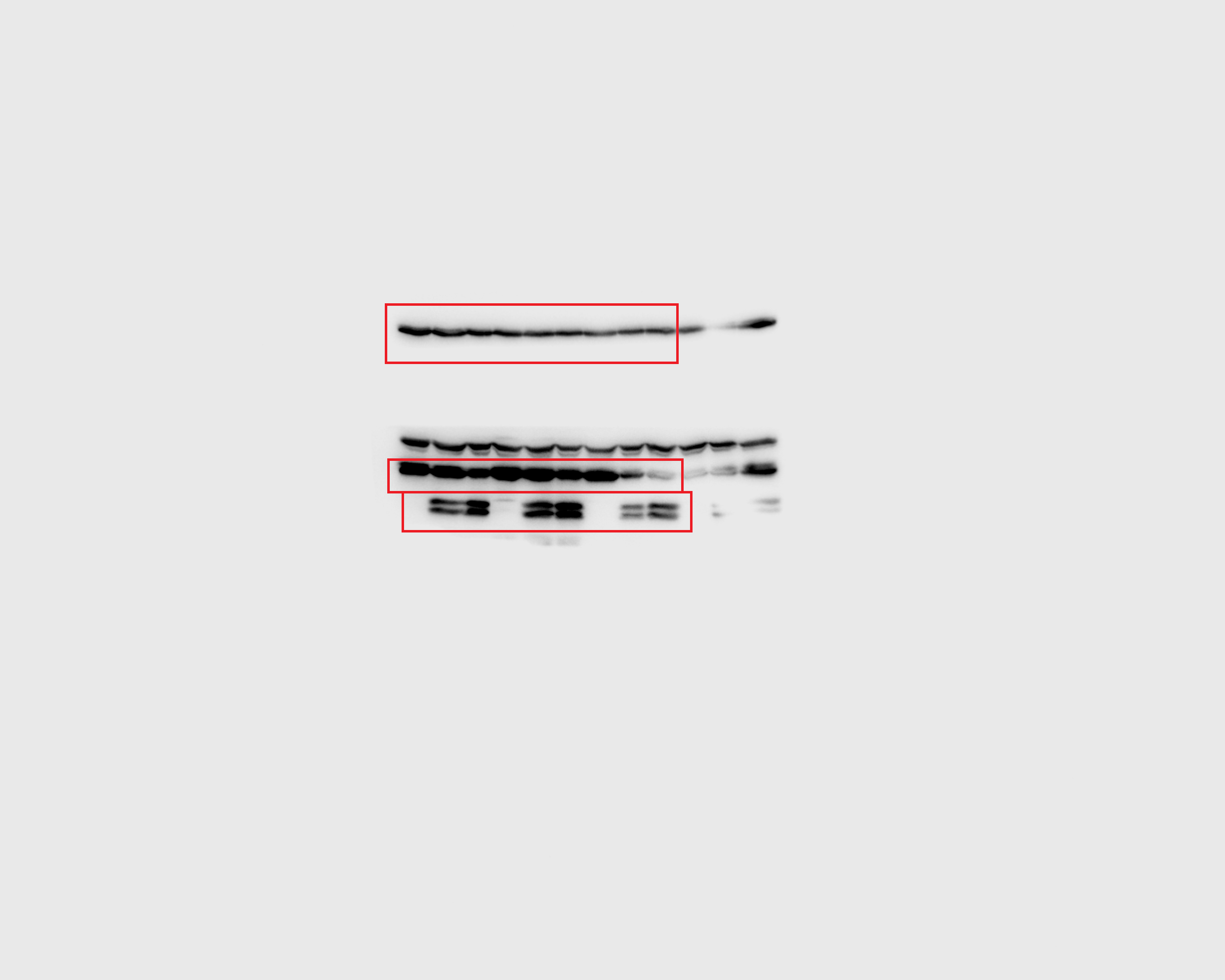

Supplement: Figure 3—source data 1. [file elife-101973-fig3-data1.zip › Figure 3-source data1/Fig3A-labeled/long exposure of RIG-I-N-Myc,ORMDL3-Myc and tubulin .tif]

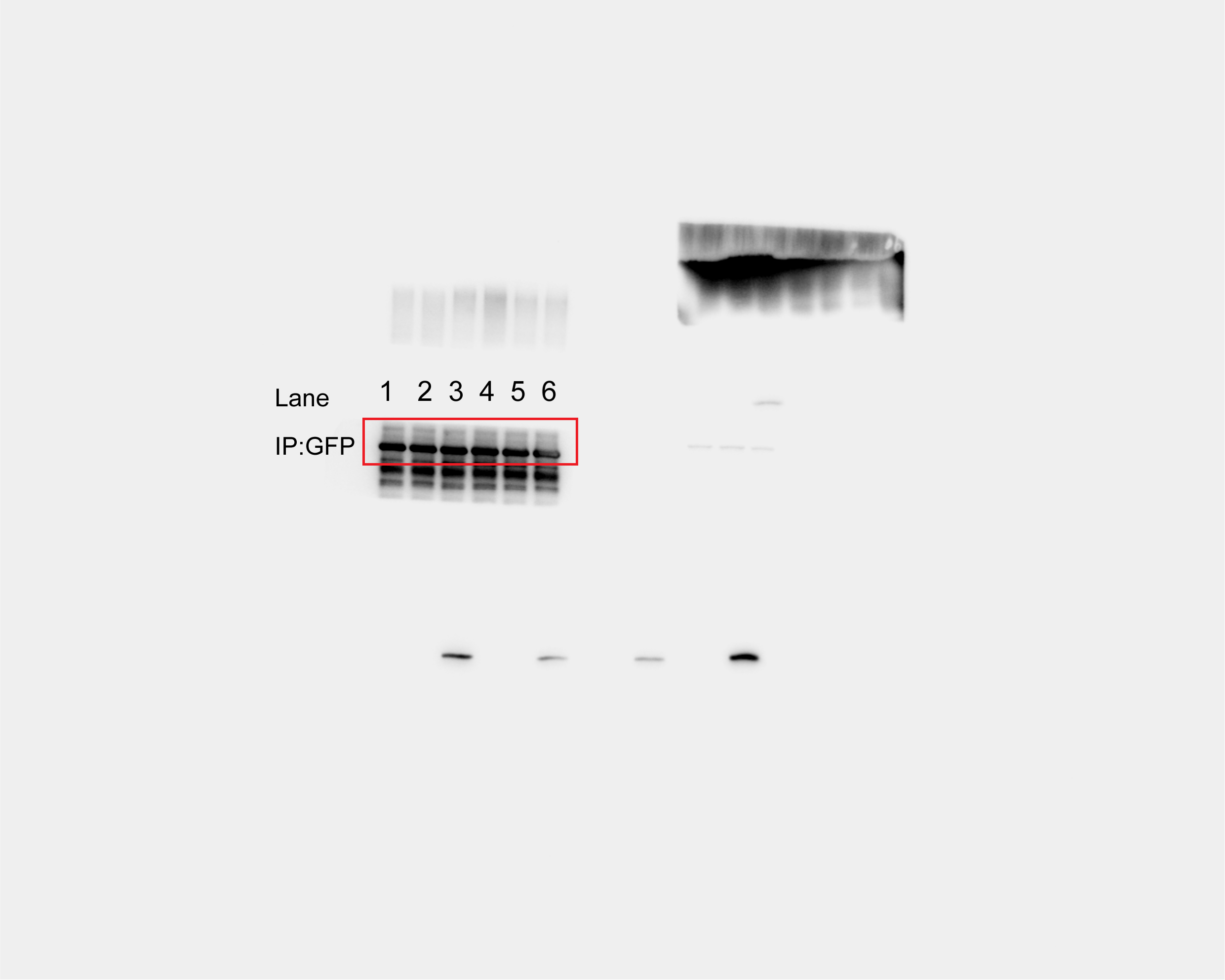

Supplement: Figure 3—source data 1. [file elife-101973-fig3-data1.zip › Figure 3-source data1/Fig3B-labeled/IPGFP.tif]

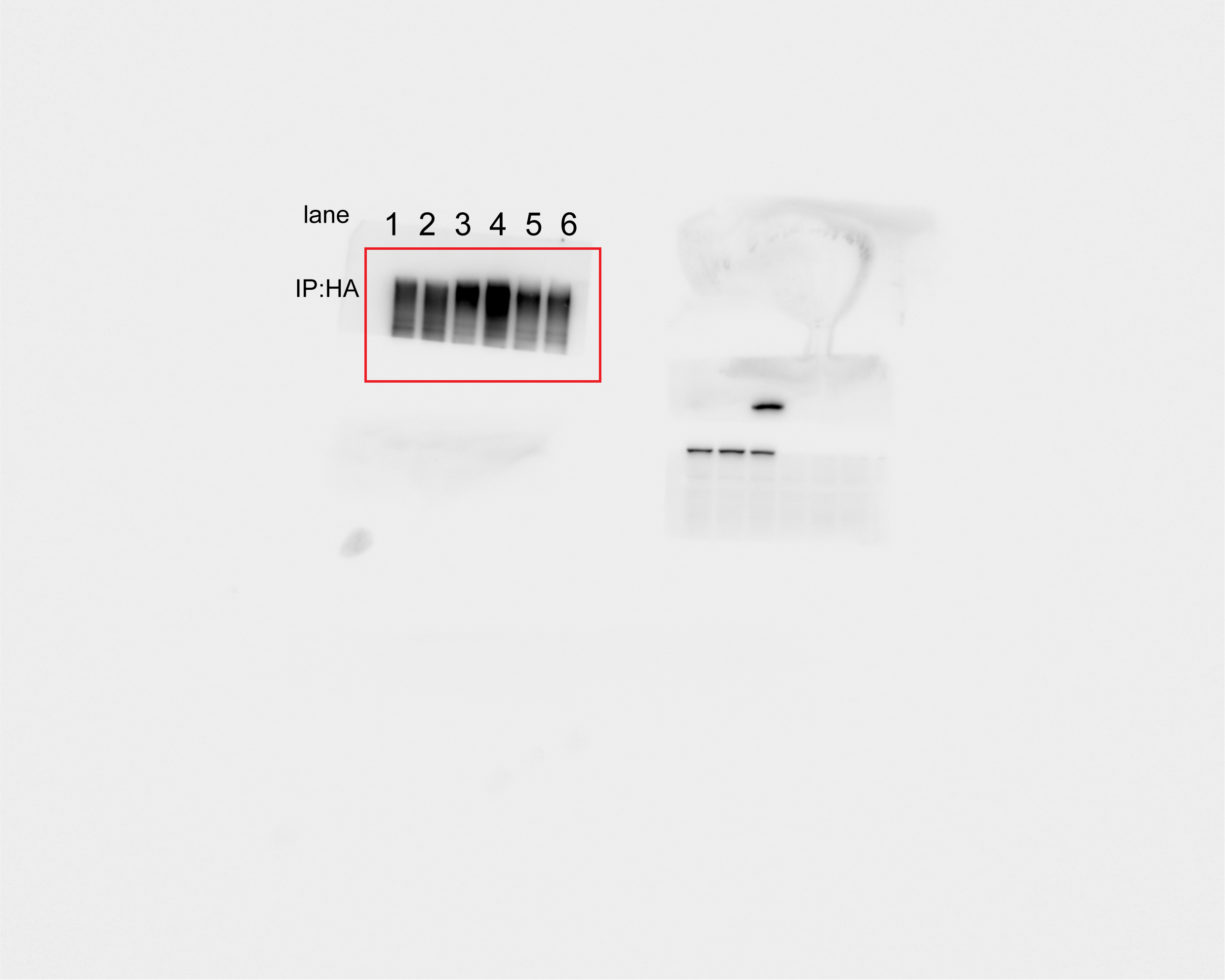

Supplement: Figure 3—source data 1. [file elife-101973-fig3-data1.zip › Figure 3-source data1/Fig3B-labeled/IPHA.tif]

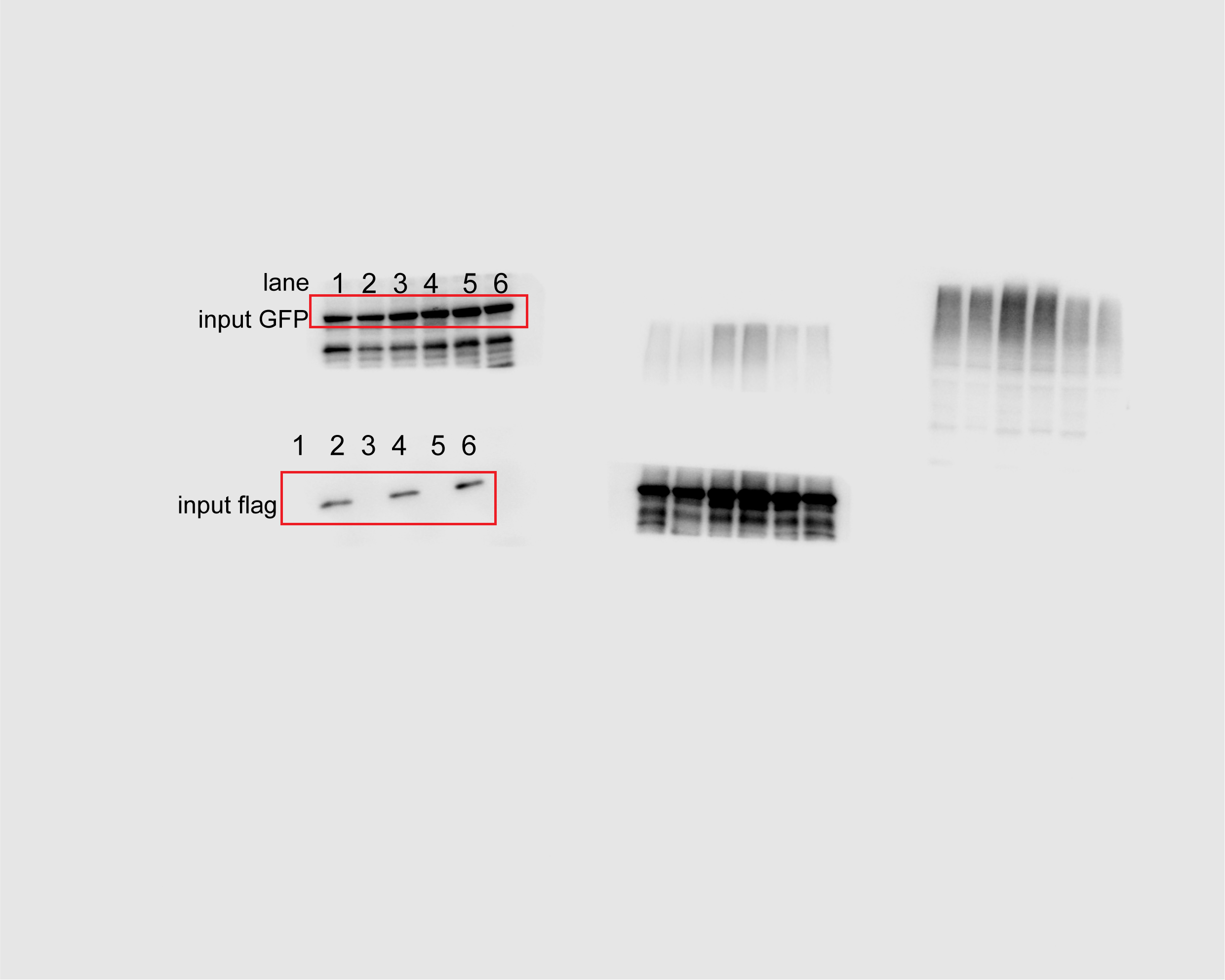

Supplement: Figure 3—source data 1. [file elife-101973-fig3-data1.zip › Figure 3-source data1/Fig3B-labeled/input GFP FLAG.tif]

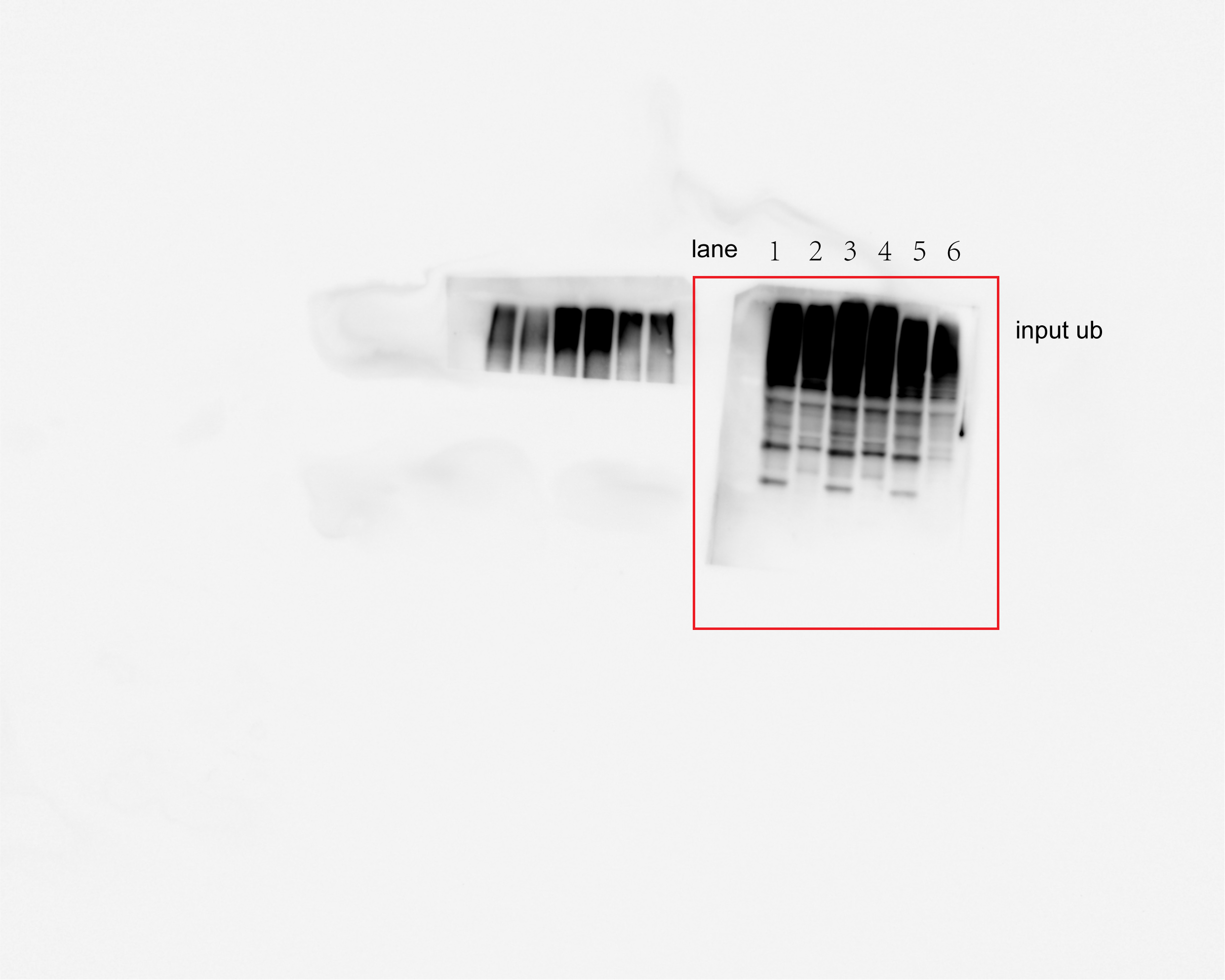

Supplement: Figure 3—source data 1. [file elife-101973-fig3-data1.zip › Figure 3-source data1/Fig3B-labeled/input HA .tif]

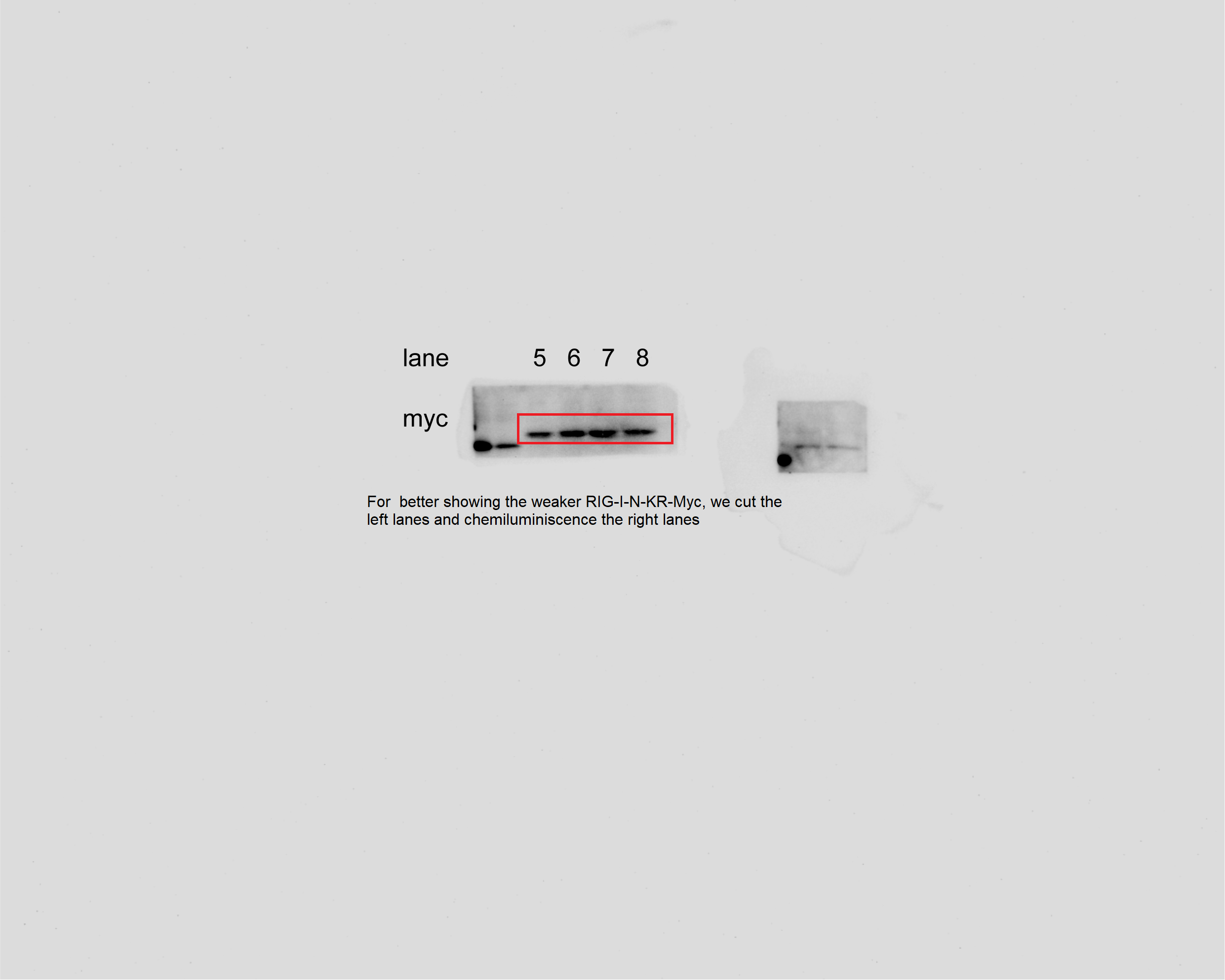

Supplement: Figure 3—source data 1. [file elife-101973-fig3-data1.zip › Figure 3-source data1/Fig3C-labeled/RIG-I-N-MYC long exposure.tif]

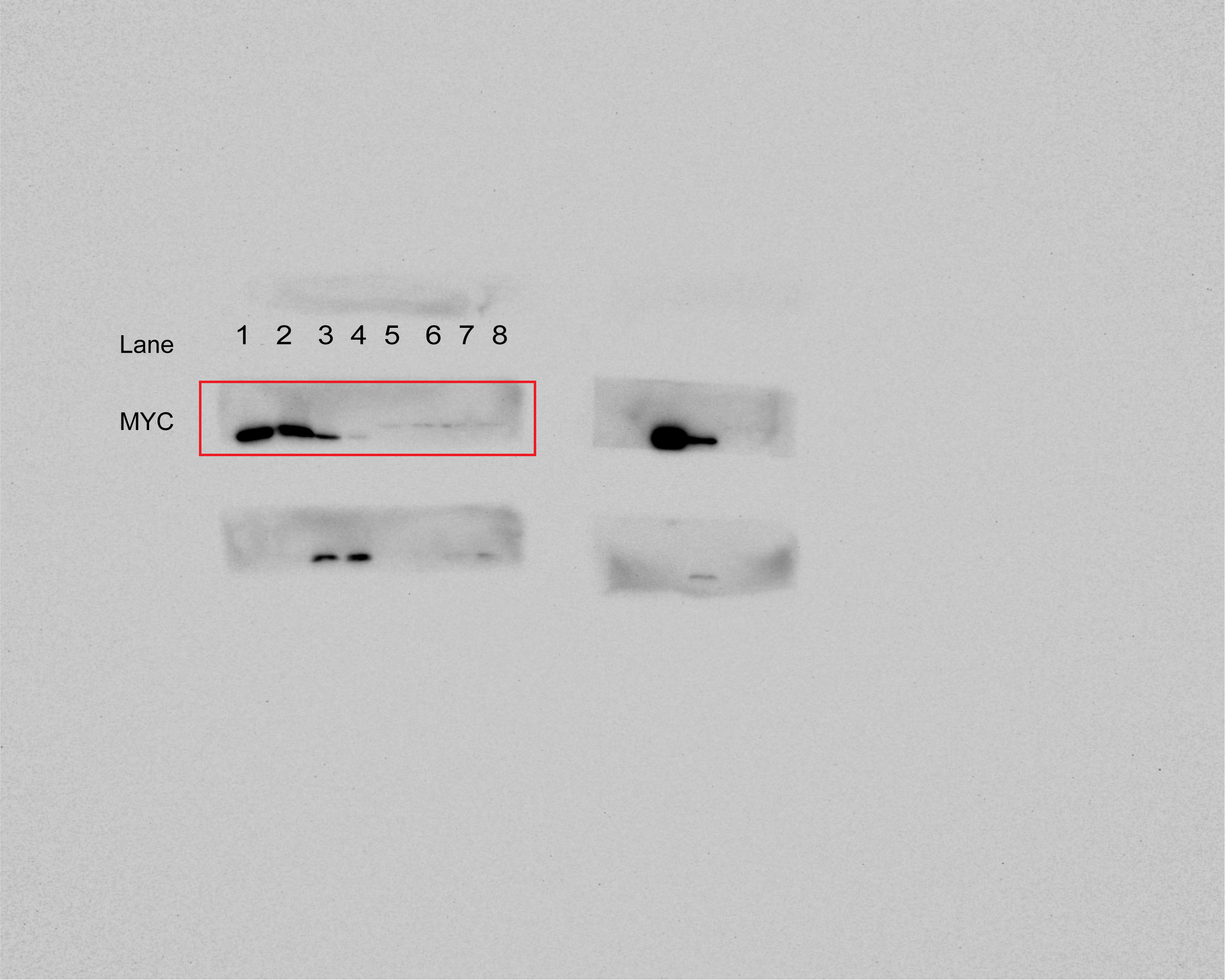

Supplement: Figure 3—source data 1. [file elife-101973-fig3-data1.zip › Figure 3-source data1/Fig3C-labeled/RIG-I-N-MYC short exposure.tif]

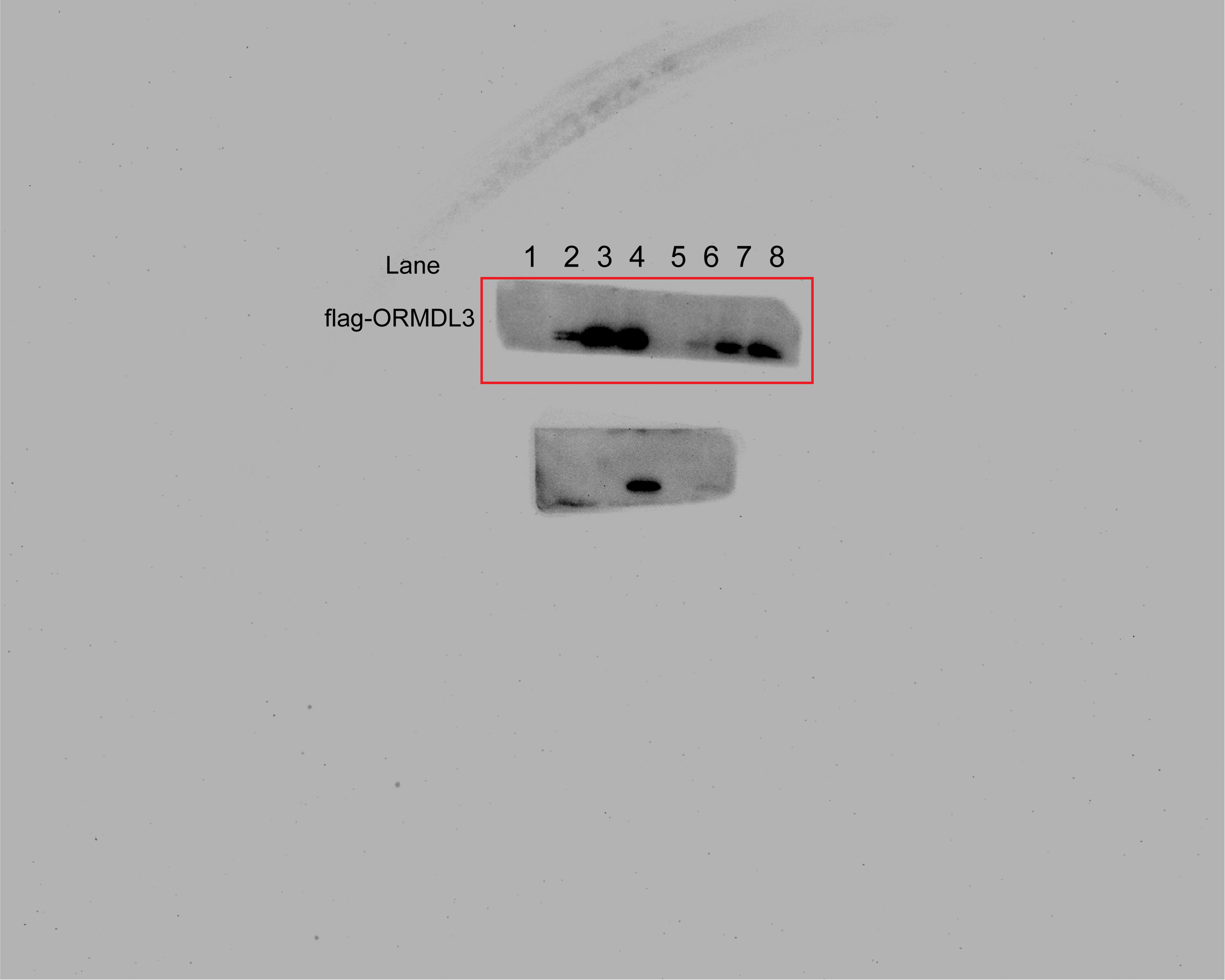

Supplement: Figure 3—source data 1. [file elife-101973-fig3-data1.zip › Figure 3-source data1/Fig3C-labeled/flag-ORMDL3.tif]

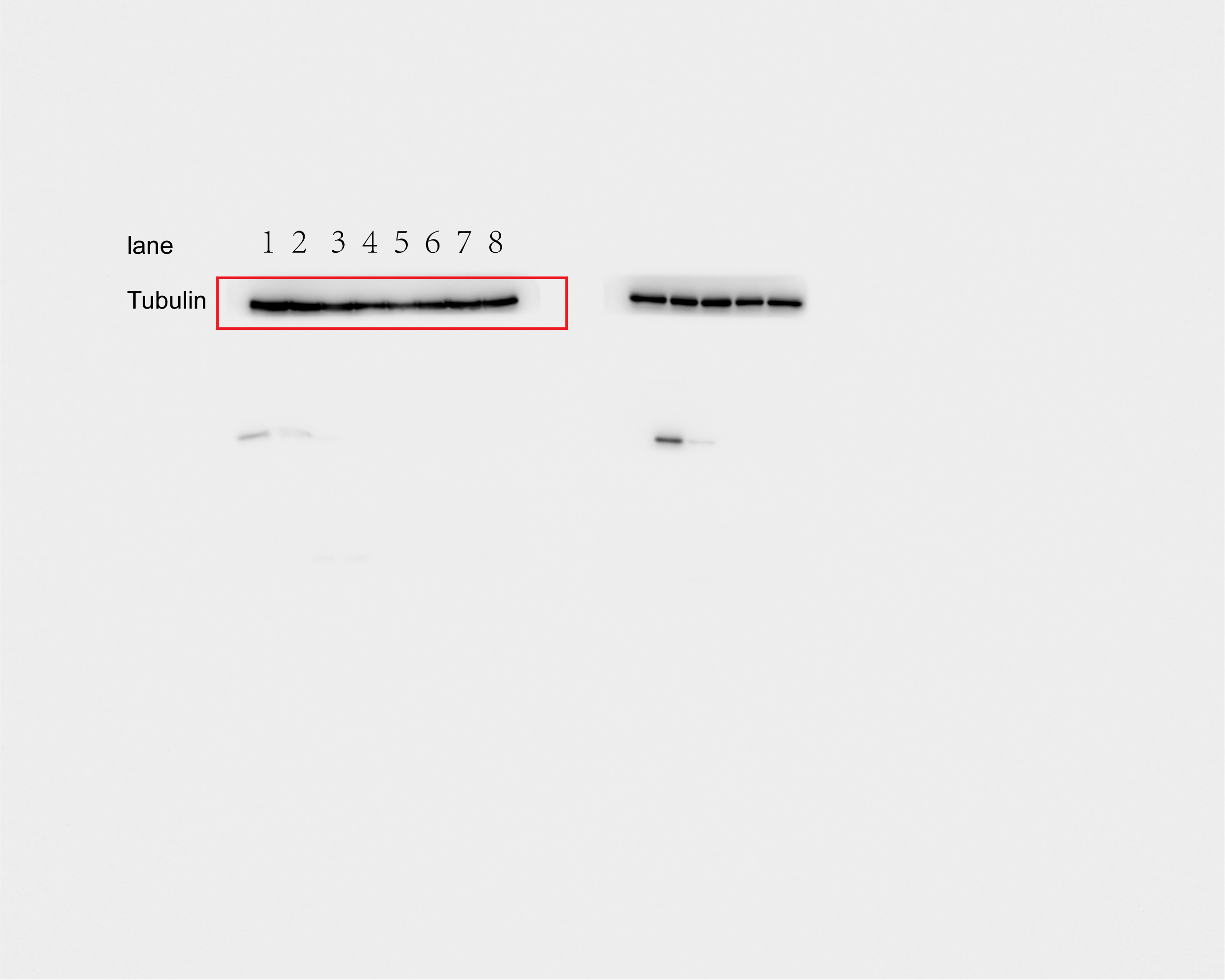

Supplement: Figure 3—source data 1. [file elife-101973-fig3-data1.zip › Figure 3-source data1/Fig3C-labeled/tubulin.tif]

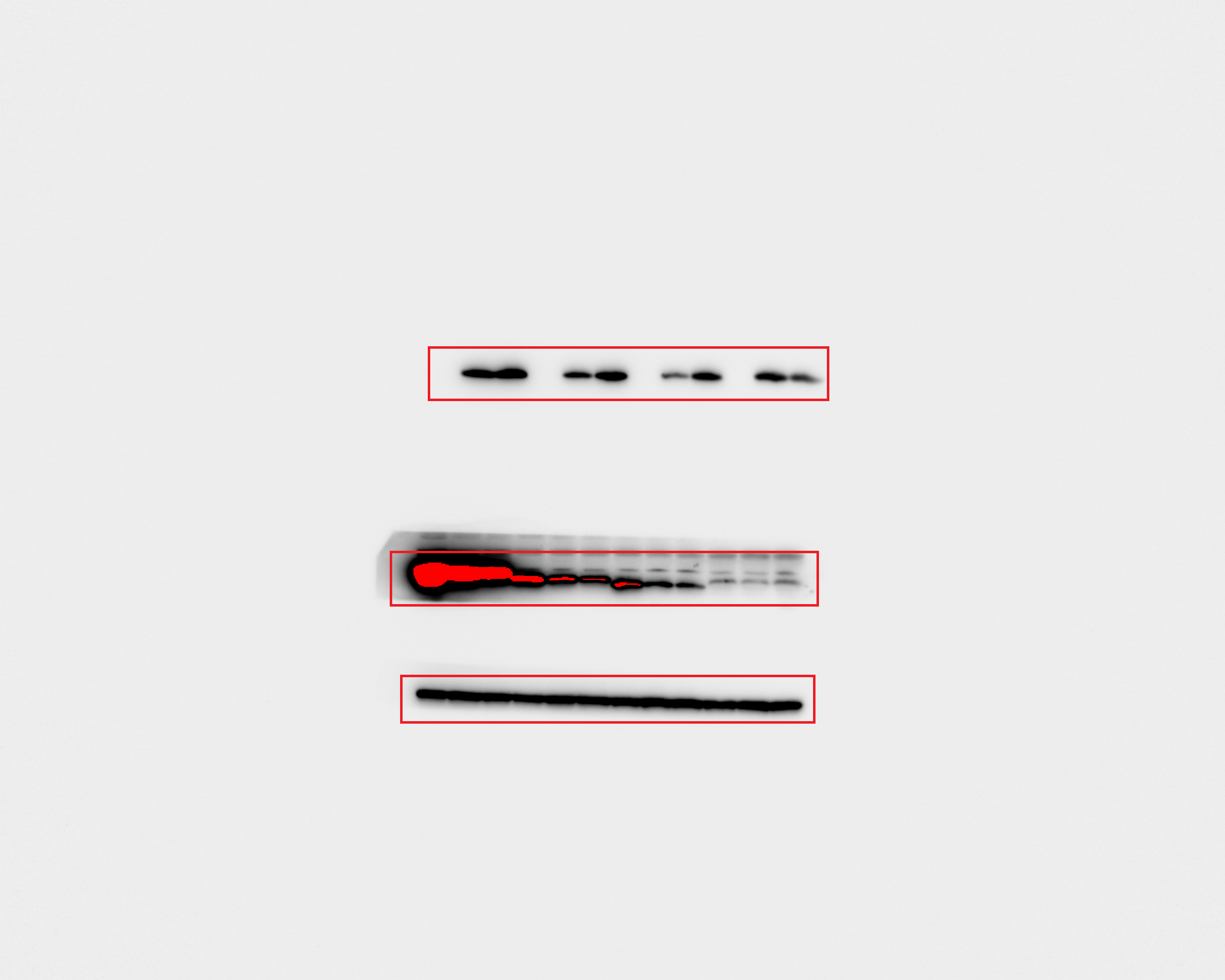

Supplement: Figure 3—source data 1. [file elife-101973-fig3-data1.zip › Figure 3-source data1/Fig3D-labeled/Long exposure.tif]

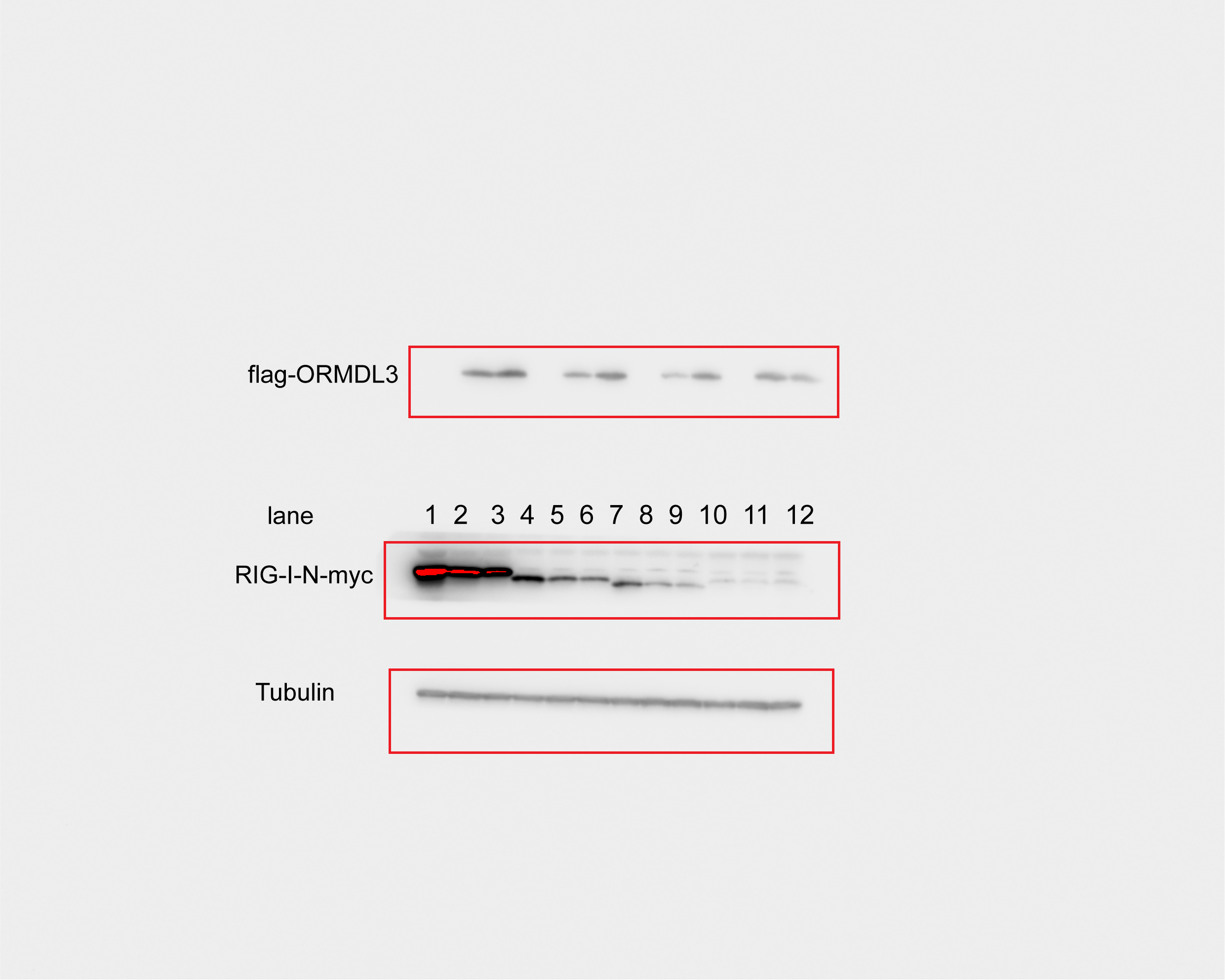

Supplement: Figure 3—source data 1. [file elife-101973-fig3-data1.zip › Figure 3-source data1/Fig3D-labeled/short exposure of myc flag and tubulin.tif]
