# Supplementary figures and images for: ORMDL3 restrains type I interferon signaling and anti-tumor immunity by promoting RIG-I degradation (part 2 of 3)
Source: eLife. 2025 Mar 24;13:RP101973. doi: 10.7554/eLife.101973 (PMC11932694; doi:10.7554/eLife.101973)

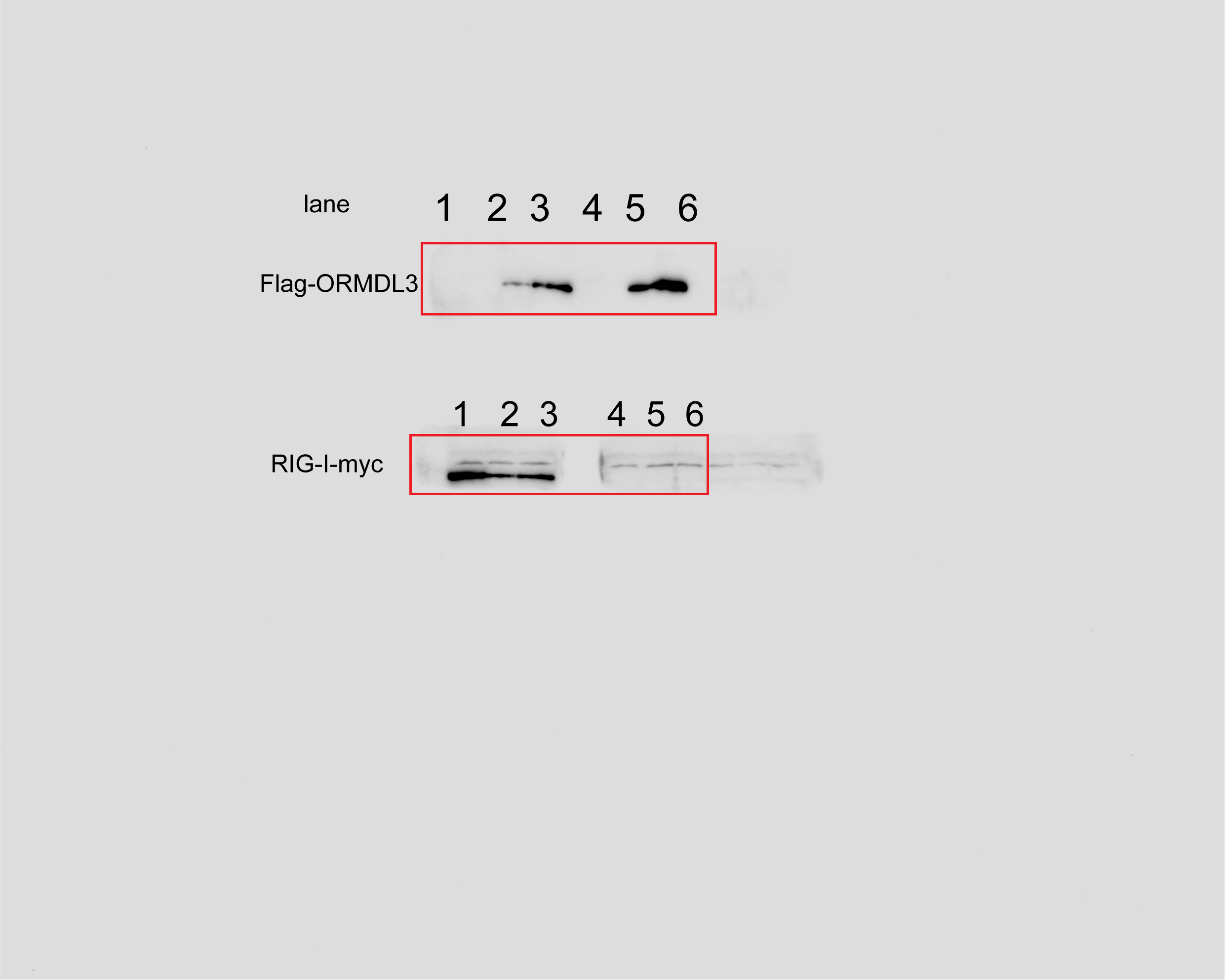

Supplement: Figure 3—source data 1. [file elife-101973-fig3-data1.zip › Figure 3-source data1/Fig3F-labeled/RIG-I-N-myc flag-ORMDL3.tif]

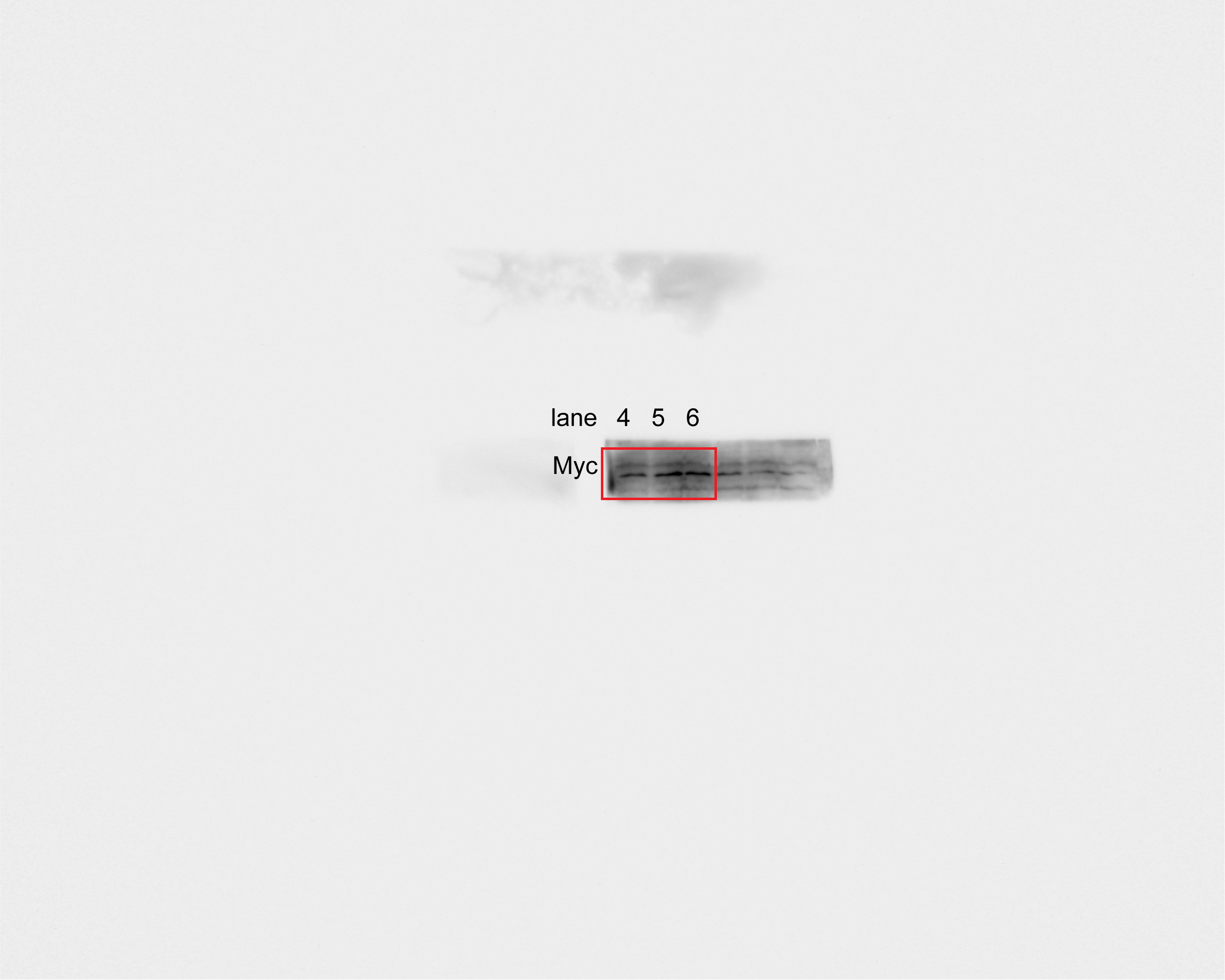

Supplement: Figure 3—source data 1. [file elife-101973-fig3-data1.zip › Figure 3-source data1/Fig3F-labeled/long exposure of RIG-I-4KR-Myc.tif]

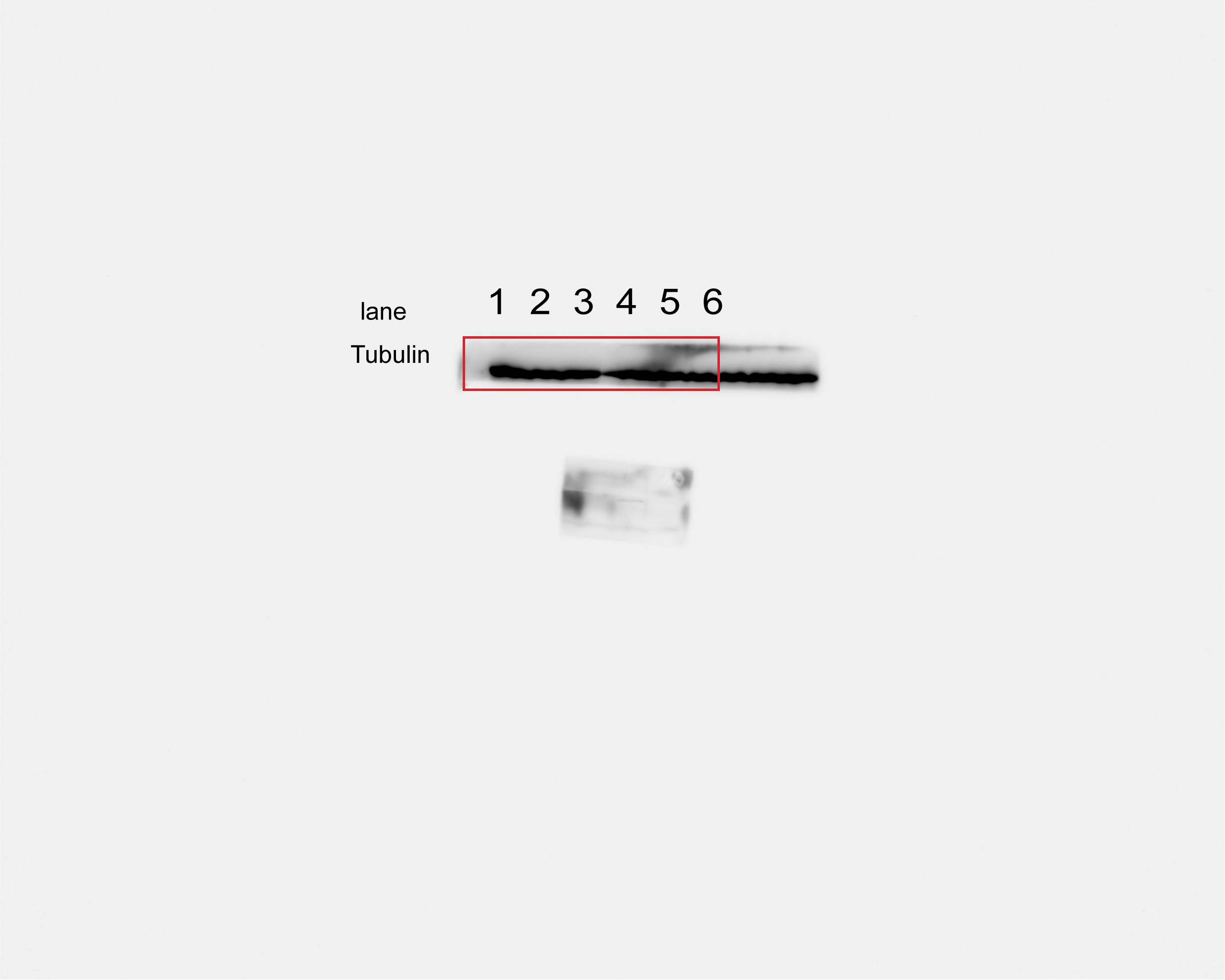

Supplement: Figure 3—source data 1. [file elife-101973-fig3-data1.zip › Figure 3-source data1/Fig3F-labeled/tubulin.tif]

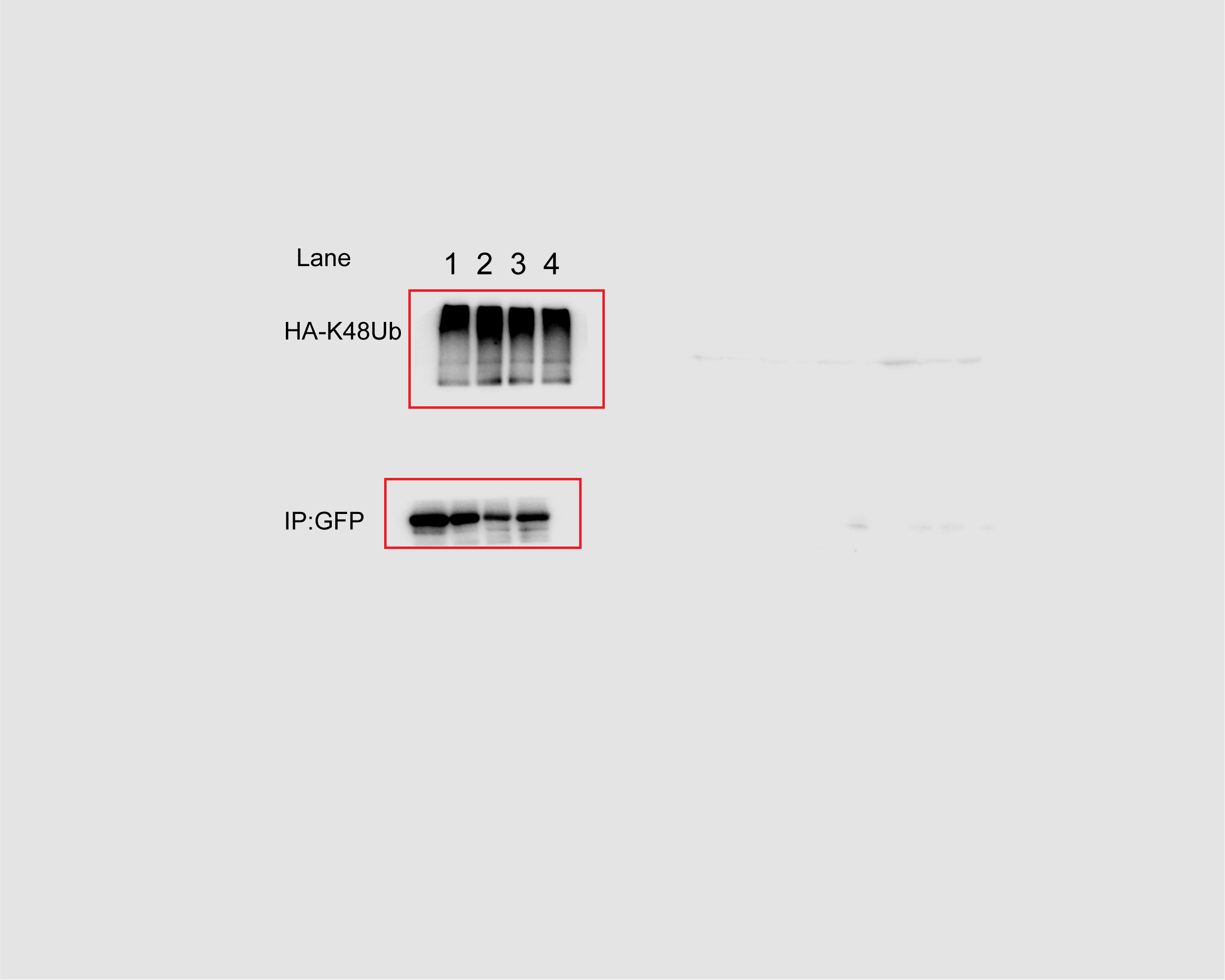

Supplement: Figure 3—source data 1. [file elife-101973-fig3-data1.zip › Figure 3-source data1/Fig3G-labeled/IP GFP and HA.tif]

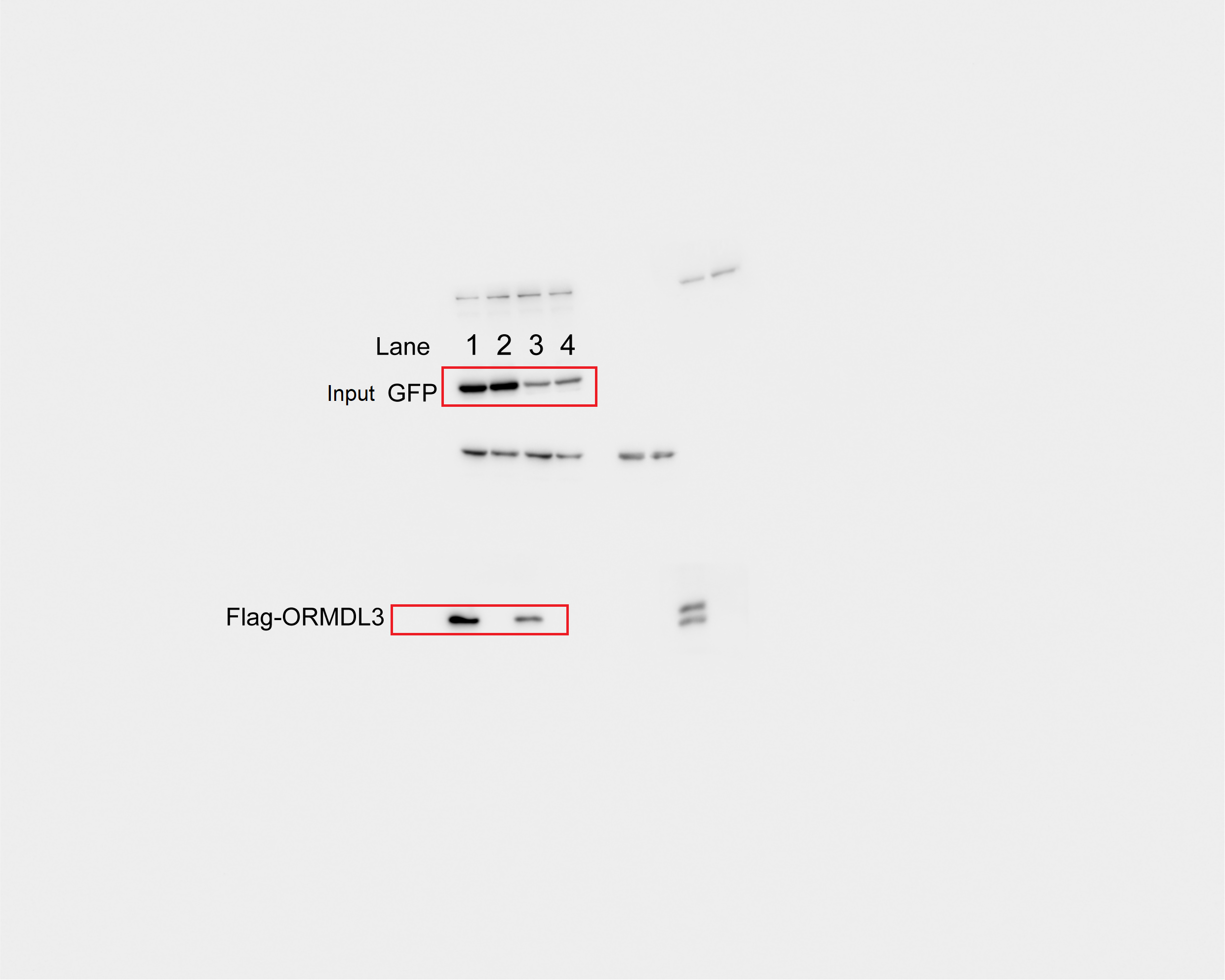

Supplement: Figure 3—source data 1. [file elife-101973-fig3-data1.zip › Figure 3-source data1/Fig3G-labeled/Input GFP and FLAG.tif]

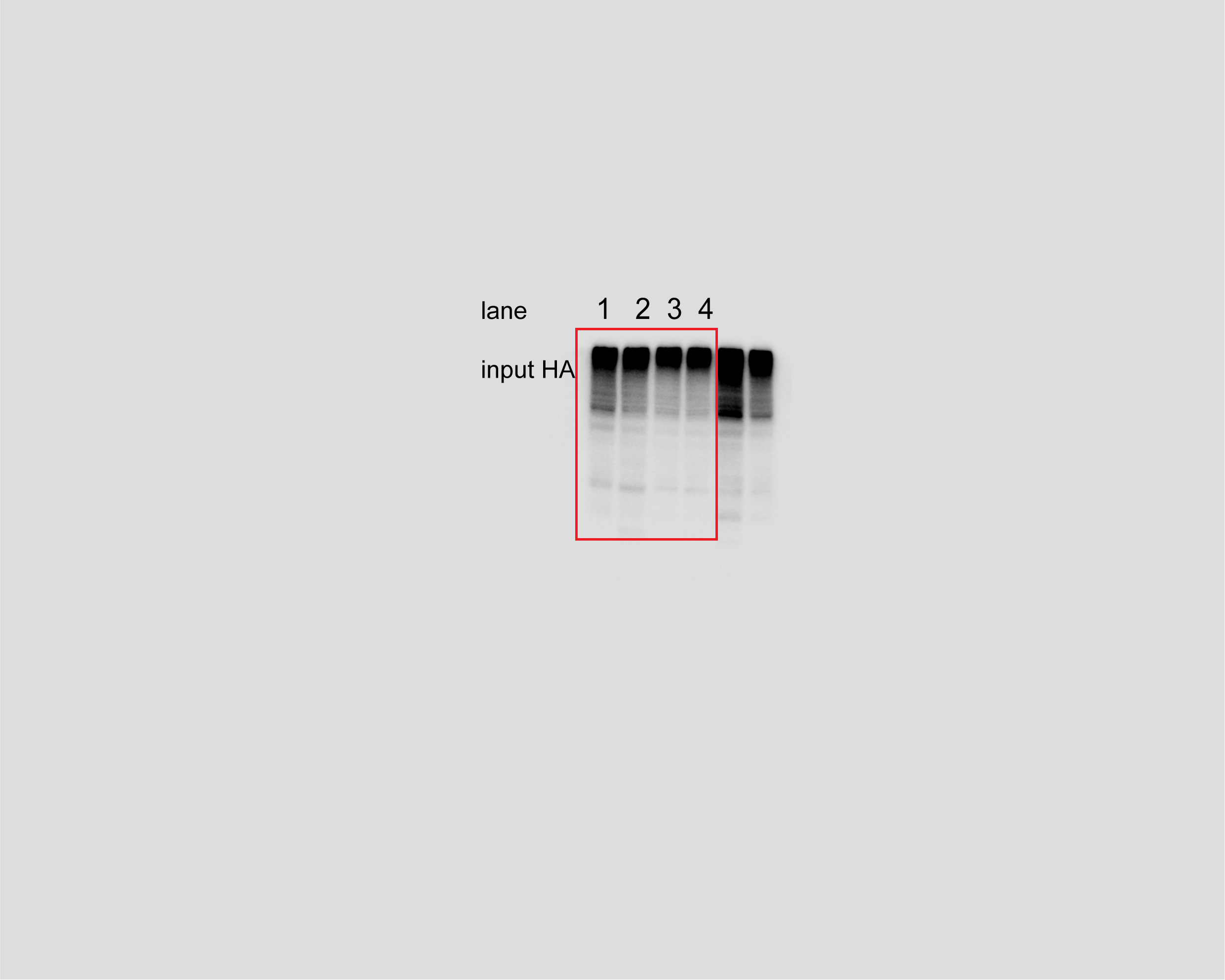

Supplement: Figure 3—source data 1. [file elife-101973-fig3-data1.zip › Figure 3-source data1/Fig3G-labeled/Input HA.tif]

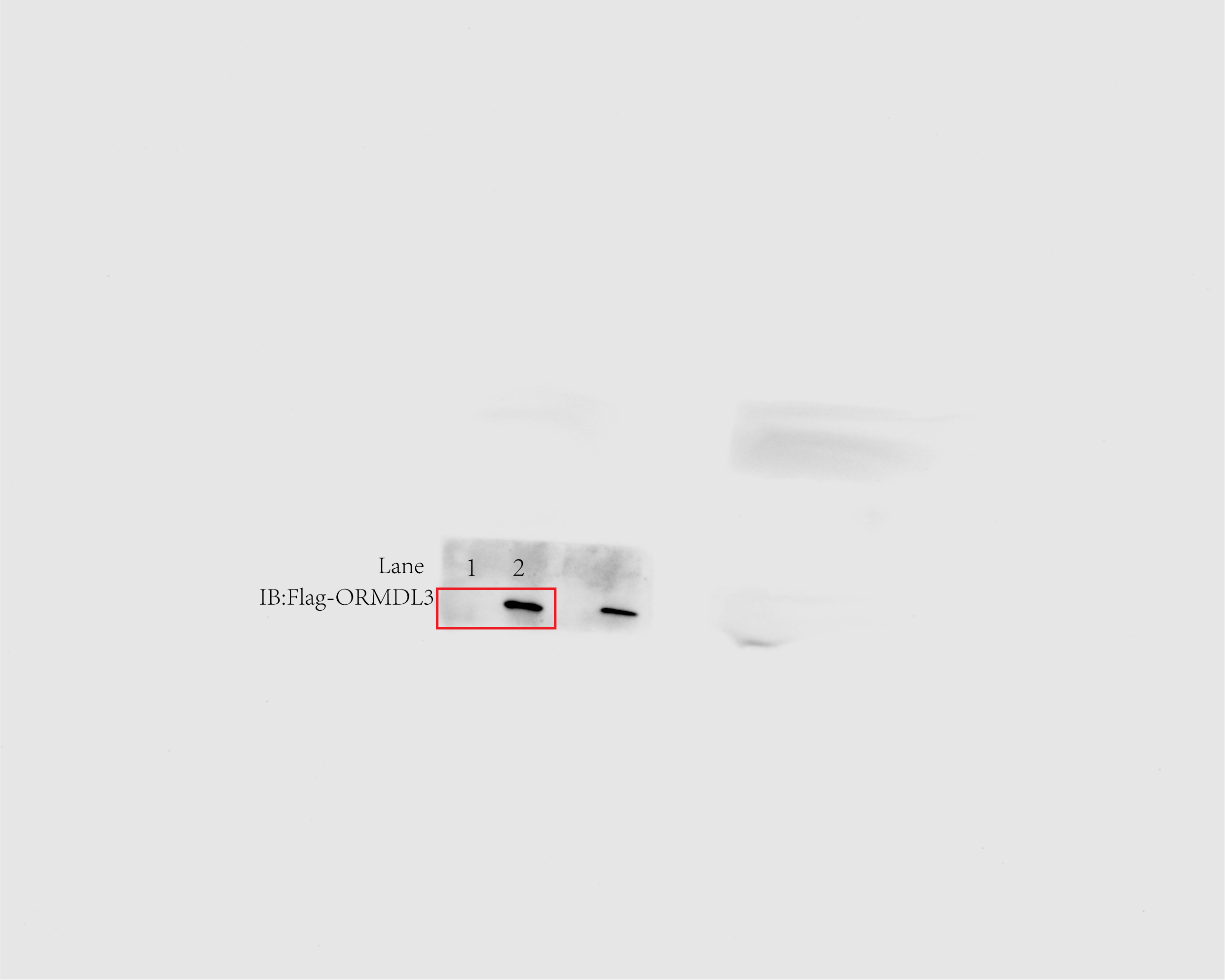

Supplement: Figure 3—source data 1. [file elife-101973-fig3-data1.zip › Figure 3-source data1/Fig3H-labeled/Flag.tif]

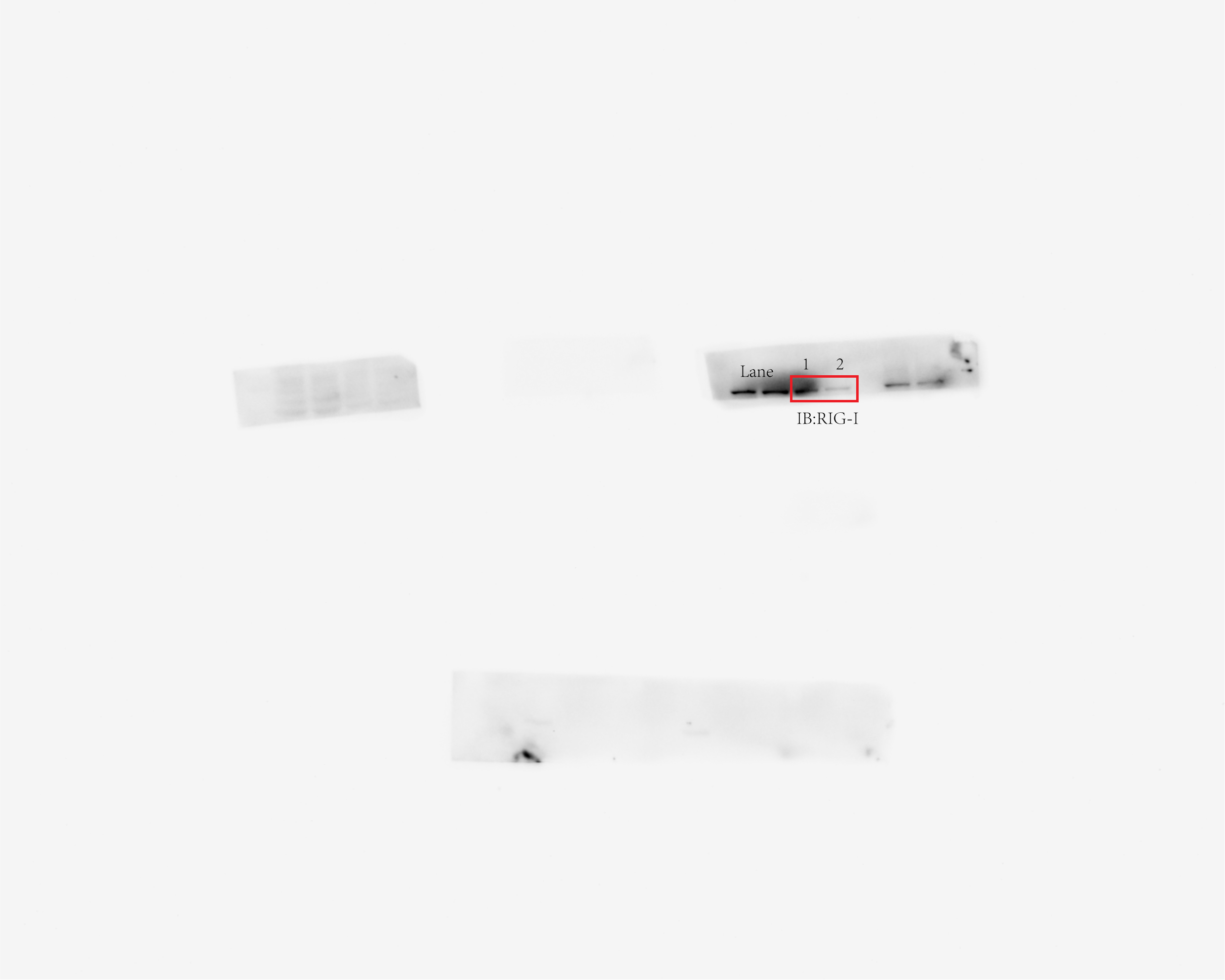

Supplement: Figure 3—source data 1. [file elife-101973-fig3-data1.zip › Figure 3-source data1/Fig3H-labeled/RIG-I.tif]

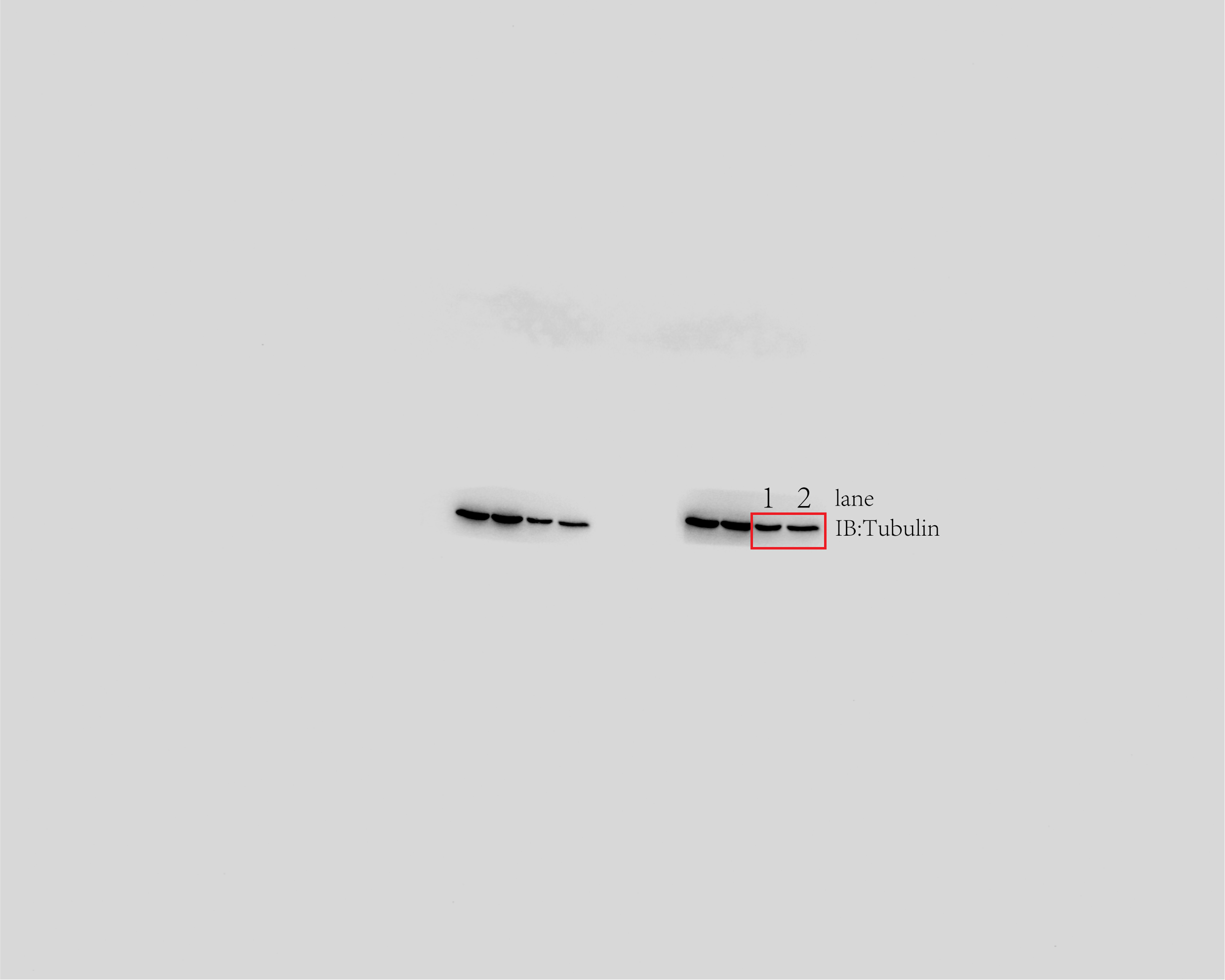

Supplement: Figure 3—source data 1. [file elife-101973-fig3-data1.zip › Figure 3-source data1/Fig3H-labeled/Tubulin.tif]

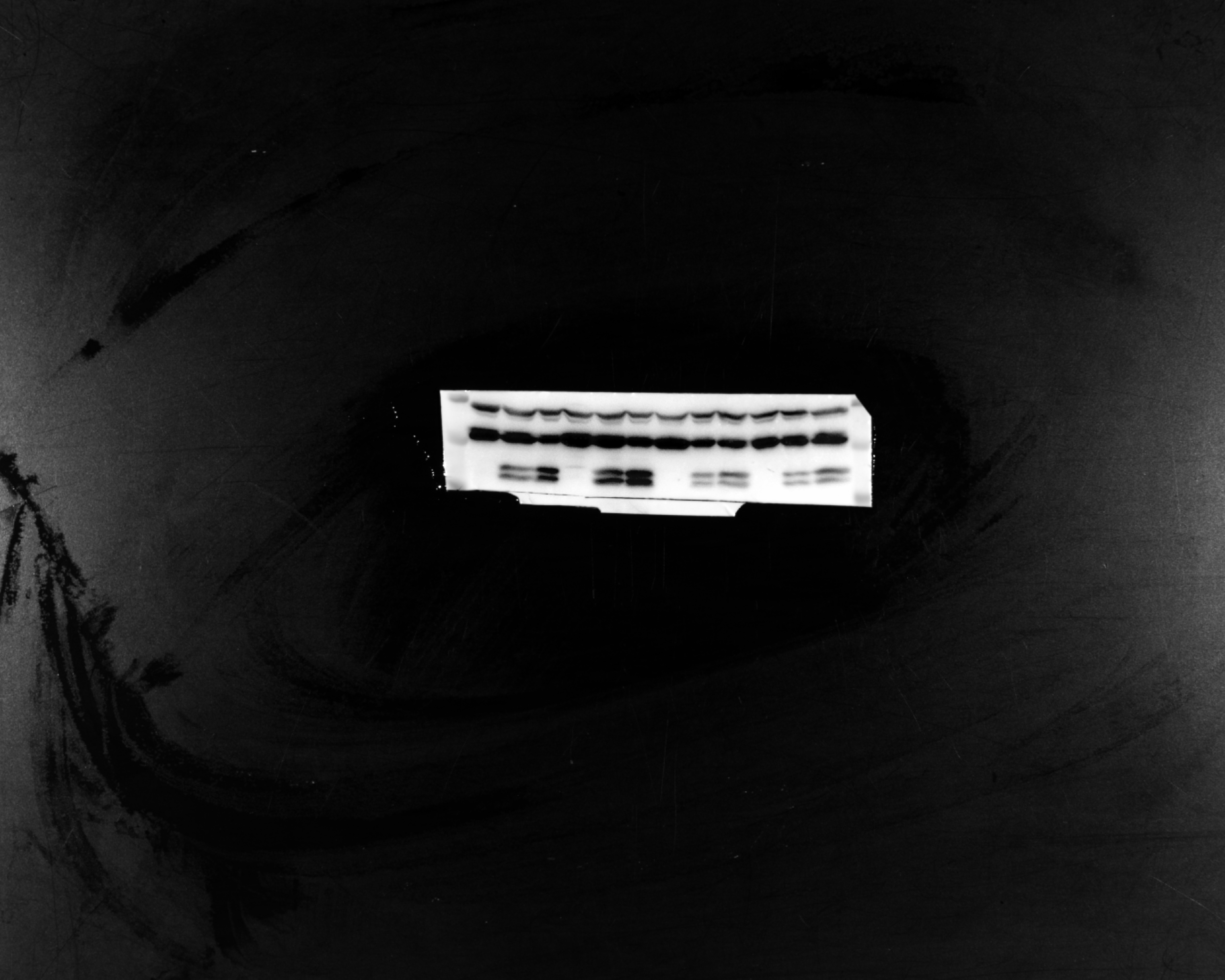

Supplement: Figure 3—source data 2. [file elife-101973-fig3-data2.zip › Figure 3-source data 2/figure 3A/RIG-I-N-Myc ORMDL3-Myc .jpg]

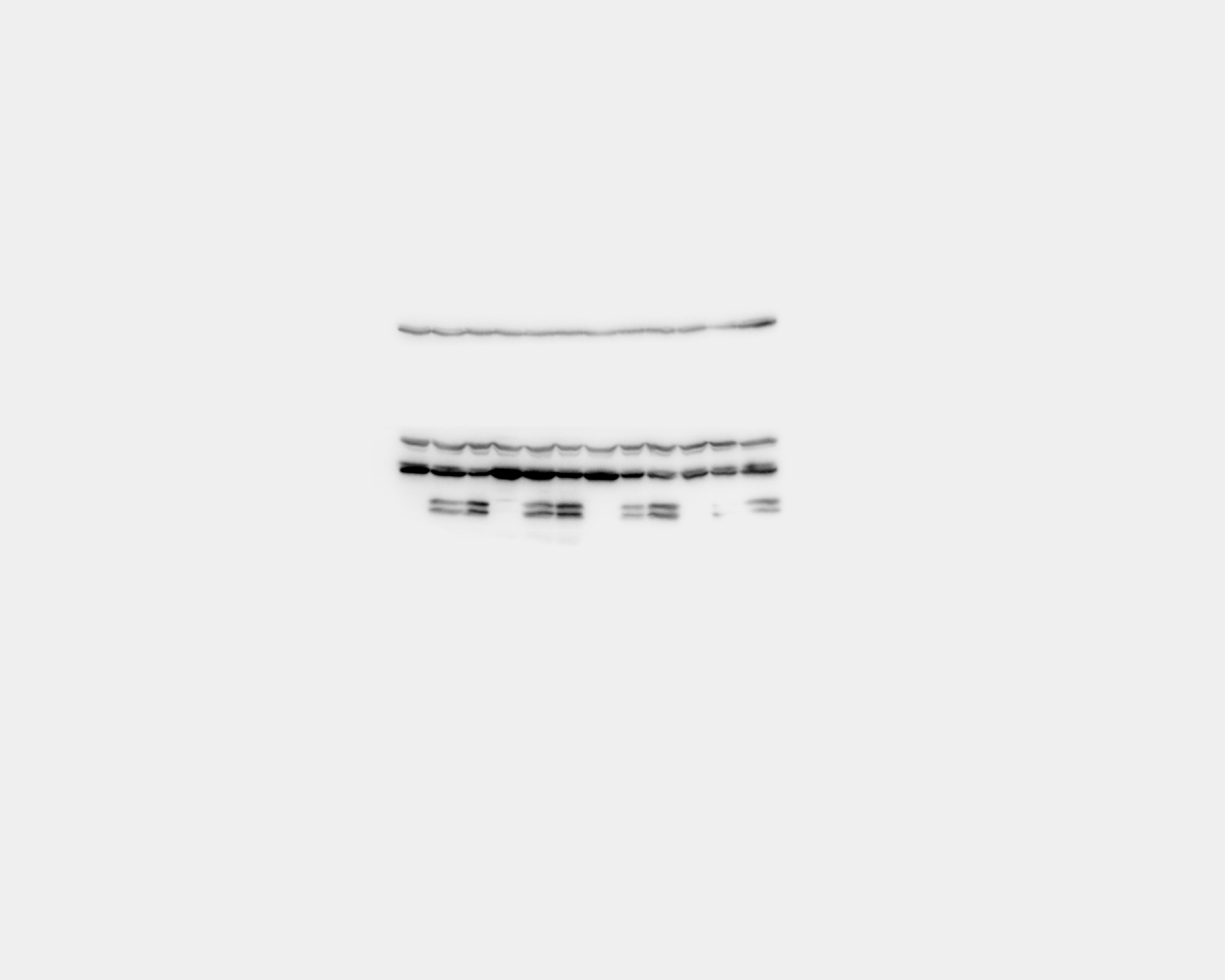

Supplement: Figure 3—source data 2. [file elife-101973-fig3-data2.zip › Figure 3-source data 2/figure 3A/RIG-I-N-Myc ORMDL3-Myc and tubulin.jpg]

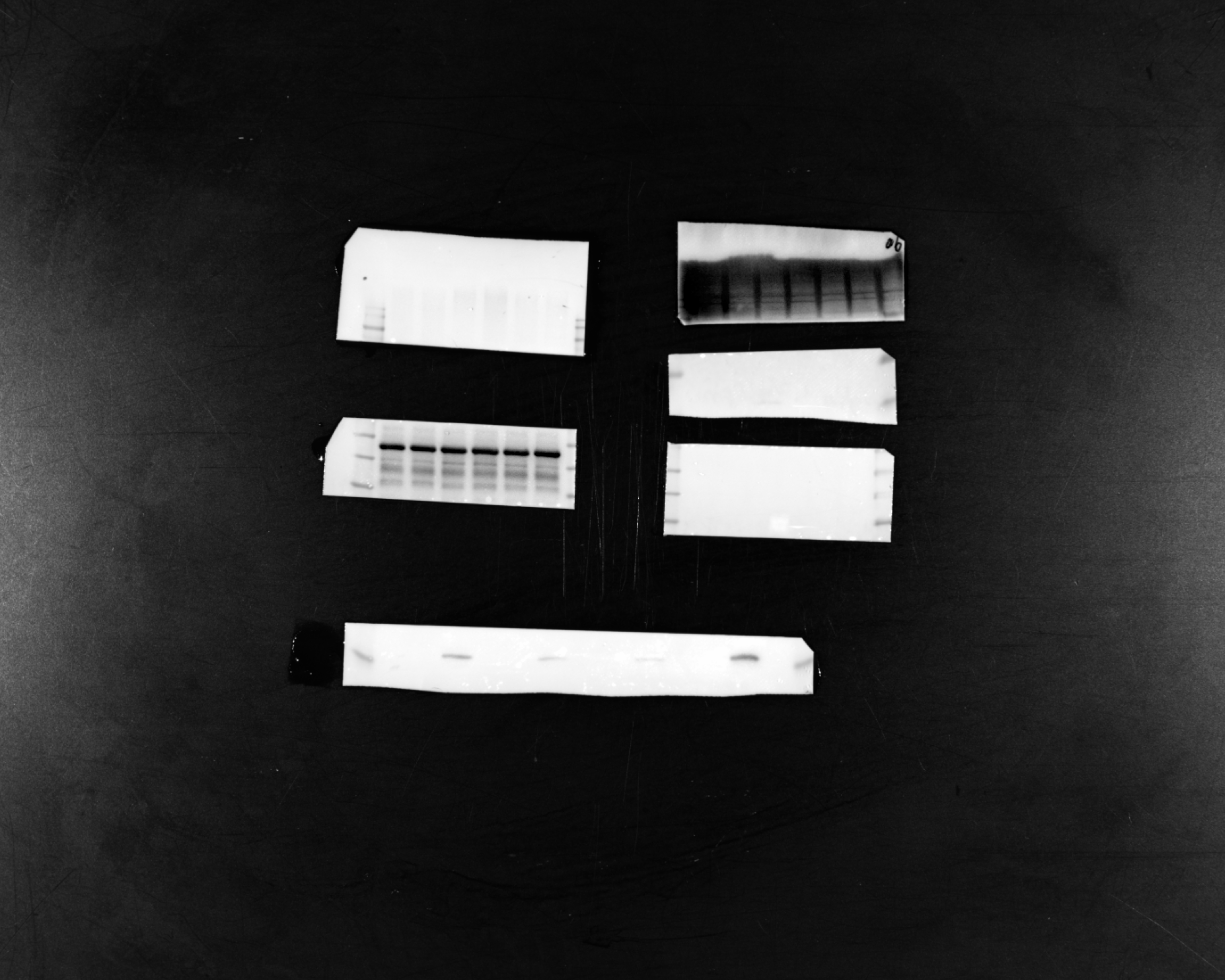

Supplement: Figure 3—source data 2. [file elife-101973-fig3-data2.zip › Figure 3-source data 2/figure 3B/IP GFP.jpg]

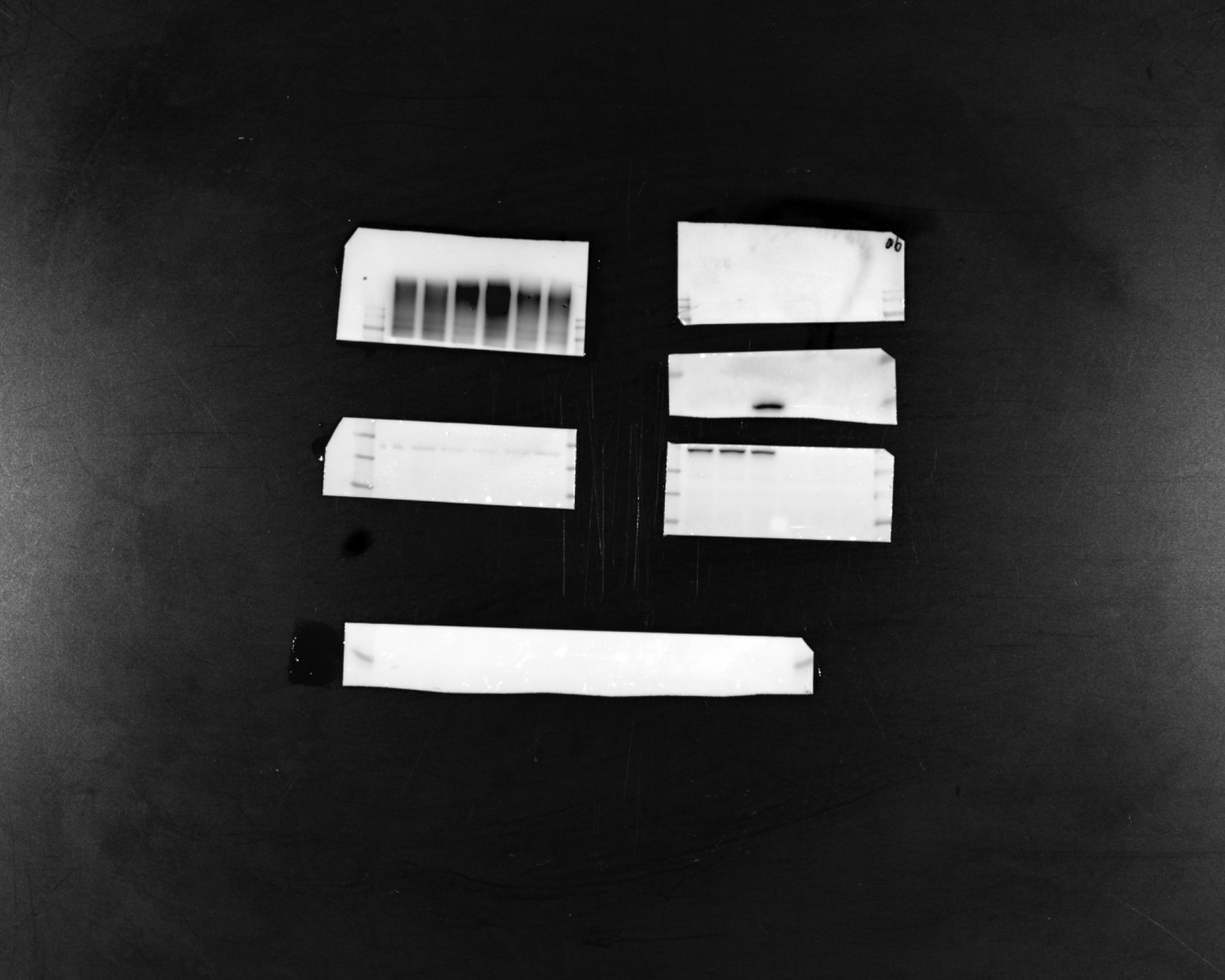

Supplement: Figure 3—source data 2. [file elife-101973-fig3-data2.zip › Figure 3-source data 2/figure 3B/IP HA.jpg]

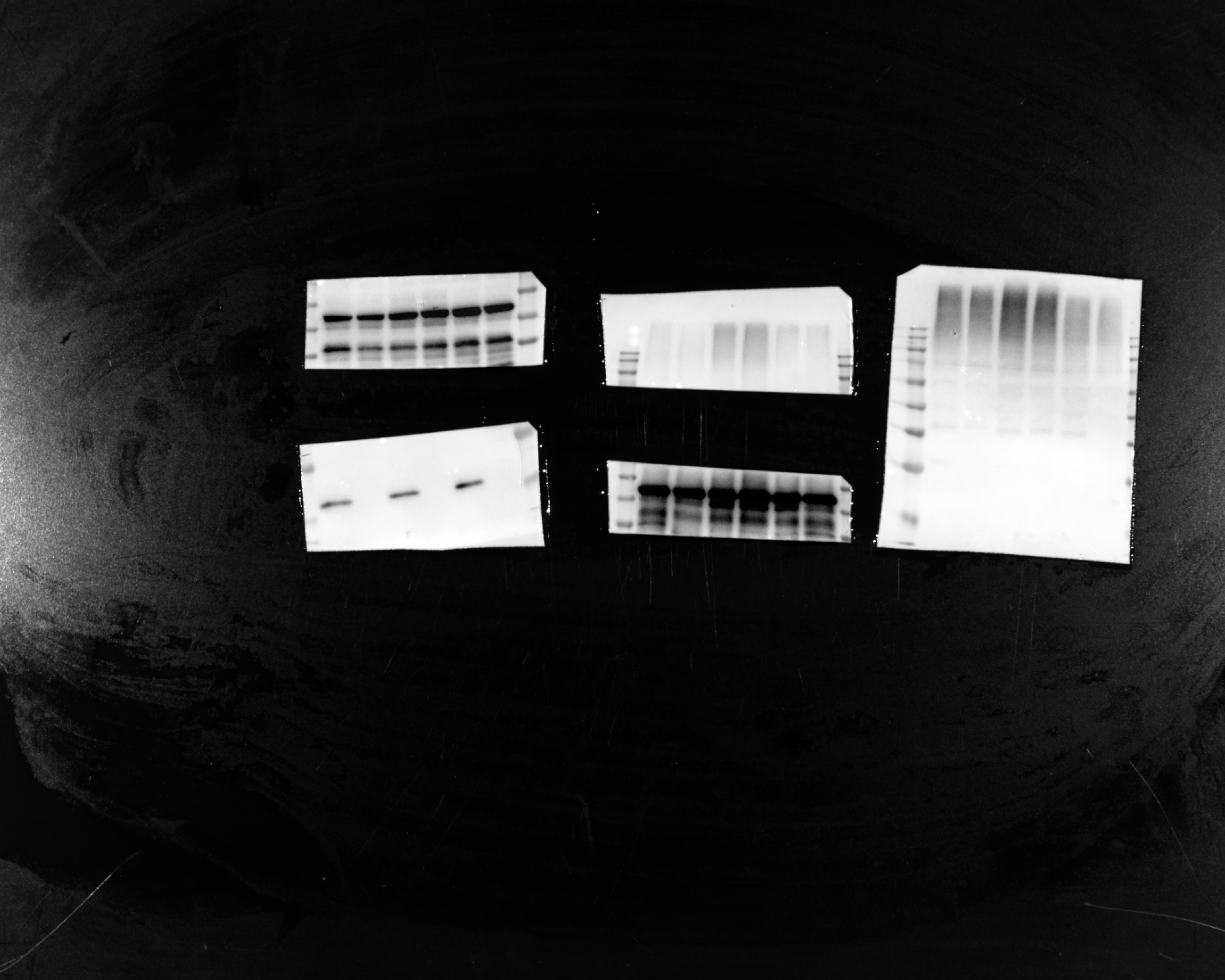

Supplement: Figure 3—source data 2. [file elife-101973-fig3-data2.zip › Figure 3-source data 2/figure 3B/input GFP Flag.jpg]

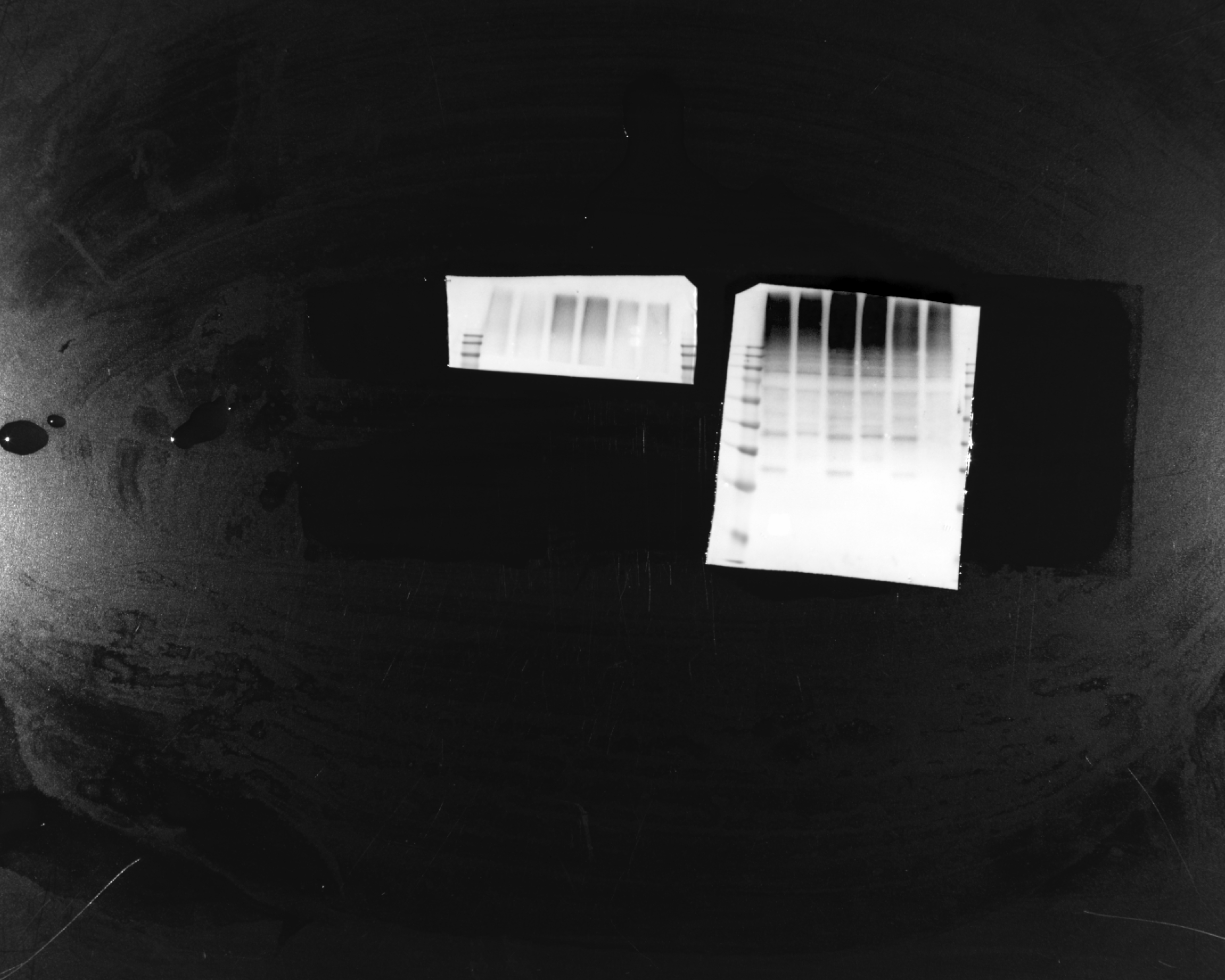

Supplement: Figure 3—source data 2. [file elife-101973-fig3-data2.zip › Figure 3-source data 2/figure 3B/input HA.jpg]

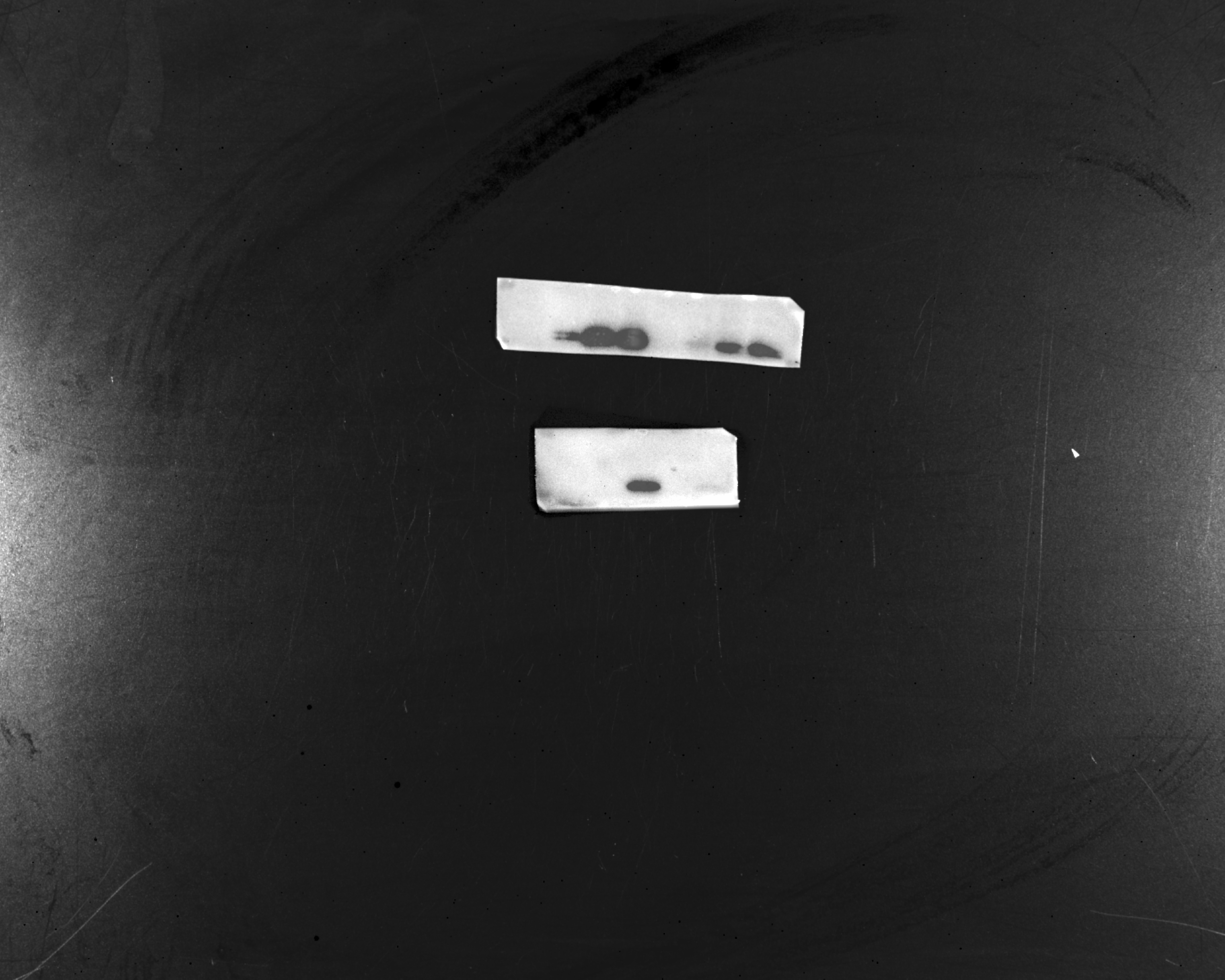

Supplement: Figure 3—source data 2. [file elife-101973-fig3-data2.zip › Figure 3-source data 2/figure 3C/Flag ORMDL3.jpg]

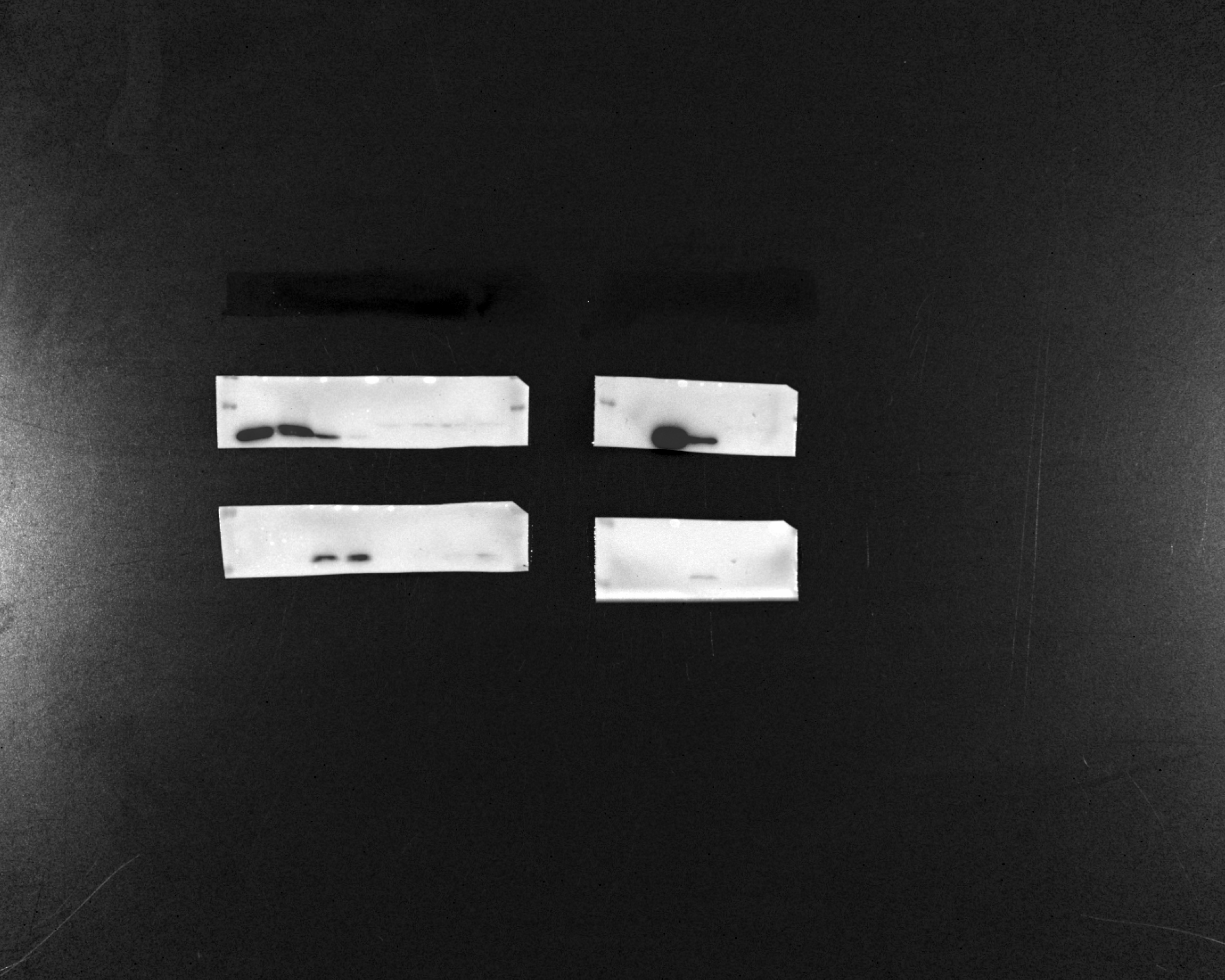

Supplement: Figure 3—source data 2. [file elife-101973-fig3-data2.zip › Figure 3-source data 2/figure 3C/RIG-I-N-Myc.jpg]

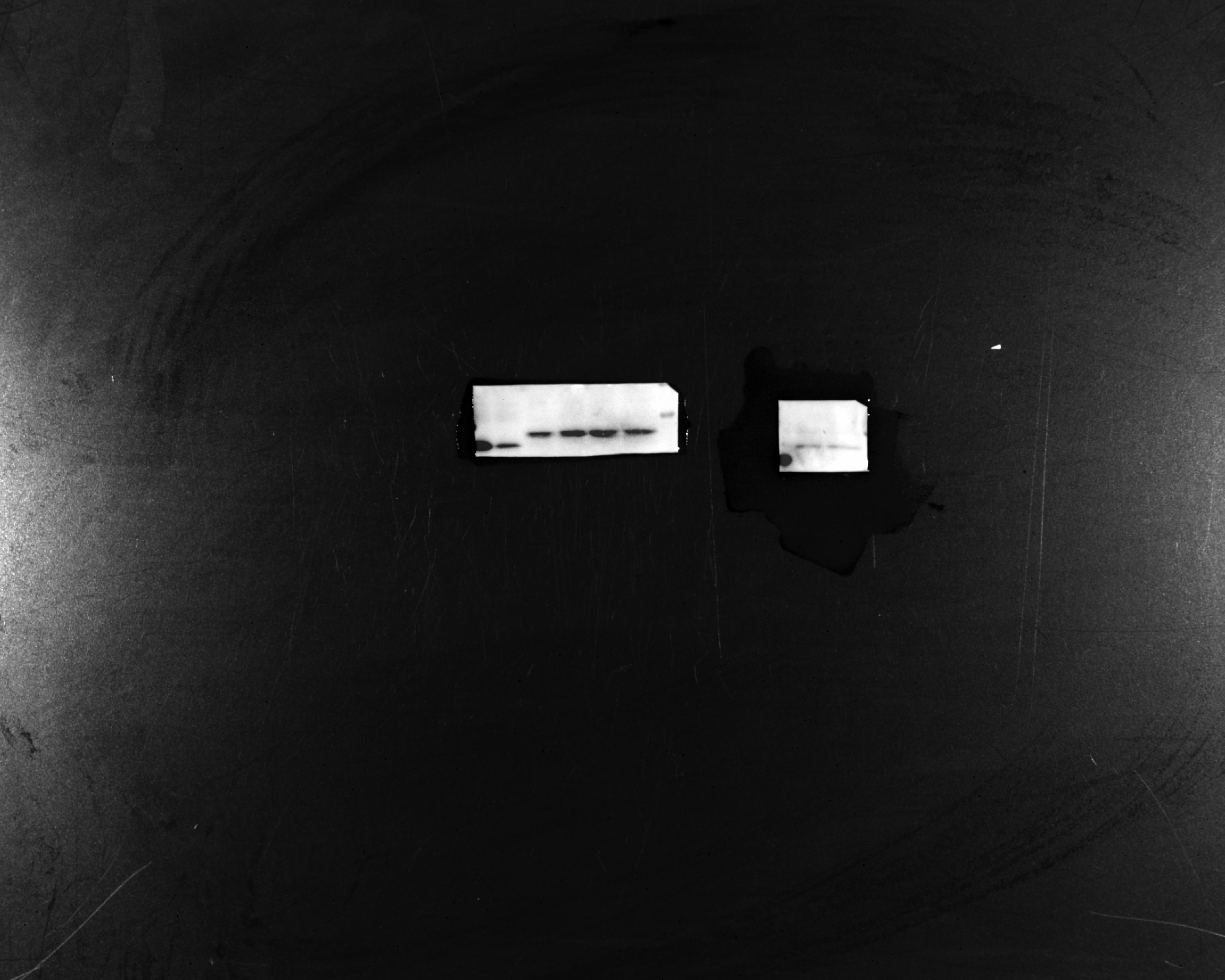

Supplement: Figure 3—source data 2. [file elife-101973-fig3-data2.zip › Figure 3-source data 2/figure 3C/long exposure of myc.jpg]

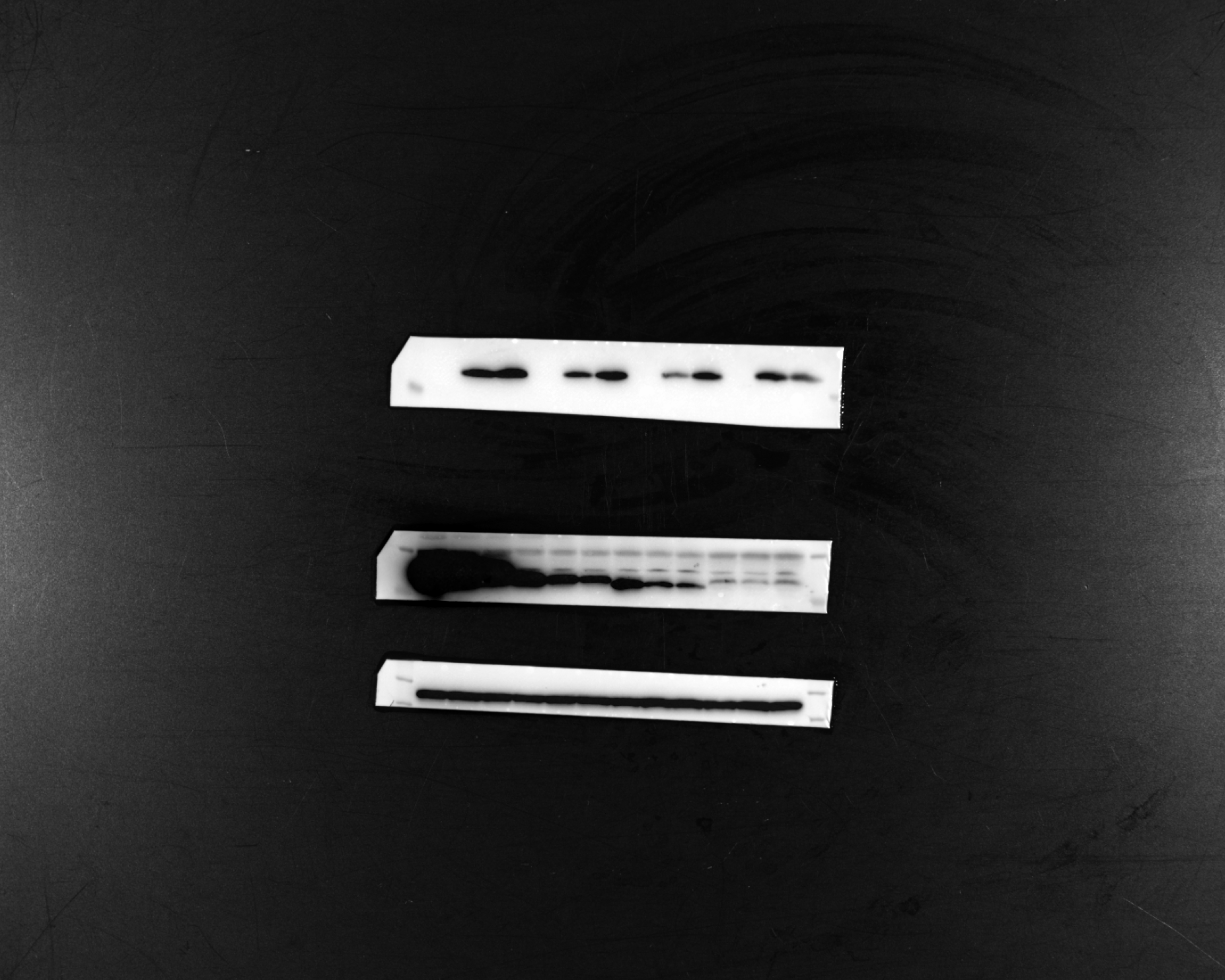

Supplement: Figure 3—source data 2. [file elife-101973-fig3-data2.zip › Figure 3-source data 2/figure 3D/long exposure of myc flag and tubulin.jpg]

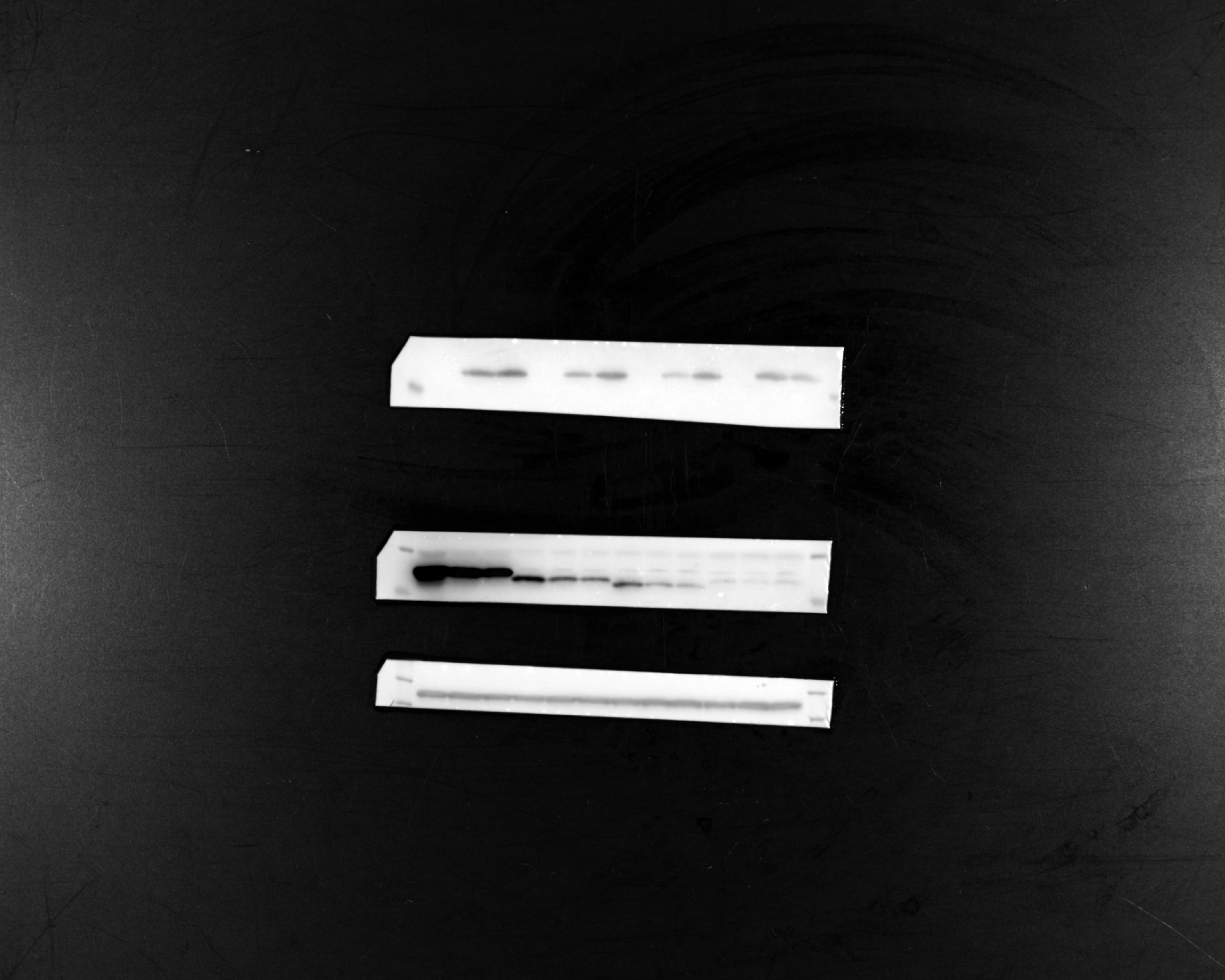

Supplement: Figure 3—source data 2. [file elife-101973-fig3-data2.zip › Figure 3-source data 2/figure 3D/short exposure of myc flag and tubulin.jpg]

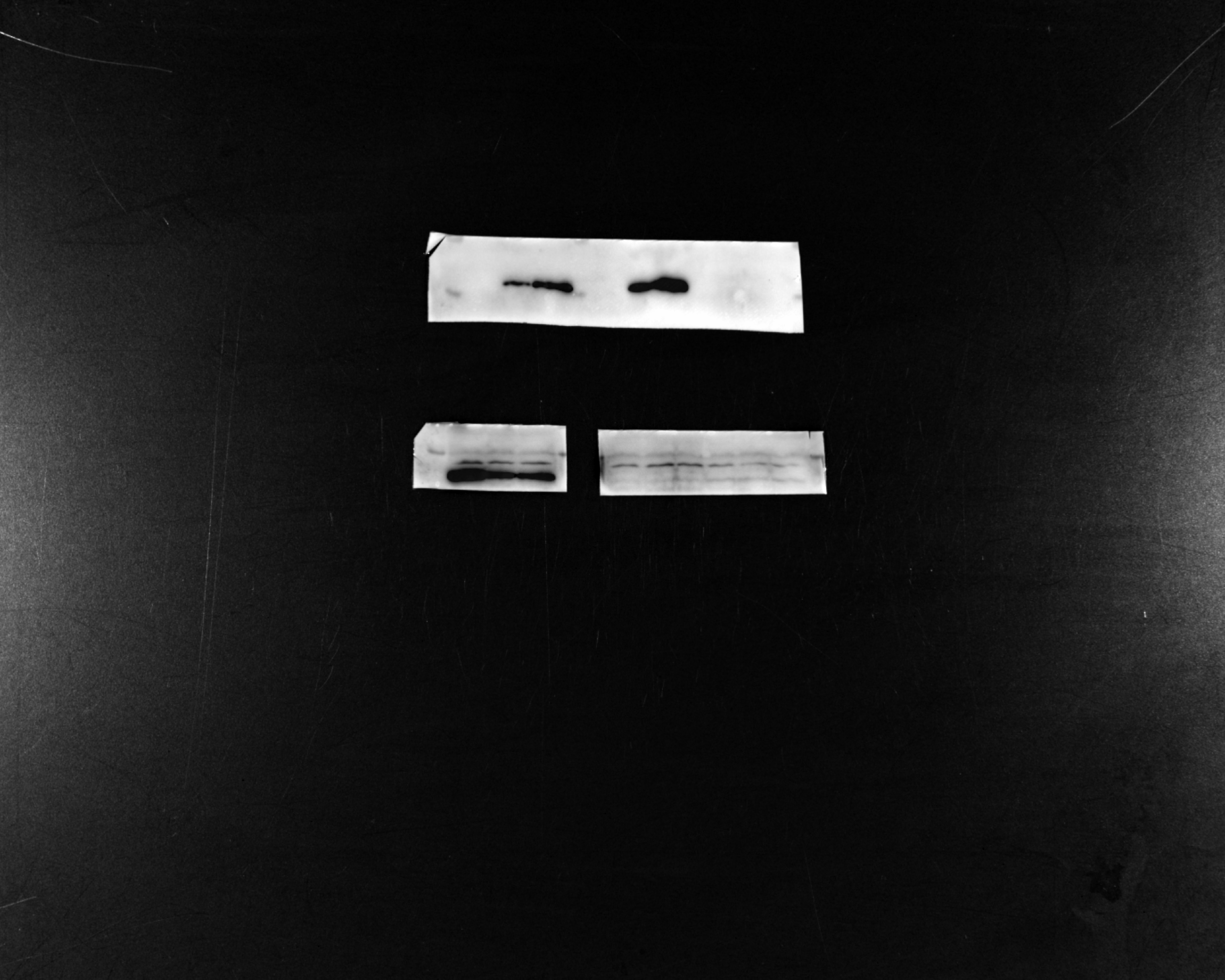

Supplement: Figure 3—source data 2. [file elife-101973-fig3-data2.zip › Figure 3-source data 2/figure 3F/long exposure of myc and flag.jpg]

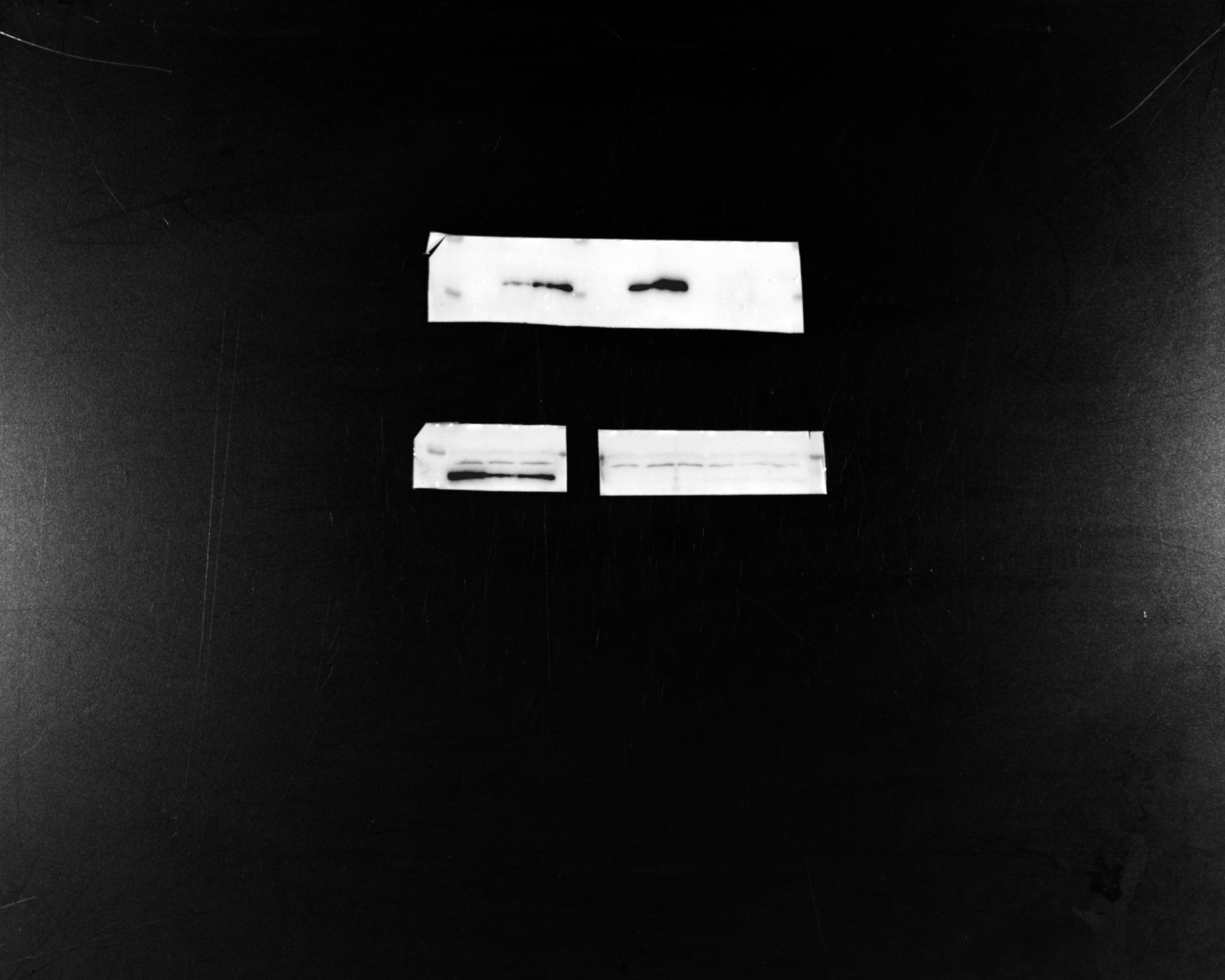

Supplement: Figure 3—source data 2. [file elife-101973-fig3-data2.zip › Figure 3-source data 2/figure 3F/short exposure of myc and flag.jpg]

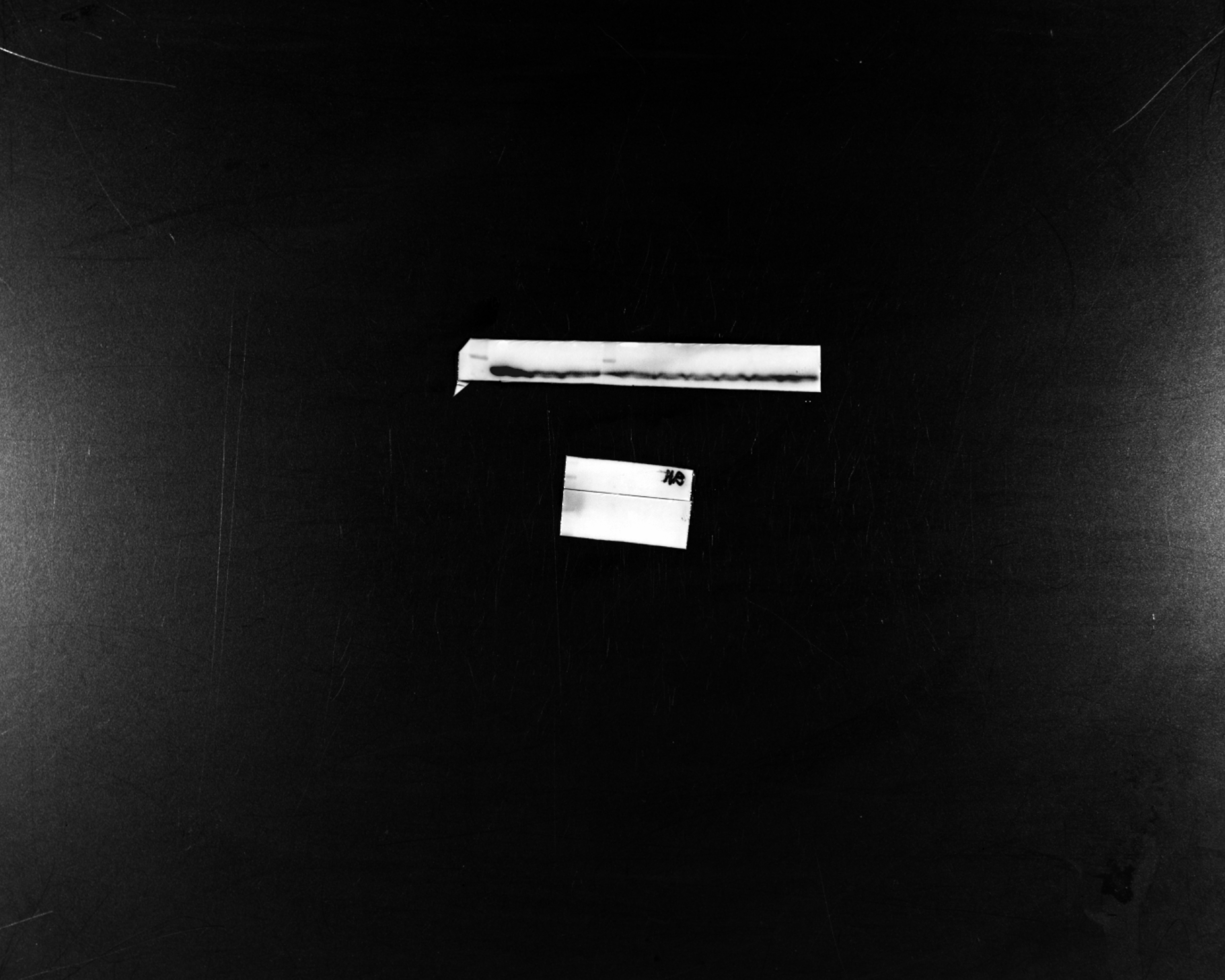

Supplement: Figure 3—source data 2. [file elife-101973-fig3-data2.zip › Figure 3-source data 2/figure 3F/tubulin.jpg]

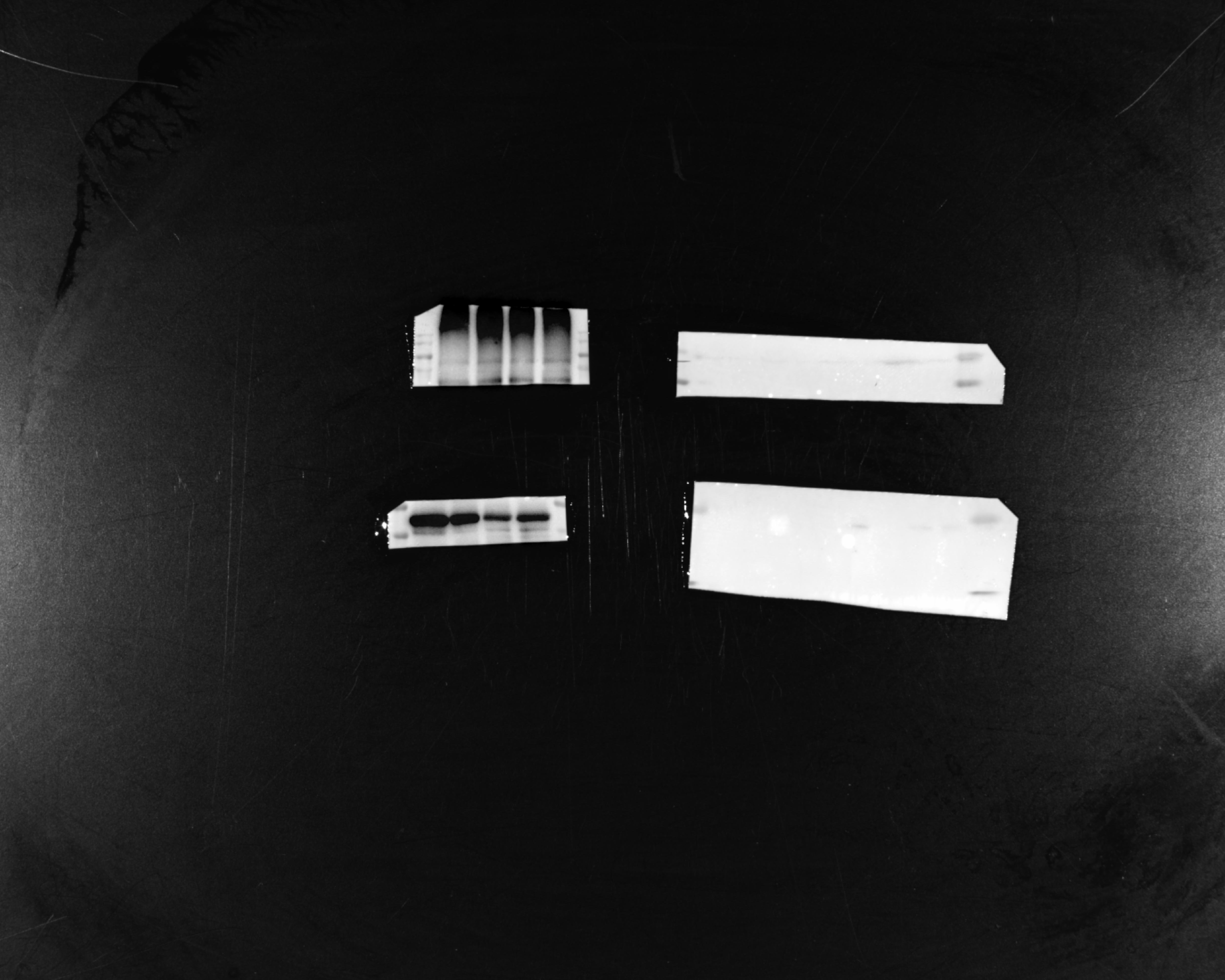

Supplement: Figure 3—source data 2. [file elife-101973-fig3-data2.zip › Figure 3-source data 2/figure 3G/IP GFP and HA.jpg]

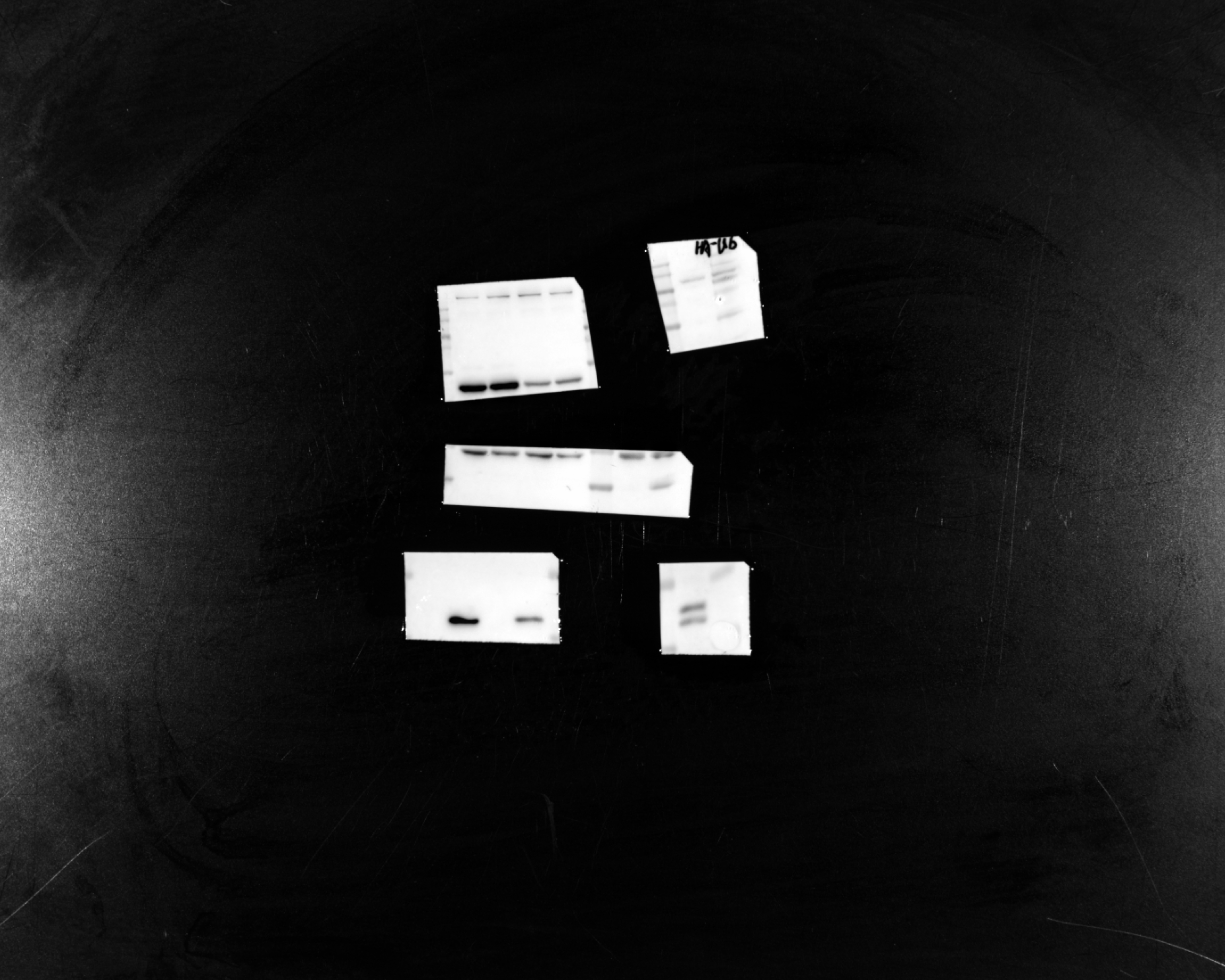

Supplement: Figure 3—source data 2. [file elife-101973-fig3-data2.zip › Figure 3-source data 2/figure 3G/input GFP and flag.jpg]

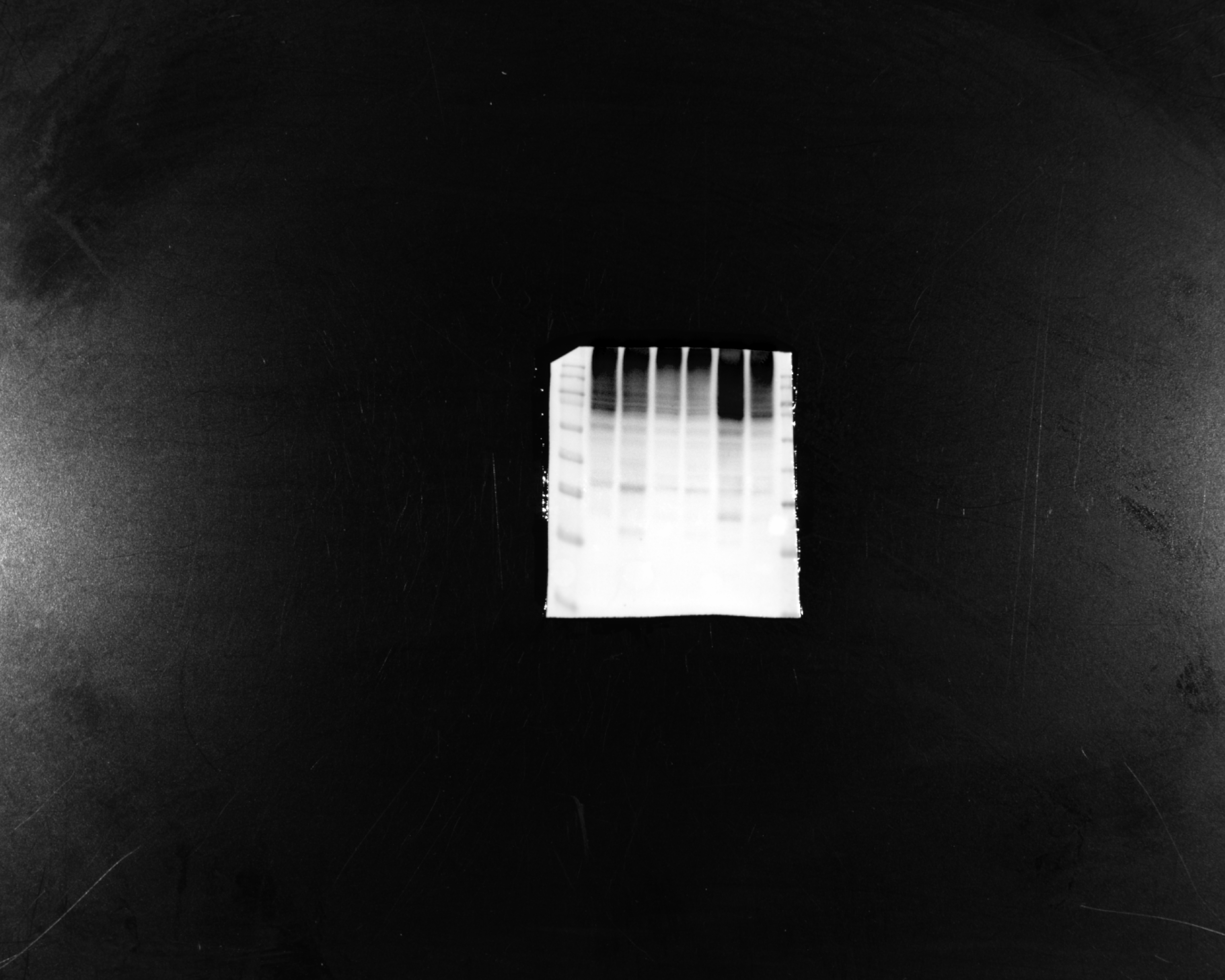

Supplement: Figure 3—source data 2. [file elife-101973-fig3-data2.zip › Figure 3-source data 2/figure 3G/input HA.jpg]

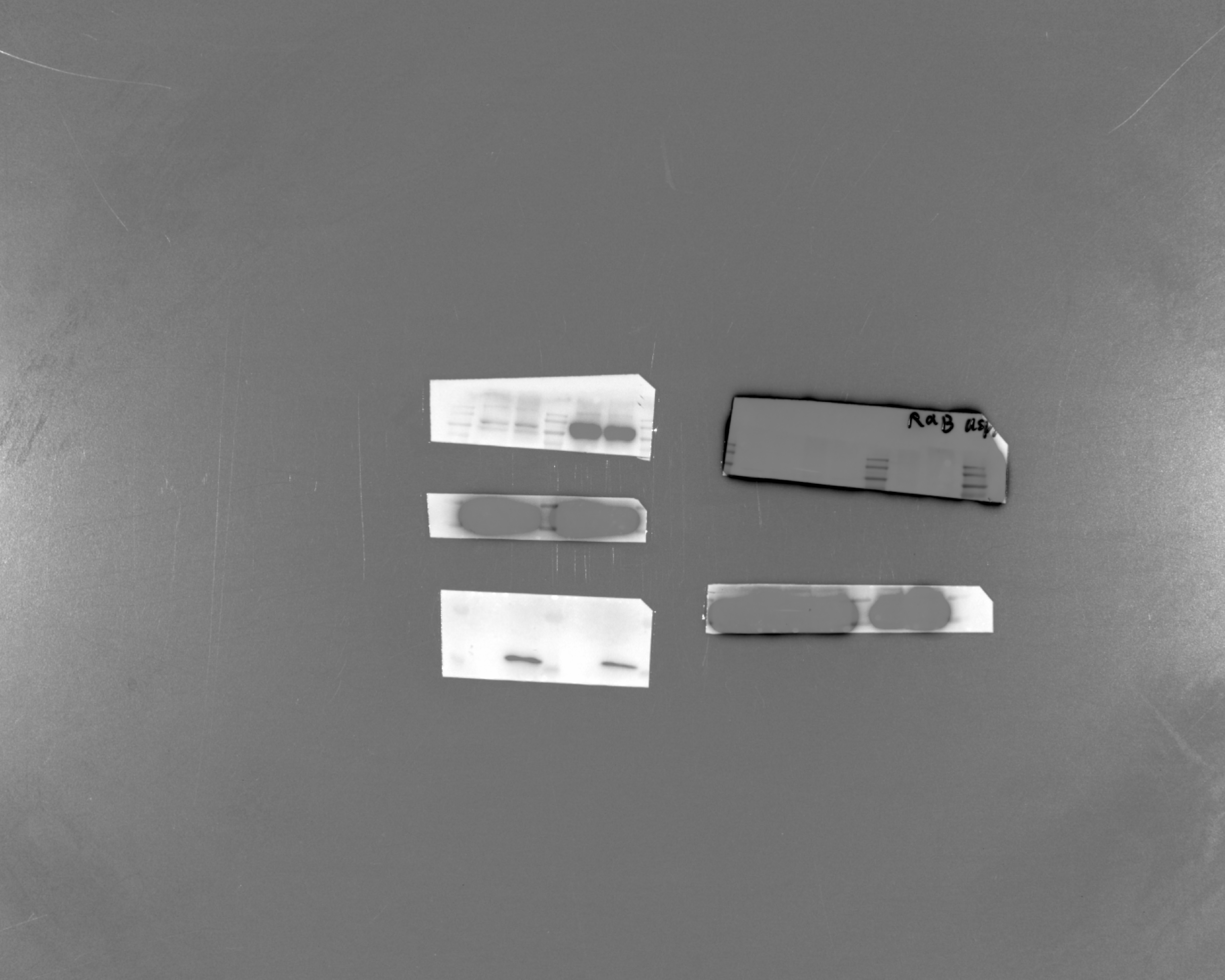

Supplement: Figure 3—source data 2. [file elife-101973-fig3-data2.zip › Figure 3-source data 2/figure 3H/Flag.jpg]

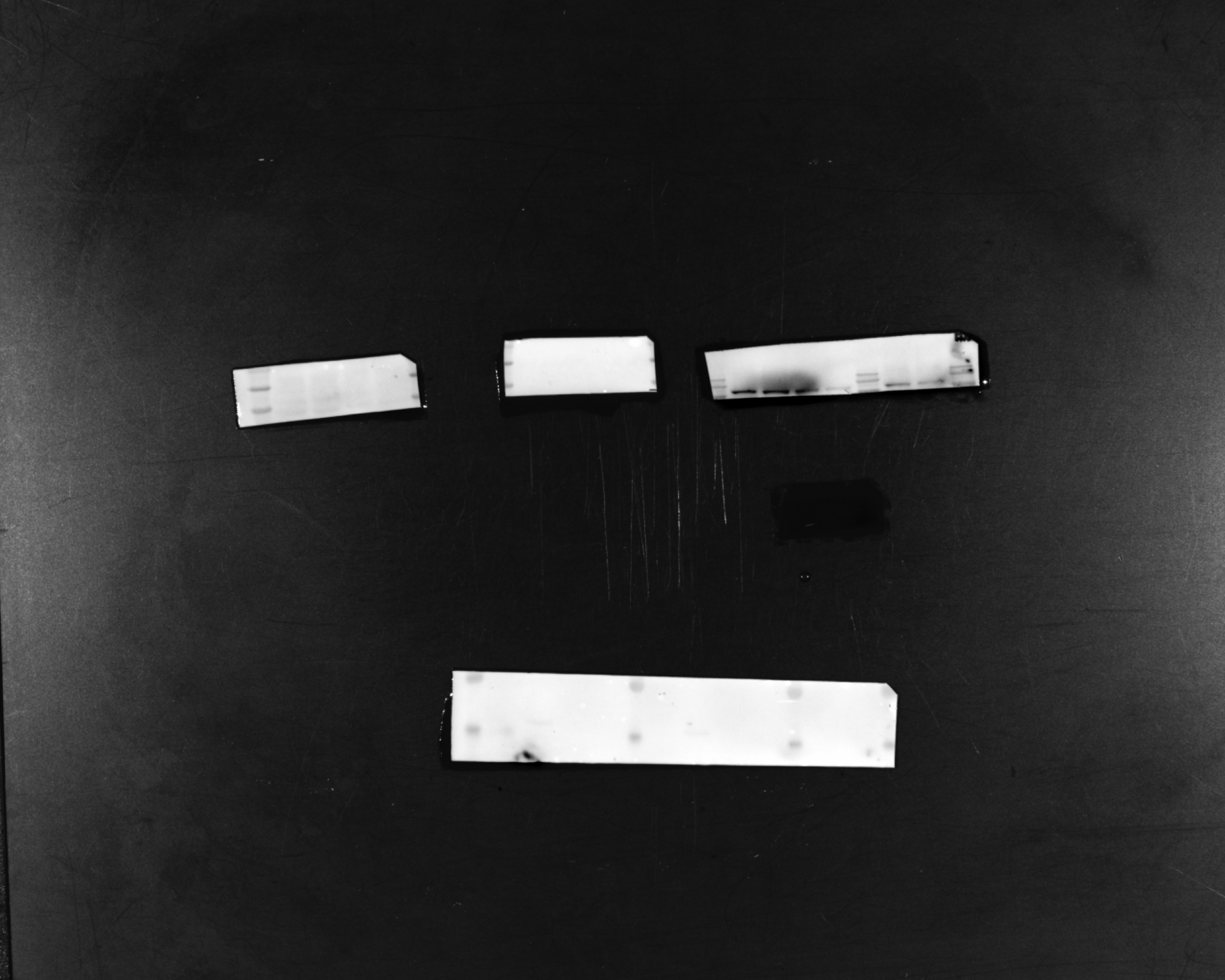

Supplement: Figure 3—source data 2. [file elife-101973-fig3-data2.zip › Figure 3-source data 2/figure 3H/RIG-I.jpg]

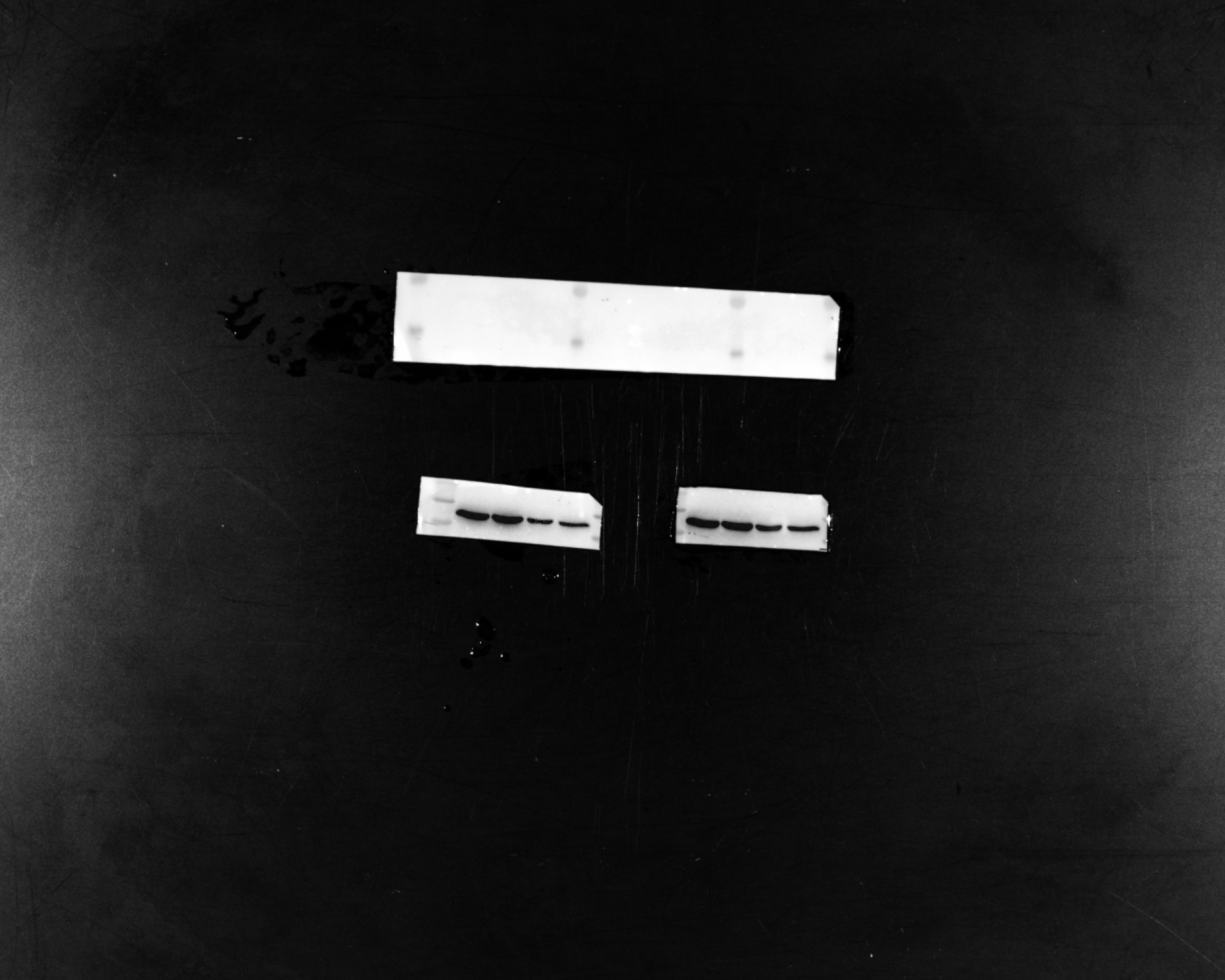

Supplement: Figure 3—source data 2. [file elife-101973-fig3-data2.zip › Figure 3-source data 2/figure 3H/tubulin.jpg]

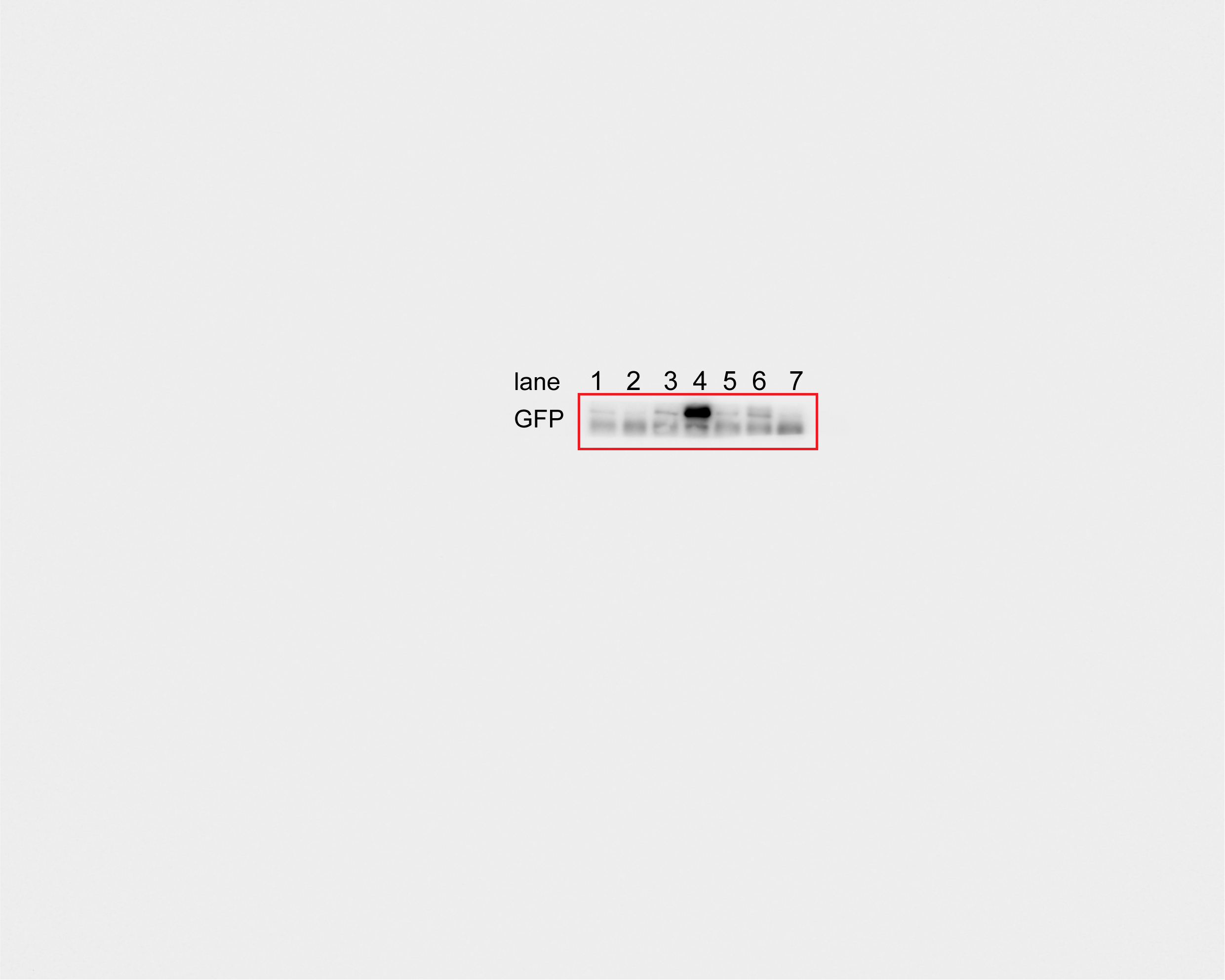

Supplement: Figure 4—source data 1. [file elife-101973-fig4-data1.zip › Figure 4-source data 1/Fig4A-labeled/IP GFP.tif]

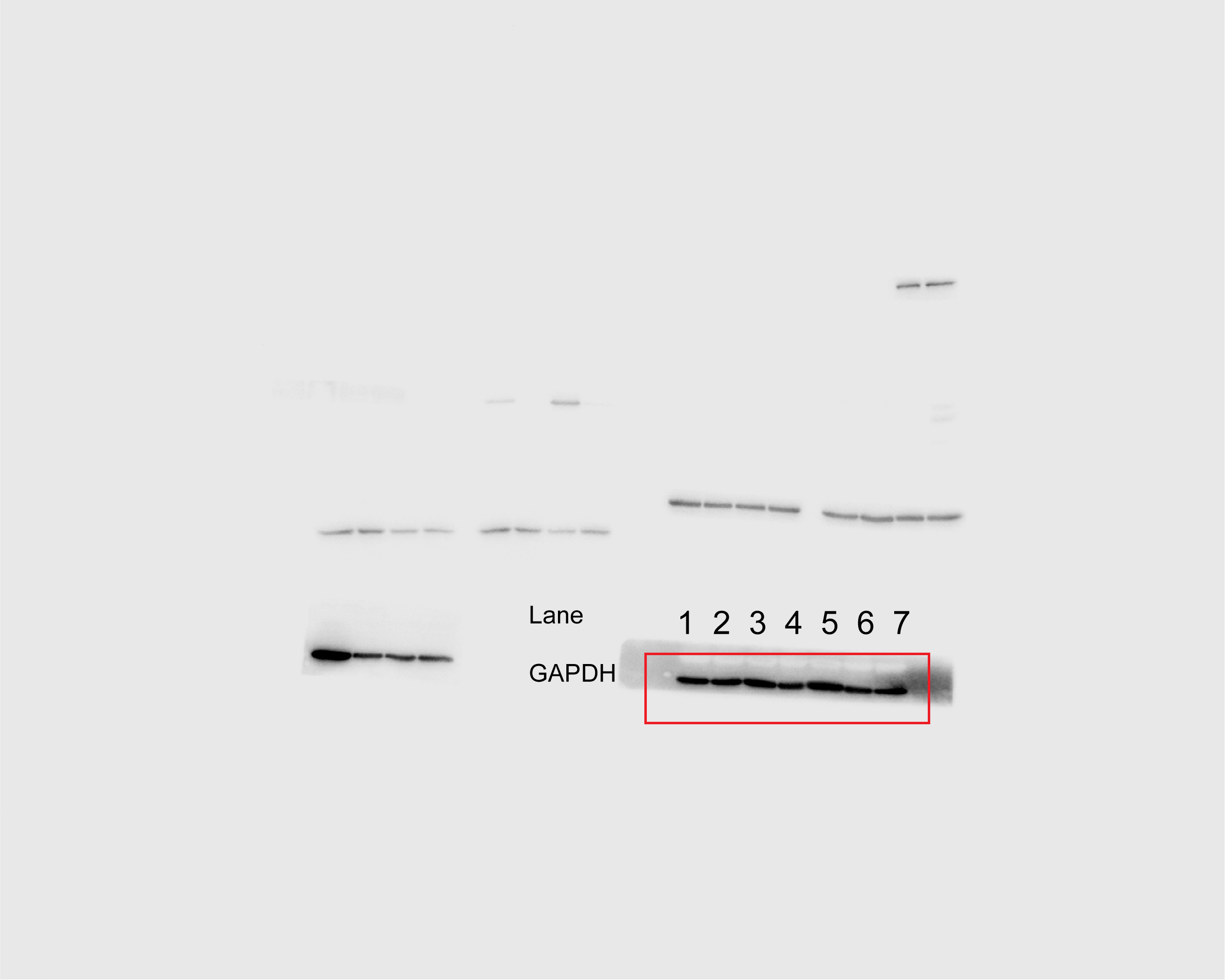

Supplement: Figure 4—source data 1. [file elife-101973-fig4-data1.zip › Figure 4-source data 1/Fig4A-labeled/Input GAPDH.tif]

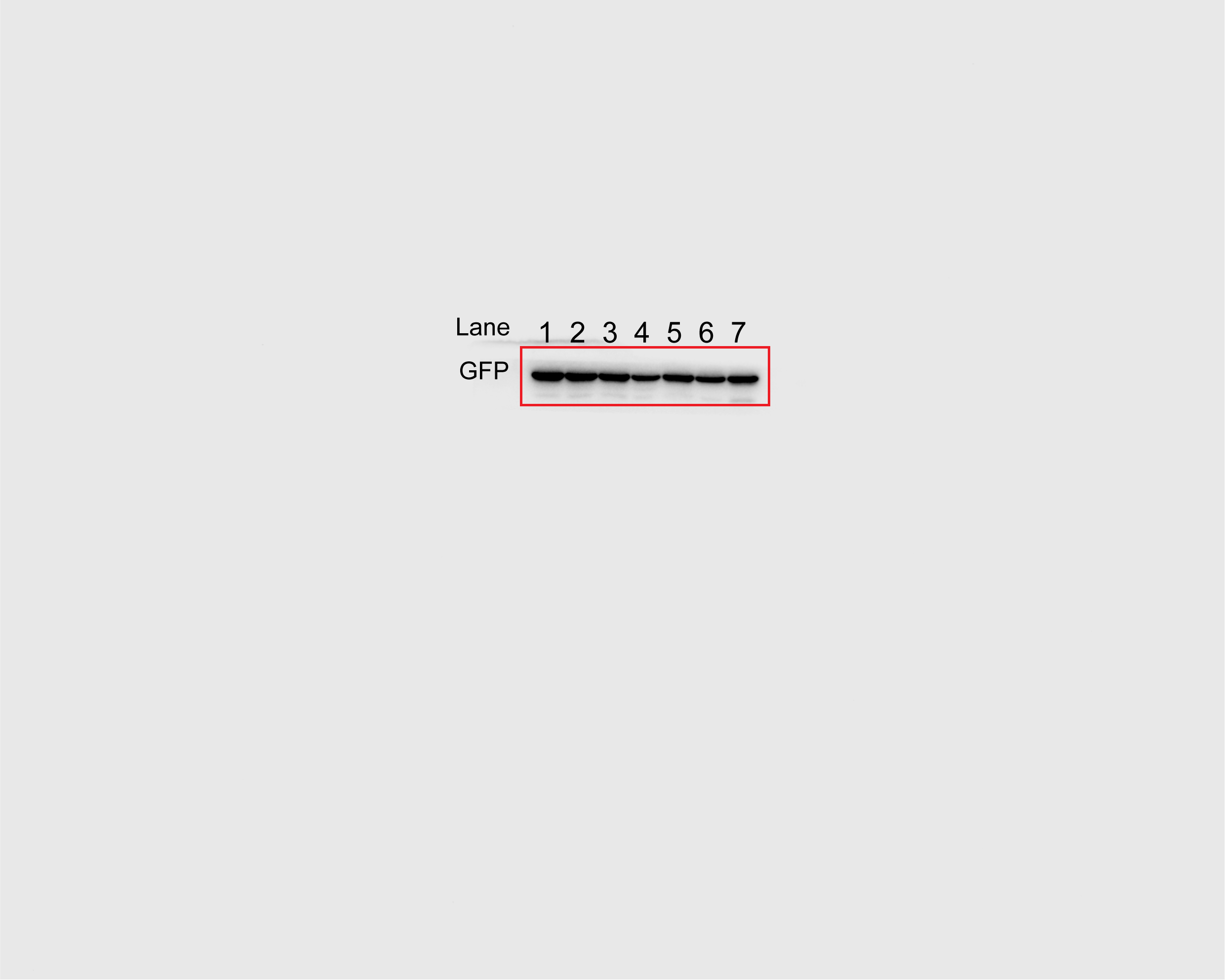

Supplement: Figure 4—source data 1. [file elife-101973-fig4-data1.zip › Figure 4-source data 1/Fig4A-labeled/input GFP.tif]

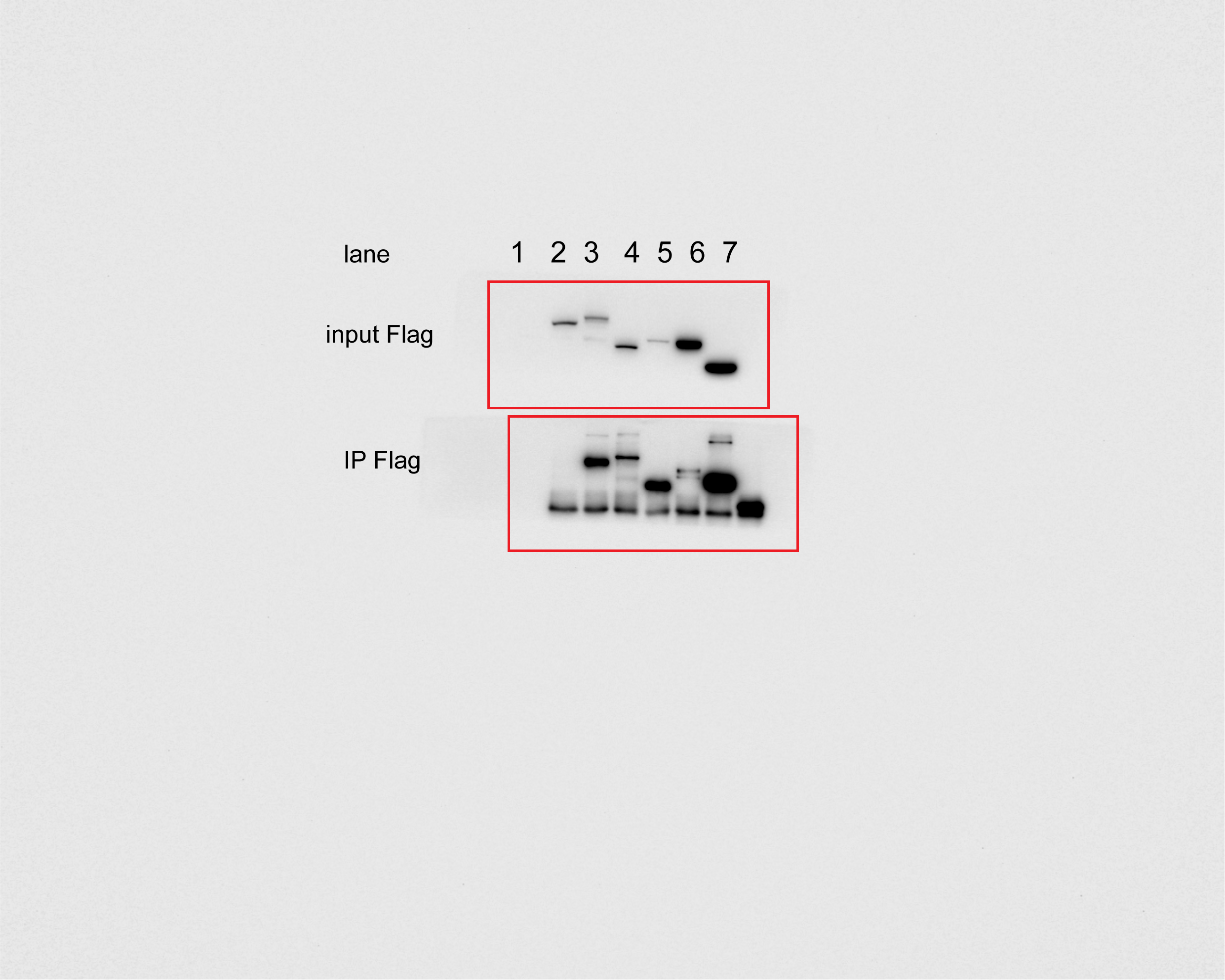

Supplement: Figure 4—source data 1. [file elife-101973-fig4-data1.zip › Figure 4-source data 1/Fig4A-labeled/input flag and IP flag.tif]

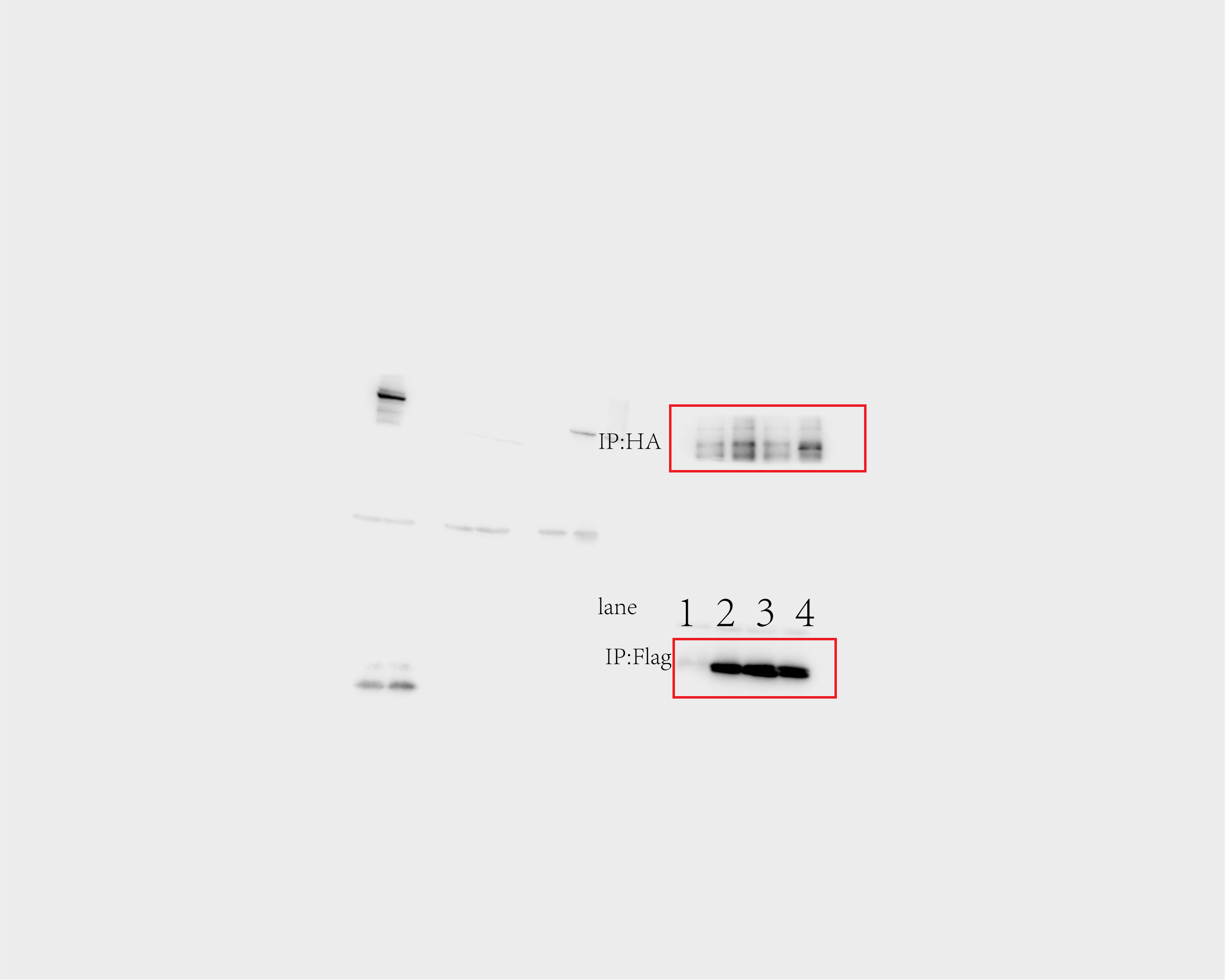

Supplement: Figure 4—source data 1. [file elife-101973-fig4-data1.zip › Figure 4-source data 1/Fig4B-labeled/IP Flag HA.tif]

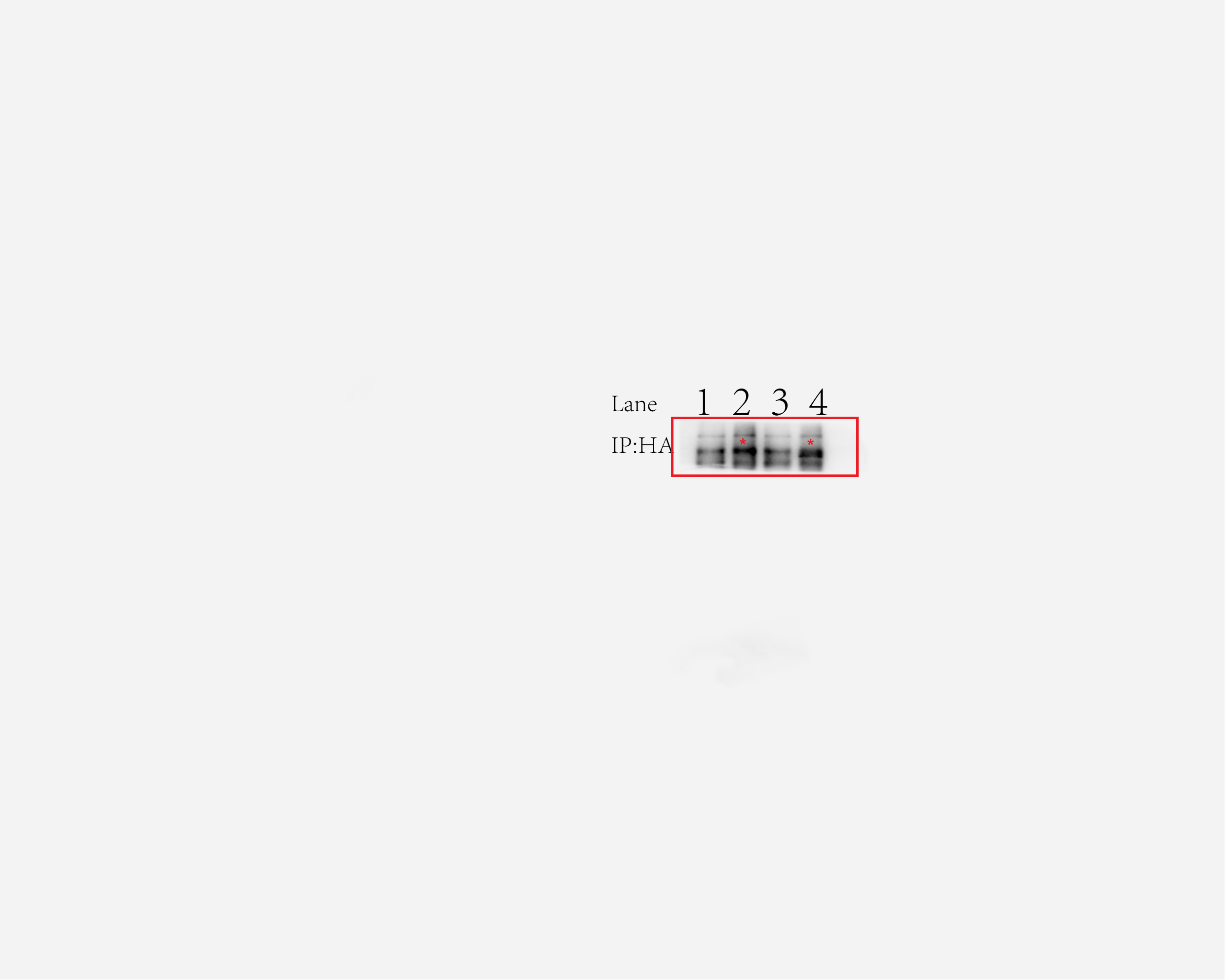

Supplement: Figure 4—source data 1. [file elife-101973-fig4-data1.zip › Figure 4-source data 1/Fig4B-labeled/IP HA long exposure.tif]

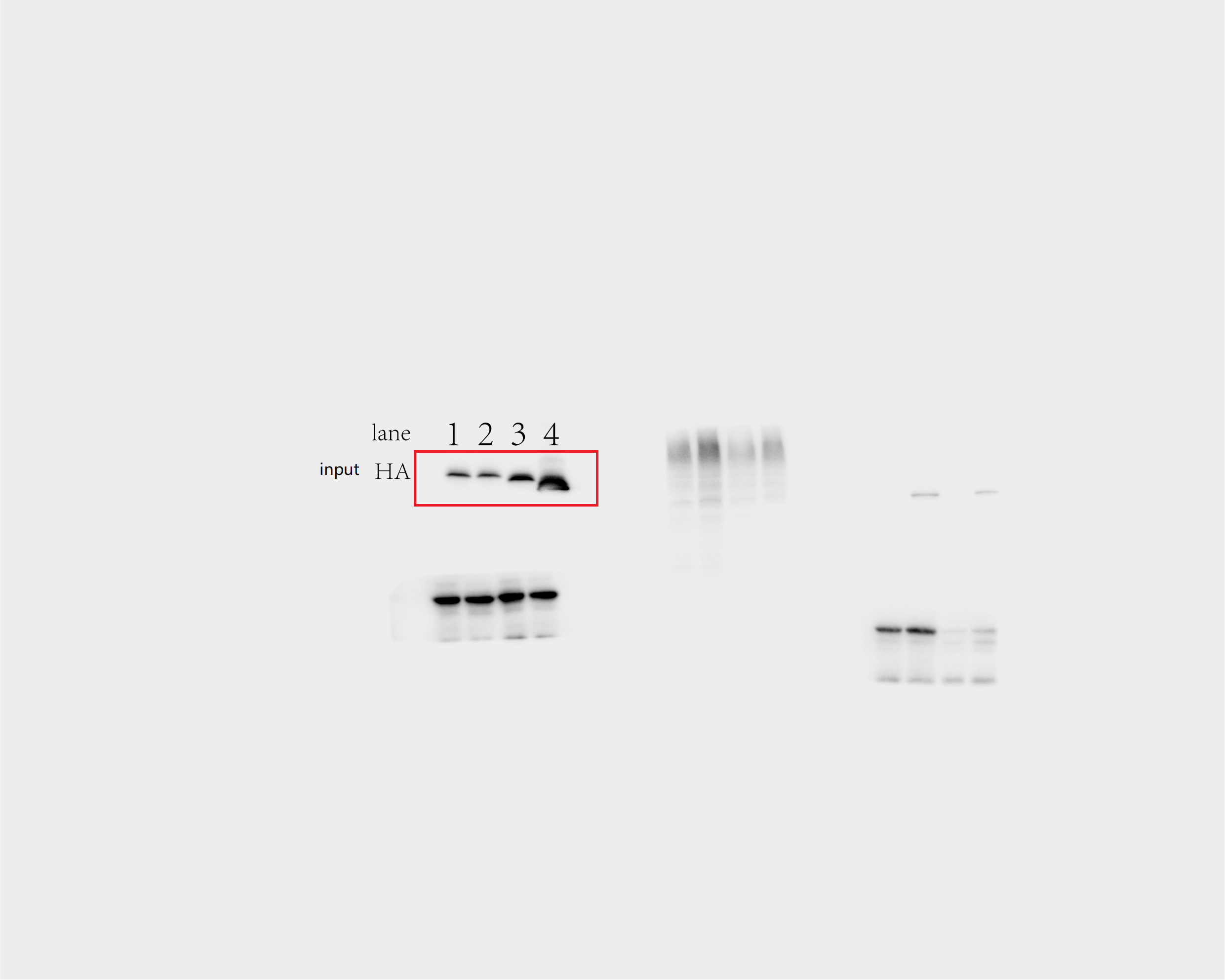

Supplement: Figure 4—source data 1. [file elife-101973-fig4-data1.zip › Figure 4-source data 1/Fig4B-labeled/input HA.tif]

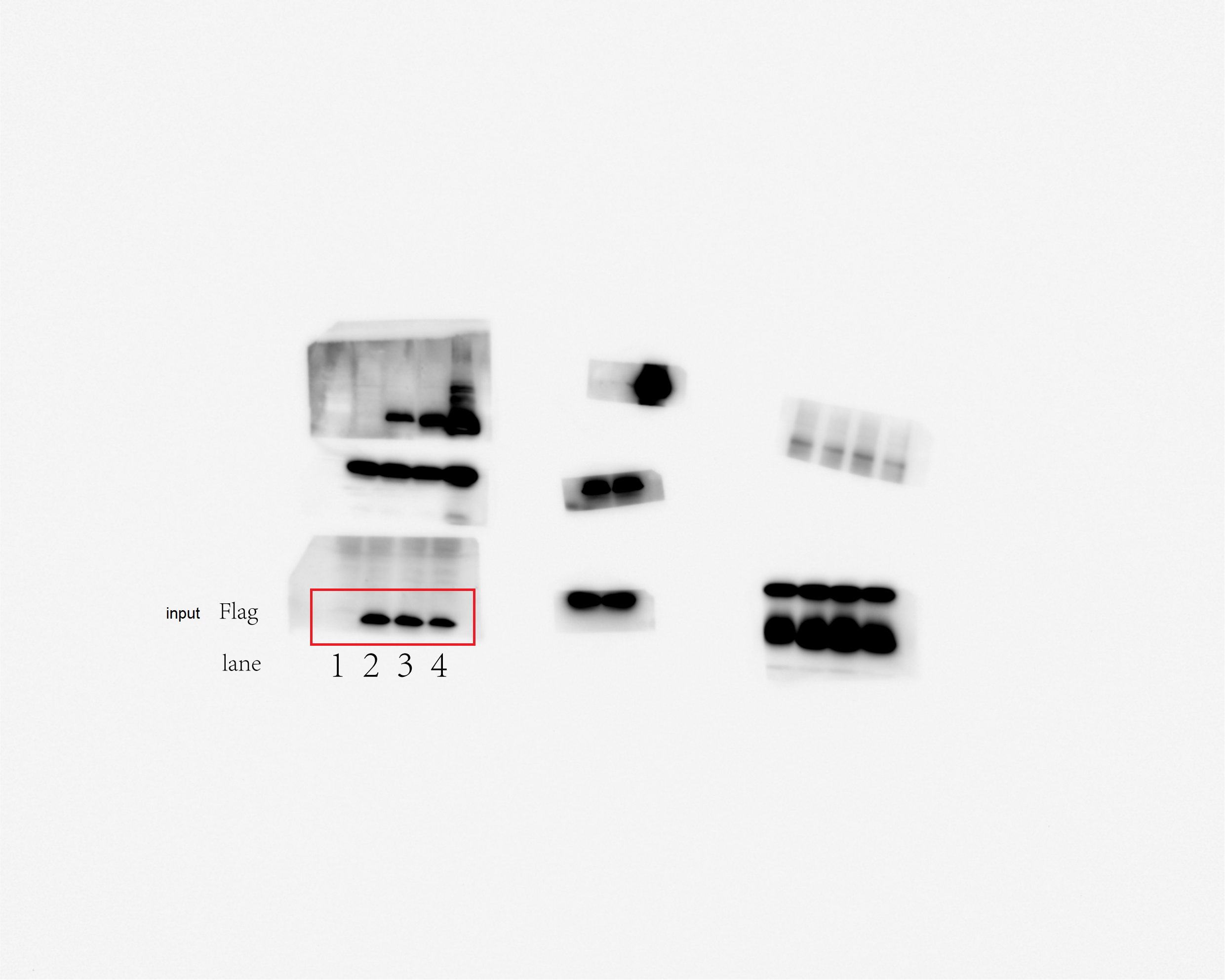

Supplement: Figure 4—source data 1. [file elife-101973-fig4-data1.zip › Figure 4-source data 1/Fig4B-labeled/input flag.tif]

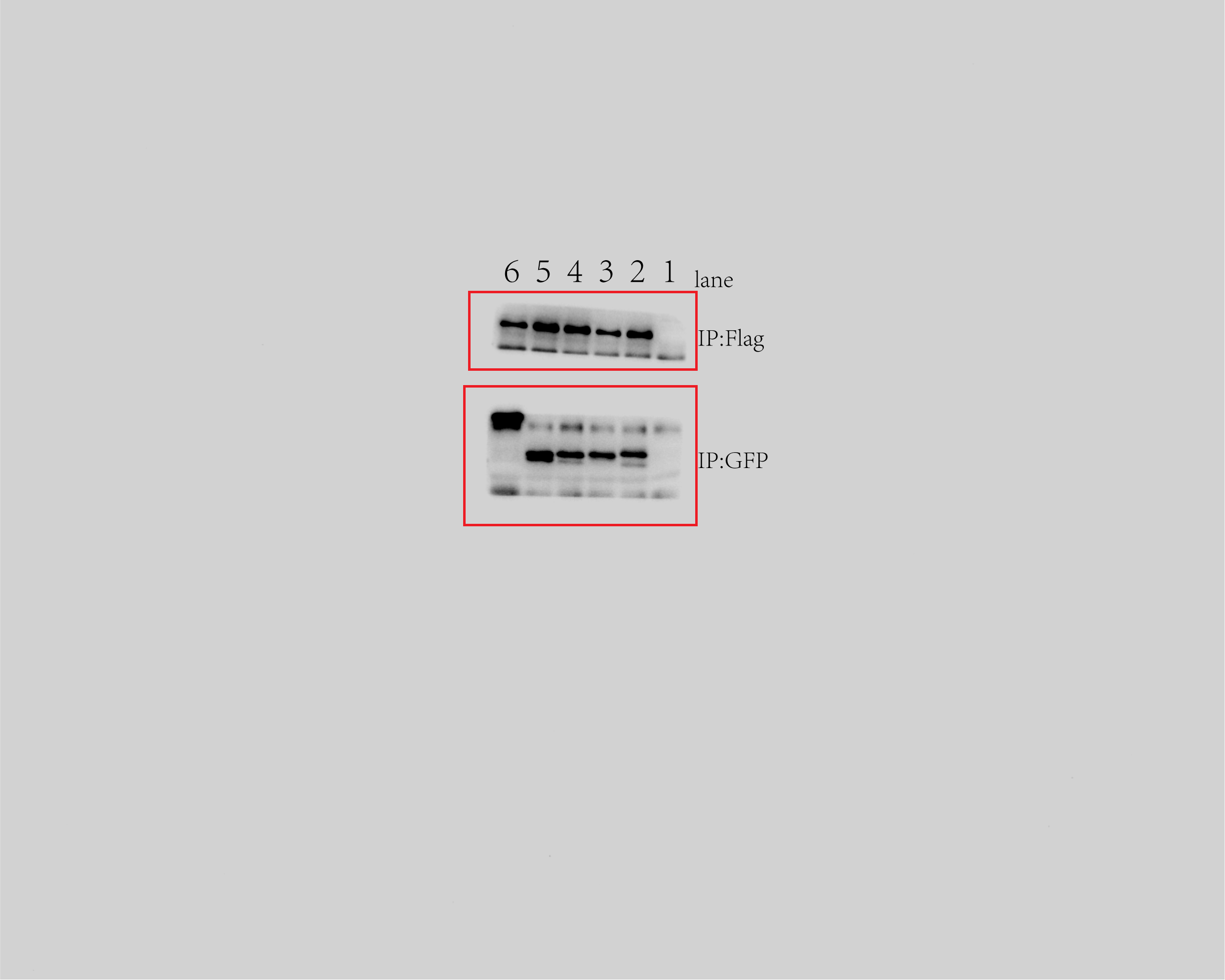

Supplement: Figure 4—source data 1. [file elife-101973-fig4-data1.zip › Figure 4-source data 1/Fig4C-labeled/IP flag and GFP.tif]

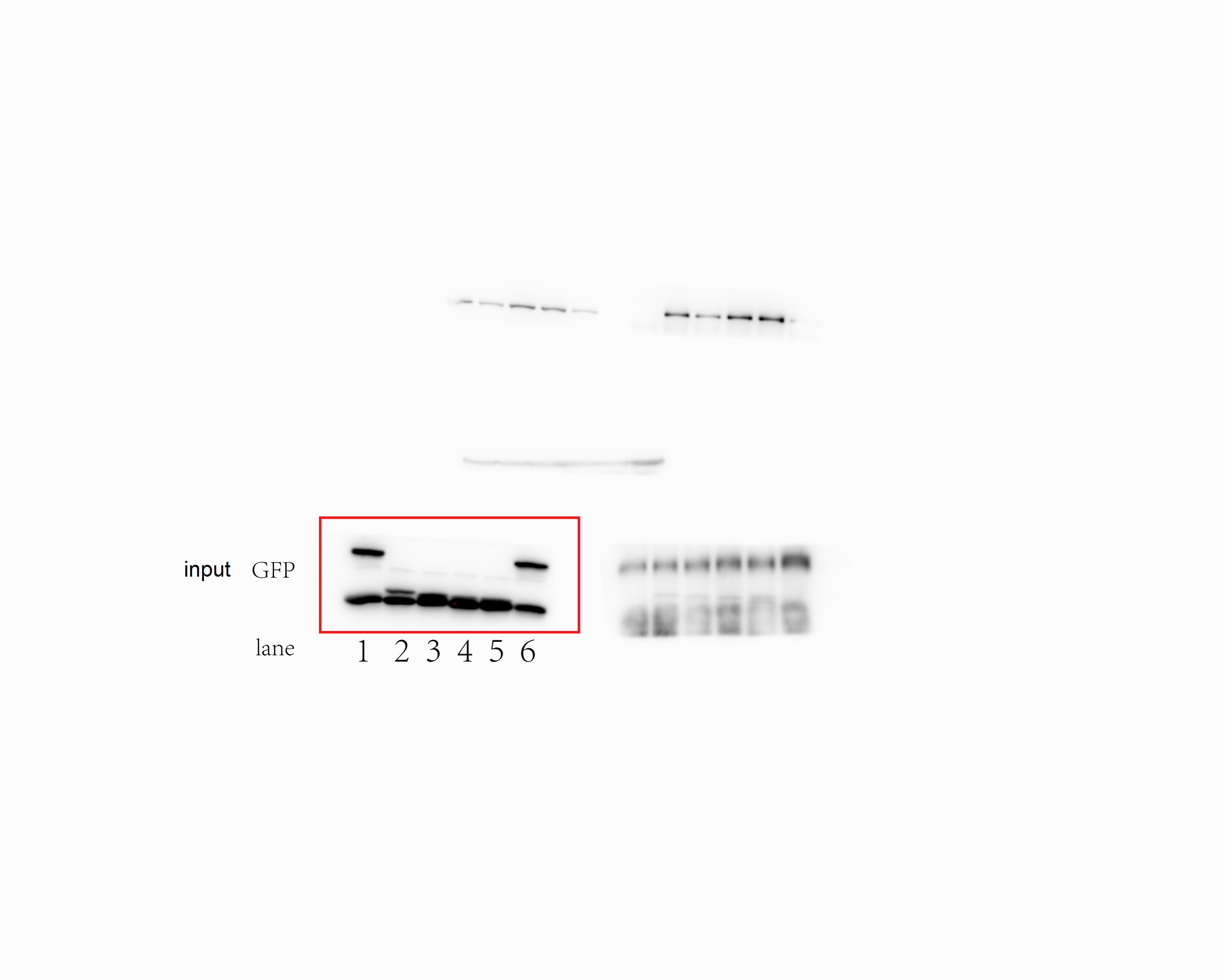

Supplement: Figure 4—source data 1. [file elife-101973-fig4-data1.zip › Figure 4-source data 1/Fig4C-labeled/input GFP.tif]

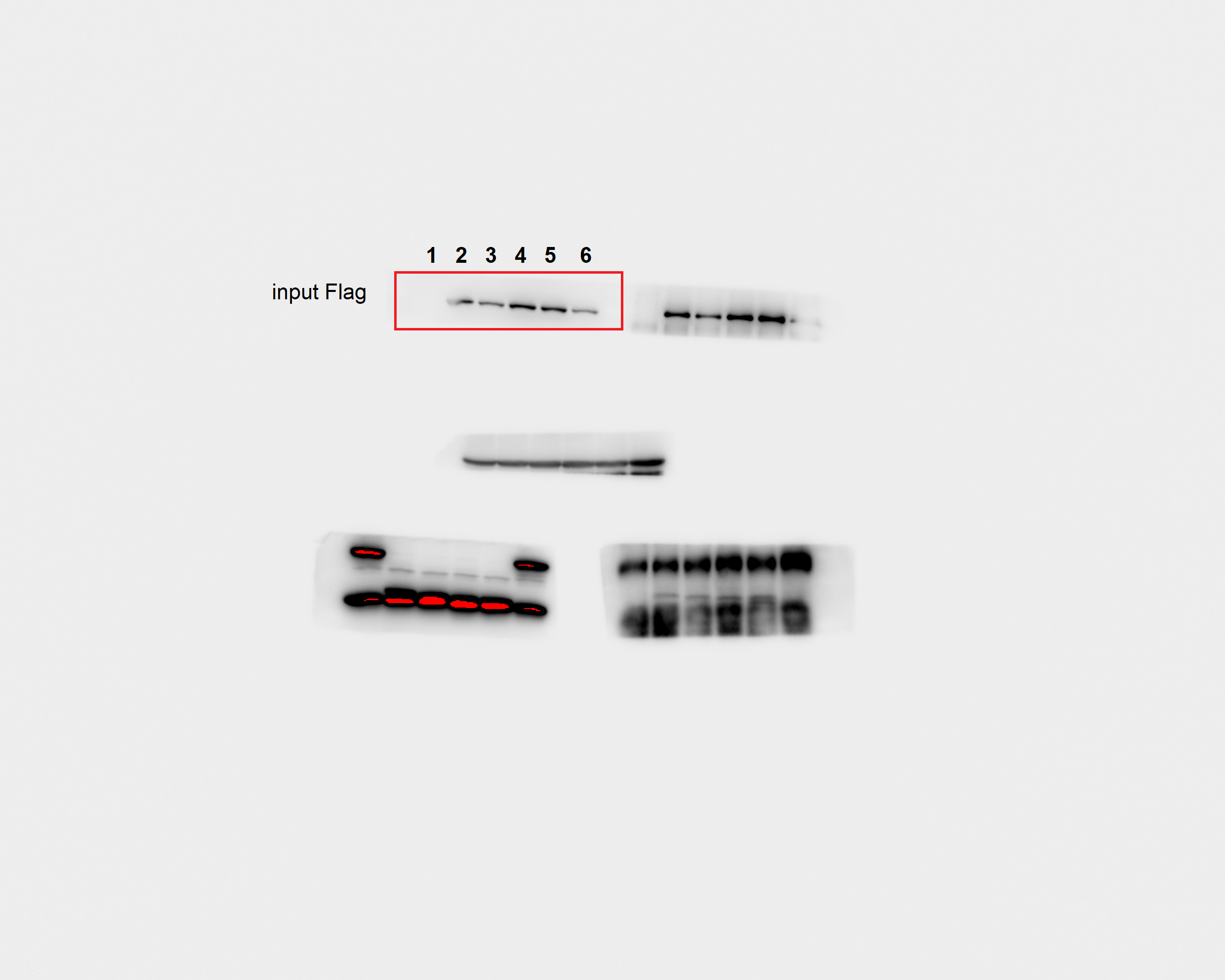

Supplement: Figure 4—source data 1. [file elife-101973-fig4-data1.zip › Figure 4-source data 1/Fig4C-labeled/input flag.tif]

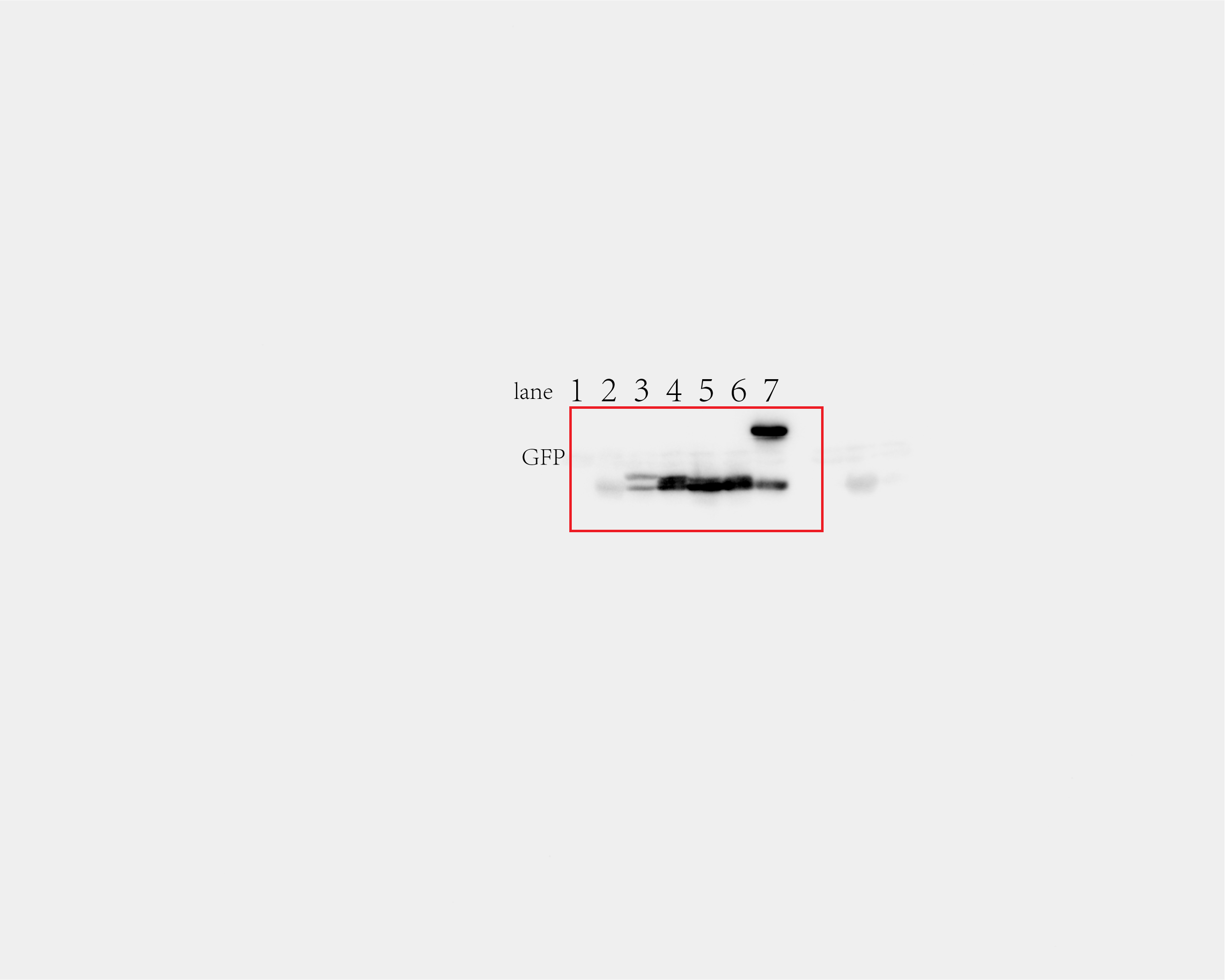

Supplement: Figure 4—source data 1. [file elife-101973-fig4-data1.zip › Figure 4-source data 1/Fig4E-labeled/GFP.tif]

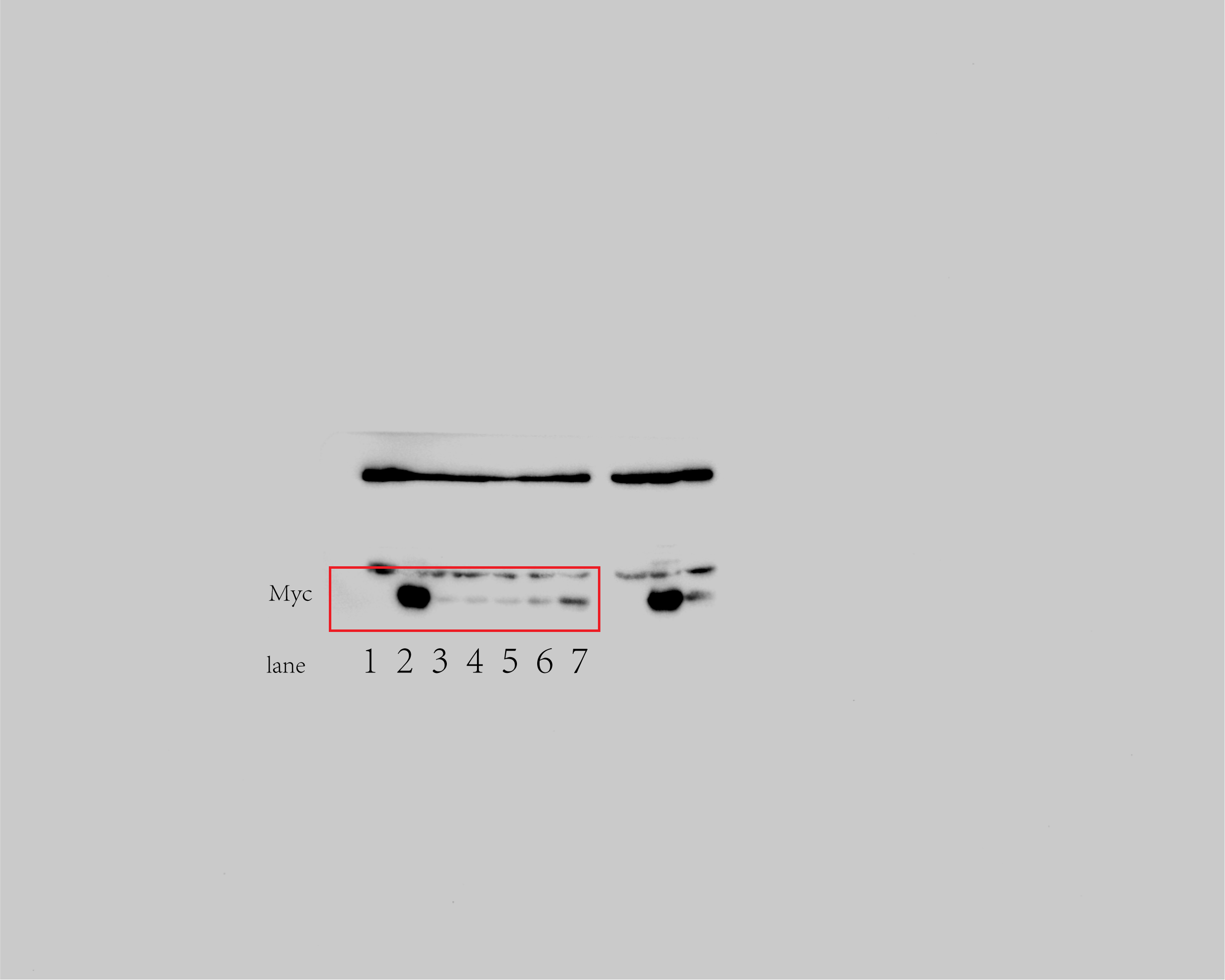

Supplement: Figure 4—source data 1. [file elife-101973-fig4-data1.zip › Figure 4-source data 1/Fig4E-labeled/Myc.tif]

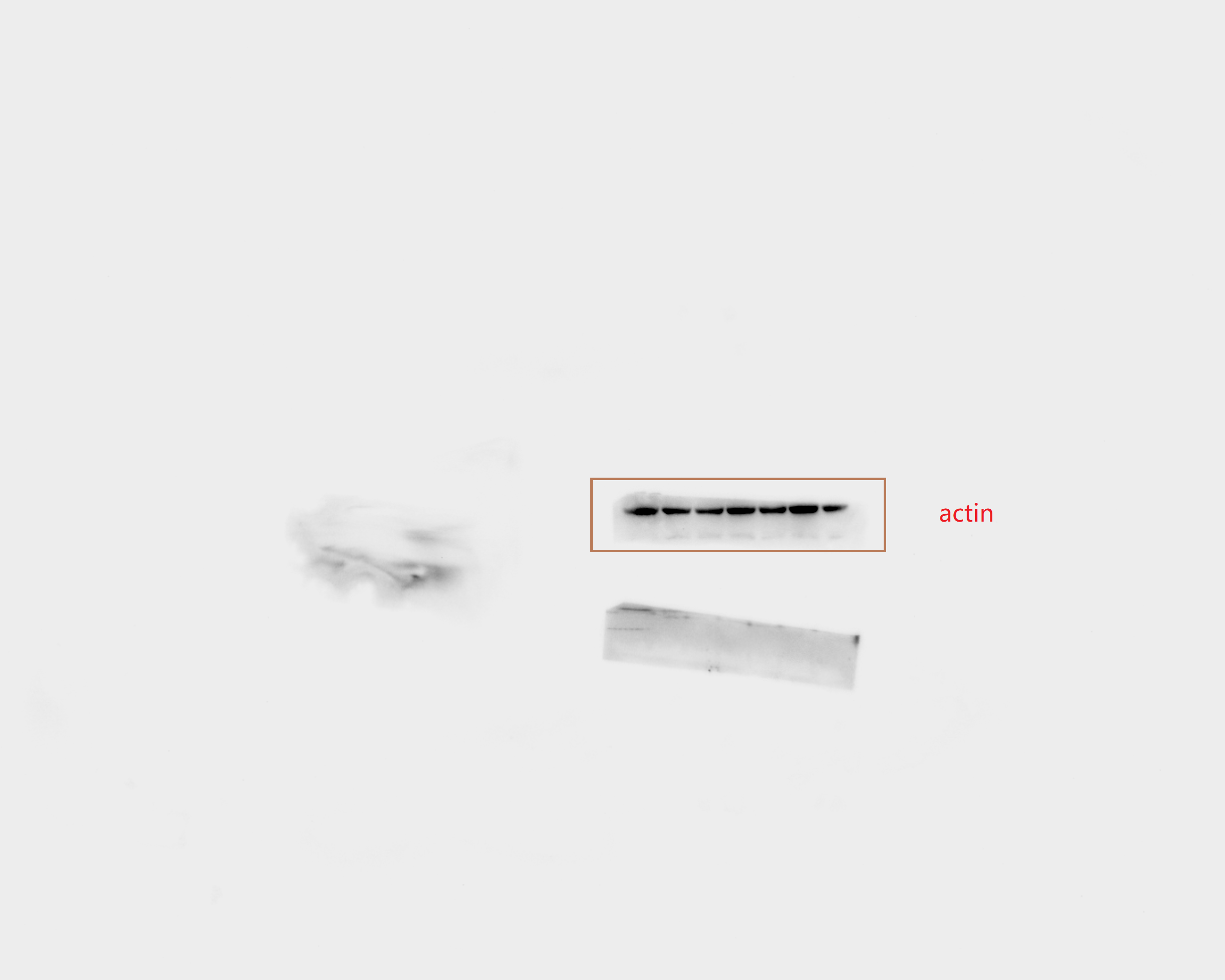

Supplement: Figure 4—source data 1. [file elife-101973-fig4-data1.zip › Figure 4-source data 1/Fig4E-labeled/actin.tif]

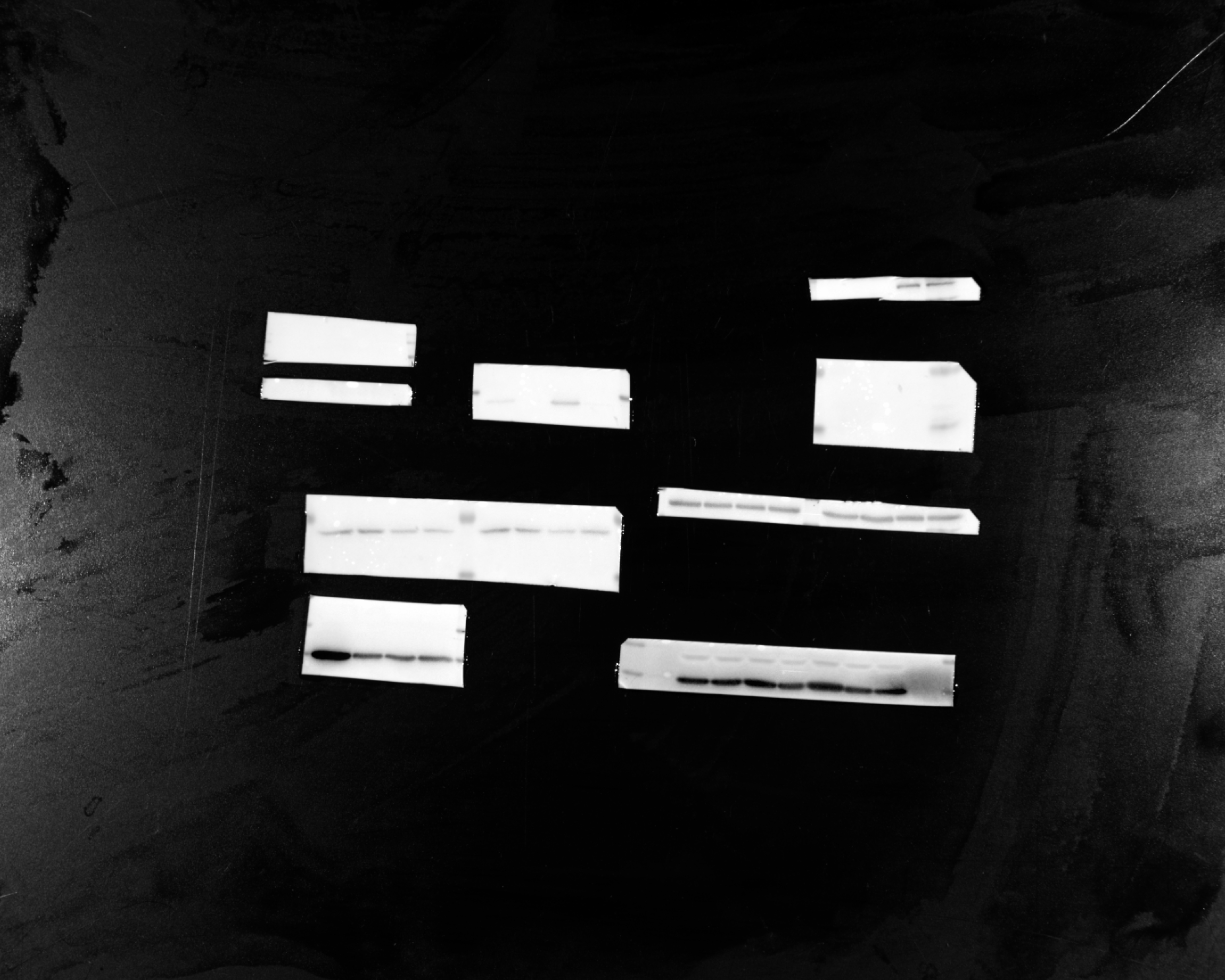

Supplement: Figure 4—source data 2. [file elife-101973-fig4-data2.zip › Figure 4-source data 2/figure 4A/input GAPDH.jpg]

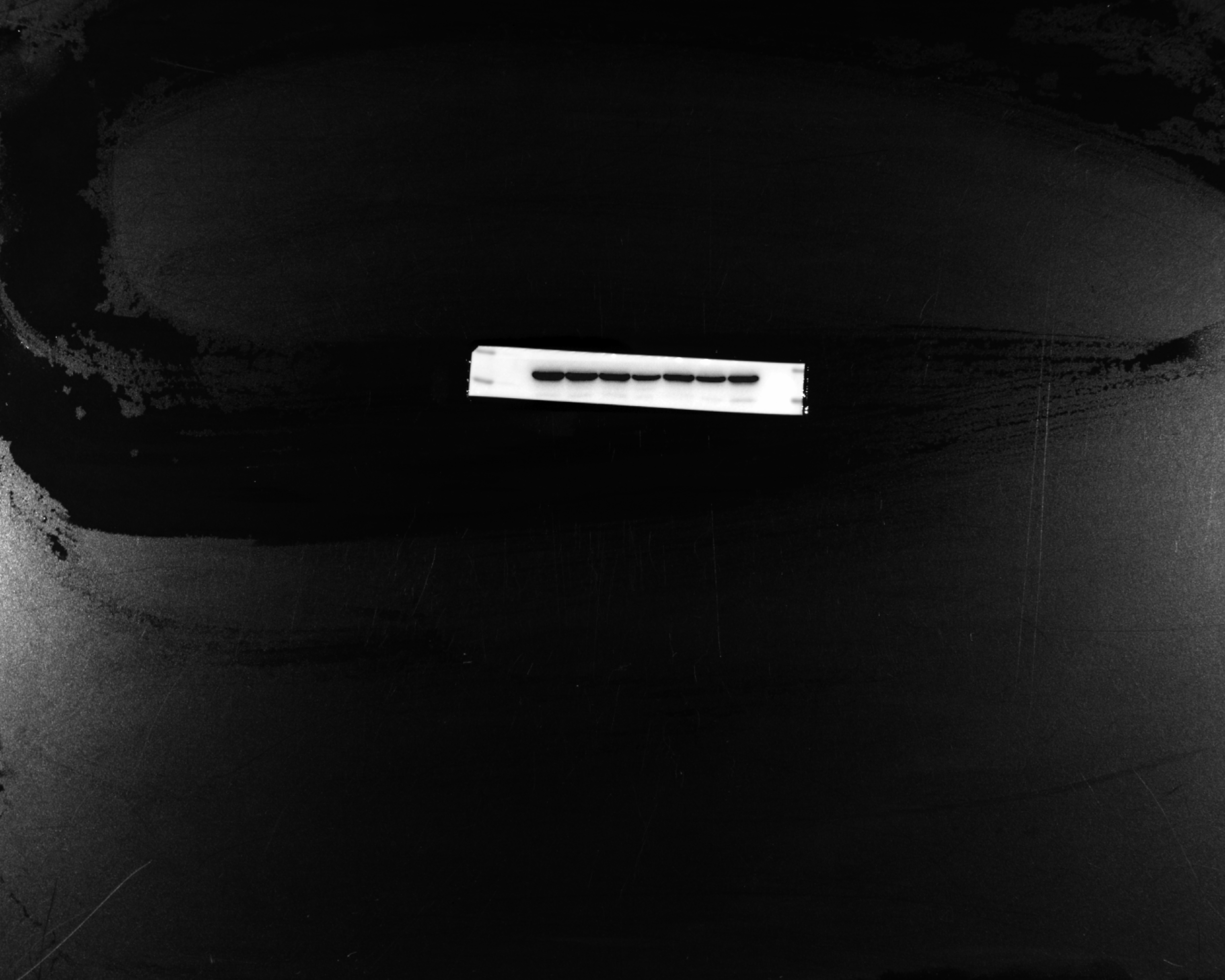

Supplement: Figure 4—source data 2. [file elife-101973-fig4-data2.zip › Figure 4-source data 2/figure 4A/input GFP.jpg]

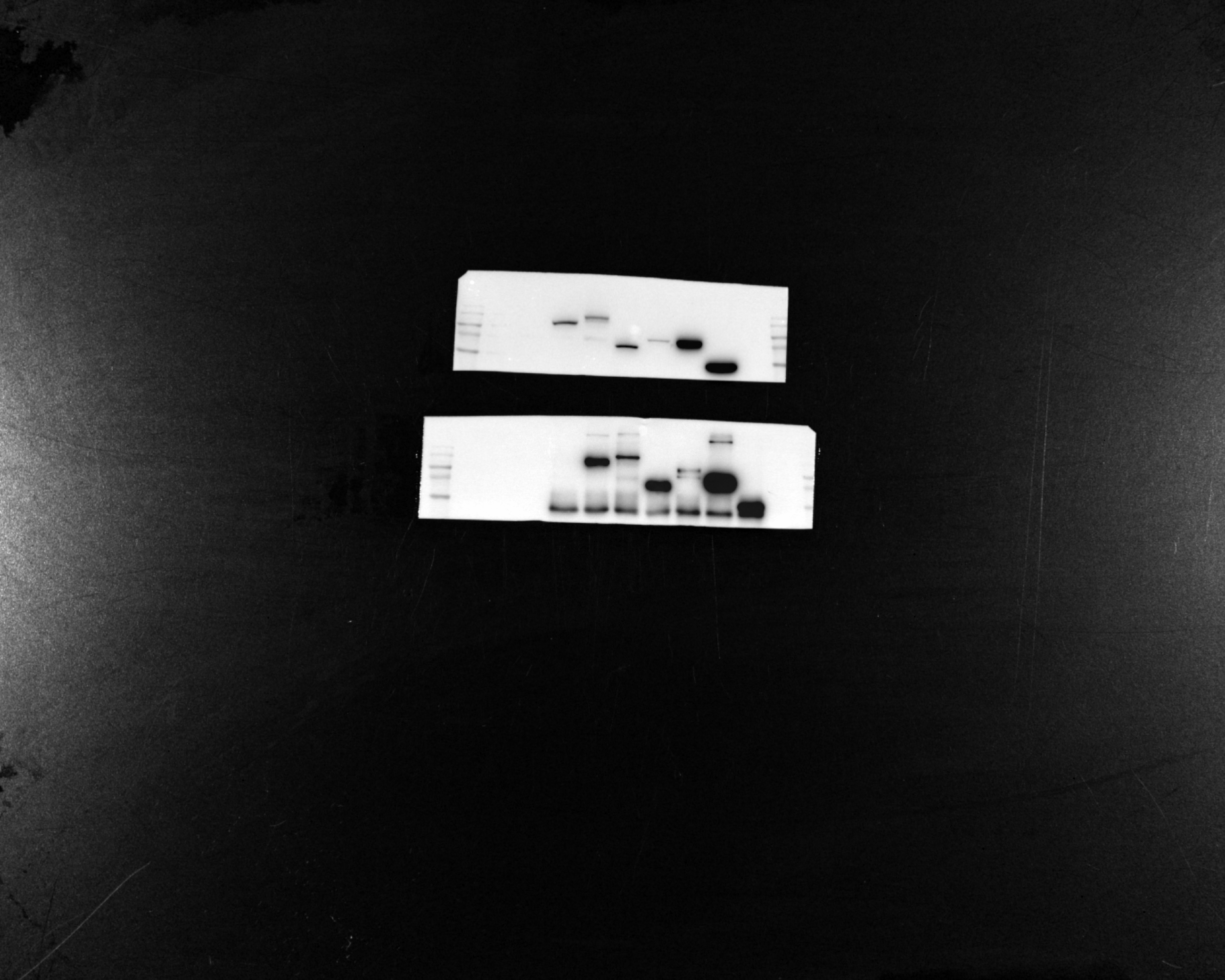

Supplement: Figure 4—source data 2. [file elife-101973-fig4-data2.zip › Figure 4-source data 2/figure 4A/input flag and IP flag.jpg]

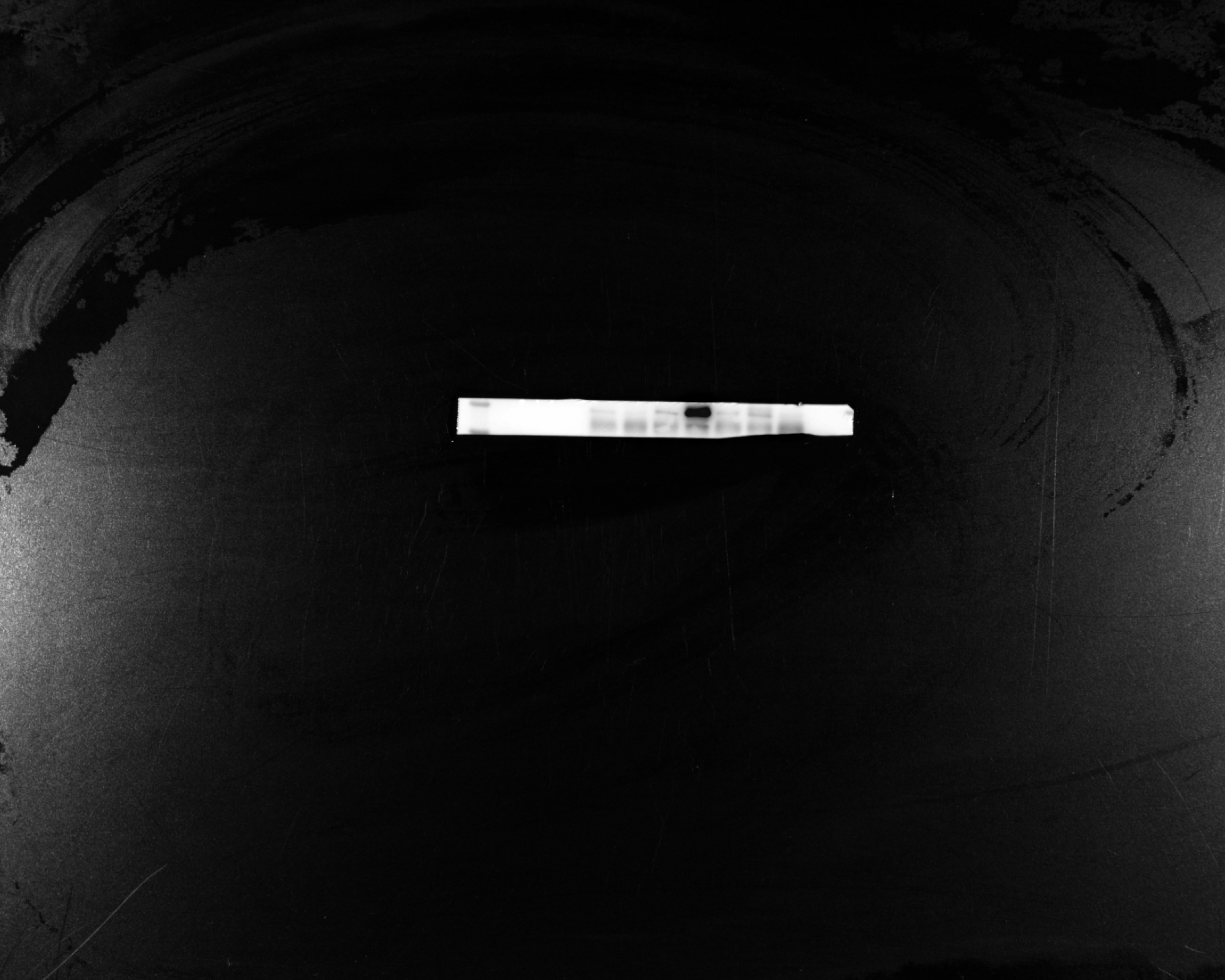

Supplement: Figure 4—source data 2. [file elife-101973-fig4-data2.zip › Figure 4-source data 2/figure 4A/ip GFP.jpg]

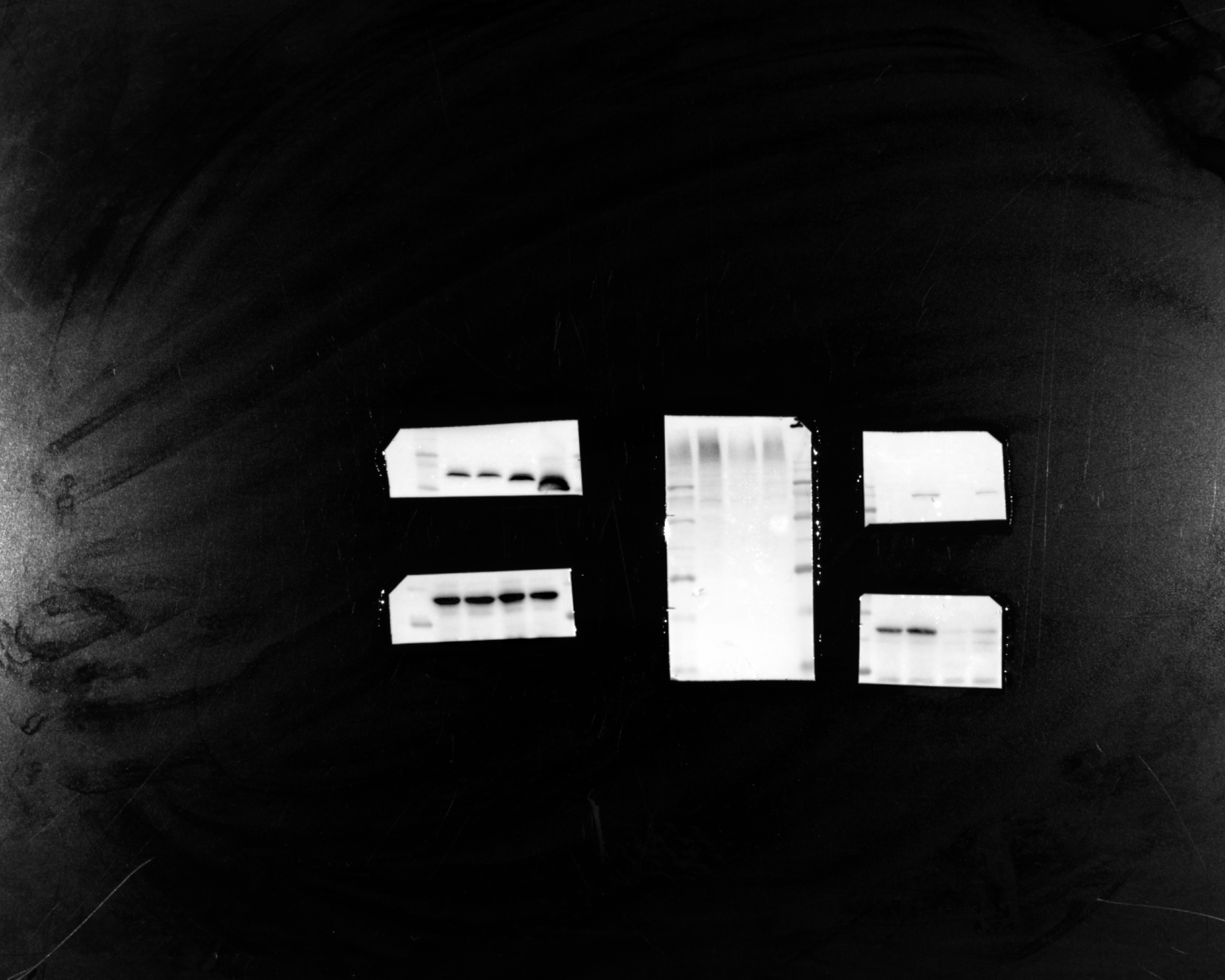

Supplement: Figure 4—source data 2. [file elife-101973-fig4-data2.zip › Figure 4-source data 2/figure 4B/input HA.jpg]

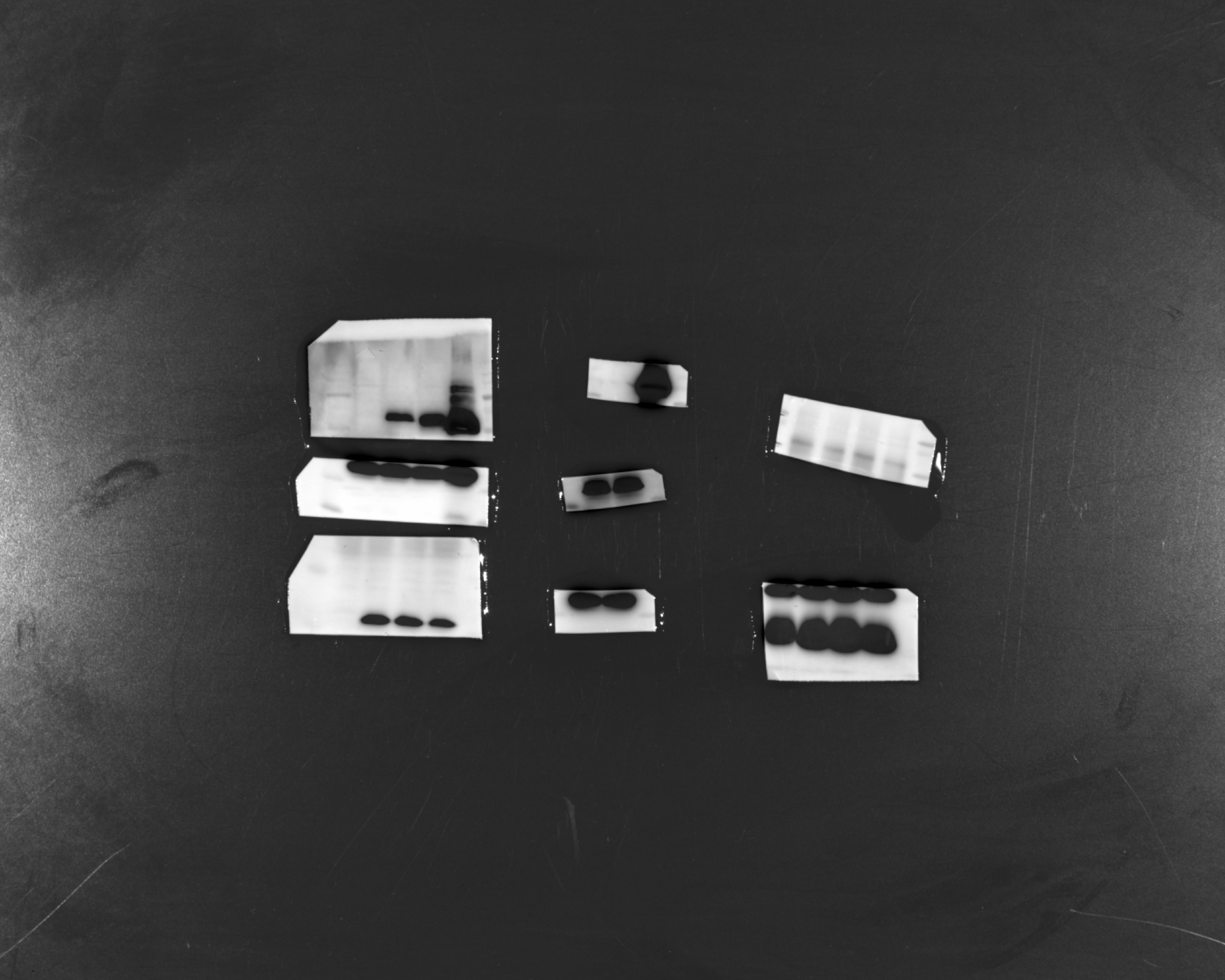

Supplement: Figure 4—source data 2. [file elife-101973-fig4-data2.zip › Figure 4-source data 2/figure 4B/input flag.jpg]

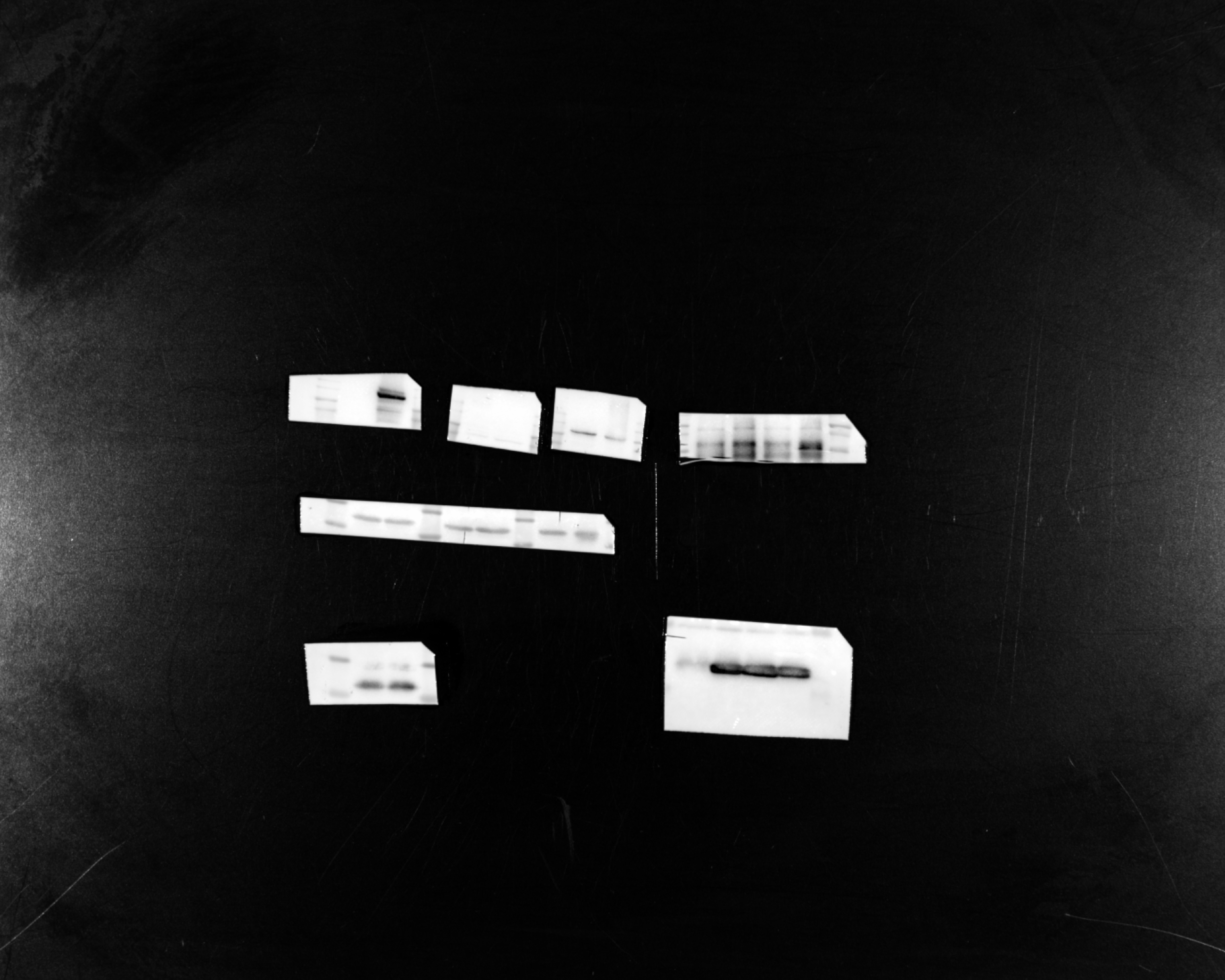

Supplement: Figure 4—source data 2. [file elife-101973-fig4-data2.zip › Figure 4-source data 2/figure 4B/ip flag HA.jpg]

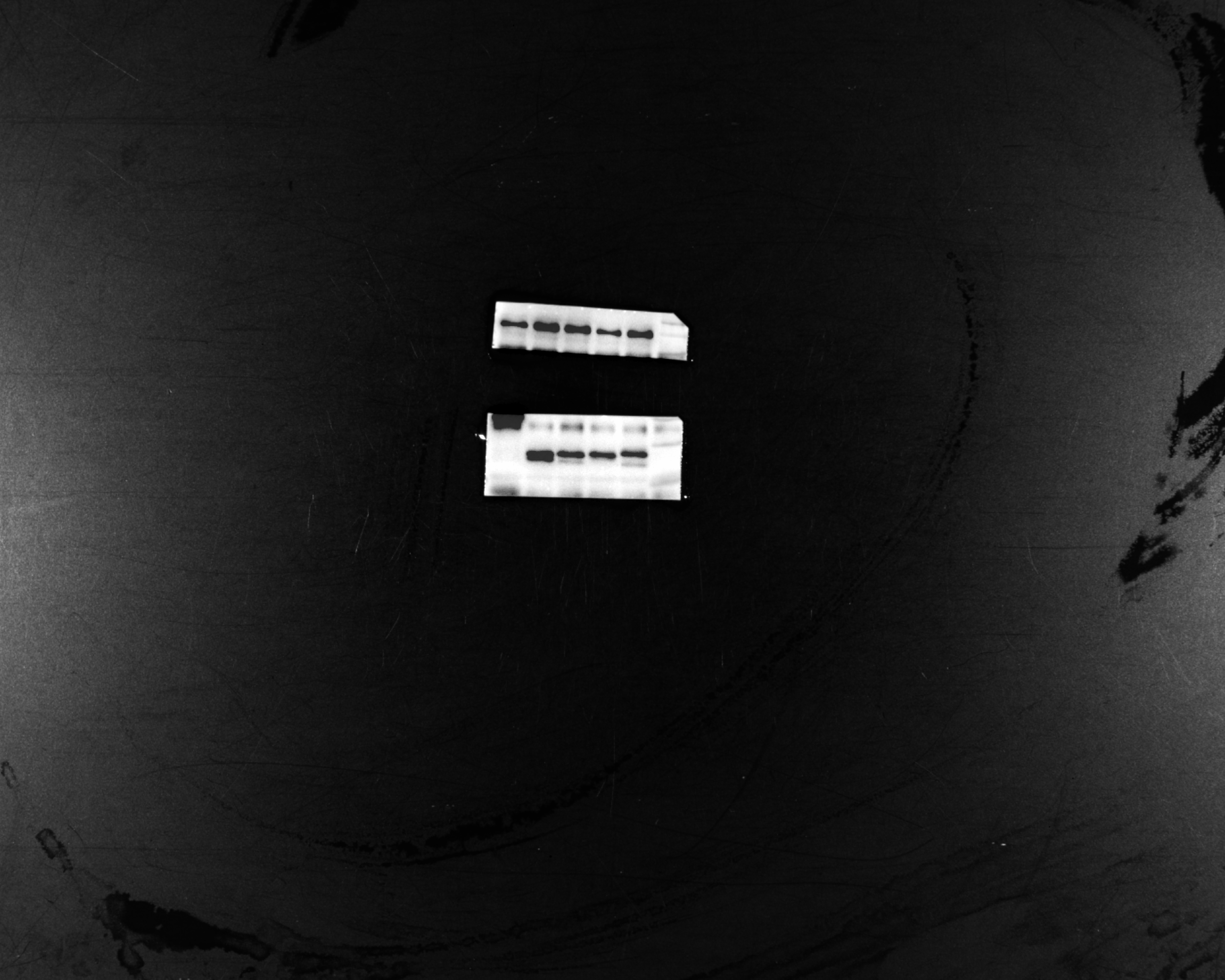

Supplement: Figure 4—source data 2. [file elife-101973-fig4-data2.zip › Figure 4-source data 2/figure 4C/IP flag and GFP.jpg]

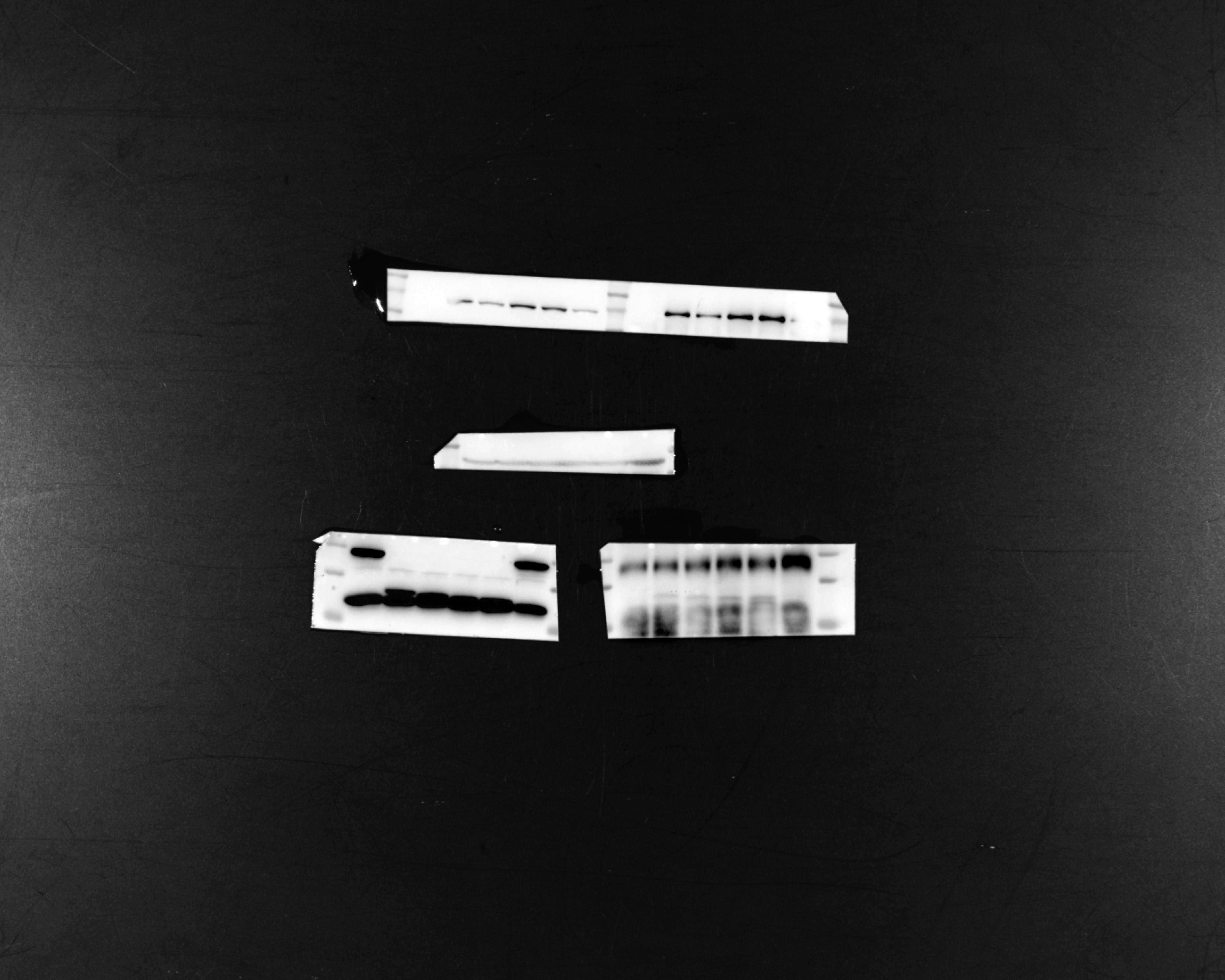

Supplement: Figure 4—source data 2. [file elife-101973-fig4-data2.zip › Figure 4-source data 2/figure 4C/input flag and GFP.jpg]

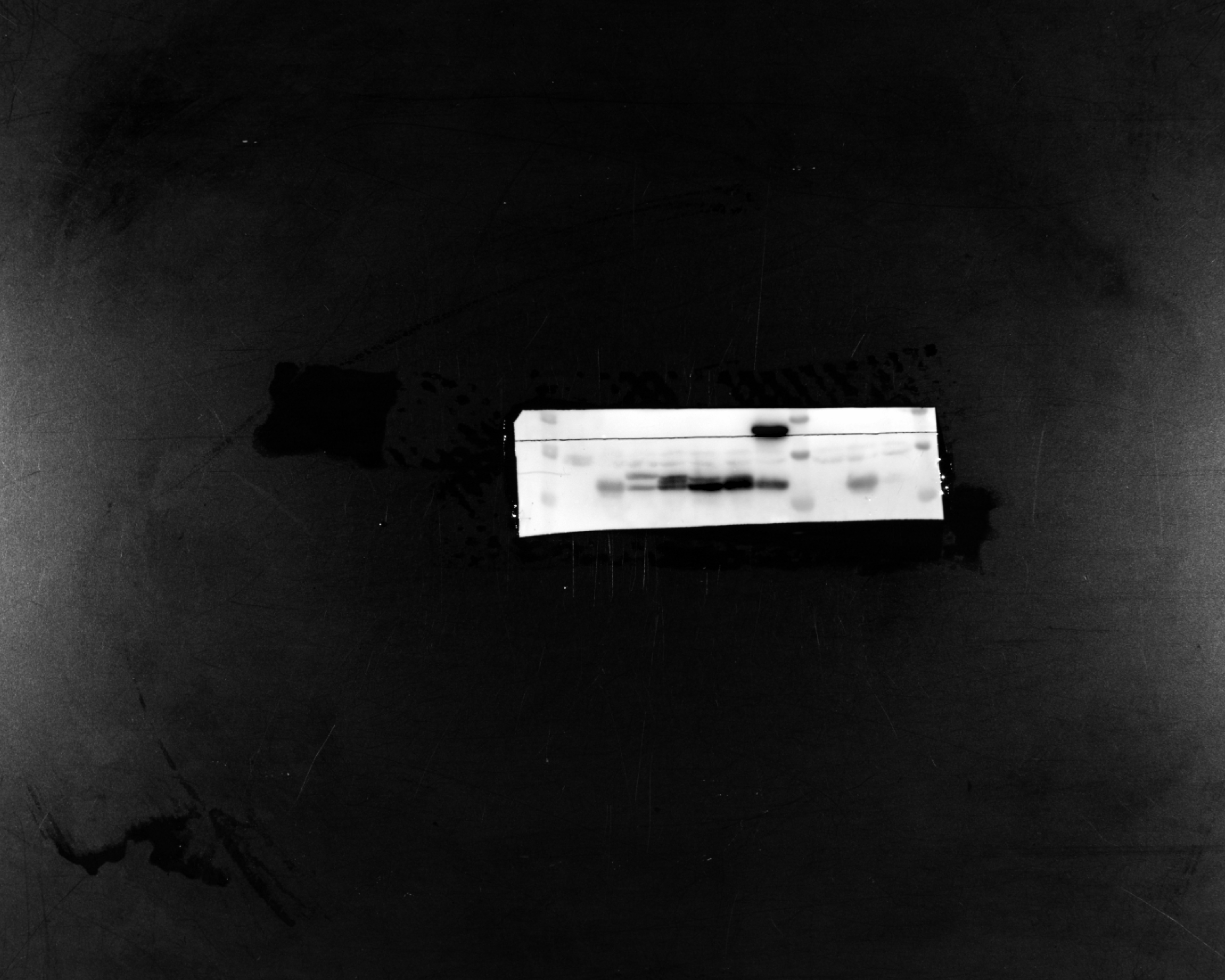

Supplement: Figure 4—source data 2. [file elife-101973-fig4-data2.zip › Figure 4-source data 2/figure 4E/GFP.jpg]

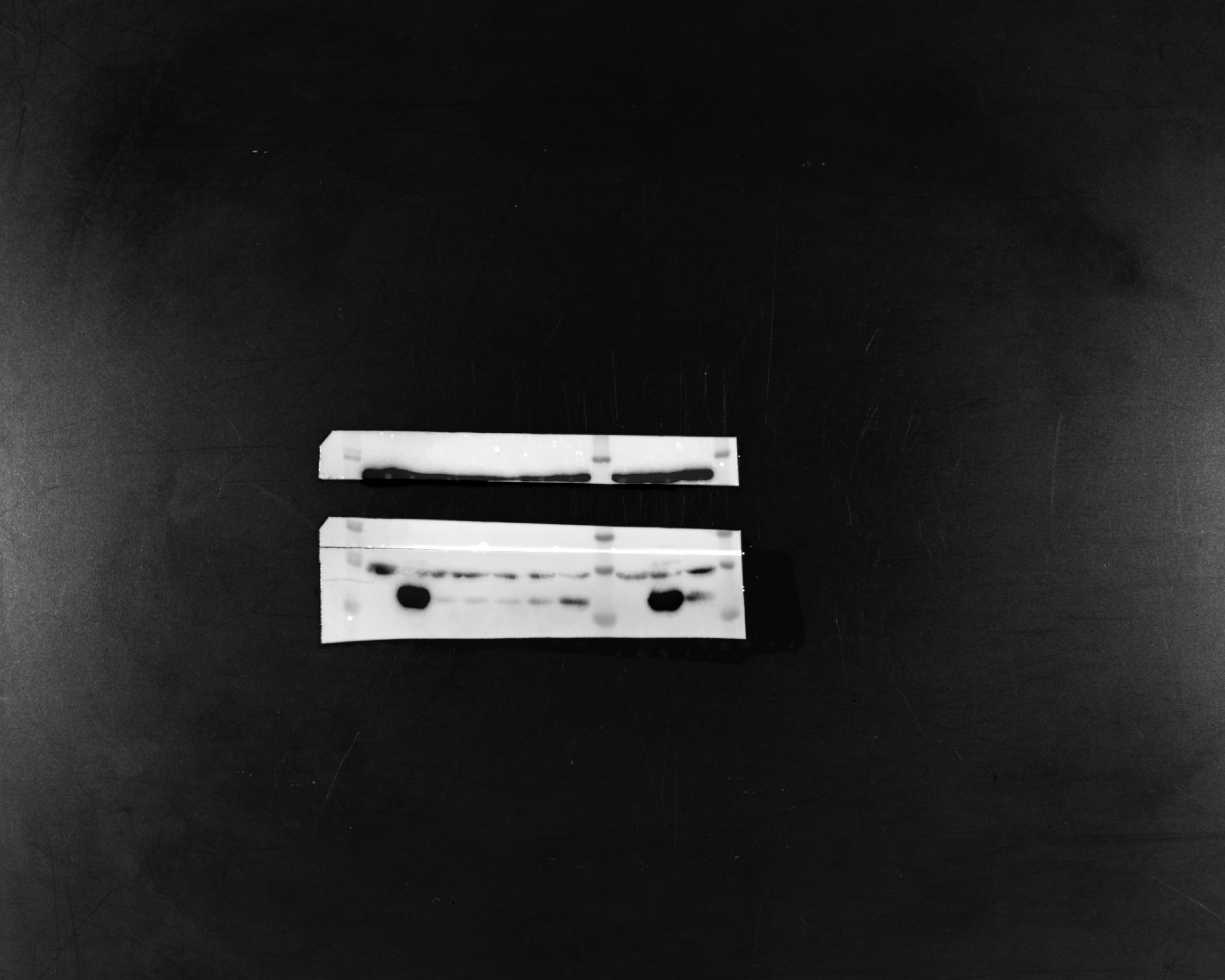

Supplement: Figure 4—source data 2. [file elife-101973-fig4-data2.zip › Figure 4-source data 2/figure 4E/Myc.jpg]

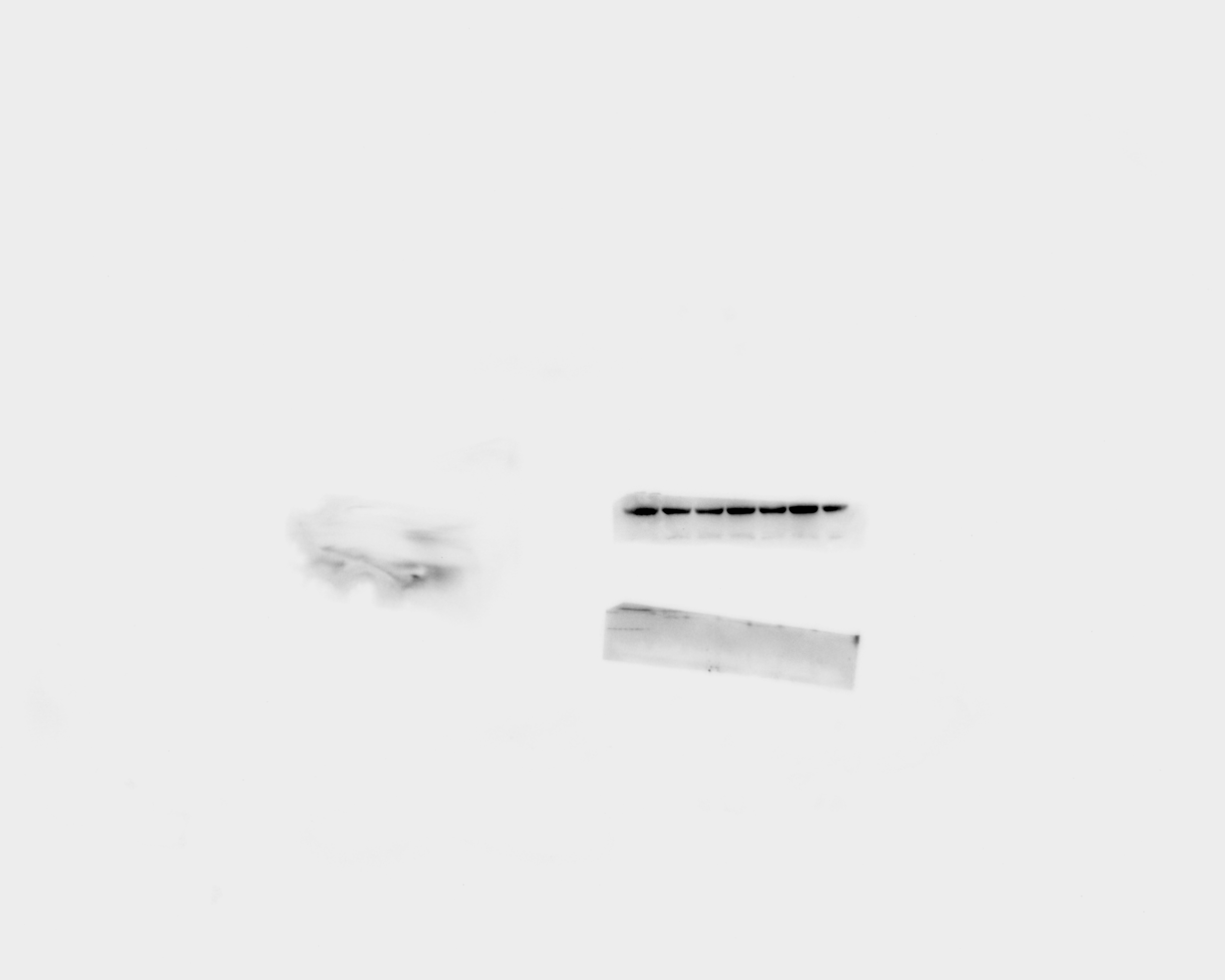

Supplement: Figure 4—source data 2. [file elife-101973-fig4-data2.zip › Figure 4-source data 2/figure 4E/actin.jpg]

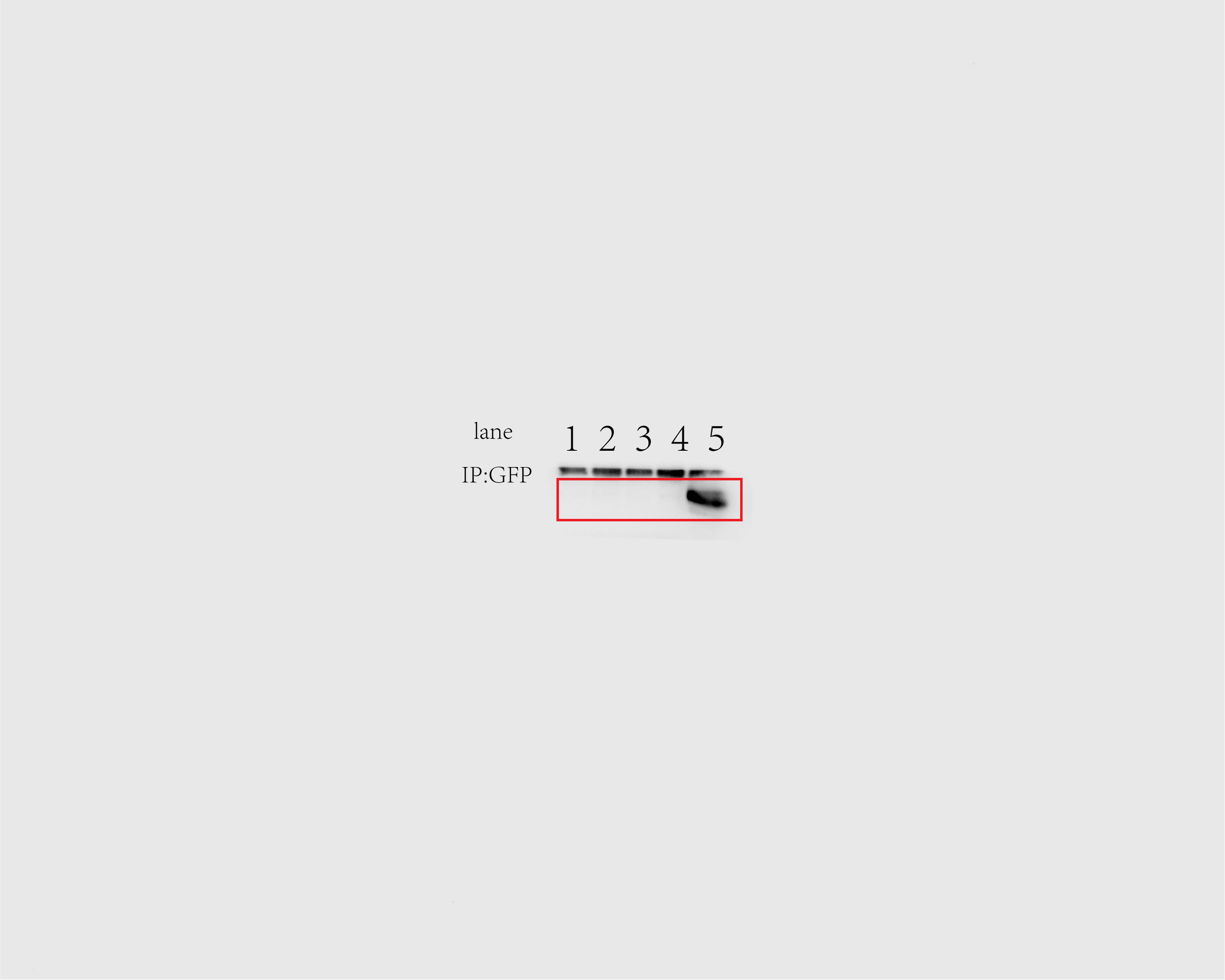

Supplement: Figure 4—figure supplement 1—source data 1. [file elife-101973-fig4-figsupp1-data1.zip › Figure 4–figure supplement 1–source data 1/Figure 4–figure supplement 1A-labeled/IP GFP.tif]

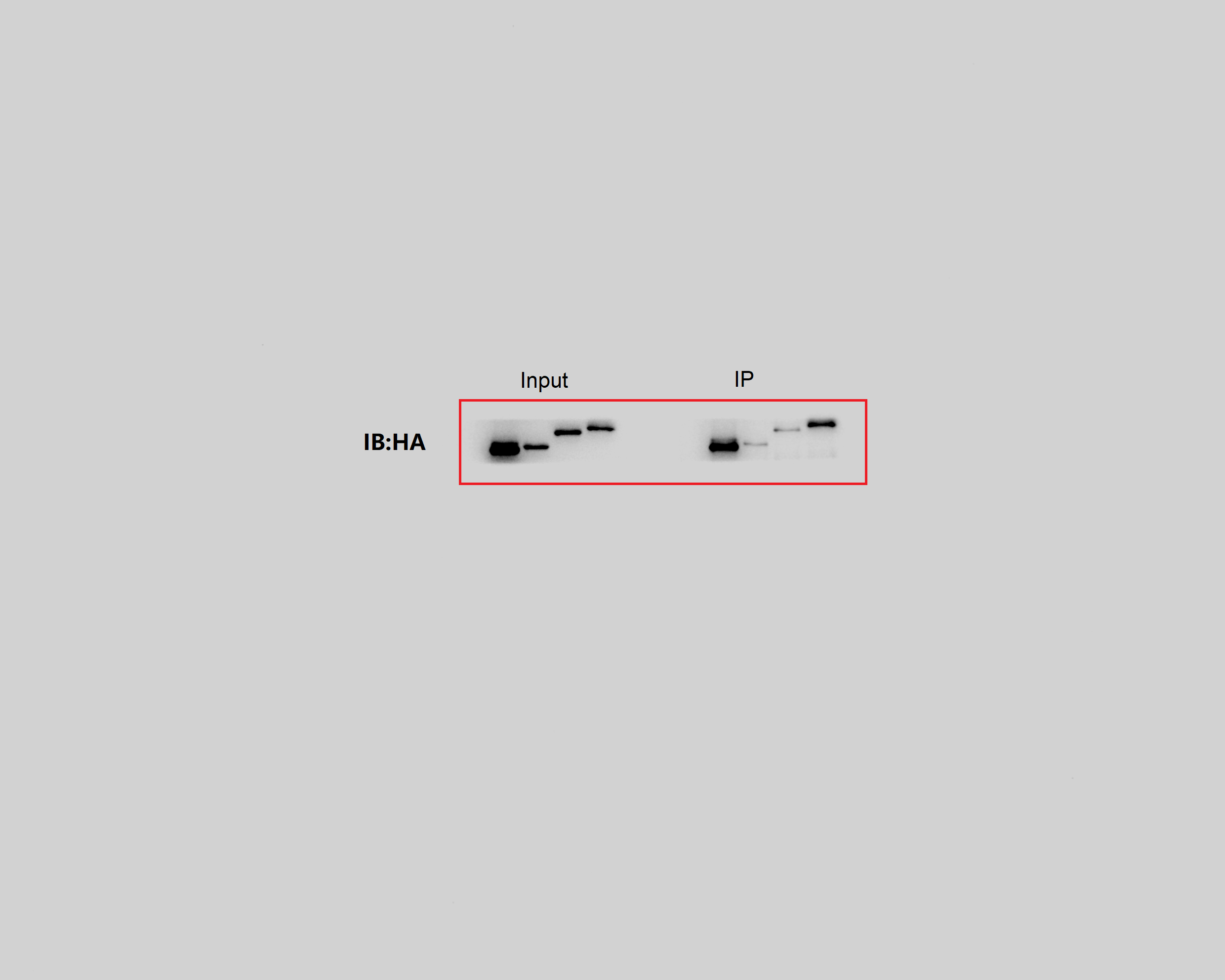

Supplement: Figure 4—figure supplement 1—source data 1. [file elife-101973-fig4-figsupp1-data1.zip › Figure 4–figure supplement 1–source data 1/Figure 4–figure supplement 1A-labeled/IP HA and input HA short exposure.tif]

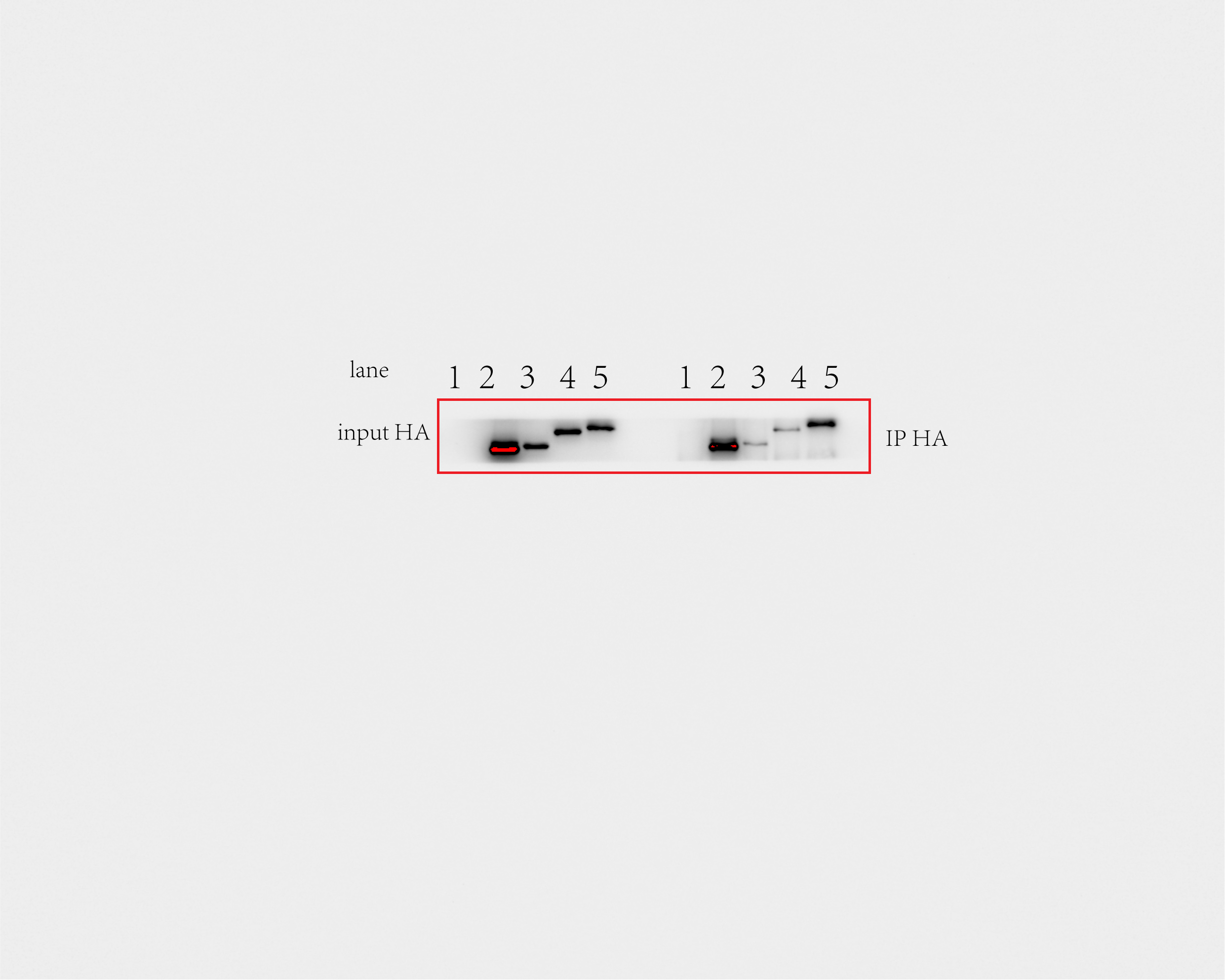

Supplement: Figure 4—figure supplement 1—source data 1. [file elife-101973-fig4-figsupp1-data1.zip › Figure 4–figure supplement 1–source data 1/Figure 4–figure supplement 1A-labeled/IP HA and input HA.tif]

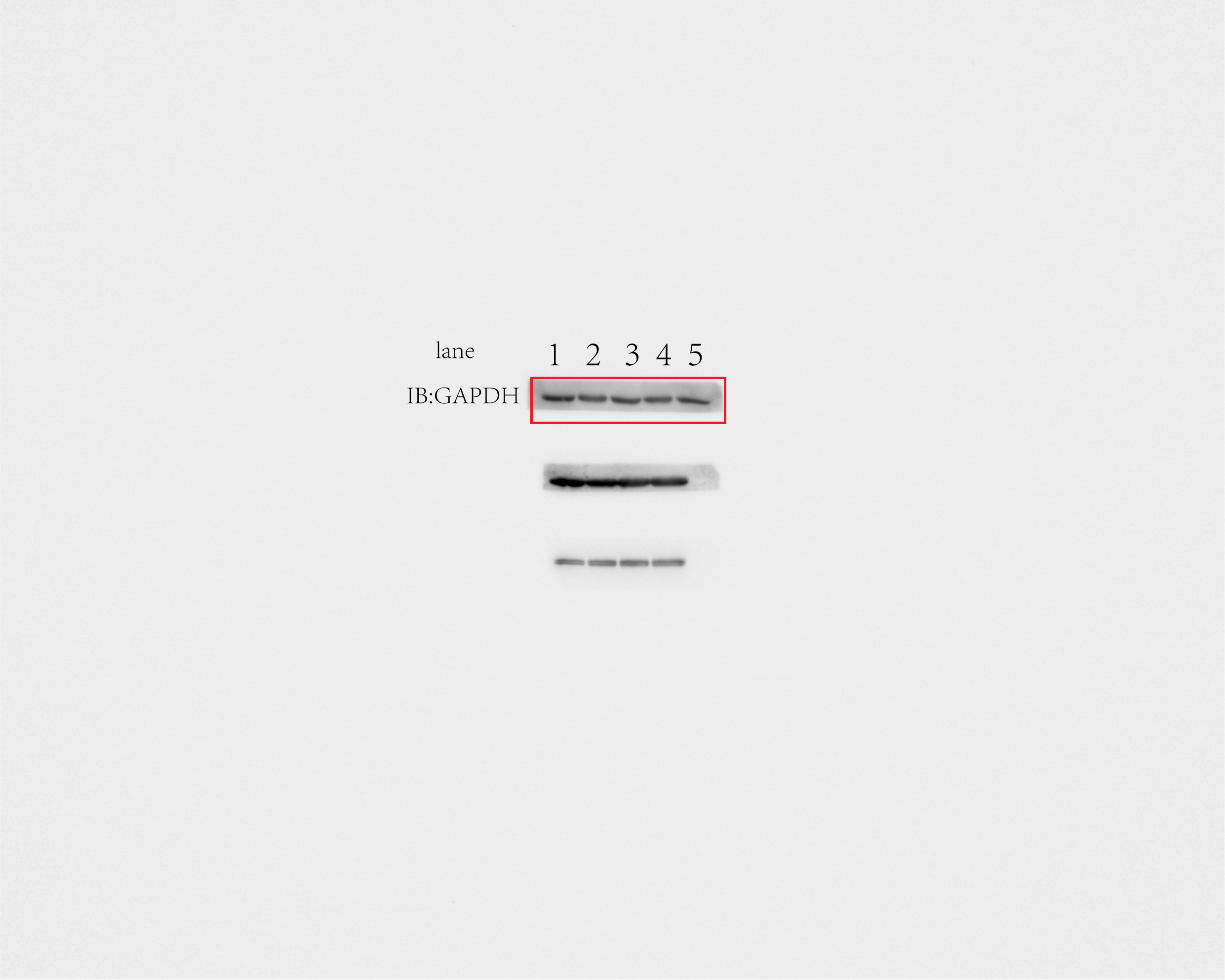

Supplement: Figure 4—figure supplement 1—source data 1. [file elife-101973-fig4-figsupp1-data1.zip › Figure 4–figure supplement 1–source data 1/Figure 4–figure supplement 1A-labeled/input GAPDH.tif]

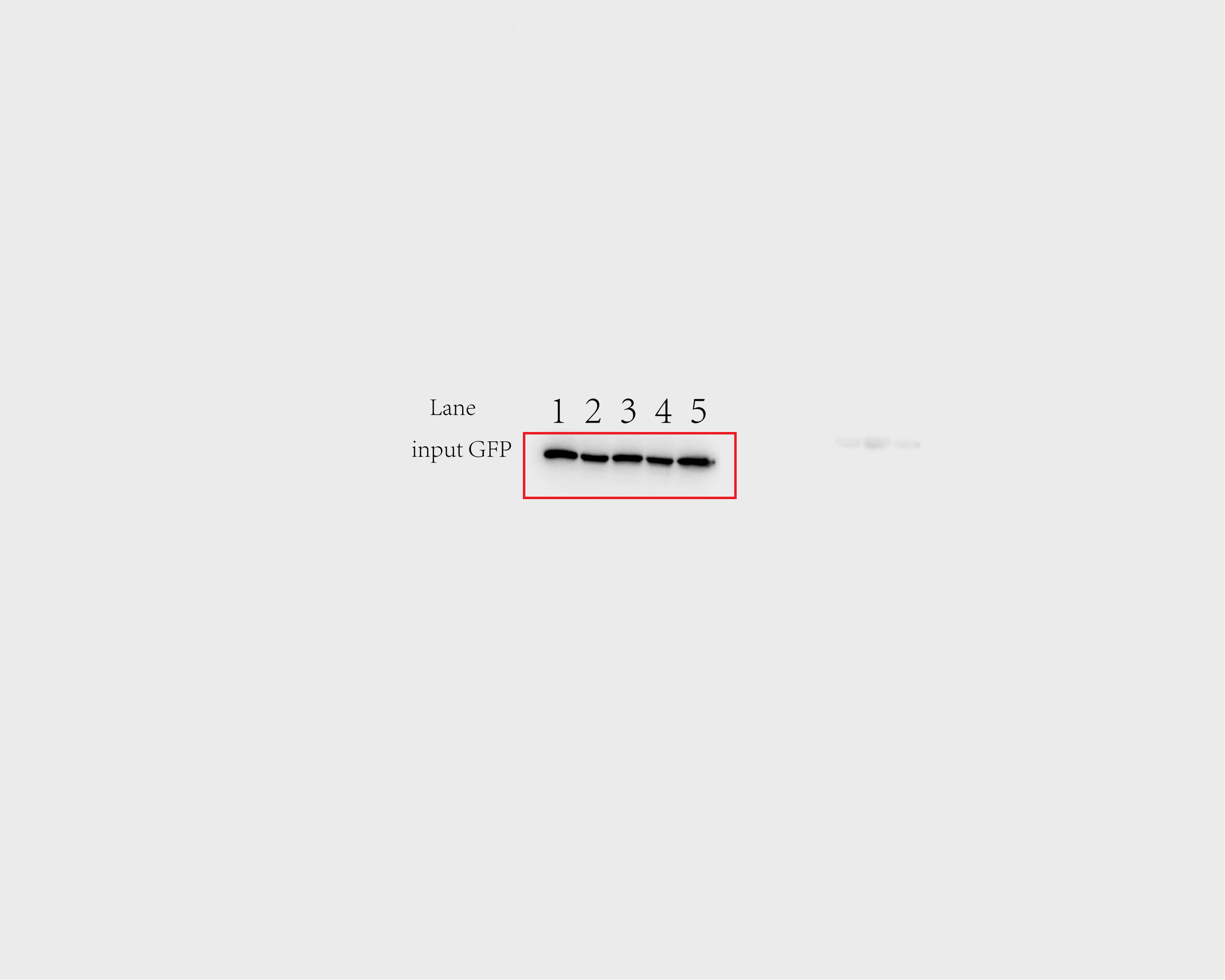

Supplement: Figure 4—figure supplement 1—source data 1. [file elife-101973-fig4-figsupp1-data1.zip › Figure 4–figure supplement 1–source data 1/Figure 4–figure supplement 1A-labeled/input GFP.tif]

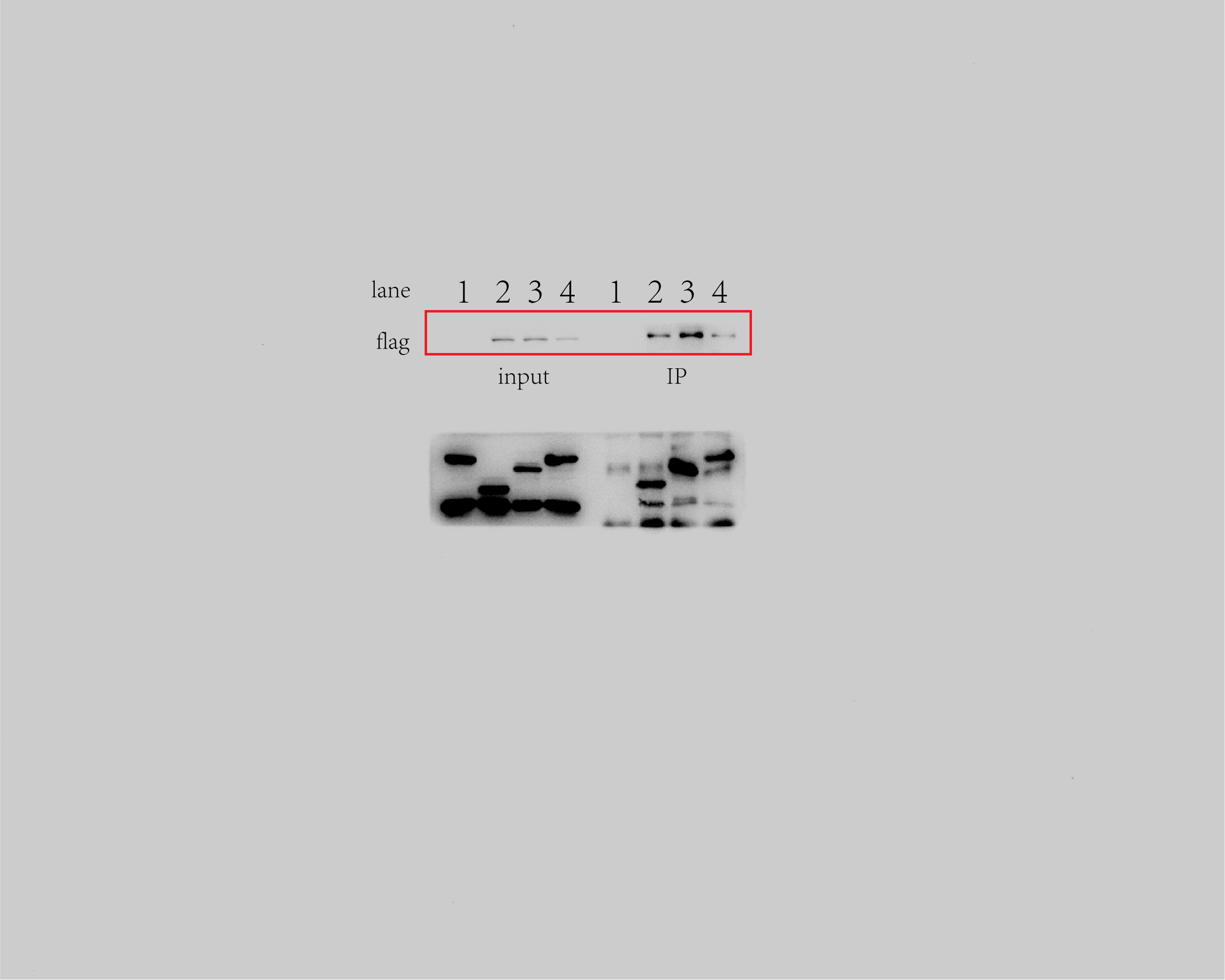

Supplement: Figure 4—figure supplement 1—source data 1. [file elife-101973-fig4-figsupp1-data1.zip › Figure 4–figure supplement 1–source data 1/Figure 4–figure supplement 1C-labeled/input and IP Flag.tif]

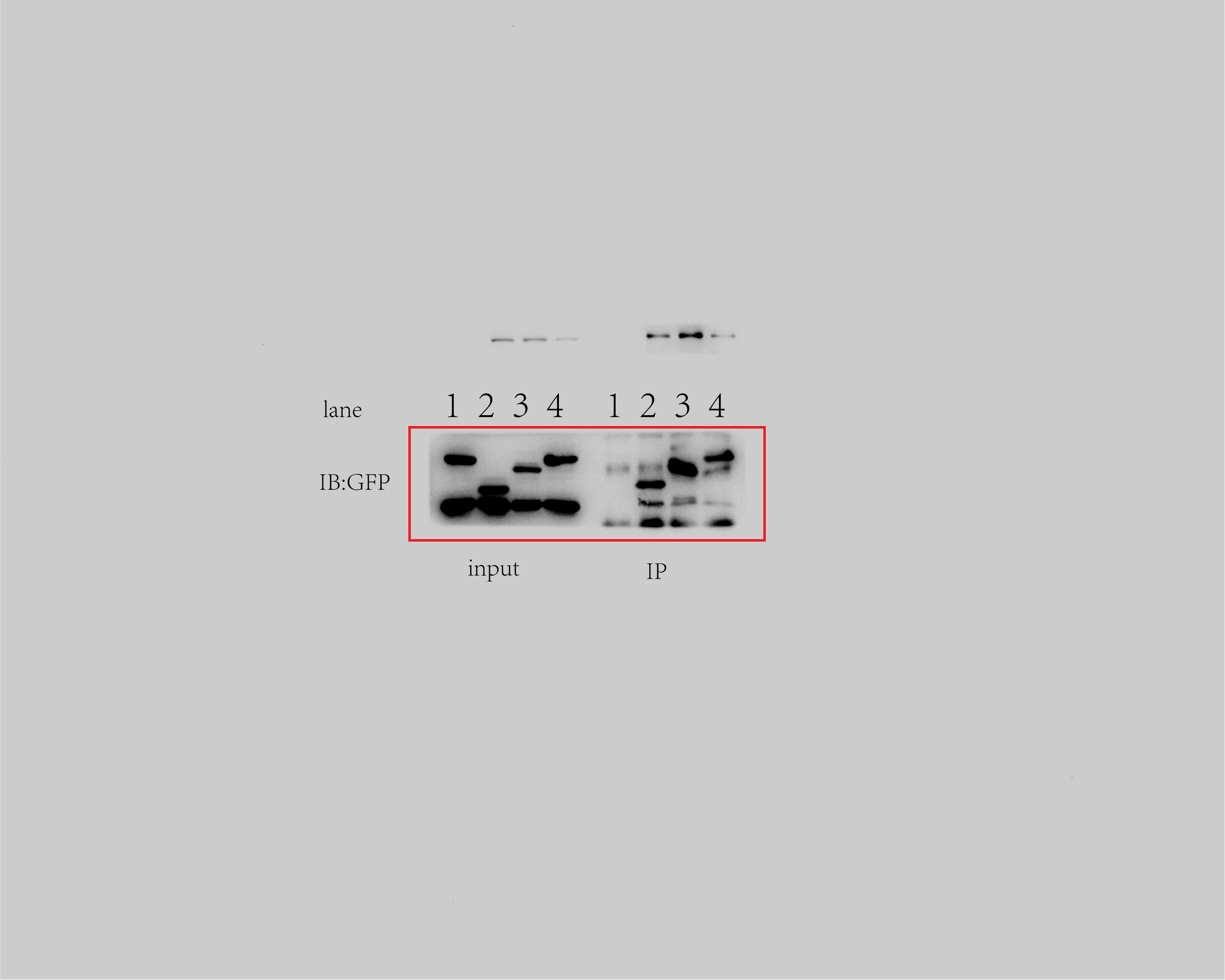

Supplement: Figure 4—figure supplement 1—source data 1. [file elife-101973-fig4-figsupp1-data1.zip › Figure 4–figure supplement 1–source data 1/Figure 4–figure supplement 1C-labeled/input and IP GFP.tif]

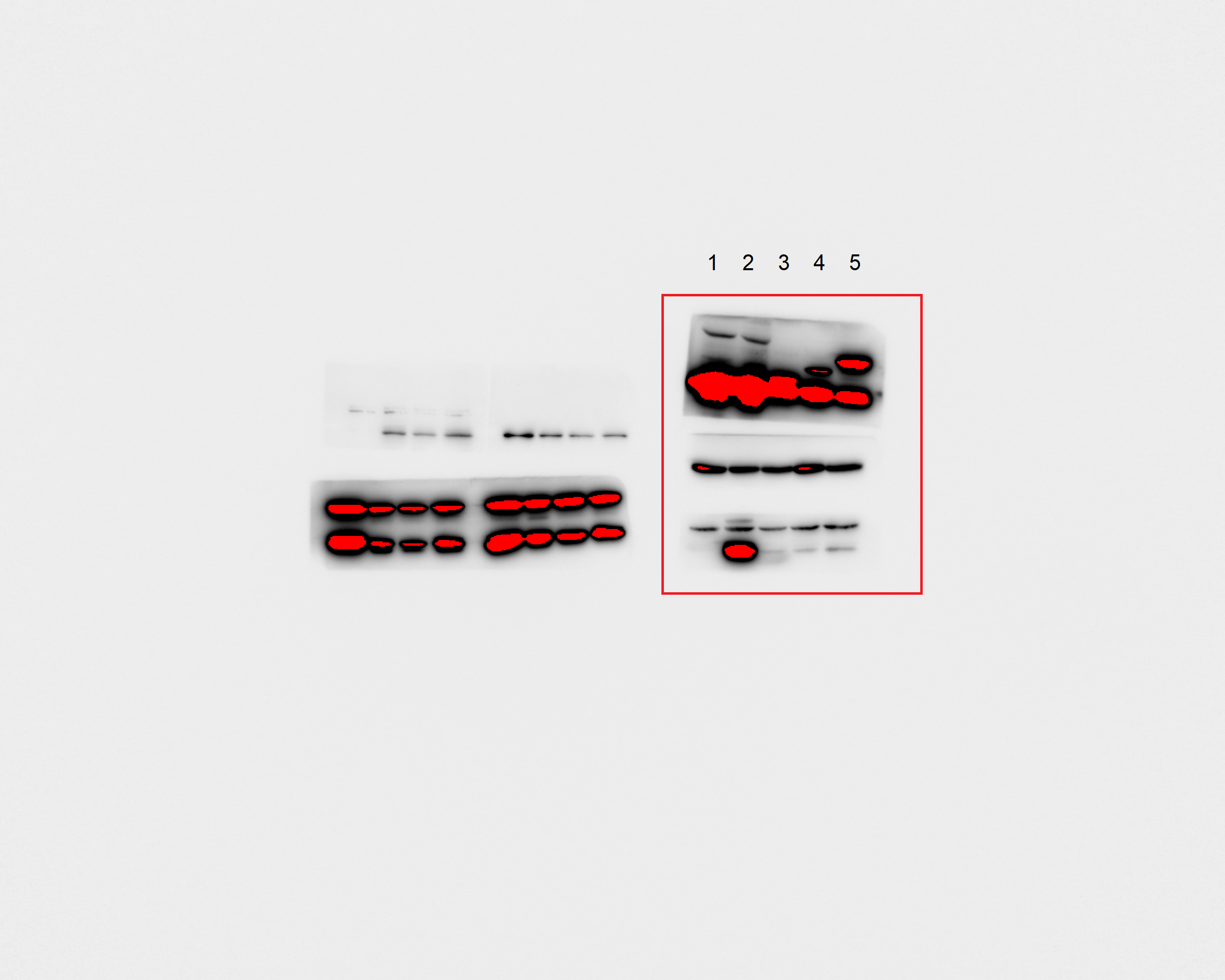

Supplement: Figure 4—figure supplement 1—source data 1. [file elife-101973-fig4-figsupp1-data1.zip › Figure 4–figure supplement 1–source data 1/Figure 4–figure supplement 1F-labeled/long exposure.tif]

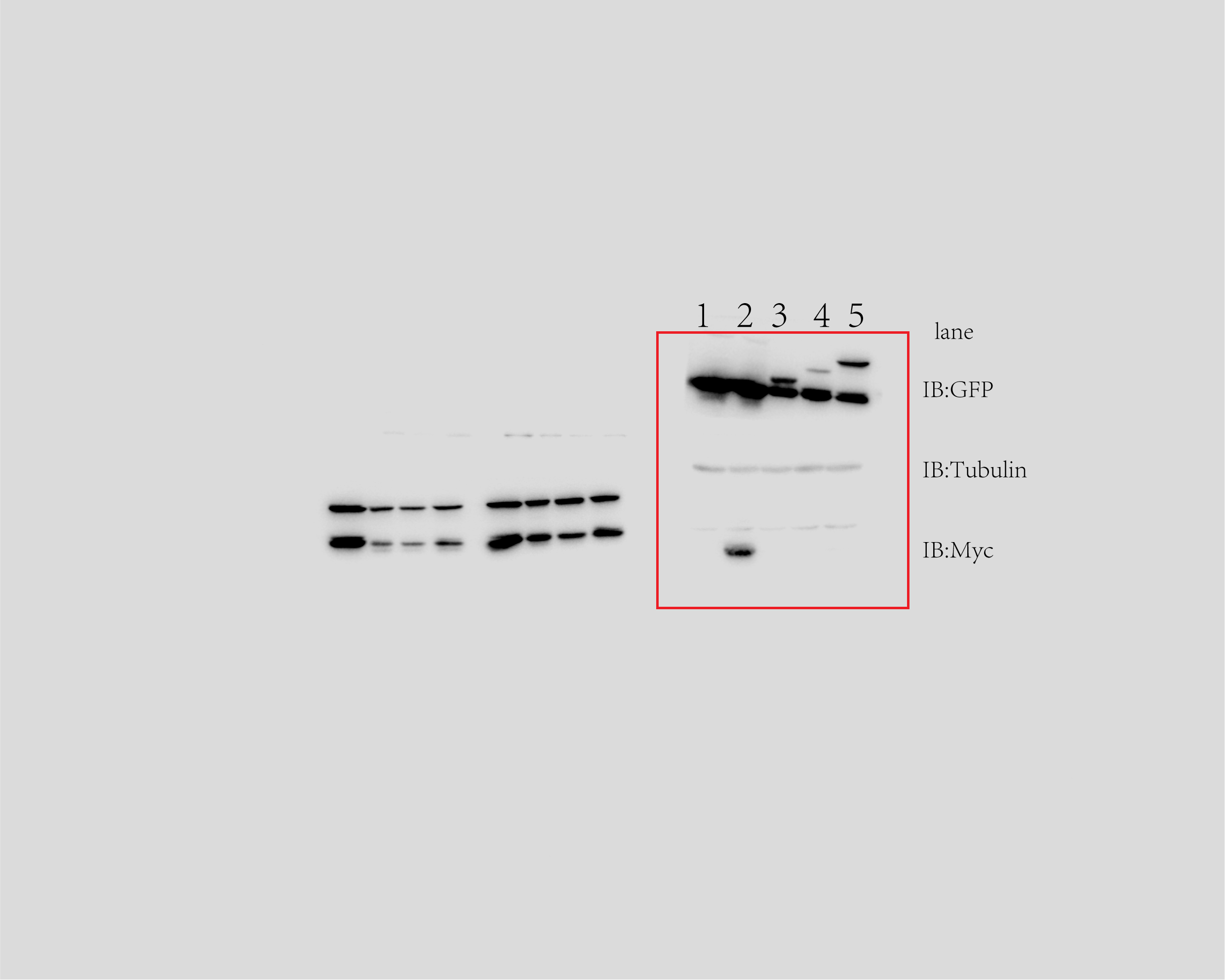

Supplement: Figure 4—figure supplement 1—source data 1. [file elife-101973-fig4-figsupp1-data1.zip › Figure 4–figure supplement 1–source data 1/Figure 4–figure supplement 1F-labeled/short exposure of Myc GFP Tubulin.tif]

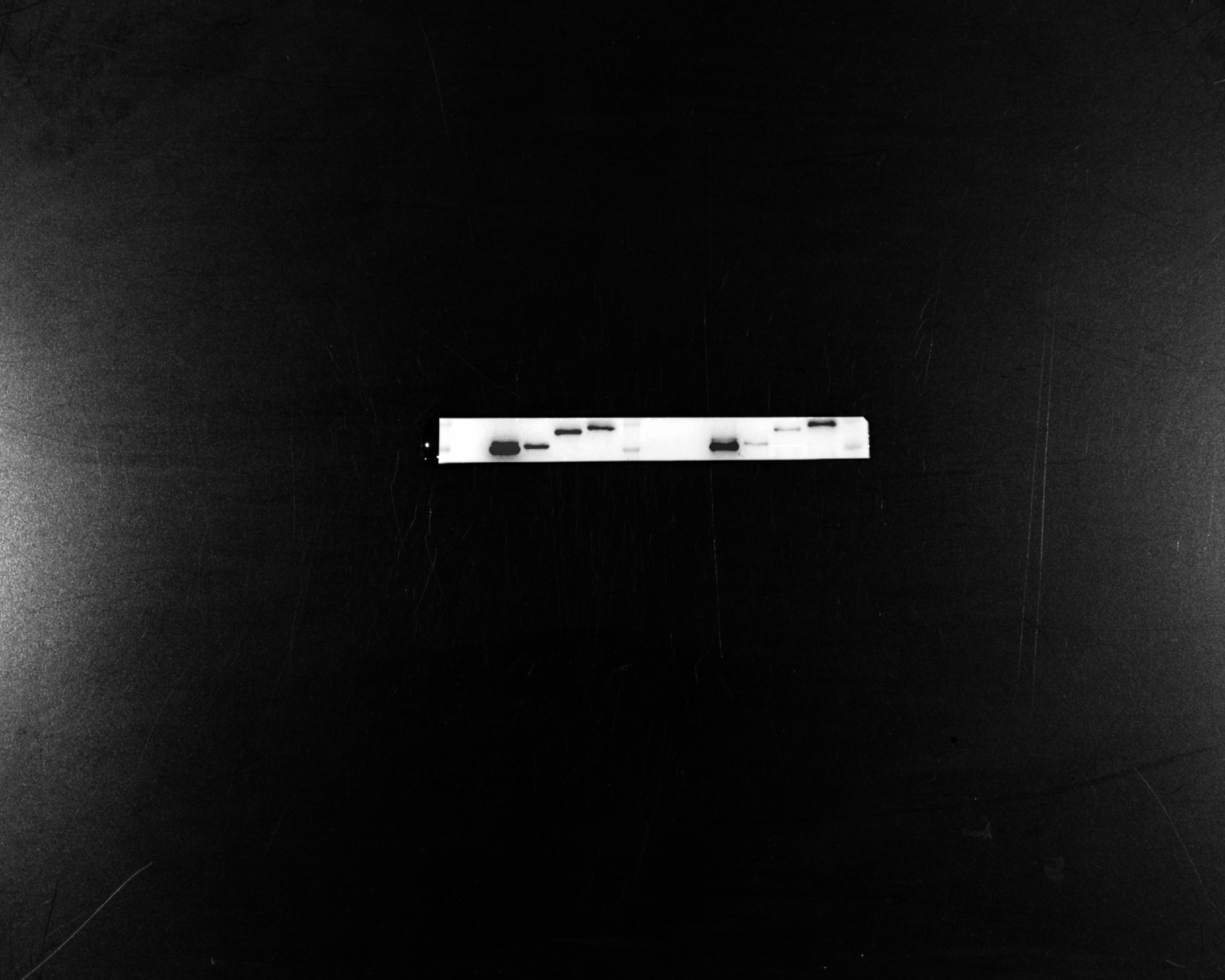

Supplement: Figure 4—figure supplement 1—source data 2. [file elife-101973-fig4-figsupp1-data2.zip › Figure 4-figure supplement 1-source data 2/Figure 4-figure supplement 1A/IP and input HA.jpg]

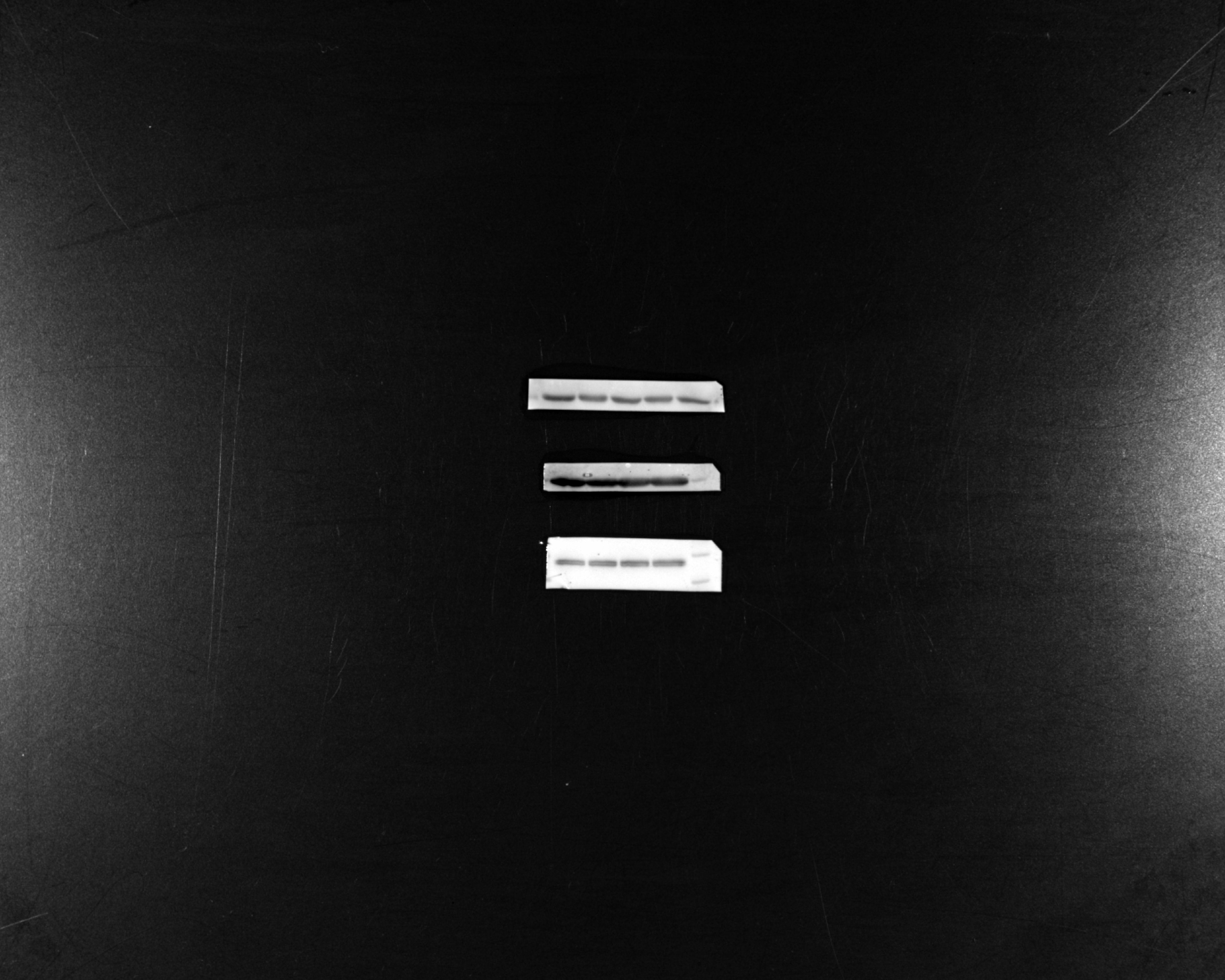

Supplement: Figure 4—figure supplement 1—source data 2. [file elife-101973-fig4-figsupp1-data2.zip › Figure 4-figure supplement 1-source data 2/Figure 4-figure supplement 1A/input GAPDH.jpg]

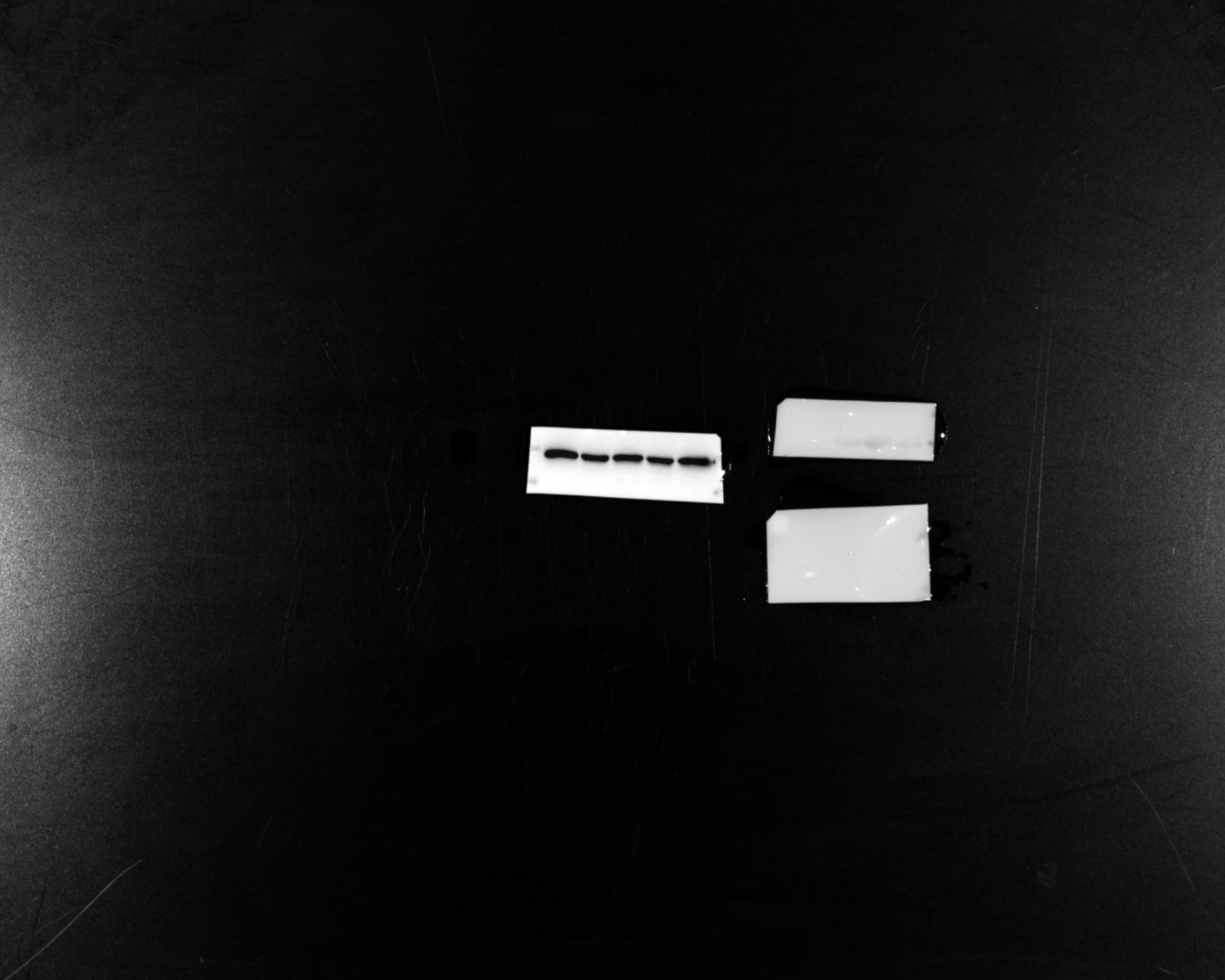

Supplement: Figure 4—figure supplement 1—source data 2. [file elife-101973-fig4-figsupp1-data2.zip › Figure 4-figure supplement 1-source data 2/Figure 4-figure supplement 1A/input GFP.jpg]

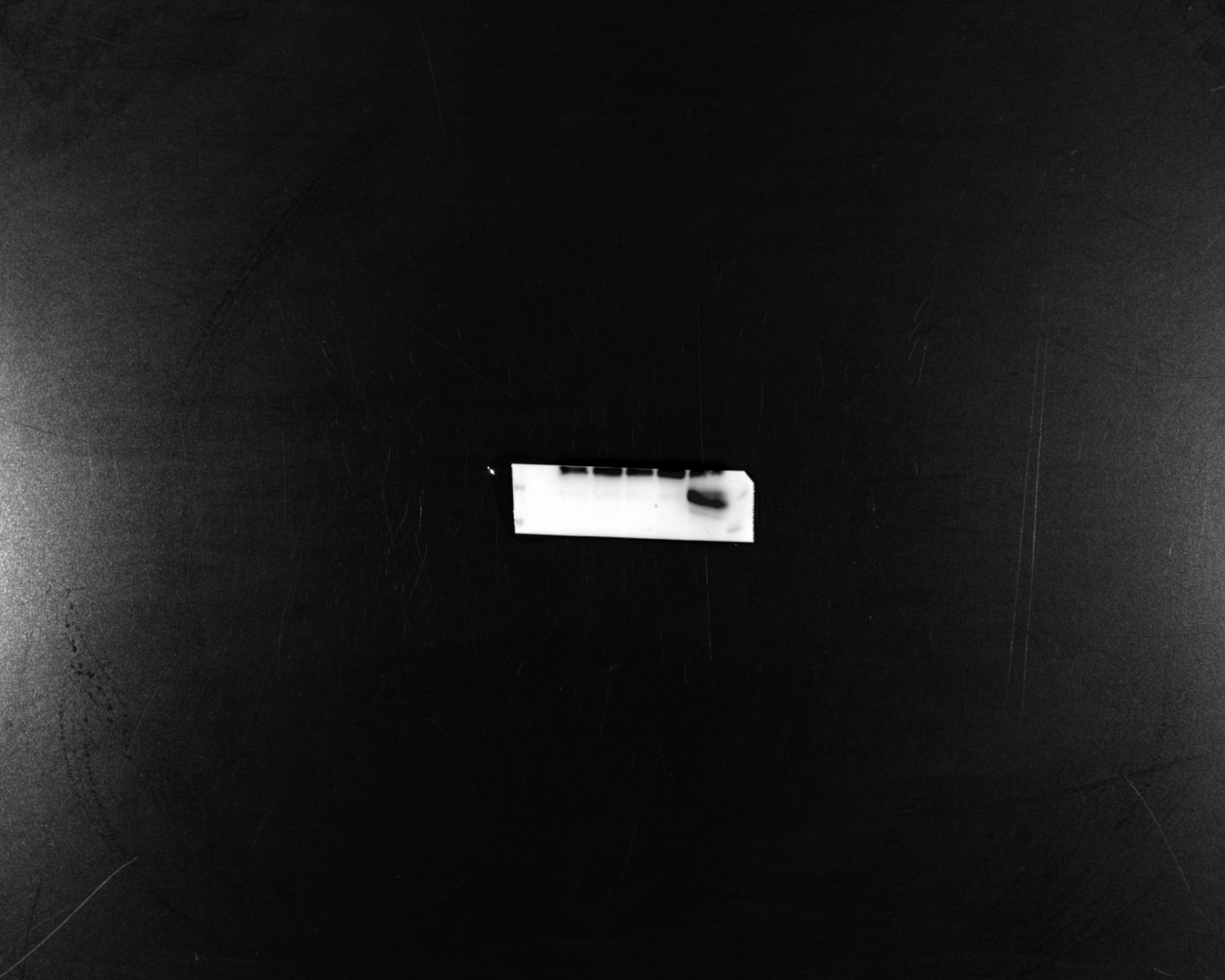

Supplement: Figure 4—figure supplement 1—source data 2. [file elife-101973-fig4-figsupp1-data2.zip › Figure 4-figure supplement 1-source data 2/Figure 4-figure supplement 1A/ip GFP.jpg]

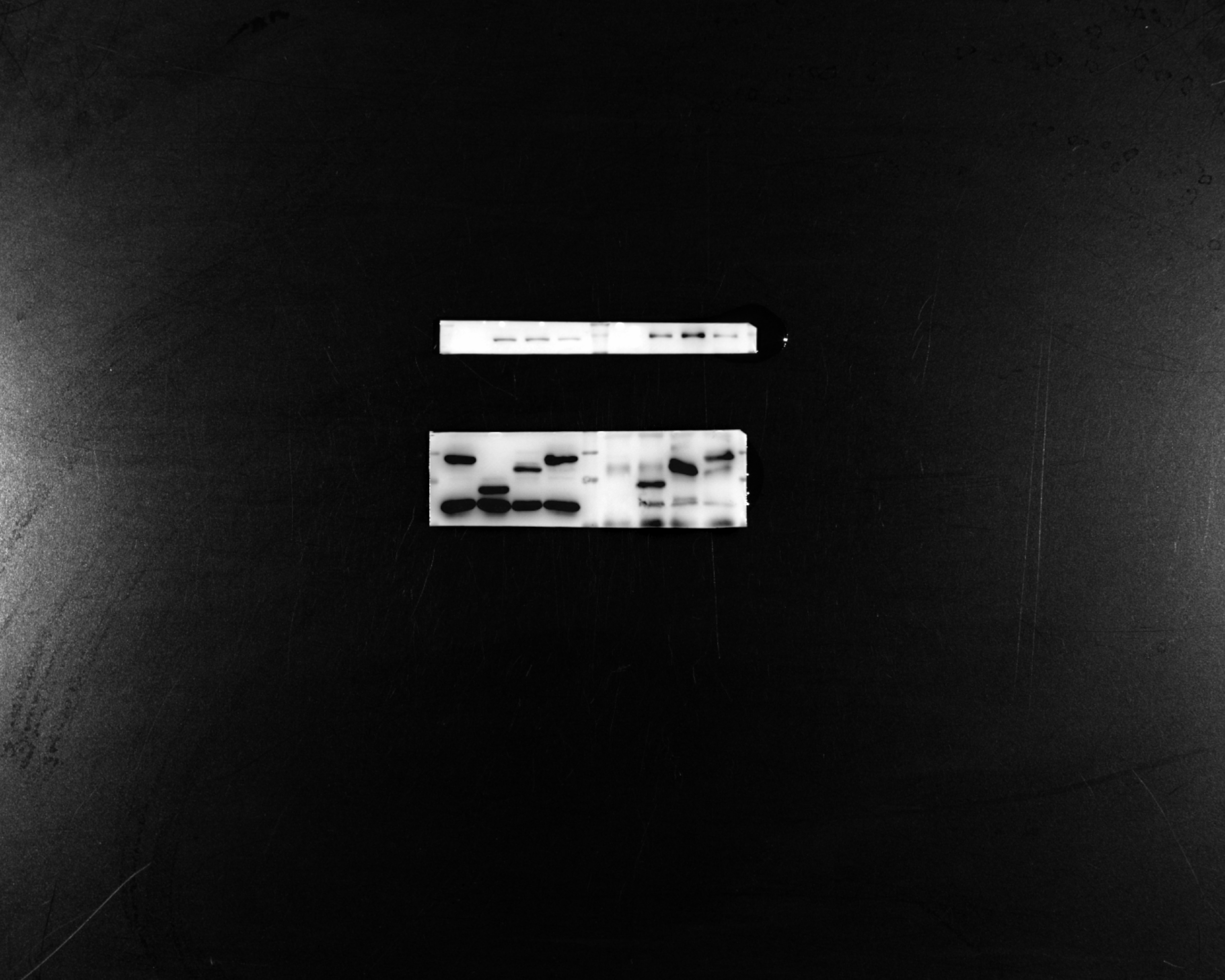

Supplement: Figure 4—figure supplement 1—source data 2. [file elife-101973-fig4-figsupp1-data2.zip › Figure 4-figure supplement 1-source data 2/Figure 4-figure supplement 1C/input and IP flag GFP.jpg]

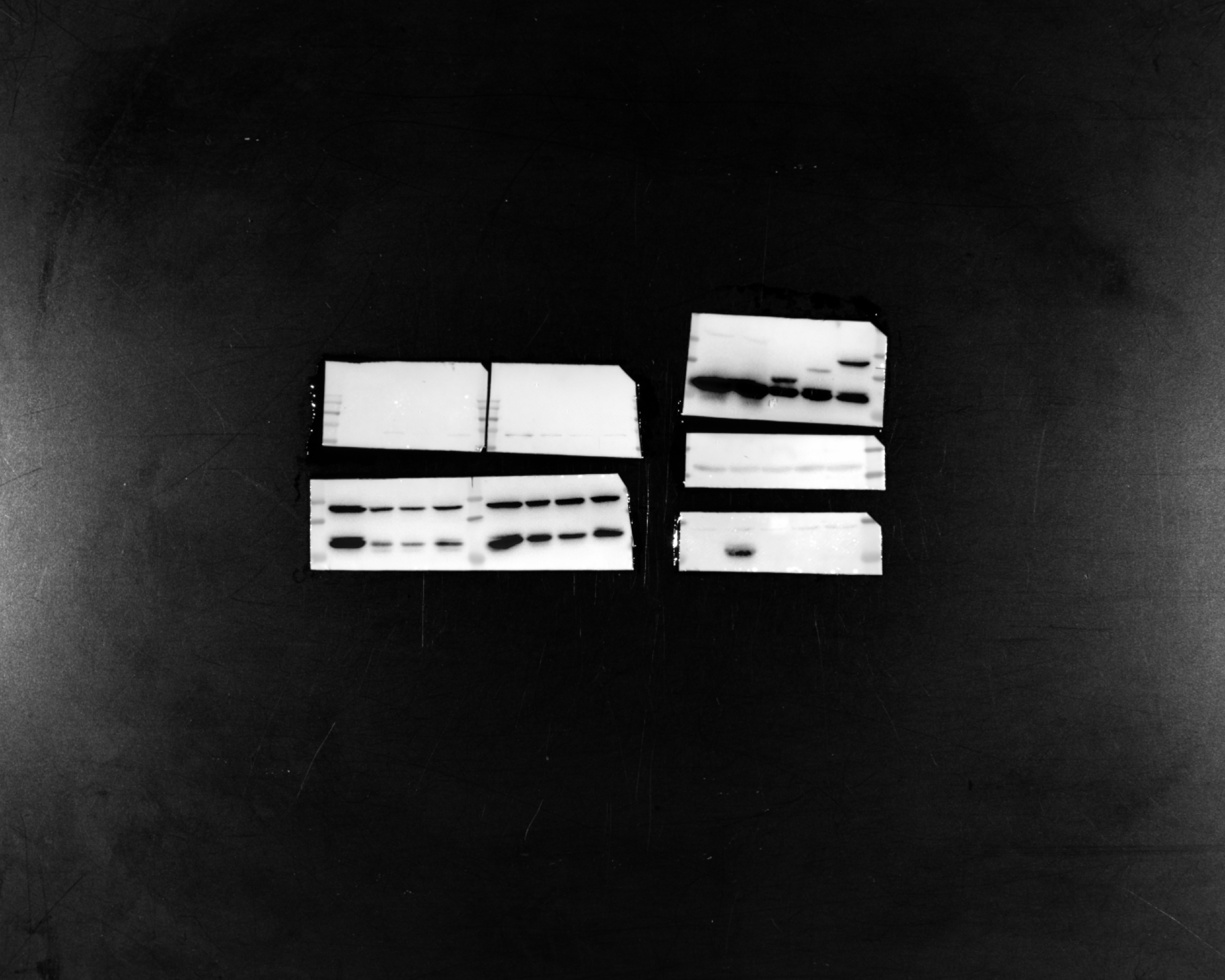

Supplement: Figure 4—figure supplement 1—source data 2. [file elife-101973-fig4-figsupp1-data2.zip › Figure 4-figure supplement 1-source data 2/Figure 4-figure supplement 1F/Myc GFP and tubulin.jpg]

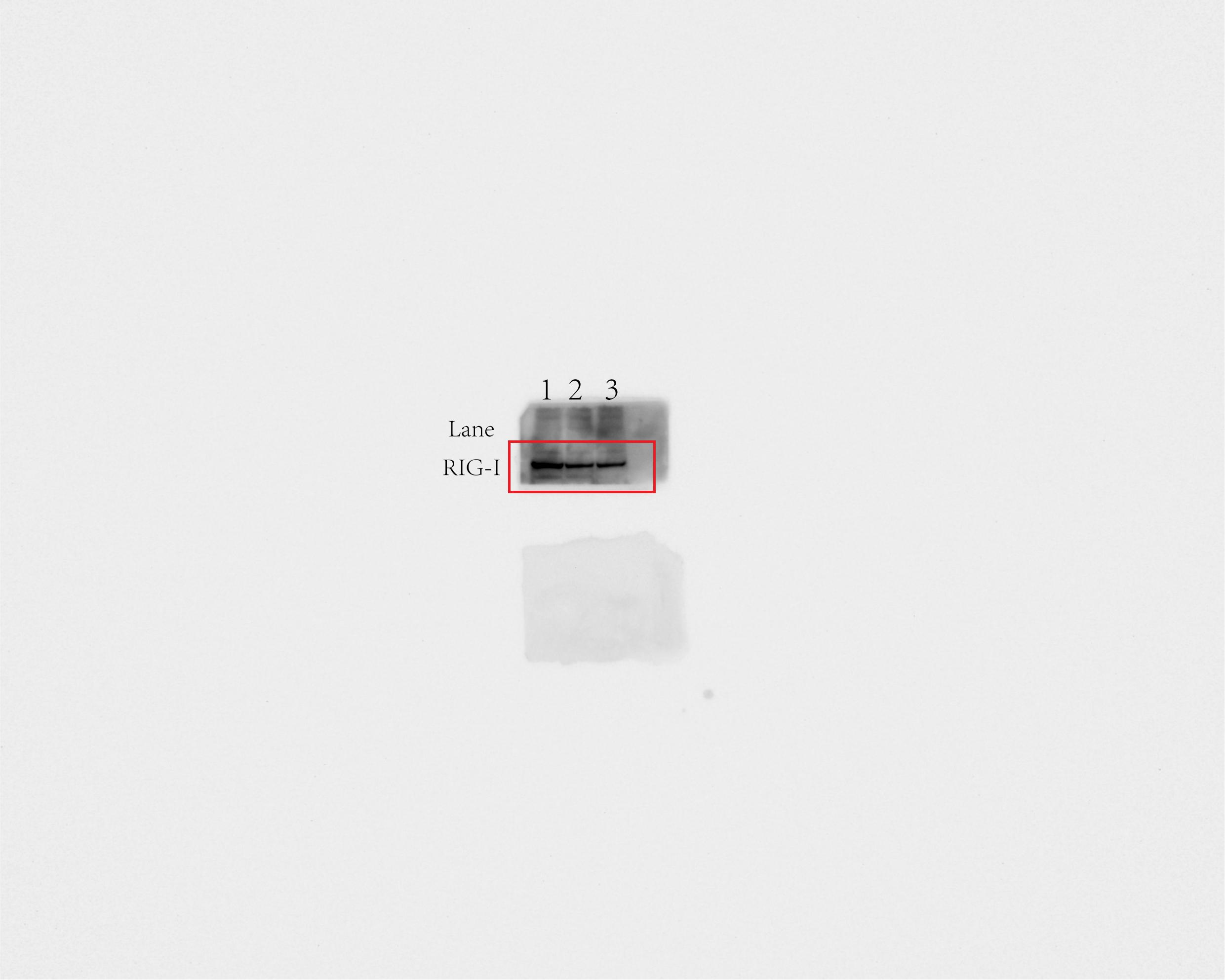

Supplement: Figure 5—source data 1. [file elife-101973-fig5-data1.zip › Figure 5–source data 1/Fig5E-labeled/RIG-I.tif]

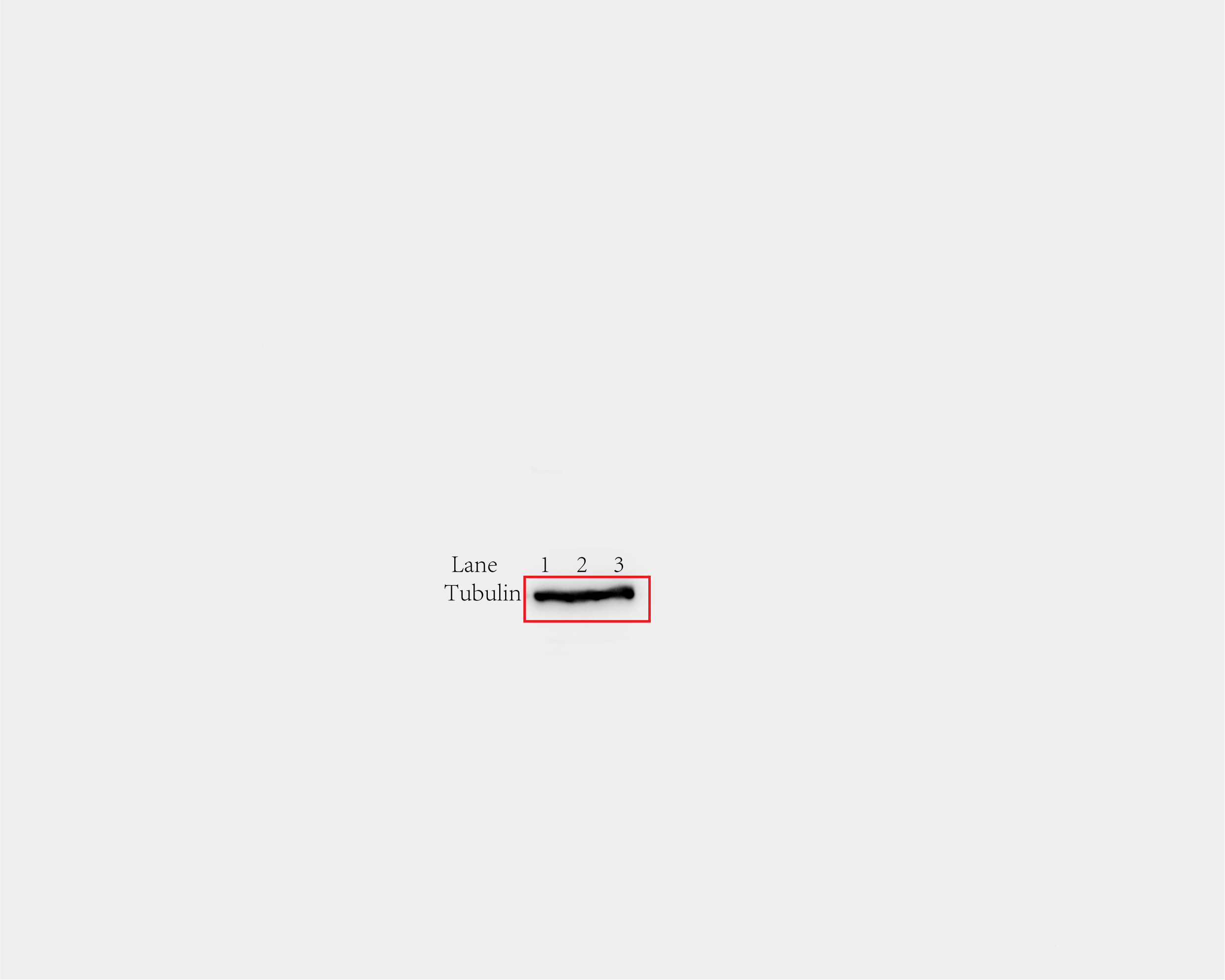

Supplement: Figure 5—source data 1. [file elife-101973-fig5-data1.zip › Figure 5–source data 1/Fig5E-labeled/Tubulin.tif]

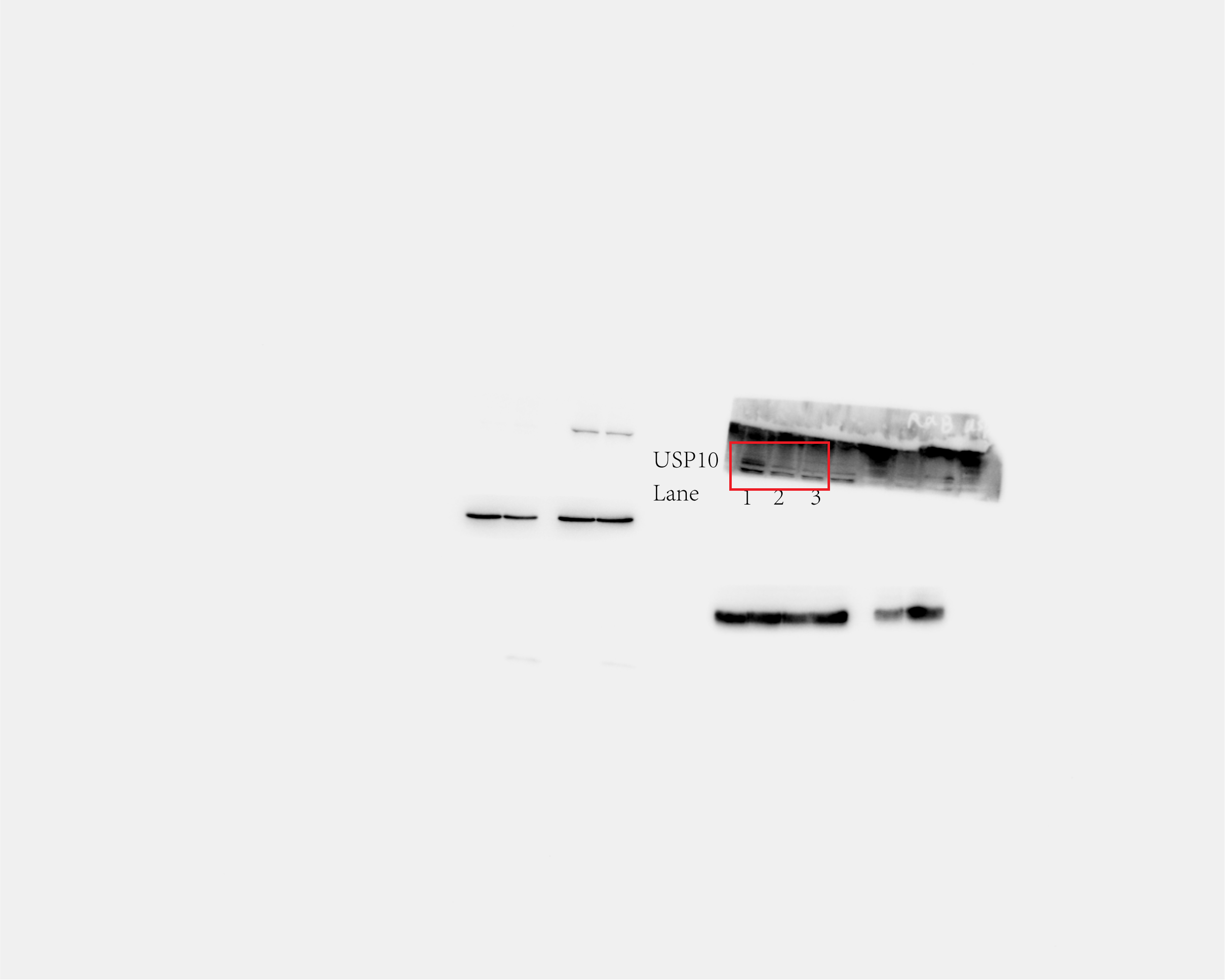

Supplement: Figure 5—source data 1. [file elife-101973-fig5-data1.zip › Figure 5–source data 1/Fig5E-labeled/USP10.tif]

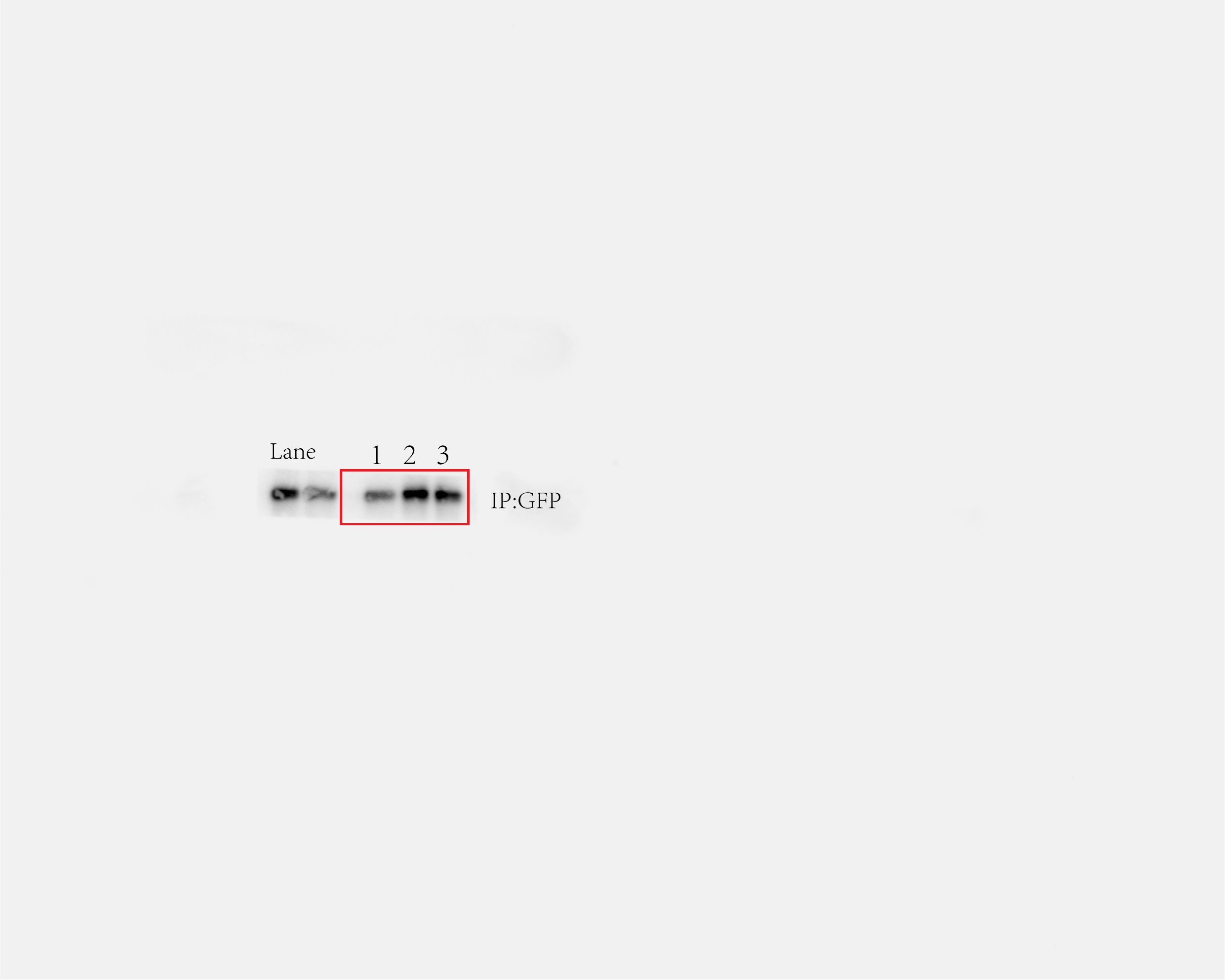

Supplement: Figure 5—source data 1. [file elife-101973-fig5-data1.zip › Figure 5–source data 1/Fig5B-labeled/IP GFP.tif]

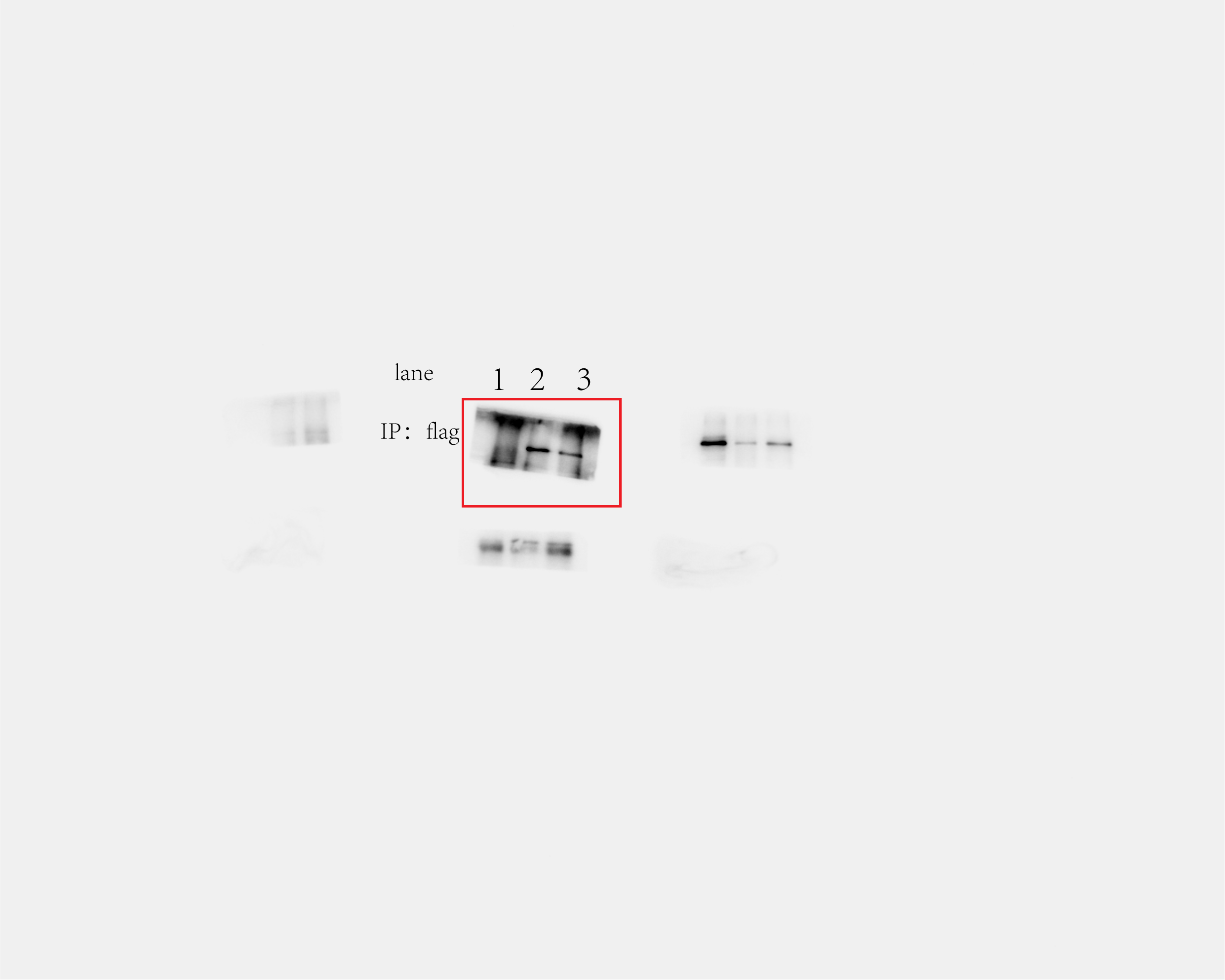

Supplement: Figure 5—source data 1. [file elife-101973-fig5-data1.zip › Figure 5–source data 1/Fig5B-labeled/IP flag.tif]

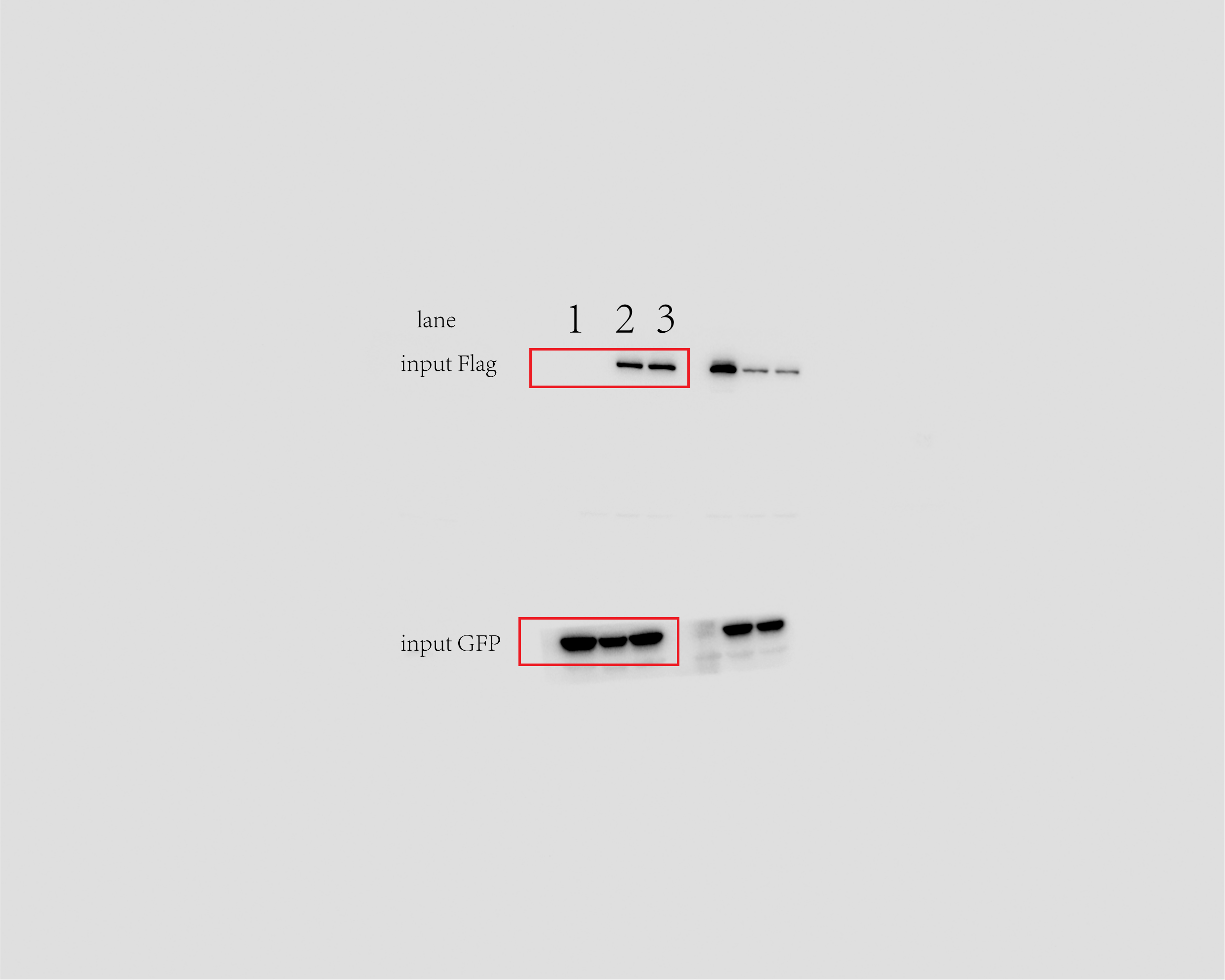

Supplement: Figure 5—source data 1. [file elife-101973-fig5-data1.zip › Figure 5–source data 1/Fig5B-labeled/input GFP flag.tif]

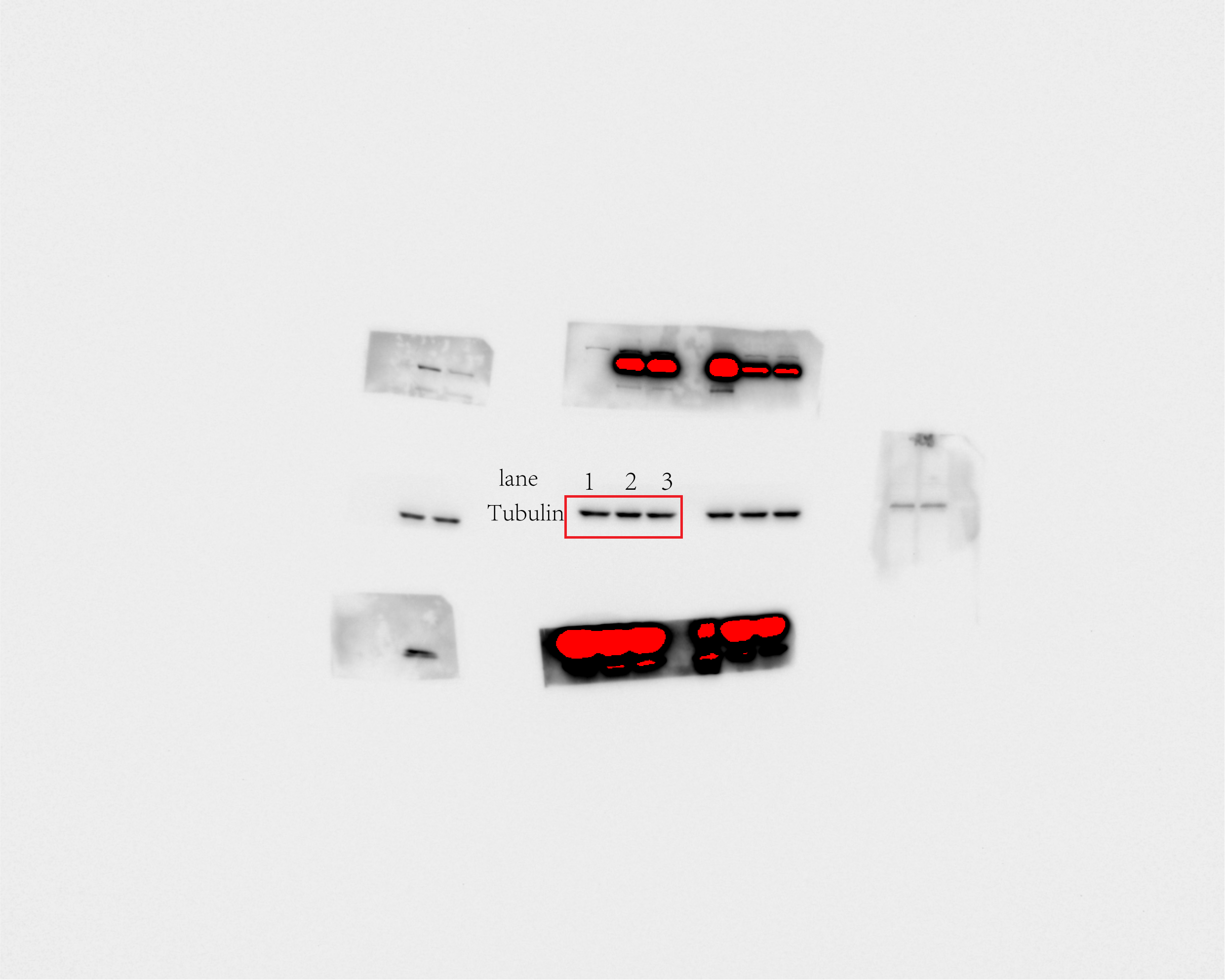

Supplement: Figure 5—source data 1. [file elife-101973-fig5-data1.zip › Figure 5–source data 1/Fig5B-labeled/input tubulin.tif]

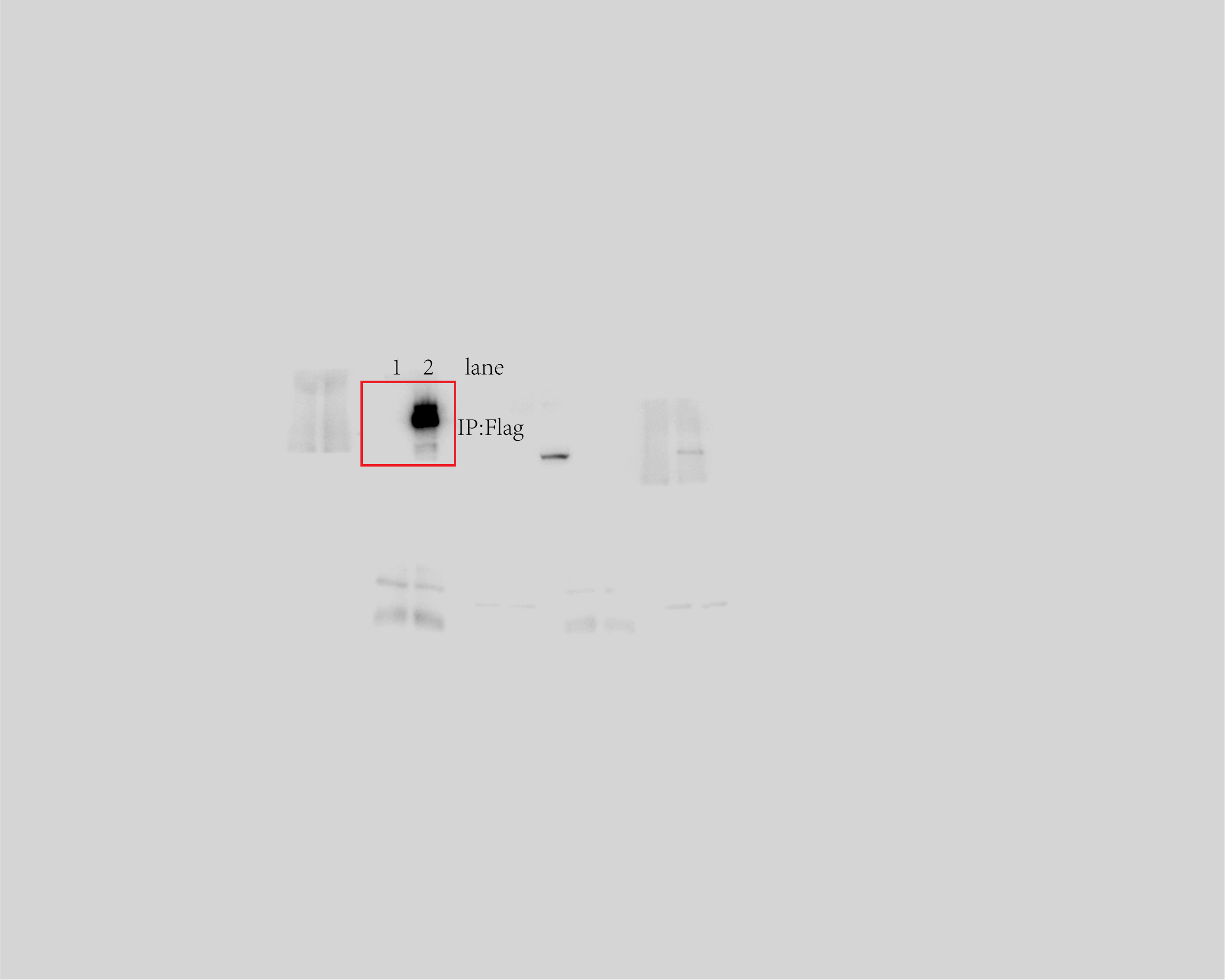

Supplement: Figure 5—source data 1. [file elife-101973-fig5-data1.zip › Figure 5–source data 1/Fig5C-labeled/IP flag.tif]

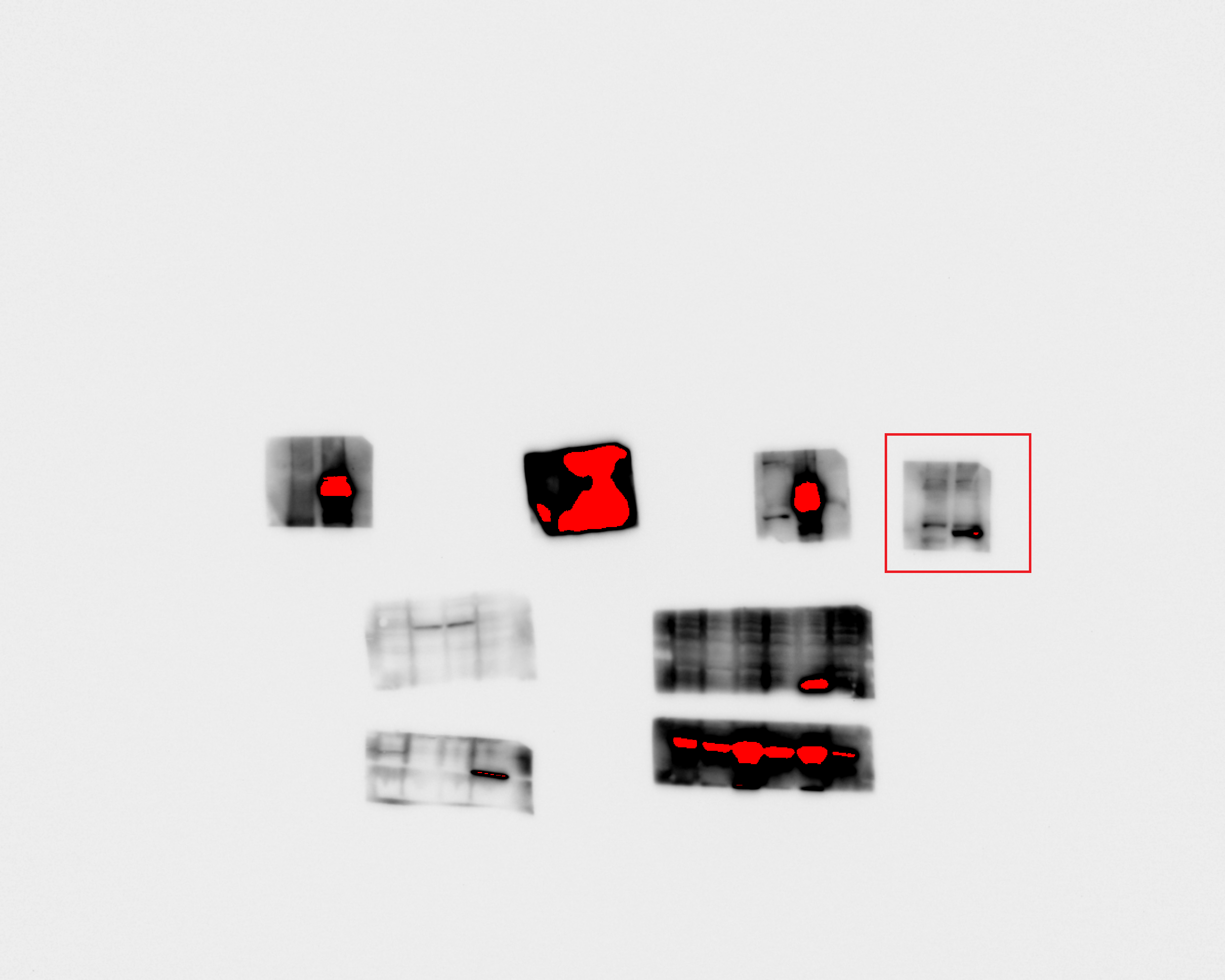

Supplement: Figure 5—source data 1. [file elife-101973-fig5-data1.zip › Figure 5–source data 1/Fig5C-labeled/IP myc long exposure.tif]

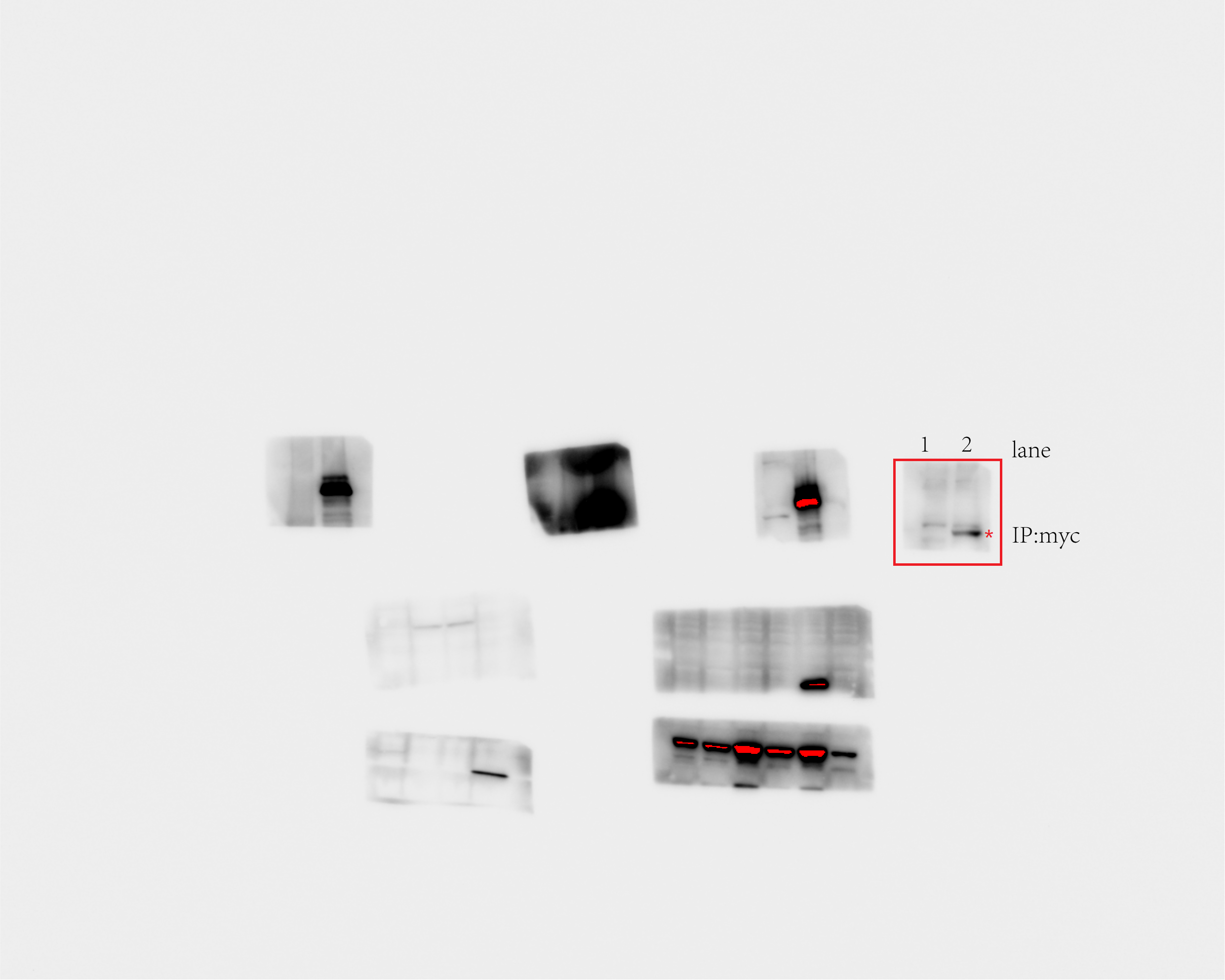

Supplement: Figure 5—source data 1. [file elife-101973-fig5-data1.zip › Figure 5–source data 1/Fig5C-labeled/IP myc.tif]

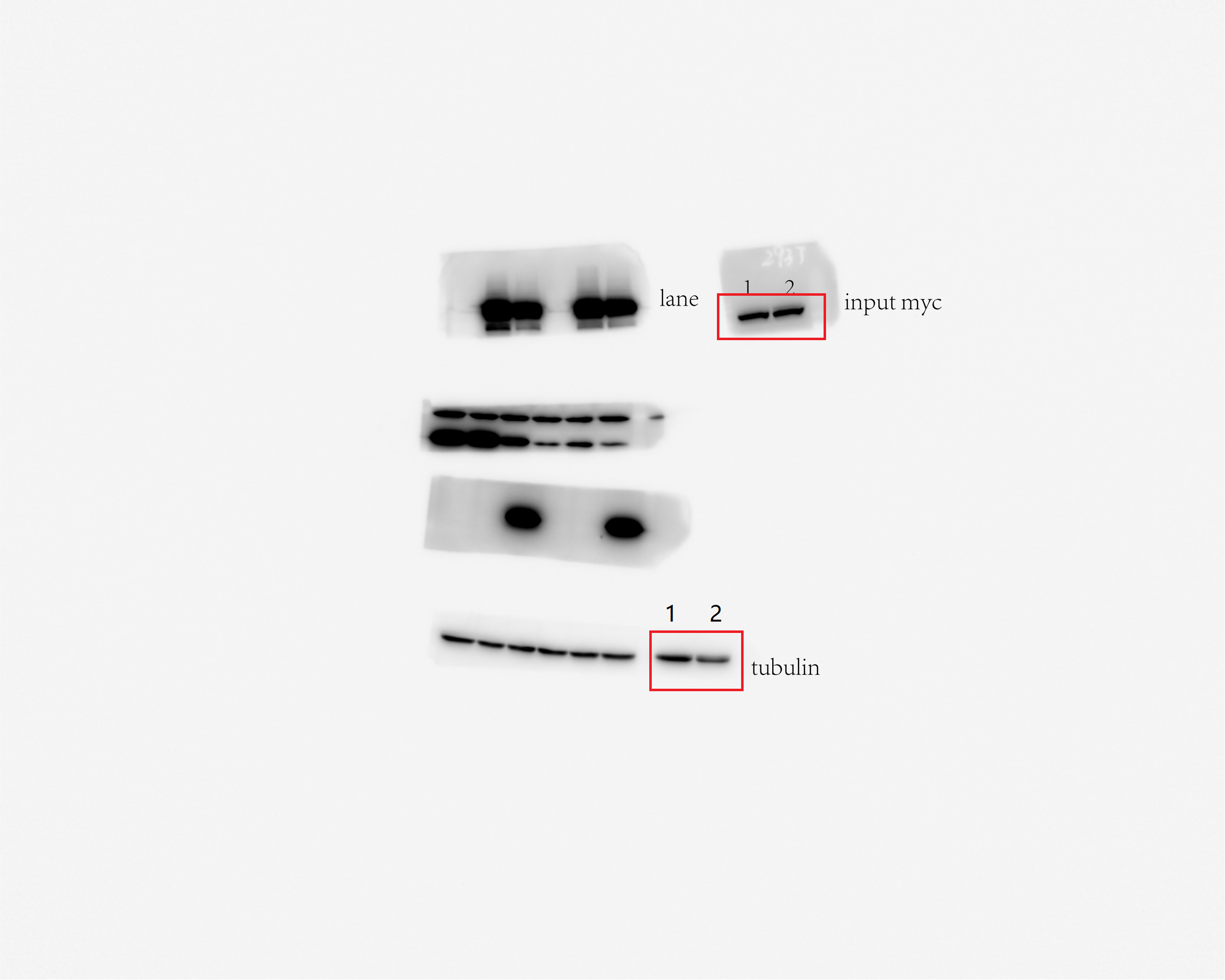

Supplement: Figure 5—source data 1. [file elife-101973-fig5-data1.zip › Figure 5–source data 1/Fig5C-labeled/Input myc and tubulin.tif]

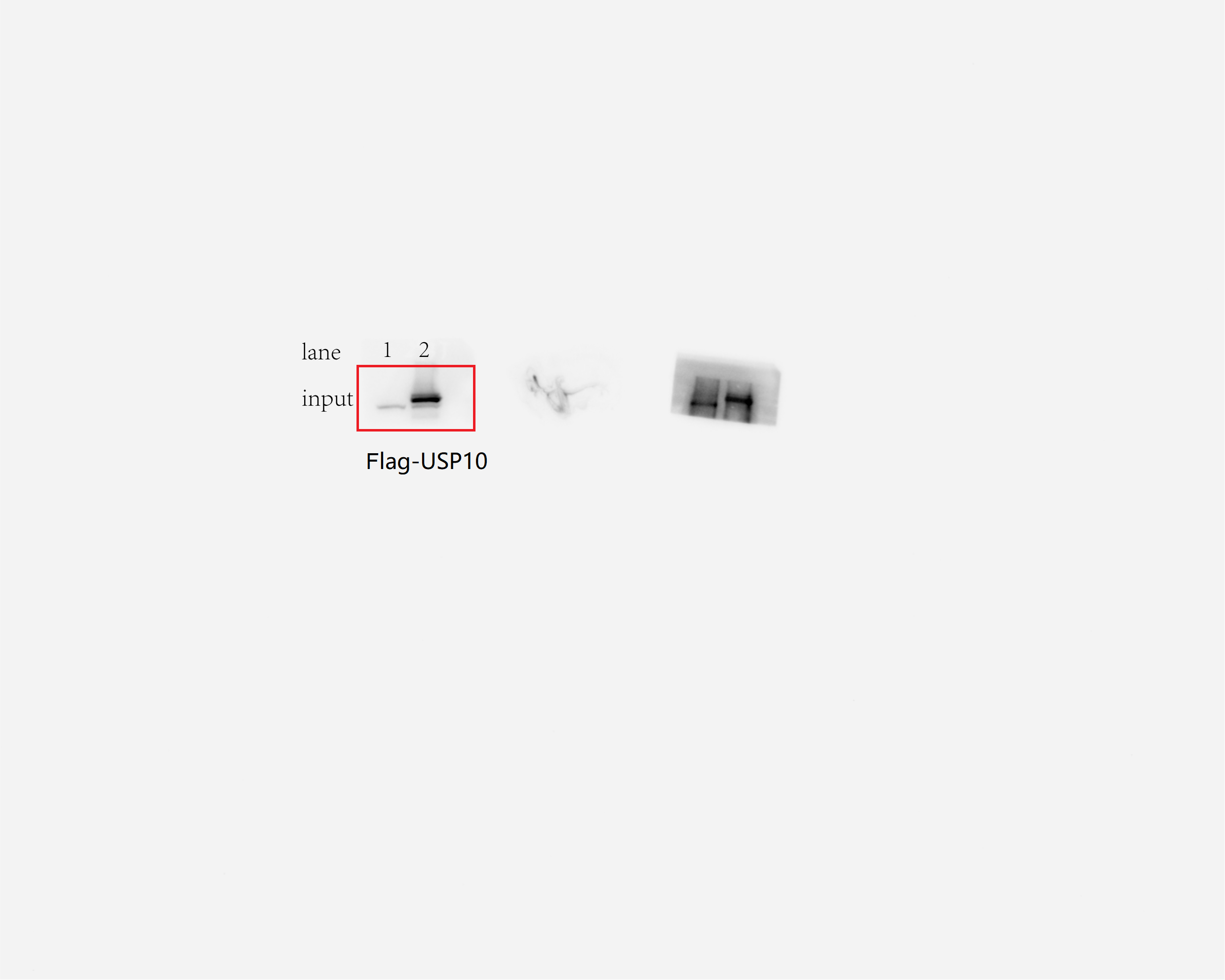

Supplement: Figure 5—source data 1. [file elife-101973-fig5-data1.zip › Figure 5–source data 1/Fig5C-labeled/input flag.tif]

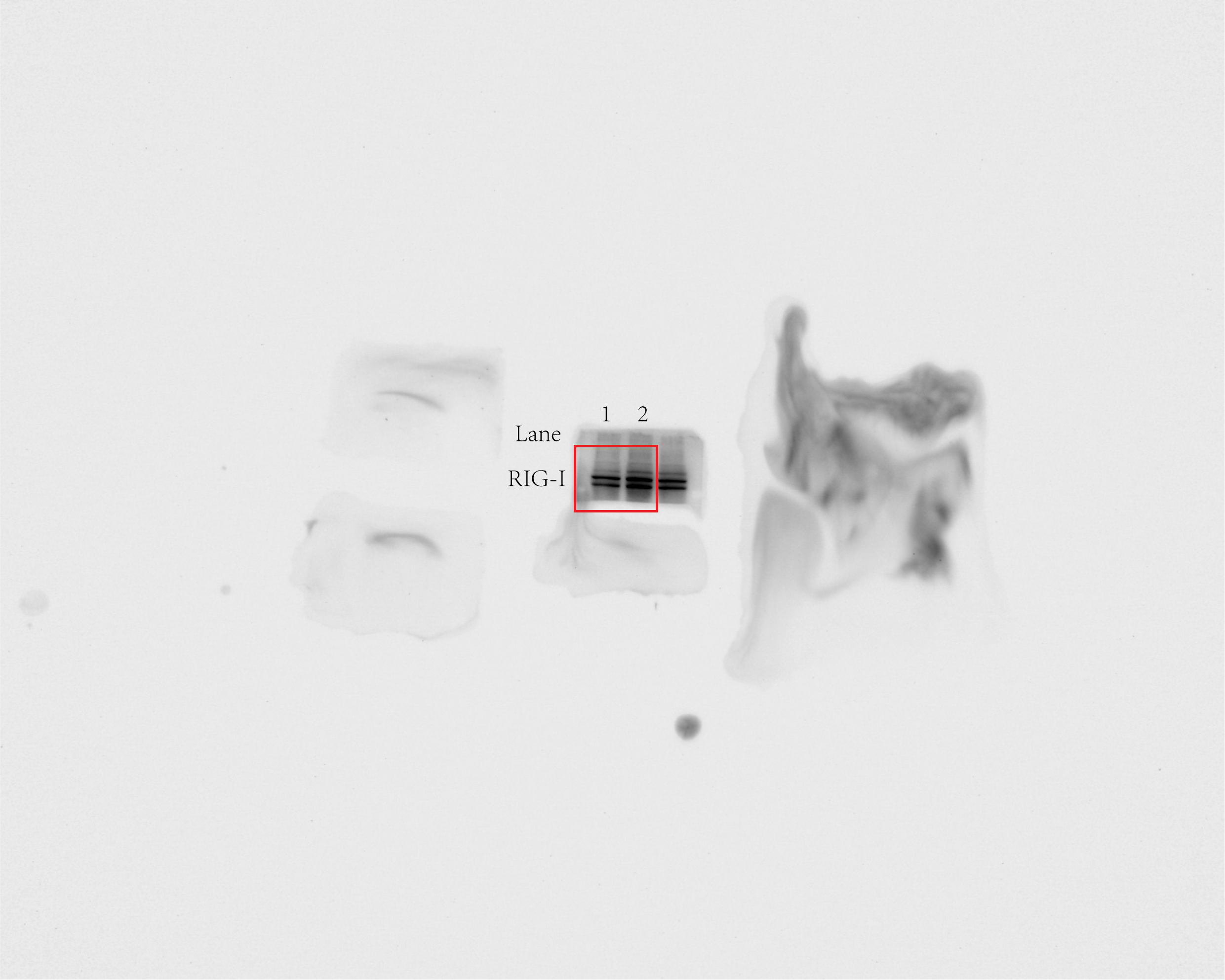

Supplement: Figure 5—source data 1. [file elife-101973-fig5-data1.zip › Figure 5–source data 1/Fig5D-labeled/RIG-I.tif]

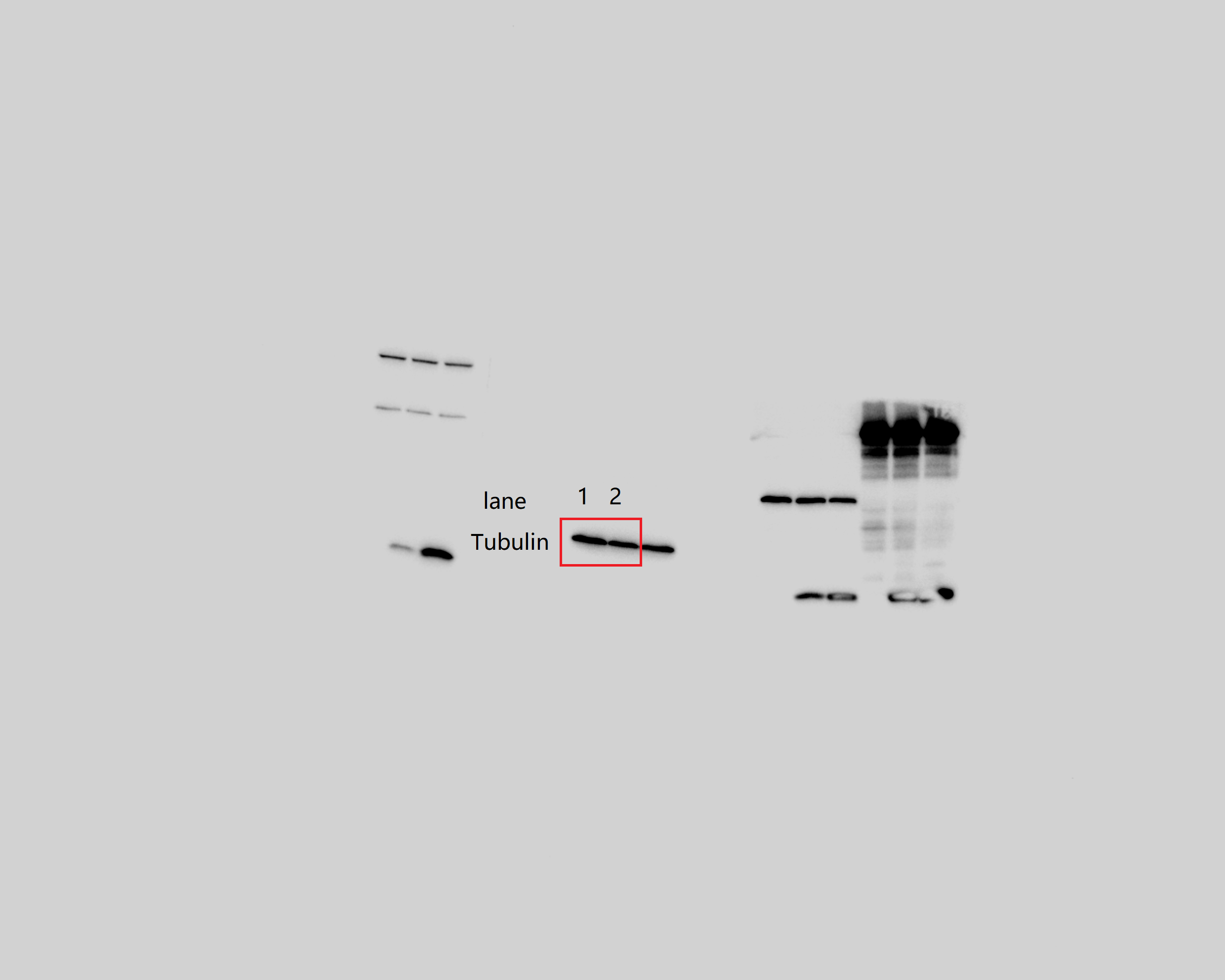

Supplement: Figure 5—source data 1. [file elife-101973-fig5-data1.zip › Figure 5–source data 1/Fig5D-labeled/Tubulin.tif]

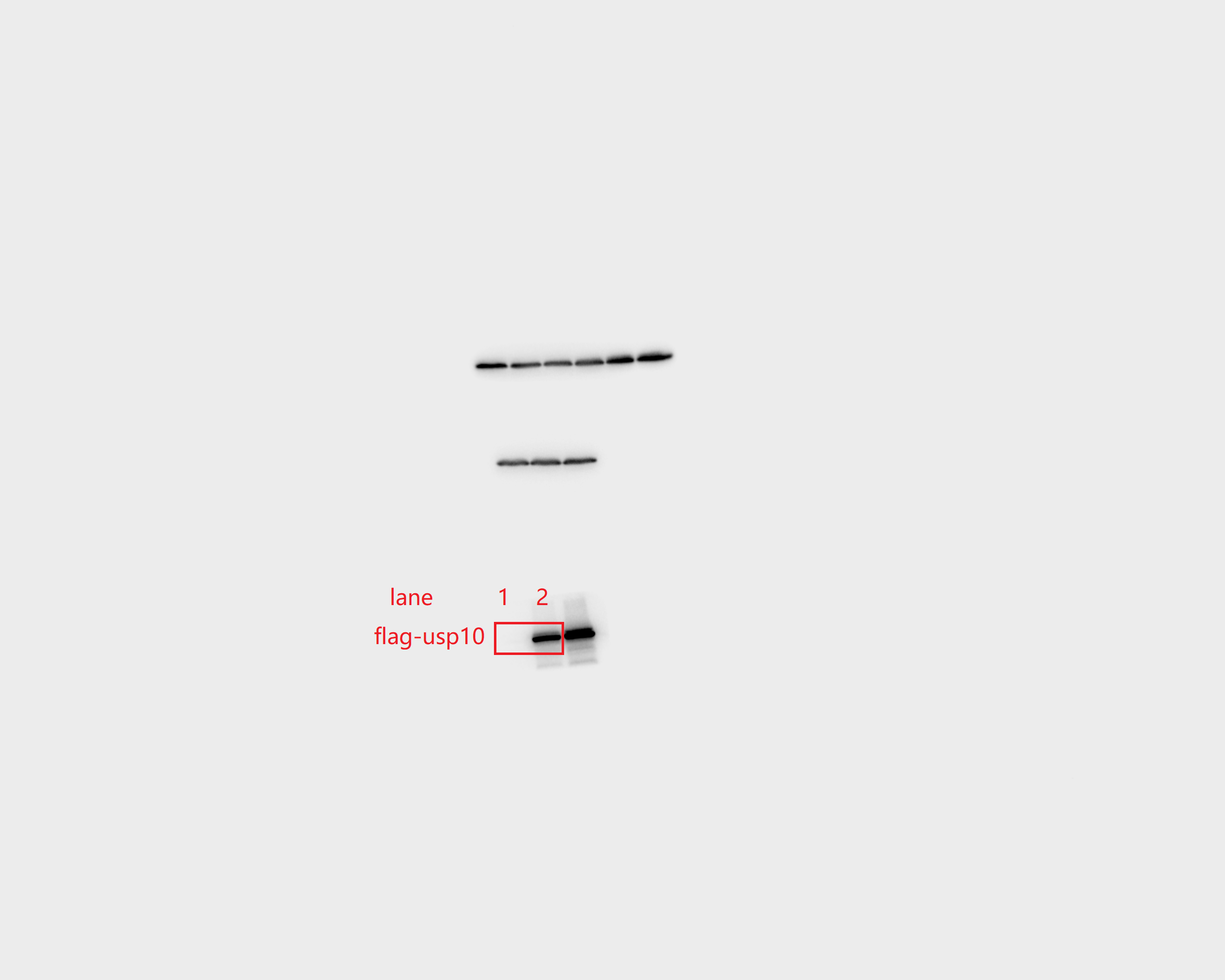

Supplement: Figure 5—source data 1. [file elife-101973-fig5-data1.zip › Figure 5–source data 1/Fig5D-labeled/flag-usp10.tif]

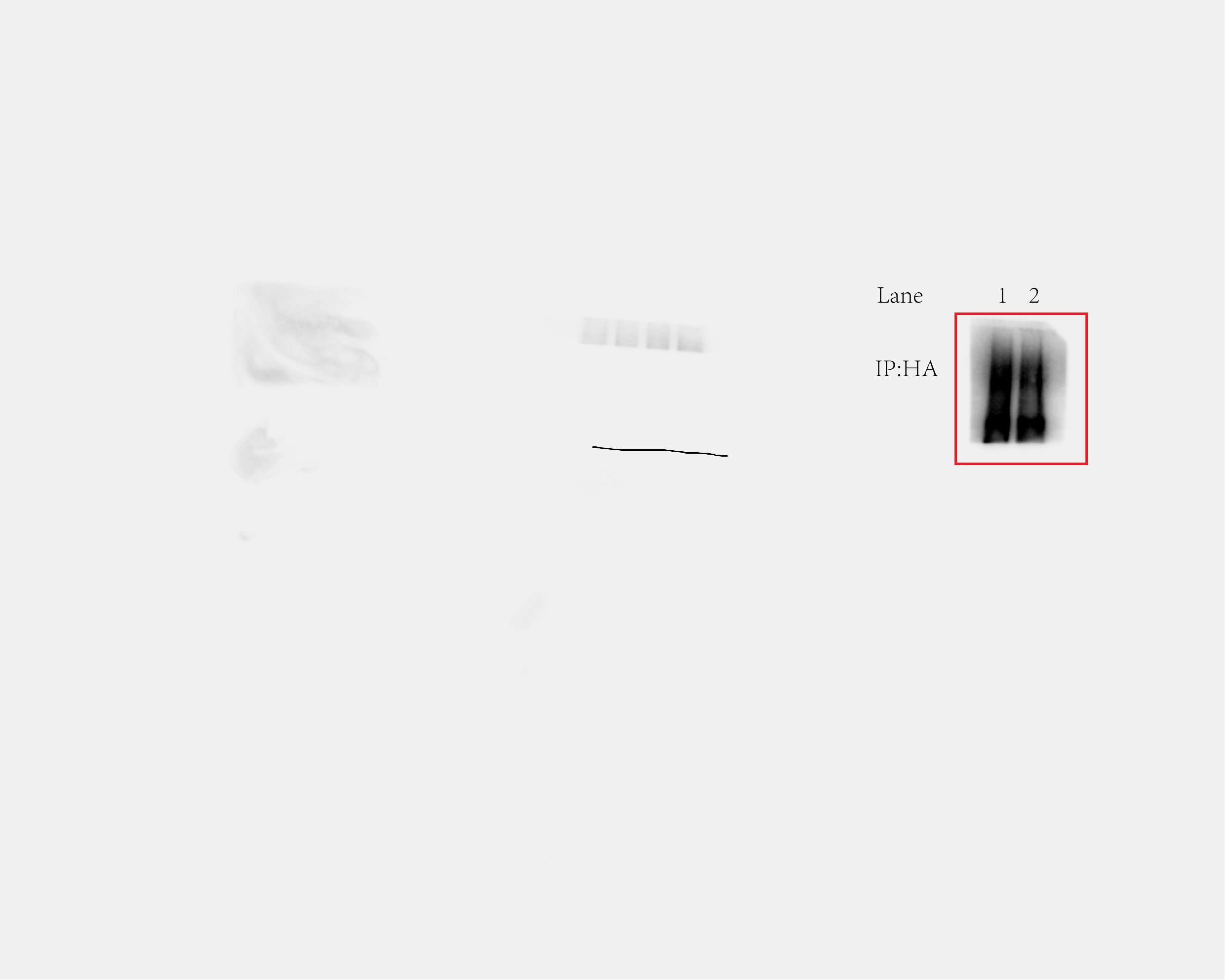

Supplement: Figure 5—source data 1. [file elife-101973-fig5-data1.zip › Figure 5–source data 1/Fig5F-labeled/IP HA.tif]

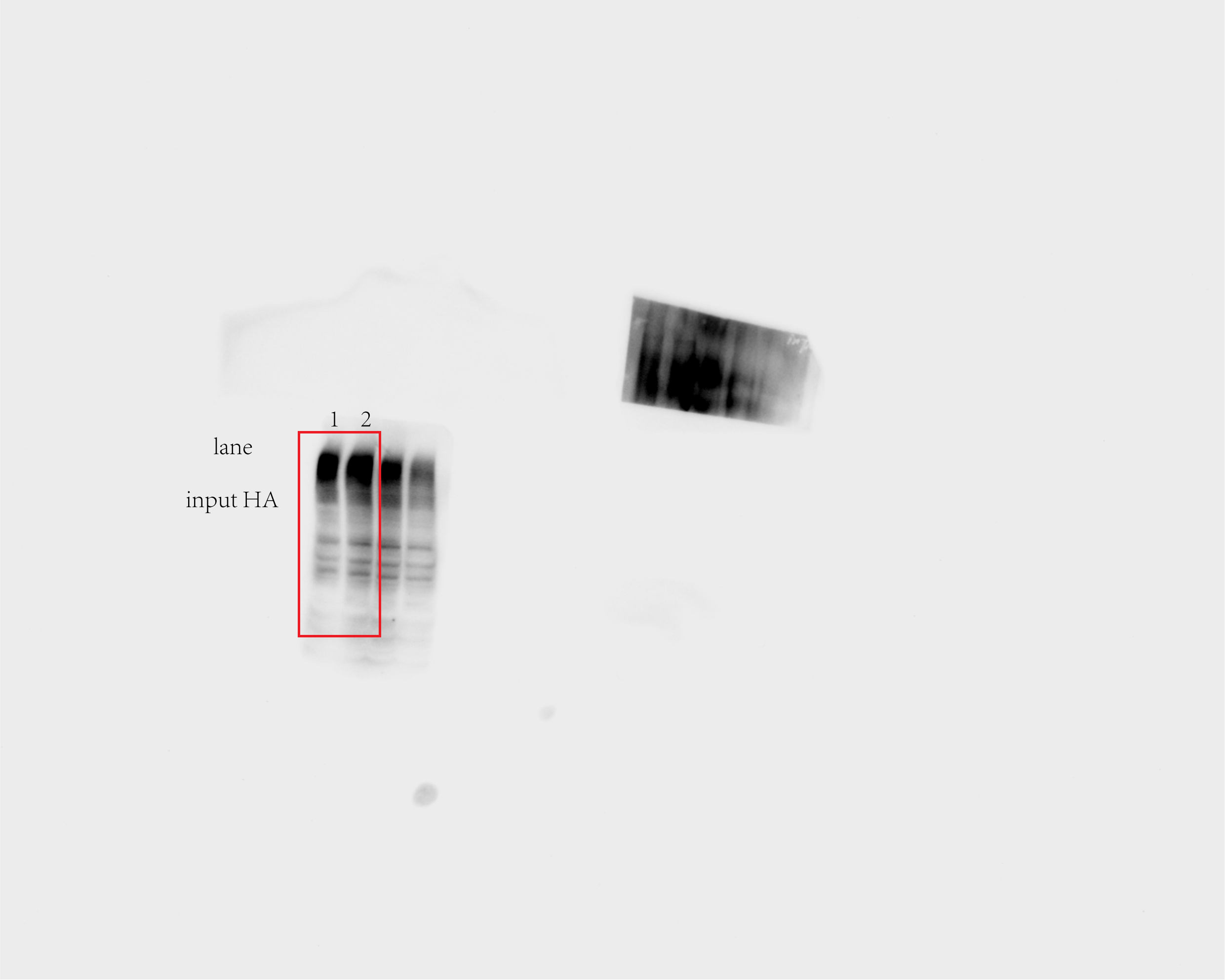

Supplement: Figure 5—source data 1. [file elife-101973-fig5-data1.zip › Figure 5–source data 1/Fig5F-labeled/input HA.tif]

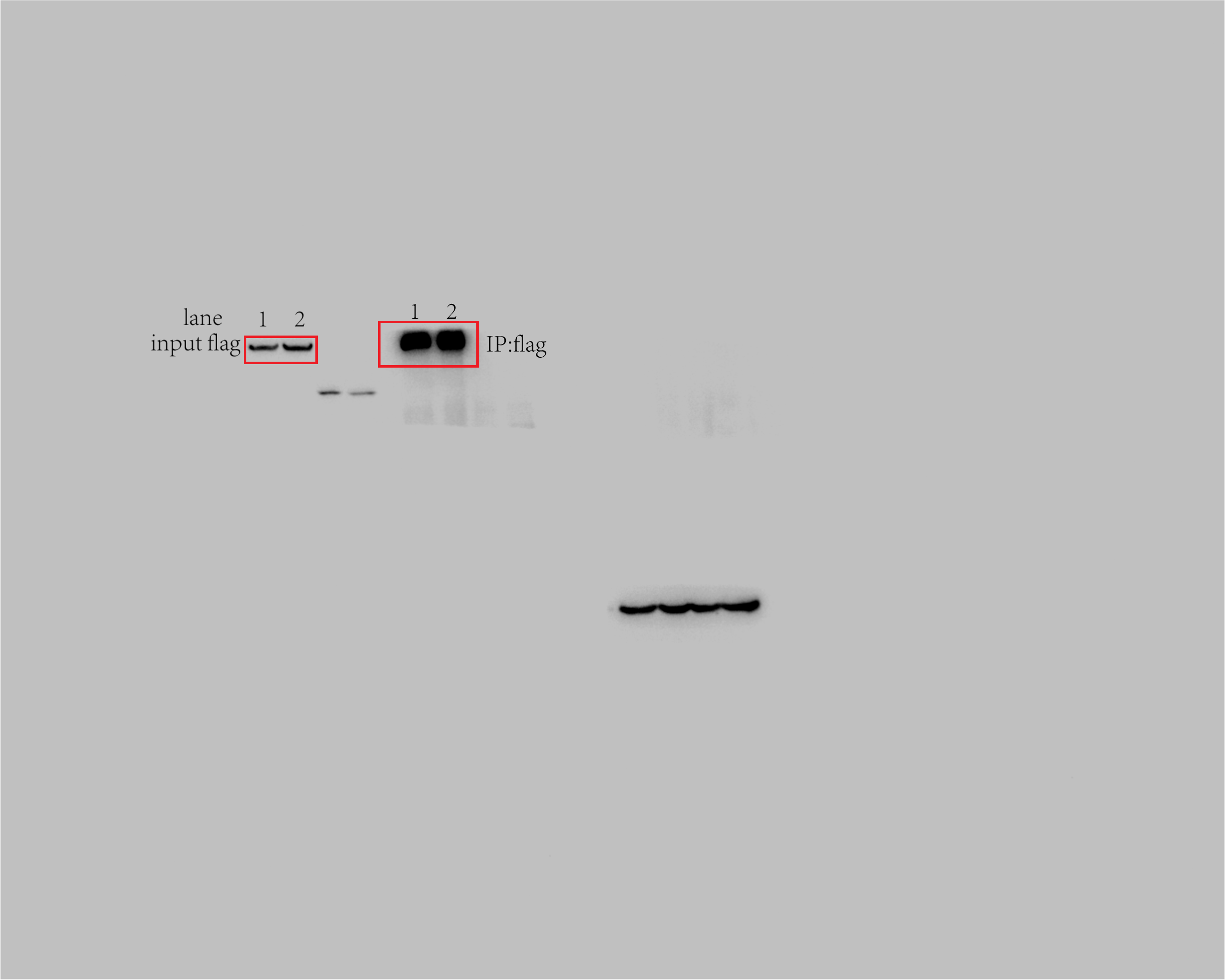

Supplement: Figure 5—source data 1. [file elife-101973-fig5-data1.zip › Figure 5–source data 1/Fig5F-labeled/input flag and IP flag.tif]

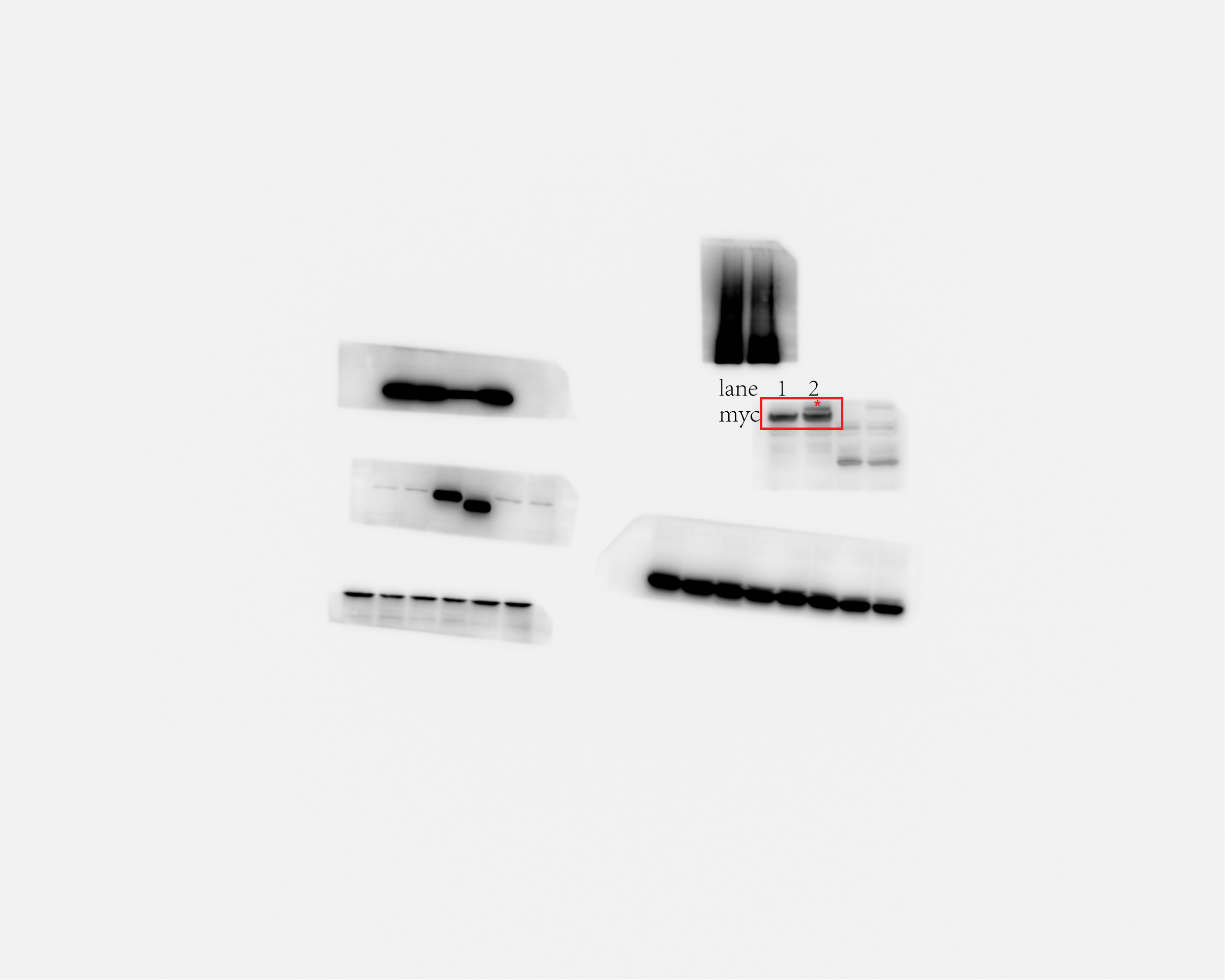

Supplement: Figure 5—source data 1. [file elife-101973-fig5-data1.zip › Figure 5–source data 1/Fig5F-labeled/input myc.tif]

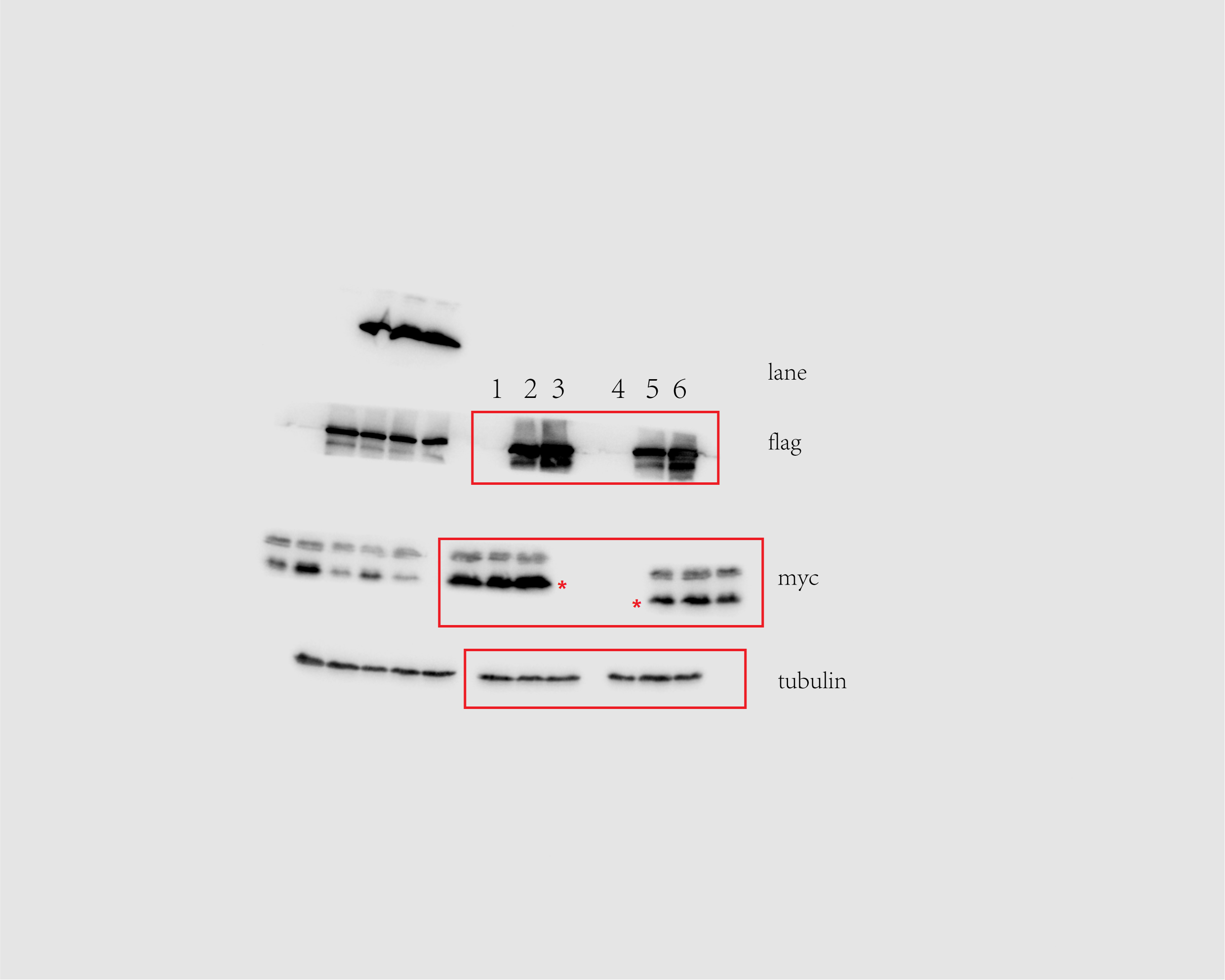

Supplement: Figure 5—source data 1. [file elife-101973-fig5-data1.zip › Figure 5–source data 1/Fig5G-labeled/Myc Flag and Tubulin.tif]

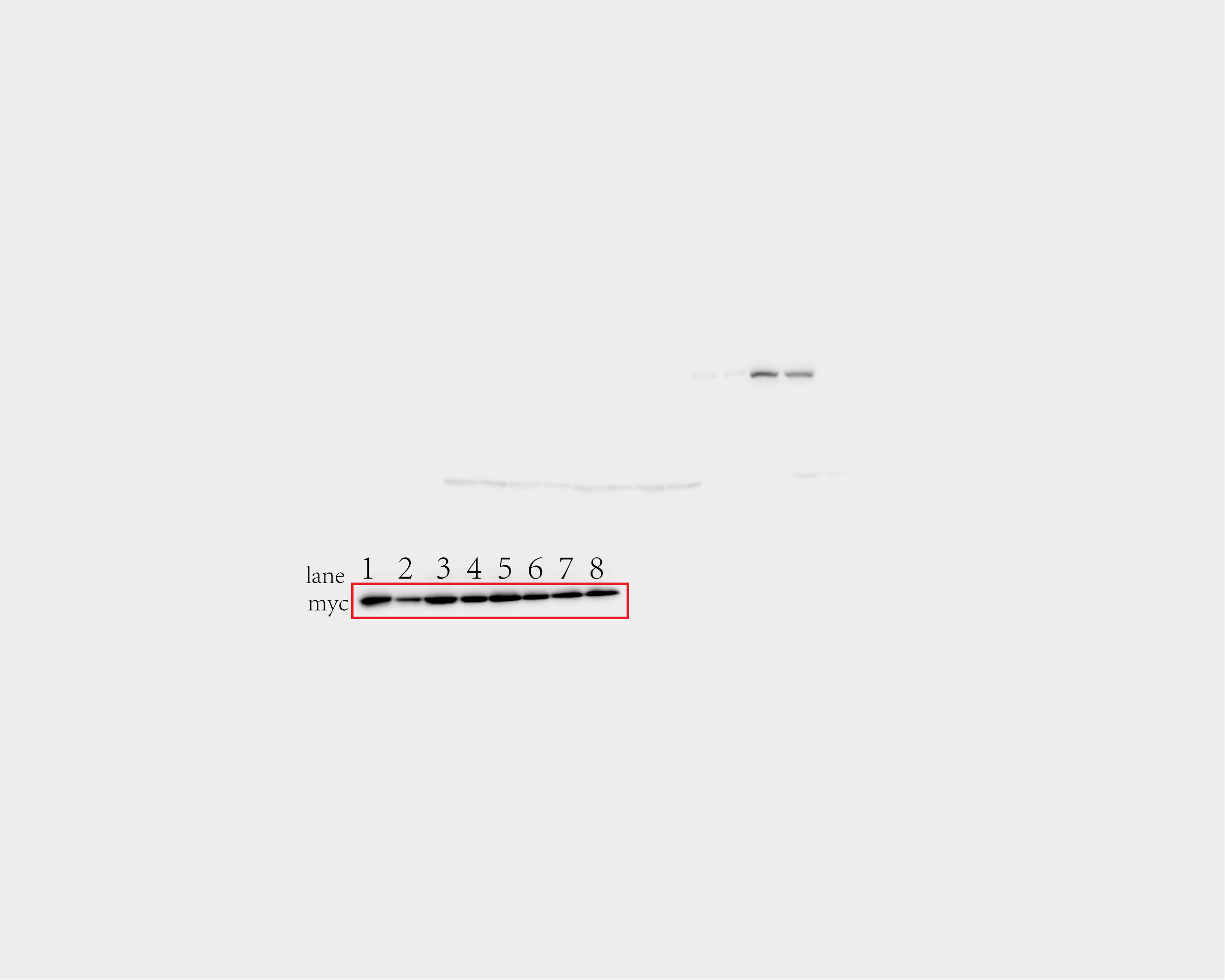

Supplement: Figure 5—source data 1. [file elife-101973-fig5-data1.zip › Figure 5–source data 1/Fig5H-labeled/myc.tif]

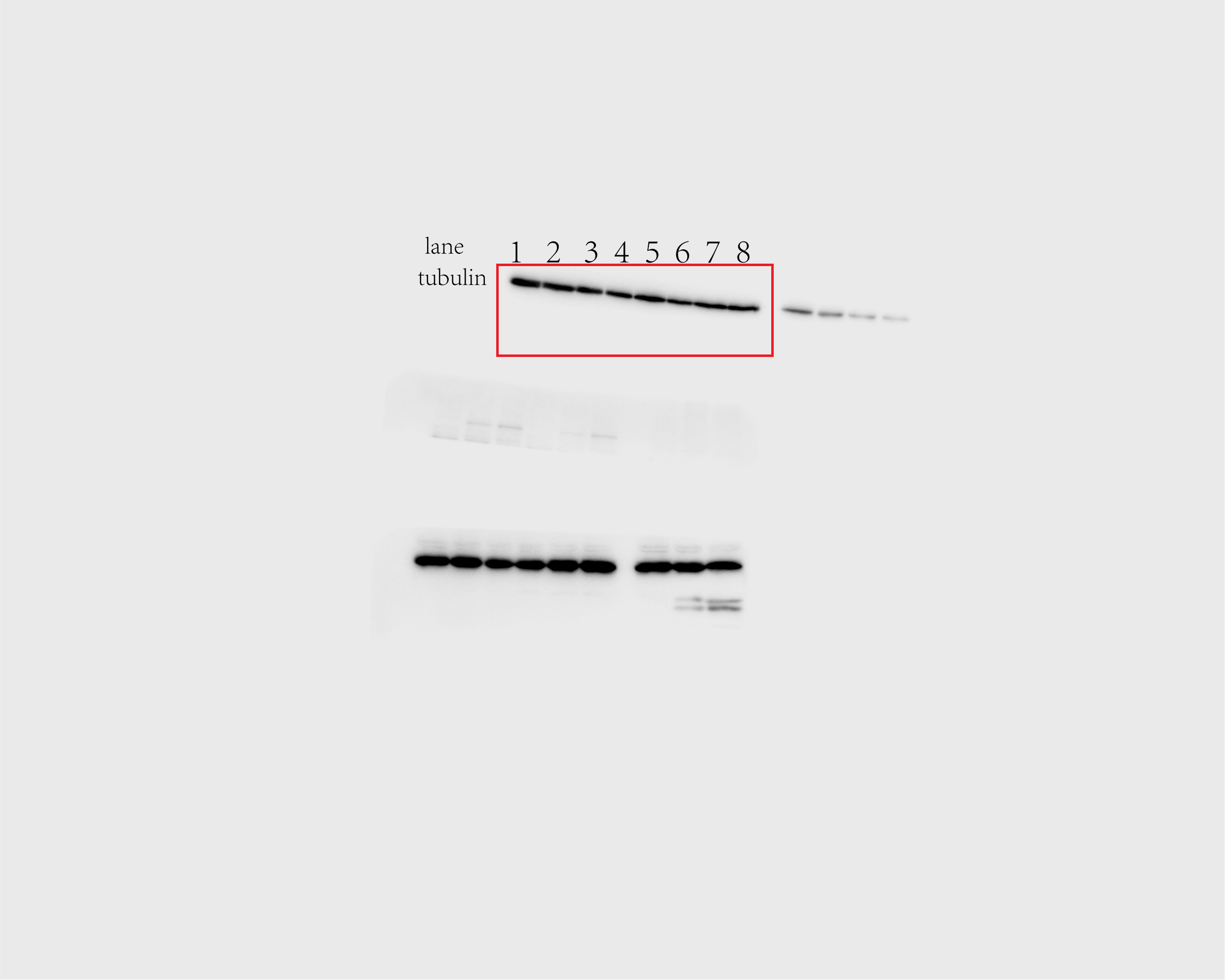

Supplement: Figure 5—source data 1. [file elife-101973-fig5-data1.zip › Figure 5–source data 1/Fig5H-labeled/tubulin.tif]

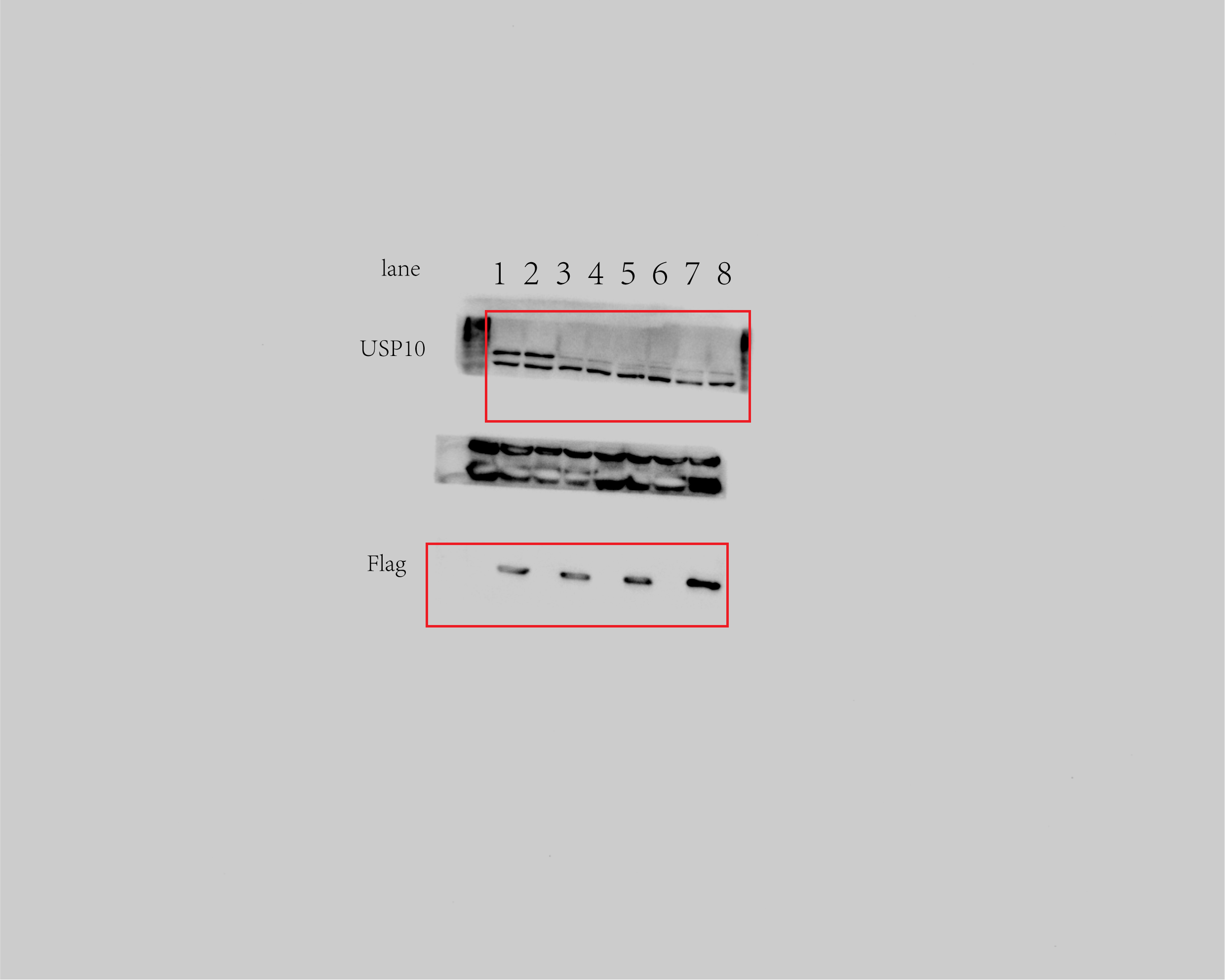

Supplement: Figure 5—source data 1. [file elife-101973-fig5-data1.zip › Figure 5–source data 1/Fig5H-labeled/usp10 and flag.tif]

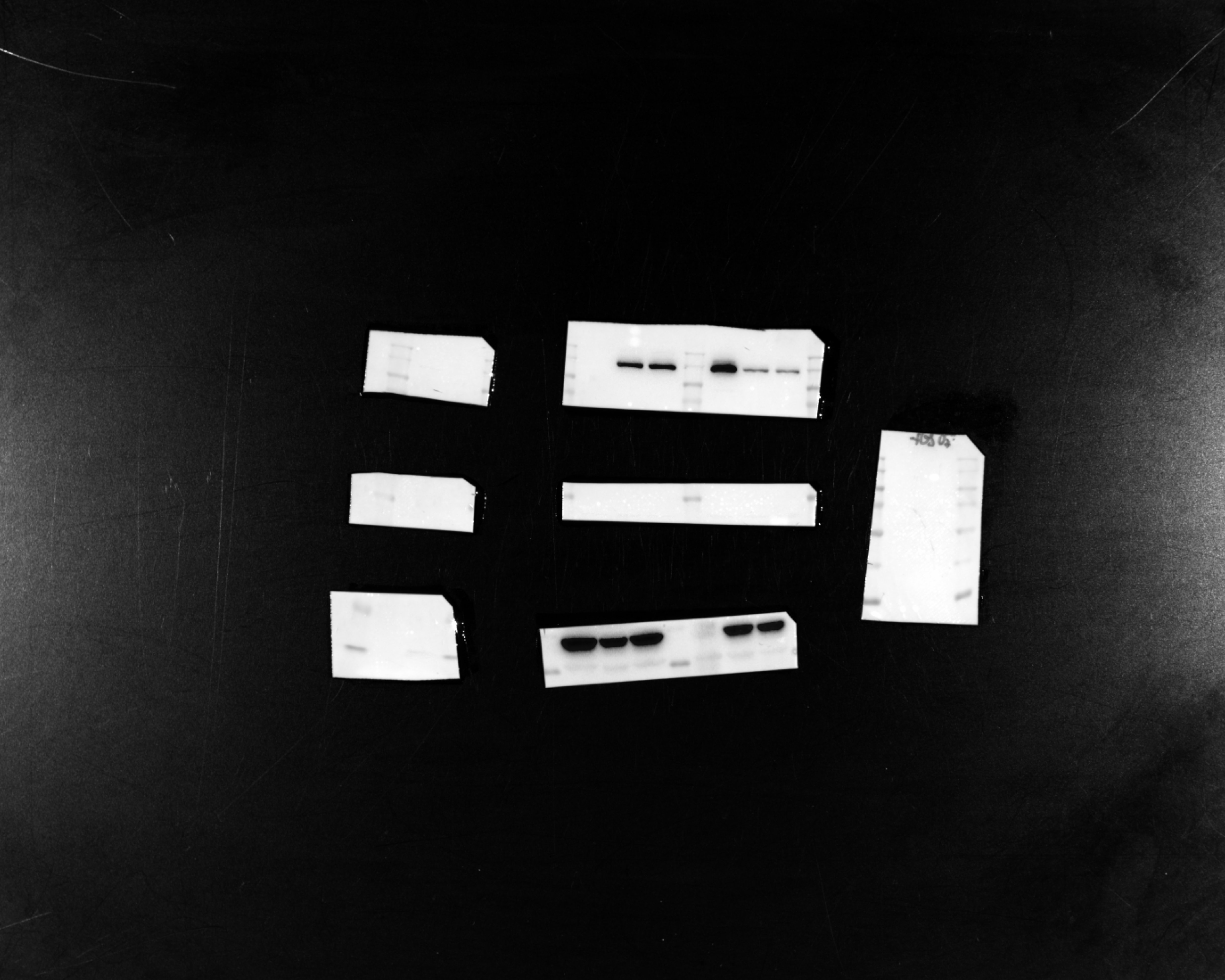

Supplement: Figure 5—source data 2. [file elife-101973-fig5-data2.zip › Figure 5–source data 2/figure 5B/input GFP Flag.jpg]

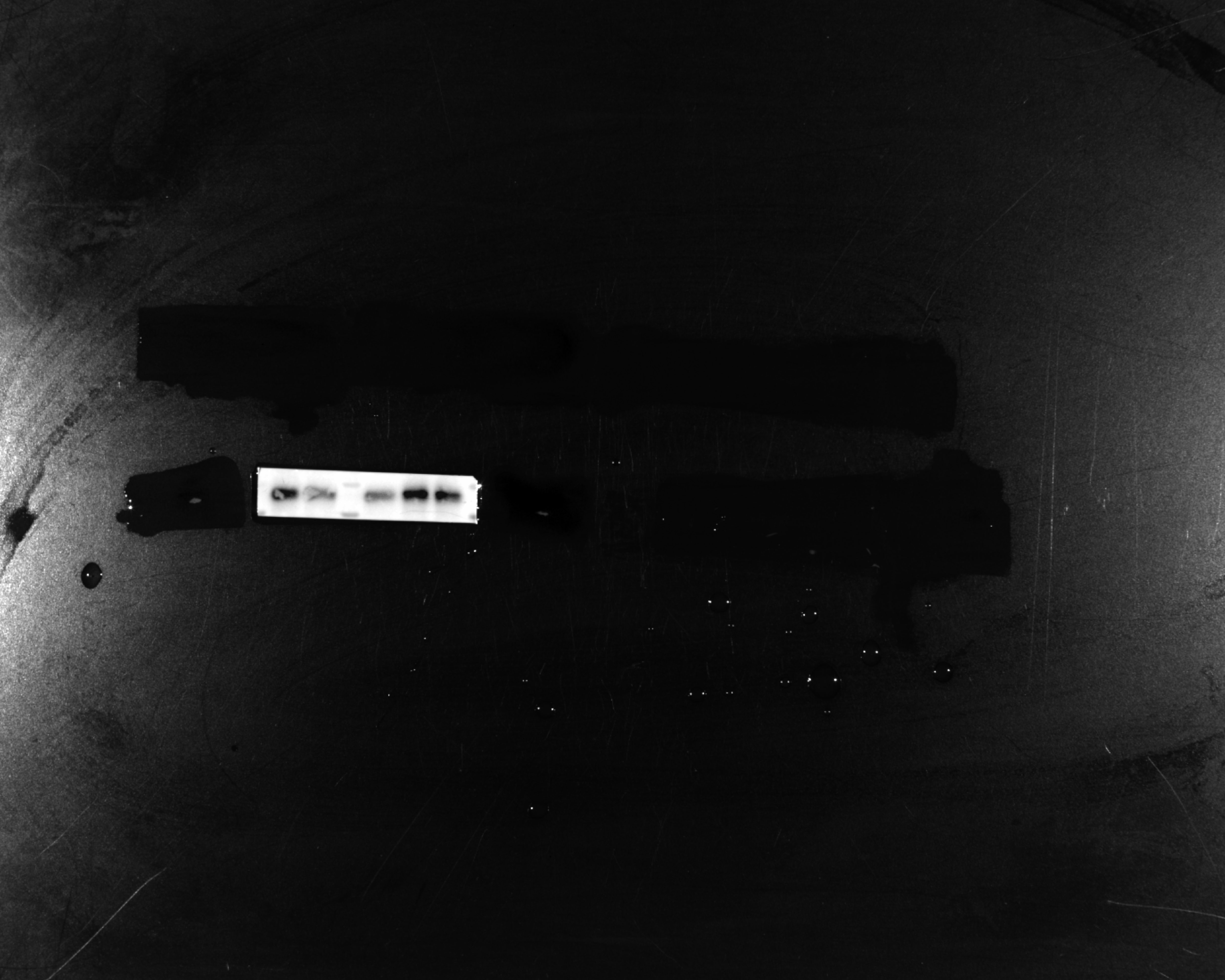

Supplement: Figure 5—source data 2. [file elife-101973-fig5-data2.zip › Figure 5–source data 2/figure 5B/ip GFP.jpg]

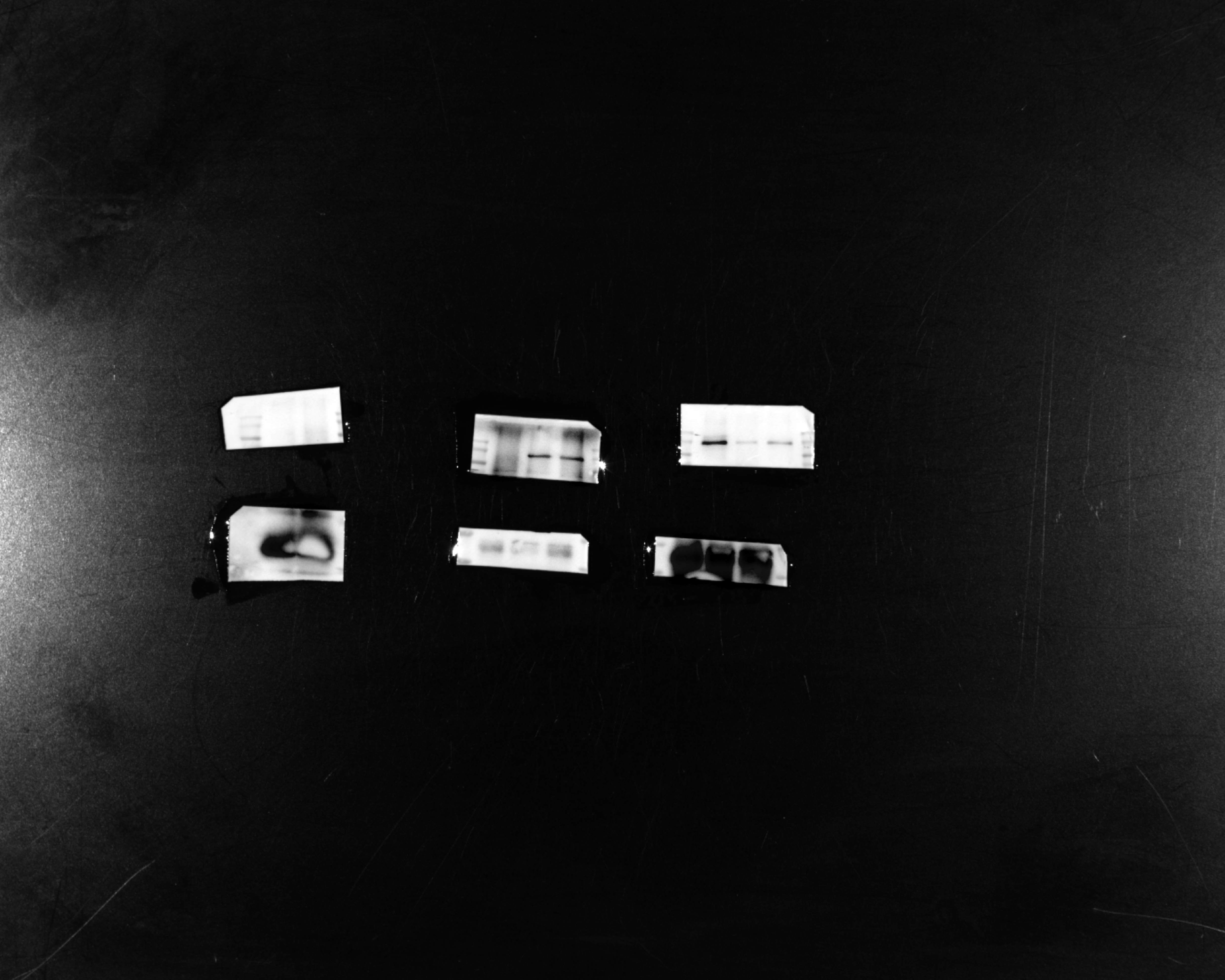

Supplement: Figure 5—source data 2. [file elife-101973-fig5-data2.zip › Figure 5–source data 2/figure 5B/ip flag.jpg]

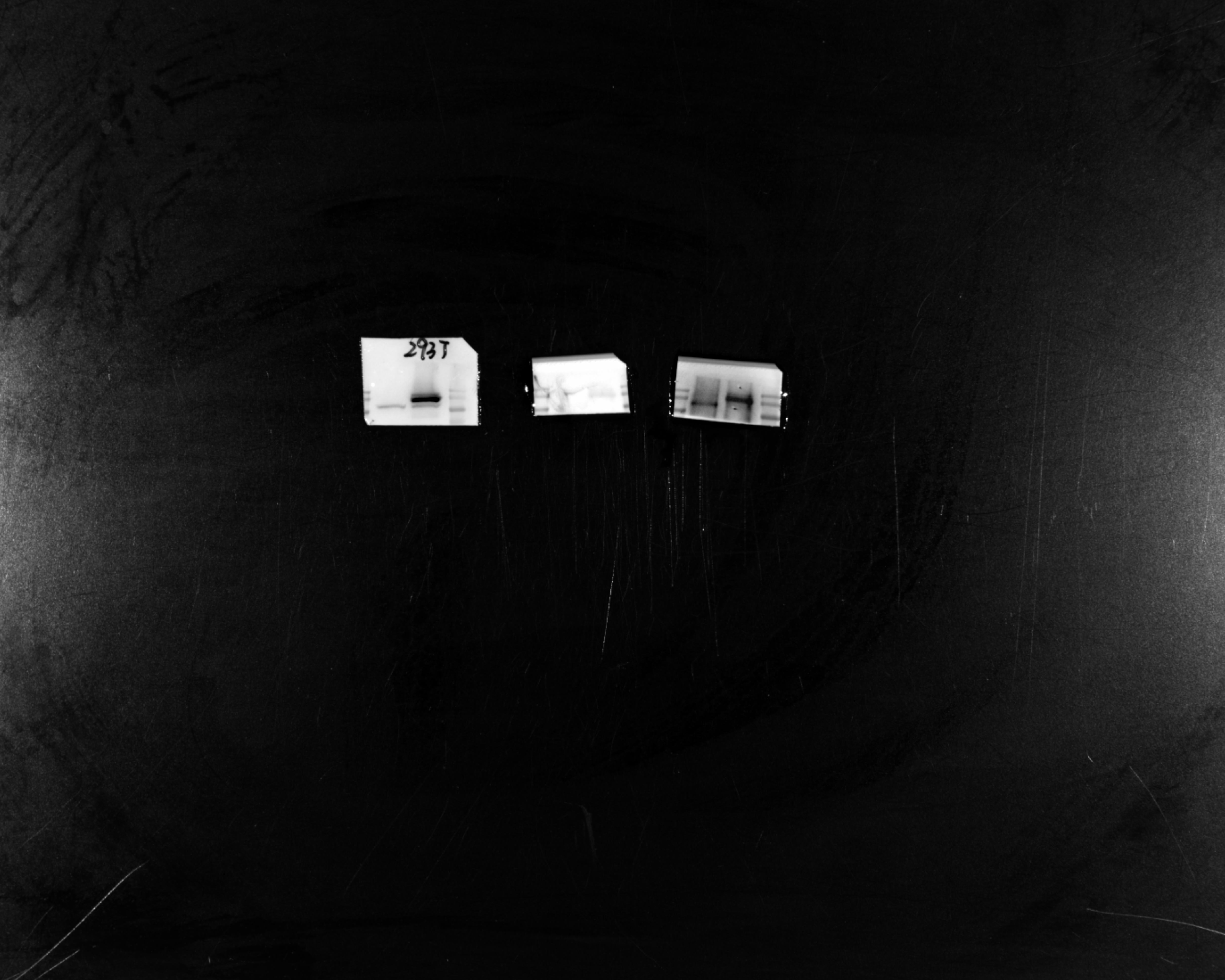

Supplement: Figure 5—source data 2. [file elife-101973-fig5-data2.zip › Figure 5–source data 2/figure 5C/input flag.jpg]

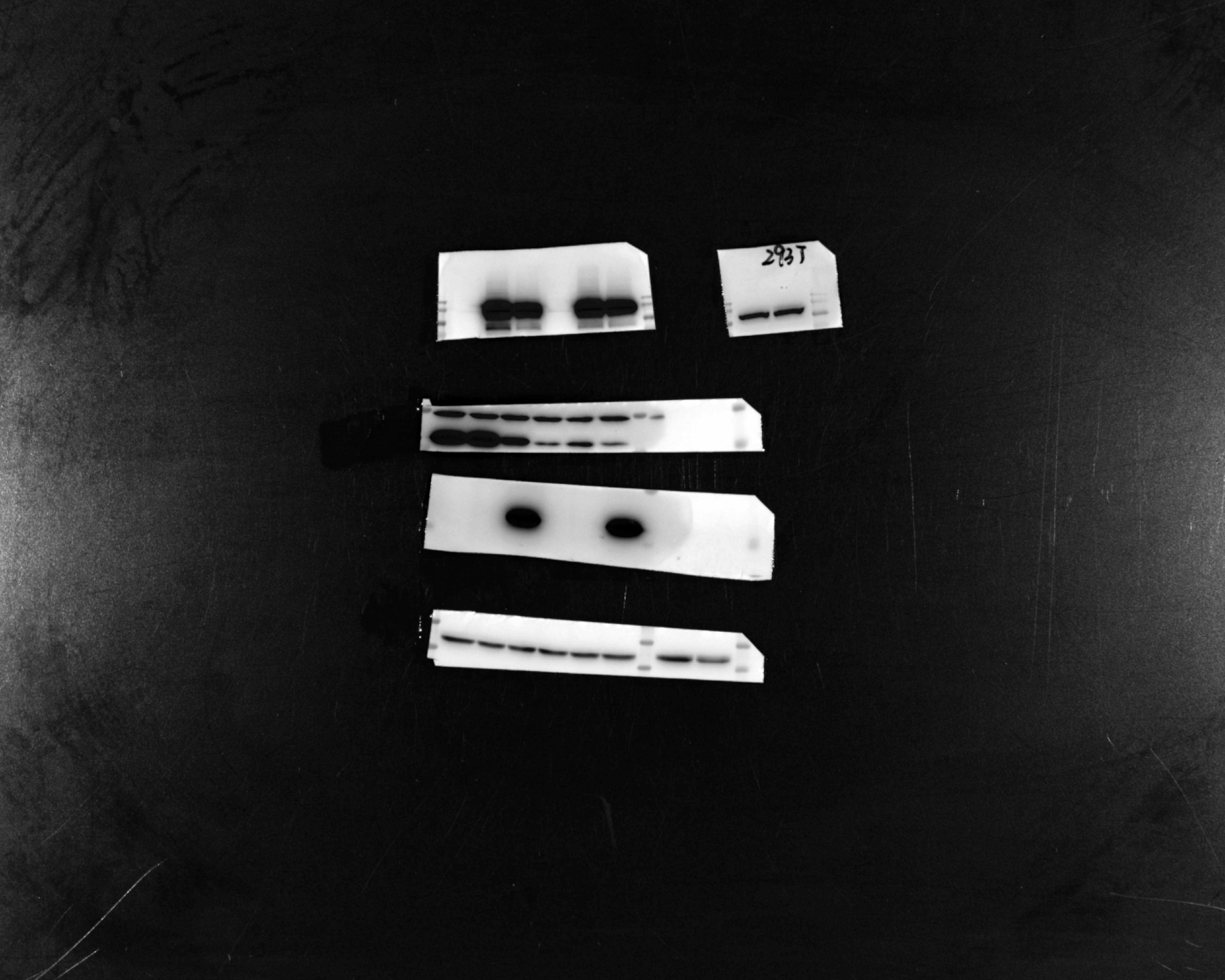

Supplement: Figure 5—source data 2. [file elife-101973-fig5-data2.zip › Figure 5–source data 2/figure 5C/input myc and tubulin.jpg]

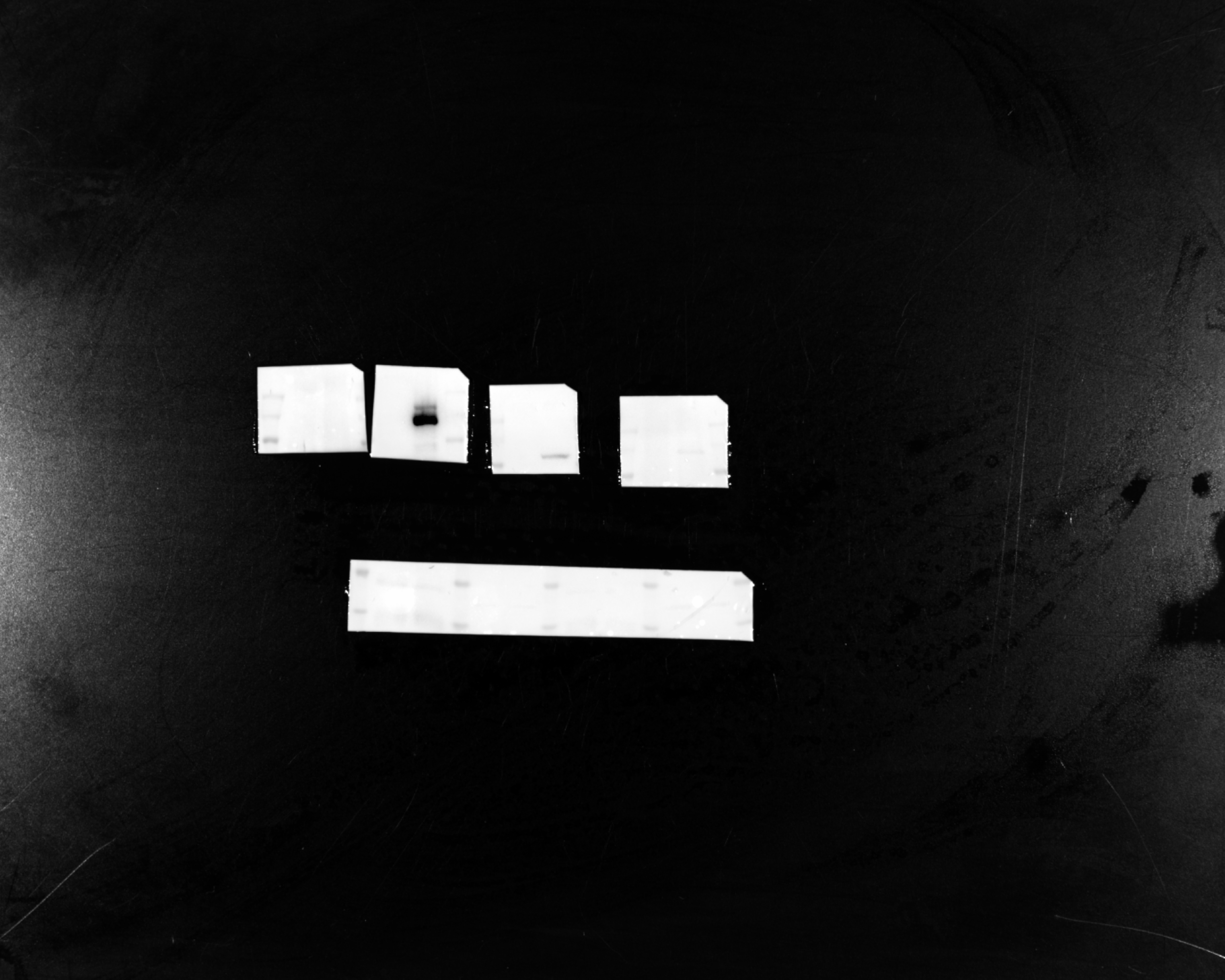

Supplement: Figure 5—source data 2. [file elife-101973-fig5-data2.zip › Figure 5–source data 2/figure 5C/ip flag.jpg]

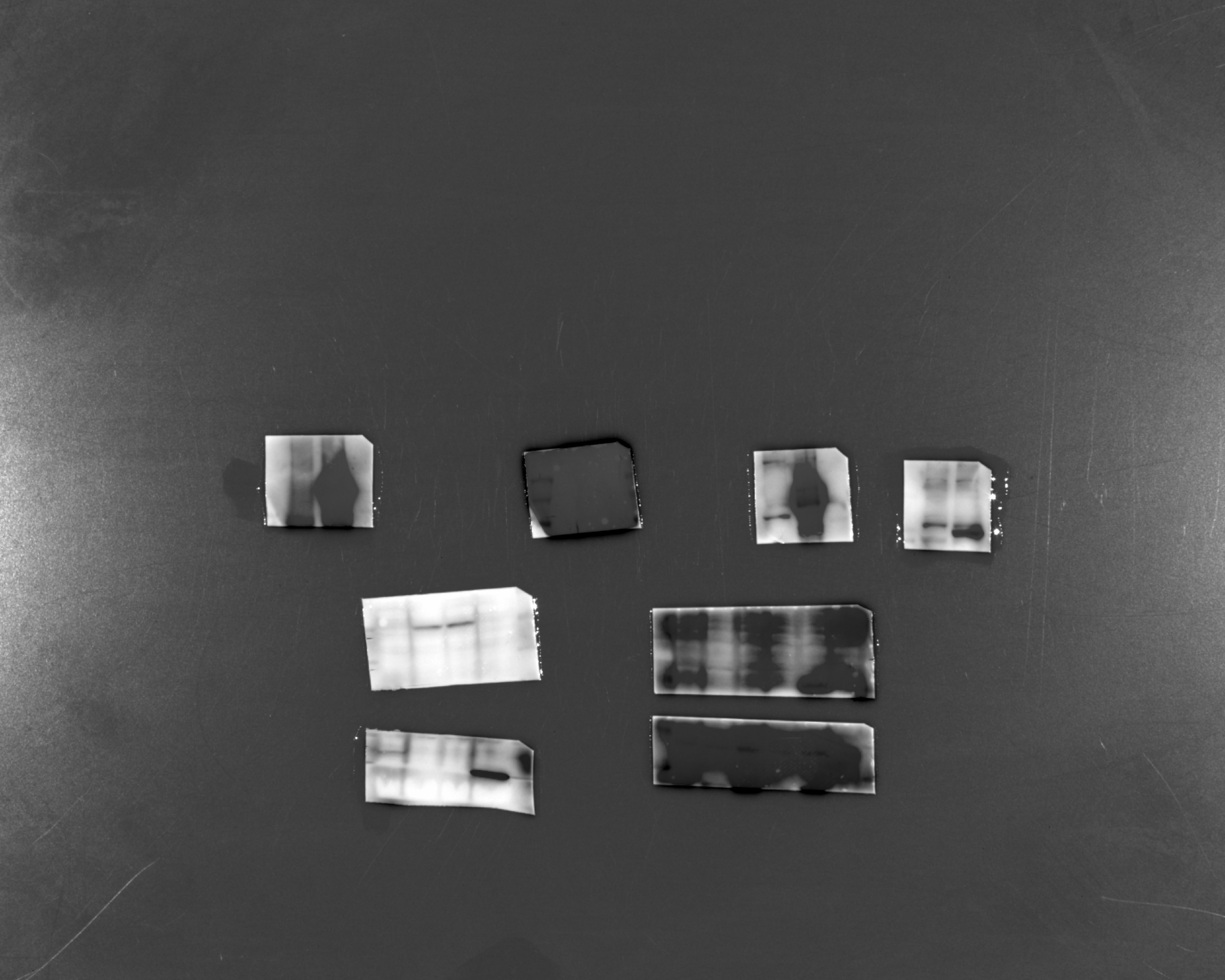

Supplement: Figure 5—source data 2. [file elife-101973-fig5-data2.zip › Figure 5–source data 2/figure 5C/ip myc.jpg]
