# Supplementary figures and images for: ORMDL3 restrains type I interferon signaling and anti-tumor immunity by promoting RIG-I degradation (part 3 of 3)
Source: eLife. 2025 Mar 24;13:RP101973. doi: 10.7554/eLife.101973 (PMC11932694; doi:10.7554/eLife.101973)

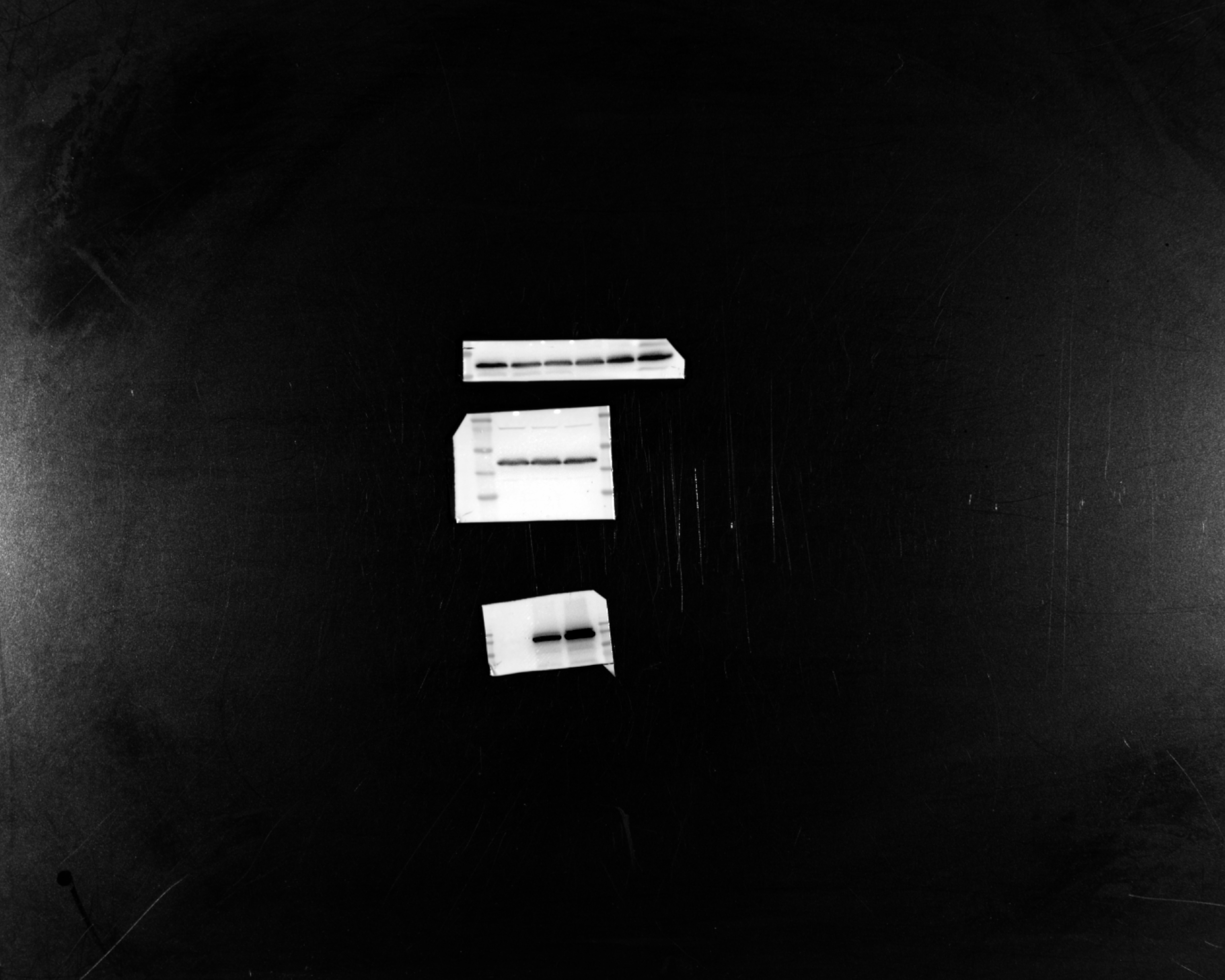

Supplement: Figure 5—source data 2. [file elife-101973-fig5-data2.zip › Figure 5–source data 2/figure 5D/Flag-USP10 .jpg]

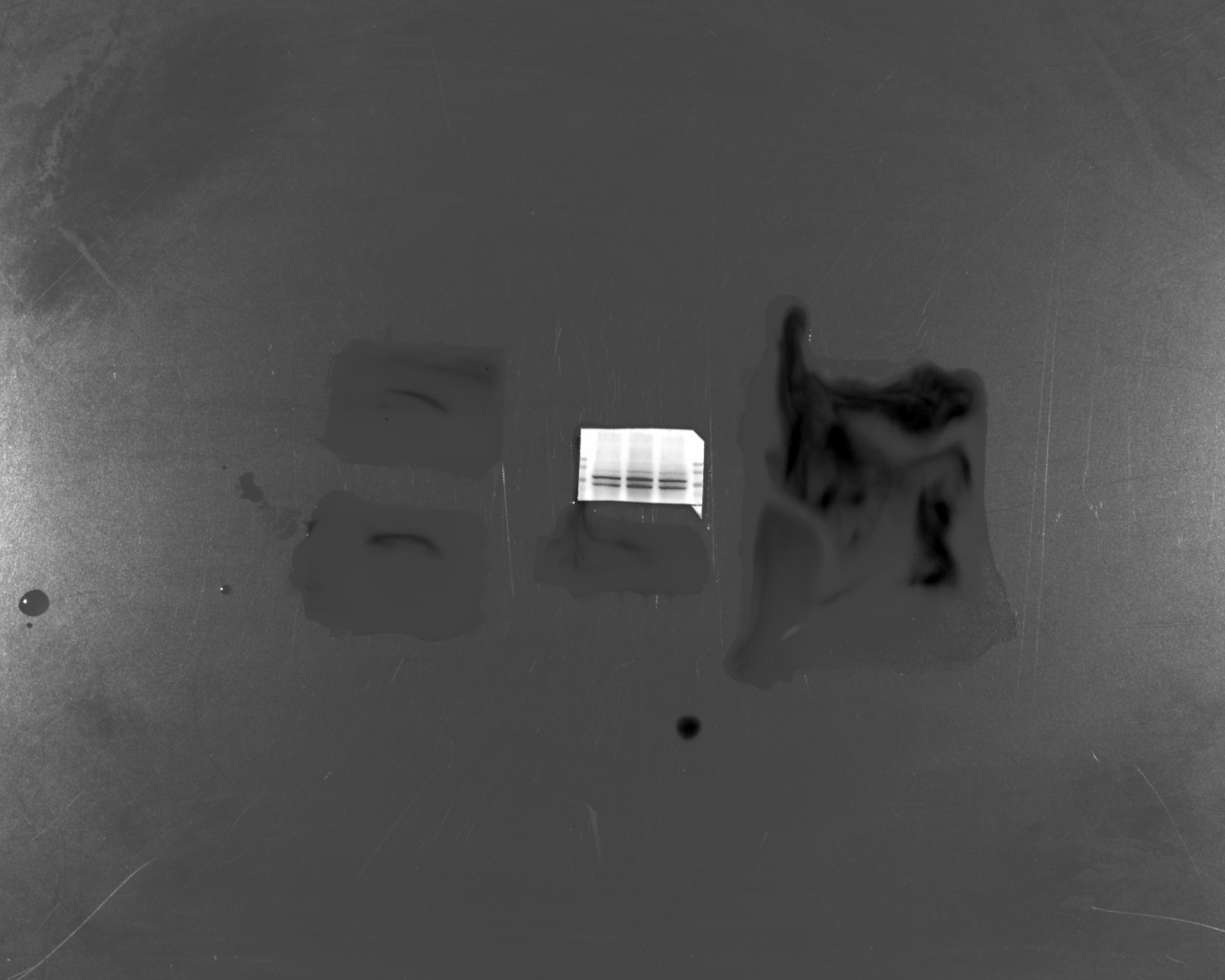

Supplement: Figure 5—source data 2. [file elife-101973-fig5-data2.zip › Figure 5–source data 2/figure 5D/RIG-I.jpg]

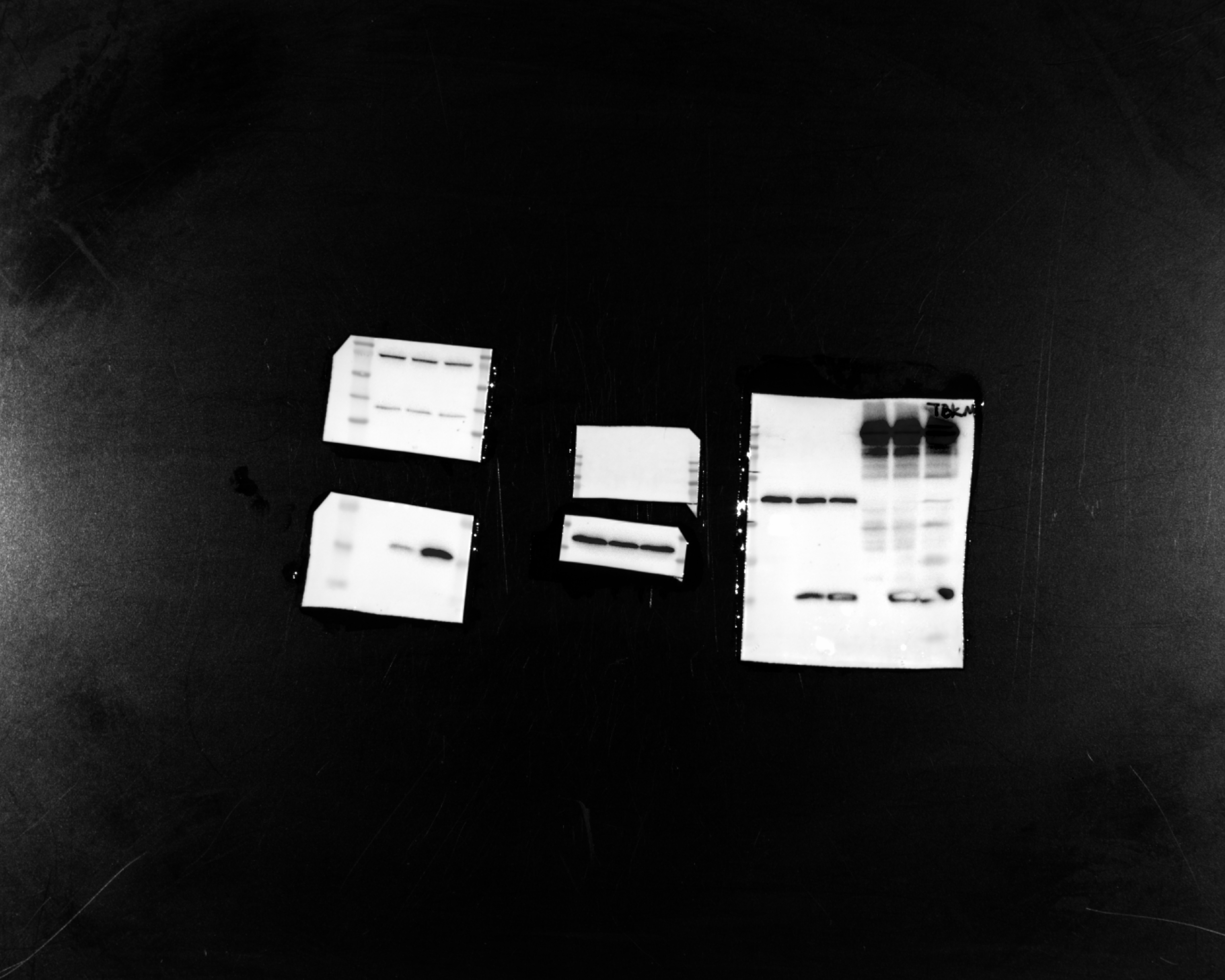

Supplement: Figure 5—source data 2. [file elife-101973-fig5-data2.zip › Figure 5–source data 2/figure 5D/tubulin.jpg]

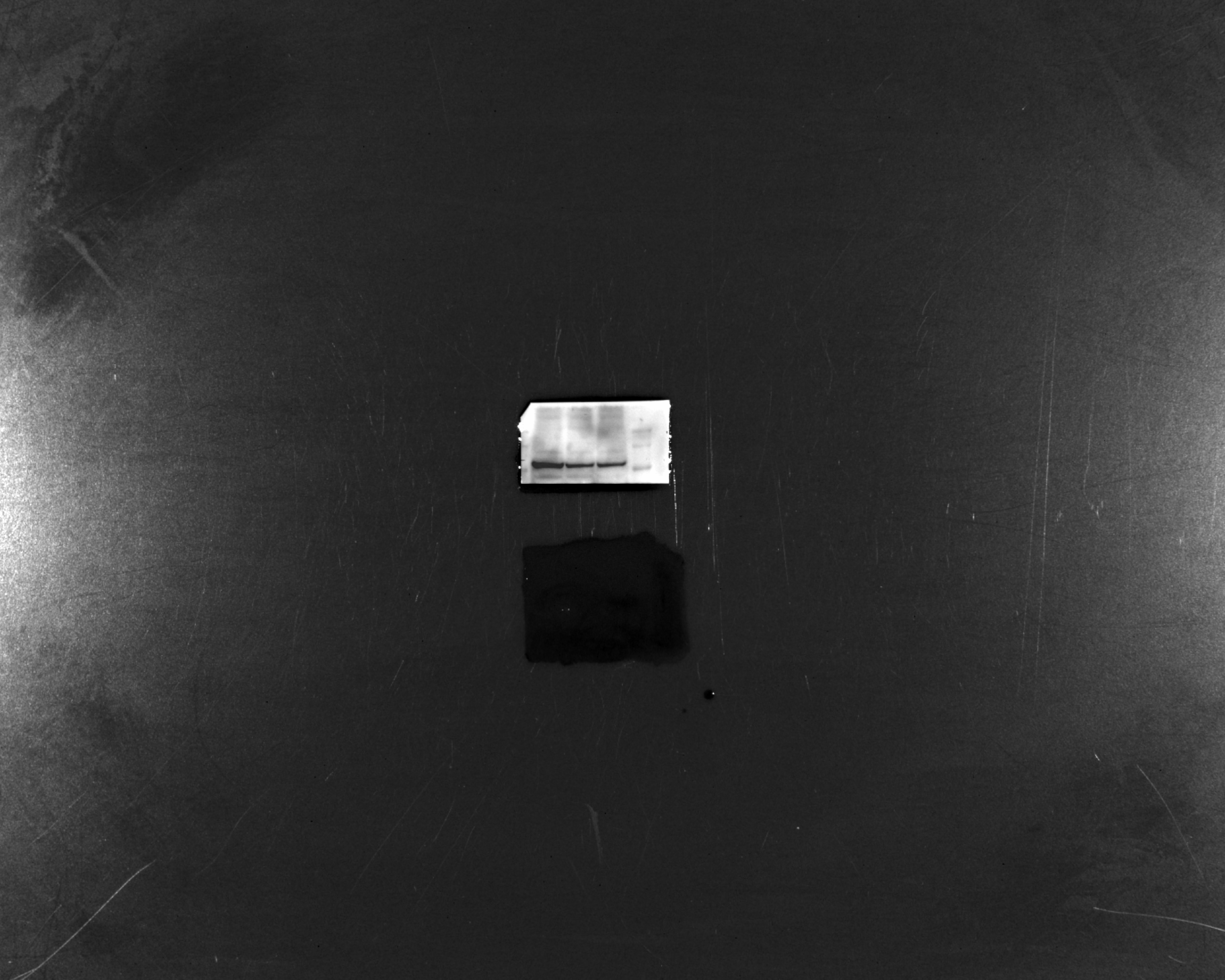

Supplement: Figure 5—source data 2. [file elife-101973-fig5-data2.zip › Figure 5–source data 2/figure 5E/RIG-I.jpg]

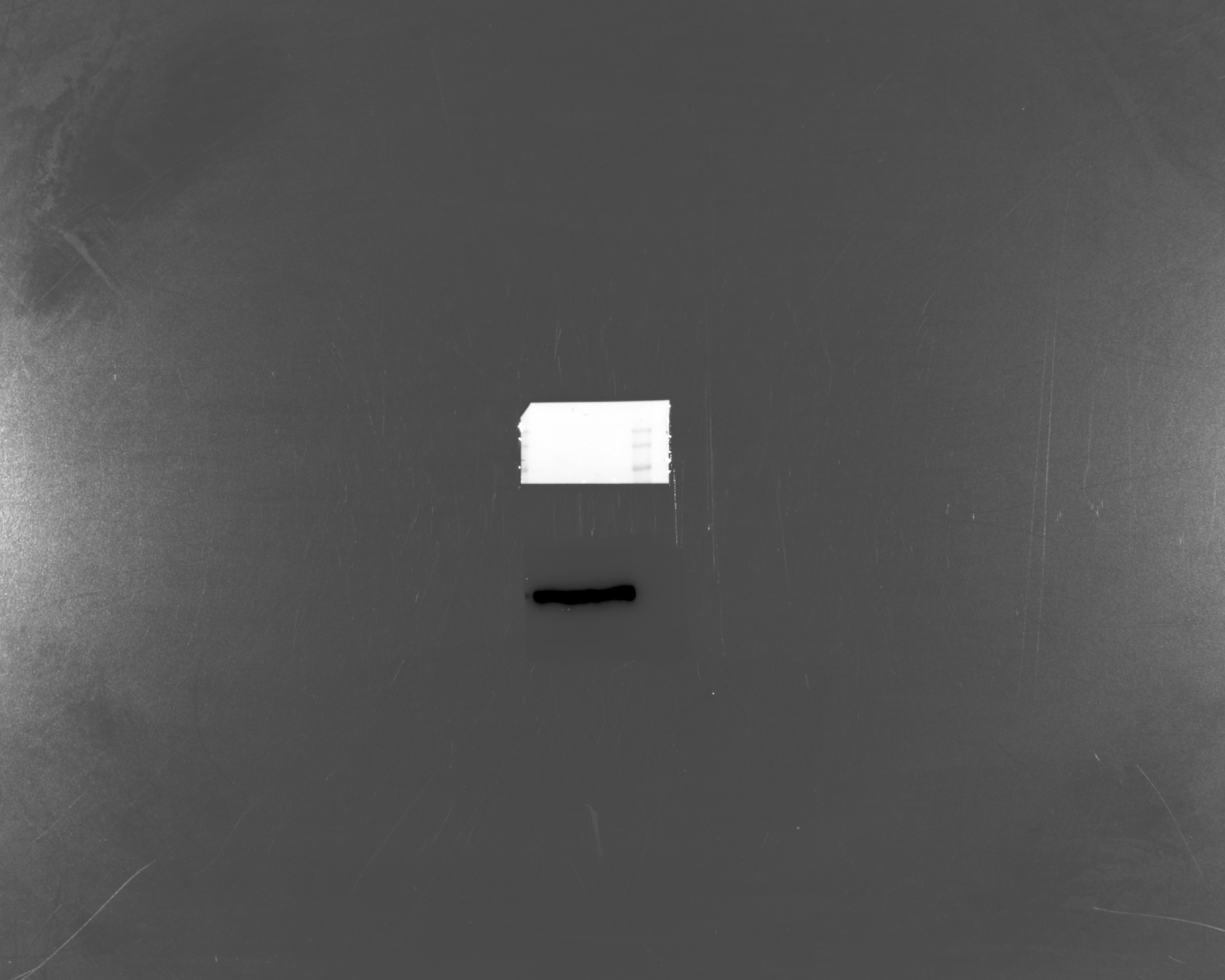

Supplement: Figure 5—source data 2. [file elife-101973-fig5-data2.zip › Figure 5–source data 2/figure 5E/Tubulin.jpg]

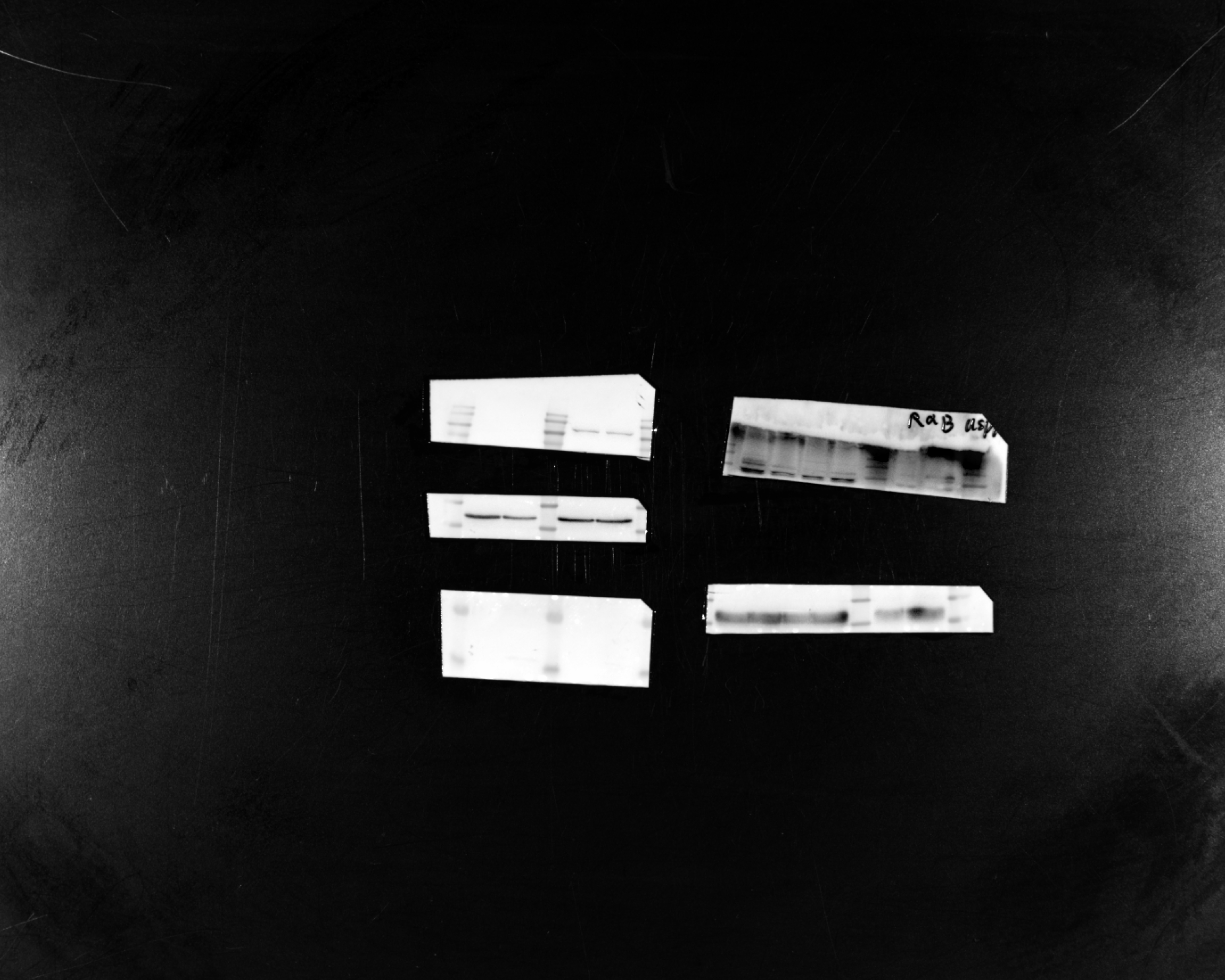

Supplement: Figure 5—source data 2. [file elife-101973-fig5-data2.zip › Figure 5–source data 2/figure 5E/USP10.jpg]

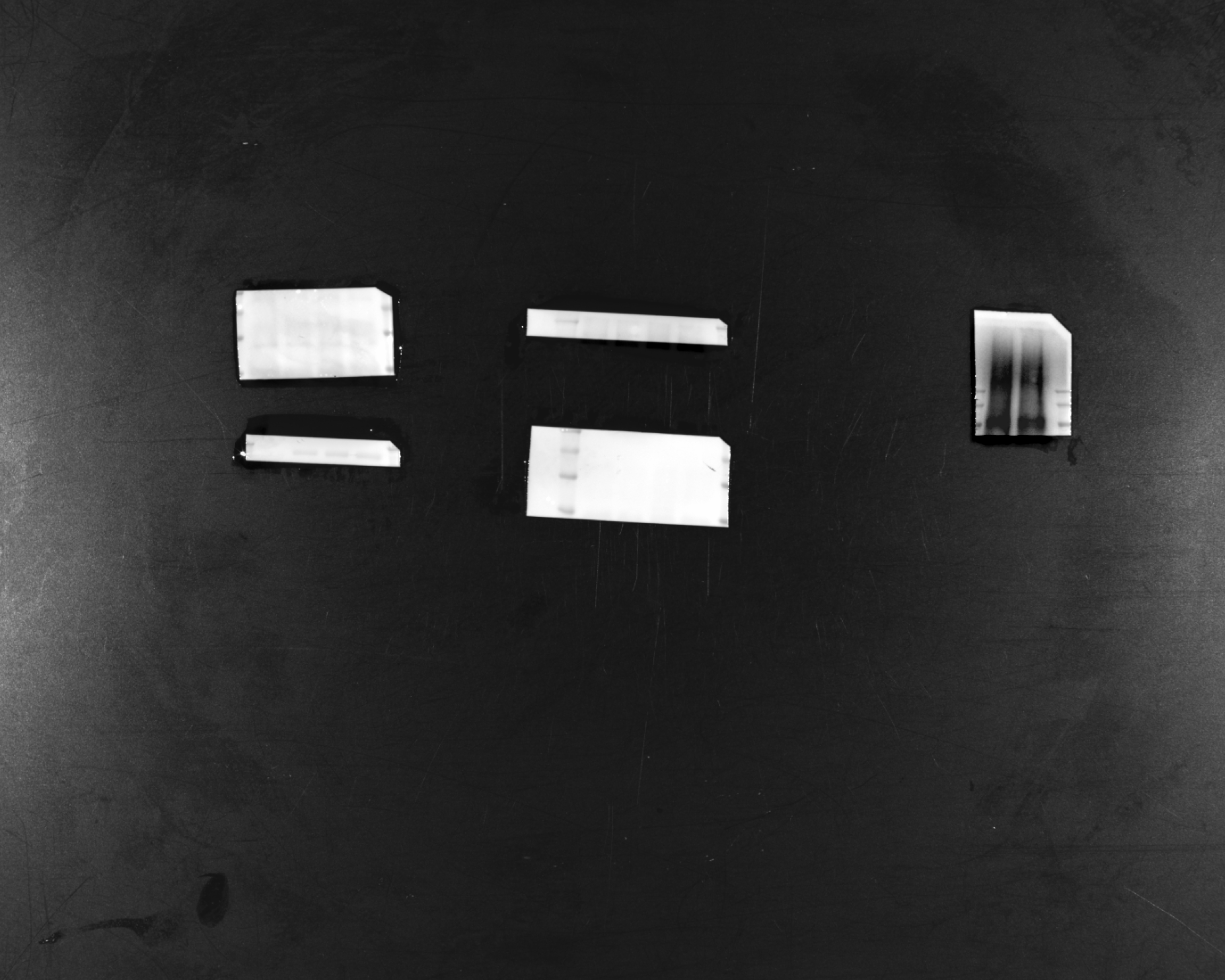

Supplement: Figure 5—source data 2. [file elife-101973-fig5-data2.zip › Figure 5–source data 2/figure 5F/IP HA.jpg]

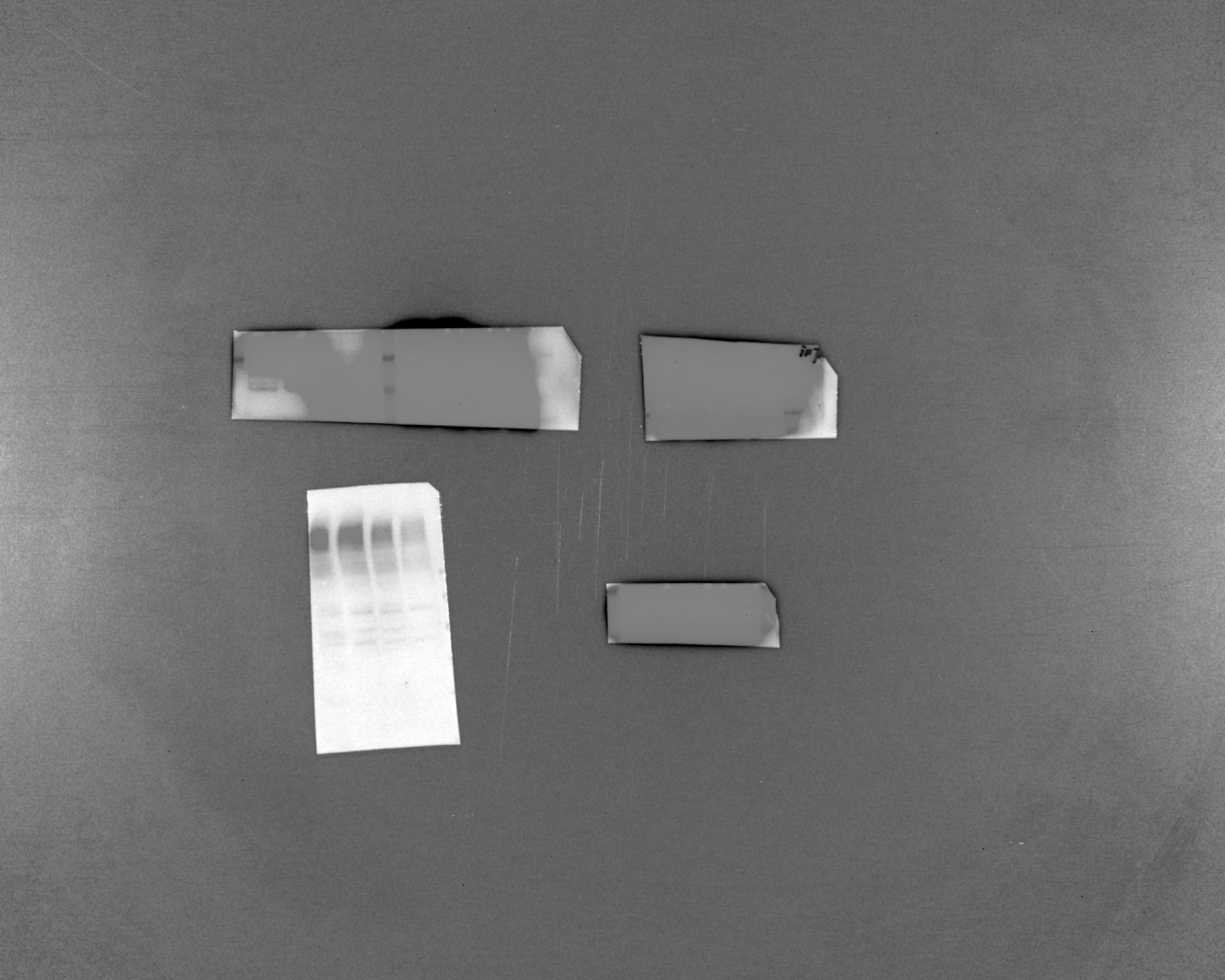

Supplement: Figure 5—source data 2. [file elife-101973-fig5-data2.zip › Figure 5–source data 2/figure 5F/input HA.jpg]

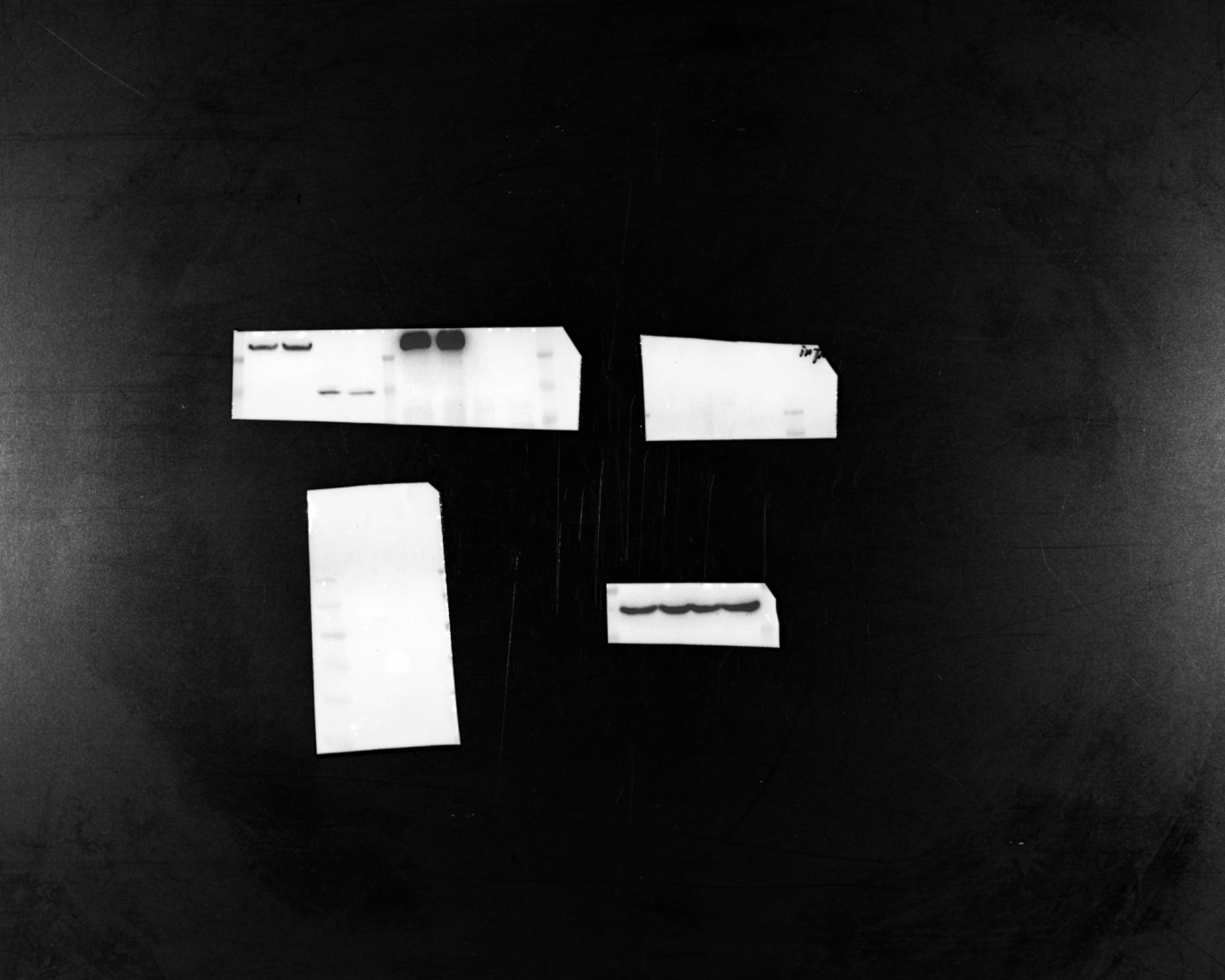

Supplement: Figure 5—source data 2. [file elife-101973-fig5-data2.zip › Figure 5–source data 2/figure 5F/input and ip flag.jpg]

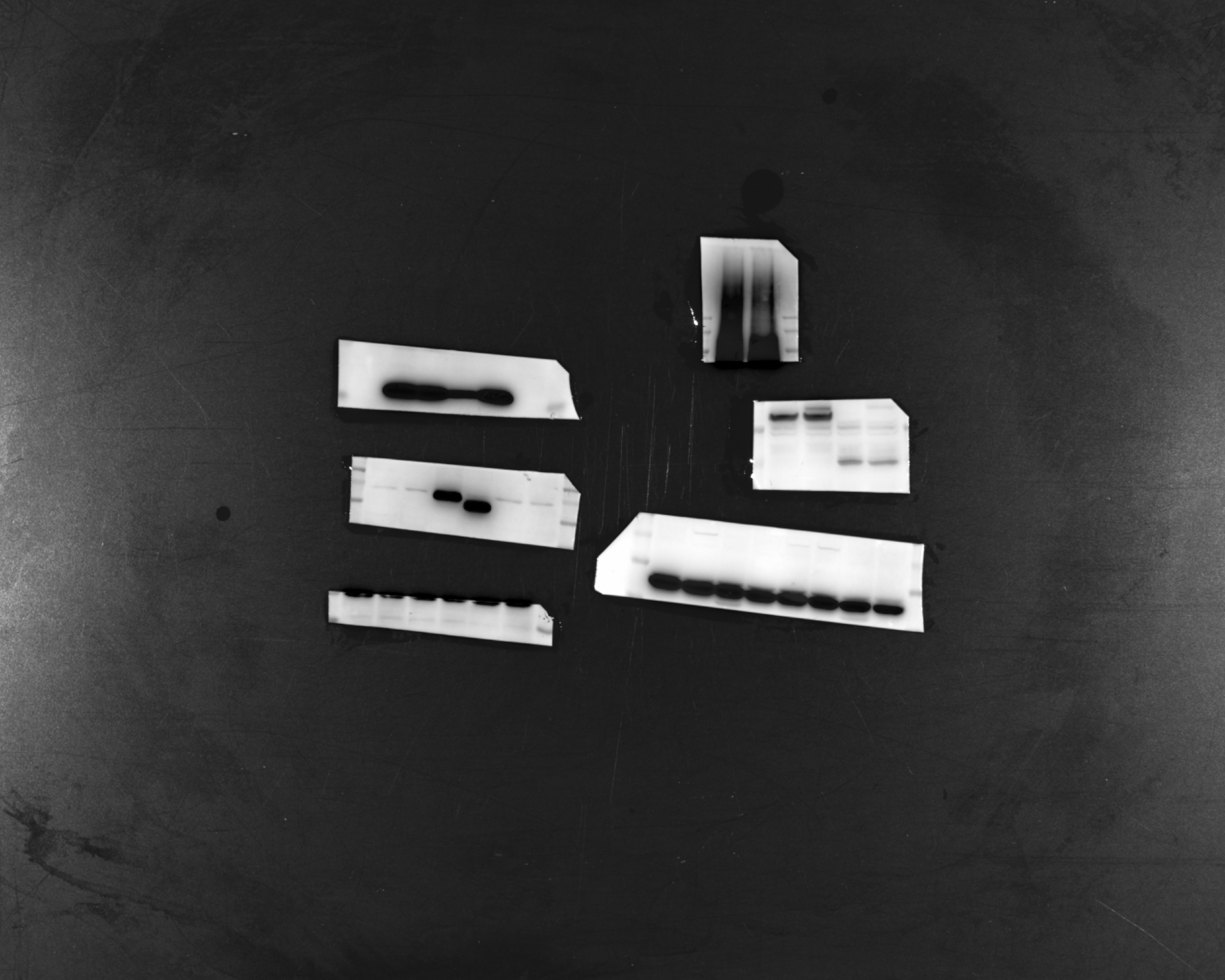

Supplement: Figure 5—source data 2. [file elife-101973-fig5-data2.zip › Figure 5–source data 2/figure 5F/input myc.jpg]

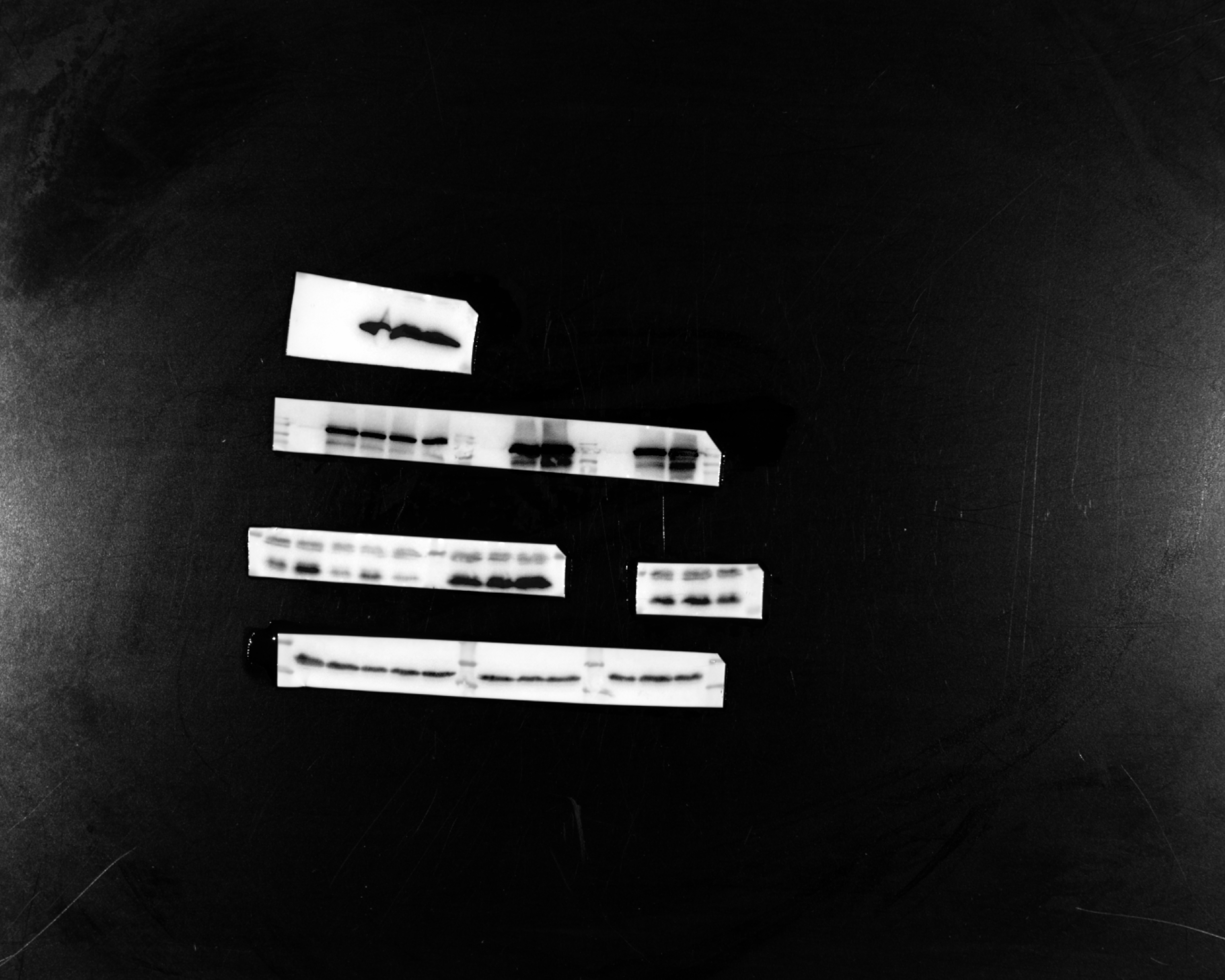

Supplement: Figure 5—source data 2. [file elife-101973-fig5-data2.zip › Figure 5–source data 2/figure 5G/Myc flag and tubulin.jpg]

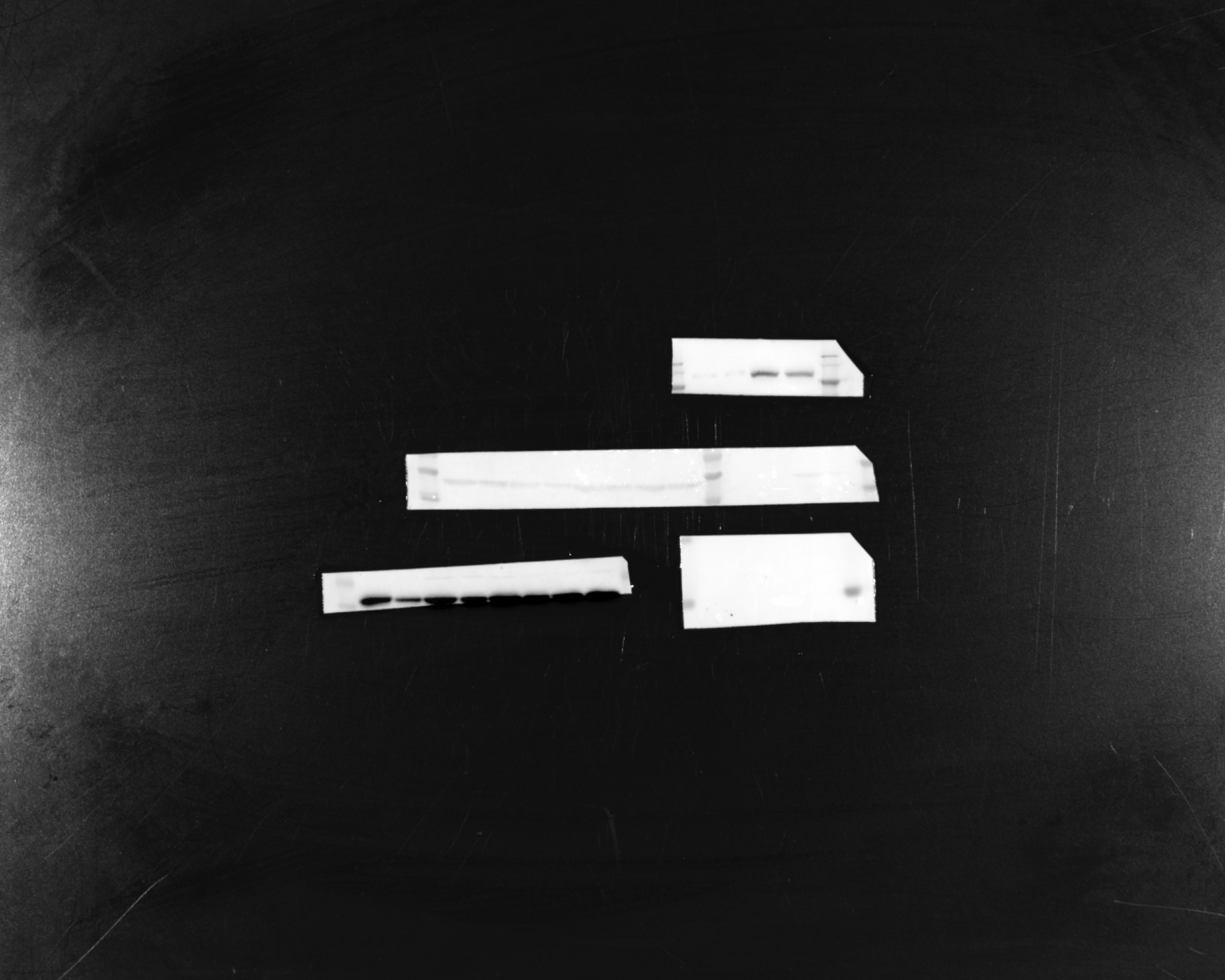

Supplement: Figure 5—source data 2. [file elife-101973-fig5-data2.zip › Figure 5–source data 2/figure 5H/Myc.jpg]

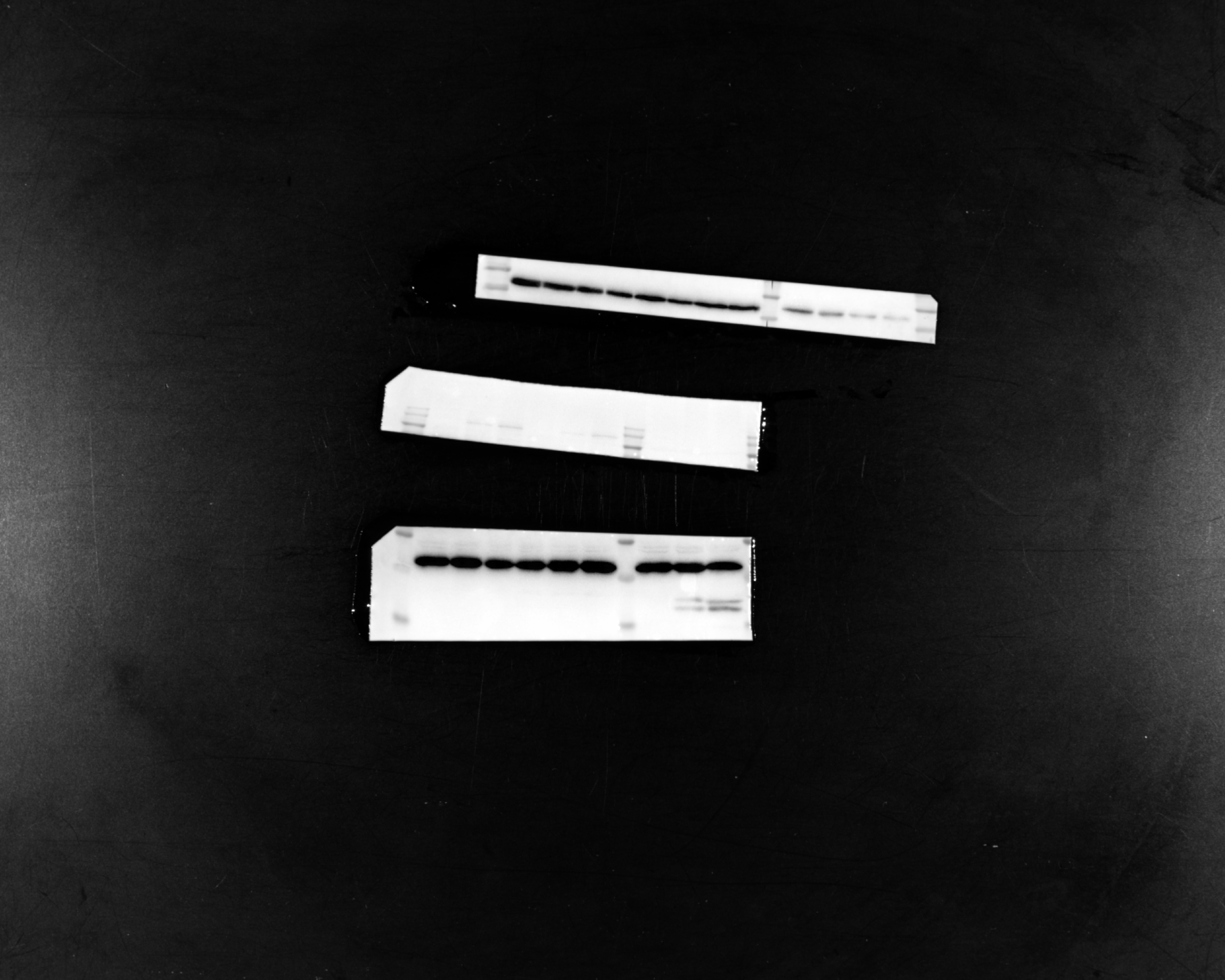

Supplement: Figure 5—source data 2. [file elife-101973-fig5-data2.zip › Figure 5–source data 2/figure 5H/Tubulin.jpg]

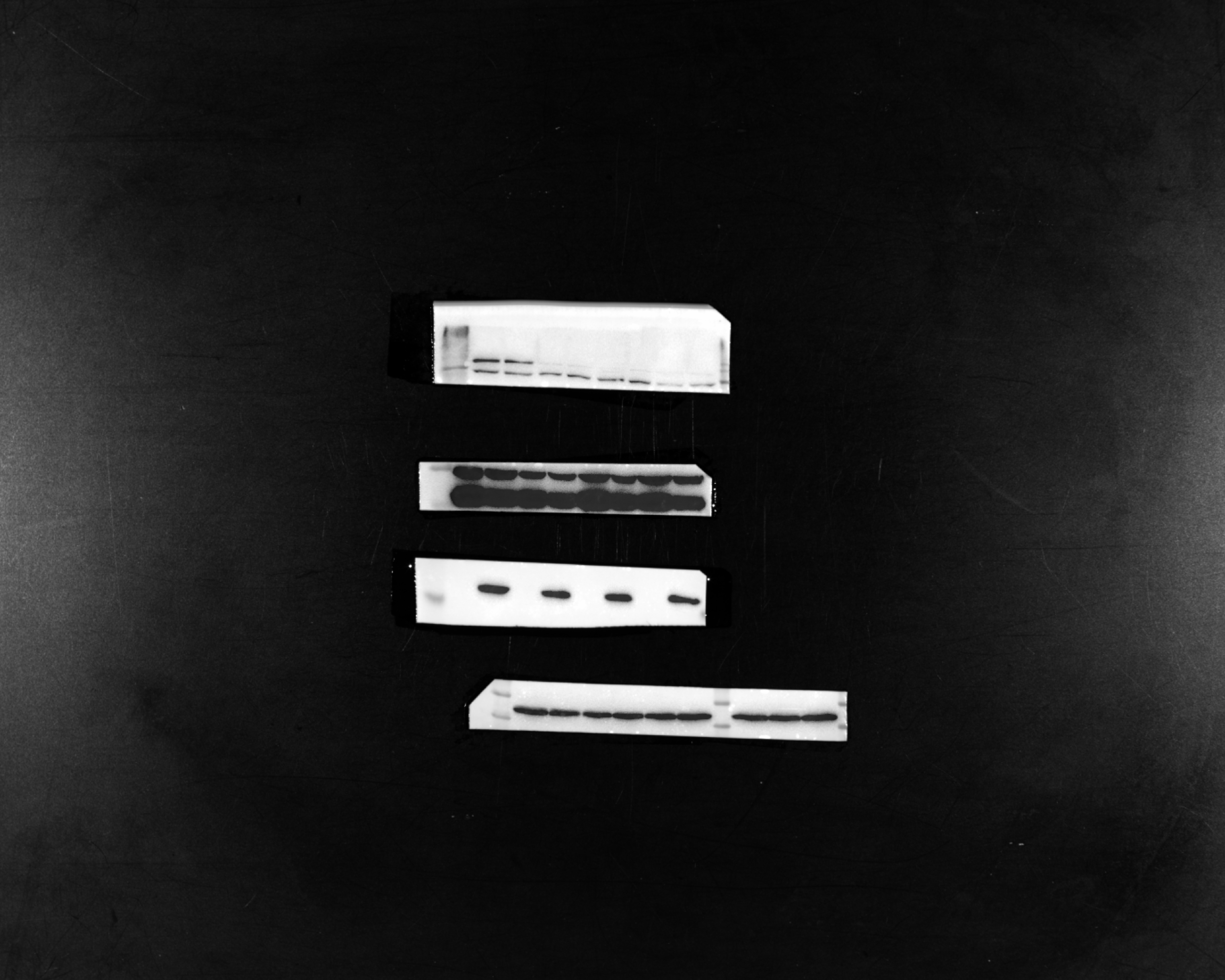

Supplement: Figure 5—source data 2. [file elife-101973-fig5-data2.zip › Figure 5–source data 2/figure 5H/USP10 and flag.jpg]

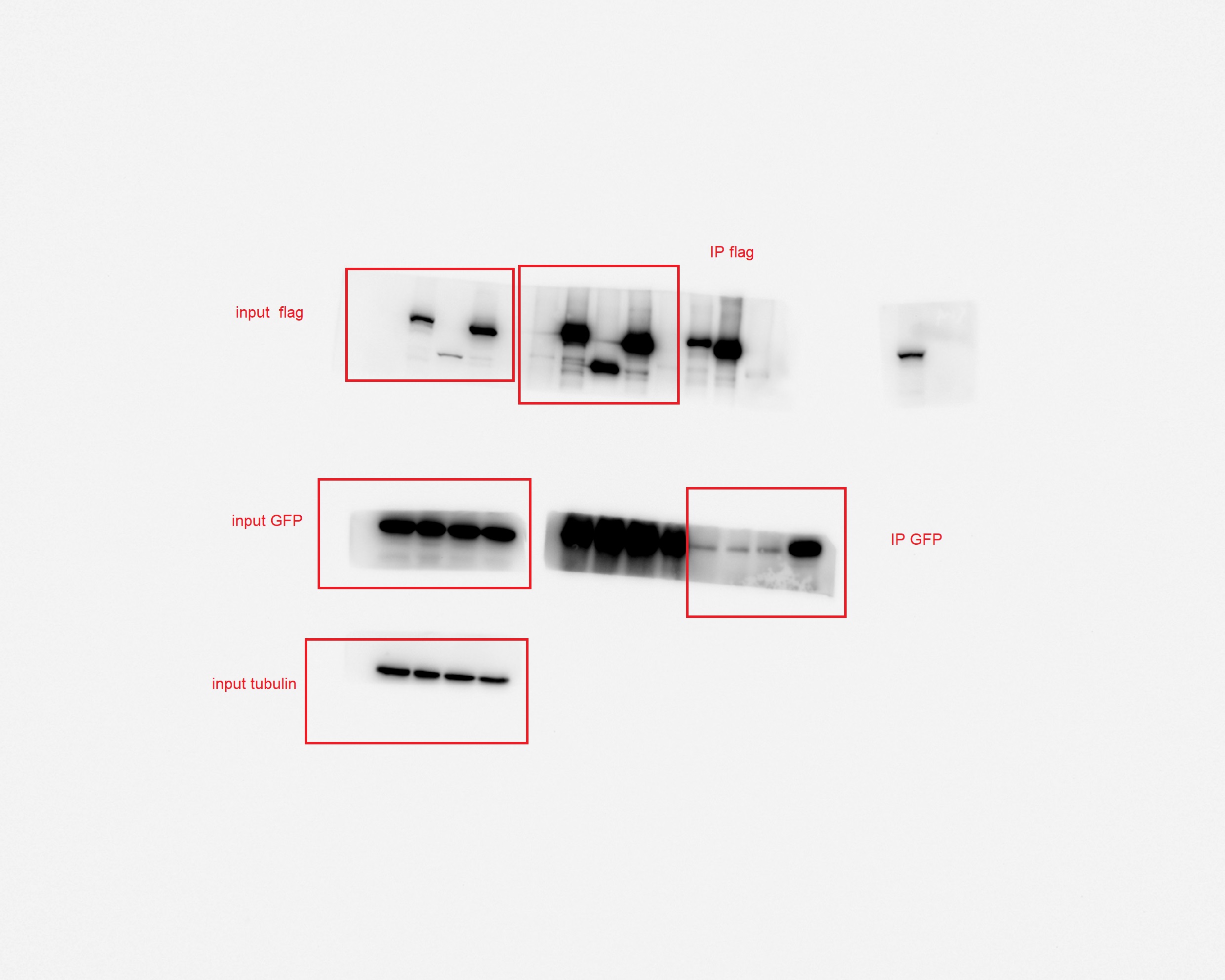

Supplement: Figure 5—figure supplement 1—source data 1. [file elife-101973-fig5-figsupp1-data1.zip › Figure 5-figure supplement1-source data1/input and IP flag GFP.jpg]

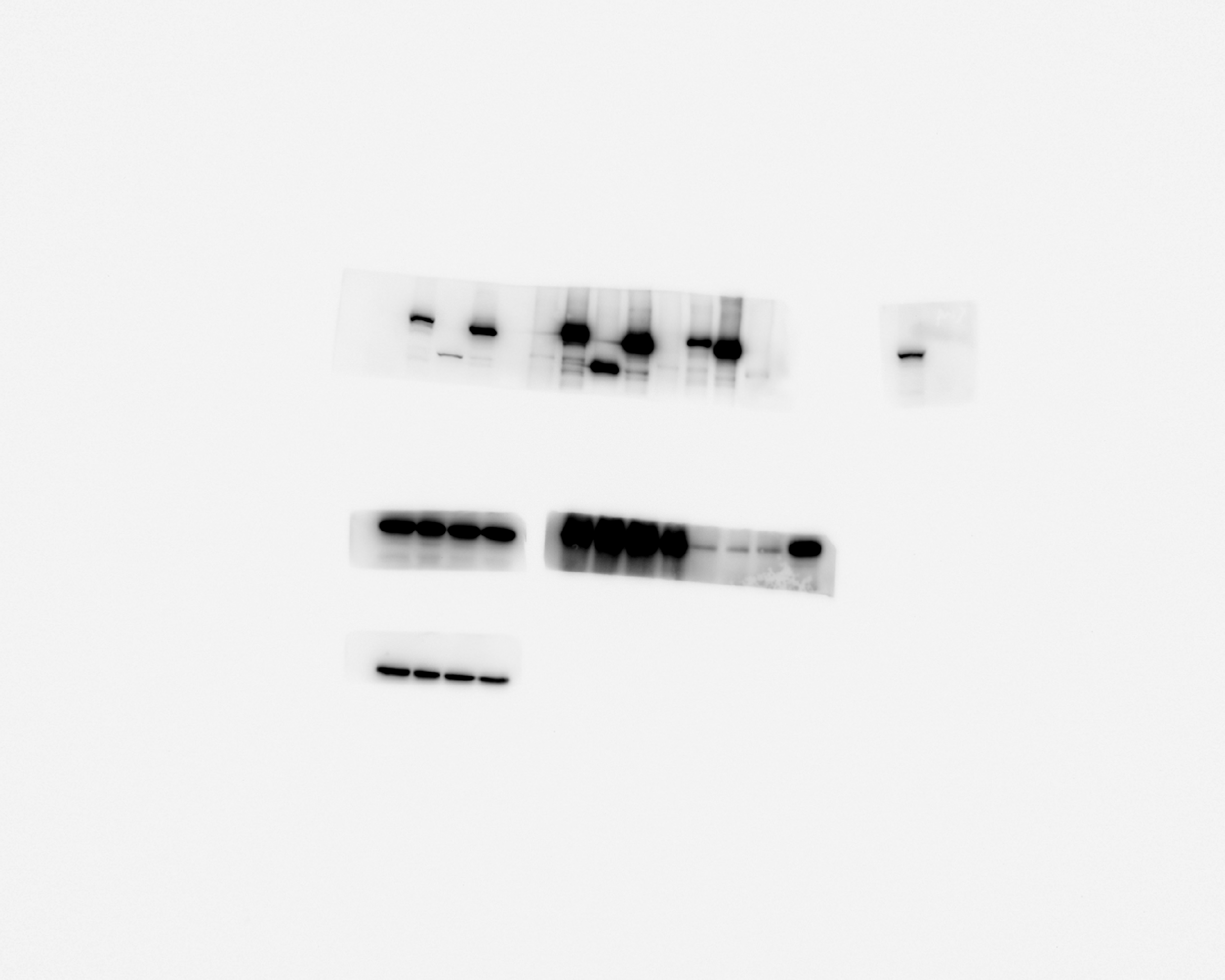

Supplement: Figure 5—figure supplement 1—source data 2. [file elife-101973-fig5-figsupp1-data2.zip › Figure 5-figure supplement 1-source data 2/input and IP flag GFP.tif]

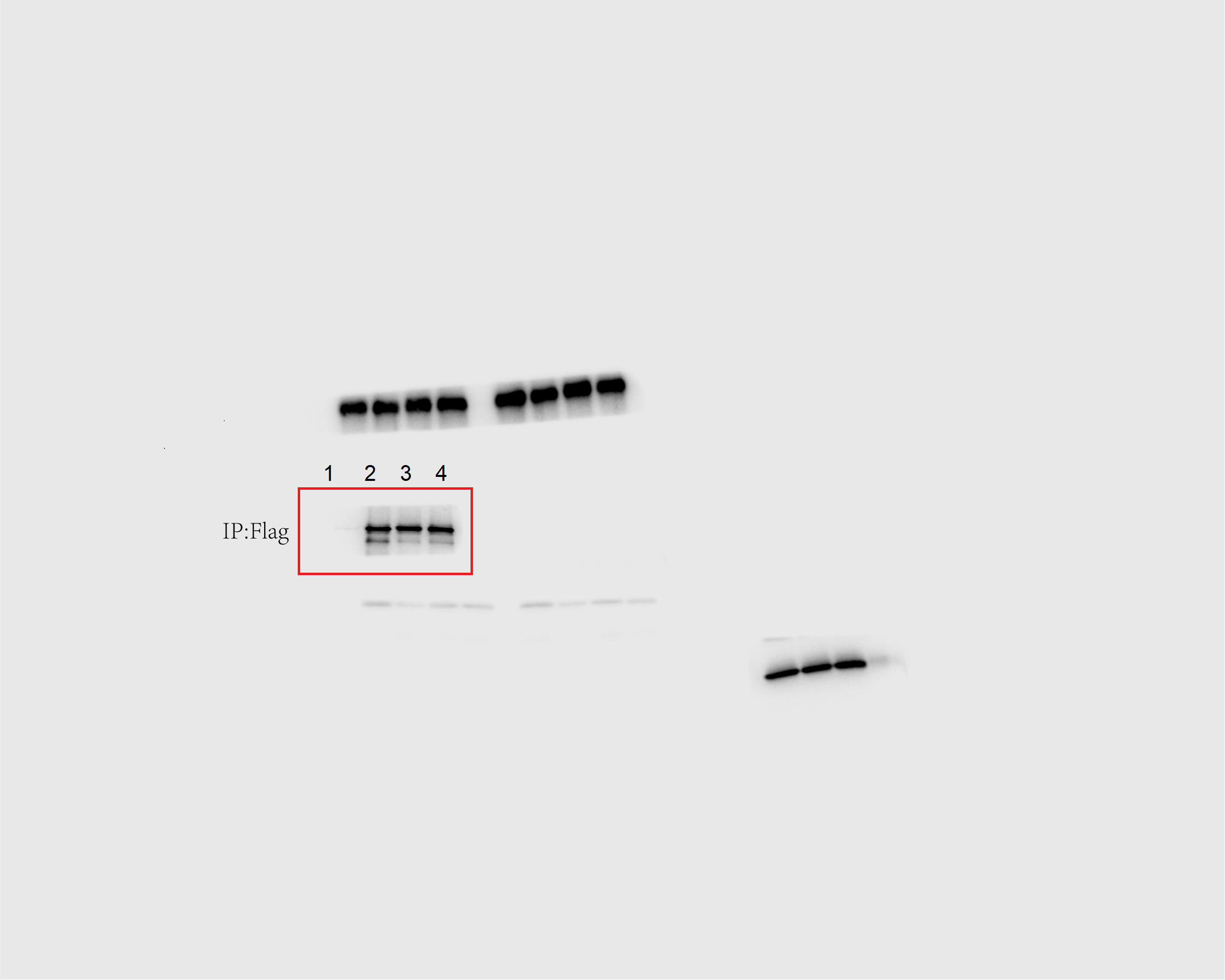

Supplement: Figure 6—source data 1. [file elife-101973-fig6-data1.zip › Figure 6-source data 1/Fig6A-labeled/IP Flag.tif]

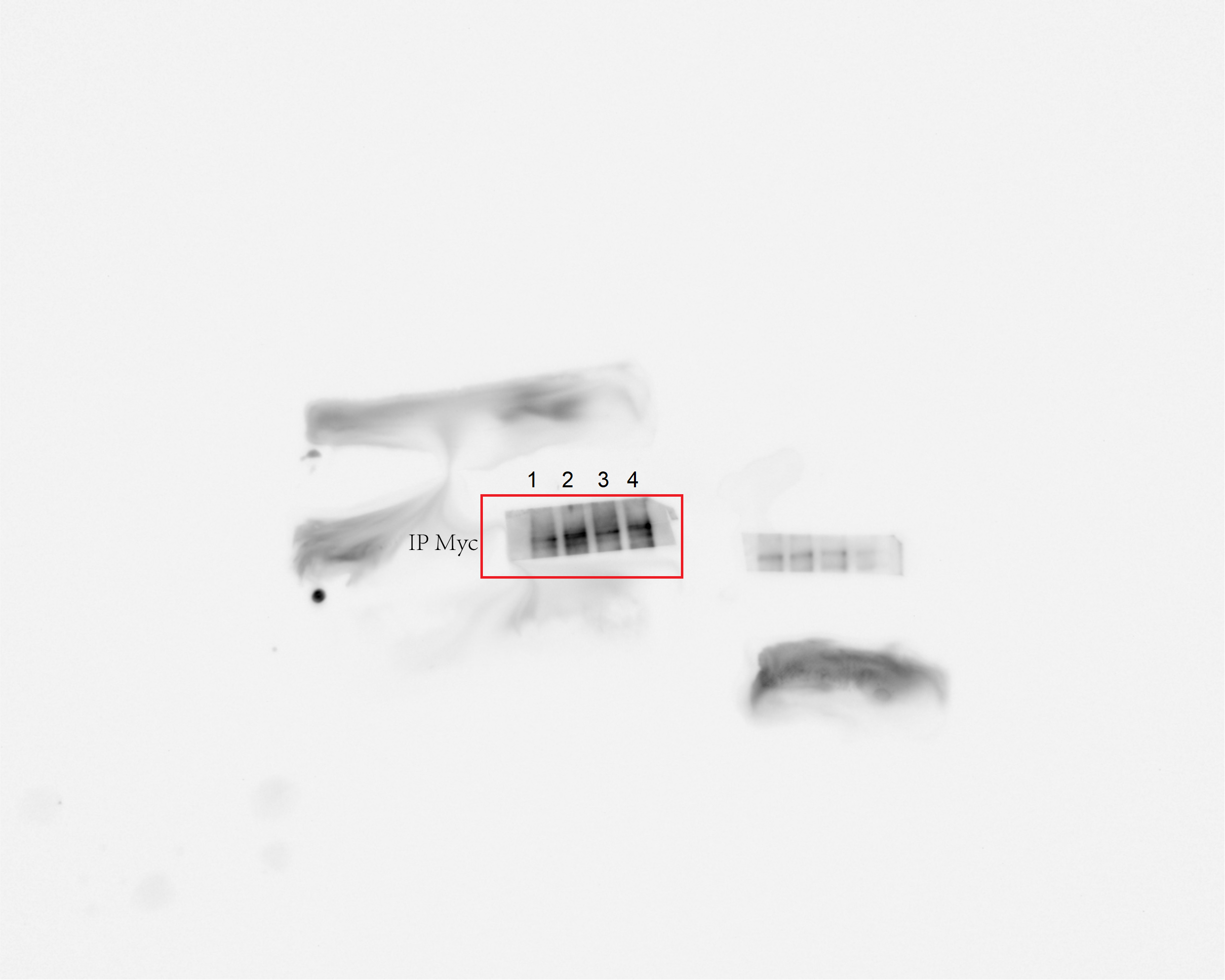

Supplement: Figure 6—source data 1. [file elife-101973-fig6-data1.zip › Figure 6-source data 1/Fig6A-labeled/IP Myc.tif]

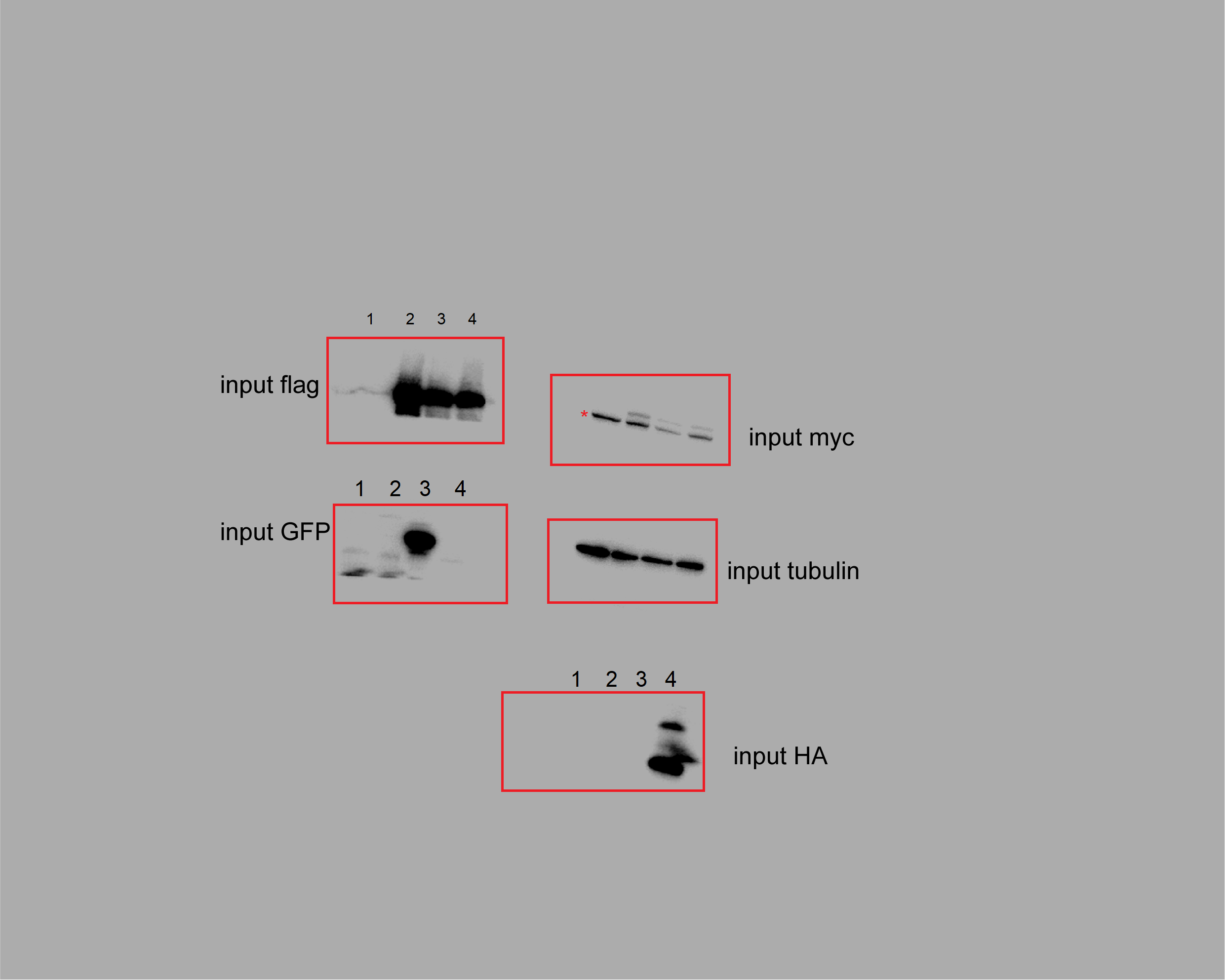

Supplement: Figure 6—source data 1. [file elife-101973-fig6-data1.zip › Figure 6-source data 1/Fig6A-labeled/Input Flag Myc GFP HA and Tubulin.tif]

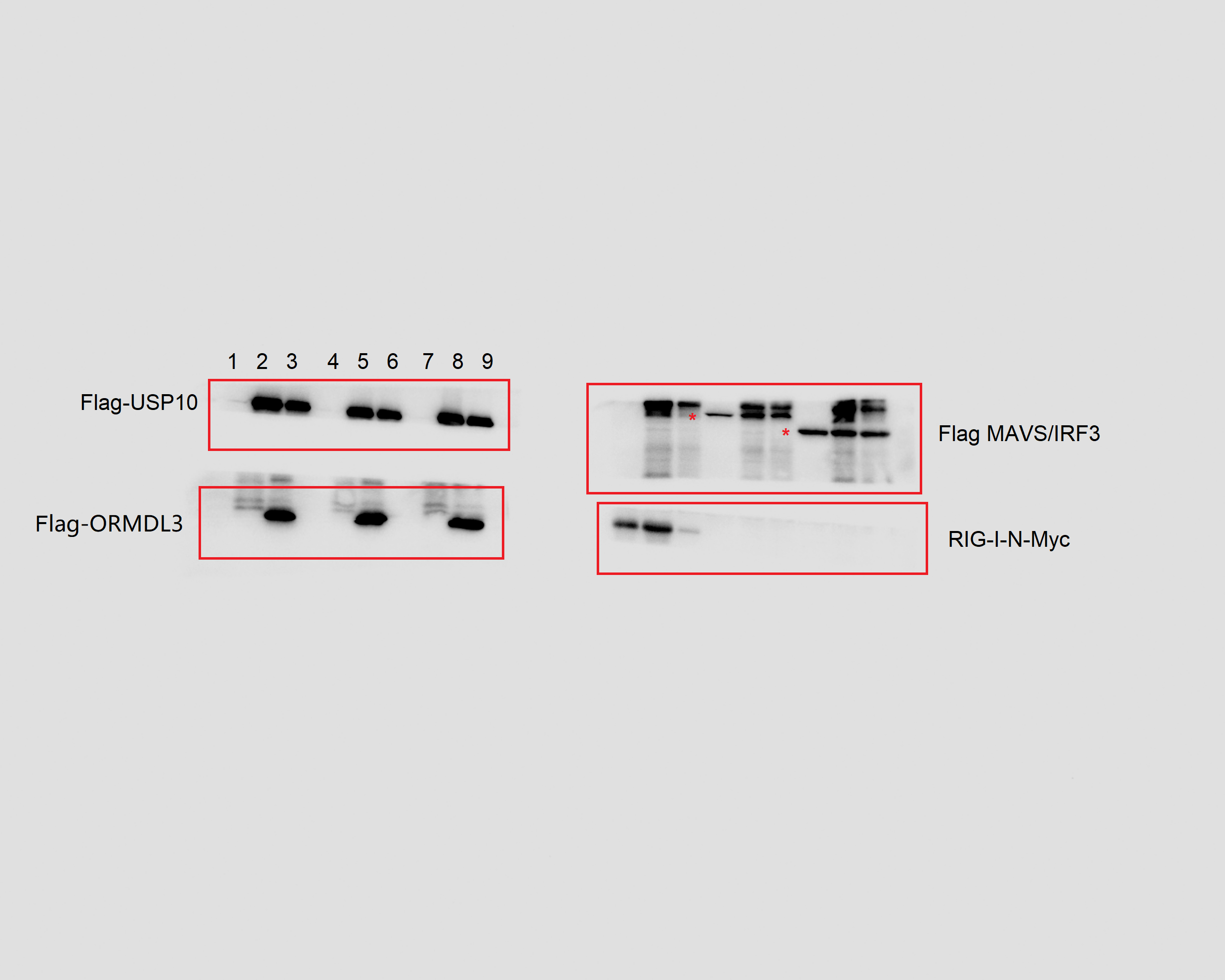

Supplement: Figure 6—source data 1. [file elife-101973-fig6-data1.zip › Figure 6-source data 1/Fig6B-labeled/Flag and Myc.tif]

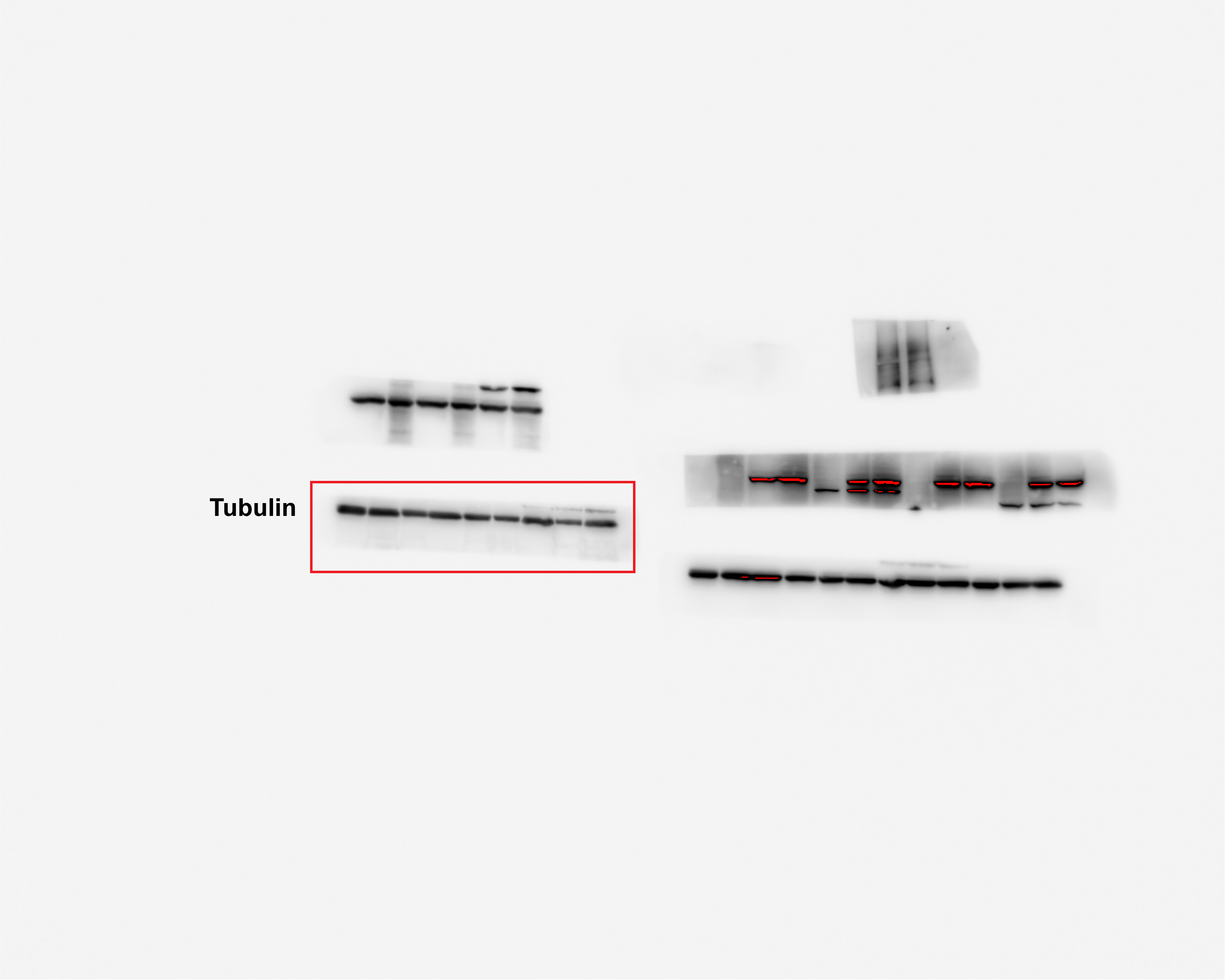

Supplement: Figure 6—source data 1. [file elife-101973-fig6-data1.zip › Figure 6-source data 1/Fig6B-labeled/Tubulin.tif]

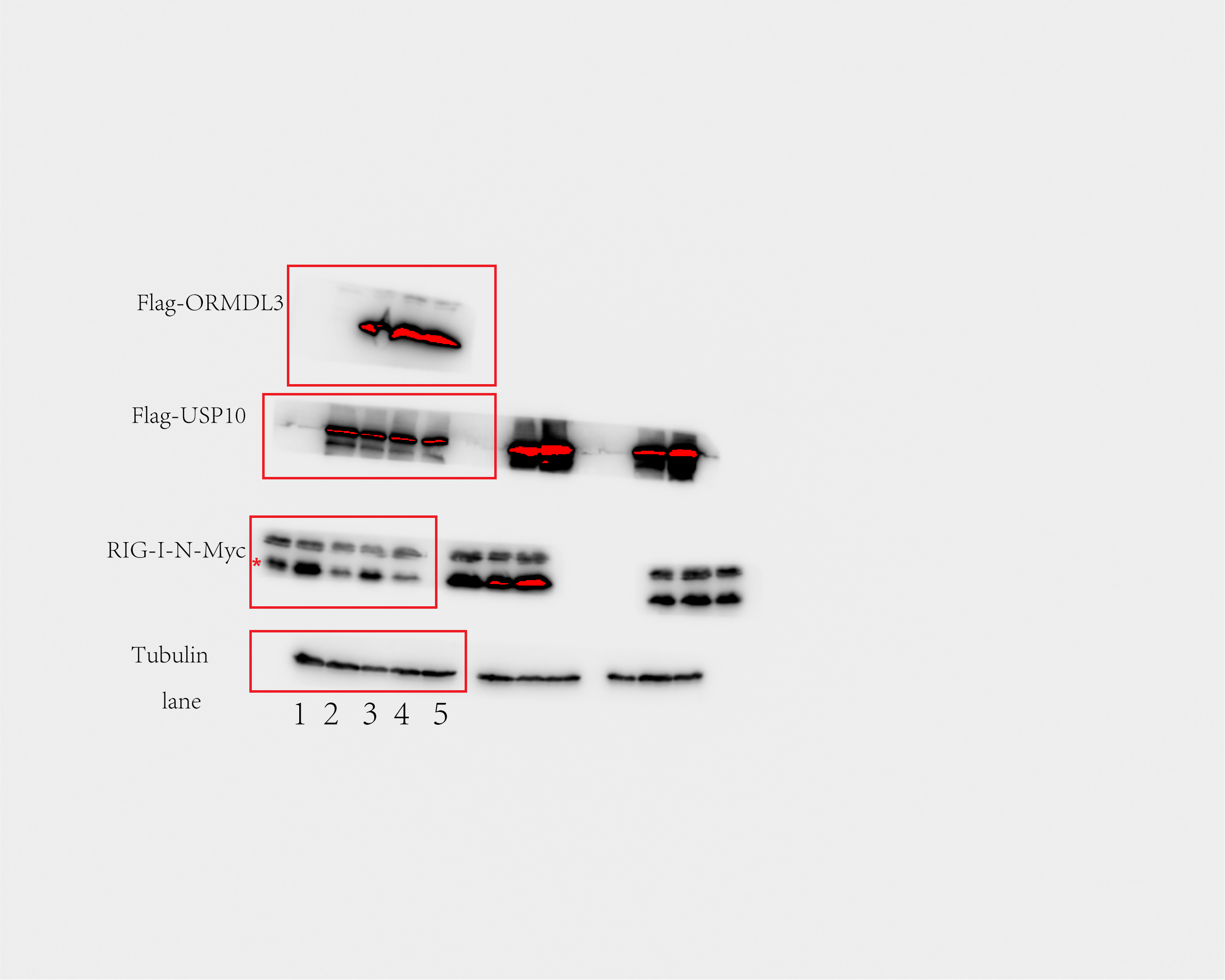

Supplement: Figure 6—source data 1. [file elife-101973-fig6-data1.zip › Figure 6-source data 1/Fig6C-labeled/long exposure of myc,flag and tubulin.tif]

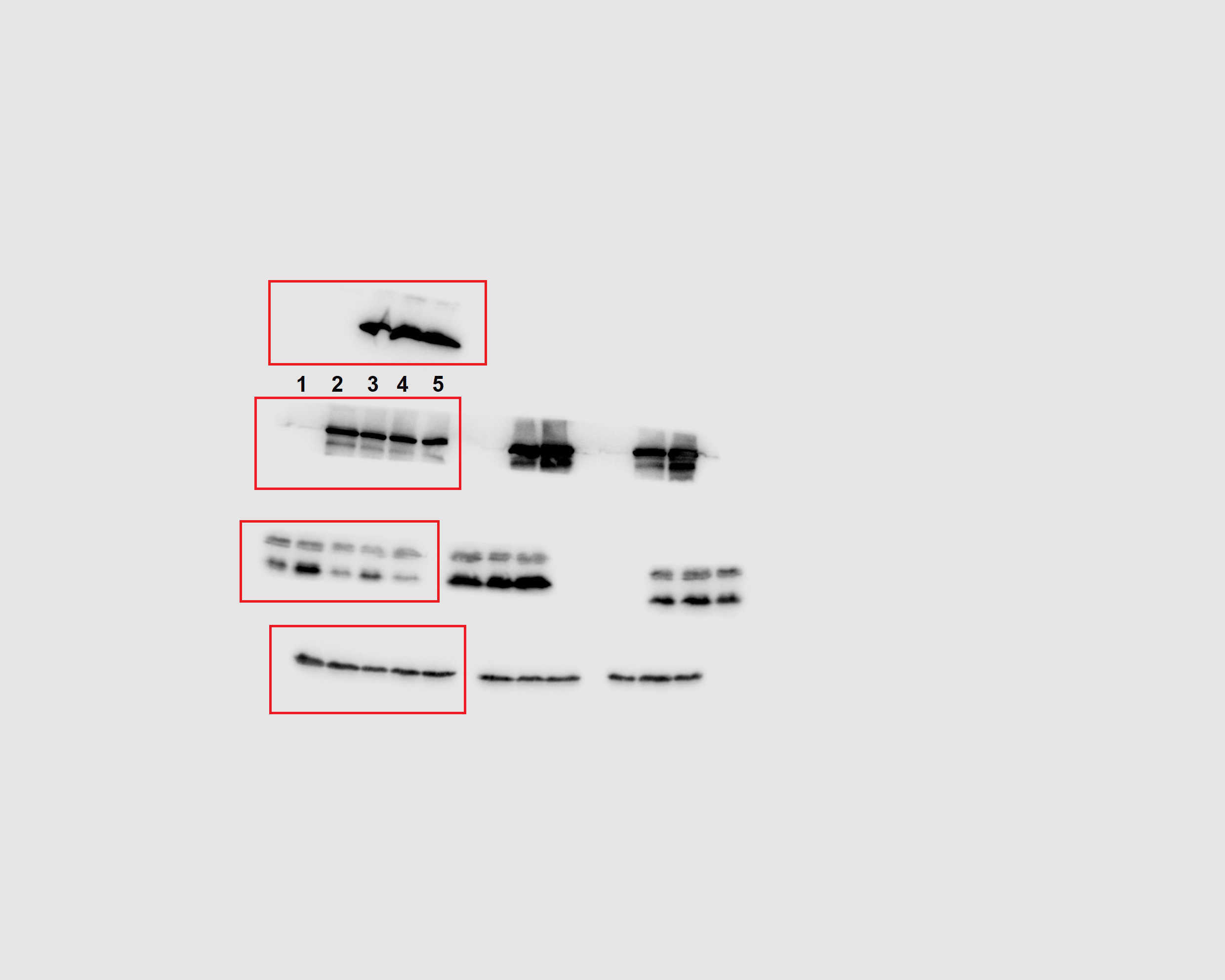

Supplement: Figure 6—source data 1. [file elife-101973-fig6-data1.zip › Figure 6-source data 1/Fig6C-labeled/short exposure.tif]

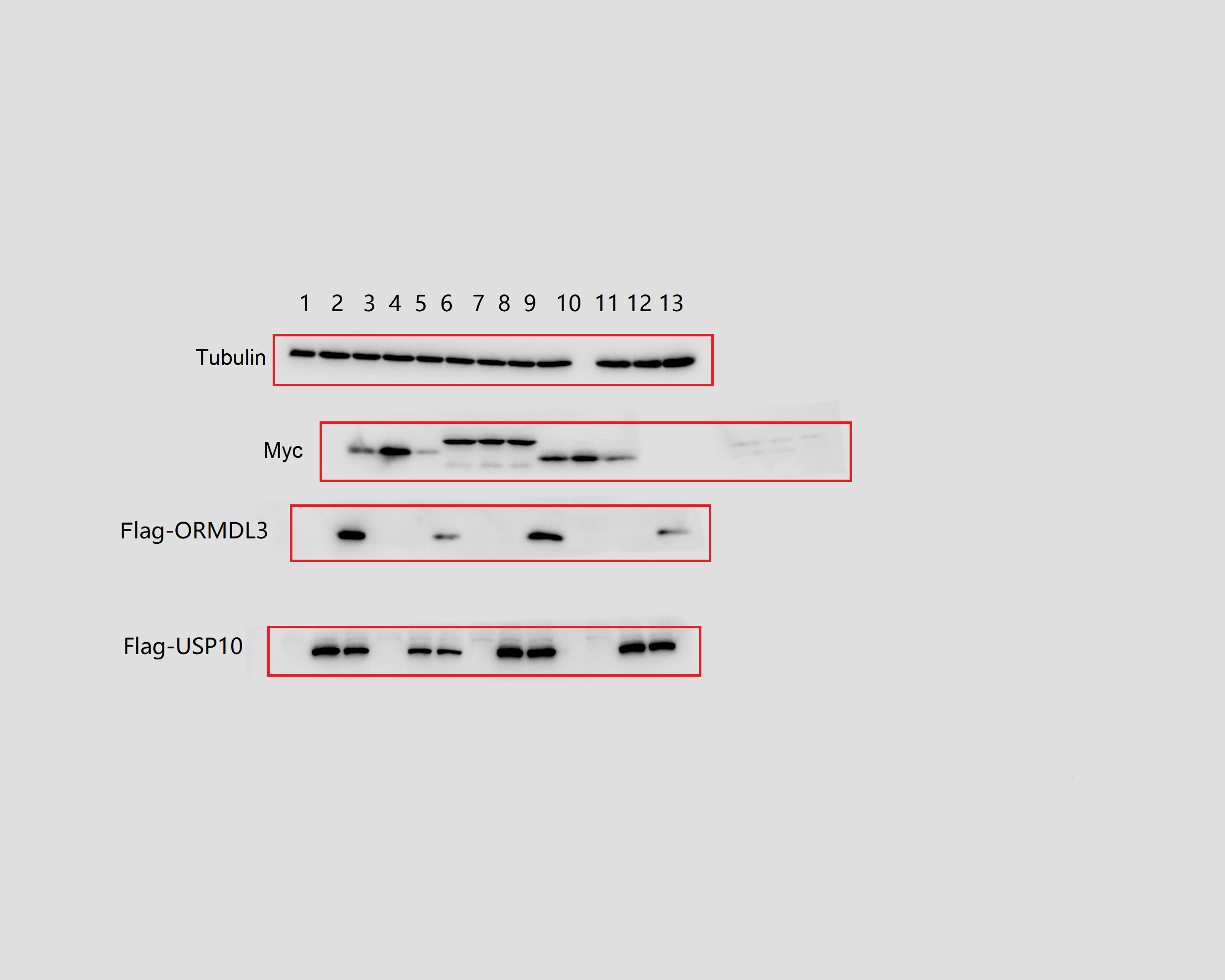

Supplement: Figure 6—source data 1. [file elife-101973-fig6-data1.zip › Figure 6-source data 1/Fig6D-labeled/Myc Flag and Tubulin.tif]

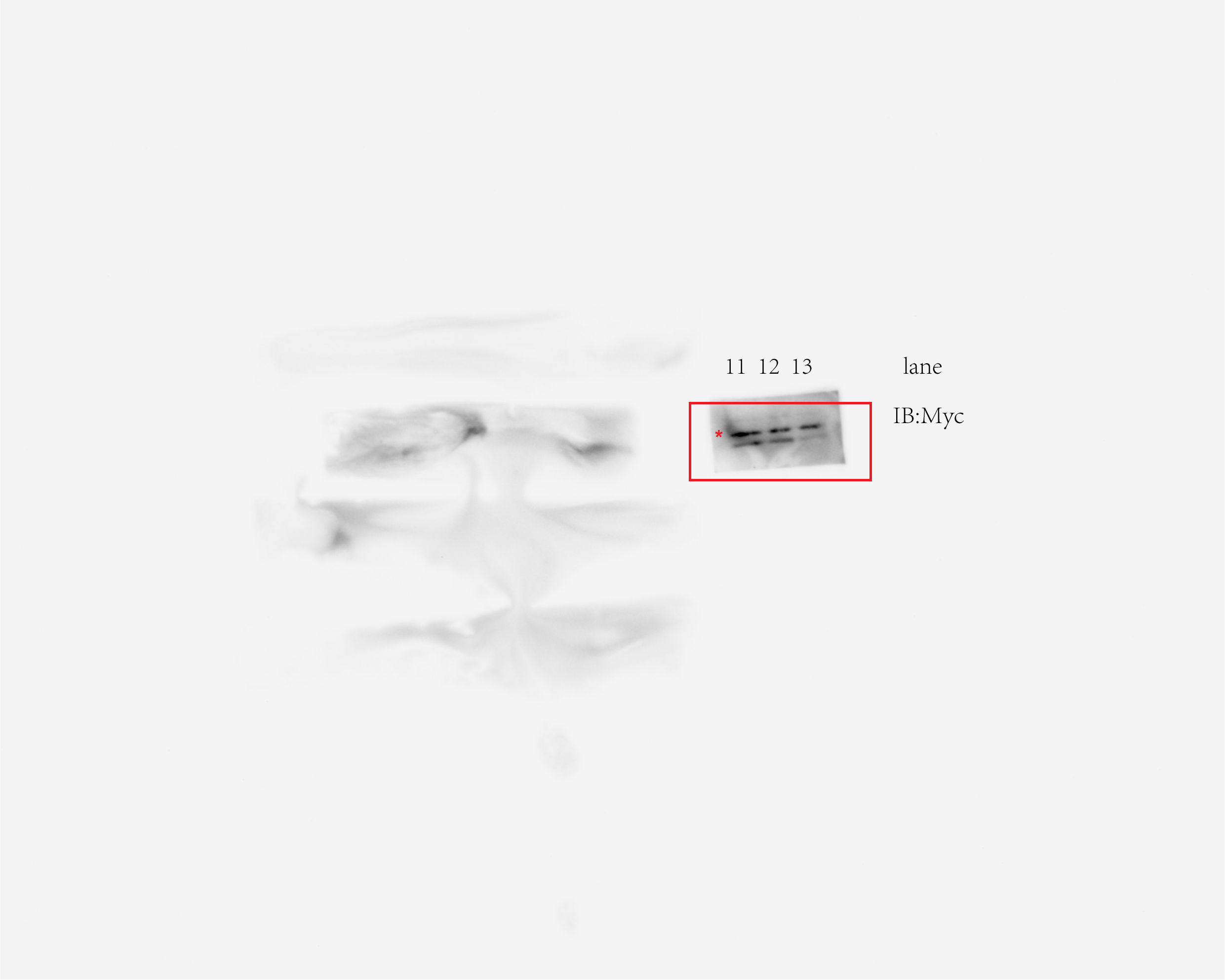

Supplement: Figure 6—source data 1. [file elife-101973-fig6-data1.zip › Figure 6-source data 1/Fig6D-labeled/long exposure of Lane 11 12 13 RIG-I-N-Myc-mutant1.tif]

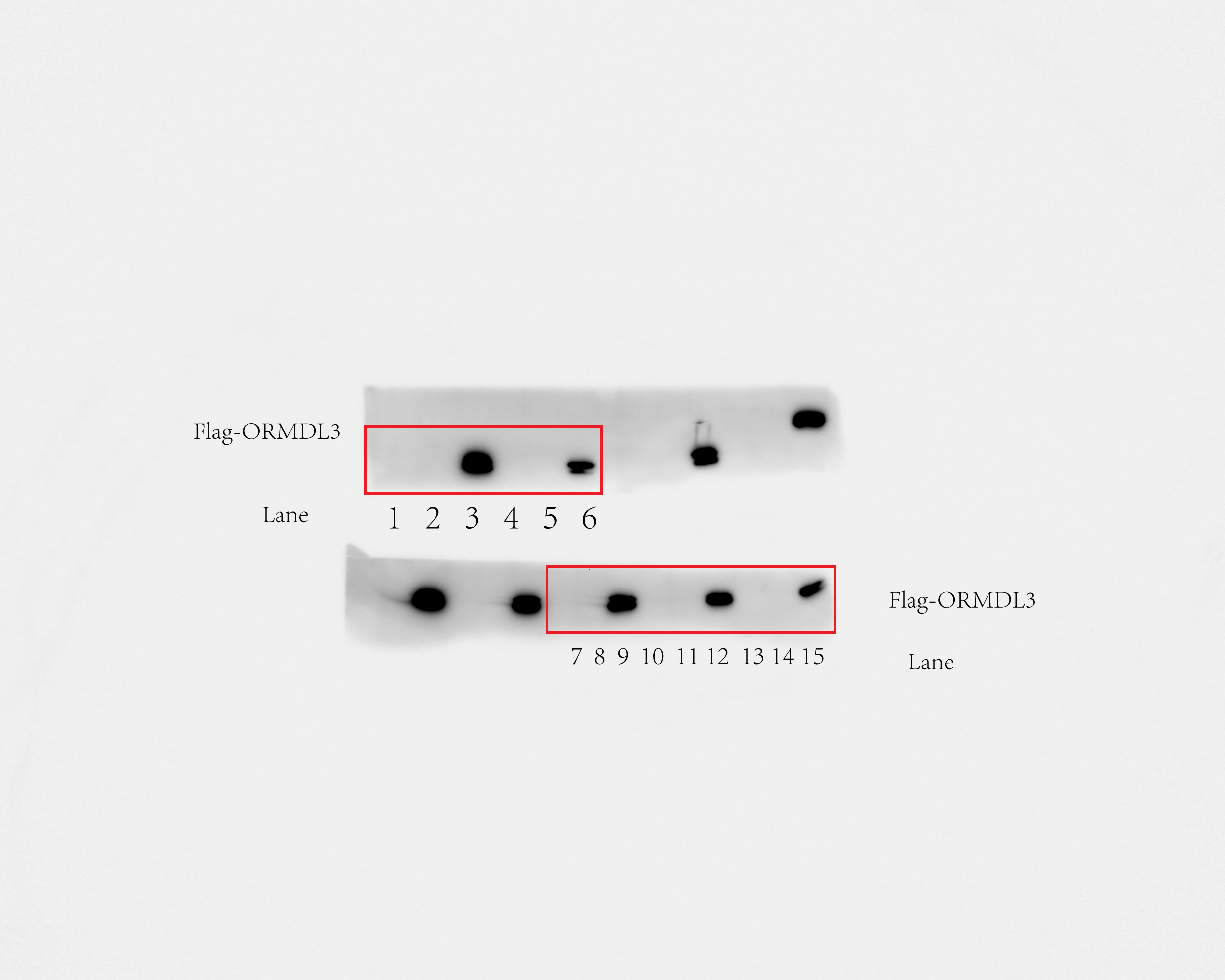

Supplement: Figure 6—source data 1. [file elife-101973-fig6-data1.zip › Figure 6-source data 1/Fig6E-labeled/Flag ORMDL3.tif]

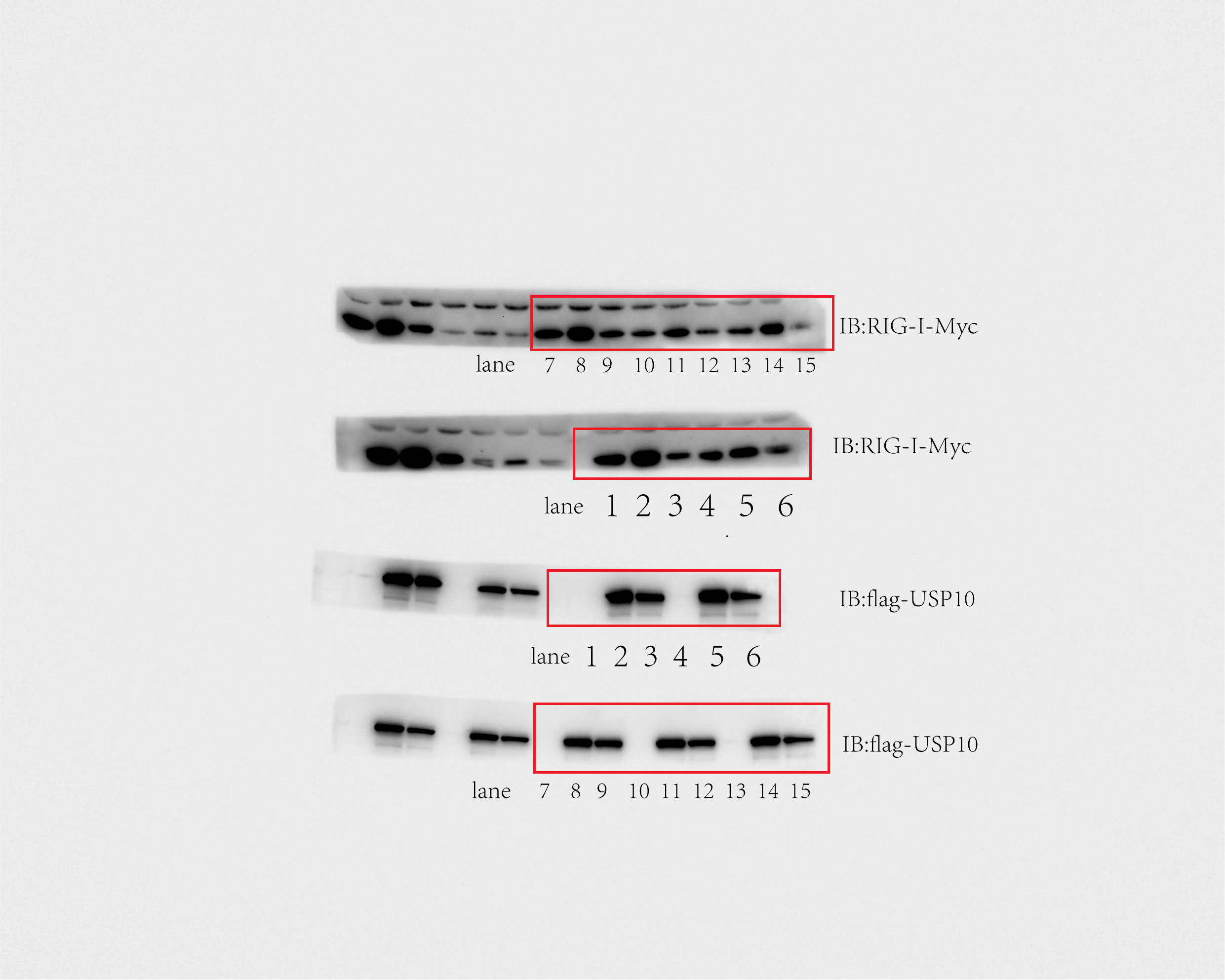

Supplement: Figure 6—source data 1. [file elife-101973-fig6-data1.zip › Figure 6-source data 1/Fig6E-labeled/Myc and Flag.tif]

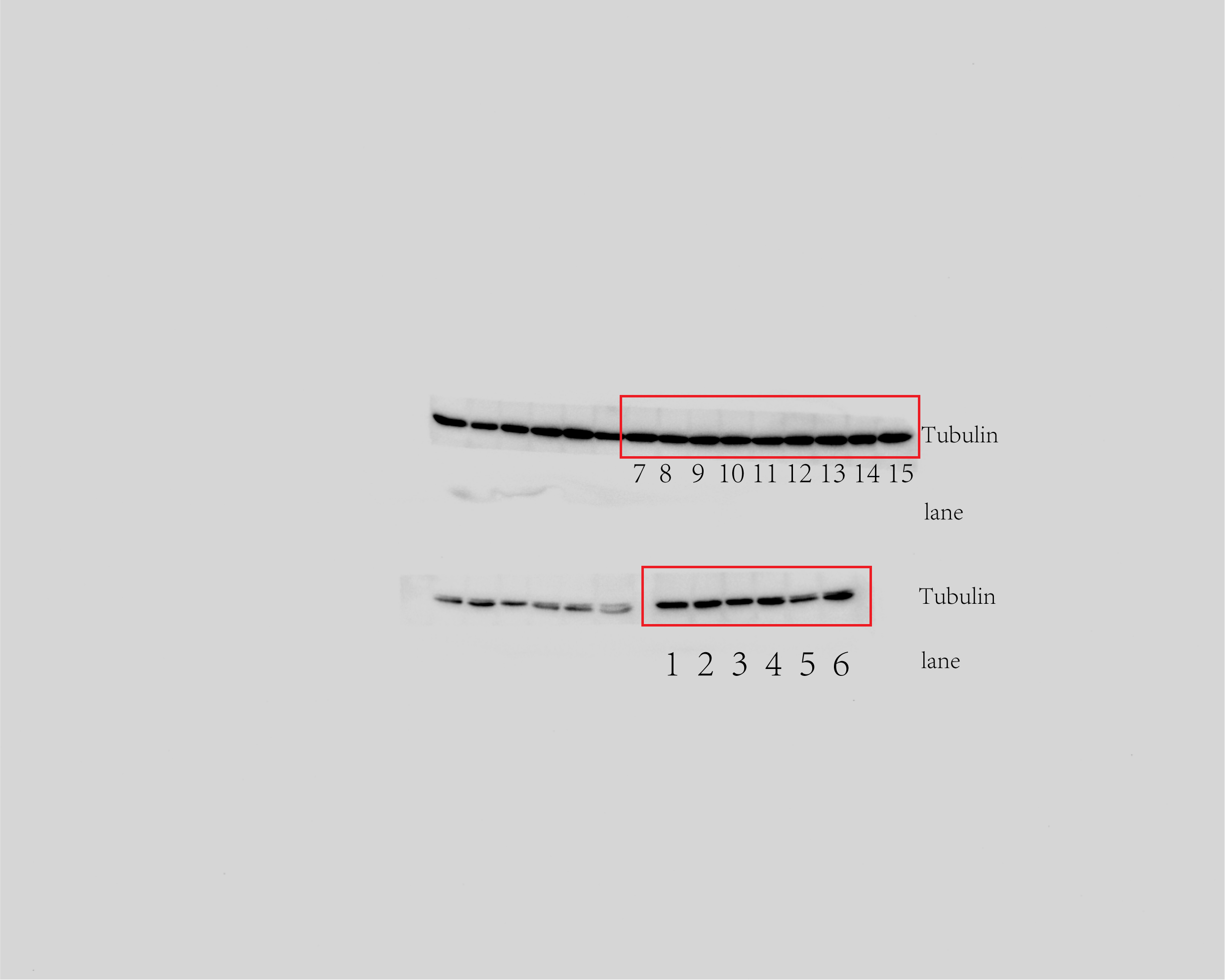

Supplement: Figure 6—source data 1. [file elife-101973-fig6-data1.zip › Figure 6-source data 1/Fig6E-labeled/Tubulin.tif]

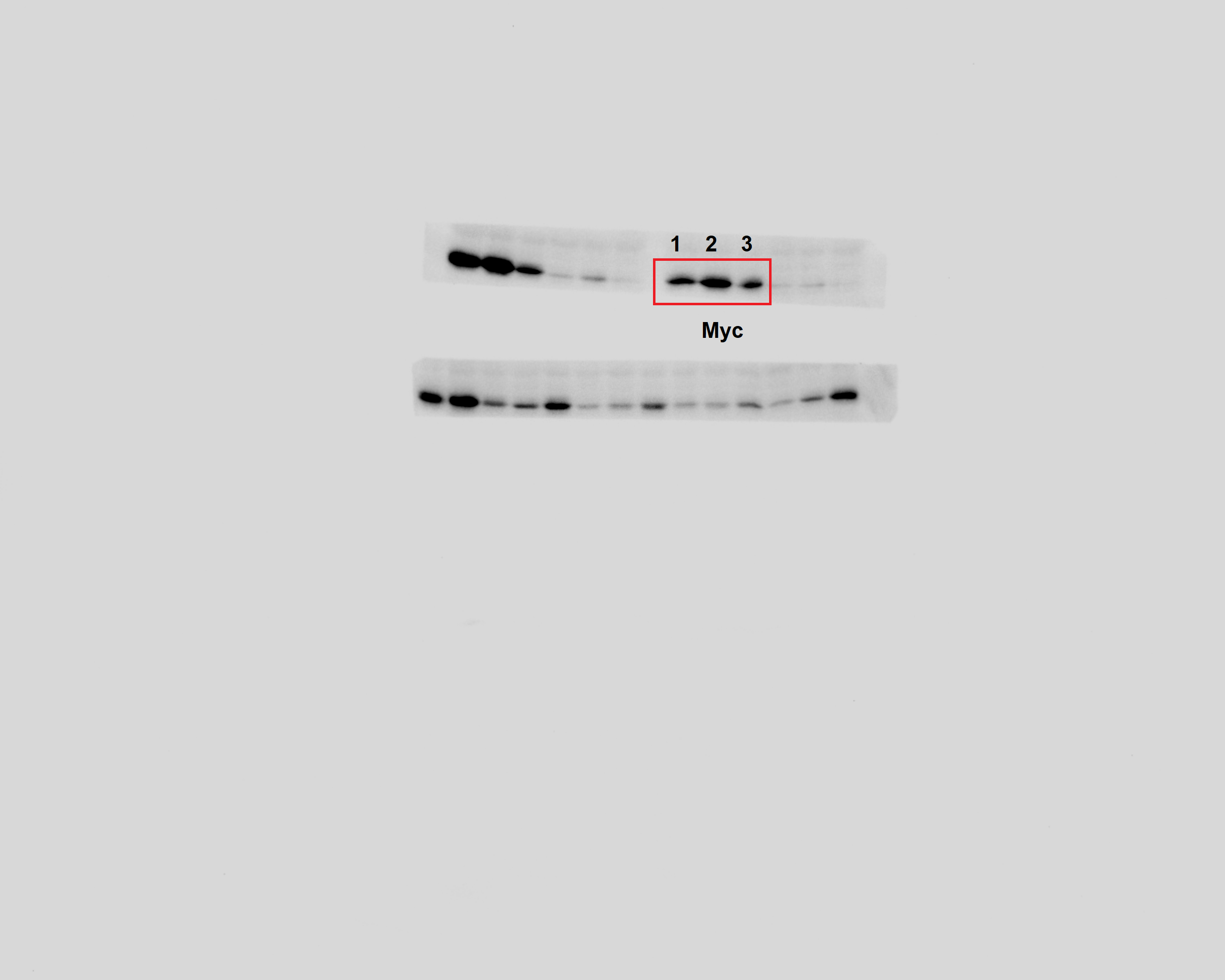

Supplement: Figure 6—source data 1. [file elife-101973-fig6-data1.zip › Figure 6-source data 1/Fig6F-labeled/lane 1 2 3 Myc.tif]

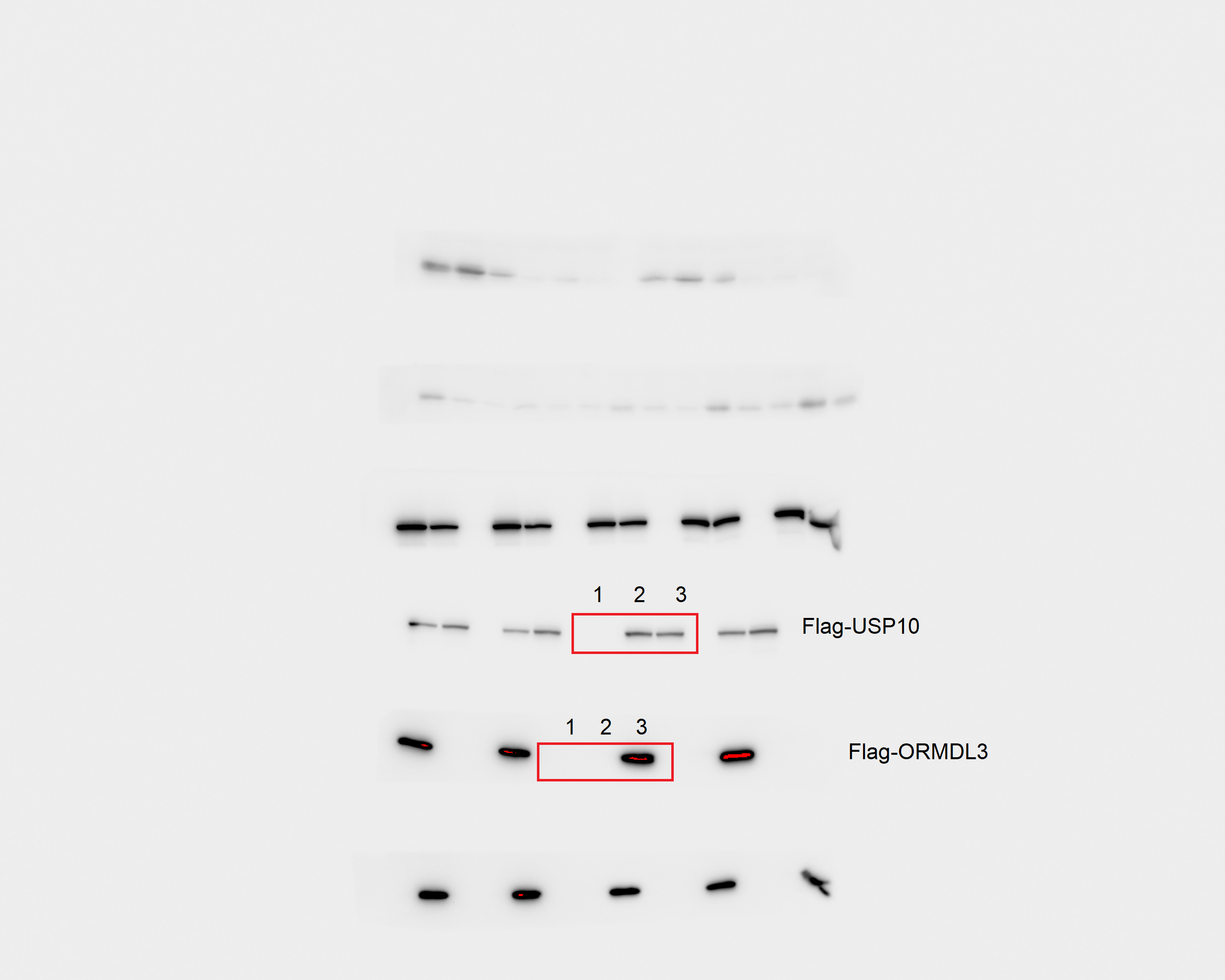

Supplement: Figure 6—source data 1. [file elife-101973-fig6-data1.zip › Figure 6-source data 1/Fig6F-labeled/lane 1 2 3 flag .tif]

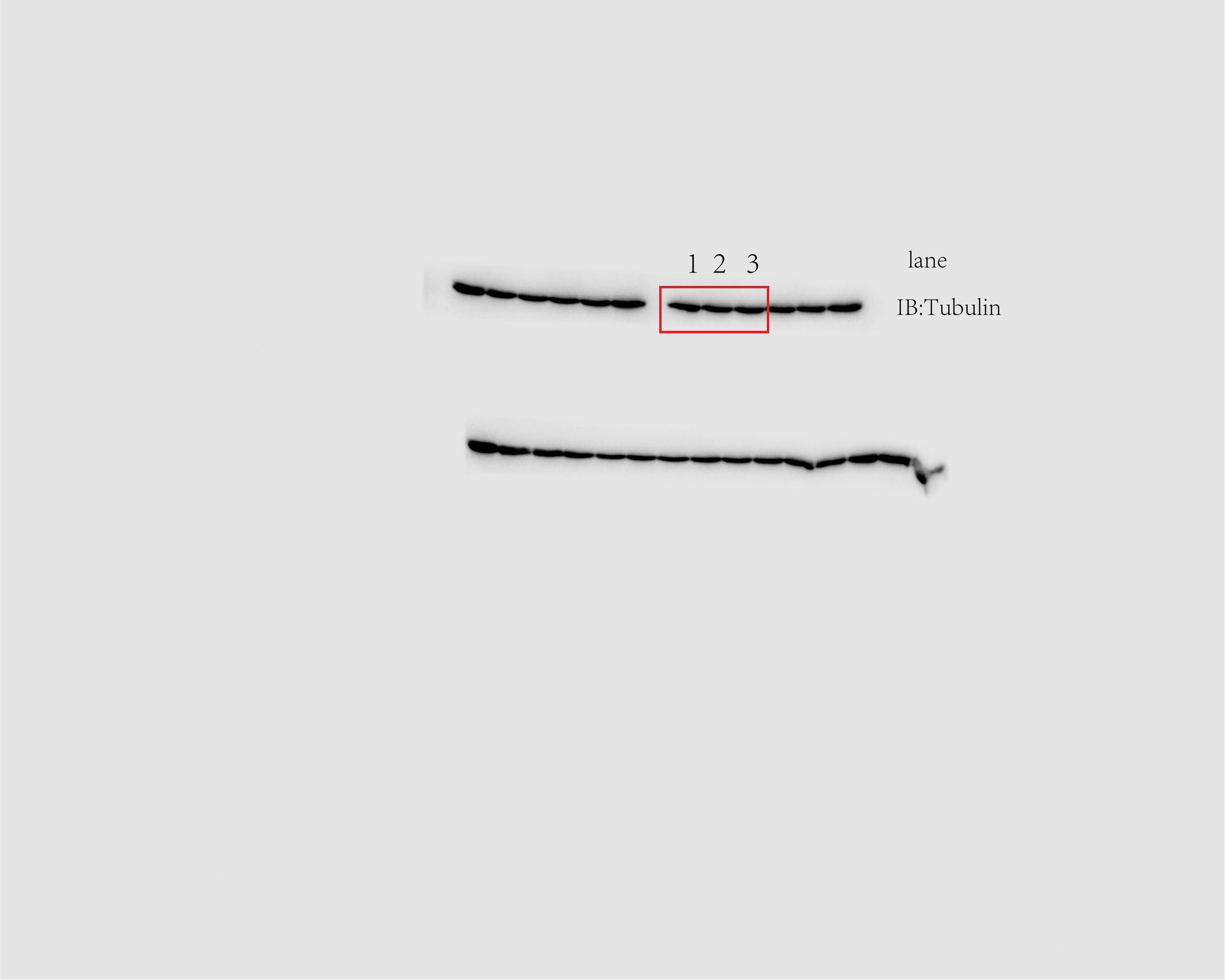

Supplement: Figure 6—source data 1. [file elife-101973-fig6-data1.zip › Figure 6-source data 1/Fig6F-labeled/lane 1 2 3 Tubulin.tif]

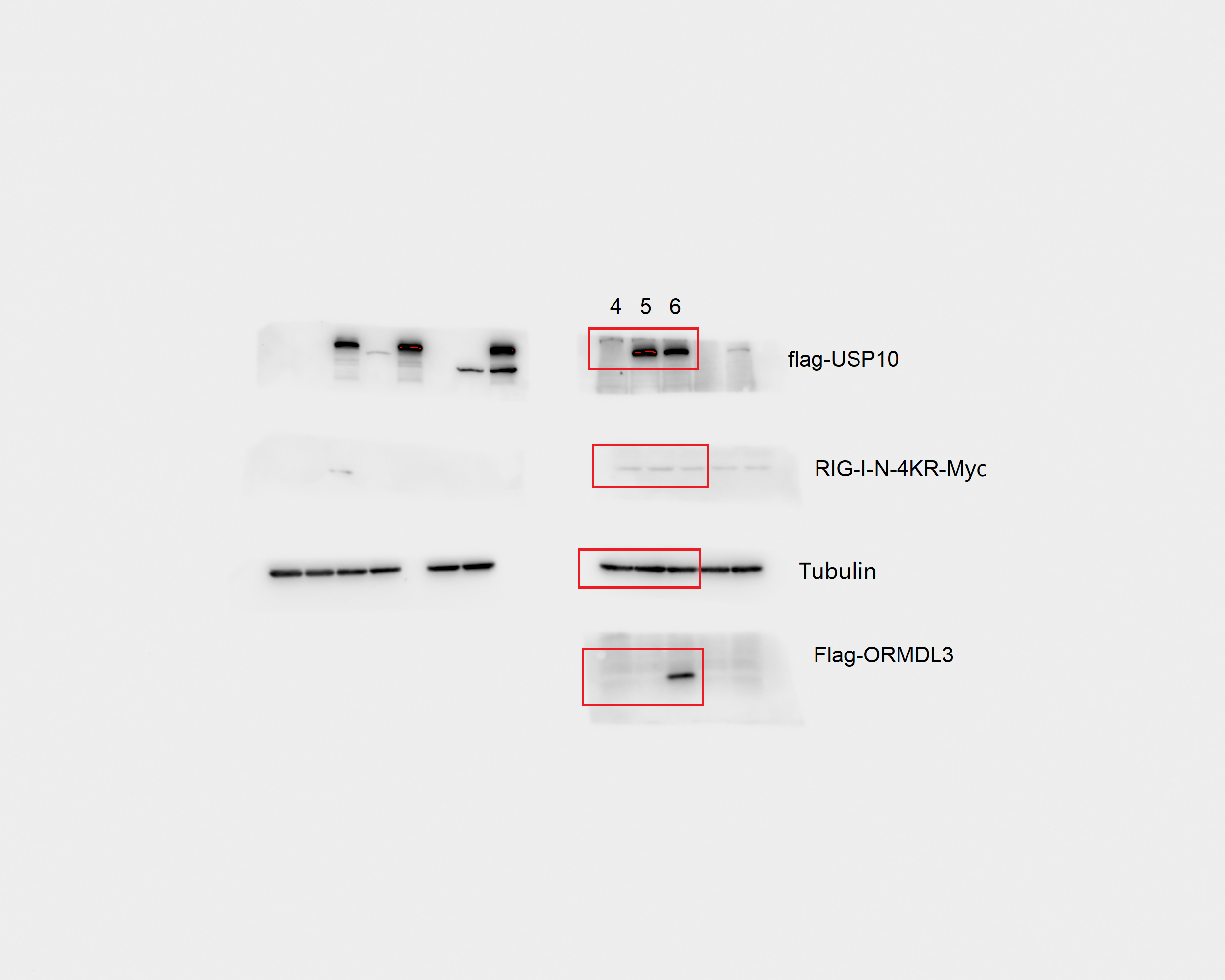

Supplement: Figure 6—source data 1. [file elife-101973-fig6-data1.zip › Figure 6-source data 1/Fig6F-labeled/lane 4 5 6 long exposure of Flag Mycand Tubulin.tif]

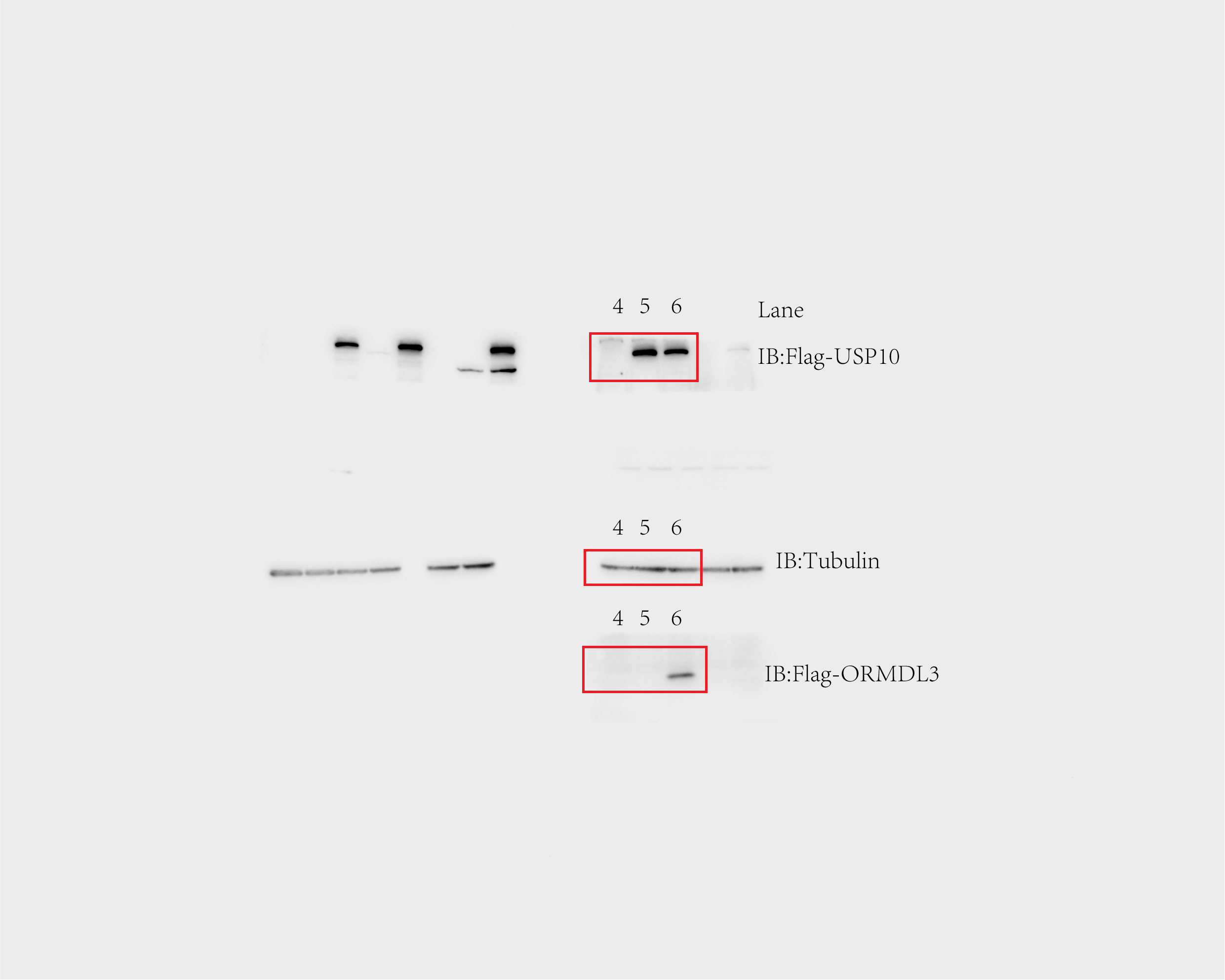

Supplement: Figure 6—source data 1. [file elife-101973-fig6-data1.zip › Figure 6-source data 1/Fig6F-labeled/lane 4 5 6 short exposure of Flag and Tubulin.tif]

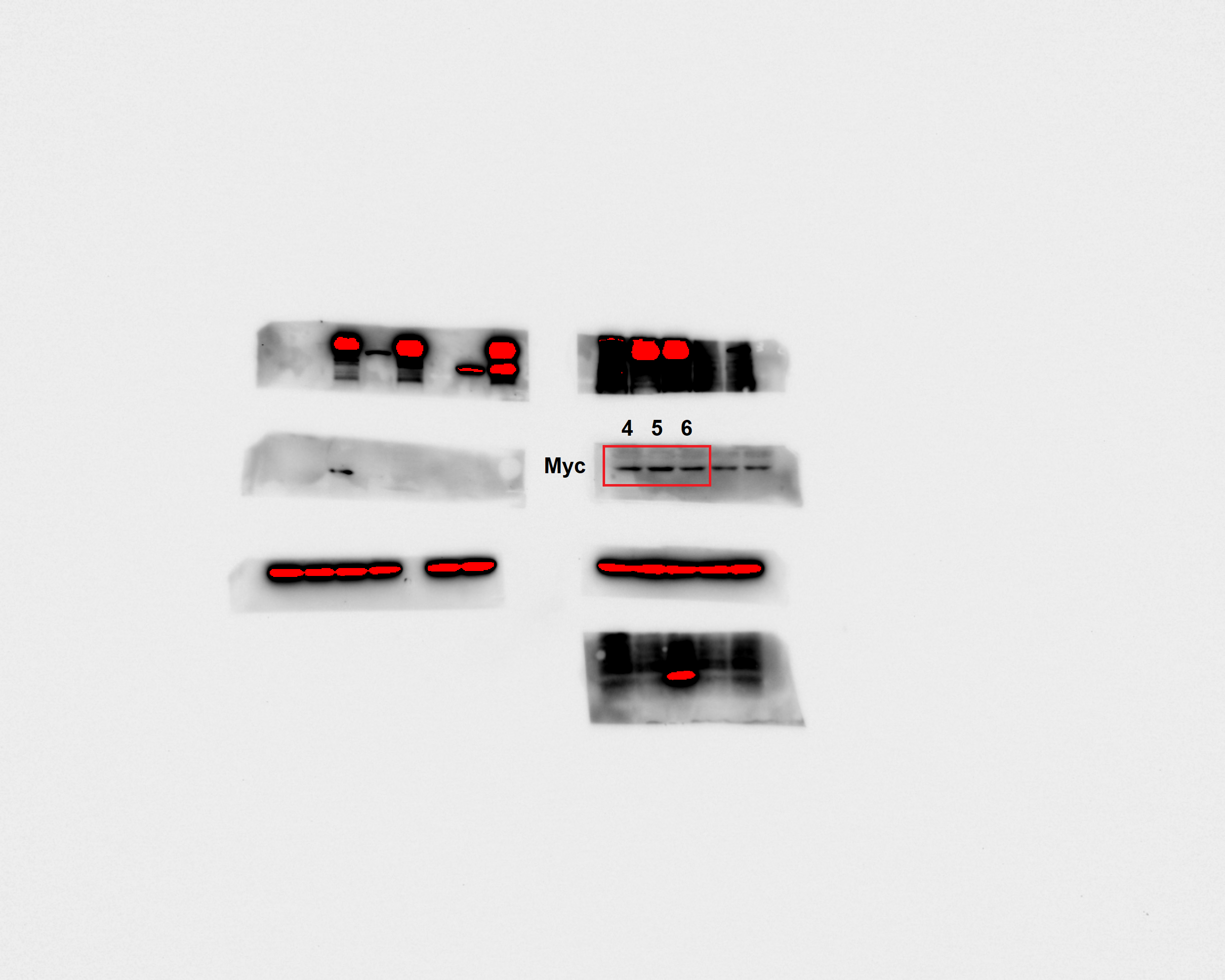

Supplement: Figure 6—source data 1. [file elife-101973-fig6-data1.zip › Figure 6-source data 1/Fig6F-labeled/long exposure oF lane 4 5 6 RIG-I-N-4KR-Myc.tif]

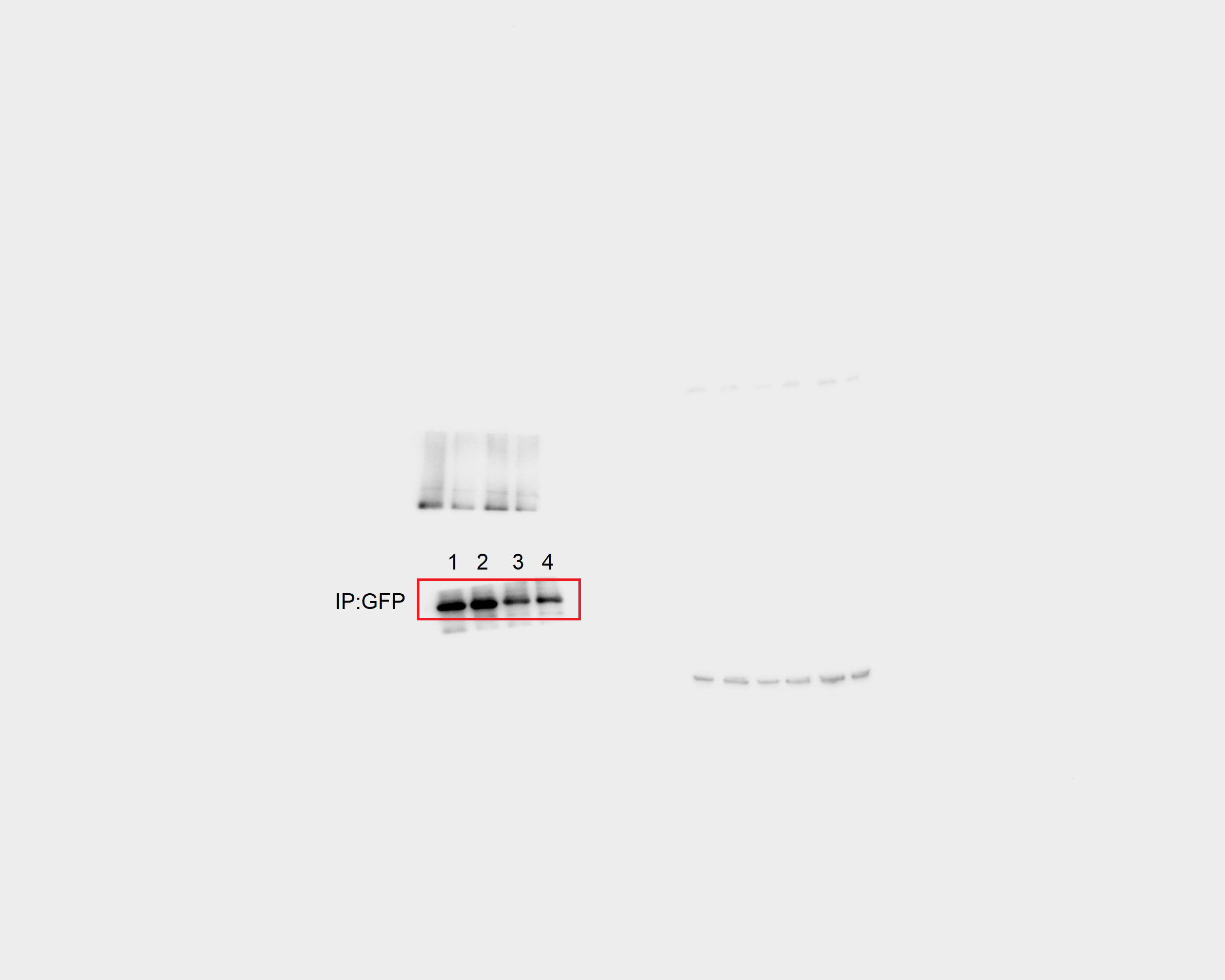

Supplement: Figure 6—source data 1. [file elife-101973-fig6-data1.zip › Figure 6-source data 1/Fig6G-labeled/IP GFP.tif]

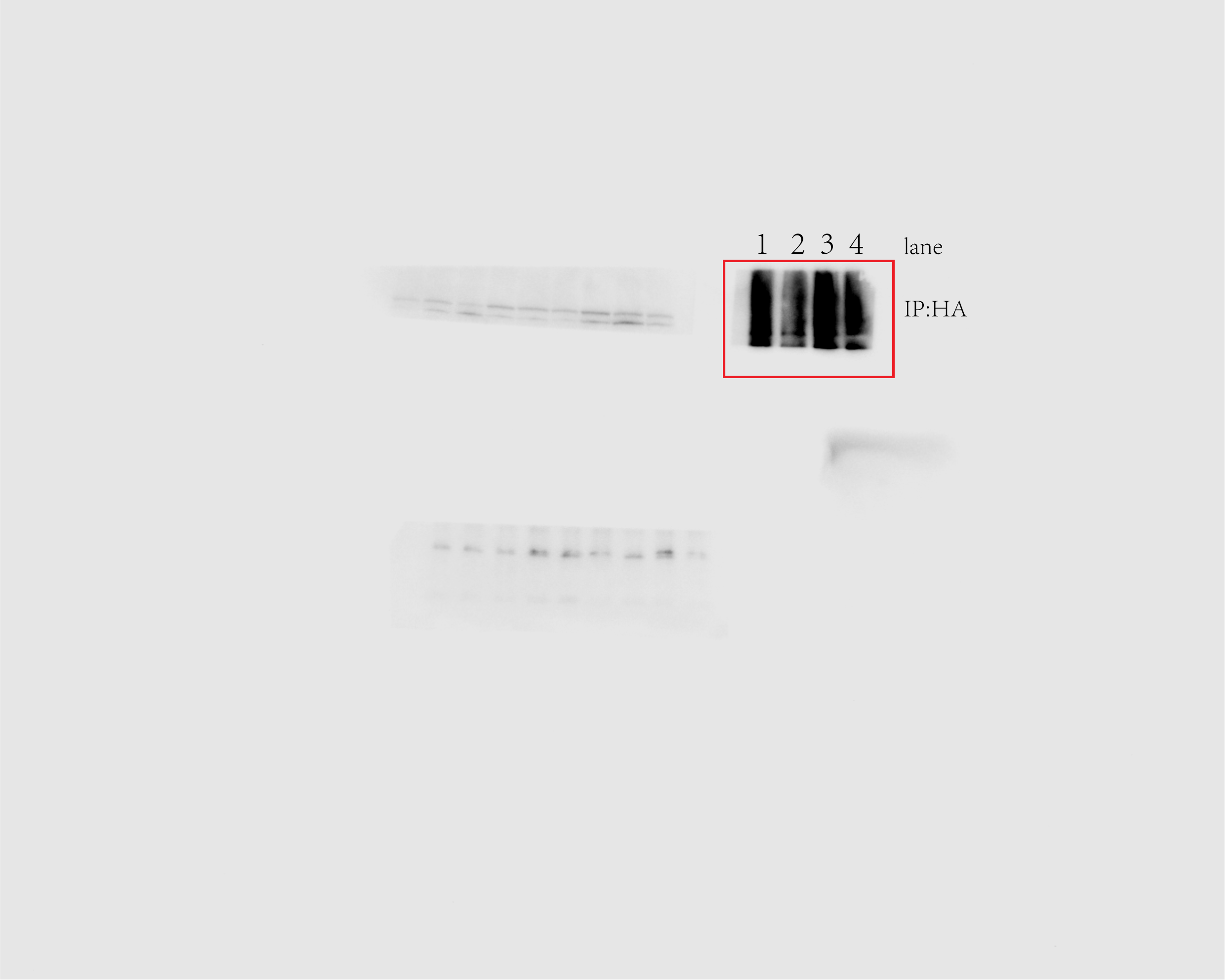

Supplement: Figure 6—source data 1. [file elife-101973-fig6-data1.zip › Figure 6-source data 1/Fig6G-labeled/IPHA.tif]

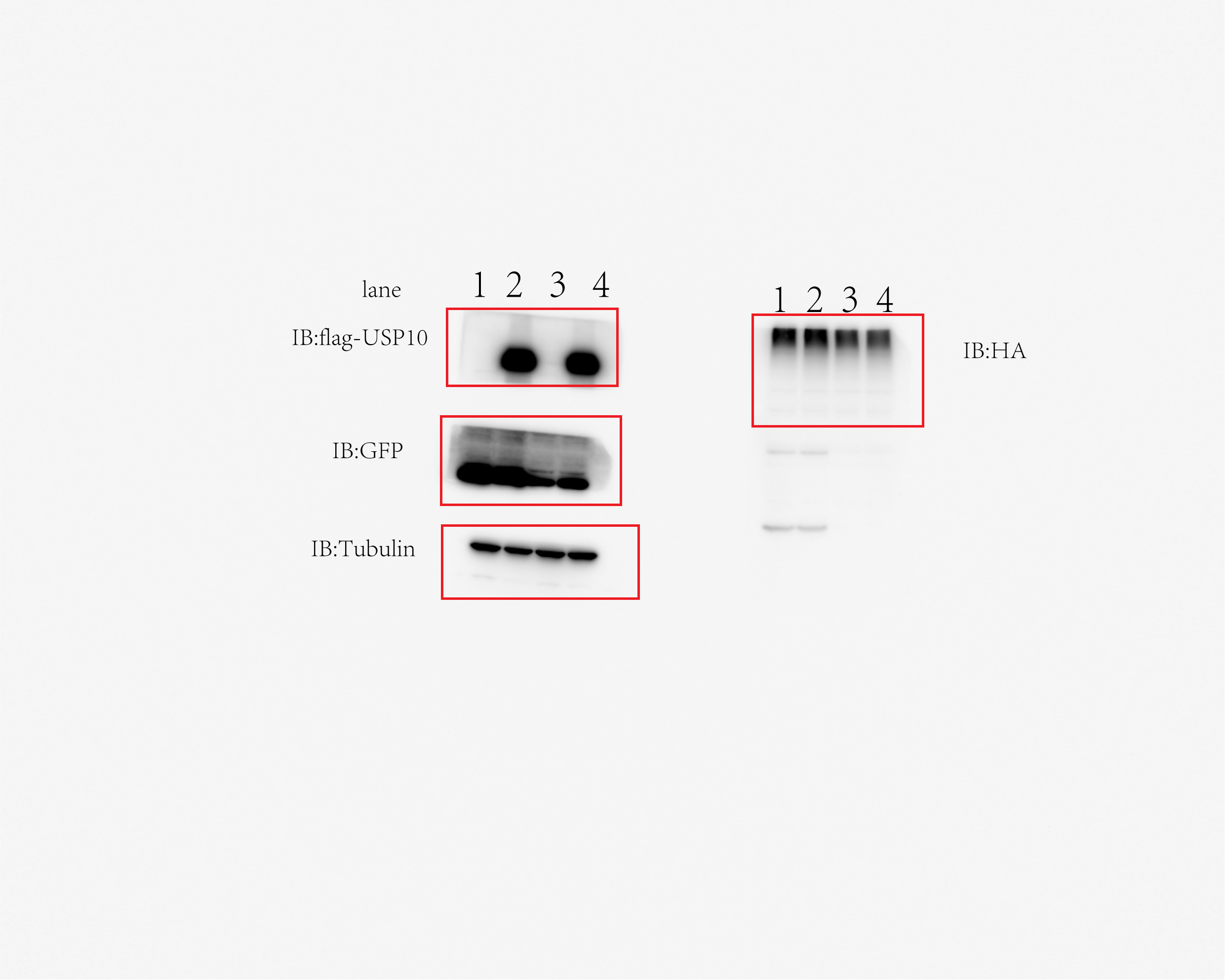

Supplement: Figure 6—source data 1. [file elife-101973-fig6-data1.zip › Figure 6-source data 1/Fig6G-labeled/input HA GFP Flag and Tubulin.tif]

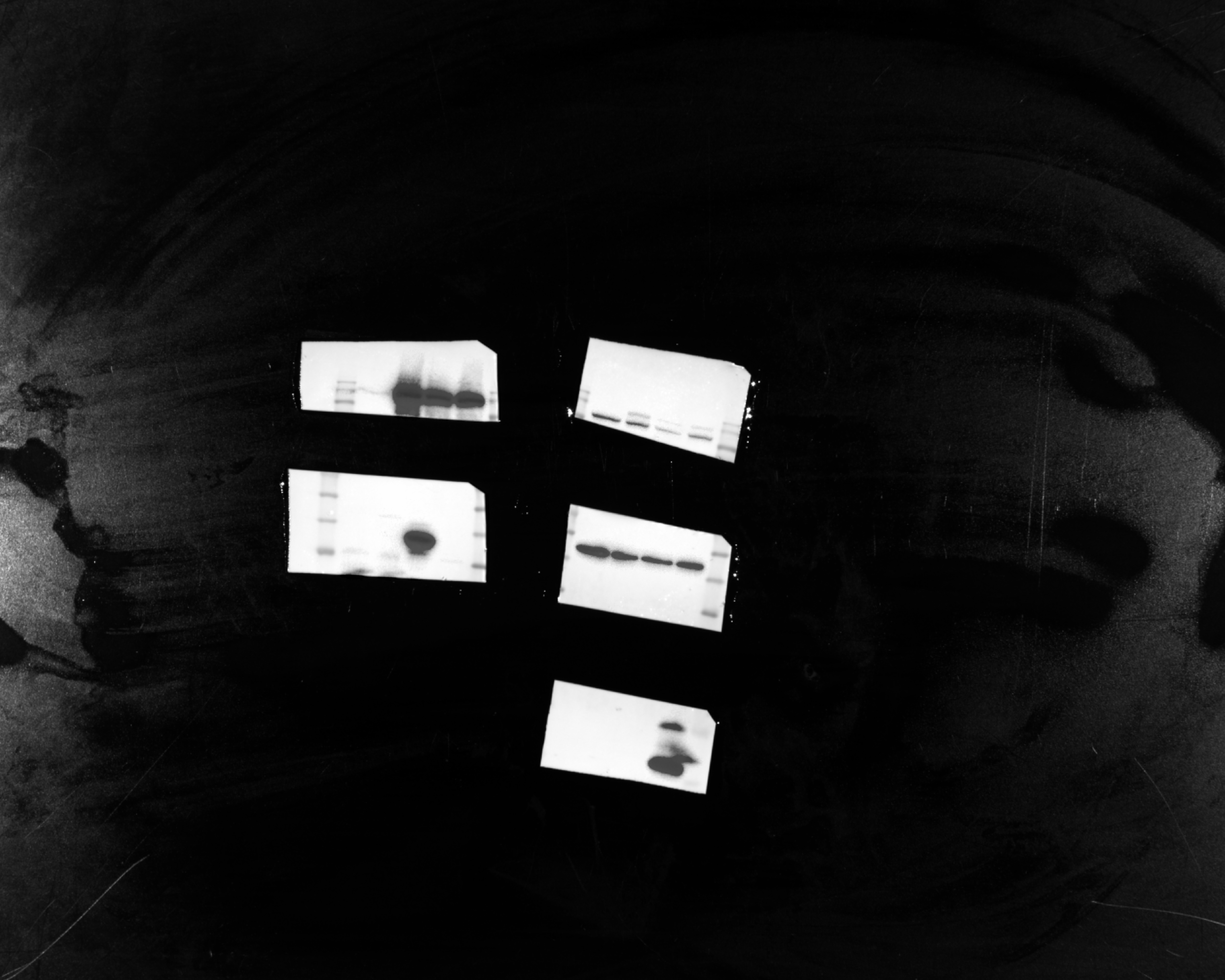

Supplement: Figure 6—source data 2. [file elife-101973-fig6-data2.zip › Figure 6-source data 2/figure 6A/input flag GFP Myc HA Tubulin.jpg]

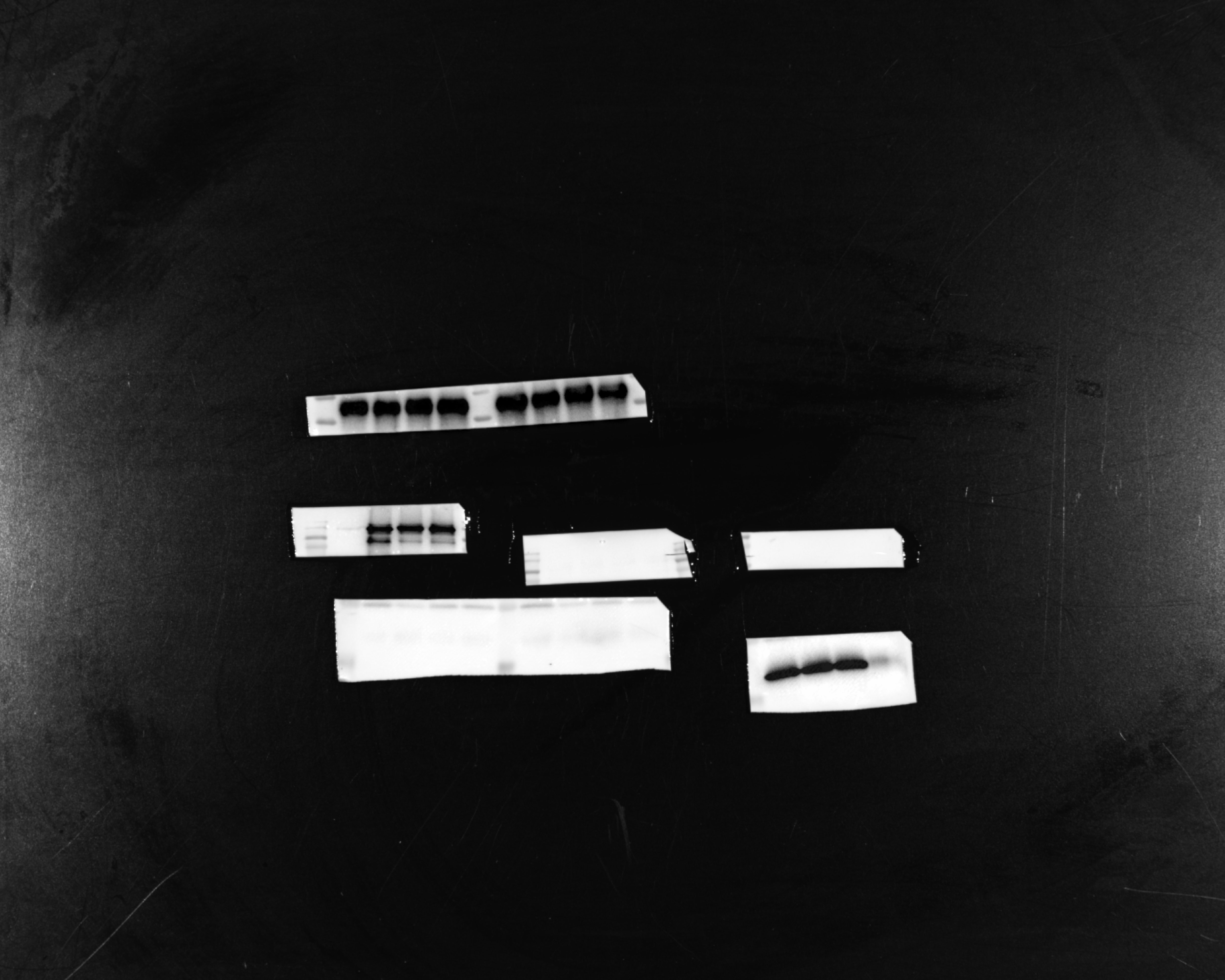

Supplement: Figure 6—source data 2. [file elife-101973-fig6-data2.zip › Figure 6-source data 2/figure 6A/ip flag.jpg]

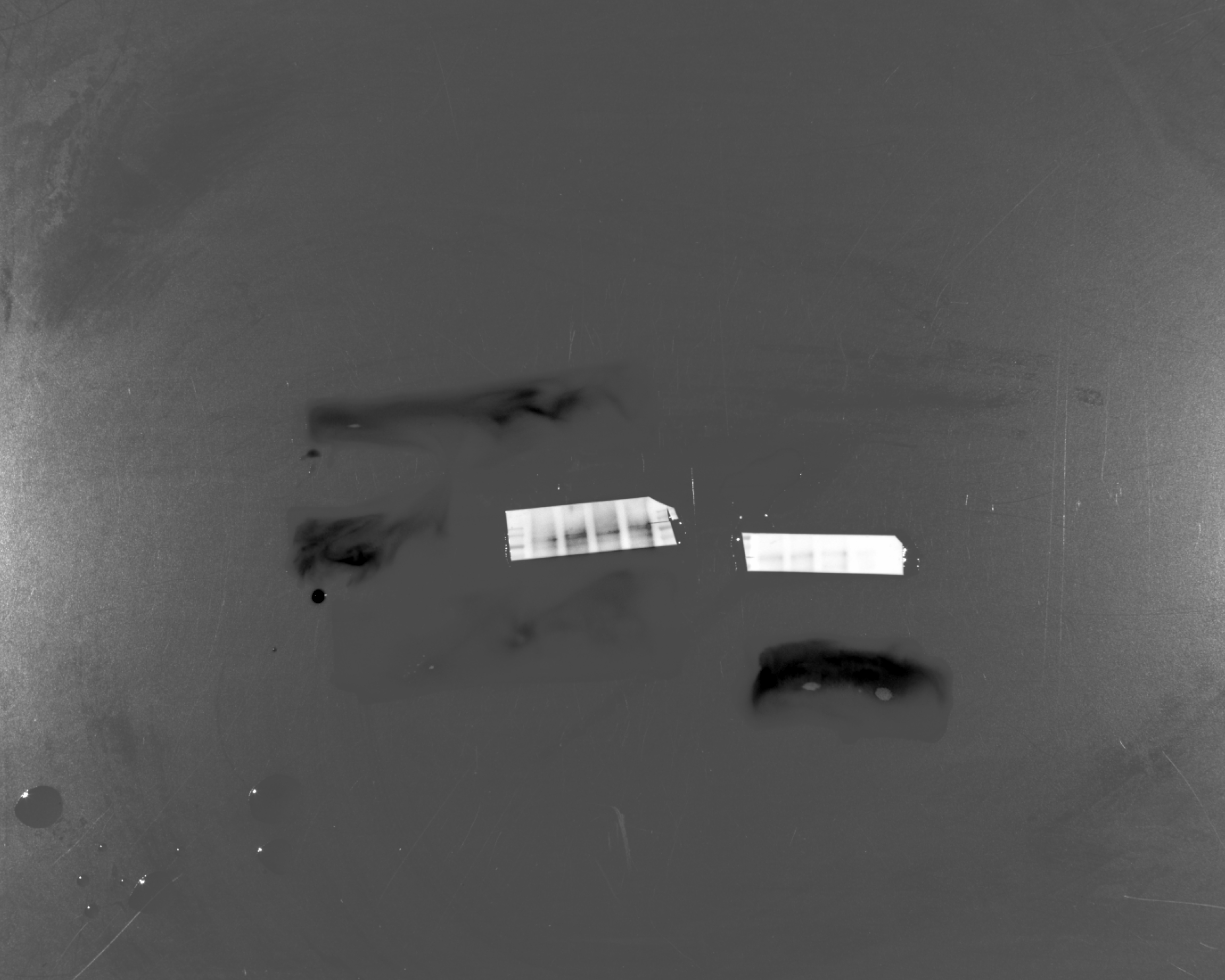

Supplement: Figure 6—source data 2. [file elife-101973-fig6-data2.zip › Figure 6-source data 2/figure 6A/ip myc.jpg]

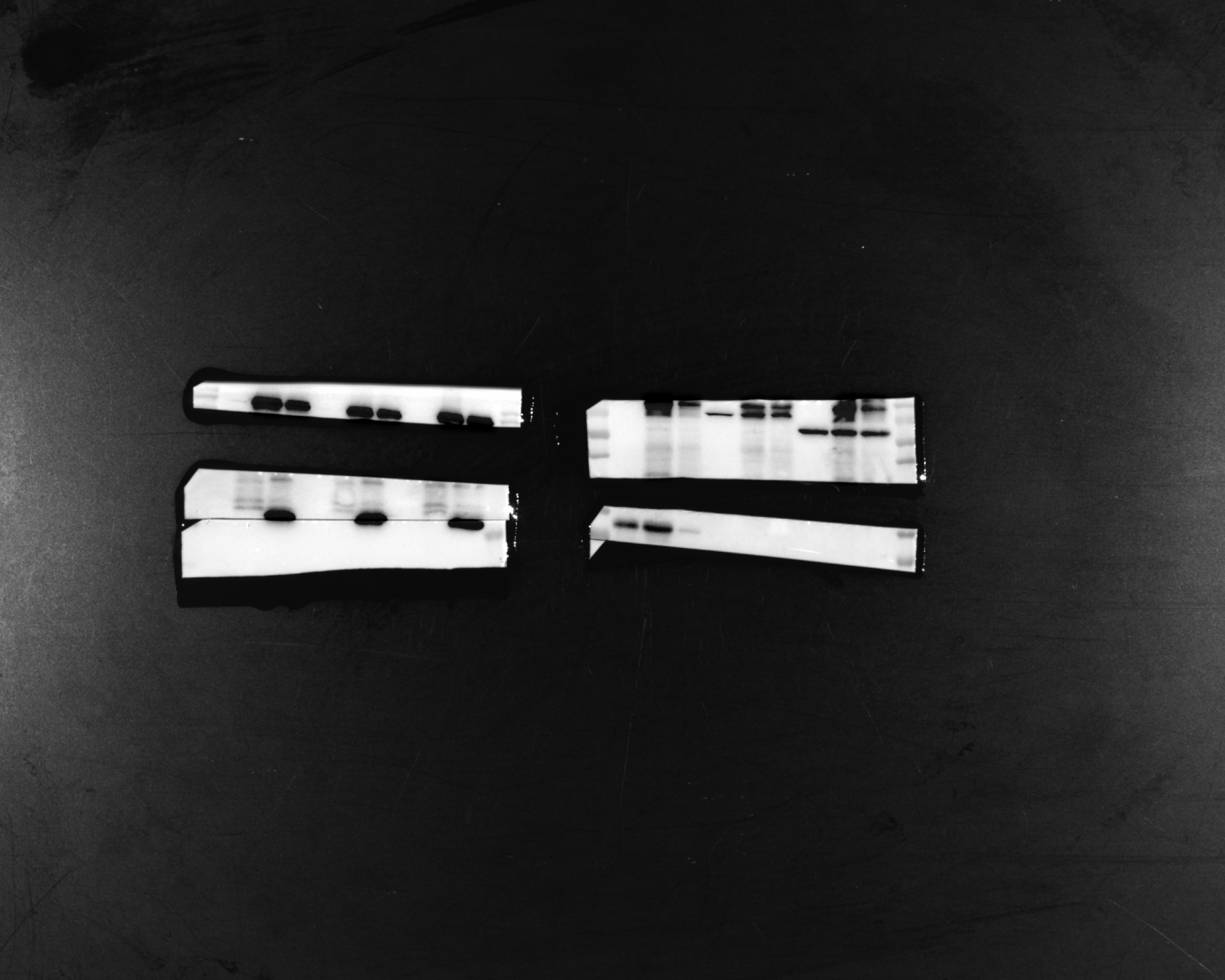

Supplement: Figure 6—source data 2. [file elife-101973-fig6-data2.zip › Figure 6-source data 2/figure 6B/flag and myc.jpg]

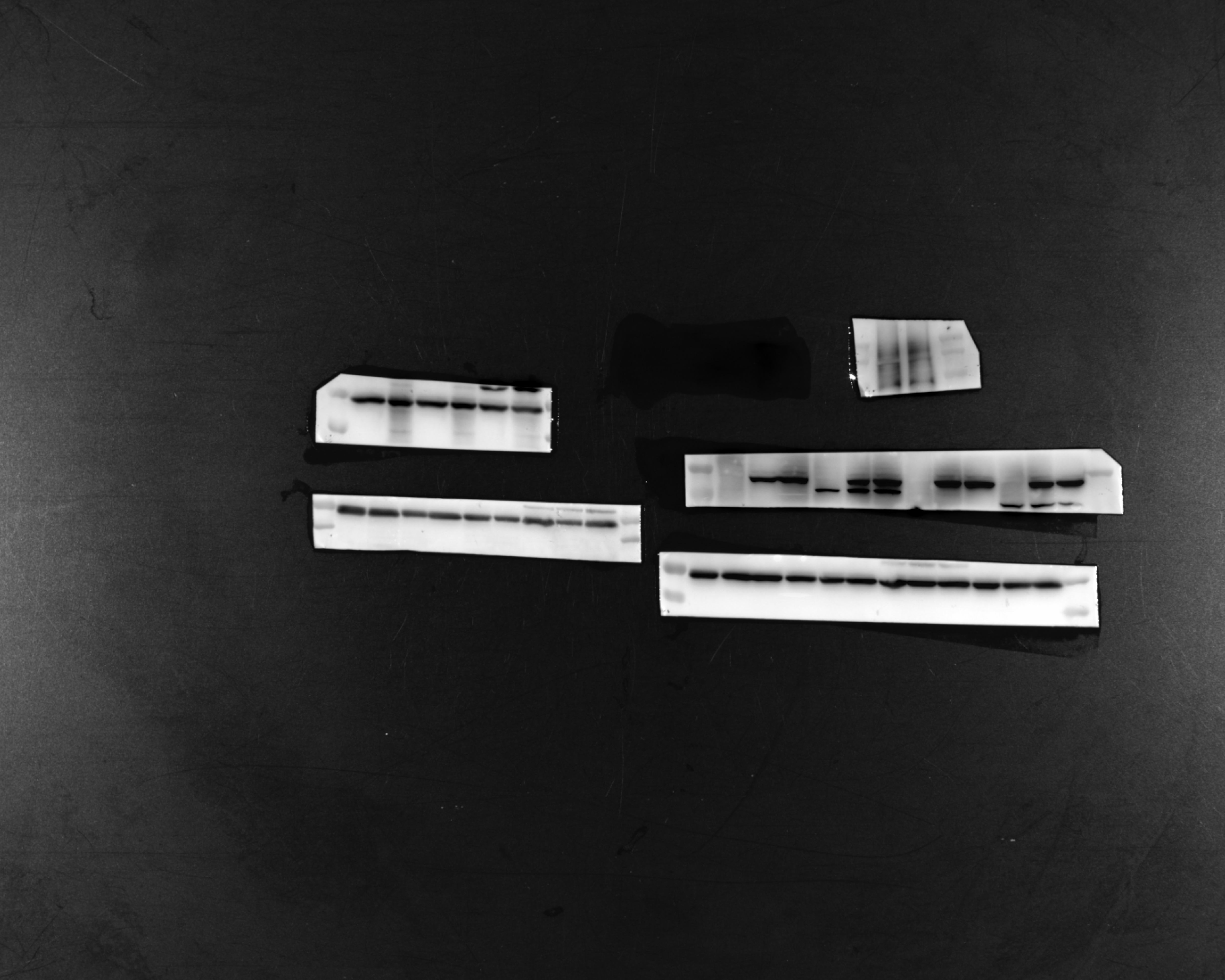

Supplement: Figure 6—source data 2. [file elife-101973-fig6-data2.zip › Figure 6-source data 2/figure 6B/tubulin.jpg]

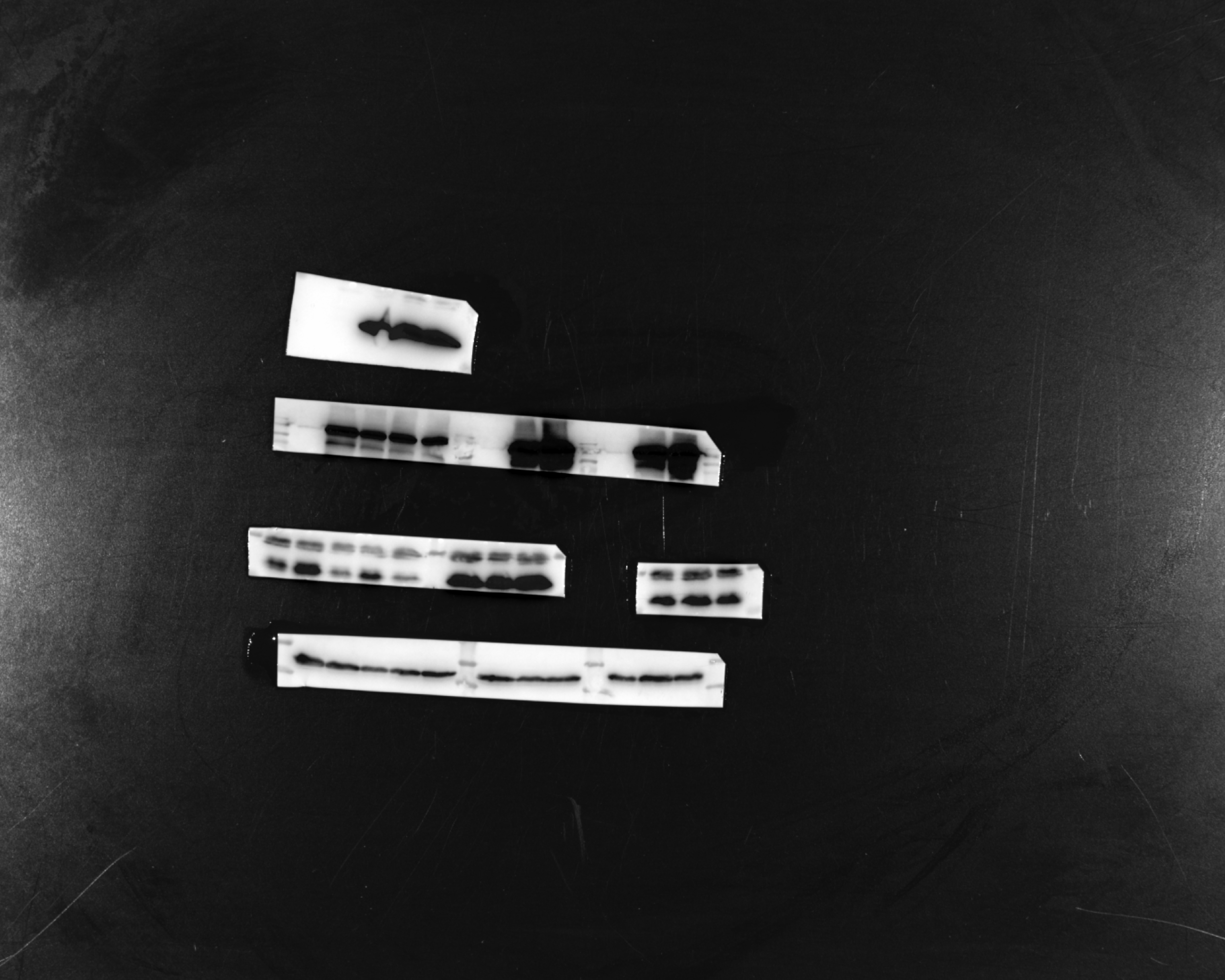

Supplement: Figure 6—source data 2. [file elife-101973-fig6-data2.zip › Figure 6-source data 2/figure 6C/myc flag and tubulin.jpg]

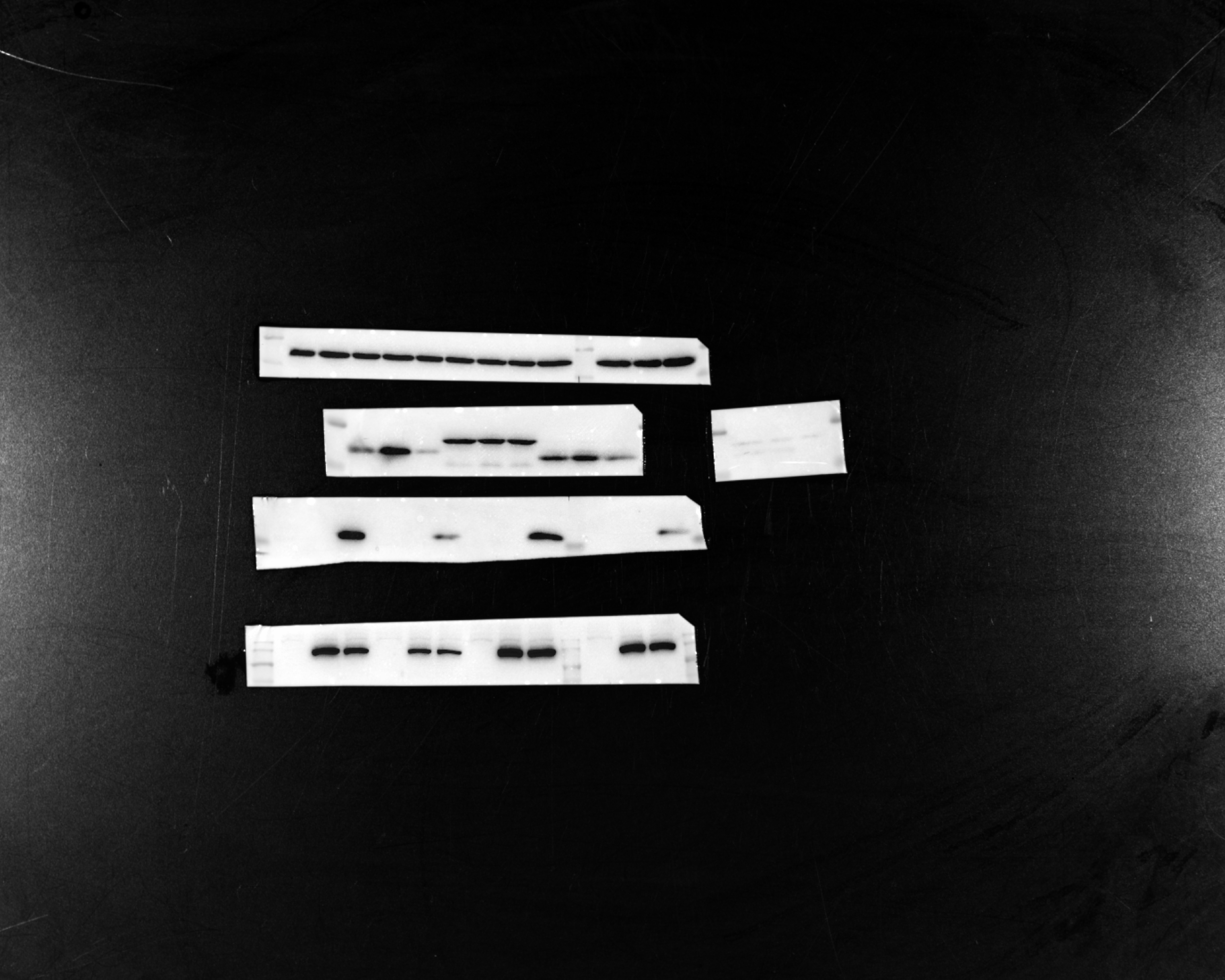

Supplement: Figure 6—source data 2. [file elife-101973-fig6-data2.zip › Figure 6-source data 2/figure 6D/Myc flag and tubulin.jpg]

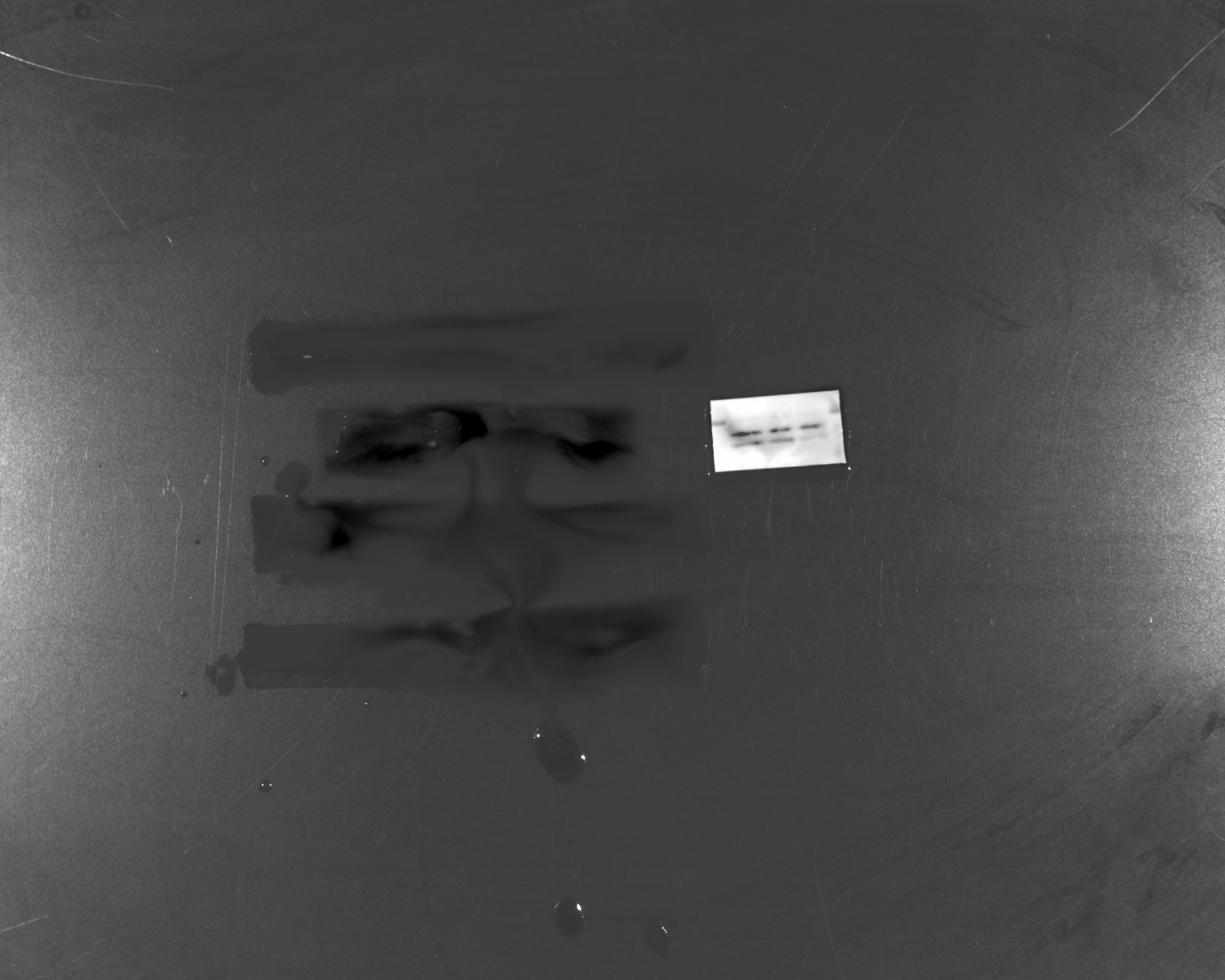

Supplement: Figure 6—source data 2. [file elife-101973-fig6-data2.zip › Figure 6-source data 2/figure 6D/long exposure of myc.jpg]

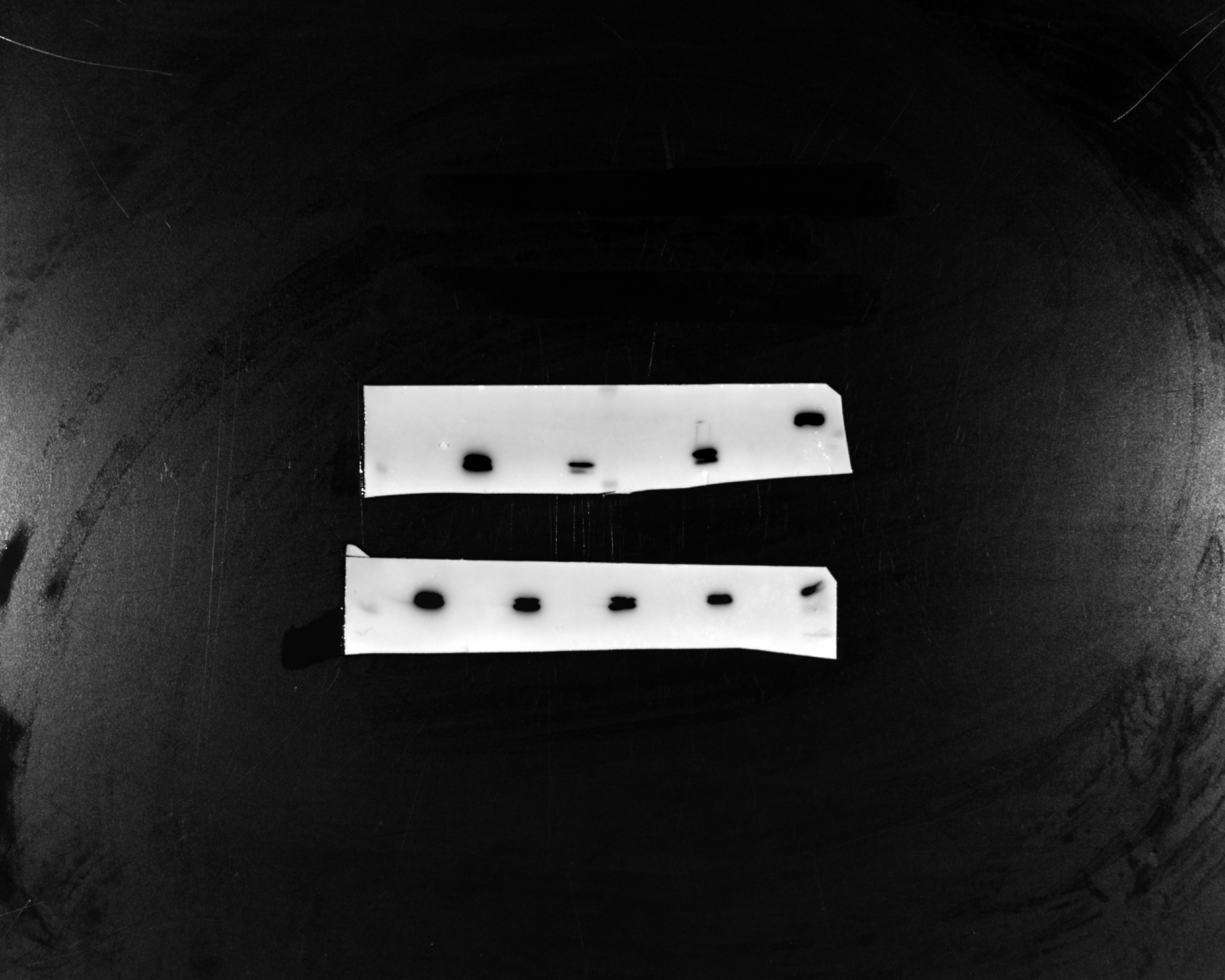

Supplement: Figure 6—source data 2. [file elife-101973-fig6-data2.zip › Figure 6-source data 2/figure 6E/Flag-ORMDL3.jpg]

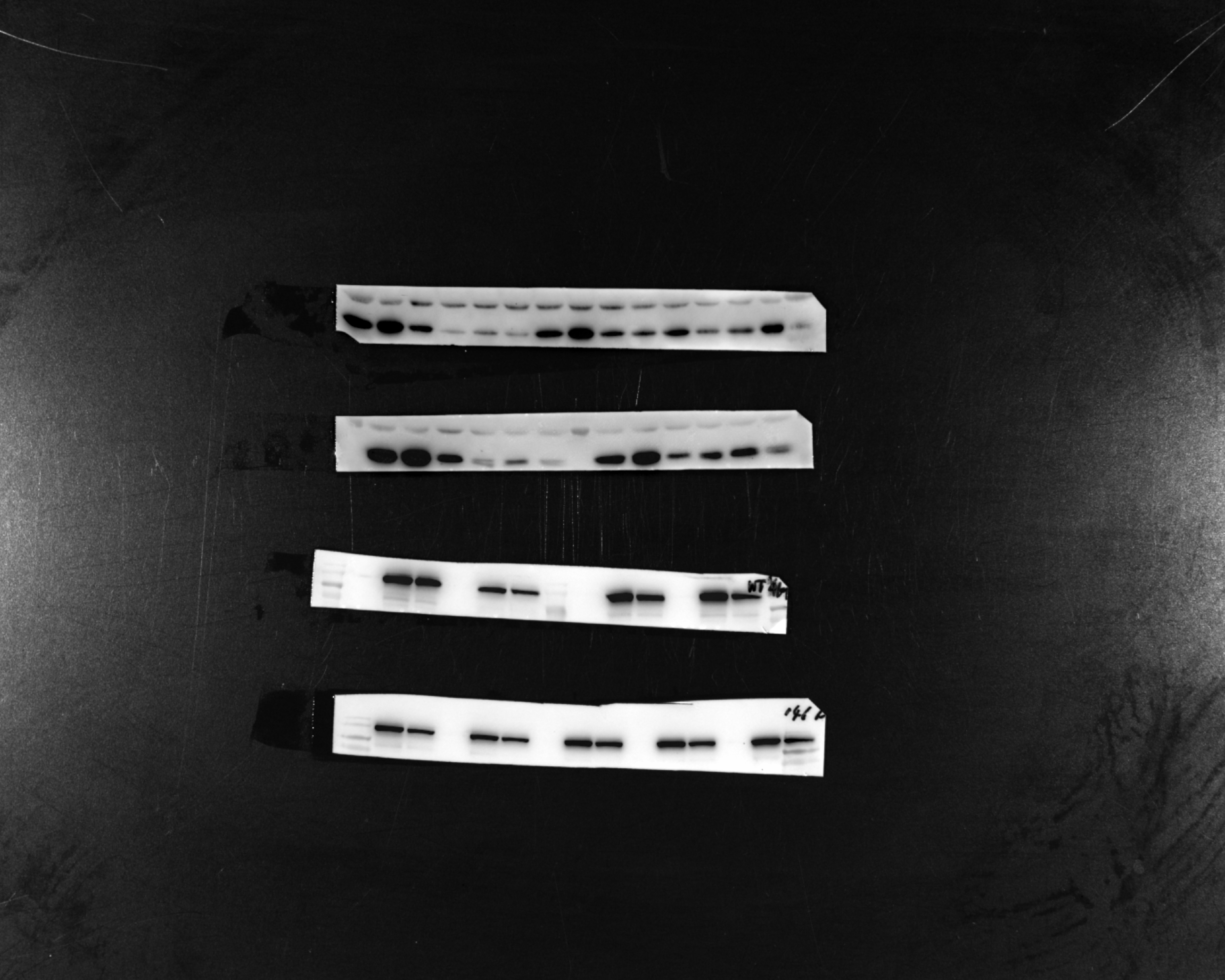

Supplement: Figure 6—source data 2. [file elife-101973-fig6-data2.zip › Figure 6-source data 2/figure 6E/Myc and flag-USP10.jpg]

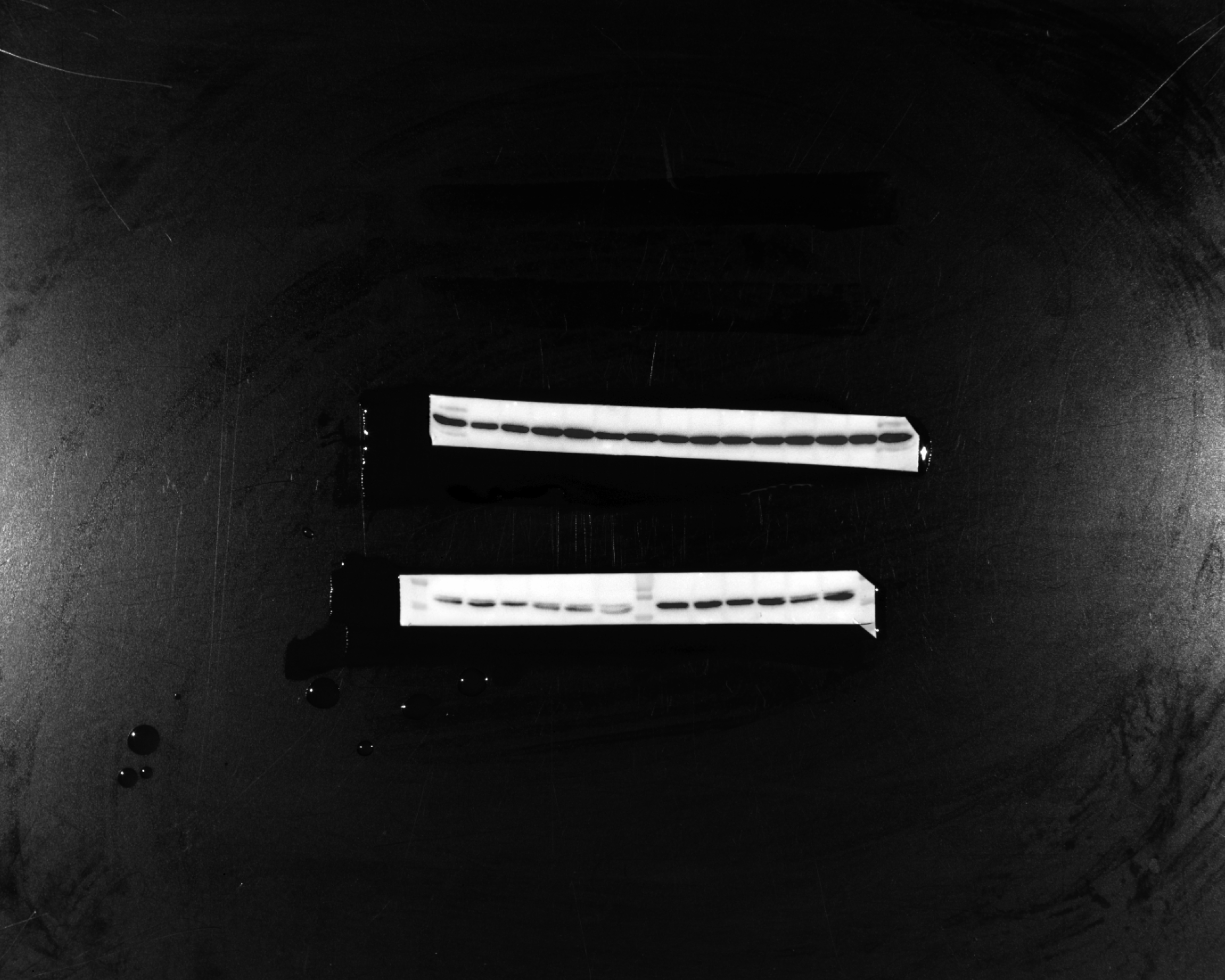

Supplement: Figure 6—source data 2. [file elife-101973-fig6-data2.zip › Figure 6-source data 2/figure 6E/Tubulin.jpg]

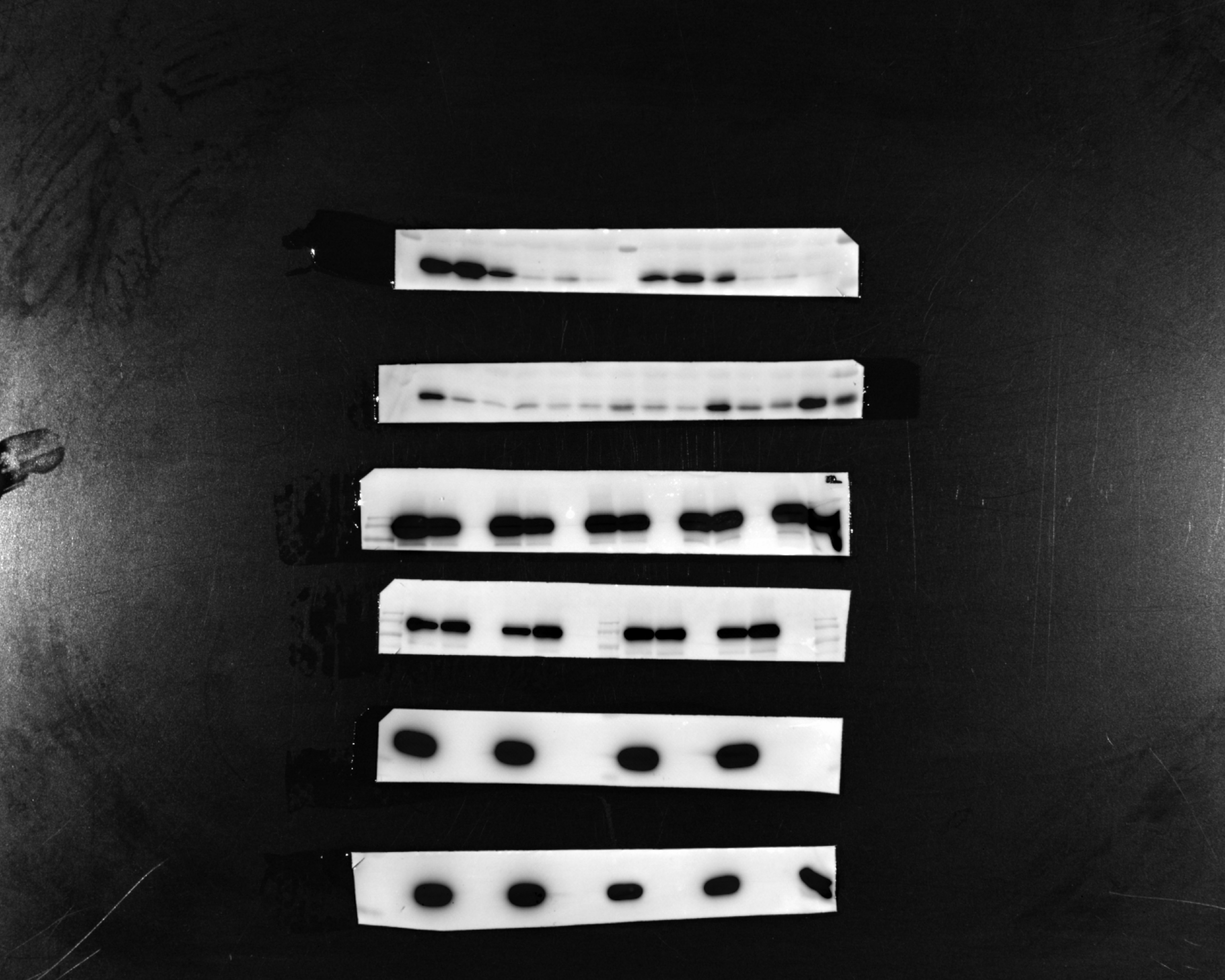

Supplement: Figure 6—source data 2. [file elife-101973-fig6-data2.zip › Figure 6-source data 2/figure 6F/lane 1 2 3 myc flag.jpg]

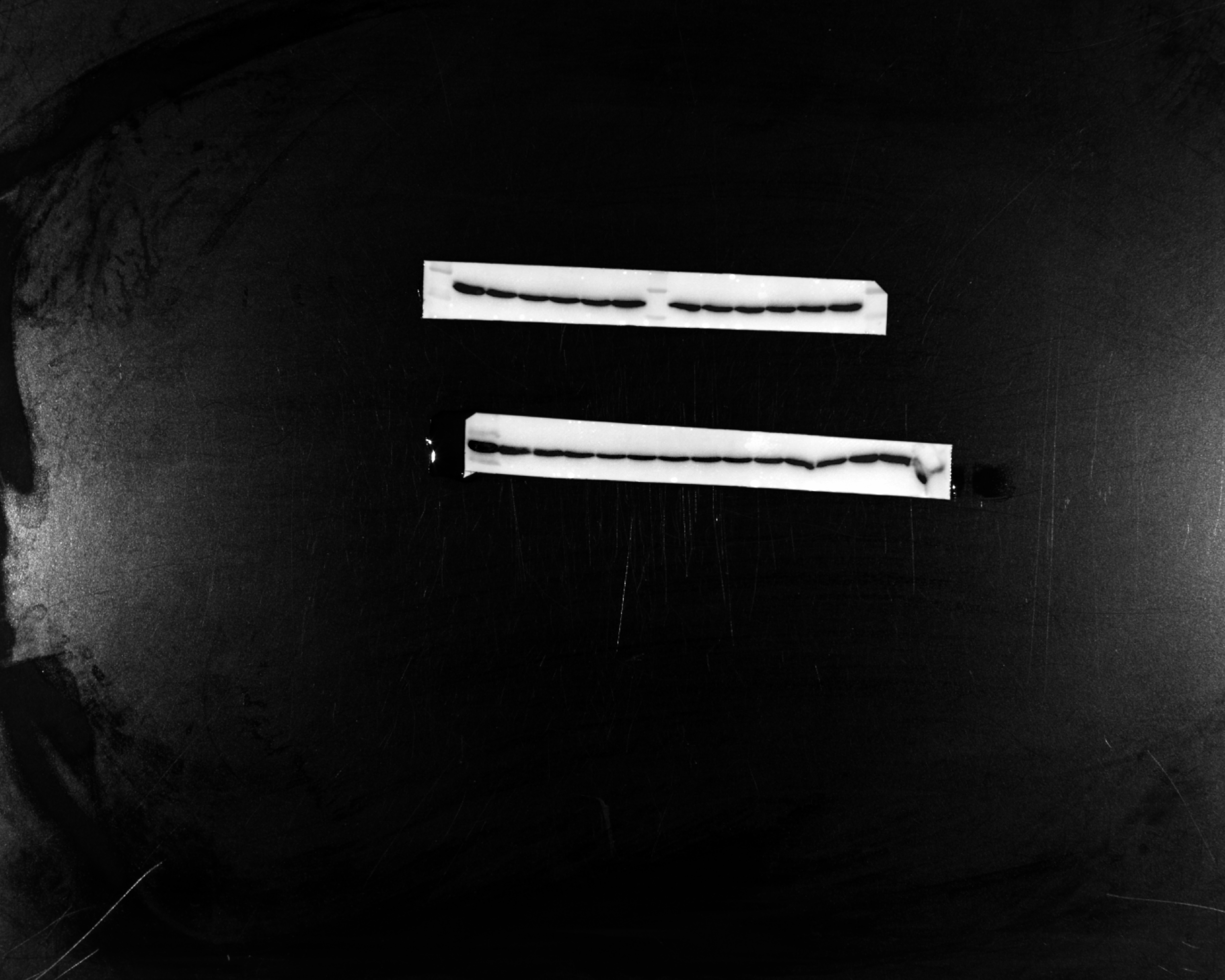

Supplement: Figure 6—source data 2. [file elife-101973-fig6-data2.zip › Figure 6-source data 2/figure 6F/lane 1 2 3 tubulin.jpg]

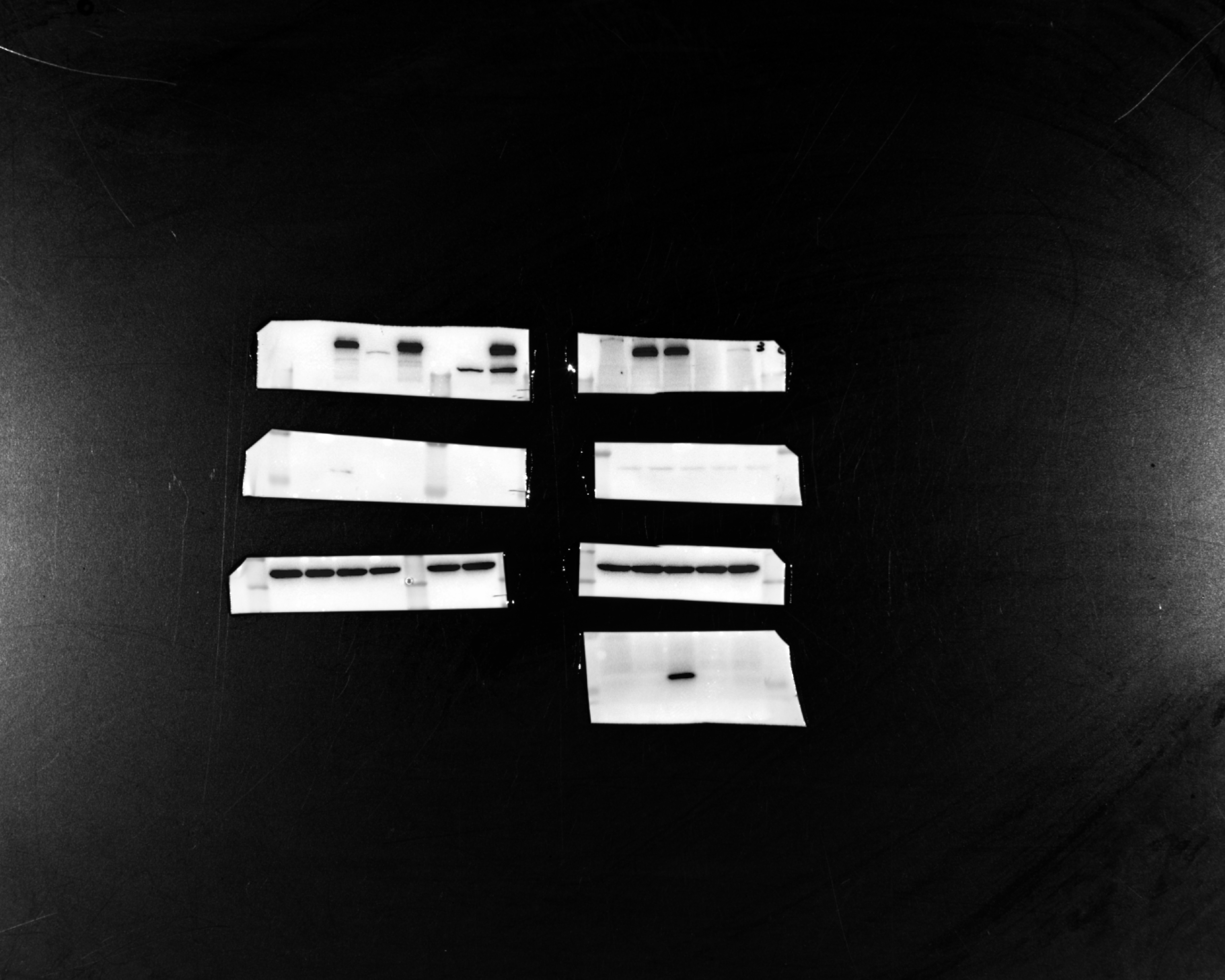

Supplement: Figure 6—source data 2. [file elife-101973-fig6-data2.zip › Figure 6-source data 2/figure 6F/lane 4 5 6 flag and tubulin.jpg]

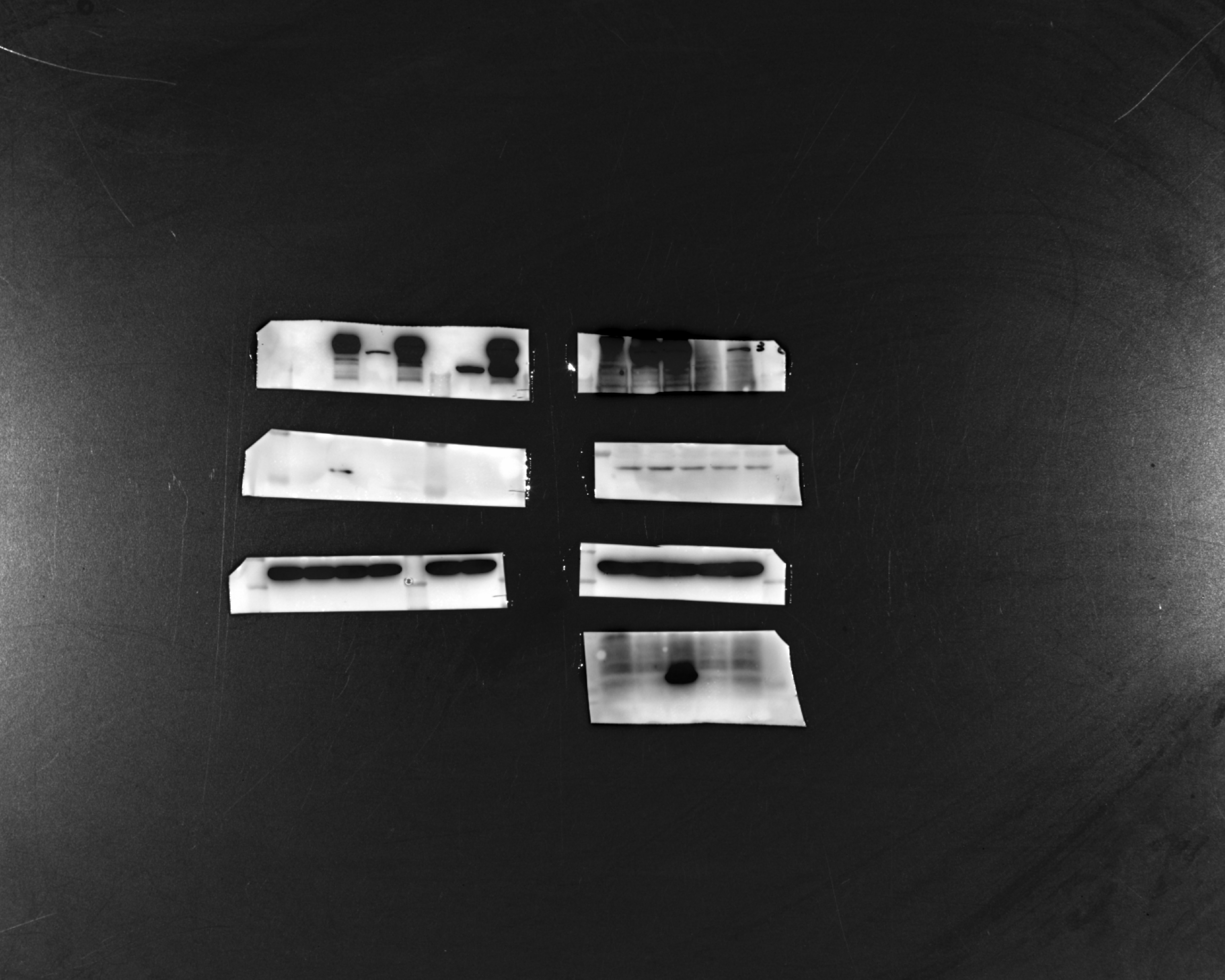

Supplement: Figure 6—source data 2. [file elife-101973-fig6-data2.zip › Figure 6-source data 2/figure 6F/lane 4 5 6 long exposure of RIG-I-4KR-Myc.jpg]

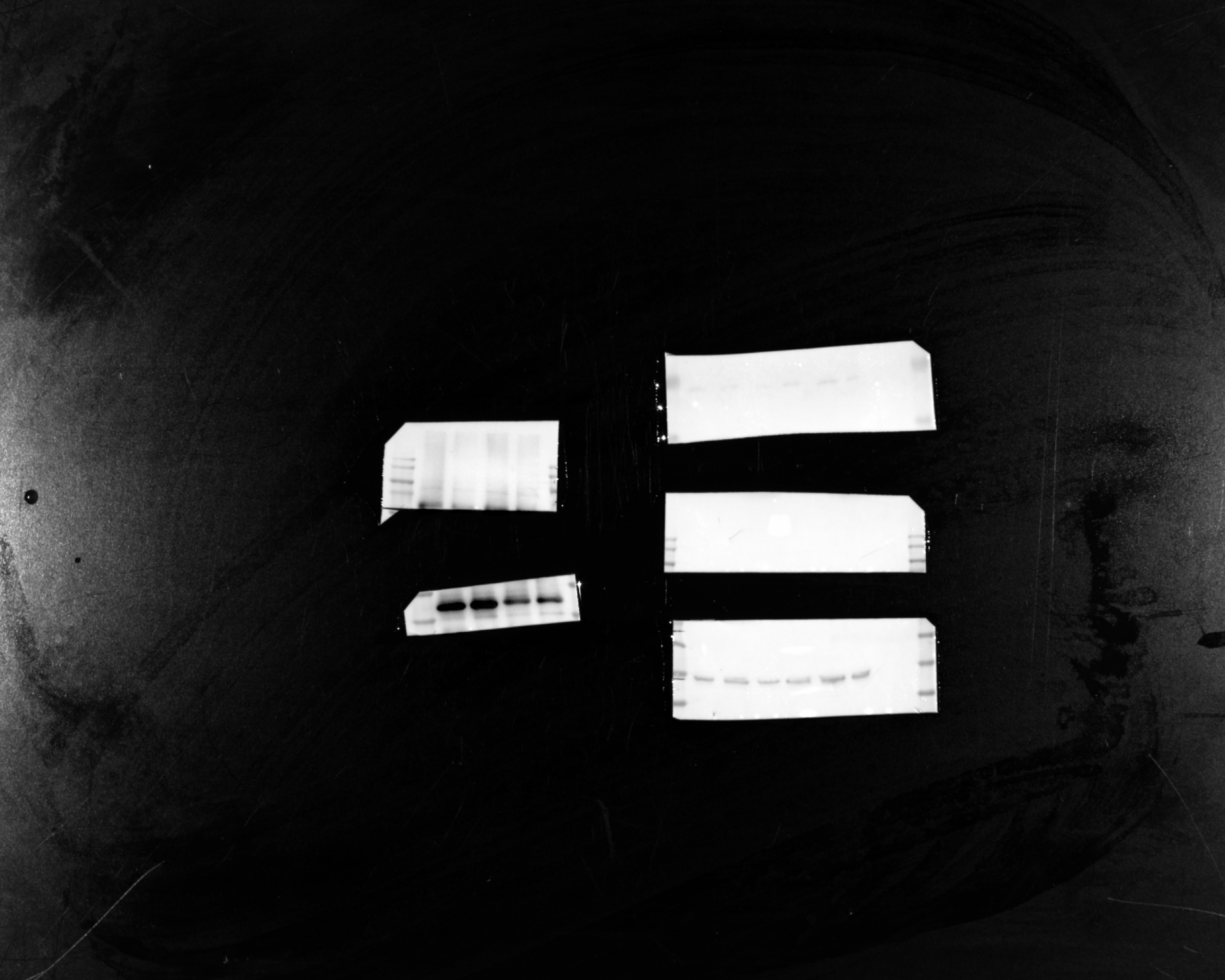

Supplement: Figure 6—source data 2. [file elife-101973-fig6-data2.zip › Figure 6-source data 2/figure 6G/IP GFP.jpg]

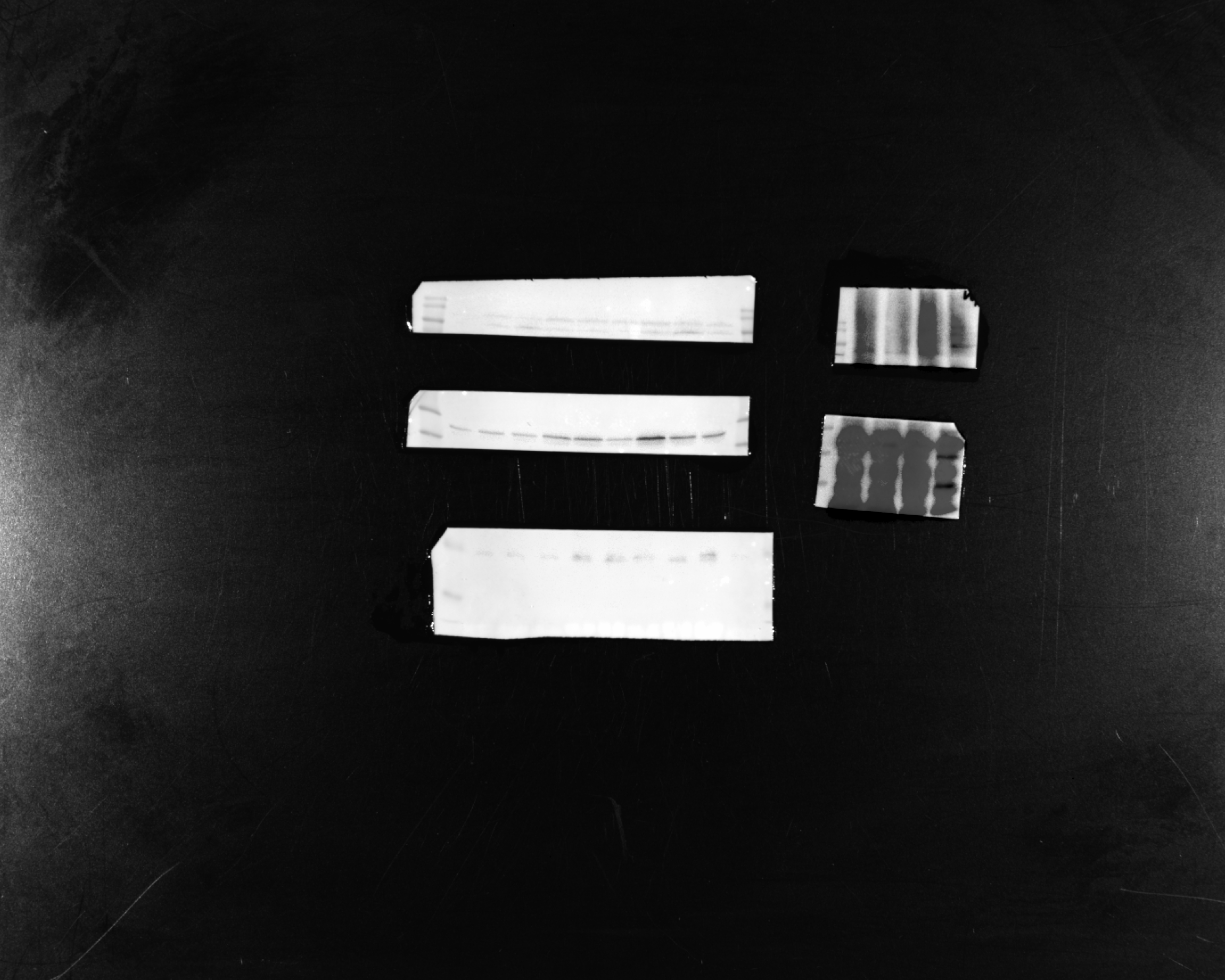

Supplement: Figure 6—source data 2. [file elife-101973-fig6-data2.zip › Figure 6-source data 2/figure 6G/IP HA .jpg]

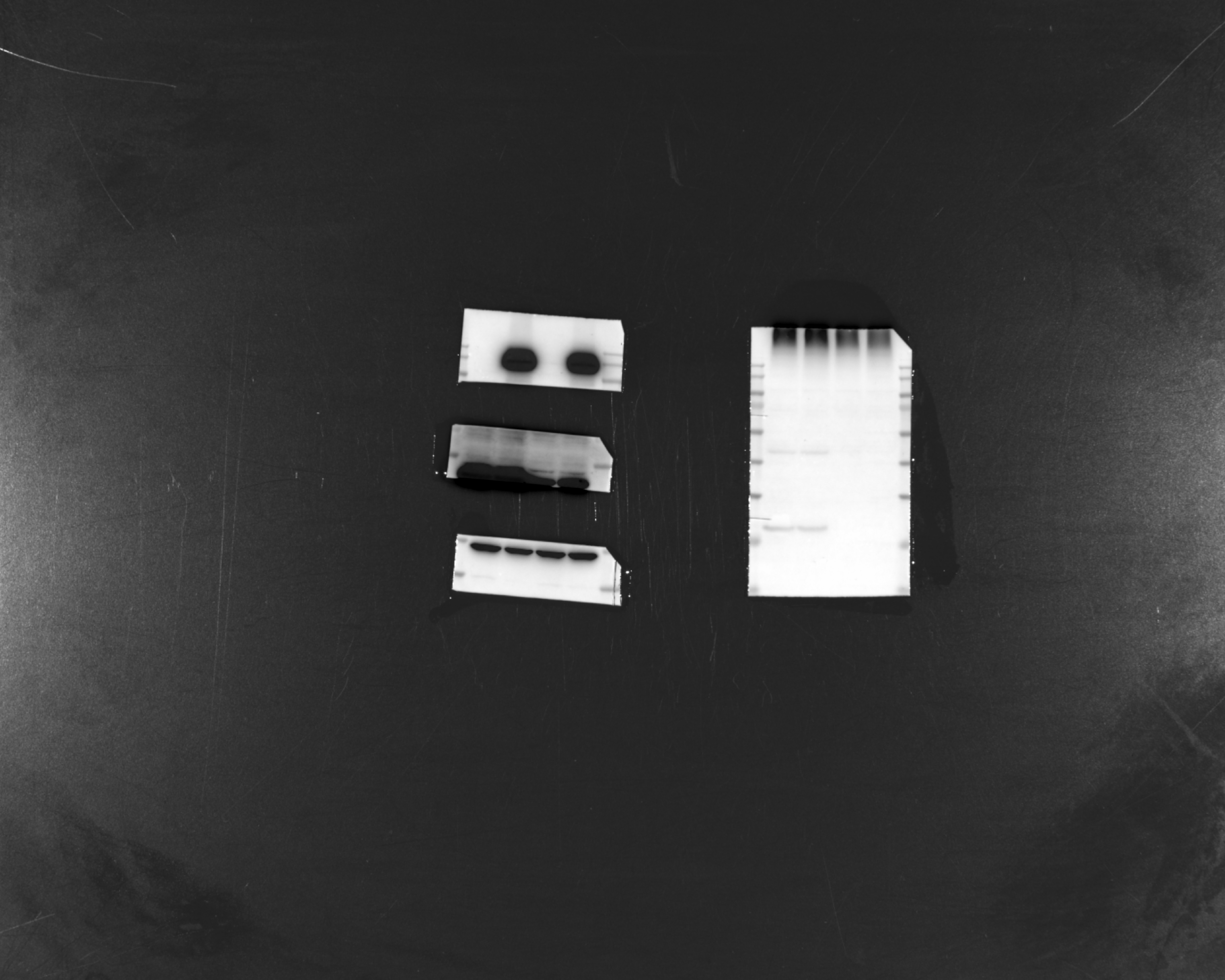

Supplement: Figure 6—source data 2. [file elife-101973-fig6-data2.zip › Figure 6-source data 2/figure 6G/input HA Flag and GFP tubulin.jpg]

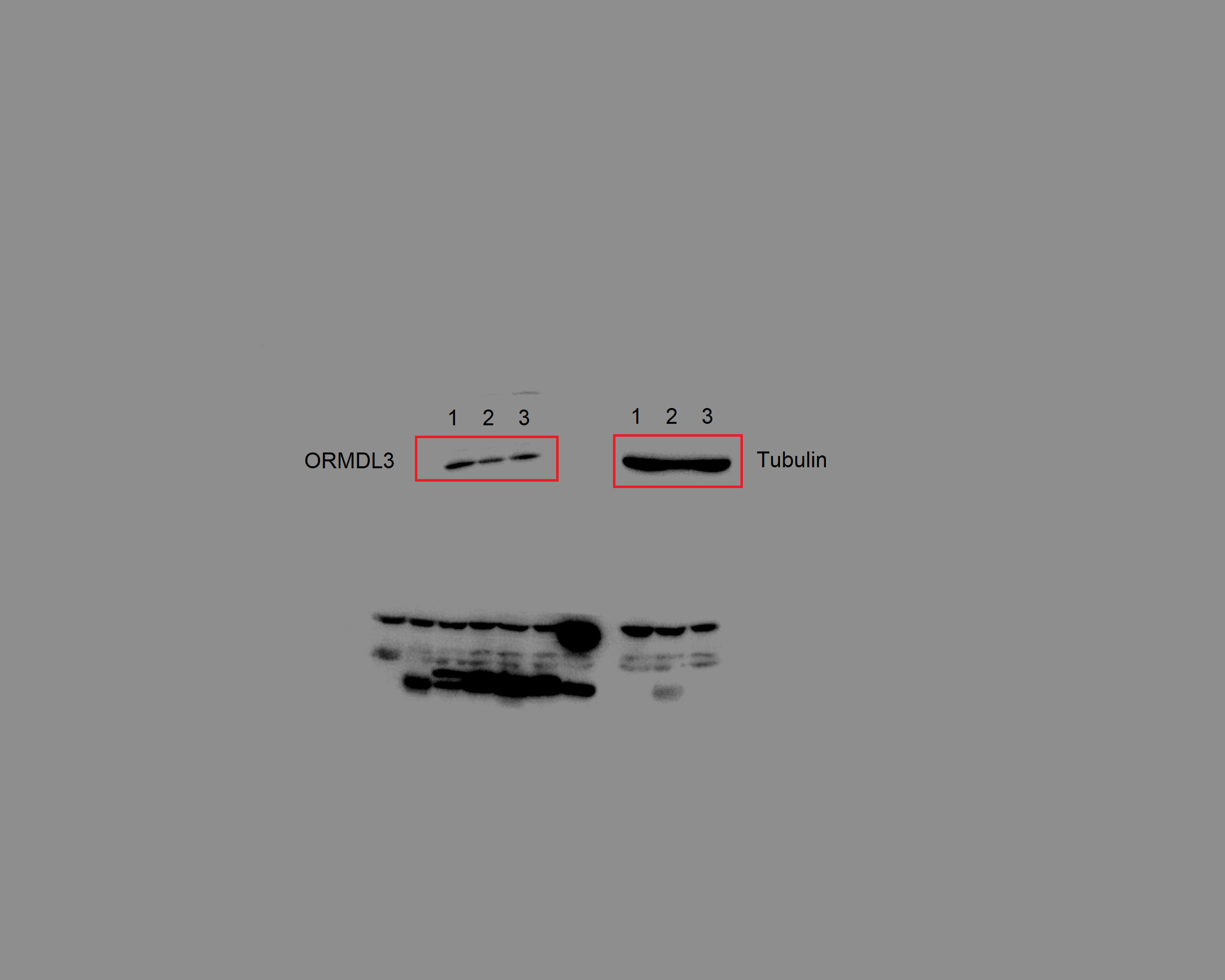

Supplement: Figure 7—figure supplement 1—source data 1. [file elife-101973-fig7-figsupp1-data1.zip › Figure 7-figure supplement 1-source data 1/Figure 7-figure supplement 1B-labeled/ORMDL3 and Tubulin.tif]

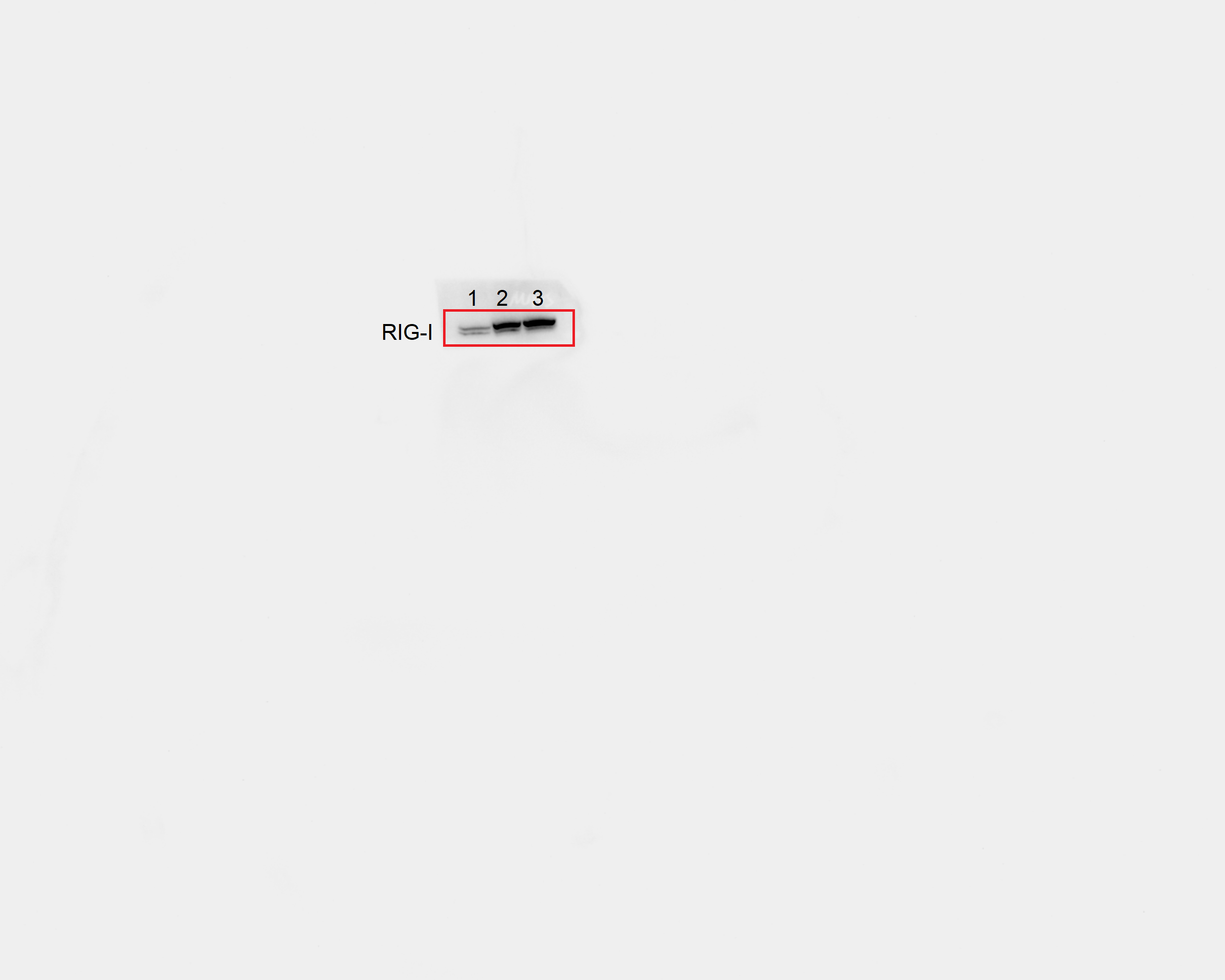

Supplement: Figure 7—figure supplement 1—source data 1. [file elife-101973-fig7-figsupp1-data1.zip › Figure 7-figure supplement 1-source data 1/Figure 7-figure supplement 1B-labeled/RIG-I.tif]

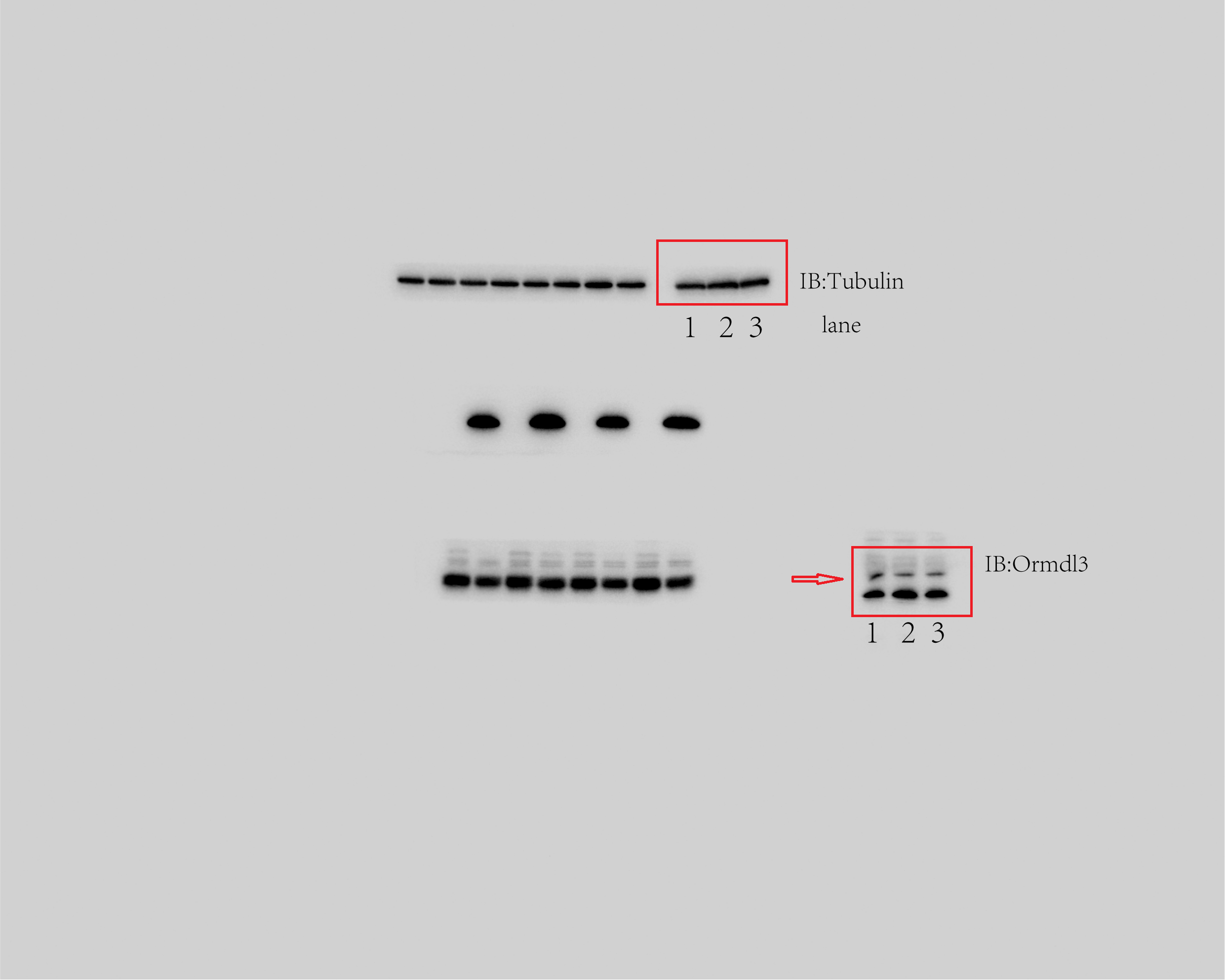

Supplement: Figure 7—figure supplement 1—source data 1. [file elife-101973-fig7-figsupp1-data1.zip › Figure 7-figure supplement 1-source data 1/Figure 7-figure supplement 1D-labeled/ORMDL3 and Tubulin.tif]

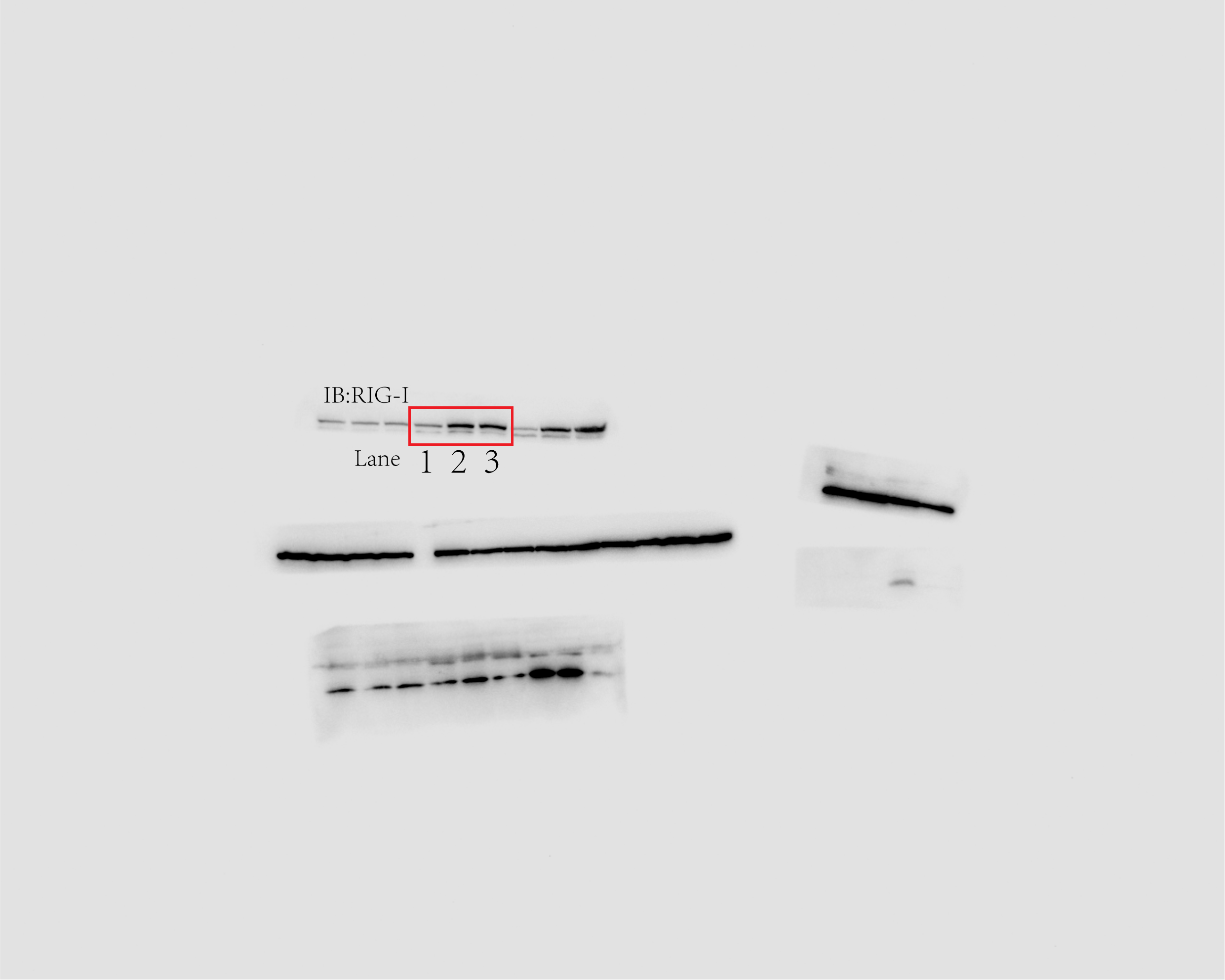

Supplement: Figure 7—figure supplement 1—source data 1. [file elife-101973-fig7-figsupp1-data1.zip › Figure 7-figure supplement 1-source data 1/Figure 7-figure supplement 1D-labeled/RIG-I.tif]

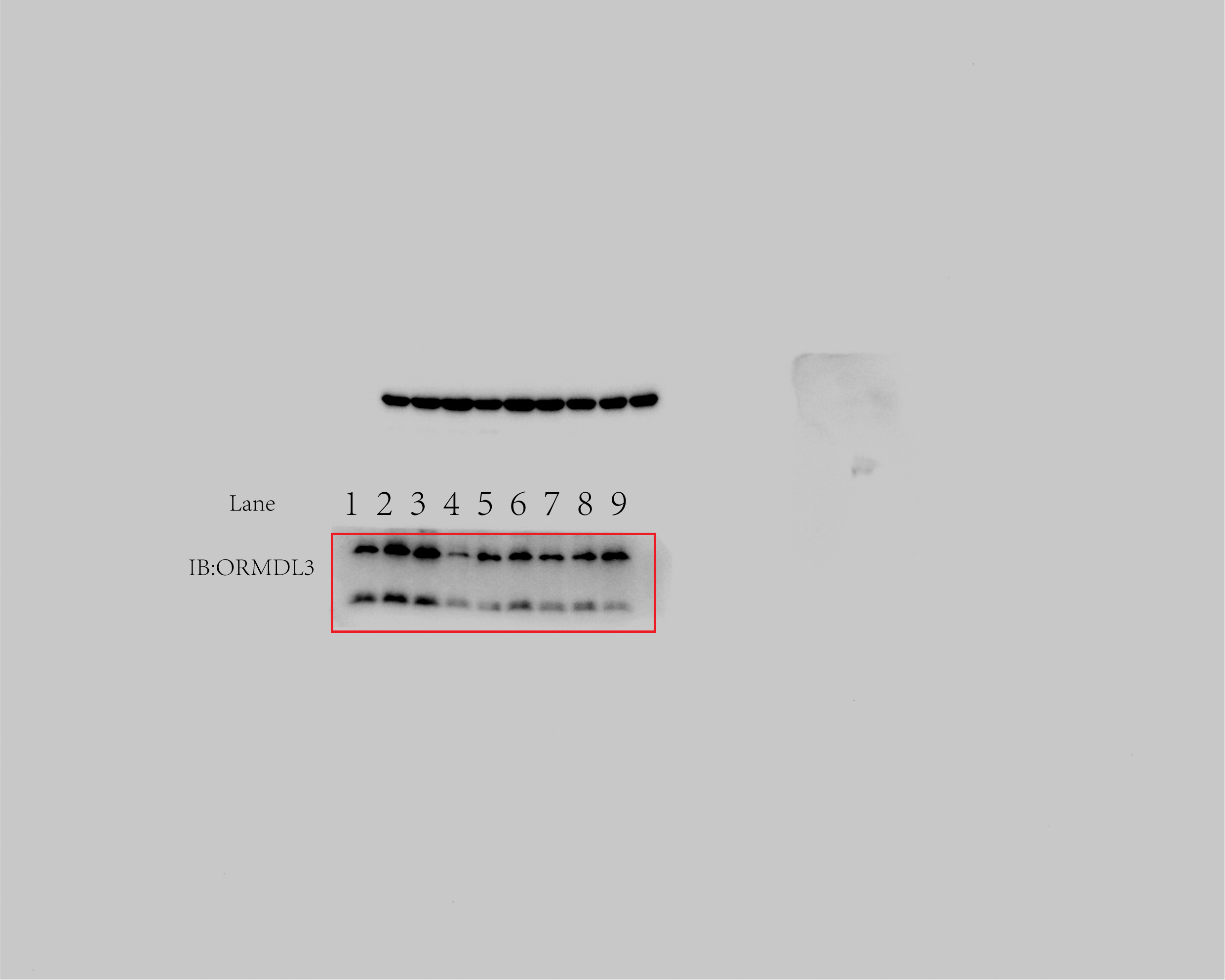

Supplement: Figure 7—figure supplement 1—source data 1. [file elife-101973-fig7-figsupp1-data1.zip › Figure 7-figure supplement 1-source data 1/Figure 7-figure supplement 1E-labeled/ORMDL3.tif]

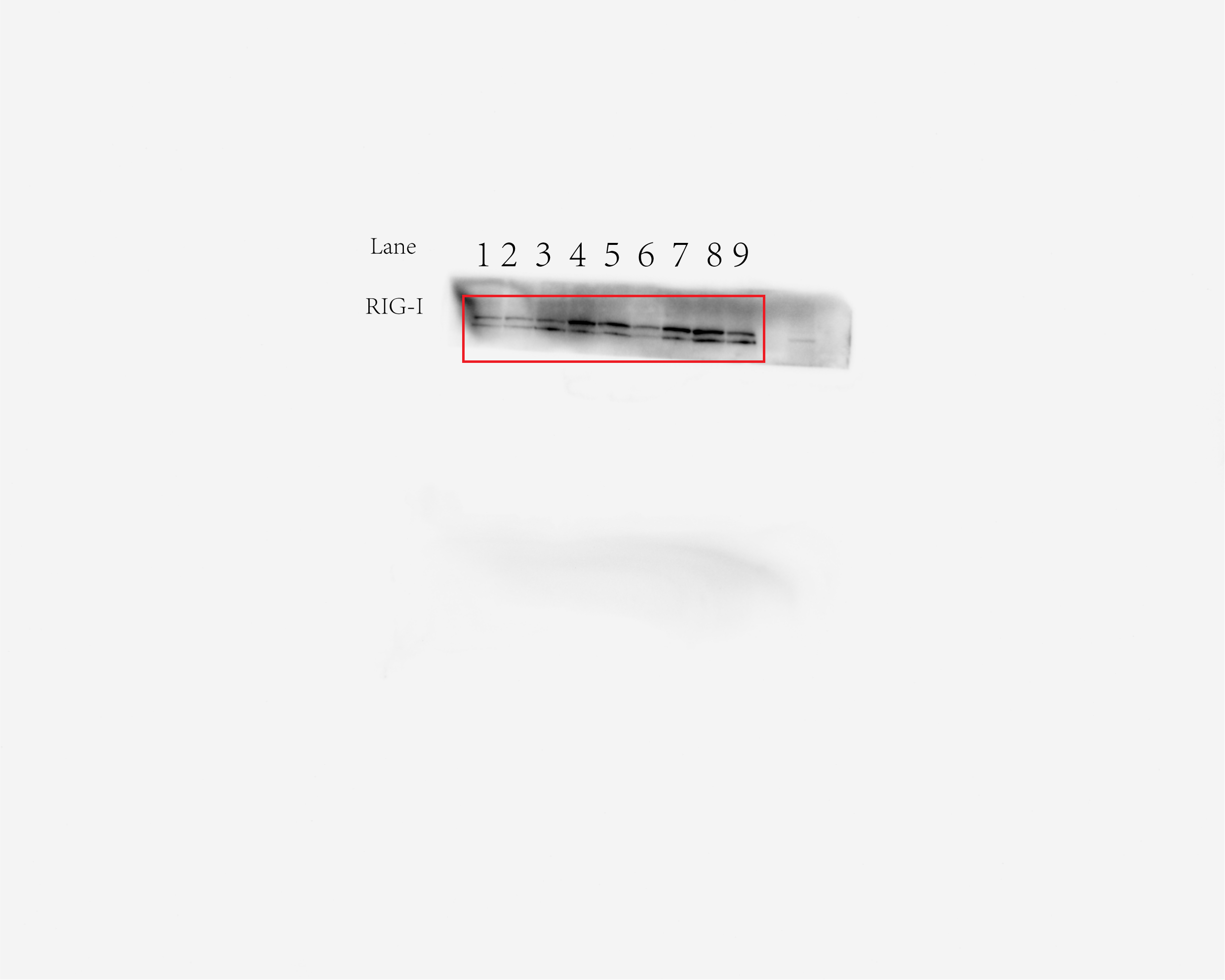

Supplement: Figure 7—figure supplement 1—source data 1. [file elife-101973-fig7-figsupp1-data1.zip › Figure 7-figure supplement 1-source data 1/Figure 7-figure supplement 1E-labeled/RIG-I.tif]

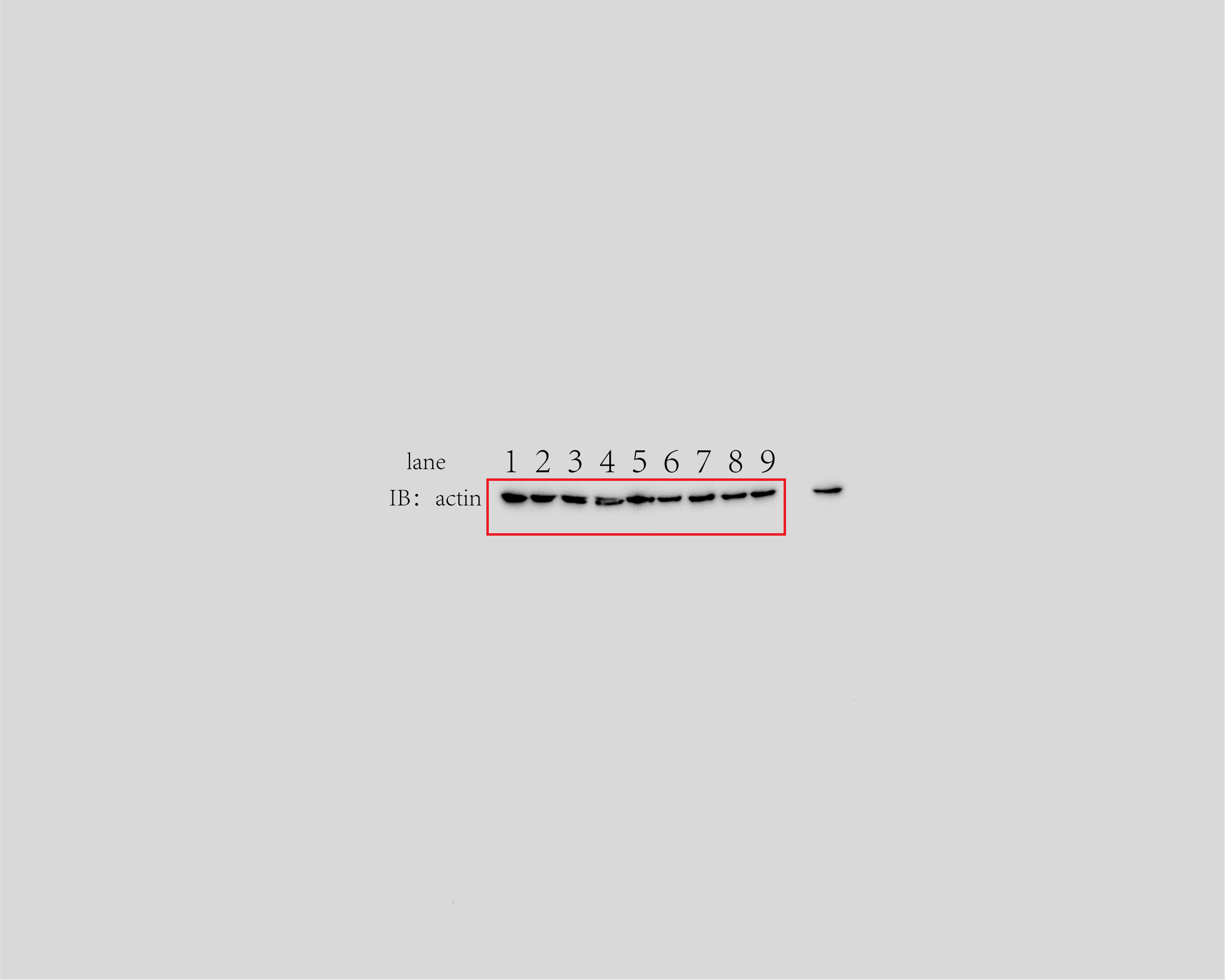

Supplement: Figure 7—figure supplement 1—source data 1. [file elife-101973-fig7-figsupp1-data1.zip › Figure 7-figure supplement 1-source data 1/Figure 7-figure supplement 1E-labeled/actin.tif]

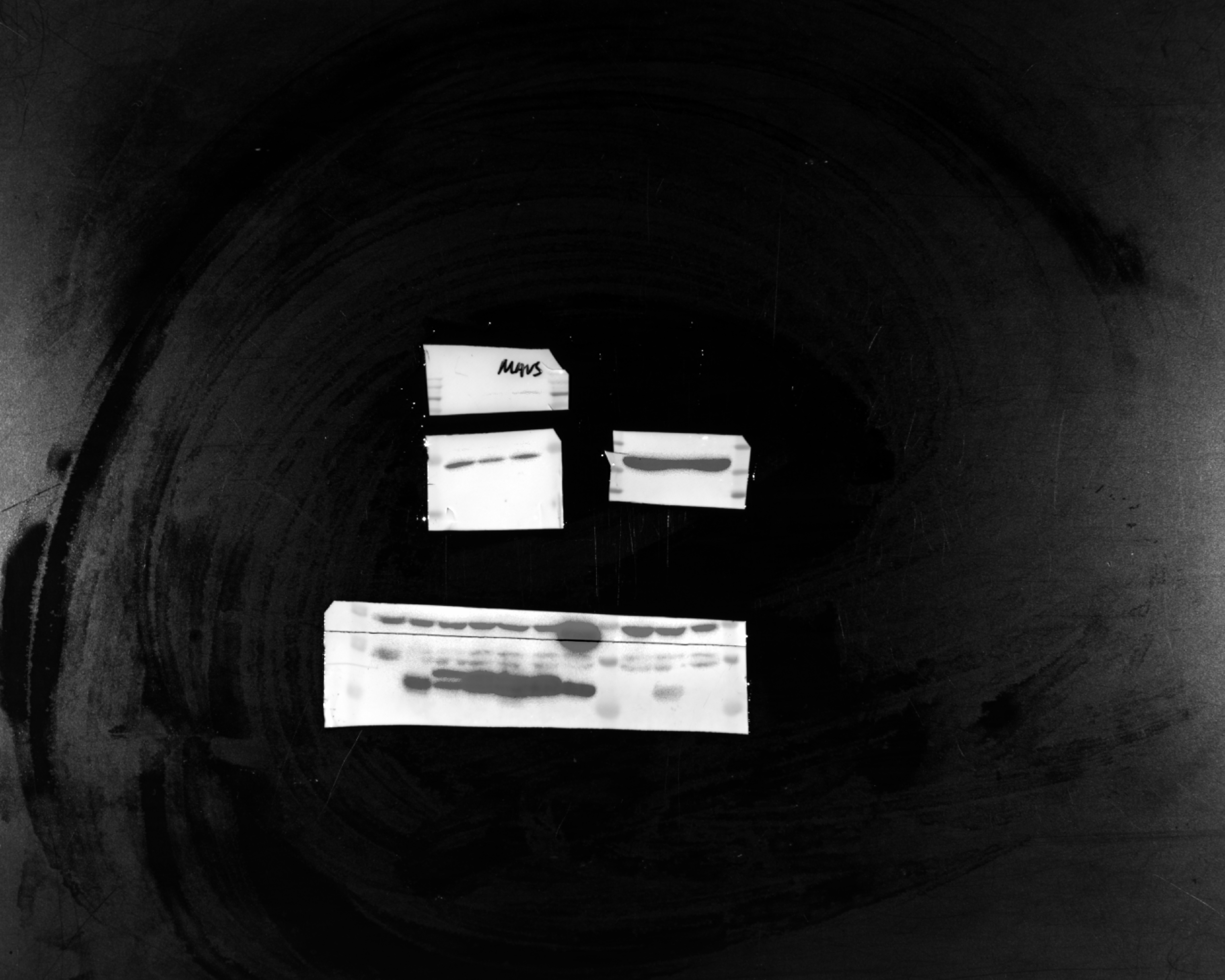

Supplement: Figure 7—figure supplement 1—source data 2. [file elife-101973-fig7-figsupp1-data2.zip › Figure 7–figure supplement 1–source data 2/Figure 7–figure supplement 1B/ORMDL3 and tubulin.jpg]

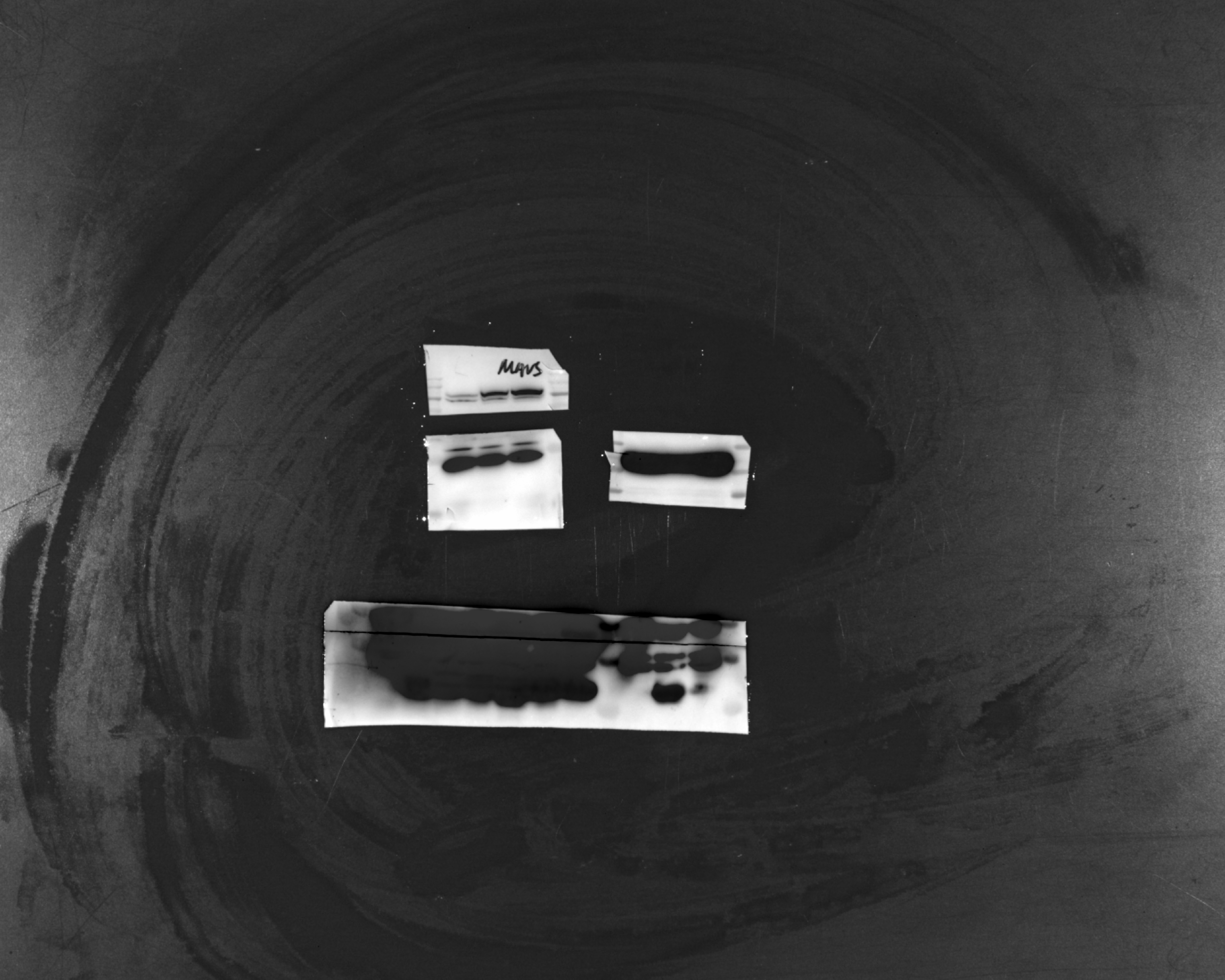

Supplement: Figure 7—figure supplement 1—source data 2. [file elife-101973-fig7-figsupp1-data2.zip › Figure 7–figure supplement 1–source data 2/Figure 7–figure supplement 1B/RIG-I.jpg]

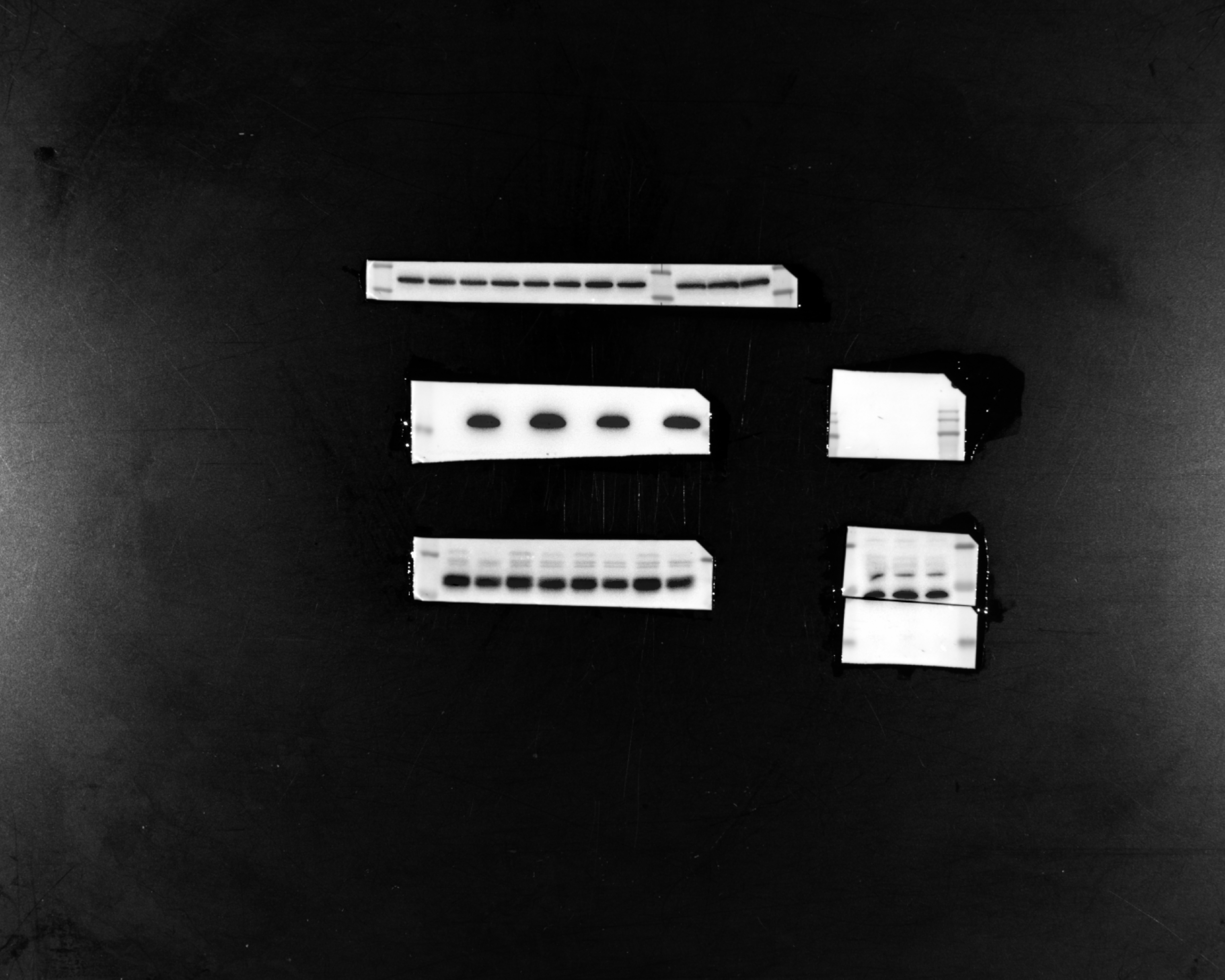

Supplement: Figure 7—figure supplement 1—source data 2. [file elife-101973-fig7-figsupp1-data2.zip › Figure 7–figure supplement 1–source data 2/Figure 7–figure supplement 1D/ORMDL3 and tubulin.jpg]

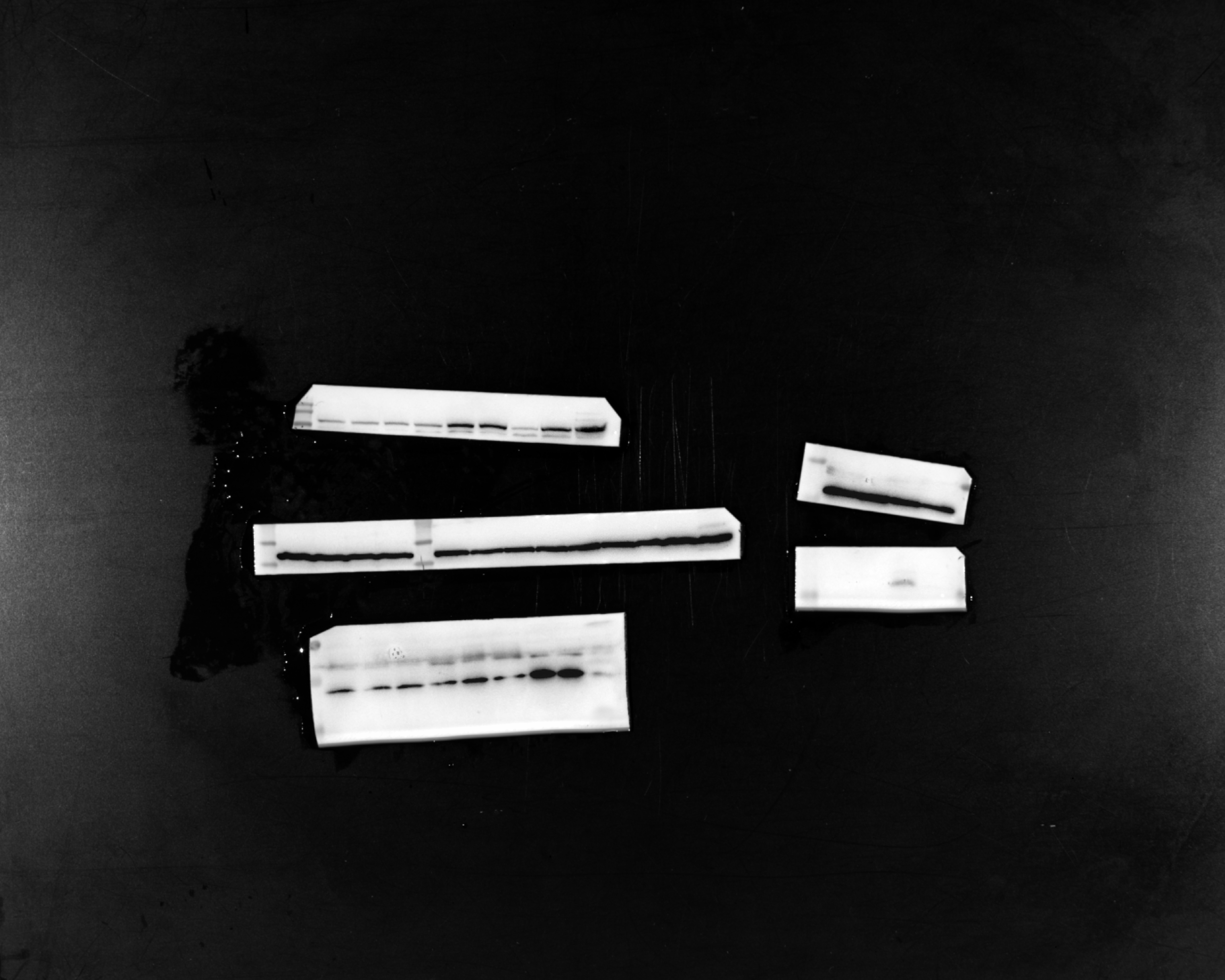

Supplement: Figure 7—figure supplement 1—source data 2. [file elife-101973-fig7-figsupp1-data2.zip › Figure 7–figure supplement 1–source data 2/Figure 7–figure supplement 1D/RIG-I.jpg]

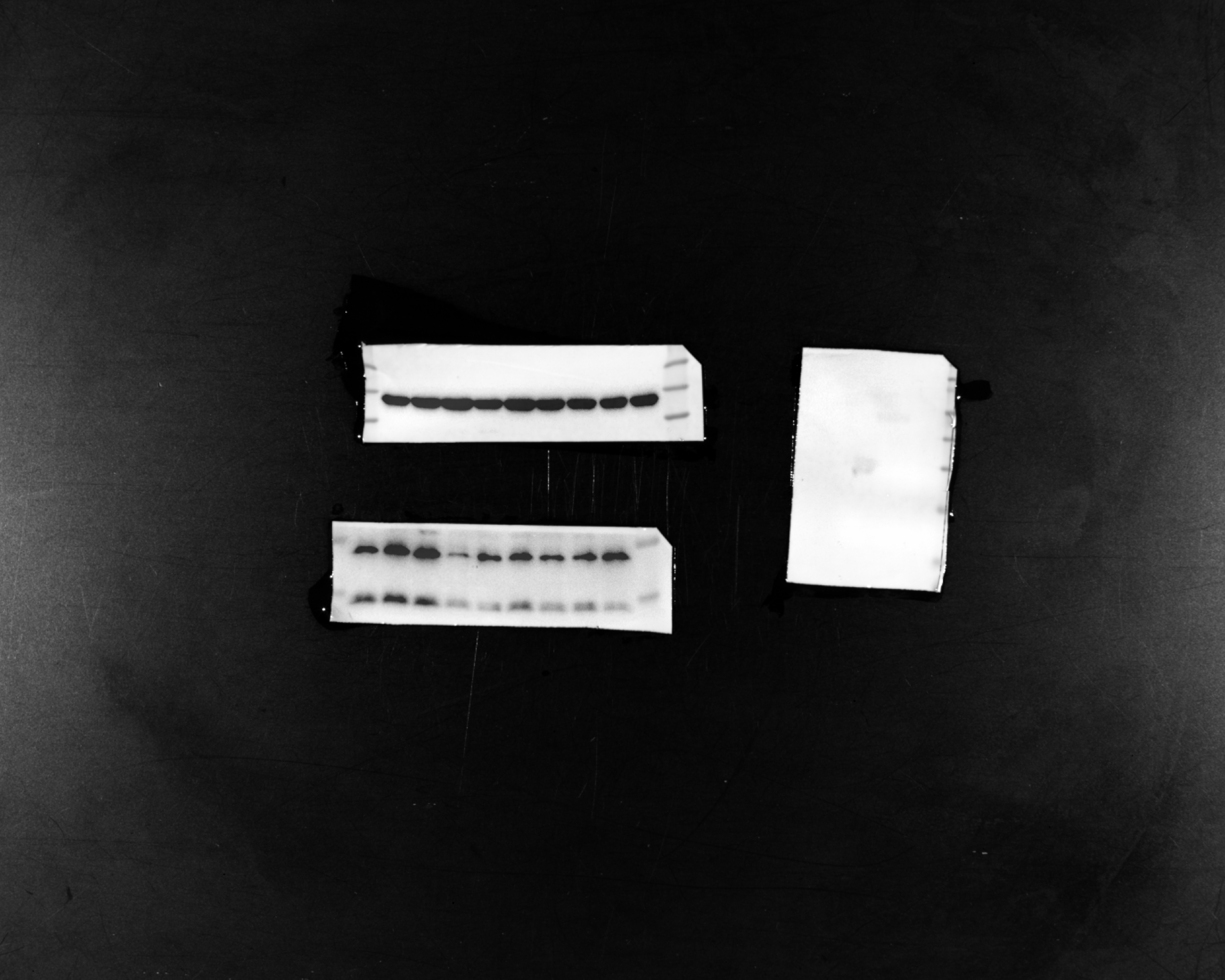

Supplement: Figure 7—figure supplement 1—source data 2. [file elife-101973-fig7-figsupp1-data2.zip › Figure 7–figure supplement 1–source data 2/Figure 7–figure supplement 1E/ORMDL3.jpg]

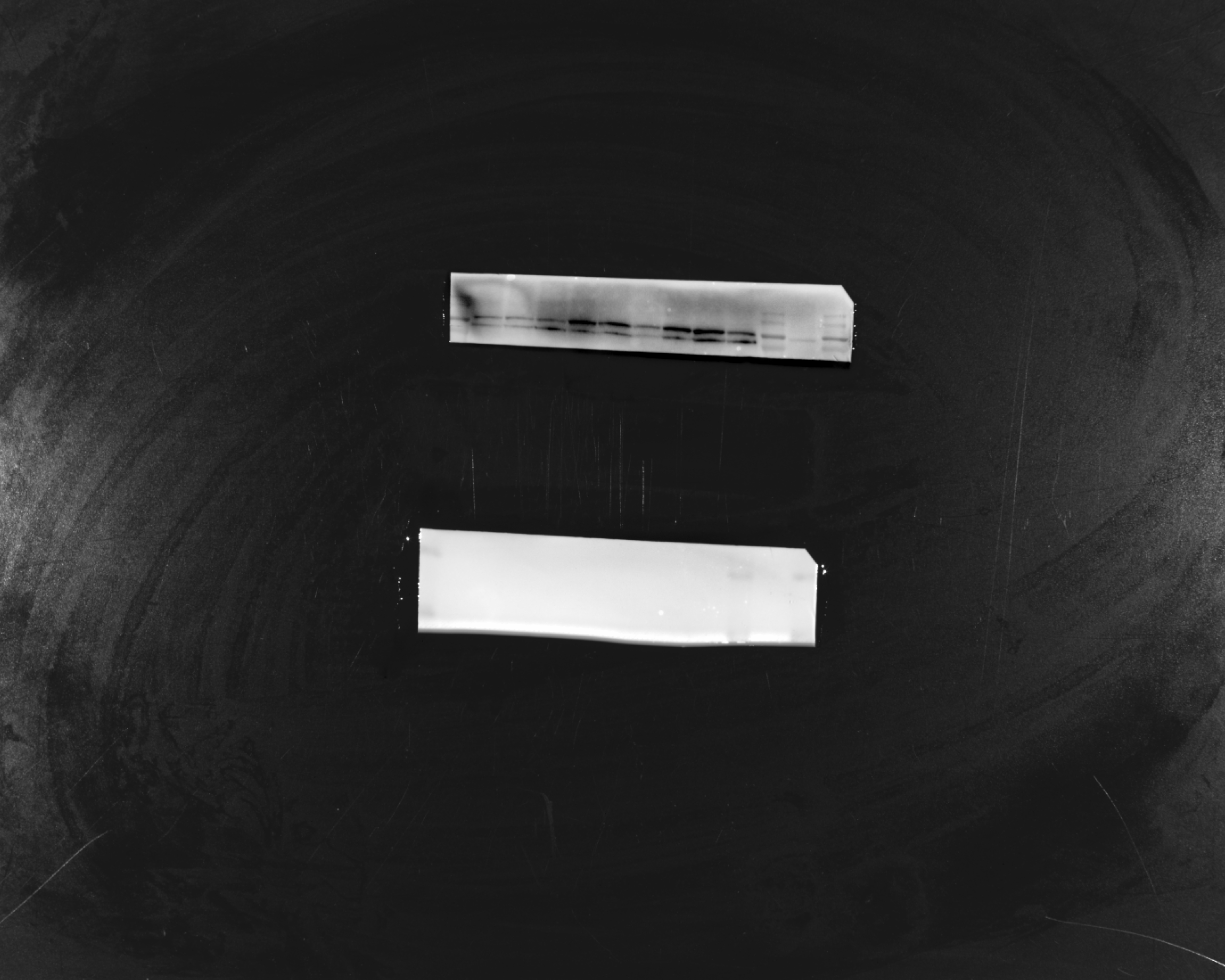

Supplement: Figure 7—figure supplement 1—source data 2. [file elife-101973-fig7-figsupp1-data2.zip › Figure 7–figure supplement 1–source data 2/Figure 7–figure supplement 1E/RIG-I.jpg]

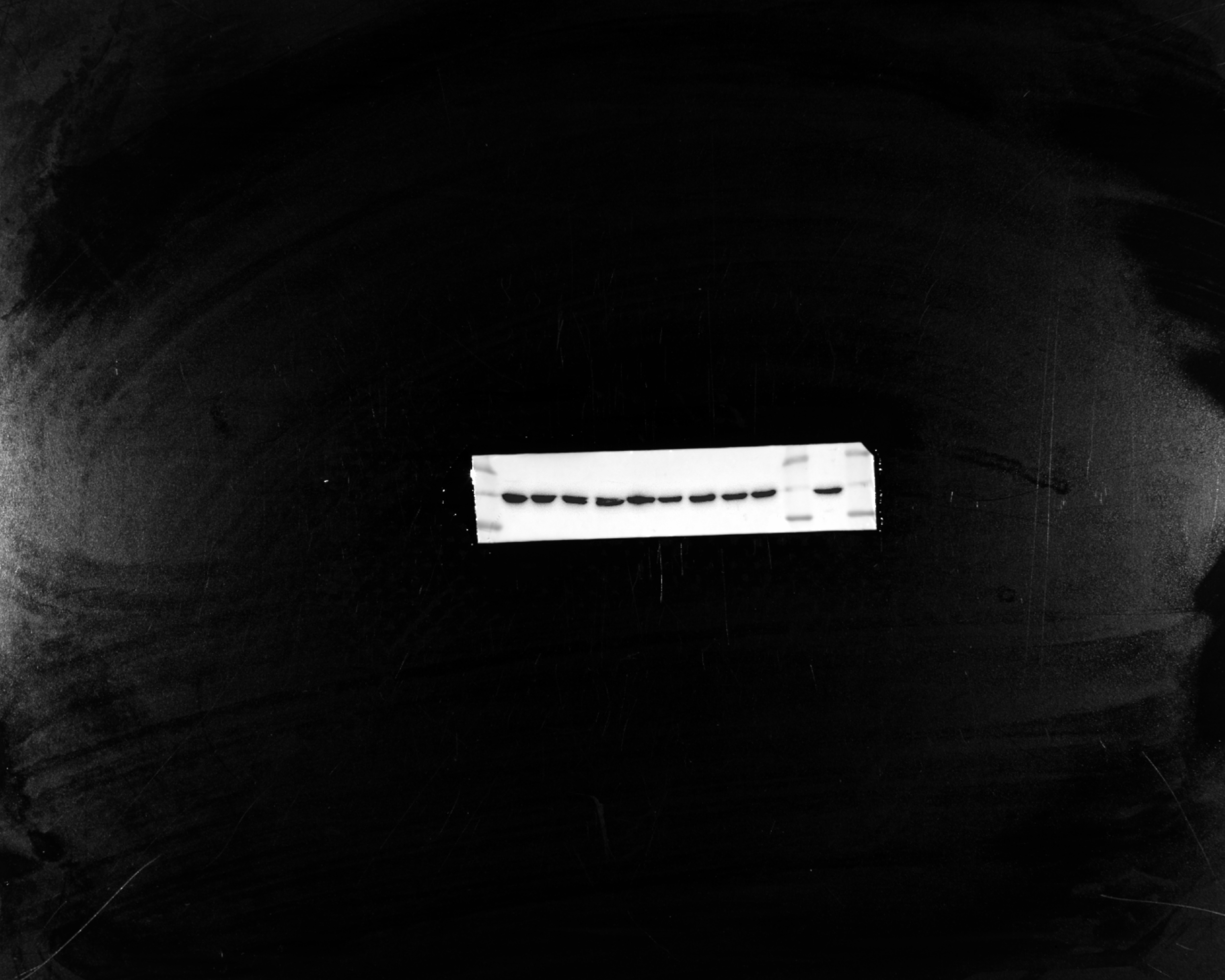

Supplement: Figure 7—figure supplement 1—source data 2. [file elife-101973-fig7-figsupp1-data2.zip › Figure 7–figure supplement 1–source data 2/Figure 7–figure supplement 1E/actin.jpg]
